# Gene dysregulation in acute HIV-1 infection – early transcriptomic analysis reveals the crucial biological functions affected

**DOI:** 10.3389/fcimb.2023.1074847

**Published:** 2023-04-03

**Authors:** Erica Parker, Melinda A. Judge, Lucia Pastor, Laura Fuente-Soro, Chenjerai Jairoce, Kim W. Carter, Denise Anderson, Inácio Mandomando, Holly D. Clifford, Denise Naniche, Peter Neils Le Souëf

**Affiliations:** ^1^ School of Medicine, University of Western Australia, Crawley, WA, Australia; ^2^ ISGlobal, Barcelona Institute for Global Health, Hospital Clinic–Universitat de Barcelona, Barcelona, Spain; ^3^ AIDS Research Institute-IrsiCaixa, Institut Germans Trias i Pujol (IGTP), Hospital Germans Trias i Pujol, Universitat Autonoma de Barcelona, Badalona, Spain; ^4^ Centro de Investigação em Saúde da Manhiça (CISM), Maputo, Mozambique; ^5^ Telethon Kids Institute, Perth, WA, Australia

**Keywords:** HIV, gene expression profiling, sub-Sahara Africa (SSA), Mozambique, acute HIV infection, host microbial interactions, innate immunity

## Abstract

**Introduction:**

Transcriptomic analyses from early human immunodeficiency virus (HIV) infection have the potential to reveal how HIV causes widespread and lasting damage to biological functions, especially in the immune system. Previous studies have been limited by difficulties in obtaining early specimens.

**Methods:**

A hospital symptom-based screening approach was applied in a rural Mozambican setting to enrol patients with suspected acute HIV infection (Fiebig stage I-IV). Blood samples were collected from all those recruited, so that acute cases and contemporaneously recruited, uninfected controls were included. PBMC were isolated and sequenced using RNA-seq. Sample cellular composition was estimated from gene expression data. Differential gene expression analysis was completed, and correlations were determined between viral load and differential gene expression. Biological implications were examined using Cytoscape, gene set enrichment analysis, and enrichment mapping.

**Results:**

Twenty-nine HIV infected subjects one month from presentation and 46 uninfected controls were included in this study. Subjects with acute HIV infection demonstrated profound gene dysregulation, with 6131 (almost 13% of the genome mapped in this study) significantly differentially expressed. Viral load was correlated with 1.6% of dysregulated genes, in particular, highly upregulated genes involved in key cell cycle functions, were correlated with viremia. The most profoundly upregulated biological functions related to cell cycle regulation, in particular, CDCA7 may drive aberrant cell division, promoted by overexpressed E2F family proteins. Also upregulated were DNA repair and replication, microtubule and spindle organization, and immune activation and response. The interferome of acute HIV was characterized by broad activation of interferon-stimulated genes with antiviral functions, most notably IFI27 and OTOF. BCL2 downregulation alongside upregulation of several apoptotic trigger genes and downstream effectors may contribute to cycle arrest and apoptosis. Transmembrane protein 155 (TMEM155) was consistently highly overexpressed during acute infection, with roles hitherto unknown.

**Discussion:**

Our study contributes to a better understanding of the mechanisms of early HIV-induced immune damage. These findings have the potential to lead to new earlier interventions that improve outcomes.

## Introduction

Acute HIV infection encompasses the period after HIV transmission and prior to seroconversion, typically 3-12 weeks (Fiebig et al., 2003), following an initial 8-10 day undetectable eclipse period (Konrad et al., 2017). The rate and severity of acute infection may be predictive of disease progression and long-term sequelae, reflecting the extent of immune damage and hyperinflammatory state during this time (Lavreys et al., 2006). Furthermore, the early failure of immune responses to curb the viral threat and the latent viral reservoirs established during the acute stage set the challenging environment in which researchers seek a therapeutic vaccine and cure (Johnston and Fauci, 2008; Davenport et al., 2019).

Enhanced understanding of the pathogenesis of acute HIV infection may offer insights toward personalized therapies, vaccine development or curative treatment (Chun et al., 2015). Additionally, diagnostic testing that enables earlier detection may reduce onward transmission during this time of heightened risk due to peak viremia (Pilcher et al., 2004; Wawer et al., 2005; Powers et al., 2011). Transcriptomic analyses can pinpoint specific molecular mechanisms and pathways altered by HIV. We recently reviewed research of gene expression during HIV infection, and this revealed that studies to date have provided fragmentary insight into the host response to HIV, with conclusions limited by small sample sizes, and variation in study designs and data reporting (Judge et al., 2020). Case-control study designs comparing individuals with HIV to healthy controls were a common approach to describing major transcriptomic changes attributable to infection. Despite acknowledgement that gene expression changes are most profound during acute infection (Li et al., 2009; Judge et al., 2020), and the potential benefits of such research, relevant information is scarce, largely due to the well-described clinical and resource-intensive screening challenges of identifying individuals with acute infection (Cohen et al., 2010; Miller et al., 2010).

We identified only four published studies examining human host gene expression during acute HIV infection. Li et al. (2009) used microarray to describe stage-specific changes in gene expression in inguinal lymphoid tissue, with greatest dysregulation observed during acute infection (n=9 samples with 358 differentially expressed genes (DEG) identified). Zhang et al. used RNA-seq and compared peripheral blood mononuclear cells (PBMC) from 3 early HIV infection subjects to 3 uninfected controls and also identified many DEG (n=2049) (Zhang et al., 2018). Hyrcza et al. reported transcriptomic changes in CD4+ and CD8+ T cells from 5 subjects with early infection and 10 with longer-term infection, and found that some changes persisted during chronic infection (Hyrcza et al., 2007). Unsurprisingly, dysregulation centred on immune activation, innate immune defenses, adaptive immunity (Li et al., 2009; Zhang et al., 2018) and interferon-stimulated genes (Hyrcza et al., 2007). A fourth paper, by Kazer et al. provides a detailed description of gene modules in PBMC of 4 individuals with acute HIV, but the focus was more on temporal changes in module weighting over time, rather than describing differential gene expression against pre-infection samples (Kazer et al., 2020). Although these studies offered key insights, conclusions are hampered by variable definitions of early infection (with sampling up to several months post-transmission), small sample sizes, varied tissue types, use of superseded technologies, and participant bias in terms of sex, geographical location, and HIV subtypes.

This study aimed to use gold-standard RNA-seq transcriptomic profiling to describe the human host response to acute HIV infection, using PBMC from a large cohort of recently infected individuals as compared with HIV-negative controls. Importantly, the study aimed to recruit both men and women from Eastern and Southern Africa (ESA) where HIV-1 subtype C is predominant, reflective of the generalized epidemic. We hypothesized that examining a cohort early in HIV infection would reveal a consistent pattern of gene dysregulation among crucial biological functions, and highlight early “gateway” paths and processes leading to known HIV pathologies.

## Materials and methods

### Human ethics approval

This research was approved by ethical review boards at Barcelona Clinic Hospital (2011/6264), the Ministry of Health of Mozambique (461/CNBS/12) and the University of Western Australia (2019/RA/4/1/6296). Written informed consent was obtained from all subjects.

### Sample collection

The present analysis is a sub-study of the Gastrointestinal biomarkers in Acute HIV-infected Mozambican Adults (GAMA) study. Procedures of recruitment for this cohort study have been described elsewhere (Pastor et al., 2017a; Pastor et al., 2017b). In brief, the cohort was enrolled in a semi-rural area of Southern Mozambique with an HIV prevalence of 39.9% (Gonzalez et al., 2012). Patients presenting with febrile illness or for voluntary HIV testing were invited to be screened for study eligibility using antibody-based fingerprick HIV rapid tests as per national diagnostic guidelines (Determine HIV 1/2 (Abbott Laboratories, Illinois) and Uni-Gold (Trinity Biotech Co., Ireland)). Those testing negative or indeterminate had HIV viral load measured *via* RT-PCR (Abbott RealTime HIV-1 assay, Illinois). Viral load positive, seronegative or indeterminate patients were diagnosed with acute HIV and invited for follow-up four weeks later. Time-matched HIV-uninfected controls were selected from patients who tested negative on rapid tests and viral load PCR, and also invited back four weeks later.

At this follow-up visit, clinical data and specimens for immunological and microbiological evaluation were collected. The Manhiça Health Research Centre (CISM) is co-located with the Manhiça District Hospital and contains a well-equipped clinical research laboratory (Sacoor et al., 2013). PBMC were isolated from peripheral blood on the day of collection, stored in RNAprotect (Qiagen, Germany) at -80°C, then shipped to Western Australia on dry ice.

### Fiebig staging

HIV-specific antibodies were analysed by Western blot assay using INNO-LIA HIV I/II Score (Innogenetics, Belgium). Staging of primary HIV infection was completed according to Fiebig and colleagues, 2003 (Fiebig et al., 2003). Date since infection was estimated by adding the mean time since infection for those in Fiebig stages I-IV (Fiebig et al., 2003) to the number of days between recruitment and sample collection.

### T-cell and plasma protein determinations

As previously described (Pastor et al., 2017b), CD4+ and CD8+ T cell counts were determined using CD3, CD8, CD4 and CD45 fluorochrome-labelled antibodies on fresh whole blood using Trucount tubes and FaCSCalibur flow cytometry (BD Biosciences, New Jersey). Cytokine levels in plasma were determined using enzyme-linked immunosorbent assay (Orgentec, Germany; Hycult Biotech, Pennsylvania; and Immunodiagnostik, Germany) and Luminex multianalyte profiling: Human Cytokine Magnetic 30-plex panel (Invitrogen, California), Bio-Plex Pro Human Th17 cytokine assay (Biorad, California), and Human Magnetic Luminex Screening Assay (R&D, Minnesota) (Pastor et al., 2017a).

### Subtyping

Previous work has demonstrated that circulating virus in Mozambique is almost exclusively HIV-1 Group M subtype C (Engelbrecht et al., 1998; Bellocchi et al., 2005; Parreira et al., 2006). One acute HIV case was the subject of a published case report (Velasco et al., 2015) and had sequencing completed to confirm subtype C.

### RNA extraction

RNA was extracted using a modified protocol for the RNeasy MinElute Clean-up kit (Qiagen, Germany). Briefly, PBMC pellets were resuspended in 1ml of TRIzol, shaken vigorously, 200µl of chloroform was added and shaken again. After centrifugation at 14000xg for 5 minutes at 4°C, the aqueous phase was added to an equal volume of 70% ethanol. After mixing by pipetting, samples were transferred to an RNeasy MinElute spin column and centrifuged at 800xg for 30 seconds. The columns were washed with 500µl of RPE buffer by centrifugation at 8000xg for 30 seconds, then the process was repeated using 80% ethanol. An additional 500µl of 80% ethanol was added and centrifuged at 8000xg for 2 minutes. Once the flow through was discarded, samples were again centrifuged at 8000xg for 5 minutes. To elute the RNA, 20µl of RNase-free water was pipetted directly onto the column membrane and stood for 1 minute prior to centrifugation at maximum speed for 2 minutes. Quality control was performed using nanodrop (Thermo Fisher Scientific, Massachusetts) and Bioanalyzer (Agilent, California); samples with a minimum concentration of 20ng/μl (total input 250ng) and an RNA integrity number ≥ 8 were sequenced.

### RNA-seq

Illumina 50 base-pair, single-end RNA-seq was completed, using HiSeq2000 at the Australian Genome Research Facility, with minimum 20 million reads/sample. Read quality was assessed using FastQC. Reads were aligned to human reference genome hg19 using HISAT. Alignment quality was assessed using SAMStat. Quantification and organization of read counts was performed using summarizeOverlaps (*GenomicAlignments* package) and voom (*limma* package), both from Bioconductor. Sequencing data was functionally validated against protein levels in plasma. Data was deposited in Gene Expression Omnibus (GSE199911).

### Statistical analysis

Sample cellular composition was estimated from gene expression data using CIBERSORT, an in silico gene expression deconvolution tool. Estimates of CD4+ and CD8+ T cell counts from CIBERSORT were examined for consistency against flow cytometry counts (Spearman’s rank order correlation). Cohort descriptive statistics were completed in R 3.4.2. Independent samples T-tests were used to compare parametric data, Mann-Whitney U-tests and Spearman’s rank order correlations were used for non-parametric data, and Chi-square or Fisher’s exact tests were used for categorical data. Comparison of acute HIV-infected versus uninfected control clinical data was performed using Spearman’s rank order correlation, with statistical significance at p<0.05.

Differential gene expression analysis was completed using TopHat and Cufflinks (Trapnell et al., 2012). Briefly, reads were aligned by TopHat (using the BowTie engine), and transcripts were assembled using Cufflinks. The latter involved analysis of immature transcriptomes and final transcriptome assembly with Cuffmerge; mapping of reads with Cuffdiff to quantify genes and transcripts and determine statistical significance of gene expression between samples; comparison with reference databases using Cuffcompare to determine presence of novel genes and transcripts; and management, visualization and integration of Cuffdiff data with CummeRbund. Data were exported to R for further analyses. Adjusted p values were calculated by Benjamini-Hochberg false discovery correction (5%). Genes with adjusted p values <0.05 were considered differentially expressed.

Differential gene expression was adjusted for participant age and sex, as well as RNA extraction year (a proxy for two individual extractors) to mitigate plausible batch effect, using an empirical Bayes framework. Viral load correlations were assessed using Spearman correlation with FDR adjustment (5%) using the psych package in R. Network Analyst generated a heatmap of raw expression counts. Cytoscape v3.8.1 and v3.9.1 were used to visualize known interactions between DEG, and between DEG and upstream regulators, using the STRING database (Szklarczyk et al., 2021) and CyTargetFinder with the ENCODE transcription factor database. Gene Set Enrichment Analysis (GSEA) was completed in GSEA 4.1.0, using a list of DEG ranked by the negative Log_10_ of adjusted p value divided by sign of the fold change (Ziemann, 2014; Dębski et al., 2016), with identifiers from Human_ENSEMBL_Gene_ID_MSigDB.v7.0.chip and gene sets from the Gene Ontology database (c5.go.bp.v7.2.symbols.gmt). Enrichment maps were created in Cytoscape v3.8.1 using GSEA results, with clusters generated using Community Cluster (GLay) Annotation Set in the AutoAnnotate plugin, with minor manual adjustments to improve language.

## Results

### Study population

**Figure 1:**
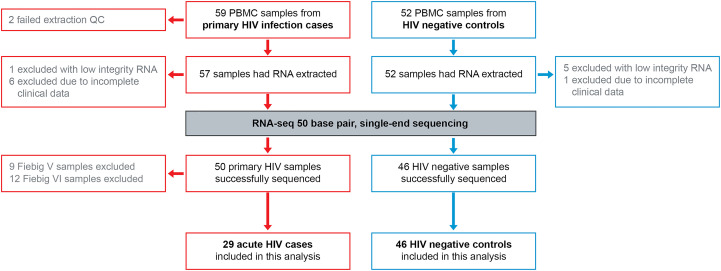
RNA-seq 50 base pair single-end sequencing was completed for PMBC from acute HIV cases and uninfected controls. PBMC, peripheral blood mononuclear cells; RNA, ribonucleic acid; QC, quality control.

Primary HIV cases (n=85) and uninfected controls (n=58) were recruited among adults aged 18-60 of both sexes, with ethnicities comprised from Indigenous African subgroups. From these subjects, 59 cases and 52 controls returned for a clinical follow-up visit one month later, during which PBMC samples were collected for gene expression analyses. RNA sequencing was completed satisfactorily for 50 primary HIV samples and 46 HIV negative samples (**Figure 1**) with an average 23 million reads/sample, and 80% mapping accuracy to human reference genome hg19. To examine batch effect, three samples were sequenced on both RNA-seq runs, with highly similar results between batches (**Supplementary Figure 1**). Given our objective was to study the earliest stages of infection, further down-selection of cases was completed based on Fiebig staging at enrolment (Fiebig et al., 2003), with only the n=29 most acute cases (Fiebig I-IV) included in further analyses.

At PBMC collection, participants enrolled as acute HIV cases had an estimated median time since infection of 8.0 weeks (range 5.9-10.6 weeks) and 83% of cases tested positive on HIV point-of-care rapid tests [which have median window periods of 19 and 32 days, respectively (Delaney et al., 2017)], while the remainder tested negative or indeterminate. While these rapid tests cannot confirm full seroconversion, they indicate initiation of an adaptive immune response among most cases. Median Log_10_ HIV viral load at PBMC collection was 5.03 (IQR 4.48-5.49) copies/mL; viral load at recruitment one month earlier had been higher, at median Log_10_ 6.92 (IQR 6.15-7.52) copies/mL. CD4+ T cell count, and CD4+/CD8+ ratio were significantly lower in cases compared with controls (**Table 1**); there were no other significant differences between groups.

**Table 1:** Characteristics of study population one month from presentation.

Characteristic	Acute HIV cases (n=29)	HIV negative controls (n=46)	P value
Sex, n (%) female	18 (62.1)	35 (76.1)	0.194
Age, median (IQR)	27.0 (19.0-35.0)	25.0 (20.7-35.5)	0.699
CD4 T cell count (flow cytometry), median (IQR)	599.0 (440.0-700.5)	995.5 (764.5-1156.0)	<0.001 ***
CD4:CD8 ratio (flow cytometry), median (IQR)	0.50 (0.27-0.69)	1.62 (1.24-2.36)	<0.001 ***
Fever in previous 24 hours (self-reported), n (%)	2 (6.9)	3 (6.5)	0.726
Diarrhoea in previous week (self-reported), n (%)	2 (6.9)	3 (6.5)	0.726
Malaria in previous month (self-reported), n (%)	5 (19.2)	9 (20.0)	0.308
Hepatitis B, n (%)	4 (14.3)	2 (4.3)	0.191
Syphilis, n (%)	2 (6.9)	3 (6.8)	0.990
*Entamoeba histolytica*, n (%)	1 (3.6)	3 (6.8)	0.826
*Giardia lamblia*, n (%)	2 (7.1)	5 (11.4)	0.083
*Cryptosporidium* spp., n (%)	0 (0.0)	0 (0.0)	NA
*Clostridium difficile*, n (%)	0 (0.0)	1 (2.3)	0.655
*Strongyloides stercoralis*, n (%)	1 (0.0)	1 (2.2)	0.424
Pregnancy, n (%)	1 (3.4)	0 (0.0)	0.259

***p < 0.001.

### Differential gene expression in acute HIV

**Figure 2:**
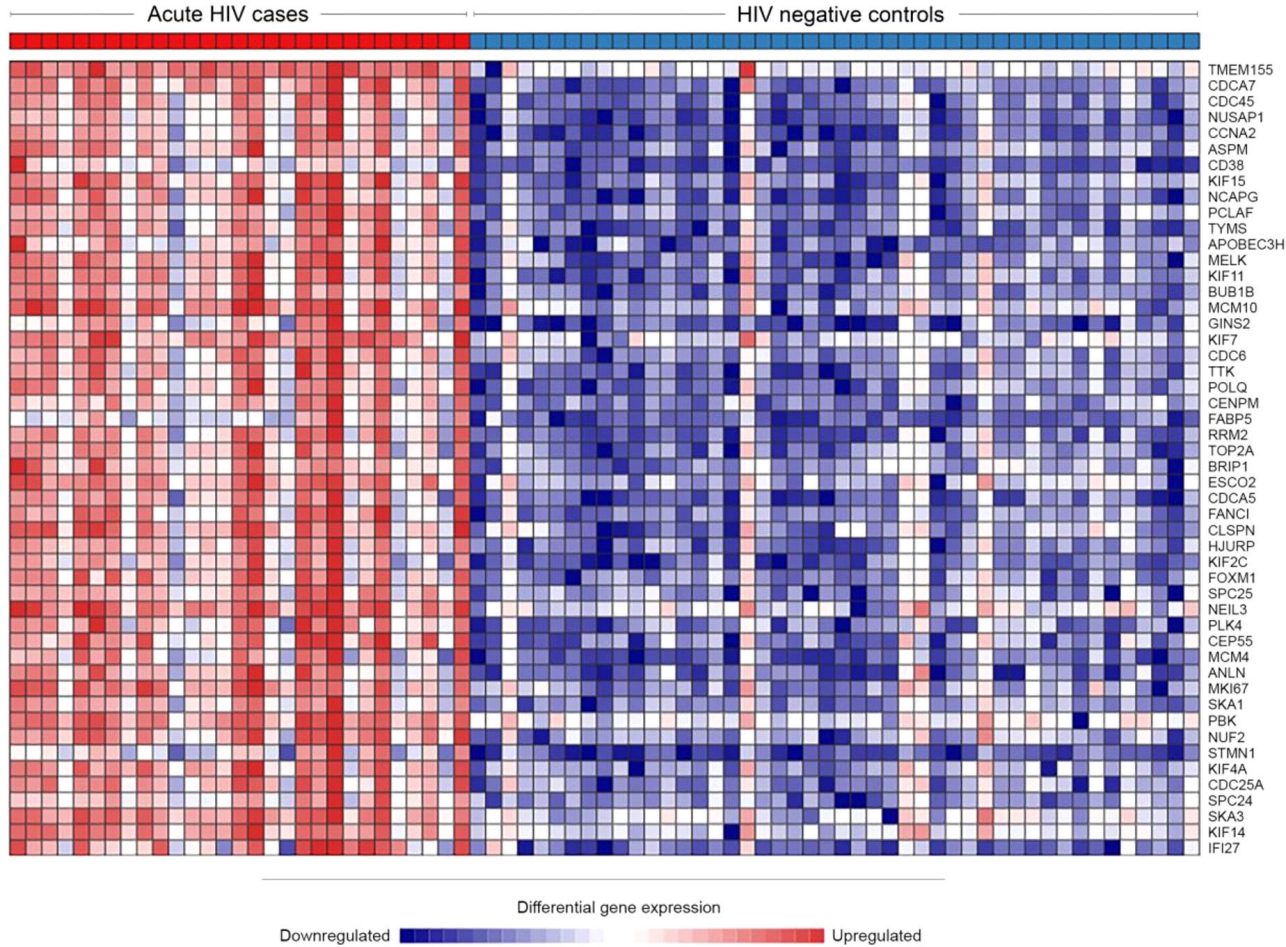
Heatmap of the top 50 most upregulated genes during HIV infection, demonstrating high consistency between individuals. Network Analyst was used to view a heatmap of the raw expression counts for each acutely infected (n=29) and HIV negative (n=46) individual. The top 50 most significantly DEGs were all upregulated during acute HIV.

Of 47,971 mapped genes, there were 6131 (12.8%) genes significantly differentially expressed during acute HIV-1 infection. Of these, 3307 genes (6.9%) were upregulated and 2824 (5.9%) were downregulated. Such high numbers of DEG have been previously described for other acute infections, such as influenza (Zhai et al., 2015), dengue (Popper et al., 2012), and tuberculosis (Wang et al., 2018). The top 50 DEG by significance are presented in **Table 2** (full list in **Supplementary Table 1**). Notably, upregulated genes tended to have greater significance, with the first downregulated gene appearing at position 123 when ranked by adjusted p value. Upregulation was also more profound than downregulation, with 221 genes upregulated to Log_2_ fold change >1.5 (the highest at Log_2_4.84) compared with 10 genes downregulated to Log_2_ fold change <-1.5 (the lowest at Log_2_-1.89). The top 50 DEG were highly consistent among cases and controls, respectively (**Figure 2**).

**Table 2:** The top 50 genes differentially expressed during acute HIV infection, eight of which were positively correlated with viral load.

	Gene name	HGNC	Log_2_ fold change	adjusted P value	viral load correlation coefficient	viral load correlation adj P value
**1**	transmembrane protein 155	TMEM155	3.267514	4.46E-35		
**2**	cell division cycle associated 7	CDCA7	2.47209	9.42E-34	0.619**	0.038*
**3**	nucleolar and spindle associated protein 1	NUSAP1	1.979959	1.08E-26		
**4**	KIAA0101	KIAA0101	2.784338	3.2E-26	0.589**	0.048*
**5**	thymidylate synthetase	TYMS	2.473498	3.2E-26		
**6**	non-SMC condensin I complex, subunit G	NCAPG	2.334234	3.2E-26		
**7**	cell division cycle 45	CDC45	2.836749	1.12E-25	0.594**	0.048*
**8**	CD8b molecule pseudogene	CD8BP	1.570053	1.12E-25		
**9**	cyclin A2	CCNA2	2.321098	3.88E-25	0.627**	0.034*
**10**	denticleless E3 ubiquitin protein ligase homolog (Drosophila)	DTL	2.886901	6.18E-25		
**11**	maternal embryonic leucine zipper kinase	MELK	2.489211	6.18E-25		
**12**	kinesin family member 11	KIF11	2.168095	6.18E-25		
**13**	CD8b molecule	CD8B	1.50107	3.77E-24		
**14**	BUB1 mitotic checkpoint serine/threonine kinase B	BUB1B	2.214816	6.3E-24		
**15**	myosin, light chain 6B, alkali, smooth muscle and non-muscle	MYL6B	1.562934	6.3E-24		
**16**	marker of proliferation Ki-67	MKI67	2.577557	6.65E-24		
**17**	anillin, actin binding protein	ANLN	2.351843	1.37E-23		
**18**	chemokine (C-C motif) ligand 5	CCL5	1.389217	1.54E-23		
**19**	ribonucleotide reductase M2	RRM2	2.648566	1.78E-23		
**20**	PDZ binding kinase	PBK	2.758444	1.99E-23		
**21**	ubiquitin-like with PHD and ring finger domains 1	UHRF1	1.923329	2.07E-23		
**22**	claspin	CLSPN	2.304573	2.77E-23		
**23**	cell division cycle 6	CDC6	2.411704	4.19E-23		
**24**	minichromosome maintenance complex component 10	MCM10	2.817904	5.57E-23		
**25**	cell division cycle associated 5	CDCA5	2.485974	1E-22		
**26**	topoisomerase (DNA) II alpha 170kDa	TOP2A	2.114054	1E-22		
**27**	apolipoprotein B mRNA editing enzyme, catalytic polypeptide-like 3H	APOBEC3H	1.665933	1E-22		
**28**	fatty acid binding protein 5 (psoriasis-associated)	FABP5	1.642369	1E-22		
**29**	kinesin family member 15	KIF15	2.38185	1.25E-22	0.663**	0.027*
**30**	asp (abnormal spindle) homolog, microcephaly associated (Drosophila)	ASPM	2.263024	1.57E-22		
**31**	BRCA1 interacting protein C-terminal helicase 1	BRIP1	1.402205	1.97E-22		
**32**	spindle and kinetochore associated complex subunit 3	SKA3	2.784406	2.39E-22	0.612**	0.042*
**33**	GINS complex subunit 2 (Psf2 homolog)	GINS2	2.33612	4.25E-22		
**34**	kinesin family member 7	KIF7	2.730583	4.96E-22		
**35**	E2F transcription factor 8	E2F8	2.679807	4.96E-22		
**36**	exonuclease 1	EXO1	2.342272	4.96E-22		
**37**	kinesin family member 2C	KIF2C	2.156903	4.96E-22		
**38**	polymerase (DNA directed), theta	POLQ	2.096836	4.96E-22		
**39**	minichromosome maintenance complex component 4	MCM4	1.709862	4.96E-22	0.588**	0.049*
**40**	TPX2, microtubule-associated	TPX2	2.327686	6.45E-22		
**41**	solute carrier family 27 (fatty acid transporter), member 2	SLC27A2	2.142166	8.65E-22		
**42**	cell division cycle 25A	CDC25A	2.626844	9.19E-22		
**43**	kinesin family member C1	KIFC1	2.118952	1.23E-21		
**44**	establishment of sister chromatid cohesion N-acetyltransferase 2	ESCO2	2.401353	1.63E-21	0.654**	0.028*
**45**	E2F transcription factor 7	E2F7	2.296616	1.73E-21		
**46**	centromere protein M	CENPM	1.953828	3.66E-21		
**47**	nei endonuclease VIII-like 3 (E. coli)	NEIL3	2.569431	4.14E-21		
**48**	CD8a molecule	CD8A	1.587898	4.22E-21		
**49**	MIR4435-1 host gene (non-protein coding)	MIR4435-1HG	1.136808	4.22E-21		
**50**	RAD54-like (S. cerevisiae)	RAD54L	1.814108	5.04E-21		

HGNC, HUGO Gene Nomenclature Committee; adjusted P value = Benjamini-Hochberg false discovery rate adjusted p-value.; viral load correlation coefficient = Spearman coefficient (only shown when also significant) * = 0.25 - <0.5 (fair); ** = 0.5 - <0.75 (moderate); viral load correlation adj P value = significance adjusted for FDR, *p<0.05; **p<0.01.

### Differential gene expression correlation with viral load

**Figure 3:**
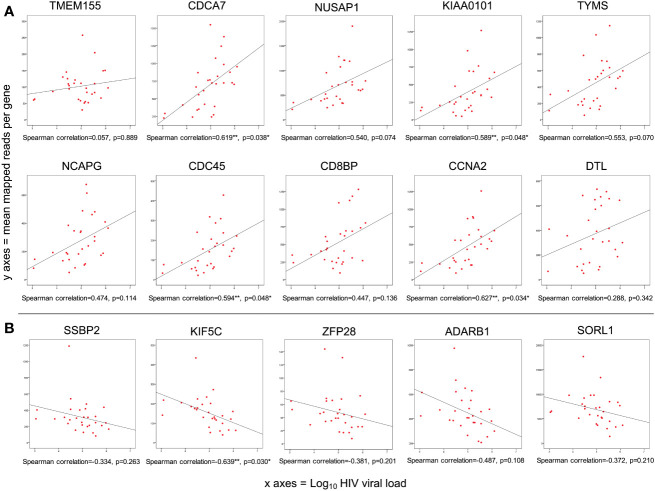
Of genes differentially expressed during acute HIV infection, 1.6% were correlated with viral load. **(A)** Spearman correlation of Log_10_ HIV viral load with mean mapped reads of the top ten upregulated genes; **(B)** Spearman correlation of Log_10_ HIV viral load with mean mapped reads of the top five downregulated genes. p<0.05; **p<0.01; ***p<0.001 (FDR adjusted).

The correlation between gene expression and Log_10_HIV viral load was examined for the 29 acute HIV cases, with FDR adjustment for multiple comparisons (α=0.05). Among the top 50 DEG, 42 showed positive association with viral load (8 significantly correlated). None were negatively correlated. The FDR-adjusted correlation of viral load with the top ten upregulated and top five downregulated genes is depicted in **Figure 3**.

**Figure 4:**
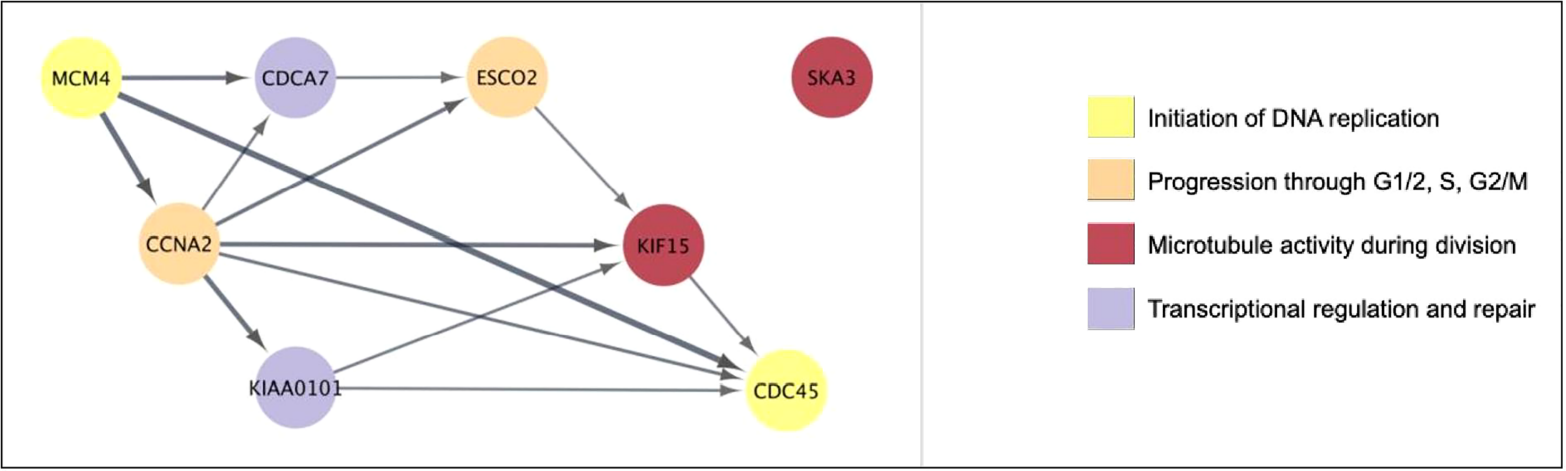
The eight genes that are both highly overexpressed during acute HIV and correlated with viral load have core cell cycle functions. Generated using Cytoscape. Nodes were colored according to broad function as per the legend. Edges represent known protein-protein interactions in the STRING database, with the arrow indicating direction of interaction (source to target), and the weight indicating confidence of association (minimum 80%).

When all 6131 DEG were examined, 51 genes (0.85%) showed significant positive correlation with viral load, while 50 genes (0.82%) showed significant negative correlation (i.e. 1.67% of all DEG correlate with viral load, **Supplementary Table 2**). As mentioned, eight of the genes positively correlated with viral load were also in the Top 50 over-expressed DEG by fold change (**Table 2**). With the exception of SKA3, these eight genes have established protein-protein interactions (**Figure 4**), with protein activities implying that viral load is associated with numerous core functions across the cell cycle process. Specifically, these core functions include initiation of DNA replication [via MCM4-led increased DNA helicase unwinding (Bochman and Schwacha, 2009), and loading of DNA polymerase alpha onto chromatin through CDC45 and possibly GINS complex activity (Bochman and Schwacha, 2009)]; and positive regulation of progression through G1, S and G2 phases through CCNA2 (Jiang et al., 2022). Notably, CCNA2 has been associated with aberrant cell cycle progression in several types of cancer, and has been posited as a potential therapeutic target that could be inhibited to repress cell proliferation (Ma, 2019; Yang et al., 2020). Heightened viral load may also be associated with sister chromatid cohesion during S phase *via* ESCO2 (Bender et al., 2020); increased microtubule activity [via KIF15-led motor activity and spindle assembly (Tanenbaum et al., 2009), and attachment to kinetochores during mitosis *via* SKA3 (Abad et al., 2016)], as well as functions in DNA repair and transcriptional regulation (CDCA7 and KIAA0101) (Gill et al., 2013; Zhang et al., 2021). CDCA7 is notable as a Myc-sensitive gene, through which apoptotic pathways and cellular proliferation can be regulated (Gill et al., 2013; Jimenez et al., 2018).

The genes that demonstrated the strongest negative correlation with viral load (**Supplementary Table 2**) also have key functional overlaps. For example, PTPN13, SFMBT1, ARMCX1, ZNF10, and ZBTB18 are all involved in repression of transcription (Huang et al., 2008; Nishitsuji et al., 2015; Liu et al., 2020; Heng et al., 2022; Xie et al., 2022). Downregulation of ZNF10 during high viremia is noteworthy as this protein has demonstrated functional capacity to limit HIV-1 gene expression *in vitro* (Nishitsuji et al., 2015). Several negatively correlated genes, including FGF9, ARMCX1, PMP22, and ZBTB18 are involved in nervous system functions, such as glial cell growth, axon regeneration, myelin development and brain tumour suppression (Li et al., 2013; Cartoni et al., 2016; Deng et al., 2018; Heng et al., 2022). High viral load may therefore be associated with inhibition of actors that regulate transcription and protect both the central and peripheral nervous systems.

### Cell populations

**Figure 5:**
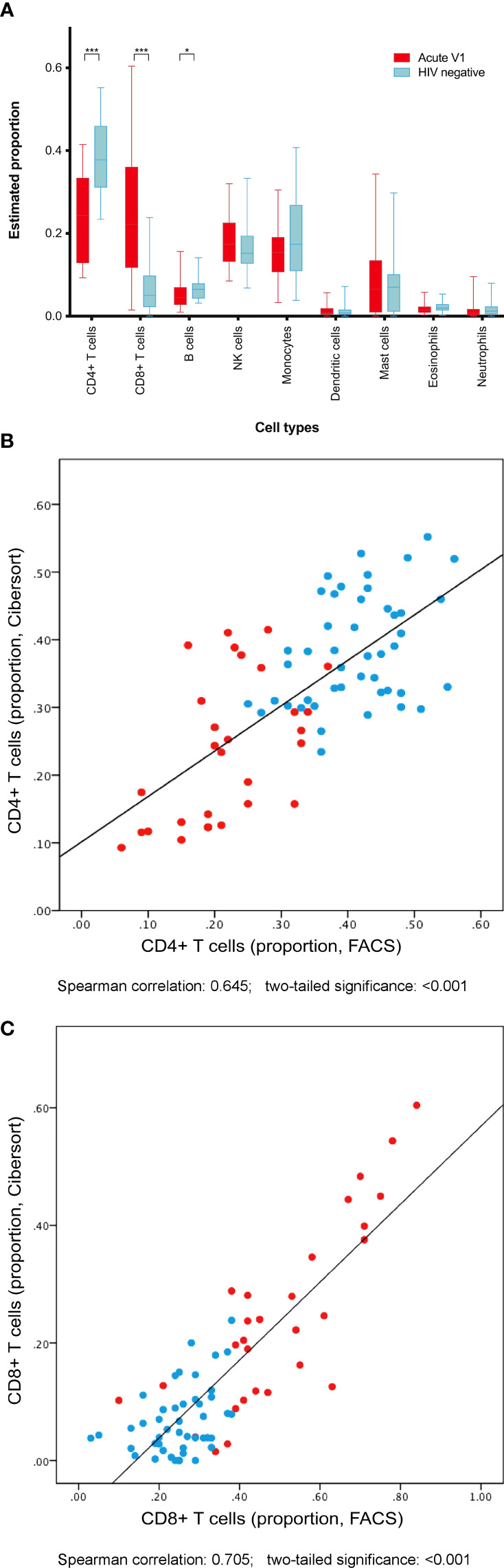
Cell populations among PBMC were predicted by interrogation of differential gene expression between acute HIV cases and uninfected controls. **(A)** Acute HIV was associated with predictable declines in CD4+ T cell and B cell populations, and expansion of CD8+ T cells. Both CD4+ T cell counts **(B)** and CD8+ T cell counts **(C)** estimated using gene expression data correlated well with cell counts *via* flow cytometry. *p<0.05. ***p<0.001.

Gene expression data were used to estimate constituent cell populations within PBMC samples using in silica deconvolution tool CIBERSORT (Newman et al., 2015; Newman et al., 2015) (**Figure 5A**). Among PBMC from acute HIV cases, CD4+ T cells contributed median 24.3% of cells, CD8+ T cells contributed 22.2%, and B cells contributed 4.5%. These were significantly different from HIV negative controls, at 37.8% (p<0.001), 5.0% (p<0.001), and 6.5% (p=0.028) respectively. Other cell populations were not significantly different between groups. These estimated data were validated by correlating with CD4+ and CD8+ counts *via* flow cytometry, with moderate Spearman correlations of 0.645 and 0.705 respectively (both p<0.001) (**Figures 5B, C**).

### Differential expression after adjustment for differences in cell populations

Given the significant differences in cell populations between acute cases and HIV-negative controls, we investigated whether cell counts were a confounding variable adversely impacting differential gene expression interpretation. CD4+ T cell count (as a proportion of lymphocytes) was shown to be inversely correlated with CD8+ T cell count (rho = -0.815, p = <0.001) for all study participants. Furthermore, both CD4+ and CD8+ T cell counts were associated with viral load (rho = -0.518 and 0.480, and p = 0.005 and 0.010, respectively) among HIV cases. CD4+ T cell count was therefore considered a suitable proxy with which to examine the impact of shifts in cell populations on gene expression.

When CD4+ T cell count (%) was adjusted for, 2530 fewer genes were recognized as differentially expressed, and 274 additional genes were recognized as differentially expressed. The impact on the makeup of the list of most differentially expressed genes was low, with only minor rearrangements to the top 50 DEG (**Supplementary Table 3**). Adjustment of gene expression for CD4+ T cell count reduced the range of fold changes of DEG, from between log_2_FC 4.84 and -1.89, to between 3.1 and -1.64.

GSEA was used to examine the biological relevance of changes that may be attributable to PBMC cell population differences between cases and controls. The 2530 genes ‘lost’ as a result of adjustment contributed to 671 upregulated gene sets and 301 downregulated gene sets. **Supplementary Figure 2** demonstrates the top 20 positively and negatively enriched processes, which are important pathways in the immune response, with many directly relevant to viral pathogenesis.

The 274 additional DEG resulting from adjustment for CD4+ T cell population contributed to 96 differentially enriched gene sets. The implication is that by not adjusting for CD4+ T cell count, these potentially important biological processes may have been missed. However, GSEA analysis of these genes indicated that all 23 upregulated gene sets, and all but 11 of the 73 downregulated gene sets, were captured by analyses without CD4+ adjustment. The 11 gene sets potentially missed pertain to downregulation of cellular organization processes (macromolecule localization, and protein complex subunit organization), intracellular signal transduction, and nucleobase compound metabolic processes.

In summary, cell population frequencies were associated with both independent variables (viral load) and outcome measures (gene expression) in this analysis, making them an effect modifier rather than a confounder. Given that the primary objective of this analysis was to provide a global overview of host PBMC gene expression and pathogenic pathways during acute infection, inclusive of changes reflective of diversity in viral load and cell populations, all subsequent analyses of biological implication were not further adjusted for cell populations. This approach is in keeping with the previous mixed cell gene expression studies during early infection that have employed a case-control study design (Li et al., 2009; Zhang et al., 2018). We continued to adjust analyses for essential confounders (age, sex, and RNA extraction personnel). We acknowledge that studies with repeat measures, or comparing different timepoints along the course of HIV infection, would necessitate cell population adjustment.

### Biological interactions and upstream regulators

**Figure 6:**
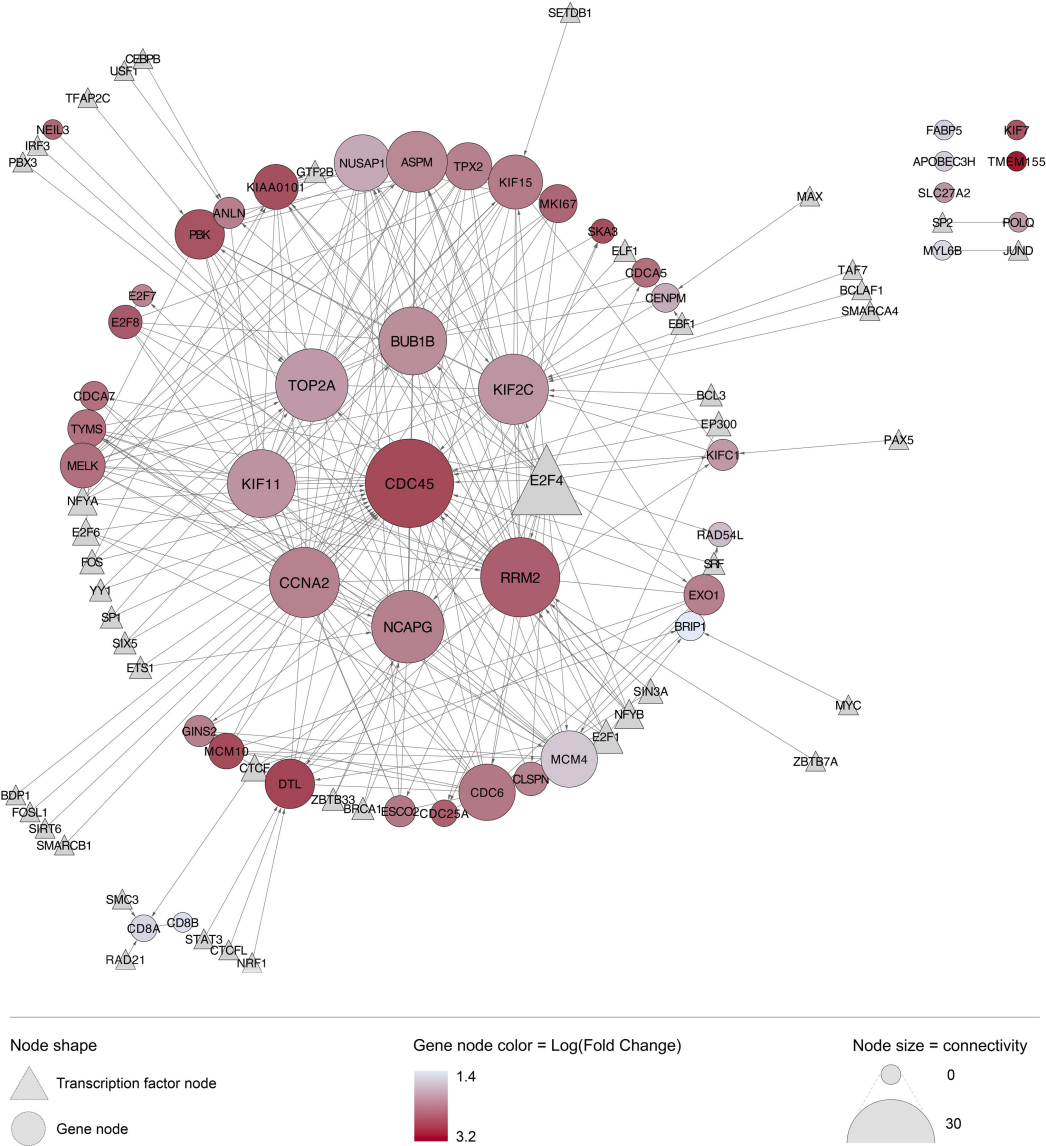
Interaction network of top 50 genes differentially expressed (by adjusted P value) during acute HIV infection. Many of the top 50 most upregulated genes have known biological interactions and upstream regulators. Generated using the STRING database in Cytoscape, with all known upstream regulators linked *via* CyTargetFinder and the ENCODE transcription factor database; circular nodes represent differentially expressed genes; triangular nodes represent upstream transcription factors; edges, shown as grey lines, represent known biological interactions with the arrow indicating direction of interaction (source to target); node color for genes reflects log_2_ fold change value (more red = more significantly differentially expressed, all upregulated in this case); node size represents degree of connectivity to other nodes in the network.

The STRING database identified 35,759 established biological interactions between the 6,131 DEG. A closer look at the top 50 DEG revealed high connectivity and co-upregulation between genes (**Figure 6**). The most highly connected genes included those encoding cell division-related proteins (CDC45, BUB1B, KIF2C, NCAPG, KIF11), cell cycle regulation-related proteins (CCNA2), and DNA replication-related proteins (RRM2 and TOP2A). Transcription factor E2F4 was identified as an upstream regulator of many genes highly over-expressed in this cohort of individuals with acute HIV infection. E2F4 was not found to be overexpressed during acute HIV in our analysis, though functionally-similar E2F1-3, as well as E2F7-8 were overexpressed. Notably, the most significantly over-expressed gene, TMEM155, had no known interactions with other DEG, and no established upstream regulators.

### Dysregulated gene sets

**Figure 7:**
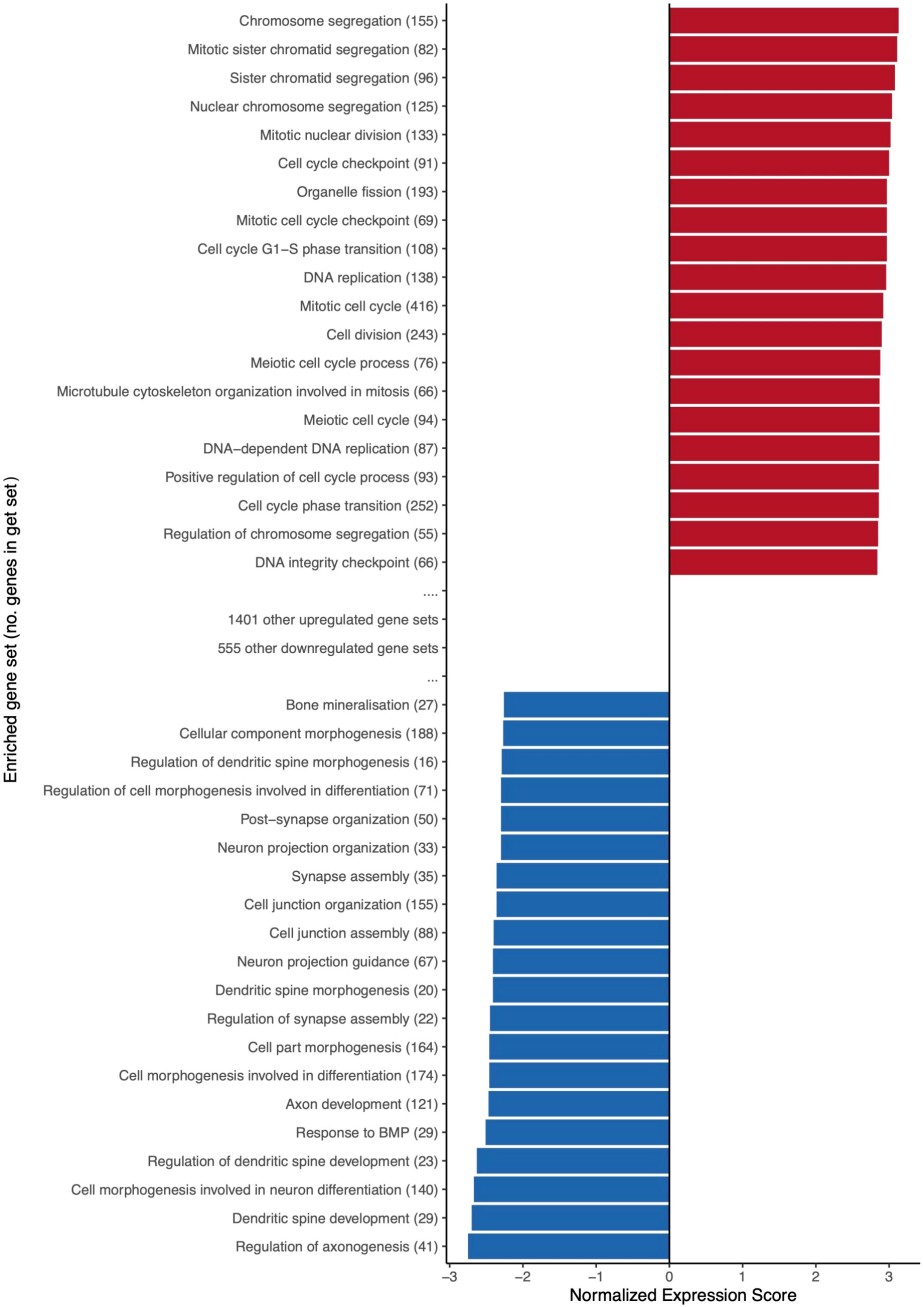
The top 20 most upregulated and downregulated biological process gene sets during acute HIV. Upregulated gene sets overlap to promote functions relating to cell cycle, DNA repair and replication, and immune response. Normalized expression score reflects the degree to which a gene set is overrepresented at the top or bottom of a ranked list of genes, adjusted for differences in gene set size and in correlations between gene sets and the expression dataset. Brackets following gene set names indicate the size of (i.e. number of genes within) an enriched gene set. Only significantly enriched gene sets (p<0.05) are featured.

GSEA was performed to determine over-represented Gene Ontology (GO) biological processes among DEG. 1421 gene sets were upregulated in acute HIV infection (405 reaching p<0.01), compared with 575 biological processes downregulated (150 reaching p<0.01). Normalized enrichment scores of the top 20 most up- and down-regulated gene sets are presented in **Figure 7**, and for all highly significant gene sets in **Supplementary Table 4**. The 20 most upregulated sets all related to cell cycle functions. The top 20 downregulated categories relate to a variety of biological processes, with commonalities around growth and differentiation, and synaptic transmission.

### The ‘interferome’ of acute HIV infection

**Figure 8:**
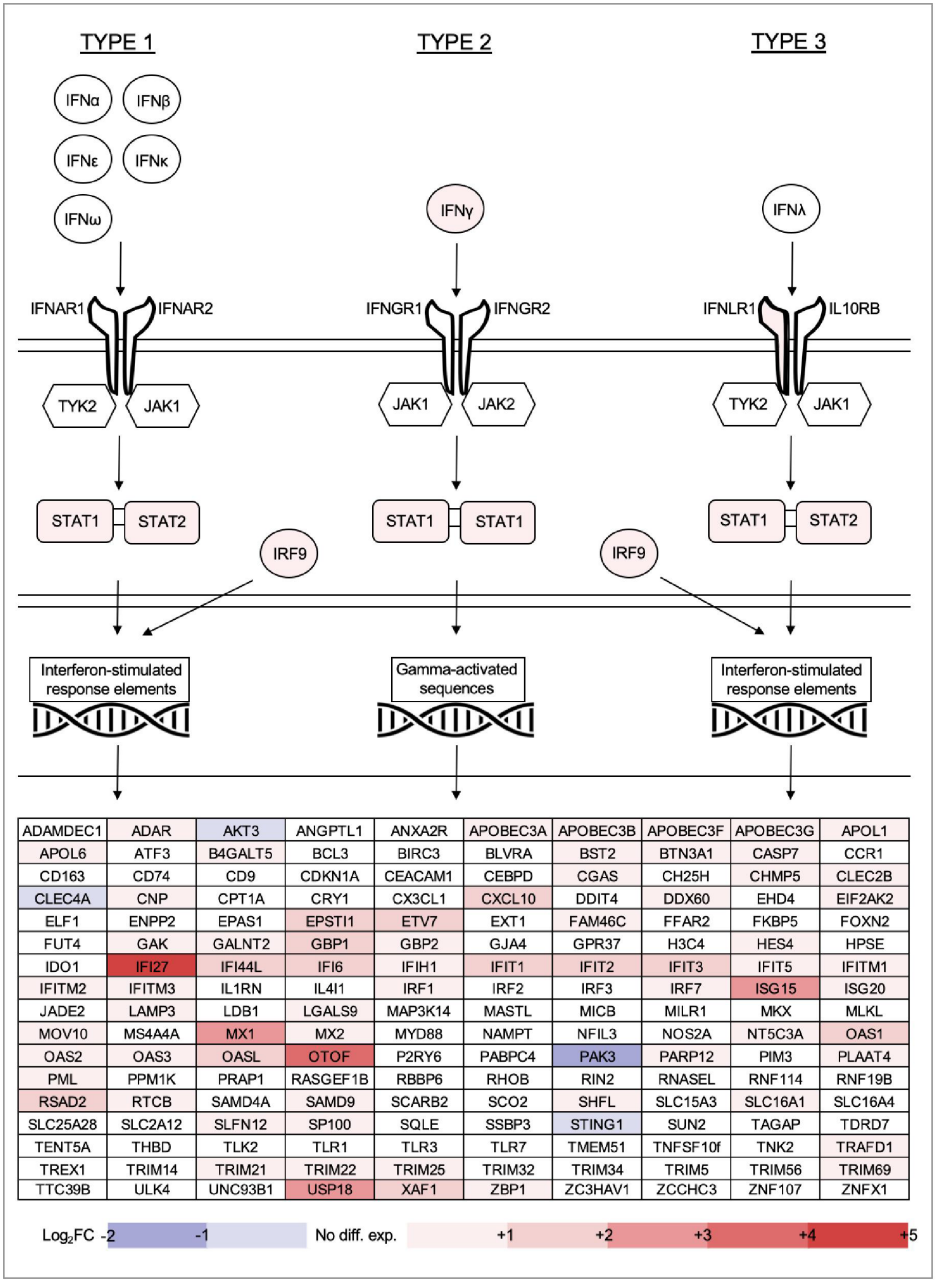
Gene expression changes for all types of interferon, key components of the Type 1, 2 and 3 interferon signalling pathways, as well as 170 other interferon-stimulated genes (ISGs) with established antiviral functions. ISGs listed in alphabetical order.

Within the GSEA results, ten pathways relating to interferon stimulation were prominent (beginning at position 187 by FDR-adjusted q value, normalized enrichment score 2.04). To explore this further, we collated from existing literature a list of interferon pathway genes, as well as interferon-stimulated genes (ISGs) with known antiviral activity (Liu et al., 2011; Schoggins and Rice, 2011; Schoggins et al., 2014; Kane et al., 2016; Yang and Li, 2020; Ullah et al., 2021; Ding et al., 2022). We mapped our differential gene expression results over this list to visualize the interferome pattern of acute HIV infection (**Figure 8**).

Of all interferon types, only IFNγ was overexpressed within PBMC of individuals with acute HIV (noting that extracellular distribution is not captured in this type of study). Of the interferon receptor heterodimers, only IFNLR1 (part of the IFNλ receptor) was upregulated. Kinases TYK2, JAK1 and JAK2 were not differentially expressed, however signal transduction molecules STAT1, STAT2 and complex subunit IRF9 were all overexpressed compared with healthy controls. Many antiviral ISGs were upregulated in early stages of infection, with IFI27, OTOF, ISG15, MX1 and USP18 the most highly overexpressed. IFI27, in particular, was the single most overexpressed gene in individuals with HIV by fold change (+4.8). Only four of the ISGs were downregulated, namely PAK3, AKT3, CLEC4A and STING1. Most ISGs can be induced by multiple types of interferon, and the antiviral ISG analysis did not reveal patterns of upregulation specific to any type of interferon response. However, GSEA results indicated that Type 1 and Type 2 pathways were most recognisable among upregulated DEG overall.

### Enrichment mapping

**Figure 9:**
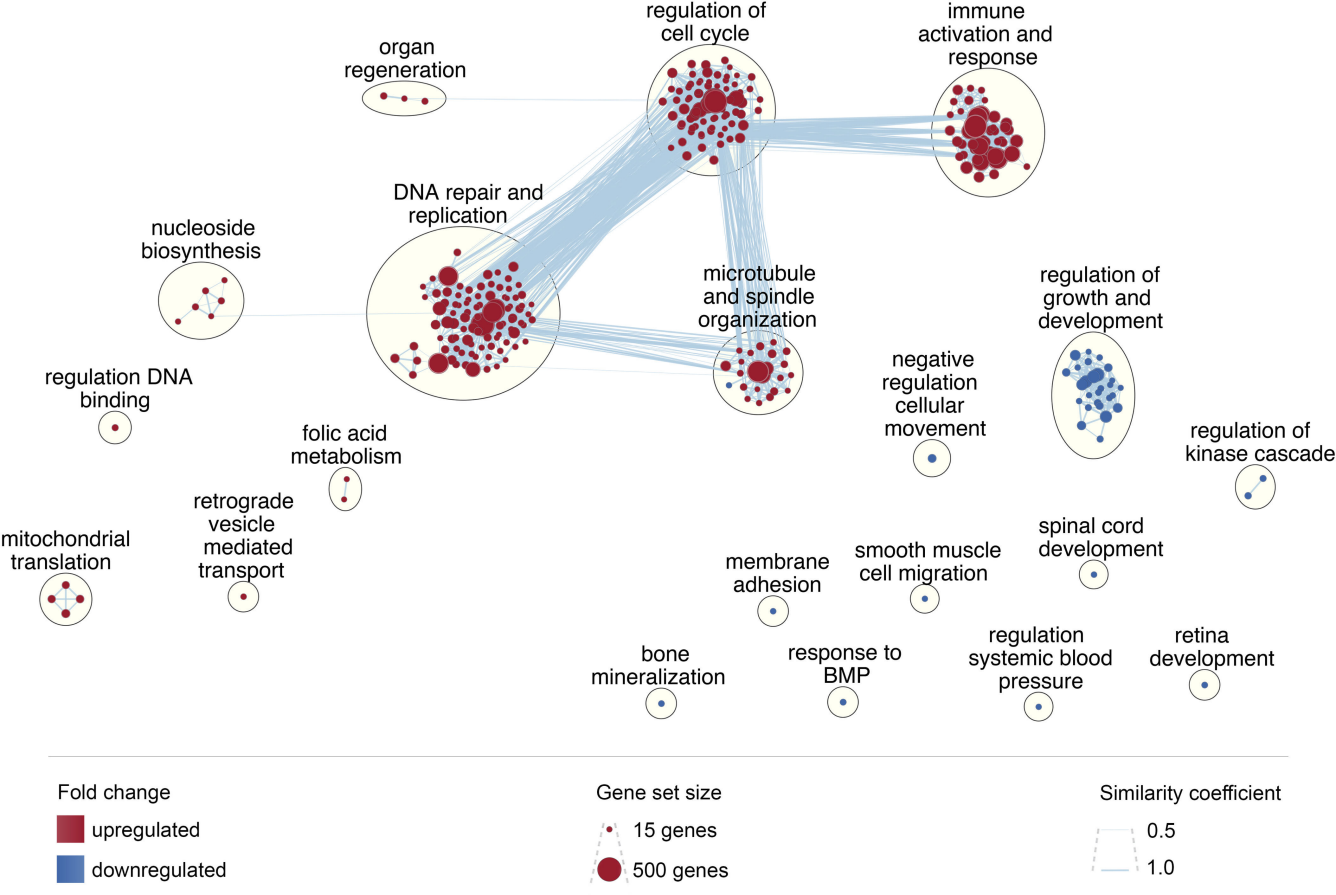
Enrichment map of enriched biological processes. Enrichment map was created in Cytoscape using GSEA results; each node represents a gene set; node size reflects gene set size; node color reflects upregulation (red) or downregulation (blue); clusters were generated using Community Cluster (GLay) Annotation Set in the AutoAnnotate plugin, with minor adjustments to improve language; edges, shown as blue lines, reflect gene set overlap between nodes; edge width reflects the similarity coefficient.

GSEA results were used to inform an unbiased enrichment map of all up- and down-regulated GO biological process gene sets (**Figure 9**). The largest gene set clusters related to DNA replication and repair, regulation of cell cycle, microtubule and spindle organization, and immune activation and response. These functional clusters also have a high degree of gene overlap and similarity. Conversely, acute HIV downregulated functions related to regulation of growth and development, as well as numerous other functions in comparatively discrete pathways. These include bone mineralization, spinal cord and retina development, and regulation of systemic blood pressure, among others.

### Plasma protein validation

The relationship between gene expression and protein expression is complex, however any observed correlation validates the reliability of sequencing data, as gene expression changes are observed to be associated with functional protein changes. Of the 29 acute HIV cases and 46 HIV negative controls in this analysis, n=15 and n=34 respectively had corresponding protein data from the same blood draw. Quantification of n=49 protein-based biomarkers in plasma was completed for this cohort as previously reported (Pastor et al., 2017a). Correlations between proteins in peripheral blood and their respective genes in circulating PBMC were significant in 20.4% of all relationships tested (**Supplementary Table 5**).

## Discussion

This study describes the global PBMC gene expression profile of humans acutely infected with HIV. By using the largest number of subjects for an early HIV transcriptomic study to date, we have been able to describe the massive gene expression dysregulation taking place in circulating PBMC, detailing the top DEG, frequency of correlation between DEG and viral load, up- and down-regulated gene sets, and enriched biological processes.

Previous studies of gene expression during early HIV expression have had six notable limitations. First, the non-specific symptoms of acute HIV make prompt diagnosis difficult and this is reflected in the time of sample collection, ranging from 1-5 months (Hyrcza et al., 2007) or within 6 months (Zhang et al., 2018) post-infection, or within 4 months post-documented seroconversion (Li et al., 2009). Kazer et al. were able to collect earlier samples but this required the resource intensive approach of monitoring a prospective cohort of at-risk women (Kazer et al., 2020). Second, sample sizes were small (between 3 and 9 individuals) which hinders interpretation and reliability. Third, almost all subjects with HIV were male, although almost half of new infections worldwide occur in women (The Joint United Nations Programme on HIV/AIDS (UNAIDS), 2020). Fourth, the study sites of North America (Hyrcza et al., 2007; Li et al., 2009) and China (Zhang et al., 2018) are regions that represent minority HIV subtypes (Hemelaar et al., 2019; Gartner et al., 2020), with low HIV burden and mainly a concentrated HIV epidemic (in comparison with the generalized epidemic present across countries in ESA which bear two-thirds of the worldwide burden of HIV [The Joint United Nations Programme on HIV/AIDS (UNAIDS), 2020)]. Fifth, each study used different sample types (T cells, lymph node tissue and PBMC) limiting comparability and meaningful conclusions. Finally, Li and Hyrcza utilized microarray technology, now largely superseded by RNA-seq (Zhao et al., 2014; Judge et al., 2020).

To address these methodological and external validity issues, this study used RNA-seq profiling to describe gene expression changes in a large cohort of recently infected individuals, compared with contemporaneously recruited, HIV-negative controls who had presented with similar symptoms and were demographically indistinguishable. The study recruited men and women from ESA where HIV-1 subtype C is predominant. PBMC were identified as an ideal tissue for analysis, being readily collectable in resource-limited settings, and able to provide a comprehensive view of the host response, encompassing both target cells of HIV, and other cells directly and indirectly involved in the immune and inflammatory response to HIV. Although RNA-seq has the capacity to generate read lengths and depth of coverage greater than those applied in our experiment, sequencing using single-end 50 base pairs reads has been shown to produce high quality differential gene expression results, with negligible difference compared to up to 100 base paired reads (Chhangawala et al., 2015) and therefore was used for this study to maintain both quality and economy. Studies looking in detail at structural rearrangements to the genome, alternate splicing, or allele-specific expression may necessitate a greater depth of sequencing.

Changes in gene expression during acute HIV infection drive early immune system dysregulation, setting the stage for lifelong infection and progressive immunological decline. Our study identified that processes relating to cell cycle and cell division are the most profoundly upregulated functions during acute infection (**Figure 9**). By design, our study is likely to have captured urgent attempts at cell regeneration triggered by CD4+ T cell loss (Mehandru et al., 2007), and rapid expansion of CD8+ T cells and immature transitional B cells, required for cytotoxic and early cellular immune responses. The quadruplicate evidence of E2F-sensitive CDCA7 ranking as the second most upregulated gene, the strong correlation of CDCA7 with viral load, the identification of an E2F-family protein as a common upstream regulator of many other overexpressed genes during acute infection, and the over-expression of E2F1-3 among early HIV cases, points toward a key role of E2F proteins in promoting CDCA7-led cell division during early HIV.

Lymphocyte survival during acute HIV infection is a very delicate balance of pro-apoptotic and pro-survival signals. Pathways related to cell cycle entry are significantly upregulated in PBMC, perhaps reflecting premature halting of cell cycle, or the posited ‘tap and drain’ hypothesis (Ho et al., 1995; Vidya Vijayan et al., 2017), as a mechanism for widespread cell death. The BCL2 family of proteins is known to protect cells from apoptosis (Schinzel et al., 2004; Kale et al., 2018). In this study, BCL2 and BCL2L2 genes were both downregulated among HIV cases, whereas pro-apoptotic genes BIM, BMAIP1 and BIK (involved in triggering the apoptosis cascade), as well as BAX and BAK (downstream apoptosis effector molecules) were all upregulated. In short, two of the key genes that protect lymphocytes from apoptosis are downregulated and five of those that promote apoptosis are upregulated. Gene set enrichment analysis systematically corroborated upregulation of pathways related to cellular stress responses, suggesting that cell death in these early stages is predominantly pathological rather than physiological.

At this stage in the course of infection, the immune response is characterized by upregulated interferon-driven inflammatory and antiviral pathways, complement activation, humoral immunity (particularly upregulated antigen processing and presentation), and increased phagocytic activity (**Supplementary Table 4**). We described the interferome of acute HIV infection, which was characterized by a broad upregulation of antiviral ISGs, with IFI27, OTOF, ISG15, MX1 and USP18 the most overexpressed. Of all DEG, the most upregulated gene by fold change was interferon, alpha-inducible protein 27 (IFI27). High levels of expression of IFI27 have been reported previously, across different stages of infection (Liu et al., 2022; Palm et al., 2022), and the gene was even put forth as a ‘potential therapeutic target for HIV infection’ based on its downregulation after ART commencement (Huang et al., 2022). Among HIV cases in our analysis, IFI27 was not correlated with viral load. The interferome is of dual interest, as a potential cause of aberrant inflammation, and also in understanding the mechanisms by which HIV evades these interferon effectors and establishes long-term infection.

We did not expect to find significant differential gene expression relating to establishment of latent reservoirs. Although the reservoir is thought to exist from the earliest stages of infection, only small numbers (one per million) of memory and functional CD4+ T cells go on to be latently infected (Kuo and Lichterfeld, 2018), and at this stage of infection there is already a diminishing population of CD4+ T cells relative to total lymphocytes. Other cell types contributing to the reservoir would comprise an even smaller proportion within our samples (Kuo and Lichterfeld, 2018). Of four markers thought to be overexpressed in infected, transcriptionally-active T cells (Descours et al., 2017; Pardons et al., 2019), only MKI67 was upregulated; FCGR2A and TNFRSF9 were not differentially expressed and IL2RA was downregulated. However, LAG3, TIGIT and HAVCR2 [three of four immune checkpoint markers found to be enriched in cells with chromosomally-integrated HIV-1 proviruses (Chew et al., 2016; Fromentin et al., 2016; Pardons et al., 2019)] were over-expressed among HIV cases in this analysis. All three genes have a broad array of roles in immune responses, so their presence likely has alternate explanations. Gene expression analyses would be of benefit to the ongoing investigation of reservoirs, though single-cell approaches would be better equipped for this task than our global PMBC study.

Acute HIV infection, particularly subtype C, is associated with tremendously high viral load. Among all DEG, 0.8% positively correlated with viral load, including 8 of the top 50 most profoundly upregulated genes (16%) which have core cell cycle functions. This suggests that high viremia may drive key pathological processes seen during acute infection, particularly cell division disruption. Gene expression correlating with viral load has been previously reported, with strong associations between expression of interferon-stimulated genes and antiretroviral defense genes with viremia (Rotger et al., 2010). An almost equal amount (0.8%) of genes differentially expressed in PBMC during acute HIV infection were negatively correlated with viral load. The relatively balanced pattern of positive and negative correlation of viral load with gene expression in PBMC contrasts previous work by Smith et al. (2009), whereby gene expression in mixed cell lymphatic tissue showed an overwhelmingly negative correlation with viral load (95% of associations) (Smith et al., 2010).

The genes consistently differentially expressed during acute HIV, but which are not correlated with viral load (even when conservative pre-FDR adjusted p value thresholds are used), are particularly interesting for exploration of biomarkers of recent infection. These candidates have the potential to remain stable during acute infection even as viral load fluctuates. Genes fitting this profile, warranting further investigation, include cell cycle transcription factor genes E2F7 and E2F8, cell cycle regulation gene DTL, DNA repair gene BRIP1, and antiretroviral gene APOBEC3H.

TMEM155 (also known as SMIM43) is a notable addition to this category. Uncorrelated with viral load, and not known to interact with any other over-expressed genes, TMEM155 was unexpectedly found atop the list of most highly over-expressed genes (in terms of significance, but also ranked 3rd in terms of fold change of overexpression). **Figure 2** highlights that upregulation of this gene was consistent among all individuals with acute HIV. TMEM155 was identified as significantly differentially expressed (a 1.7-fold increase) in Li et al., 2009, but was not highlighted further as an important gene. The chromosomal region encoding TMEM155 was found to be one of many differentially methylated during HIV infection (Shiau, 2017). Outside of HIV, TMEM155 has been associated with hepatitis C liver cirrhosis (Ijaz et al., 2019) and brain tumours (Wang et al., 2010; Grupenmacher et al., 2013), and is possibly associated with essential tremor (Odgerel et al., 2019), basal cell nevus syndrome fibroblasts (Phatak et al., 2019), nystagmus (Toyono et al., 2015), cerebellar atrophy with motor neuronopathies (Burns et al., 2018), and regulation of female fertility (Barragán et al., 2017).

The biological functions downregulated during acute HIV infection are remarkable for their resemblance to the longer-term consequences of HIV infection. Hypertension, retinopathy, osteopenia and muscular atrophy are well characterized sequelae of HIV, and this study demonstrates that the gene sets potentially predictive of these changes may well be disturbed within the first few weeks of infection (**Figure 5B**).

Our study has a number of inherent limitations. Completing analyses on mixed cell samples (as opposed to single cell) does introduce a level of effect modification which we have attempted to describe using cell population deconvolution analysis to estimate population frequencies, and describing the effects of CD4+ T cell population adjustment. As with all genome-wide association studies, we cannot rule out an element of reverse causation; for example, there is a possibility that some genes were over-expressed as a by-product of heightened cell turnover, rather than because they contribute mechanistically to the turnover. Almost certainly our DEG results reflect a combination of both. Others have attempted to untangle this issue *in vitro* for specific genes, arguing that individual genes are essential mediators of cell proliferative signalling, rather than consequences of said proliferation (Jimenez et al., 2018; Zhang et al., 2021).

Compared to previous studies, this study offers a large sample size of PMBC collected *ex vivo* from patients in the earliest stages of HIV infection, prior to treatment. Although sequencing of samples from the recruitment visit would have been ideal, it was unfeasible for our rural laboratory to process more than 3000 blood samples collected at this time point, when only 2.7% of participants would be diagnosed with acute HIV infection (Pastor et al., 2017a). Additionally, most study participants presented with a non-specific febrile illness, and recall at four weeks post-recruitment allowed time for resolution of other illnesses, thereby enabling comparisons with relatively healthy, time-matched controls. The burden of acute infections is high in ESA; however, **Table 1** clearly shows that there were no significant differences in infections other than HIV between the groups.

In this cohort, gene expression was measured intracellularly in PBMC, whereas protein biomarkers were measured in plasma (Pastor et al., 2017a). Despite the limitations of correlating intracellular gene expression with peripherally circulating proteins, 20% of measured proteins correlated with gene expression, approximating previous reports (Chen et al., 2002; Du et al., 2014). Although RNA-seq does not require functional validation in the classical sense (Coenye, 2021), these findings support the biological plausibility of our results.

## Conclusions

This work demonstrates that acute HIV severely disrupts circulating immune cells, profoundly upregulating many genes, while downregulating others. The degree of dysregulation was frequently, but not universally, correlated with viral load. Analysis of the impacted genes, gene sets and pathways reveals in detail the mechanisms behind the shift toward a system of high cell turnover, cell death and cell expansion, and unwitting viral propagation. This work highlights a need for further investigation of the role of E2F transcription factors in driving exhaustive cell division during acute infection; the role of BCL2 family downregulation in permitting unchecked lymphocyte death, and the unknown role of highly over-expressed TMEM155 during acute infection. CCNA2, CDCA7, and IFI27 are being explored as therapeutic targets within oncology spheres, and heightened expression of these transcripts in acute infection suggest that learnings may be relevant for application in HIV treatments. Additionally, the high degree of consistency in gene expression among HIV cases hints at the possibility that transcriptomic biomarkers might be useful as indicators of recent incident HIV infection. These findings illuminate the molecular mechanisms underlying the pathogenesis of acute HIV infection *in vivo*, and have implications for designing therapeutic interventions aiming to prevent or halt this early immune damage.

## Data availability statement

The transcriptomic datasets presented in this study can be found in online repositories. The names of the repository/repositories and accession number(s) can be found below: https://www.ncbi.nlm.nih.gov/geo/, GSE199911.

## Ethics statement

The studies involving human participants were reviewed and approved by ethical review boards at Barcelona Clinic Hospital (2011/6264), the Ministry of Health of Mozambique (461/CNBS/12) and the University of Western Australia (2019/RA/4/1/6296). The patients/participants provided their written informed consent to participate in this study.

## Author contributions

Conceptualization, EP, MJ, and PS; Methodology, EP and MJ; Software, EP, MJ, KC, and DA; Formal Analysis, EP and MJ; Investigation, all authors; Resources, EP, MJ, LP, LF-S, and CJ; Data Curation, EP and MJ; Writing – Original Draft, EP and MJ; Writing – Review & Editing, all authors; Visualization, EP and MJ; Supervision, DN and PS; Project Administration, EP and MJ; Funding Acquisition, HC, DN and PS. All authors contributed to the article and approved the submitted version. 

## Supplementary material

Supplementary Table 1 Full list of differentially expressed genes – acute HIV cases versus HIV negative controls. # = Rank by adj.P.Val; EnsemblID = Ensembl Stable ID name of the transcript; HGNC = HUGO Gene Nomenclature Committee name of the gene; logFC = Log_2_ fold change; AveExpr = average expression across all samples, in log2 counts per million reads; adj.P.Val = Benjamini-Hochberg false discovery rate adjusted p-value.

Supplementary Table 2 Full list of differentially expressed genes that correlate with viral load in acute HIV cases. # = Rank by rho; EnsemblID = Ensembl Stable ID name of the transcript; HGNC = HUGO Gene Nomenclature Committee name of the gene; rho = Spearman correlation coefficient; adj.P.Val = false discovery rate adjusted p-value.

Supplementary Table 3 Effect of adjustment for CD4+ T cell count (%) on Top 50 DEG. Pos. = Position ranked by adj.P.Val; HGNC = HUGO Gene Nomenclature Committee name of the gene; logFC = Log2 fold change; AveExpr = average expression across all samples, in log2 counts per million reads; adj.P.Val = Benjamini- Hochberg false discovery rate adjusted p-value; no-adj pos. = comparative position in the previous table, without adjustment for CD4+ T cell count (%). Yellow = top 50 DEG concordant in both analyses; orange = top 50 DEG discordant between analyses (though all within top 100).

Supplementary Table 4 Full list of differentially expressed gene sets – acute HIV cases versus HIV negative controls as determined by gene set enrichment analysis (GSEA). **(A)** Overexpressed gene sets. **(B)** Underexpressed gene sets. # = Rank by NES; Name = Gene Ontology name of the geneset; Size = the number of genes in the geneset; NES = Normalized Enrichment Score; NOM p-val = nominal P value (statistical significance of the observed ES relative to null distribution); FDR q-val = False Discovery Rate Q value (the P value has been adjusted for FDR).

Supplementary Table 5 Intracellular gene expression changes correlated moderately with measured plasma proteins (significant results only, of forty-nine quantifiable soluble biomarkers).

Supplementary Figure 1 Mean mapped reads of three case samples as sequenced on two different RNAseq batches.

Supplementary Figure 2 Top 20 most up and down-regulated gene sets, associated with adjustment for cell populations.
