# Supplementary material for: Gene dysregulation in acute HIV-1 infection – early transcriptomic analysis reveals the crucial biological functions affected
Source: Front Cell Infect Microbiol. 2023 Apr 3;13:1074847. doi: 10.3389/fcimb.2023.1074847 (PMC10106835; doi:10.3389/fcimb.2023.1074847)
Supplement: Supplementary Table 1 — Full list of differentially expressed genes – acute HIV cases versus HIV negative controls. # = Rank by adj.P.Val; EnsemblID = Ensembl Stable ID name of the transcript; HGNC = HUGO Gene Nomenclature Committee name of the gene; logFC = Log2 fold change; AveExpr = average expression across all samples, in log2 counts per million reads; adj.P.Val = Benjamini-Hochberg false discovery rate adjusted p-value. [file DataSheet_1.pdf]

Supplementary Table 1: Full list of differentially expressed genes – acute HIV cases versus HIV negative controls.

#	EnsemblID	HGNC	logFC	AveExpr	adj.P.Val
1	ENSG00000164112	TMEM155	3.267514	1.456046	4.46E-35
2	ENSG00000144354	CDCA7	2.47209	3.923762	9.42E-34
3	ENSG00000137804	NUSAP1	1.979959	4.082081	1.08E-26
4	ENSG00000166803	KIAA0101	2.784338	2.649991	3.2E-26
5	ENSG00000176890	TYMS	2.473498	2.944304	3.2E-26
6	ENSG00000109805	NCAPG	2.334234	2.421143	3.2E-26
7	ENSG00000093009	CDC45	2.836749	1.211185	1.12E-25
8	ENSG00000254126	CD8BP	1.570053	4.146289	1.12E-25
9	ENSG00000145386	CCNA2	2.321098	3.171912	3.88E-25
10	ENSG00000143476	DTL	2.886901	2.316516	6.18E-25
11	ENSG00000165304	MELK	2.489211	1.517418	6.18E-25
12	ENSG00000138160	KIF11	2.168095	3.633372	6.18E-25
13	ENSG00000172116	CD8B	1.50107	6.851844	3.77E-24
14	ENSG00000156970	BUB1B	2.214816	1.903625	6.3E-24
15	ENSG00000196465	MYL6B	1.562934	2.430646	6.3E-24
16	ENSG00000148773	MKI67	2.577557	4.837443	6.65E-24
17	ENSG00000011426	ANLN	2.351843	1.528399	1.37E-23
18	ENSG00000161570	CCL5	1.389217	9.832015	1.54E-23
19	ENSG00000171848	RRM2	2.648566	4.21435	1.78E-23
20	ENSG00000168078	PBK	2.758444	0.548038	1.99E-23
21	ENSG00000034063	UHRF1	1.923329	2.584488	2.07E-23
22	ENSG00000092853	CLSPN	2.304573	2.043356	2.77E-23
23	ENSG00000094804	CDC6	2.411704	2.13909	4.19E-23
24	ENSG00000065328	MCM10	2.817904	0.883438	5.57E-23
25	ENSG00000146670	CDCA5	2.485974	2.105461	1E-22
26	ENSG00000131747	TOP2A	2.114054	4.696429	1E-22
27	ENSG00000100298	APOBEC3H	1.665933	2.120077	1E-22
28	ENSG00000164687	FABP5	1.642369	3.838538	1E-22
29	ENSG00000163808	KIF15	2.38185	1.760965	1.25E-22
30	ENSG00000066279	ASPM	2.263024	2.797666	1.57E-22
31	ENSG00000136492	BRIP1	1.402205	2.482367	1.97E-22
32	ENSG00000165480	SKA3	2.784406	0.303896	2.39E-22
33	ENSG00000131153	GINS2	2.33612	1.673982	4.25E-22
34	ENSG00000166813	KIF7	2.730583	-0.83294	4.96E-22
35	ENSG00000129173	E2F8	2.679807	0.486855	4.96E-22
36	ENSG00000174371	EXO1	2.342272	1.191835	4.96E-22
37	ENSG00000142945	KIF2C	2.156903	1.835663	4.96E-22
38	ENSG00000051341	POLQ	2.096836	1.558815	4.96E-22
39	ENSG00000104738	MCM4	1.709862	4.8154	4.96E-22
40	ENSG00000088325	TPX2	2.327686	2.669634	6.45E-22
41	ENSG00000140284	SLC27A2	2.142166	1.831892	8.65E-22

42	ENSG00000164045	CDC25A	2.626844	0.725675	9.19E-22
43	ENSG00000237649	KIFC1	2.118952	2.135628	1.23E-21
44	ENSG00000171320	ESCO2	2.401353	0.820294	1.63E-21
45	ENSG00000165891	E2F7	2.296616	1.314674	1.73E-21
46	ENSG00000100162	CENPM	1.953828	1.515999	3.66E-21
47	ENSG00000109674	NEIL3	2.569431	-0.33828	4.14E-21
48	ENSG00000153563	CD8A	1.587898	8.661943	4.22E-21
49	ENSG00000172965	MIR4435-1HG	1.136808	4.940479	4.22E-21
50	ENSG00000085999	RAD54L	1.814108	0.59971	5.04E-21
51	ENSG00000152253	SPC25	2.706831	-0.16786	5.25E-21
52	ENSG00000100526	CDKN3	1.975177	1.457688	6.34E-21
53	ENSG00000004468	CD38	1.761085	6.373514	9.16E-21
54	ENSG00000161888	SPC24	2.279101	0.311504	1.02E-20
55	ENSG00000186185	KIF18B	2.803076	-0.01898	1.14E-20
56	ENSG00000154839	SKA1	2.291266	1.159663	1.14E-20
57	ENSG00000123485	HJURP	2.28303	1.577897	1.31E-20
58	ENSG00000140525	FANCI	1.173914	4.631696	1.65E-20
59	ENSG00000117632	STMN1	1.514723	5.910346	1.9E-20
60	ENSG00000075218	GTSE1	2.16065	0.801961	1.95E-20
61	ENSG00000164104	HMGB2	1.174833	7.606025	2.17E-20
62	ENSG00000222041	LINC00152	1.159367	4.543222	3.43E-20
63	ENSG00000112742	TTK	2.062414	1.778929	3.72E-20
64	ENSG00000064886	CHI3L2	2.090383	4.988008	4.43E-20
65	ENSG00000236044	FABP5P2	1.580411	1.810599	4.75E-20
66	ENSG00000140534	TICRR	2.380427	-0.34512	5.34E-20
67	ENSG00000213201	FABP5P10	1.764527	0.193176	5.34E-20
68	ENSG00000123473	STIL	1.561518	2.460079	5.34E-20
69	ENSG00000147889	CDKN2A	1.499349	1.238975	5.37E-20
70	ENSG00000178999	AURKB	2.069394	1.147768	6.21E-20
71	ENSG00000139734	DIAPH3	2.253998	-0.41911	7.05E-20
72	ENSG00000111206	FOXM1	1.942938	1.925084	8.41E-20
73	ENSG00000119969	HELLS	1.363783	3.510083	1.07E-19
74	ENSG00000184661	CDCA2	2.362916	0.935526	1.48E-19
75	ENSG00000143228	NUF2	1.547539	2.036793	2.12E-19
76	ENSG00000138180	CEP55	2.461691	1.801148	2.42E-19
77	ENSG00000142731	PLK4	1.392426	3.14894	2.56E-19
78	ENSG00000167513	CDT1	2.357518	1.283315	2.6E-19
79	ENSG00000151725	CENPU	1.58602	3.223663	3.38E-19
80	ENSG00000035499	DEPDC1B	1.838608	1.868048	3.94E-19
81	ENSG00000080986	NDC80	1.325479	3.384164	4.2E-19
82	ENSG00000169607	CKAP2L	1.950626	1.527843	4.75E-19
83	ENSG00000149554	CHEK1	1.452785	3.335998	7.4E-19
84	ENSG00000106462	EZH2	1.234252	5.13744	8.62E-19
85	ENSG00000118193	KIF14	2.078205	1.127328	9.1E-19

86	ENSG00000090889	KIF4A	2.433674	0.744707	9.11E-19
87	ENSG00000171241	SHCBP1	1.70445	2.674962	9.15E-19
88	ENSG00000134901	KDELC1	1.860777	-0.11023	1.08E-18
89	ENSG00000122952	ZWINT	1.84136	3.056527	1.3E-18
90	ENSG00000132646	PCNA	1.372976	5.955444	1.3E-18
91	ENSG00000198826	ARHGAP11A	1.05236	3.809685	3.13E-18
92	ENSG00000134690	CDCA8	1.8512	1.589467	3.13E-18
93	ENSG00000164626	KCNK5	1.744278	1.166009	4.17E-18
94	ENSG00000085840	ORC1	1.73668	2.194547	6.5E-18
95	ENSG00000075702	WDR62	1.199335	1.743337	6.55E-18
96	ENSG00000089685	BIRC5	2.463511	1.775044	8.68E-18
97	ENSG00000100479	POLE2	1.627119	1.146607	9.09E-18
98	ENSG00000175063	UBE2C	2.265234	1.225381	1.03E-17
99	ENSG00000051180	RAD51	1.933007	1.263404	1.16E-17
100	ENSG00000164611	PTTG1	1.206949	3.687117	1.59E-17
101	ENSG00000122966	CIT	1.60374	1.2033	1.99E-17
102	ENSG00000135451	TROAP	2.41916	0.43942	1.99E-17
103	ENSG00000111247	RAD51AP1	1.639933	2.290961	1.99E-17
104	ENSG00000121211	MND1	2.318664	-0.2941	2.25E-17
105	ENSG00000146410	MTFR2	1.411081	1.150525	2.35E-17
106	ENSG00000121152	NCAPH	1.690229	2.600256	2.39E-17
107	ENSG00000165409	TSHR	2.086794	-0.36095	3.09E-17
108	ENSG00000127564	PKMYT1	2.518406	0.155653	3.11E-17
109	ENSG00000092470	WDR76	0.962602	3.453407	5.31E-17
110	ENSG00000024526	DEPDC1	2.350866	0.070326	5.94E-17
111	ENSG00000240350		1.417834	1.036741	7.42E-17
112	ENSG00000185480	PARPBP	1.068406	1.97464	8.75E-17
113	ENSG00000167900	TK1	2.061659	2.579126	8.93E-17
114	ENSG00000111665	CDCA3	1.750177	0.66588	1.01E-16
115	ENSG00000162063	CCNF	1.310596	1.947577	1.08E-16
116	ENSG00000072571	HMMR	2.087549	1.551898	1.16E-16
117	ENSG00000170312	CDK1	1.801306	3.12501	1.19E-16
118	ENSG00000157456	CCNB2	2.154034	1.730001	1.21E-16
119	ENSG00000160791	CCR5	1.92809	5.490944	1.37E-16
120	ENSG00000077152	UBE2T	1.537064	1.692498	1.48E-16
121	ENSG00000112312	GMNN	1.362507	3.427214	2.21E-16
122	ENSG00000143740	SNAP47	0.840453	4.2895	2.66E-16
123	ENSG00000145687	SSBP2	-0.86979	4.450167	3.64E-16
124	ENSG00000164109	MAD2L1	1.055703	3.897652	3.87E-16
125	ENSG00000117724	CENPF	1.754647	4.022238	3.93E-16
126	ENSG00000101003	GINS1	1.875855	0.789763	4.36E-16
127	ENSG00000115163	CENPA	2.08138	-0.18154	4.38E-16
128	ENSG00000137807	KIF23	1.674044	2.021331	4.73E-16
129	ENSG00000105374	NKG7	1.276514	9.446487	5.09E-16

130	ENSG00000136982	DSCC1	1.734532	0.839233	5.33E-16
131	ENSG00000182628	SKA2	0.964036	4.633558	5.99E-16
132	ENSG00000137812	CASC5	1.558259	3.010789	6.73E-16
133	ENSG00000077327	SPAG6	3.105541	-1.28092	9.62E-16
134	ENSG00000173207	CKS1B	0.932186	3.403461	1.04E-15
135	ENSG00000126787	DLGAP5	2.484008	1.708799	1.32E-15
136	ENSG00000183856	IQGAP3	1.91939	-0.94786	1.58E-15
137	ENSG00000109685	WHSC1	0.572295	5.893648	1.87E-15
138	ENSG00000158402	CDC25C	2.309491	-1.1471	2.58E-15
139	ENSG00000030110	BAK1	0.823608	5.841076	3.22E-15
140	ENSG00000160957	RECQL4	1.187103	1.942591	3.84E-15
141	ENSG00000168280	KIF5C	-0.92949	3.336398	3.98E-15
142	ENSG00000128578	STRIP2	1.245079	0.819035	5.8E-15
143	ENSG00000187741	FANCA	0.99403	3.155502	6.28E-15
144	ENSG00000112029	FBXO5	1.011807	3.944714	6.92E-15
145	ENSG00000101447	FAM83D	1.46671	2.451344	7.42E-15
146	ENSG00000188011	CXXC11	1.623019	1.522773	7.53E-15
147	ENSG00000145649	GZMA	1.232436	7.91889	8.12E-15
148	ENSG00000073111	MCM2	1.496087	3.930282	8.77E-15
149	ENSG00000241735	FABP5P3	1.591017	-0.53044	9.31E-15
150	ENSG00000182054	IDH2	1.011264	6.613985	9.53E-15
151	ENSG00000183918	SH2D1A	1.206249	6.809219	9.94E-15
152	ENSG00000163507	KIAA1524	1.005842	3.002044	1.19E-14
153	ENSG00000119333	WDR34	1.255009	2.380306	1.34E-14
154	ENSG00000144395	CCDC150	1.48673	-0.26733	1.6E-14
155	ENSG00000161800	RACGAP1	1.023523	3.839032	1.89E-14
156	ENSG00000196867	ZFP28	-1.06718	1.734427	4.1E-14
157	ENSG00000111445	RFC5	0.618501	3.70515	4.16E-14
158	ENSG00000271288	IGHV1OR15-3	1.735745	0.649412	4.31E-14
159	ENSG00000159259	CHAF1B	1.014834	2.141055	5.25E-14
160	ENSG00000169679	BUB1	1.679609	2.7434	6.93E-14
161	ENSG00000211659	IGLV3-25	2.484001	2.280345	7.39E-14
162	ENSG00000139579	NABP2	0.812625	3.881258	7.39E-14
163	ENSG00000091651	ORC6	1.097555	1.775627	8.75E-14
164	ENSG00000197381	ADARB1	-0.5965	4.920486	9.87E-14
165	ENSG00000120509	PDZD11	0.748167	3.770448	1.25E-13
166	ENSG00000140451	PIF1	1.280256	1.972505	1.32E-13
167	ENSG00000180198	RCC1	0.588785	4.811446	1.32E-13
168	ENSG00000138092	CENPO	0.769263	2.797394	1.54E-13
169	ENSG00000100206	DMC1	2.224426	-1.40114	1.61E-13
170	ENSG00000166508	MCM7	0.66883	6.471827	1.64E-13
171	ENSG00000105011	ASF1B	1.538293	2.271618	1.76E-13
172	ENSG00000203760	CENPW	1.27155	1.833118	1.8E-13
173	ENSG00000187456	RDM1	2.155391	-1.63072	2.05E-13

174	ENSG00000101057	MYBL2	1.663994	3.800009	2.16E-13
175	ENSG00000012048	BRCA1	1.360115	2.803076	2.54E-13
176	ENSG00000124788	ATXN1	0.557416	6.87401	2.54E-13
177	ENSG00000137563	GGH	1.24688	2.315915	3.13E-13
178	ENSG00000165949	IFI27	4.842724	3.378715	3.61E-13
179	ENSG00000097021	ACOT7	1.252569	3.29019	3.76E-13
180	ENSG00000165948	IFI27L1	0.923238	2.120568	4.13E-13
181	ENSG00000089692	LAG3	1.459772	4.515968	4.43E-13
182	ENSG00000145220	LYAR	0.691521	6.085272	5.02E-13
183	ENSG00000211973	IGHV1-69	2.393615	2.418617	5.05E-13
184	ENSG00000136824	SMC2	1.019138	4.594255	5.05E-13
185	ENSG00000092621	PHGDH	1.397088	1.985895	6.13E-13
186	ENSG00000198554	WDHD1	1.020518	2.729624	6.72E-13
187	ENSG00000149636	DSN1	0.586025	3.778595	7.49E-13
188	ENSG00000137642	SORL1	-0.74871	8.769712	7.57E-13
189	ENSG00000183763	TRAIP	0.929401	0.922529	8.47E-13
190	ENSG00000213186	TRIM59	0.662249	4.424461	9.02E-13
191	ENSG00000163918	RFC4	0.785755	3.796406	9.27E-13
192	ENSG00000117399	CDC20	2.240809	0.95937	9.51E-13
193	ENSG00000197238	HIST1H4J	1.260079	0.019088	1.04E-12
194	ENSG00000104219	ZDHHC2	-0.54427	5.975703	1.05E-12
195	ENSG00000159111	MRPL10	0.879733	5.977396	1.11E-12
196	ENSG00000169715	MT1E	2.258139	2.276309	1.17E-12
197	ENSG00000197914	HIST1H4K	1.142583	0.059143	1.2E-12
198	ENSG00000156509	FBXO43	1.679027	-1.46668	1.27E-12
199	ENSG00000172653	C17orf66	1.26273	2.130199	1.29E-12
200	ENSG00000163009	C2orf48	1.996321	-1.32963	1.46E-12
201	ENSG00000160050	CCDC28B	0.904166	2.379609	1.76E-12
202	ENSG00000184979	USP18	2.193237	3.104214	1.85E-12
203	ENSG00000115155	OTOF	3.690265	1.685258	2.14E-12
204	ENSG00000138778	CENPE	1.395629	3.066257	2.14E-12
205	ENSG00000100297	MCM5	0.818103	5.560764	2.23E-12
206	ENSG00000075188	NUP37	0.745285	3.777754	2.36E-12
207	ENSG00000235162	C12orf75	0.813171	6.061397	2.37E-12
208	ENSG00000134897	BIVM	0.769072	3.203142	3.25E-12
209	ENSG00000166851	PLK1	1.767519	1.291986	3.29E-12
210	ENSG00000100450	GZMH	1.102971	8.094068	3.29E-12
211	ENSG00000152582	SPEF2	-1.15376	2.737292	3.29E-12
212	ENSG00000108578	BLMH	0.629289	5.382942	3.43E-12
213	ENSG00000147234	FRMPD3	1.128402	2.921946	3.57E-12
214	ENSG00000129810	SGOL1	1.059841	1.073291	4.04E-12
215	ENSG00000162062	C16orf59	2.066886	-1.39169	4.41E-12
216	ENSG00000145850	TIMD4	1.412444	0.396258	4.55E-12
217	ENSG00000211665	IGLV3-16	2.346968	0.689191	4.69E-12

218	ENSG00000221829	FANCG	0.677158	3.510398	4.69E-12
219	ENSG00000135476	ESPL1	1.638619	1.55546	4.79E-12
220	ENSG00000133321	RARRES3	0.751419	6.735873	4.86E-12
221	ENSG00000022267	FHL1	-1.01798	3.518881	5.13E-12
222	ENSG00000175730	BAK1P1	0.87884	0.959977	5.38E-12
223	ENSG00000070087	PFN2	-1.06998	1.314802	5.38E-12
224	ENSG00000162692	VCAM1	2.777735	2.821813	5.95E-12
225	ENSG00000177602	GSG2	1.670741	-0.08308	6.27E-12
226	ENSG00000113319	RASGRF2	-0.99509	4.33149	6.88E-12
227	ENSG00000165704	HPRT1	0.58038	5.13884	7.28E-12
228	ENSG00000143493	INTS7	0.763871	4.562911	7.84E-12
229	ENSG00000114346	ECT2	1.101184	3.114584	7.96E-12
230	ENSG00000211668	IGLV2-11	1.956889	3.716442	8.23E-12
231	ENSG00000224607	IGKV1D-27	2.18129	0.389157	8.69E-12
232	ENSG00000244575	IGKV1-27	2.20116	1.562427	9.84E-12
233	ENSG00000101412	E2F1	1.113711	2.979525	1.08E-11
234	ENSG00000162676	GFI1	1.008785	4.745597	1.39E-11
235	ENSG00000173218	VANGL1	0.901294	3.404074	1.94E-11
236	ENSG00000134291	TMEM106C	0.635472	4.771917	2.08E-11
237	ENSG00000181938	GINS3	0.980418	1.611919	2.23E-11
238	ENSG00000132386	SERPINF1	-0.84361	3.058726	2.95E-11
239	ENSG00000186638	KIF24	1.329759	-0.27067	3.09E-11
240	ENSG00000114812	VIPR1	-1.09168	3.792759	3.14E-11
241	ENSG00000067141	NEO1	-1.02438	3.427244	3.18E-11
242	ENSG00000198417	MT1F	1.422404	2.202444	3.22E-11
243	ENSG00000108010	GLRX3	0.395002	5.367547	3.34E-11
244	ENSG00000071539	TRIP13	1.493805	0.857851	4.04E-11
245	ENSG00000272373		-0.49299	3.285907	4.18E-11
246	ENSG00000124191	TOX2	1.384321	1.823245	4.25E-11
247	ENSG00000164087	POC1A	1.239135	1.020814	4.51E-11
248	ENSG00000109654	TRIM2	-1.24761	0.808473	4.66E-11
249	ENSG00000131462	TUBG1	0.745222	2.49825	4.94E-11
250	ENSG00000224959		1.078631	-0.08213	5E-11
251	ENSG00000153094	BCL2L11	0.983035	6.633913	5.3E-11
252	ENSG00000134057	CCNB1	1.416169	2.202286	5.4E-11
253	ENSG00000187951	ARHGAP11B	0.747647	3.253861	5.41E-11
254	ENSG00000147536	GINS4	1.017454	1.565578	5.41E-11
255	ENSG00000188610	FAM72B	1.129373	0.979803	5.79E-11
256	ENSG00000166483	WEE1	0.654443	4.363304	5.82E-11
257	ENSG00000184271	POU6F1	-0.912	3.682744	5.97E-11
258	ENSG00000135069	PSAT1	1.253085	2.339158	6.14E-11
259	ENSG00000142920	ADC	-0.98373	1.276792	6.27E-11
260	ENSG00000151623	NR3C2	-1.20021	3.813933	6.33E-11
261	ENSG00000029993	HMGB3	0.933621	2.85641	6.95E-11

262	ENSG00000168496	FEN1	0.90075	4.265893	7.91E-11
263	ENSG00000163564	PYHIN1	0.788767	6.361518	7.91E-11
264	ENSG00000123080	CDKN2C	0.833247	2.872088	8.95E-11
265	ENSG00000099139	PCSK5	-0.83166	3.50644	9.9E-11
266	ENSG00000135299	ANKRD6	-0.72662	1.585183	1.01E-10
267	ENSG00000239672	NME1	0.909255	3.577941	1.01E-10
268	ENSG00000100749	VRK1	0.589452	4.965594	1.05E-10
269	ENSG00000072609	CHFR	0.297137	6.096141	1.08E-10
270	ENSG00000171150	SOCS5	-0.47852	4.575524	1.16E-10
271	ENSG00000082458	DLG3	0.663097	4.528527	1.22E-10
272	ENSG00000131507	NDFIP1	-0.4276	6.987115	1.32E-10
273	ENSG00000011590	ZBTB32	1.55446	2.878224	1.4E-10
274	ENSG00000211673	IGLV3-1	2.067993	3.131265	1.43E-10
275	ENSG00000105974	CAV1	1.523296	0.908038	1.44E-10
276	ENSG00000145391	SETD7	-0.92478	5.126349	1.54E-10
277	ENSG00000153044	CENPH	0.742003	2.572286	1.6E-10
278	ENSG00000166432	ZMAT1	-1.07022	5.391745	1.7E-10
279	ENSG00000138613	APH1B	-0.48549	4.334618	1.71E-10
280	ENSG00000261801	LOXL1-AS1	-0.97512	1.732176	1.72E-10
281	ENSG00000196584	XRCC2	1.159964	0.677027	1.93E-10
282	ENSG00000164032	H2AFZ	0.577835	7.256673	1.97E-10
283	ENSG00000102384	CENPI	1.297842	-0.17369	2.17E-10
284	ENSG00000175305	CCNE2	0.936682	2.031243	2.21E-10
285	ENSG00000138346	DNA2	0.668523	2.699251	2.24E-10
286	ENSG00000161980	POLR3K	0.633235	3.230247	2.27E-10
287	ENSG00000127423	AUNIP	1.061914	-0.27108	2.28E-10
288	ENSG00000135314	KHDC1	1.0786	0.600869	2.29E-10
289	ENSG00000181847	TIGIT	0.91718	6.459644	2.29E-10
290	ENSG00000163832	ELP6	0.515516	3.959895	2.29E-10
291	ENSG00000123975	CKS2	1.202066	3.586324	2.3E-10
292	ENSG00000123374	CDK2	0.47178	4.249606	2.58E-10
293	ENSG00000097046	CDC7	0.747996	4.317069	2.75E-10
294	ENSG00000161133	USP41	2.000253	1.269804	2.79E-10
295	ENSG00000211950	IGHV1-24	2.061735	0.507161	2.93E-10
296	ENSG00000143891	GALM	0.871256	4.647286	3.21E-10
297	ENSG00000006625	GGCT	0.669025	3.730762	3.23E-10
298	ENSG00000117650	NEK2	1.90413	1.188974	3.27E-10
299	ENSG00000104147	OIP5	1.550031	-0.70273	3.3E-10
300	ENSG00000144935	TRPC1	-1.18262	1.169699	3.56E-10
301	ENSG00000131067	GGT7	-0.73036	3.67287	3.6E-10
302	ENSG00000113088	GZMK	0.903479	6.089609	3.63E-10
303	ENSG00000178852	EFCAB13	-0.93989	1.696116	3.63E-10
304	ENSG00000080561	MID2	-1.25947	1.377514	3.64E-10
305	ENSG00000075131	TIPIN	0.682955	1.979878	3.73E-10

306	ENSG00000154920	EME1	0.907187	0.057701	3.85E-10
307	ENSG00000181894	ZNF329	-0.75647	3.475664	3.99E-10
308	ENSG00000181019	NQO1	0.977737	1.370523	4E-10
309	ENSG00000114405	C3orf14	1.070461	2.11994	4.3E-10
310	ENSG00000167670	CHAF1A	0.671196	3.862722	4.52E-10
311	ENSG00000112984	KIF20A	2.082233	-0.08254	4.55E-10
312	ENSG00000122126	OCRL	-0.78787	2.800316	4.64E-10
313	ENSG00000188389	PDCD1	1.516794	3.841247	4.93E-10
314	ENSG00000232022		-1.19139	0.72285	4.94E-10
315	ENSG00000112118	MCM3	0.933368	5.503735	5.01E-10
316	ENSG00000057294	PKP2	-1.68159	-0.03224	5.56E-10
317	ENSG00000081870	HSPB11	0.629243	4.650327	5.84E-10
318	ENSG00000211896	IGHG1	2.241534	8.425932	5.96E-10
319	ENSG00000188985	DHFRP1	1.26356	0.035339	6.16E-10
320	ENSG00000196440	ARMCX4	-0.80271	2.682954	6.16E-10
321	ENSG00000121621	KIF18A	1.087626	1.909129	6.64E-10
322	ENSG00000228716	DHFR	1.010559	4.682025	6.76E-10
323	ENSG00000099901	RANBP1	0.452244	5.176837	6.76E-10
324	ENSG00000223350	IGLV9-49	2.598199	0.080268	7.19E-10
325	ENSG00000166451	CENPN	1.140445	3.202	7.26E-10
326	ENSG00000170921	TANC2	-0.82135	4.155294	7.37E-10
327	ENSG00000134709	HOOK1	-1.04925	3.160948	7.63E-10
328	ENSG00000076248	UNG	0.677668	3.48262	7.81E-10
329	ENSG00000181751	C5orf30	0.615548	2.992649	7.97E-10
330	ENSG00000087586	AURKA	1.188146	1.819895	8.28E-10
331	ENSG00000174292	TNK1	-0.93957	1.62841	8.59E-10
332	ENSG00000134909	ARHGAP32	-0.86945	3.044992	9.65E-10
333	ENSG00000128050	PAICS	0.675077	5.339602	1.03E-09
334	ENSG00000099341	PSMD8	0.404363	6.676511	1.05E-09
335	ENSG00000008283	CYB561	-0.59622	4.424353	1.08E-09
336	ENSG00000185436	IFNLR1	0.838932	3.657568	1.09E-09
337	ENSG00000229056		-1.25271	0.964489	1.09E-09
338	ENSG00000102054	RBBP7	0.373268	6.956988	1.11E-09
339	ENSG00000203817	FAM72C	1.232412	-0.31787	1.11E-09
340	ENSG00000239975	IGKV1D-33	1.69917	2.196471	1.16E-09
341	ENSG00000244509	APOBEC3C	0.839787	6.583564	1.19E-09
342	ENSG00000213123	TCTEX1D2	0.980121	1.088006	1.19E-09
343	ENSG00000007968	E2F2	1.402987	3.176863	1.21E-09
344	ENSG00000175279	APITD1	0.820576	0.199343	1.34E-09
345	ENSG00000125124	BBS2	-0.57166	5.334921	1.4E-09
346	ENSG00000163687	DNASE1L3	-1.38107	0.290501	1.5E-09
347	ENSG00000116641	DOCK7	-0.75128	3.073678	1.58E-09
348	ENSG00000164483	SAMD3	0.847151	6.604707	1.61E-09
349	ENSG00000165806	CASP7	0.657394	5.671614	1.62E-09

350	ENSG00000135972	MRPS9	0.49942	4.09143	1.63E-09
351	ENSG00000211962	IGHV1-46	1.983136	0.875078	1.65E-09
352	ENSG00000231607	DLEU2	0.761424	2.965336	1.65E-09
353	ENSG00000242076	IGKV1-33	1.690189	2.318383	1.68E-09
354	ENSG00000136514	RTP4	1.088441	3.140253	1.68E-09
355	ENSG00000171723	GPHN	-0.57576	2.200407	1.94E-09
356	ENSG00000017483	SLC38A5	0.914824	3.86758	1.99E-09
357	ENSG00000189350	FAM179A	0.918653	3.549273	2.01E-09
358	ENSG00000173894	CBX2	1.319894	-1.1781	2.03E-09
359	ENSG00000121413	ZSCAN18	-0.88404	2.903553	2.13E-09
360	ENSG00000105171	POP4	0.512492	5.05577	2.17E-09
361	ENSG00000101158	NELFCD	0.269774	6.289552	2.28E-09
362	ENSG00000243466	IGKV1-5	1.610328	4.390031	2.38E-09
363	ENSG00000138311	ZNF365	-1.38716	2.027781	2.39E-09
364	ENSG00000211947	IGHV3-21	1.952395	2.38623	2.55E-09
365	ENSG00000104324	CPQ	-0.69219	5.338949	2.64E-09
366	ENSG00000114948	ADAM23	-1.6613	0.731224	2.69E-09
367	ENSG00000057019	DCBLD2	-0.96989	0.899906	2.75E-09
368	ENSG00000234664	HMGN2P5	0.600951	4.82702	2.8E-09
369	ENSG00000242861		-0.75933	2.60436	2.83E-09
370	ENSG00000198901	PRC1	1.06043	4.009058	2.97E-09
371	ENSG00000071242	RPS6KA2	-0.79503	2.780876	2.99E-09
372	ENSG00000205794		1.298802	-0.76504	3.07E-09
373	ENSG00000143179	UCK2	0.598991	3.404887	3.1E-09
374	ENSG00000163518	FCRL4	2.390582	-2.1935	3.15E-09
375	ENSG00000116288	PARK7	0.452912	6.950928	3.34E-09
376	ENSG00000166532	RIMKLB	-0.74854	3.951802	3.43E-09
377	ENSG00000101842	VSIG1	-1.0943	3.239147	3.43E-09
378	ENSG00000198168	SVIP	-0.59145	5.645701	4.03E-09
379	ENSG00000204789	ZNF204P	-1.20228	1.97335	4.03E-09
380	ENSG00000112242	E2F3	0.498054	5.738304	4.04E-09
381	ENSG00000185386	MAPK11	0.657567	2.479841	4.06E-09
382	ENSG00000153048	CARHSP1	0.445852	4.67034	4.07E-09
383	ENSG00000211949	IGHV3-23	1.958901	2.757262	4.19E-09
384	ENSG00000084090	STARD7	0.447956	7.553473	4.42E-09
385	ENSG00000198176	TFDP1	0.561423	6.036998	4.53E-09
386	ENSG00000133142	TCEAL4	-0.62541	3.067565	4.65E-09
387	ENSG00000146143	PRIM2	0.546807	3.259548	4.69E-09
388	ENSG00000121039	RDH10	0.655346	3.38023	5.15E-09
389	ENSG00000157077	ZFYVE9	-1.00878	1.503756	5.3E-09
390	ENSG00000198924	DCLRE1A	0.812108	3.689766	5.58E-09
391	ENSG00000122707	RECK	-0.61748	3.697262	5.6E-09
392	ENSG00000008311	AASS	-1.0006	1.591911	5.73E-09
393	ENSG00000170340	B3GNT2	0.683554	6.611026	5.8E-09

394	ENSG00000165501	LRR1	0.590607	3.675861	5.82E-09
395	ENSG00000186298	PPP1CC	0.327909	7.9838	5.84E-09
396	ENSG00000214578	HMGN2P15	0.554075	2.551196	5.95E-09
397	ENSG00000132849	INADL	-0.7858	4.472329	6.05E-09
398	ENSG00000070159	PTPN3	1.596971	-0.63742	6.17E-09
399	ENSG00000109084	TMEM97	0.642879	2.355759	6.61E-09
400	ENSG00000183690	EFHC2	-0.98055	1.553783	6.97E-09
401	ENSG00000267152		-1.12836	0.722189	7.01E-09
402	ENSG00000076382	SPAG5	0.895899	3.440711	7.01E-09
403	ENSG00000137265	IRF4	0.971171	6.486892	7.11E-09
404	ENSG00000116299	KIAA1324	-0.94513	3.05785	7.12E-09
405	ENSG00000160299	PCNT	0.602511	6.084833	7.24E-09
406	ENSG00000197540	GZMM	0.789772	6.15588	7.94E-09
407	ENSG00000113810	SMC4	0.708822	6.620605	8.13E-09
408	ENSG00000182621	PLCB1	-0.90997	3.206397	8.71E-09
409	ENSG00000089006	SNX5	0.345297	6.78575	8.74E-09
410	ENSG00000162378	ZYG11B	-0.59867	5.081231	9.11E-09
411	ENSG00000136153	LMO7	-0.68431	3.638722	9.73E-09
412	ENSG00000133119	RFC3	0.710315	3.668937	9.86E-09
413	ENSG00000110318	KIAA1377	-0.93193	1.997812	9.86E-09
414	ENSG00000035115	SH3YL1	-0.63935	5.014403	1.01E-08
415	ENSG00000260231	JHDM1D-AS1	0.756275	2.751414	1.06E-08
416	ENSG00000008226	DLEC1	-0.77451	2.071468	1.07E-08
417	ENSG00000101868	POLA1	0.718245	4.040713	1.16E-08
418	ENSG00000105173	CCNE1	0.733032	2.440994	1.17E-08
419	ENSG00000110063	DCPS	0.699798	4.144581	1.17E-08
420	ENSG00000165244	ZNF367	0.65612	3.256034	1.17E-08
421	ENSG00000211956	IGHV4-34	1.94293	2.572495	1.18E-08
422	ENSG00000230547	HMGB1P11	0.516652	2.202391	1.2E-08
423	ENSG00000142192	APP	-0.85682	6.399026	1.2E-08
424	ENSG00000261612	SUB1P3	0.696798	3.035657	1.2E-08
425	ENSG00000111962	UST	-0.57723	2.654991	1.2E-08
426	ENSG00000197299	BLM	0.570949	3.702274	1.24E-08
427	ENSG00000162591	MEGF6	-1.06389	3.240517	1.24E-08
428	ENSG00000115896	PLCL1	-0.87858	4.339564	1.24E-08
429	ENSG00000211640	IGLV6-57	2.075658	2.357621	1.27E-08
430	ENSG00000198937	CCDC167	0.74852	3.944451	1.32E-08
431	ENSG00000008282	SYPL1	-0.49934	5.871357	1.32E-08
432	ENSG00000049541	RFC2	0.488137	3.878033	1.34E-08
433	ENSG00000260549	MT1L	2.03487	-1.13192	1.37E-08
434	ENSG00000143819	EPHX1	-0.65865	2.960741	1.38E-08
435	ENSG00000197992	CLEC9A	-1.64723	-1.28446	1.4E-08
436	ENSG00000137441	FGFBP2	0.927904	7.900928	1.41E-08
437	ENSG00000074696	PTPLAD1	0.70511	5.097755	1.47E-08

438	ENSG00000197077	KIAA1671	1.090866	4.549924	1.54E-08
439	ENSG00000248988		0.94186	2.548538	1.54E-08
440	ENSG00000188486	H2AFX	0.924171	3.549308	1.58E-08
441	ENSG00000211945	IGHV1-18	1.9782	2.312352	1.64E-08
442	ENSG00000243290	IGKV1-12	1.773347	1.81345	1.83E-08
443	ENSG00000213551	DNAJC9	0.46806	4.015217	1.86E-08
444	ENSG00000176595	KBTBD11	-0.84284	3.349897	1.86E-08
445	ENSG00000156052	GNAQ	-0.67673	6.359537	1.87E-08
446	ENSG00000176894	PXMP2	0.842755	0.831049	1.88E-08
447	ENSG00000197321	SVIL	-0.63947	4.949063	1.98E-08
448	ENSG00000115233	PSMD14	0.468587	5.295026	2.05E-08
449	ENSG00000096996	IL12RB1	0.575599	5.688002	2.07E-08
450	ENSG00000154229	PRKCA	-0.70529	5.353201	2.08E-08
451	ENSG00000167862	ICT1	0.569596	3.659656	2.1E-08
452	ENSG00000211662	IGLV3-21	1.929721	3.157966	2.22E-08
453	ENSG00000008517	IL32	0.74312	8.310845	2.22E-08
454	ENSG00000198830	HMGN2	0.475046	7.271942	2.42E-08
455	ENSG00000158301	GPRASP2	-0.77415	0.731685	2.44E-08
456	ENSG00000240834	IGKV1D-12	1.722495	1.510648	2.46E-08
457	ENSG00000147324	MFHAS1	-0.66185	5.194795	2.46E-08
458	ENSG00000085465	OVGP1	-0.86443	2.024369	2.49E-08
459	ENSG00000145014	TMEM44	0.764811	1.327558	2.54E-08
460	ENSG00000006530	AGK	0.376299	5.644942	2.54E-08
461	ENSG00000138376	BARD1	0.595445	3.741525	2.58E-08
462	ENSG00000211955	IGHV3-33	1.758297	2.472787	2.61E-08
463	ENSG00000136770	DNAJC1	0.481477	5.069883	2.61E-08
464	ENSG00000137692	DCUN1D5	0.512474	3.859365	2.64E-08
465	ENSG00000134308	YWHAQ	0.40194	8.086395	2.65E-08
466	ENSG00000100307	CBX7	-0.60129	5.885269	2.65E-08
467	ENSG00000215784	FAM72D	1.08057	0.517655	2.73E-08
468	ENSG00000133106	EPSTI1	1.10444	7.006983	2.73E-08
469	ENSG00000111602	TIMELESS	0.81588	4.112397	2.75E-08
470	ENSG00000269974		1.229973	-1.05823	2.79E-08
471	ENSG00000211897	IGHG3	1.918992	6.538618	2.94E-08
472	ENSG00000163466	ARPC2	0.329119	9.763962	2.96E-08
473	ENSG00000006025	OSBPL7	0.689045	5.33062	2.97E-08
474	ENSG00000211892	IGHG4	1.99886	5.915357	3.04E-08
475	ENSG00000266999		1.526835	-1.23031	3.07E-08
476	ENSG00000139193	CD27	0.929872	4.740159	3.09E-08
477	ENSG00000150054	MPP7	-0.61232	5.544076	3.1E-08
478	ENSG00000077984	CST7	0.796995	7.78631	3.11E-08
479	ENSG00000144036	EXOC6B	-0.48928	3.750411	3.13E-08
480	ENSG00000120254	MTHFD1L	0.842613	2.892683	3.15E-08
481	ENSG00000239713	APOBEC3G	0.843534	6.966496	3.17E-08

482	ENSG00000106268	NUDT1	0.701766	3.558508	3.23E-08
483	ENSG00000111249	CUX2	-1.25118	-0.69192	3.24E-08
484	ENSG00000141570	CBX8	0.747206	1.622458	3.38E-08
485	ENSG00000079616	KIF22	0.469707	5.938807	3.58E-08
486	ENSG00000134684	YARS	0.677709	6.515297	3.58E-08
487	ENSG00000143106	PSMA5	0.593864	6.668077	3.67E-08
488	ENSG00000211598	IGKV4-1	1.637379	4.721012	3.86E-08
489	ENSG00000186310	NAP1L3	-0.93194	1.680001	3.97E-08
490	ENSG00000168005	C11orf84	0.543721	3.366137	4.02E-08
491	ENSG00000104381	GDAP1	0.643529	3.539812	4.12E-08
492	ENSG00000171606	ZNF274	-0.4551	4.895293	4.16E-08
493	ENSG00000174109	C16orf91	0.481567	3.08563	4.17E-08
494	ENSG00000169288	MRPL1	0.626396	4.526391	4.17E-08
495	ENSG00000114670	NEK11	-0.78766	0.384904	4.33E-08
496	ENSG00000233355	CHRM3-AS2	-1.05582	5.147145	4.33E-08
497	ENSG00000100911	PSME2	0.706053	7.791304	4.35E-08
498	ENSG00000211663	IGLV3-19	1.98179	3.079313	4.36E-08
499	ENSG00000116221	MRPL37	0.493908	4.887642	4.42E-08
500	ENSG00000081177	EXD2	-0.60472	2.232916	4.42E-08
501	ENSG00000250303		-1.04931	0.30226	4.42E-08
502	ENSG00000100714	MTHFD1	0.735269	4.776464	4.68E-08
503	ENSG00000136950	ARPC5L	0.491127	6.026649	4.77E-08
504	ENSG00000136213	CHST12	0.869392	4.397303	4.96E-08
505	ENSG00000240225	ZNF542	-0.73403	1.97747	5.02E-08
506	ENSG00000187837	HIST1H1C	1.006504	3.881803	5.08E-08
507	ENSG00000152455	SUV39H2	0.590791	2.371197	5.14E-08
508	ENSG00000054392	HHAT	-0.76047	1.826109	5.32E-08
509	ENSG00000167286	CD3D	0.607748	7.669562	5.33E-08
510	ENSG00000184903	IMMP2L	-0.65506	1.394705	5.48E-08
511	ENSG00000102053	ZC3H12B	-0.72663	1.056083	5.48E-08
512	ENSG00000166979	EVA1C	-0.75726	2.348887	5.48E-08
513	ENSG00000160145	KALRN	-0.89108	0.938646	5.69E-08
514	ENSG00000171617	ENC1	1.058359	7.511792	5.73E-08
515	ENSG00000155016	CYP2U1	-0.61127	2.718461	5.86E-08
516	ENSG00000187608	ISG15	1.740801	5.810074	6.16E-08
517	ENSG00000067177	PHKA1	1.055972	-0.86432	6.23E-08
518	ENSG00000095015	MAP3K1	-0.57046	8.099909	6.45E-08
519	ENSG00000107362	ABHD17B	0.660153	5.286271	6.93E-08
520	ENSG00000035664	DAPK2	0.77914	3.498756	7.01E-08
521	ENSG00000161912	ADCY10P1	-0.69151	3.338809	7.03E-08
522	ENSG00000188312	CENPP	0.648932	2.342751	7.3E-08
523	ENSG00000214222	TUBBP2	0.728546	4.018219	7.54E-08
524	ENSG00000163629	PTPN13	-0.79571	2.880864	7.65E-08
525	ENSG00000106809	OGN	1.204977	-0.81086	7.71E-08

526	ENSG00000143401	ANP32E	0.410445	7.095118	7.71E-08
527	ENSG00000000971	CFH	-1.21765	2.196042	7.71E-08
528	ENSG00000110852	CLEC2B	0.601	7.332906	8.11E-08
529	ENSG00000196550	FAM72A	0.987335	1.098463	8.45E-08
530	ENSG00000198933	TBKBP1	0.789438	3.92128	8.47E-08
531	ENSG00000187862	TTC24	0.84672	3.112404	8.51E-08
532	ENSG00000146918	NCAPG2	0.7941	4.116158	8.57E-08
533	ENSG00000165283	STOML2	0.405733	5.256326	8.57E-08
534	ENSG00000136828	RALGPS1	-0.55763	3.430564	8.62E-08
535	ENSG00000140443	IGF1R	-0.7994	4.799035	8.73E-08
536	ENSG00000128951	DUT	0.518325	5.258441	9.05E-08
537	ENSG00000139405	RITA1	0.627731	3.112246	9.55E-08
538	ENSG00000260266		0.526446	2.486925	9.69E-08
539	ENSG00000116898	MRPS15	0.368345	5.202577	9.83E-08
540	ENSG00000122873	CISD1	0.792659	2.586644	9.84E-08
541	ENSG00000064652	SNX24	-0.53859	1.862437	9.98E-08
542	ENSG00000164051	CCDC51	0.537043	2.095278	1E-07
543	ENSG00000244116	IGKV2-28	1.462186	2.920765	1.03E-07
544	ENSG00000120896	SORBS3	-0.76495	3.822977	1.03E-07
545	ENSG00000115415	STAT1	0.879852	8.881553	1.03E-07
546	ENSG00000165905	GYLTL1B	-1.13303	1.425045	1.05E-07
547	ENSG00000111775	COX6A1	0.399736	6.366285	1.06E-07
548	ENSG00000123612	ACVR1C	-0.96683	2.49725	1.08E-07
549	ENSG00000145214	DGKQ	0.649111	5.598089	1.08E-07
550	ENSG00000152240	HAUS1	0.555623	4.264109	1.08E-07
551	ENSG00000167088	SNRPD1	0.516004	5.355667	1.09E-07
552	ENSG00000211939	IGHV1-8	1.796767	0.842691	1.1E-07
553	ENSG00000117281	CD160	1.080062	4.552724	1.1E-07
554	ENSG00000160654	CD3G	0.657437	6.687979	1.11E-07
555	ENSG00000196187	TMEM63A	-0.70454	6.415545	1.11E-07
556	ENSG00000211953	IGHV3-30	1.759272	2.471179	1.12E-07
557	ENSG00000089177	KIF16B	-0.68788	3.343153	1.13E-07
558	ENSG00000073417	PDE8A	-0.57577	4.692815	1.17E-07
559	ENSG00000184384	MAML2	-0.53518	5.738371	1.2E-07
560	ENSG00000196141	SPATS2L	1.139053	3.855082	1.21E-07
561	ENSG00000159147	DONSON	0.470517	3.487767	1.21E-07
562	ENSG00000116005	PCYOX1	-0.55036	4.418247	1.22E-07
563	ENSG00000147044	CASK	-0.51918	4.601253	1.23E-07
564	ENSG00000068903	SIRT2	0.490731	6.227395	1.24E-07
565	ENSG00000182463	TSHZ2	-0.97147	2.980134	1.24E-07
566	ENSG00000109881	CCDC34	0.867923	1.339493	1.26E-07
567	ENSG00000104731	KLHDC4	0.526864	5.832745	1.32E-07
568	ENSG00000048028	USP28	0.754753	5.903484	1.33E-07
569	ENSG00000124562	SNRPC	0.399218	5.430842	1.36E-07

570	ENSG00000214402	LCNL1	-1.59672	-0.62263	1.39E-07
571	ENSG00000249149		0.608741	2.320679	1.39E-07
572	ENSG00000126217	MCF2L	-0.94158	1.592212	1.42E-07
573	ENSG00000101182	PSMA7	0.356444	7.104186	1.42E-07
574	ENSG00000196704	AMZ2	0.366271	6.225957	1.44E-07
575	ENSG00000222017		1.845859	-1.86165	1.46E-07
576	ENSG00000169861	IGHV1OR21-1	1.9375	-1.37114	1.54E-07
577	ENSG00000161981	SNRNP25	0.70672	3.833061	1.54E-07
578	ENSG00000157570	TSPAN18	-0.97964	2.979448	1.57E-07
579	ENSG00000156299	TIAM1	-0.59043	5.334777	1.6E-07
580	ENSG00000211946	IGHV3-20	2.018282	0.338779	1.6E-07
581	ENSG00000164983	TMEM65	-0.49037	4.034391	1.62E-07
582	ENSG00000119326	CTNNAL1	1.182354	2.752538	1.66E-07
583	ENSG00000242534	IGKV2D-28	1.417788	2.864497	1.67E-07
584	ENSG00000088854	C20orf194	-0.46136	4.113068	1.68E-07
585	ENSG00000162817	C1orf115	-0.93577	0.211044	1.69E-07
586	ENSG00000100852	ARHGAP5	-0.72226	5.752951	1.72E-07
587	ENSG00000204217	BMPR2	-0.43276	5.689352	1.73E-07
588	ENSG00000181929	PRKAG1	0.398671	5.502119	1.75E-07
589	ENSG00000126709	IFI6	1.515514	6.379605	1.76E-07
590	ENSG00000213402	PTPRCAP	0.735676	7.015208	1.76E-07
591	ENSG00000105426	PTPRS	-0.93668	1.40402	1.76E-07
592	ENSG00000214975	PPIAP29	0.420474	6.743436	1.8E-07
593	ENSG00000211671	IGLV2-8	1.530203	3.244851	1.81E-07
594	ENSG00000271975		-0.61893	3.150165	1.81E-07
595	ENSG00000089876	DHX32	-0.54017	3.264921	1.83E-07
596	ENSG00000170476	MZB1	1.312722	4.59224	1.85E-07
597	ENSG00000110723	EXPH5	-0.86519	0.754284	1.85E-07
598	ENSG00000151692	RNF144A	-0.65456	4.494819	1.9E-07
599	ENSG00000073861	TBX21	0.711323	5.868398	1.94E-07
600	ENSG00000197943	PLCG2	0.542546	6.386562	2.02E-07
601	ENSG00000182670	TTC3	-0.42907	7.826063	2.03E-07
602	ENSG00000253497	IGKV1-13	1.527738	0.636075	2.03E-07
603	ENSG00000205629	LCMT1	0.36067	4.220972	2.04E-07
604	ENSG00000145358	DDIT4L	1.463349	-0.54405	2.06E-07
605	ENSG00000105963	ADAP1	0.672992	4.329951	2.06E-07
606	ENSG00000107282	APBA1	-1.54352	-0.84124	2.14E-07
607	ENSG00000146858	ZC3HAV1L	1.08807	-0.17852	2.15E-07
608	ENSG00000102245	CD40LG	-0.83849	3.747895	2.15E-07
609	ENSG00000075239	ACAT1	0.533627	4.690482	2.17E-07
610	ENSG00000011523	CEP68	-0.5557	4.604643	2.18E-07
611	ENSG00000100908	EMC9	0.519966	2.526667	2.18E-07
612	ENSG00000226981	ABHD17AP6	0.620565	3.583695	2.18E-07
613	ENSG00000186648	LRRC16B	0.985397	2.722187	2.22E-07

614	ENSG00000185627	PSMD13	0.340151	6.896507	2.22E-07
615	ENSG00000154027	AK5	-1.25668	2.726385	2.23E-07
616	ENSG00000211966	IGHV5-51	1.740229	2.365693	2.29E-07
617	ENSG00000153898	MCOLN2	0.822539	5.649279	2.29E-07
618	ENSG00000113048	MRPS27	0.314046	5.569047	2.29E-07
619	ENSG00000211648	IGLV1-47	1.610013	2.890048	2.33E-07
620	ENSG00000086504	MRPL28	0.51638	4.926446	2.33E-07
621	ENSG00000167483	FAM129C	-1.03933	4.235693	2.33E-07
622	ENSG00000163535	SGOL2	0.834784	2.507049	2.37E-07
623	ENSG00000151422	FER	-0.57119	3.82351	2.37E-07
624	ENSG00000137078	SIT1	0.725399	4.394866	2.39E-07
625	ENSG00000211974	IGHV2-70	1.93591	0.058897	2.58E-07
626	ENSG00000211964	IGHV3-48	1.763489	1.220507	2.59E-07
627	ENSG00000211934	IGHV1-2	1.840786	2.151995	2.59E-07
628	ENSG00000213707	HMGB1P10	0.591778	3.477827	2.61E-07
629	ENSG00000027075	PRKCH	0.493239	8.235775	2.61E-07
630	ENSG00000179362	HMGN2P46	0.514062	3.969829	2.65E-07
631	ENSG00000197928	ZNF677	-0.85335	2.079469	2.75E-07
632	ENSG00000258741		0.761932	1.727177	2.82E-07
633	ENSG00000106153	CHCHD2	0.405666	7.043601	2.91E-07
634	ENSG00000100865	CINP	0.391306	4.050389	2.92E-07
635	ENSG00000185651	UBE2L3	0.372486	6.646849	2.92E-07
636	ENSG00000236901	MIR600HG	-0.82313	2.842907	2.92E-07
637	ENSG00000236264	RPL26P30	0.634895	1.784299	2.93E-07
638	ENSG00000225492	GBP1P1	1.199898	4.23516	3E-07
639	ENSG00000154451	GBP5	0.887204	9.165354	3E-07
640	ENSG00000112208	BAG2	0.639884	3.537983	3.01E-07
641	ENSG00000012963	UBR7	0.400777	5.325513	3.11E-07
642	ENSG00000128394	APOBEC3F	0.728518	3.670894	3.2E-07
643	ENSG00000211666	IGLV2-14	1.41471	4.490219	3.23E-07
644	ENSG00000070018	LRP6	-1.22064	1.121228	3.25E-07
645	ENSG00000164828	SUN1	-0.37926	5.892824	3.25E-07
646	ENSG00000168685	IL7R	-0.95922	9.444083	3.39E-07
647	ENSG00000121579	NAA50	0.691928	7.685676	3.49E-07
648	ENSG00000176533	GNG7	-0.70424	3.619544	3.51E-07
649	ENSG00000174136	RGMB	-0.82138	2.438062	3.53E-07
650	ENSG00000241244	IGKV1D-16	1.499482	1.487554	3.54E-07
651	ENSG00000147155	EBP	0.512042	4.950759	3.54E-07
652	ENSG00000266904	LINC00663	-0.89867	0.273757	3.54E-07
653	ENSG00000150764	DIXDC1	-1.01916	-0.09331	3.59E-07
654	ENSG00000211801	TRAV21	0.94028	1.530097	3.64E-07
655	ENSG00000123240	OPTN	0.485953	6.932707	3.64E-07
656	ENSG00000037241	RPL26L1	0.568056	2.70506	3.66E-07
657	ENSG00000152492	CCDC50	-0.49199	6.443607	3.66E-07

658	ENSG00000091409	ITGA6	-0.64652	6.23223	3.66E-07
659	ENSG00000211943	IGHV3-15	1.657211	1.602208	3.66E-07
660	ENSG00000256553	TRAV1-2	0.883057	1.319674	3.68E-07
661	ENSG00000161551	ZNF577	-0.61893	3.082712	3.71E-07
662	ENSG00000182199	SHMT2	0.452493	5.65624	3.86E-07
663	ENSG00000099282	TSPAN15	-1.18545	-0.15301	3.88E-07
664	ENSG00000143889	HNRNPLL	0.602646	5.811551	4.18E-07
665	ENSG00000172172	MRPL13	0.511024	4.186996	4.23E-07
666	ENSG00000137959	IFI44L	1.95768	7.106815	4.42E-07
667	ENSG00000139668	WDFY2	-0.48803	4.777783	4.45E-07
668	ENSG00000183684	ALYREF	0.472896	5.437284	4.5E-07
669	ENSG00000110955	ATP5B	0.287033	8.922986	4.51E-07
670	ENSG00000230633		-1.14881	-0.51753	4.67E-07
671	ENSG00000073910	FRY	-0.62781	4.979193	4.72E-07
672	ENSG00000160752	FDPS	0.332435	5.315448	4.73E-07
673	ENSG00000155115	GTF3C6	0.438793	4.857022	4.74E-07
674	ENSG00000213569		0.564191	1.080263	4.78E-07
675	ENSG00000198948	MFAP3L	-1.17439	2.915589	4.78E-07
676	ENSG00000157303	SUSD3	-0.58072	4.053244	4.78E-07
677	ENSG00000168056	LTBP3	-0.82157	4.717466	4.8E-07
678	ENSG00000048140	TSPAN17	0.645081	5.416041	4.82E-07
679	ENSG00000167005	NUDT21	0.277046	6.997717	4.82E-07
680	ENSG00000118263	KLF7	-0.63738	5.878129	4.87E-07
681	ENSG00000080546	SESN1	-0.50474	4.931277	4.88E-07
682	ENSG00000128322	IGLL1	1.423451	2.143638	4.89E-07
683	ENSG00000068489	PRR11	0.910829	3.09801	4.89E-07
684	ENSG00000169031	COL4A3	-1.14211	1.598919	5.03E-07
685	ENSG00000136856	SLC2A8	0.508724	2.358705	5.09E-07
686	ENSG00000113368	LMNB1	0.867379	6.025027	5.1E-07
687	ENSG00000100918	REC8	0.677761	5.256637	5.11E-07
688	ENSG00000146416	AIG1	-0.49141	3.433166	5.14E-07
689	ENSG00000211660	IGLV2-23	1.455876	3.905611	5.16E-07
690	ENSG00000169627	BOLA2B	0.582775	3.853127	5.19E-07
691	ENSG00000165916	PSMC3	0.364109	6.094252	5.24E-07
692	ENSG00000083720	OXCT1	0.463651	5.719897	5.38E-07
693	ENSG00000128626	MRPS12	0.505175	3.987475	5.48E-07
694	ENSG00000119318	RAD23B	0.360652	7.047674	5.53E-07
695	ENSG00000198648	STK39	0.564532	5.602072	5.54E-07
696	ENSG00000158079	PTPDC1	-0.70612	1.979074	5.55E-07
697	ENSG00000076555	ACACB	-0.60919	3.606496	5.78E-07
698	ENSG00000196839	ADA	0.863104	5.956578	5.81E-07
699	ENSG00000253173		0.605929	0.476305	5.81E-07
700	ENSG00000158555	GDPD5	-0.69627	5.051586	5.87E-07
701	ENSG00000154518	ATP5G3	0.374339	6.630222	5.89E-07

702	ENSG00000168393	DTYMK	0.622862	3.150287	5.94E-07
703	ENSG00000253755	IGHGP	1.800283	5.072211	6.01E-07
704	ENSG00000196220	SRGAP3	-0.81148	0.381525	6.06E-07
705	ENSG00000163950	SLBP	0.554442	6.050941	6.12E-07
706	ENSG00000108384	RAD51C	0.526242	3.232891	6.14E-07
707	ENSG00000171311	EXOSC1	0.39741	4.674694	6.27E-07
708	ENSG00000072071	LPHN1	-0.58861	3.776666	6.27E-07
709	ENSG00000121964	GTDC1	0.332557	5.467427	6.35E-07
710	ENSG00000165795	NDRG2	-0.88003	2.916529	6.35E-07
711	ENSG00000167083	GNGT2	0.569093	3.791282	6.39E-07
712	ENSG00000137133	HINT2	0.65355	3.303501	6.42E-07
713	ENSG00000111639	MRPL51	0.560921	5.008497	6.57E-07
714	ENSG00000224287	MSL3P1	0.592797	1.215639	6.77E-07
715	ENSG00000159055	MIS18A	0.532151	2.359604	6.79E-07
716	ENSG00000240382	IGKV1-17	1.509357	1.524781	6.79E-07
717	ENSG00000172915	NBEA	-0.78275	1.831154	6.79E-07
718	ENSG00000166595	FAM96B	0.357712	5.174495	6.86E-07
719	ENSG00000198658	ABHD17AP2	0.631098	3.529969	6.88E-07
720	ENSG00000132341	RAN	0.346467	7.267366	6.9E-07
721	ENSG00000211938	IGHV3-7	1.603719	2.491466	7.07E-07
722	ENSG00000000003	TSPAN6	-1.28456	-0.88599	7.08E-07
723	ENSG00000117602	RCAN3	-0.77588	6.534714	7.11E-07
724	ENSG00000235621	LINC00494	-1.40634	0.271792	7.15E-07
725	ENSG00000176871	WSB2	0.384544	5.68661	7.28E-07
726	ENSG00000124256	ZBP1	0.712475	5.461921	7.36E-07
727	ENSG00000256043	CTSO	-0.50504	5.566862	7.37E-07
728	ENSG00000111640	GAPDH	0.461255	9.9765	7.5E-07
729	ENSG00000135605	TEC	-0.53441	2.36822	7.56E-07
730	ENSG00000099194	SCD	1.000369	3.621902	7.75E-07
731	ENSG00000185885	IFITM1	0.733468	7.481921	7.78E-07
732	ENSG00000233609		-1.11678	-0.03571	7.8E-07
733	ENSG00000130487	KLHDC7B	1.131412	2.76686	8.02E-07
734	ENSG00000235194	PPP1R3E	-0.58635	3.886746	8.02E-07
735	ENSG00000243452	NBPF15	-0.38127	5.538226	8.15E-07
736	ENSG00000136930	PSMB7	0.334953	6.285269	8.31E-07
737	ENSG00000104889	RNASEH2A	0.812577	2.46974	8.37E-07
738	ENSG00000231292	IGKV1OR2-108	1.579241	-0.63283	8.56E-07
739	ENSG00000110841	PPFIBP1	-0.62695	0.771629	8.59E-07
740	ENSG00000147180	ZNF711	-0.78005	1.67091	8.59E-07
741	ENSG00000139618	BRCA2	0.83806	3.373074	8.62E-07
742	ENSG00000167711	SERPINF2	-0.84265	0.005739	8.62E-07
743	ENSG00000167642	SPINT2	-0.63849	4.547739	8.84E-07
744	ENSG00000100296	THOC5	0.299011	4.973006	8.85E-07
745	ENSG00000259673	IQCH-AS1	-0.69474	2.08835	8.85E-07

746	ENSG00000206530	WDR52	-0.74708	3.77123	8.87E-07
747	ENSG00000266088		0.743603	2.282194	8.89E-07
748	ENSG00000100263	RHBDD3	0.454542	2.59526	8.95E-07
749	ENSG00000134242	PTPN22	0.678835	7.206219	8.95E-07
750	ENSG00000178947	LINC00086	-1.16854	-0.73602	8.95E-07
751	ENSG00000099917	MED15	0.454779	7.057833	8.96E-07
752	ENSG00000248626	GAPDHP40	0.52987	4.112591	8.98E-07
753	ENSG00000184924	PTRHD1	0.484667	3.925763	9.02E-07
754	ENSG00000251235		0.723771	-0.14961	9.03E-07
755	ENSG00000125995	ROMO1	0.521058	4.081518	9.03E-07
756	ENSG00000154153	FAM134B	-0.81229	4.557059	9.03E-07
757	ENSG00000211772	TRBC2	0.486168	9.635175	9.03E-07
758	ENSG00000124541	RRP36	0.333774	4.8427	9.09E-07
759	ENSG00000069974	RAB27A	0.407776	7.395235	9.11E-07
760	ENSG00000125962	ARMCX5	-0.40828	3.933851	9.12E-07
761	ENSG00000186854	TRABD2A	-0.8402	5.707749	9.18E-07
762	ENSG00000092010	PSME1	0.458576	8.162596	9.26E-07
763	ENSG00000138316	ADAMTS14	1.268741	-0.58852	9.27E-07
764	ENSG00000157978	LDLRAP1	-0.69121	5.786787	9.27E-07
765	ENSG00000178038	ALS2CL	-0.9896	3.247809	9.27E-07
766	ENSG00000224078	SNHG14	-0.5158	5.540829	9.4E-07
767	ENSG00000117228	GBP1	1.251028	7.329192	9.48E-07
768	ENSG00000236124	HMGN2P23	0.651224	0.533756	9.48E-07
769	ENSG00000162639	HENMT1	0.428461	5.011153	9.51E-07
770	ENSG00000169432	SCN9A	-1.19289	0.620572	9.53E-07
771	ENSG00000211595	IGKJ3	1.356005	3.043382	9.59E-07
772	ENSG00000091483	FH	0.409	4.973777	9.69E-07
773	ENSG00000170074	FAM153A	-1.22593	3.050374	9.69E-07
774	ENSG00000217094	PPIAP31	0.546259	1.19335	9.94E-07
775	ENSG00000166788	SAAL1	0.380854	3.972605	9.94E-07
776	ENSG00000061676	NCKAP1	-0.64442	3.195801	9.97E-07
777	ENSG00000152127	MGAT5	-0.52967	6.232822	1E-06
778	ENSG00000117411	B4GALT2	0.709637	1.385937	1.02E-06
779	ENSG00000218502	H2AFZP3	0.494991	2.540781	1.03E-06
780	ENSG00000163508	EOMES	1.073254	5.093768	1.04E-06
781	ENSG00000115541	HSPE1	0.5832	5.472704	1.04E-06
782	ENSG00000129255	MPDU1	0.432079	5.614715	1.06E-06
783	ENSG00000183010	PYCR1	1.366578	-0.25922	1.06E-06
784	ENSG00000148848	ADAM12	-1.12104	1.120999	1.07E-06
785	ENSG00000063180	CA11	-0.52475	2.554525	1.07E-06
786	ENSG00000103056	SMPD3	-0.62562	2.885119	1.09E-06
787	ENSG00000232354	VIPR1-AS1	-1.23149	-0.88993	1.1E-06
788	ENSG00000005187	ACSM3	-0.56705	1.711442	1.11E-06
789	ENSG00000123836	PFKFB2	-0.57646	3.834136	1.14E-06

790	ENSG00000231331		2.100505	-2.04492	1.14E-06
791	ENSG00000052126	PLEKHA5	-0.95624	2.700153	1.14E-06
792	ENSG00000134321	RSAD2	1.903659	5.353028	1.15E-06
793	ENSG00000184613	NELL2	-0.99225	6.171557	1.15E-06
794	ENSG00000144712	CAND2	-1.11276	-0.22042	1.15E-06
795	ENSG00000225131	PSME2P2	0.74194	1.776486	1.18E-06
796	ENSG00000087053	MTMR2	0.365892	4.878833	1.19E-06
797	ENSG00000184076	UQCR10	0.415635	5.454499	1.19E-06
798	ENSG00000137266	SLC22A23	-0.70334	1.995605	1.2E-06
799	ENSG00000198178	CLEC4C	-1.24808	0.18114	1.2E-06
800	ENSG00000211649	IGLV7-46	1.451763	1.192747	1.23E-06
801	ENSG00000166387	PPFIBP2	-0.52125	4.014258	1.24E-06
802	ENSG00000100116	GCAT	1.022328	-0.27716	1.26E-06
803	ENSG00000186231	KLHL32	-0.88924	0.093099	1.27E-06
804	ENSG00000142627	EPHA2	-1.34378	-0.58107	1.28E-06
805	ENSG00000152969	JAKMIP1	0.924035	4.338924	1.28E-06
806	ENSG00000065717	TLE2	-0.898	1.66967	1.29E-06
807	ENSG00000196262	PPIA	0.329834	9.095816	1.3E-06
808	ENSG00000100897	DCAF11	0.289936	6.608622	1.31E-06
809	ENSG00000211752	TRBV27	0.880949	2.165899	1.32E-06
810	ENSG00000170540	ARL6IP1	0.462244	7.83991	1.35E-06
811	ENSG00000185920	PTCH1	-0.81283	4.301468	1.36E-06
812	ENSG00000085377	PREP	0.33511	5.253412	1.37E-06
813	ENSG00000124374	PAIP2B	-0.80528	2.454711	1.38E-06
814	ENSG00000198932	GPRASP1	-0.86119	4.959217	1.38E-06
815	ENSG00000137547	MRPL15	0.518439	4.196821	1.39E-06
816	ENSG00000145494	NDUFS6	0.460475	4.316538	1.39E-06
817	ENSG00000135114	OASL	1.395193	5.802068	1.39E-06
818	ENSG00000176974	SHMT1	0.509374	3.411418	1.4E-06
819	ENSG00000183723	CMTM4	-0.85816	2.296007	1.4E-06
820	ENSG00000113387	SUB1	0.417136	8.46569	1.41E-06
821	ENSG00000102349	KLF8	-0.82883	2.982274	1.42E-06
822	ENSG00000082269	FAM135A	-0.54337	3.668256	1.42E-06
823	ENSG00000187240	DYNC2H1	-0.72783	2.598113	1.43E-06
824	ENSG00000100632	ERH	0.391617	6.397108	1.44E-06
825	ENSG00000255987		0.814604	2.107814	1.47E-06
826	ENSG00000145390	USP53	-0.68346	4.613692	1.48E-06
827	ENSG00000102575	ACP5	0.566722	5.089373	1.51E-06
828	ENSG00000136261	BZW2	0.338513	4.972242	1.56E-06
829	ENSG00000120915	EPHX2	-0.8477	3.508471	1.56E-06
830	ENSG00000178741	COX5A	0.504249	5.35708	1.61E-06
831	ENSG00000169248	CXCL11	2.031557	-0.3055	1.63E-06
832	ENSG00000211792	TRAV14DV4	1.073561	1.791481	1.64E-06
833	ENSG00000144283	PKP4	-0.52671	3.526484	1.65E-06

834	ENSG00000211599	IGKV5-2	1.81132	-0.63824	1.66E-06
835	ENSG00000147408	CSGALNACT1	-0.63829	5.166494	1.67E-06
836	ENSG00000188732	FAM221A	-0.4575	2.31617	1.7E-06
837	ENSG00000151914	DST	-0.71726	3.873425	1.7E-06
838	ENSG00000158715	SLC45A3	-1.08263	2.113168	1.7E-06
839	ENSG00000255291		0.446787	2.43403	1.73E-06
840	ENSG00000072110	ACTN1	-0.62374	5.807178	1.73E-06
841	ENSG00000150627	WDR17	-0.85191	0.87375	1.73E-06
842	ENSG00000119718	EIF2B2	0.392579	3.8561	1.74E-06
843	ENSG00000137942	FNBP1L	-0.73243	0.749887	1.74E-06
844	ENSG00000188596	C12orf55	1.086012	-1.15475	1.74E-06
845	ENSG00000196275	GTF2IRD2	-0.4441	3.760276	1.74E-06
846	ENSG00000197959	DNM3	-0.75934	2.02624	1.75E-06
847	ENSG00000149582	TMEM25	-0.7107	1.842058	1.76E-06
848	ENSG00000167081	PBX3	-0.3656	4.310401	1.78E-06
849	ENSG00000204271	SPIN3	-0.86484	0.955476	1.78E-06
850	ENSG00000182180	MRPS16	0.297487	5.350997	1.78E-06
851	ENSG00000116830	TTF2	0.356279	4.439612	1.78E-06
852	ENSG00000132780	NASP	0.452761	6.47172	1.79E-06
853	ENSG00000041357	PSMA4	0.551002	7.145729	1.85E-06
854	ENSG00000119632	IFI27L2	0.688972	4.24044	1.86E-06
855	ENSG00000080200	CRYBG3	-0.85088	2.391676	1.86E-06
856	ENSG00000115750	TAF1B	0.417539	4.341299	1.86E-06
857	ENSG00000130940	CASZ1	0.748363	3.517414	1.87E-06
858	ENSG00000111696	NT5DC3	-0.57012	2.537166	1.87E-06
859	ENSG00000171101	SIGLEC17P	-1.22223	2.021013	1.88E-06
860	ENSG00000234231		0.307082	4.200944	1.89E-06
861	ENSG00000241755	IGKV1-9	1.43067	2.176909	1.9E-06
862	ENSG00000115840	SLC25A12	0.361786	4.647945	1.91E-06
863	ENSG00000147853	AK3	0.345562	5.512093	1.92E-06
864	ENSG00000211959	IGHV4-39	1.666235	2.406491	1.97E-06
865	ENSG00000069702	TGFBR3	0.684063	7.18231	1.97E-06
866	ENSG00000213370	RANP6	0.420587	2.801548	1.97E-06
867	ENSG00000134825	TMEM258	0.395935	5.5735	1.98E-06
868	ENSG00000142230	SAE1	0.45665	5.755062	2.05E-06
869	ENSG00000203835	ABHD17AP1	0.61281	3.527576	2.05E-06
870	ENSG00000260947		-1.27987	-0.5431	2.06E-06
871	ENSG00000125611	CHCHD5	0.518525	3.201391	2.06E-06
872	ENSG00000183617	MRPL54	0.487517	5.25933	2.13E-06
873	ENSG00000197978	GOLGA6L9	-0.75739	0.924208	2.21E-06
874	ENSG00000175550	DRAP1	0.548267	5.95906	2.23E-06
875	ENSG00000132963	POMP	0.527249	6.406035	2.25E-06
876	ENSG00000136490	LIMD2	0.322496	8.457099	2.32E-06
877	ENSG00000081377	CDC14B	-0.66758	3.442611	2.32E-06

878	ENSG00000251546	IGKV1D-39	1.661727	2.566874	2.33E-06
879	ENSG00000233452	STXBP5-AS1	-0.71032	0.719586	2.37E-06
880	ENSG00000198478	SH3BGRL2	-1.13086	3.780827	2.38E-06
881	ENSG00000115355	CCDC88A	-0.5922	6.129698	2.4E-06
882	ENSG00000185880	TRIM69	0.823495	5.745249	2.41E-06
883	ENSG00000156802	ATAD2	0.667153	5.613674	2.41E-06
884	ENSG00000127589	TUBBP1	0.654485	2.990204	2.42E-06
885	ENSG00000100346	CACNA1I	-0.95343	3.787133	2.42E-06
886	ENSG00000272529		-0.79337	2.019296	2.43E-06
887	ENSG00000084764	MAPRE3	-0.57475	1.427573	2.46E-06
888	ENSG00000096063	SRPK1	0.316001	5.820131	2.48E-06
889	ENSG00000198467	TPM2	-0.69415	3.165442	2.5E-06
890	ENSG00000102174	PHEX	-0.81056	0.02586	2.5E-06
891	ENSG00000239855	IGKV1-6	1.570408	0.964546	2.59E-06
892	ENSG00000140740	UQCRC2	0.305047	7.477304	2.68E-06
893	ENSG00000234814	SVILP1	-0.59027	1.112702	2.68E-06
894	ENSG00000102098	SCML2	-0.59159	0.217523	2.71E-06
895	ENSG00000117020	AKT3	-0.6646	5.096036	2.76E-06
896	ENSG00000165138	ANKS6	-0.5794	3.198247	2.81E-06
897	ENSG00000116771	AGMAT	0.625327	2.421906	2.85E-06
898	ENSG00000159210	SNF8	0.300722	5.436829	2.88E-06
899	ENSG00000198420	FAM115A	-0.72905	3.465936	2.89E-06
900	ENSG00000262814	MRPL12	0.629578	2.260231	2.95E-06
901	ENSG00000240864	IGKV1-16	1.395396	1.862031	3.03E-06
902	ENSG00000198356	ASNA1	0.401596	5.50784	3.04E-06
903	ENSG00000182983	ZNF662	-1.08014	0.415403	3.05E-06
904	ENSG00000117280	RAB7L1	0.474522	6.872629	3.09E-06
905	ENSG00000137100	DCTN3	0.348362	5.376819	3.09E-06
906	ENSG00000136111	TBC1D4	-0.61942	6.435258	3.09E-06
907	ENSG00000168288	MMADHC	0.413155	6.521142	3.09E-06
908	ENSG00000196152	ZNF79	0.572546	2.649519	3.09E-06
909	ENSG00000074966	TXK	-0.62385	5.487847	3.1E-06
910	ENSG00000140853	NLRC5	0.38848	8.599133	3.12E-06
911	ENSG00000166086	JAM3	-0.60817	3.20345	3.14E-06
912	ENSG00000163617	KIAA1407	-0.60682	3.193162	3.17E-06
913	ENSG00000235084	CHCHD2P6	0.521503	3.780248	3.18E-06
914	ENSG00000211651	IGLV1-44	1.468668	2.805243	3.21E-06
915	ENSG00000100084	HIRA	0.498876	4.040323	3.23E-06
916	ENSG00000104205	SGK3	-0.48112	3.622512	3.25E-06
917	ENSG00000267488		1.361475	0.799289	3.25E-06
918	ENSG00000211941	IGHV3-11	1.641148	1.735075	3.26E-06
919	ENSG00000116030	SUMO1	0.389156	6.982356	3.29E-06
920	ENSG00000184307	ZDHHC23	-0.61266	2.790849	3.29E-06
921	ENSG00000137965	IFI44	1.253714	6.818205	3.29E-06

922	ENSG00000196814	MVB12B	0.621156	4.616982	3.29E-06
923	ENSG00000168275	COA6	0.527242	3.379383	3.29E-06
924	ENSG00000183150	GPR19	0.681153	0.395354	3.33E-06
925	ENSG00000101911	PRPS2	0.402387	4.728364	3.33E-06
926	ENSG00000155636	RBM45	0.363639	3.746447	3.34E-06
927	ENSG00000136104	RNASEH2B	0.412997	5.399339	3.42E-06
928	ENSG00000133401	PDZD2	-0.56164	0.775916	3.44E-06
929	ENSG00000231744		0.542226	0.492511	3.45E-06
930	ENSG00000181652	ATG9B	-0.79975	1.100413	3.45E-06
931	ENSG00000265415		0.956915	0.118381	3.45E-06
932	ENSG00000026751	SLAMF7	0.936321	7.504017	3.45E-06
933	ENSG00000150779	TIMM8B	0.547973	4.023126	3.47E-06
934	ENSG00000151503	NCAPD3	0.40328	4.96611	3.47E-06
935	ENSG00000135960	EDAR	-1.11948	2.228294	3.47E-06
936	ENSG00000259781		0.612414	3.688292	3.49E-06
937	ENSG00000116675	DNAJC6	-1.0107	0.314863	3.49E-06
938	ENSG00000211805	TRAV24	1.058656	-0.27519	3.49E-06
939	ENSG00000118513	MYB	0.565349	2.880676	3.52E-06
940	ENSG00000129235	TXNDC17	0.493236	4.154821	3.52E-06
941	ENSG00000157601	MX1	1.187241	6.982718	3.54E-06
942	ENSG00000162194	C11orf48	0.363095	4.575066	3.54E-06
943	ENSG00000162607	USP1	0.36972	6.477755	3.6E-06
944	ENSG00000079263	SP140	0.510942	5.842243	3.61E-06
945	ENSG00000172260	NEGR1	-1.15363	-0.40653	3.62E-06
946	ENSG00000008256	CYTH3	-0.64647	3.407796	3.62E-06
947	ENSG00000169139	UBE2V2	0.464683	5.358507	3.63E-06
948	ENSG00000110801	PSMD9	0.285827	3.974712	3.63E-06
949	ENSG00000182230	FAM153B	-1.15031	2.629454	3.65E-06
950	ENSG00000010803	SCMH1	-0.37185	3.690042	3.66E-06
951	ENSG00000154764	WNT7A	-1.41344	1.250811	3.71E-06
952	ENSG00000116212	LRRC42	0.432934	3.26551	3.73E-06
953	ENSG00000104756	KCTD9	0.458176	5.317505	3.75E-06
954	ENSG00000160111	CPAMD8	-0.98868	1.189738	3.81E-06
955	ENSG00000211694	TRGV10	0.776425	3.587031	3.81E-06
956	ENSG00000109323	MANBA	-0.39089	5.951772	3.84E-06
957	ENSG00000160447	PKN3	0.635912	1.219781	3.86E-06
958	ENSG00000166794	PPIB	0.404666	6.202843	3.88E-06
959	ENSG00000160256	FAM207A	0.629343	2.322996	3.91E-06
960	ENSG00000197982	C1orf122	0.514757	3.625647	3.94E-06
961	ENSG00000155970	MICU3	-0.99752	1.389965	3.96E-06
962	ENSG00000168268	NT5DC2	0.895751	2.627661	3.97E-06
963	ENSG00000221963	APOL6	0.576079	8.462226	3.99E-06
964	ENSG00000121570	DPPA4	-0.88397	0.2616	4.02E-06
965	ENSG00000125319	C17orf53	0.830973	-0.09259	4.1E-06

966	ENSG00000242371	IGKV1-39	1.680089	2.758739	4.16E-06
967	ENSG00000196526	AFAP1	-0.7259	3.153817	4.17E-06
968	ENSG00000138796	HADH	0.435305	4.069381	4.21E-06
969	ENSG00000196507	TCEAL3	-0.52292	1.628069	4.22E-06
970	ENSG00000248546	ANP32C	0.653689	1.358971	4.26E-06
971	ENSG00000112667	DNPH1	0.552912	2.70589	4.26E-06
972	ENSG00000149970	CNKSR2	-0.69674	2.518242	4.29E-06
973	ENSG00000211951	IGHV2-26	1.928059	-1.07015	4.29E-06
974	ENSG00000048462	TNFRSF17	1.441396	2.628118	4.29E-06
975	ENSG00000246363		-1.43578	0.355423	4.34E-06
976	ENSG00000156475	PPP2R2B	0.623484	4.140733	4.35E-06
977	ENSG00000188177	ZC3H6	-0.48883	4.493315	4.35E-06
978	ENSG00000211970	IGHV4-61	1.605316	1.450063	4.35E-06
979	ENSG00000143815	LBR	0.42431	7.541264	4.4E-06
980	ENSG00000101474	APMAP	0.390571	7.552288	4.42E-06
981	ENSG00000196411	EPHB4	-0.72647	1.822887	4.43E-06
982	ENSG00000225720		1.018227	1.471852	4.44E-06
983	ENSG00000135622	SEMA4F	-0.49395	2.836574	4.44E-06
984	ENSG00000059573	ALDH18A1	0.496131	4.750127	4.45E-06
985	ENSG00000189238	LINC00943	-1.15947	0.767762	4.46E-06
986	ENSG00000197586	ENTPD6	-0.3307	5.377972	4.48E-06
987	ENSG00000138449	SLC40A1	-1.03747	4.947764	4.49E-06
988	ENSG00000189403	HMGB1	0.350539	9.312232	4.52E-06
989	ENSG00000133328	HRASLS2	1.221643	0.542994	4.53E-06
990	ENSG00000248641	HMGA1P2	0.632193	1.335229	4.54E-06
991	ENSG00000119866	BCL11A	-0.60407	3.263986	4.56E-06
992	ENSG00000100629	CEP128	0.536446	3.319431	4.72E-06
993	ENSG00000163170	BOLA3	0.535738	2.555559	4.73E-06
994	ENSG00000187118	CMC1	0.695906	5.994585	4.75E-06
995	ENSG00000120334	CENPL	0.574585	2.929453	4.8E-06
996	ENSG00000211630	IGKV1D-13	1.300673	1.479867	4.81E-06
997	ENSG00000105379	ETFB	0.515606	4.3644	4.81E-06
998	ENSG00000136717	BIN1	0.434832	6.490668	4.85E-06
999	ENSG00000120437	ACAT2	0.394646	4.298939	4.86E-06
1000	ENSG00000132465	IGJ	1.262939	8.614958	4.87E-06
1001	ENSG00000100154	TTC28	-0.85135	0.692892	4.92E-06
1002	ENSG00000103121	CMC2	0.515503	5.137856	4.99E-06
1003	ENSG00000245146	LINC01024	-0.62231	1.254413	5E-06
1004	ENSG00000144218	AFF3	-0.84343	3.928667	5.06E-06
1005	ENSG00000176208	ATAD5	0.506929	3.36334	5.09E-06
1006	ENSG00000143774	GUK1	0.443082	7.493106	5.11E-06
1007	ENSG00000225733	FGD5-AS1	-0.21489	7.486507	5.12E-06
1008	ENSG00000155368	DBI	0.465796	6.505838	5.17E-06
1009	ENSG00000242580	IGKV1D-43	1.644113	0.04144	5.18E-06

1010	ENSG00000184164	CRELD2	0.397586	4.632476	5.24E-06
1011	ENSG00000182489	XKRX	-1.23276	-1.37905	5.27E-06
1012	ENSG00000135250	SRPK2	0.33677	6.329107	5.3E-06
1013	ENSG00000260027	HOXB7	1.115413	-1.03105	5.33E-06
1014	ENSG00000136122	BORA	0.387855	3.702311	5.33E-06
1015	ENSG00000156374	PCGF6	0.460935	3.162326	5.35E-06
1016	ENSG00000137494	ANKRD42	-0.35754	3.395194	5.35E-06
1017	ENSG00000224373	IGHV4-59	1.517484	1.726071	5.36E-06
1018	ENSG00000228335		0.503552	2.481622	5.36E-06
1019	ENSG00000078487	ZCWPW1	-0.37012	2.930086	5.36E-06
1020	ENSG00000130589	HELZ2	0.798232	6.021023	5.41E-06
1021	ENSG00000231475	IGHV4-31	1.844844	0.753486	5.41E-06
1022	ENSG00000107317	PTGDS	-1.01999	2.859769	5.41E-06
1023	ENSG00000186654	PRR5	0.547644	4.362677	5.44E-06
1024	ENSG00000115902	SLC1A4	1.329213	5.715705	5.54E-06
1025	ENSG00000125144	MT1G	1.305068	0.703216	5.54E-06
1026	ENSG00000168826	ZBTB49	0.51933	4.163855	5.55E-06
1027	ENSG00000138604	GLCE	-0.68247	3.189528	5.58E-06
1028	ENSG00000189159	HN1	0.542374	5.52733	5.61E-06
1029	ENSG00000145730	PAM	0.399419	4.950345	5.65E-06
1030	ENSG00000078596	ITM2A	0.579601	7.677486	5.66E-06
1031	ENSG00000175792	RUVBL1	0.54233	4.923293	5.66E-06
1032	ENSG00000273131		-0.436	4.40282	5.66E-06
1033	ENSG00000261061		1.090554	0.08081	5.73E-06
1034	ENSG00000103018	CYB5B	0.376501	5.775523	5.75E-06
1035	ENSG00000110171	TRIM3	-0.40868	3.348195	5.77E-06
1036	ENSG00000111674	ENO2	-0.57518	4.875787	5.79E-06
1037	ENSG00000168434	COG7	0.278278	3.92154	5.79E-06
1038	ENSG00000172183	ISG20	0.592398	7.012594	5.9E-06
1039	ENSG00000184677	ZBTB40	-0.41027	6.40141	5.92E-06
1040	ENSG00000179294	C17orf96	0.979534	0.522468	5.97E-06
1041	ENSG00000225151	GOLGA2P7	-0.66177	1.191495	6.06E-06
1042	ENSG00000181104	F2R	0.664664	6.095868	6.08E-06
1043	ENSG00000211721	TRBV6-5	0.649939	2.397028	6.08E-06
1044	ENSG00000211652	IGLV7-43	1.507458	1.012911	6.09E-06
1045	ENSG00000136854	STXBP1	-0.67108	0.60155	6.14E-06
1046	ENSG00000137309	HMGA1	0.650818	6.37789	6.17E-06
1047	ENSG00000167912		0.606638	2.033009	6.27E-06
1048	ENSG00000197536	C5orf56	0.444018	5.031058	6.27E-06
1049	ENSG00000197776	KLHDC1	-0.64288	2.166446	6.29E-06
1050	ENSG00000250536	ABHD17AP3	0.592879	2.513798	6.32E-06
1051	ENSG00000051596	THOC3	0.365075	4.107994	6.32E-06
1052	ENSG00000138722	MMRN1	-1.18307	1.414337	6.32E-06
1053	ENSG00000076003	MCM6	0.953	6.203906	6.39E-06

1054	ENSG00000114054	PCCB	0.282118	4.195212	6.47E-06
1055	ENSG00000014641	MDH1	0.323134	6.731932	6.5E-06
1056	ENSG00000170291	ELP5	0.275102	4.620314	6.5E-06
1057	ENSG00000267369		0.516633	4.602893	6.65E-06
1058	ENSG00000205220	PSMB10	0.539259	5.308382	6.65E-06
1059	ENSG00000234614		-0.8421	0.412211	6.66E-06
1060	ENSG00000143155	TIPRL	0.413904	5.19871	6.68E-06
1061	ENSG00000117395	EBNA1BP2	0.360518	4.372289	6.7E-06
1062	ENSG00000162775	RBM15	0.399142	5.595628	6.73E-06
1063	ENSG00000100320	RBFOX2	-1.18082	-0.17535	6.77E-06
1064	ENSG00000141101	NOB1	0.31586	4.840041	6.82E-06
1065	ENSG00000115758	ODC1	0.447408	7.169628	6.93E-06
1066	ENSG00000125885	MCM8	0.585558	3.167412	7E-06
1067	ENSG00000172057	ORMDL3	0.461876	6.702972	7.02E-06
1068	ENSG00000157107	FCHO2	-0.58248	4.266942	7.02E-06
1069	ENSG00000175643	RMI2	0.926538	1.24202	7.05E-06
1070	ENSG00000146904	EPHA1	-0.64231	2.377698	7.05E-06
1071	ENSG00000088538	DOCK3	-0.71962	1.454699	7.05E-06
1072	ENSG00000211696	TRGV8	0.818347	1.848655	7.1E-06
1073	ENSG00000074657	ZNF532	-0.50933	3.62735	7.24E-06
1074	ENSG00000215749	GOLGA6L18	-0.69501	0.827214	7.24E-06
1075	ENSG00000128872	TMOD2	-0.82211	4.791774	7.32E-06
1076	ENSG00000142507	PSMB6	0.357865	5.953813	7.36E-06
1077	ENSG00000068079	IFI35	0.912625	5.298596	7.63E-06
1078	ENSG00000181991	MRPS11	0.409018	4.091992	7.63E-06
1079	ENSG00000106415	GLCCI1	0.630138	5.823444	7.7E-06
1080	ENSG00000197558	SSPO	-1.05012	0.567123	7.76E-06
1081	ENSG00000257151	PWAR6	-0.77044	3.304359	7.8E-06
1082	ENSG00000227051	C14orf132	-1.45485	0.548854	7.8E-06
1083	ENSG00000170270	C14orf142	0.48451	3.146079	7.83E-06
1084	ENSG00000197375	SLC22A5	-0.62742	2.77255	7.91E-06
1085	ENSG00000128581	RABL5	0.530583	1.280724	7.92E-06
1086	ENSG00000211933	IGHV6-1	1.976626	0.350106	7.92E-06
1087	ENSG00000262714		-1.37747	-0.27271	8.1E-06
1088	ENSG00000182481	KPNA2	0.748979	6.012611	8.11E-06
1089	ENSG00000162654	GBP4	0.788931	7.695981	8.21E-06
1090	ENSG00000166562	SEC11C	0.451209	5.701952	8.21E-06
1091	ENSG00000162650	ATXN7L2	0.440848	3.752448	8.23E-06
1092	ENSG00000211658	IGLV3-27	1.774084	-0.39472	8.34E-06
1093	ENSG00000080824	HSP90AA1	0.609255	10.27234	8.38E-06
1094	ENSG00000174428	GTF2IRD2B	-0.40397	3.860568	8.38E-06
1095	ENSG00000163599	CTLA4	0.61647	5.110681	8.48E-06
1096	ENSG00000151715	TMEM45B	-0.94162	1.106455	8.5E-06
1097	ENSG00000211644	IGLV1-51	1.474847	2.971627	8.63E-06

1098	ENSG00000135631	RAB11FIP5	-0.76084	4.16705	8.67E-06
1099	ENSG00000110080	ST3GAL4	0.52844	3.847169	8.77E-06
1100	ENSG00000131781	FMO5	-0.76262	2.707245	8.81E-06
1101	ENSG00000124215	CDH26	-0.86895	0.051644	8.85E-06
1102	ENSG00000105671	DDX49	0.281401	4.848985	8.88E-06
1103	ENSG00000165629	ATP5C1	0.383028	7.153016	9.04E-06
1104	ENSG00000114739	ACVR2B	-0.56443	2.70835	9.05E-06
1105	ENSG00000186615	KTN1-AS1	-0.75674	-0.43724	9.05E-06
1106	ENSG00000219481	NBPF1	-0.39235	5.408496	9.09E-06
1107	ENSG00000248019	FAM13A-AS1	-0.49768	4.043594	9.09E-06
1108	ENSG00000156587	UBE2L6	0.763186	7.149432	9.17E-06
1109	ENSG00000022277	RTFDC1	0.277276	6.444726	9.17E-06
1110	ENSG00000211637	IGLV4-69	1.432749	1.418691	9.22E-06
1111	ENSG00000160321	ZNF208	-1.1804	0.451499	9.22E-06
1112	ENSG00000189057	FAM111B	1.061257	2.972945	9.23E-06
1113	ENSG00000106399	RPA3	0.489084	4.417117	9.37E-06
1114	ENSG00000235587	GAPDHP65	0.470464	3.458256	9.37E-06
1115	ENSG00000162599	NFIA	-0.62215	2.042757	9.38E-06
1116	ENSG00000216657		0.452013	1.20512	9.44E-06
1117	ENSG00000183742	MACC1	0.815248	0.879988	9.46E-06
1118	ENSG00000110422	HIPK3	-0.33628	7.809668	9.53E-06
1119	ENSG00000105409	ATP1A3	0.750456	3.350279	9.56E-06
1120	ENSG00000010017	RANBP9	0.285197	6.130255	9.59E-06
1121	ENSG00000119335	SET	0.26257	8.434674	9.75E-06
1122	ENSG00000117560	FASLG	1.407129	3.677293	9.76E-06
1123	ENSG00000155367	PPM1J	-1.0221	-0.86111	9.93E-06
1124	ENSG00000224411		0.611324	5.611727	9.95E-06
1125	ENSG00000105810	CDK6	0.369717	6.182386	1E-05
1126	ENSG00000165533	TTC8	-0.54791	0.856904	1.04E-05
1127	ENSG00000099800	TIMM13	0.395505	4.016035	1.05E-05
1128	ENSG00000162415	ZSWIM5	-0.89329	0.727266	1.05E-05
1129	ENSG00000049656	CLPTM1L	0.317908	6.215703	1.06E-05
1130	ENSG00000246090		-0.76531	0.334528	1.06E-05
1131	ENSG00000116983	HPCAL4	-0.75459	3.063051	1.06E-05
1132	ENSG00000126453	BCL2L12	0.395011	2.633026	1.07E-05
1133	ENSG00000268758	EMR4P	-0.9944	2.032302	1.07E-05
1134	ENSG00000004660	CAMKK1	-0.51273	3.36327	1.07E-05
1135	ENSG00000110660	SLC35F2	0.525099	3.3774	1.1E-05
1136	ENSG00000161381	PLXDC1	-0.84443	3.482898	1.1E-05
1137	ENSG00000139946	PELI2	-0.56793	5.477509	1.1E-05
1138	ENSG00000258581		0.526179	2.002589	1.1E-05
1139	ENSG00000134056	MRPS36	0.376656	4.265415	1.1E-05
1140	ENSG00000160563	MED27	0.334059	3.842502	1.1E-05
1141	ENSG00000140287	HDC	-1.07267	4.222873	1.1E-05

1142	ENSG00000143412	ANXA9	-1.05248	0.523836	1.11E-05
1143	ENSG00000258759		0.479592	1.717302	1.14E-05
1144	ENSG00000260159		0.535762	0.810218	1.14E-05
1145	ENSG00000131495	NDUFA2	0.411697	5.228435	1.15E-05
1146	ENSG00000213148		0.710826	0.119431	1.16E-05
1147	ENSG00000211594	IGKJ4	1.205105	4.198626	1.17E-05
1148	ENSG00000165521	EML5	-0.55805	3.08991	1.17E-05
1149	ENSG00000271178	IGHV3OR16-13	1.427586	0.992969	1.19E-05
1150	ENSG00000143674		-0.60993	1.459297	1.2E-05
1151	ENSG00000067840	PDZD4	-0.87753	4.366166	1.2E-05
1152	ENSG00000156017	C9orf41	0.454253	4.628371	1.2E-05
1153	ENSG00000079999	KEAP1	0.506181	4.510106	1.2E-05
1154	ENSG00000156206	C15orf26	-1.17958	-0.27537	1.21E-05
1155	ENSG00000106537	TSPAN13	-0.64706	3.185998	1.21E-05
1156	ENSG00000179172	HNRNPCL1	0.381348	3.552602	1.23E-05
1157	ENSG00000184047	DIABLO	0.493518	3.163241	1.23E-05
1158	ENSG00000181690	PLAG1	-0.67912	3.373339	1.23E-05
1159	ENSG00000130433	CACNG6	-1.60227	-1.36613	1.23E-05
1160	ENSG00000005189		0.608923	0.759706	1.24E-05
1161	ENSG00000088881	EBF4	-0.89711	0.172345	1.24E-05
1162	ENSG00000270605		0.720138	2.097931	1.25E-05
1163	ENSG00000141499	WRAP53	0.313966	3.506037	1.26E-05
1164	ENSG00000116667	C1orf21	0.696908	6.087589	1.26E-05
1165	ENSG00000139266	MARCH9	-0.48964	4.278966	1.26E-05
1166	ENSG00000116161	CACYBP	0.521824	6.120717	1.27E-05
1167	ENSG00000239961	LILRA4	-0.88597	2.091496	1.27E-05
1168	ENSG00000173890	GPR160	-0.49342	4.464212	1.28E-05
1169	ENSG00000108106	UBE2S	0.582338	4.274782	1.28E-05
1170	ENSG00000204136	GGTA1P	-1.04555	3.08075	1.29E-05
1171	ENSG00000256128	LINC00944	-0.93391	2.137073	1.29E-05
1172	ENSG00000185507	IRF7	0.80944	5.924686	1.3E-05
1173	ENSG00000163297	ANTXR2	-0.54265	5.963425	1.31E-05
1174	ENSG00000181754	AMIGO1	-0.74315	3.506832	1.34E-05
1175	ENSG00000088387	DOCK9	-0.4337	5.971597	1.35E-05
1176	ENSG00000166689	PLEKHA7	-0.76007	0.861851	1.37E-05
1177	ENSG00000071626	DAZAP1	0.272182	6.703416	1.4E-05
1178	ENSG00000169398	PTK2	-0.48269	3.690542	1.4E-05
1179	ENSG00000167785	ZNF558	-0.45104	4.495806	1.41E-05
1180	ENSG00000112130	RNF8	0.369853	3.798195	1.41E-05
1181	ENSG00000216285		0.419143	5.200907	1.42E-05
1182	ENSG00000112902	SEMA5A	-1.3125	0.133617	1.42E-05
1183	ENSG00000129270	MMP28	-1.12152	-0.08357	1.45E-05
1184	ENSG00000166770	ZNF667-AS1	-0.87797	0.782312	1.46E-05
1185	ENSG00000233006		-1.04647	-0.06547	1.47E-05

1186	ENSG00000158769	F11R	-0.48894	4.442423	1.48E-05
1187	ENSG00000132967	HMGB1P5	0.693389	4.098162	1.48E-05
1188	ENSG00000151718	WWC2	-0.64811	2.347556	1.49E-05
1189	ENSG00000211677	IGLC2	1.273415	7.234707	1.49E-05
1190	ENSG00000260032	LINC00657	-0.22231	7.993747	1.49E-05
1191	ENSG00000161298	ZNF382	-0.81088	1.459165	1.5E-05
1192	ENSG00000211936	IGHV4-4	1.525475	1.866292	1.5E-05
1193	ENSG00000166228	PCBD1	0.525476	2.974029	1.51E-05
1194	ENSG00000254986	DPP3	0.495261	4.302556	1.51E-05
1195	ENSG00000137628	DDX60	0.846981	6.361831	1.52E-05
1196	ENSG00000172031	EPHX4	-1.19162	-0.5816	1.56E-05
1197	ENSG00000082515	MRPL22	0.433153	4.213589	1.57E-05
1198	ENSG00000122786	CALD1	-1.16836	0.385914	1.58E-05
1199	ENSG00000159445	THEM4	-0.59345	4.78843	1.58E-05
1200	ENSG00000268518		-0.59585	0.070118	1.58E-05
1201	ENSG00000136193	SCRN1	-0.5732	5.213805	1.58E-05
1202	ENSG00000170627	GTSF1	0.708868	1.603256	1.59E-05
1203	ENSG00000239951	IGKV3-20	1.294007	4.801681	1.6E-05
1204	ENSG00000126368	NR1D1	-0.75677	3.123573	1.6E-05
1205	ENSG00000097096	SYDE2	-0.82623	0.776027	1.6E-05
1206	ENSG00000106803	SEC61B	0.34224	6.0459	1.6E-05
1207	ENSG00000064393	HIPK2	-0.55235	6.282917	1.61E-05
1208	ENSG00000185418	TARSL2	-0.3091	4.278809	1.62E-05
1209	ENSG00000108797	CNTNAP1	-0.7454	1.605428	1.62E-05
1210	ENSG00000119771	KLHL29	-0.62669	1.046474	1.62E-05
1211	ENSG00000173113	TRMT112	0.309979	5.884252	1.63E-05
1212	ENSG00000228878		-0.80651	0.669401	1.63E-05
1213	ENSG00000142156	COL6A1	-1.04856	1.032115	1.63E-05
1214	ENSG00000084623	EIF3I	0.262541	6.881857	1.65E-05
1215	ENSG00000152766	ANKRD22	1.66036	2.523409	1.66E-05
1216	ENSG00000165659	DACH1	-0.85832	-0.37693	1.67E-05
1217	ENSG00000163701	IL17RE	-1.03152	-0.94145	1.67E-05
1218	ENSG00000158050	DUSP2	0.735916	8.870467	1.72E-05
1219	ENSG00000064102	ASUN	0.42687	4.373594	1.72E-05
1220	ENSG00000241468	ATP5J2	0.37413	5.54886	1.72E-05
1221	ENSG00000186918	ZNF395	-0.5462	5.219211	1.72E-05
1222	ENSG00000129968	ABHD17A	0.523599	7.023694	1.76E-05
1223	ENSG00000164953	TMEM67	-0.52091	1.641113	1.76E-05
1224	ENSG00000164114	MAP9	-0.57444	3.322267	1.76E-05
1225	ENSG00000203780	FANK1	-1.07163	-1.196	1.76E-05
1226	ENSG00000132423	COQ3	0.517955	1.529972	1.78E-05
1227	ENSG00000162437	RAVER2	-0.56833	3.033213	1.78E-05
1228	ENSG00000154582	TCEB1	0.365109	5.12753	1.79E-05
1229	ENSG00000211592	IGKC	1.275066	4.376898	1.8E-05

1230	ENSG00000198046	ZNF667	-1.04973	-0.20949	1.8E-05
1231	ENSG00000198056	PRIM1	0.6477	3.388584	1.81E-05
1232	ENSG00000125871	MGME1	0.383595	4.150969	1.81E-05
1233	ENSG00000137070	IL11RA	-0.54303	4.940033	1.81E-05
1234	ENSG00000170624	SGCD	-1.4847	0.279898	1.83E-05
1235	ENSG00000109943	CRTAM	0.816898	4.907373	1.83E-05
1236	ENSG00000213928	IRF9	0.491158	4.907668	1.83E-05
1237	ENSG00000163541	SUCLG1	0.262401	5.878649	1.84E-05
1238	ENSG00000160932	LY6E	0.973174	7.513852	1.86E-05
1239	ENSG00000163565	IFI16	0.61781	8.293075	1.88E-05
1240	ENSG00000153561	RMND5A	0.350498	6.971068	1.9E-05
1241	ENSG00000245904		-0.68262	1.256397	1.94E-05
1242	ENSG00000166963	MAP1A	-0.82628	1.861718	1.94E-05
1243	ENSG00000205045	SLFN12L	0.536065	5.938442	1.95E-05
1244	ENSG00000142583	SLC2A5	1.034704	1.338632	1.95E-05
1245	ENSG00000173465	SSSCA1	0.365213	3.442591	1.96E-05
1246	ENSG00000127328	RAB3IP	-0.34154	3.856632	1.98E-05
1247	ENSG00000182742	HOXB4	0.696237	0.994517	2E-05
1248	ENSG00000111057	KRT18	-0.818	-0.24811	2E-05
1249	ENSG00000249096		0.851834	-0.64805	2.01E-05
1250	ENSG00000091164	TXNL1	0.283277	5.673471	2.02E-05
1251	ENSG00000182010	RTKN2	-0.75785	3.59806	2.02E-05
1252	ENSG00000230910		-1.89188	-0.77048	2.02E-05
1253	ENSG00000198908	BHLHB9	-0.53647	0.890271	2.04E-05
1254	ENSG00000146063	TRIM41	0.271529	5.073646	2.05E-05
1255	ENSG00000214544	GTF2IRD2P1	-0.47233	2.584828	2.05E-05
1256	ENSG00000261770		-1.09331	-1.24079	2.05E-05
1257	ENSG00000154240	CEP112	-0.79658	-0.57999	2.06E-05
1258	ENSG00000229230	MT1P3	1.398988	-0.34614	2.08E-05
1259	ENSG00000250654		0.725441	0.137815	2.08E-05
1260	ENSG00000251247	ZNF345	-0.42929	3.153606	2.08E-05
1261	ENSG00000213300	HNRNPA3P6	0.421732	2.13425	2.08E-05
1262	ENSG00000205100	HSP90AA4P	0.595633	3.915109	2.1E-05
1263	ENSG00000186049	KRT73	-1.37421	1.1483	2.11E-05
1264	ENSG00000189007	ADAT2	0.473938	4.576077	2.13E-05
1265	ENSG00000156042	TTC18	-0.76998	0.775948	2.13E-05
1266	ENSG00000230373	GOLGA6L5P	-0.68658	1.428821	2.16E-05
1267	ENSG00000235555	SUMO1P4	0.411482	1.760629	2.18E-05
1268	ENSG00000069020	MAST4	-0.57538	3.840165	2.19E-05
1269	ENSG00000092096	SLC22A17	-0.92534	1.000281	2.2E-05
1270	ENSG00000125726	CD70	0.902538	1.188288	2.2E-05
1271	ENSG00000236935		0.778413	0.318309	2.21E-05
1272	ENSG00000134326	CMPK2	1.21502	5.051621	2.22E-05
1273	ENSG00000143869	GDF7	-1.26581	-1.12341	2.22E-05

1274	ENSG00000211669	IGLV3-10	1.946679	0.283469	2.23E-05
1275	ENSG00000065675	PRKCQ	0.405862	6.371278	2.23E-05
1276	ENSG00000100453	GZMB	0.74381	7.748282	2.26E-05
1277	ENSG00000141161	UNC45B	0.836613	0.189118	2.26E-05
1278	ENSG00000217241	CBX3P9	0.408875	3.149247	2.26E-05
1279	ENSG00000147684	NDUFB9	0.347438	6.15527	2.29E-05
1280	ENSG00000204899	MZT1	0.3566	4.870703	2.3E-05
1281	ENSG00000063046	EIF4B	-0.30458	9.784793	2.3E-05
1282	ENSG00000100522	GNPNAT1	0.378125	3.697924	2.3E-05
1283	ENSG00000134809	TIMM10	0.438894	3.705223	2.3E-05
1284	ENSG00000196263	ZNF471	-1.26431	-0.75474	2.31E-05
1285	ENSG00000229107	ABHD17AP4	0.584745	2.735846	2.32E-05
1286	ENSG00000126934	MAP2K2	0.412289	6.090349	2.32E-05
1287	ENSG00000141759	TXNL4A	0.267356	5.283632	2.34E-05
1288	ENSG00000120675	DNAJC15	0.348959	5.332628	2.34E-05
1289	ENSG00000169992	NLGN2	-0.73483	1.440291	2.35E-05
1290	ENSG00000175416	CLTB	0.360883	5.183783	2.35E-05
1291	ENSG00000069188	SDK2	-1.14971	1.593063	2.36E-05
1292	ENSG00000169507	SLC38A11	-1.63751	-0.35649	2.36E-05
1293	ENSG00000254056	IGHV3-71	1.459669	-1.55319	2.37E-05
1294	ENSG00000183960	KCNH8	-1.14597	1.07486	2.37E-05
1295	ENSG00000140365	COMMD4	0.390139	4.247543	2.38E-05
1296	ENSG00000167261	DPEP2	-0.62139	5.46641	2.39E-05
1297	ENSG00000148158	SNX30	-0.52536	5.840693	2.42E-05
1298	ENSG00000136404	TM6SF1	-0.61496	3.156994	2.44E-05
1299	ENSG00000137941	TTLL7	-0.86674	0.488037	2.45E-05
1300	ENSG00000170485	NPAS2	-0.80961	0.639802	2.46E-05
1301	ENSG00000067057	PFKP	0.508212	5.39813	2.47E-05
1302	ENSG00000256262	USP30-AS1	0.688656	1.452067	2.5E-05
1303	ENSG00000169855	ROBO1	-1.01188	-0.07927	2.51E-05
1304	ENSG00000099219	ERMP1	0.464427	4.815305	2.54E-05
1305	ENSG00000101096	NFATC2	0.588857	6.773889	2.55E-05
1306	ENSG00000227372	TP73-AS1	-0.50094	4.108089	2.56E-05
1307	ENSG00000260077		-0.65313	1.752641	2.56E-05
1308	ENSG00000143977	SNRPG	0.41468	5.436846	2.56E-05
1309	ENSG00000228223	HCG11	-0.48704	3.408495	2.61E-05
1310	ENSG00000204677	FAM153C	-1.16565	0.976983	2.62E-05
1311	ENSG00000178445	GLDC	1.284832	0.866694	2.63E-05
1312	ENSG00000211653	IGLV1-40	1.395199	3.108765	2.63E-05
1313	ENSG00000119878	CRIPT	0.428131	3.579468	2.63E-05
1314	ENSG00000186056	MATN1-AS1	-0.59054	2.299016	2.63E-05
1315	ENSG00000105662	CRTC1	-0.5071	3.677494	2.7E-05
1316	ENSG00000168894	RNF181	0.394967	5.09901	2.73E-05
1317	ENSG00000131174	COX7B	0.387237	6.235317	2.73E-05

1318	ENSG00000154269	ENPP3	-0.95753	1.022032	2.76E-05
1319	ENSG00000173626	TRAPPC3L	-1.29359	-1.08927	2.76E-05
1320	ENSG00000169570	DTWD2	-0.46889	1.94062	2.77E-05
1321	ENSG00000151458	ANKRD50	-0.74482	3.387001	2.81E-05
1322	ENSG00000169891	REPS2	-0.72307	2.744705	2.84E-05
1323	ENSG00000273247		-0.43631	1.988816	2.89E-05
1324	ENSG00000186469	GNG2	0.453114	8.579912	2.89E-05
1325	ENSG00000169583	CLIC3	0.880601	3.069016	2.95E-05
1326	ENSG00000149679	CABLES2	0.392404	3.321038	2.95E-05
1327	ENSG00000168283	BMI1	-0.31462	5.818118	2.96E-05
1328	ENSG00000196381	ZNF781	-0.89151	0.385214	2.96E-05
1329	ENSG00000176340	COX8A	0.400462	6.166921	2.96E-05
1330	ENSG00000100129	EIF3L	-0.39071	8.921733	2.97E-05
1331	ENSG00000106245	BUD31	0.345749	4.975538	2.97E-05
1332	ENSG00000115289	PCGF1	0.286063	3.651285	2.97E-05
1333	ENSG00000235532	LINC00402	-1.01247	3.798892	2.97E-05
1334	ENSG00000123933	MXD4	0.528746	7.006457	2.99E-05
1335	ENSG00000159873	CCDC117	0.415318	5.286097	2.99E-05
1336	ENSG00000123737	EXOSC9	0.378254	5.94416	2.99E-05
1337	ENSG00000242766	IGKV1D-17	1.289532	1.559722	3.01E-05
1338	ENSG00000120162	MOB3B	-0.58892	2.489866	3.01E-05
1339	ENSG00000010810	FYN	0.505036	8.62571	3.02E-05
1340	ENSG00000185745	IFIT1	1.68341	5.006349	3.03E-05
1341	ENSG00000151062	CACNA2D4	-0.53872	3.324319	3.04E-05
1342	ENSG00000082293	COL19A1	-1.07735	1.384975	3.04E-05
1343	ENSG00000130816	DNMT1	0.372546	6.388973	3.07E-05
1344	ENSG00000147457	CHMP7	-0.40415	6.35912	3.07E-05
1345	ENSG00000174547	MRPL11	0.380772	4.702292	3.1E-05
1346	ENSG00000123609	NMI	0.589651	5.838895	3.1E-05
1347	ENSG00000142655	PEX14	0.367899	3.443433	3.11E-05
1348	ENSG00000170275	CRTAP	-0.54553	6.572477	3.18E-05
1349	ENSG00000271130	IGHV3OR16-8	1.334525	-0.41219	3.19E-05
1350	ENSG00000213430	HSPD1P1	0.571537	2.282693	3.21E-05
1351	ENSG00000132530	XAF1	1.077495	7.373763	3.22E-05
1352	ENSG00000184515	BEX5	-0.93838	0.886169	3.29E-05
1353	ENSG00000135951	TSGA10	-0.58571	1.513345	3.31E-05
1354	ENSG00000162946	DISC1	-0.65021	3.599429	3.33E-05
1355	ENSG00000173406	DAB1	-1.01973	-1.08094	3.33E-05
1356	ENSG00000179958	DCTPP1	0.447828	3.575973	3.33E-05
1357	ENSG00000176641	RNF152	1.521223	1.159975	3.34E-05
1358	ENSG00000211642	IGLV10-54	1.940722	0.418598	3.4E-05
1359	ENSG00000138496	PARP9	0.797334	7.248772	3.43E-05
1360	ENSG00000077721	UBE2A	0.321538	6.26198	3.44E-05
1361	ENSG00000087302	C14orf166	0.293389	6.747989	3.46E-05

1362	ENSG00000204219	TCEA3	-0.86613	2.041485	3.46E-05
1363	ENSG00000167118	URM1	0.32049	5.374787	3.48E-05
1364	ENSG00000223648	IGHV3-64	1.515281	-0.15554	3.48E-05
1365	ENSG00000077264	PAK3	-1.31926	-1.87852	3.48E-05
1366	ENSG00000168002	POLR2G	0.261819	5.99533	3.49E-05
1367	ENSG00000109684	CLNK	-1.01365	-0.82849	3.54E-05
1368	ENSG00000074266	EED	0.383806	5.287086	3.55E-05
1369	ENSG00000059378	PARP12	0.506932	6.685478	3.57E-05
1370	ENSG00000241294	IGKV2-24	1.311172	1.459552	3.58E-05
1371	ENSG00000089127	OAS1	1.099181	6.289436	3.59E-05
1372	ENSG00000122507	BBS9	-0.40183	3.737852	3.62E-05
1373	ENSG00000101162	TUBB1	-1.06353	5.992291	3.63E-05
1374	ENSG00000174442	ZWILCH	0.508185	4.07562	3.63E-05
1375	ENSG00000115085	ZAP70	0.352568	7.913391	3.63E-05
1376	ENSG00000164142	FAM160A1	-1.06017	-0.80836	3.64E-05
1377	ENSG00000135446	CDK4	0.452237	3.888154	3.68E-05
1378	ENSG00000171960	PPIH	0.286276	4.482721	3.68E-05
1379	ENSG00000112874	NUDT12	-1.16848	0.123691	3.69E-05
1380	ENSG00000119203	CPSF3	0.295293	4.898093	3.7E-05
1381	ENSG00000182502	ABHD17AP5	0.593378	2.667923	3.71E-05
1382	ENSG00000215414	PSMA6P1	0.51776	3.103786	3.75E-05
1383	ENSG00000143256	PFDN2	0.402897	4.525855	3.75E-05
1384	ENSG00000172336	POP7	0.383884	3.064825	3.75E-05
1385	ENSG00000165322	ARHGAP12	-0.43873	5.580411	3.78E-05
1386	ENSG00000155363	MOV10	0.409366	5.802245	3.81E-05
1387	ENSG00000127922	SHFM1	0.423535	5.509161	3.83E-05
1388	ENSG00000185088	RPS27L	0.386032	5.618952	3.83E-05
1389	ENSG00000185630	PBX1	-0.90206	1.037852	3.89E-05
1390	ENSG00000183662	FAM19A1	-0.9989	0.063391	3.89E-05
1391	ENSG00000042980	ADAM28	-0.72977	4.9632	3.89E-05
1392	ENSG00000119917	IFIT3	1.46657	6.261579	3.91E-05
1393	ENSG00000137824	RMDN3	0.218036	4.869096	3.93E-05
1394	ENSG00000247982	LINC00926	-0.79754	5.173888	3.96E-05
1395	ENSG00000156253	RWDD2B	0.607435	2.839814	3.98E-05
1396	ENSG00000184260	HIST2H2AC	0.789169	2.21281	3.98E-05
1397	ENSG00000260822		-0.42078	4.961791	3.98E-05
1398	ENSG00000188322	SBK1	0.583423	4.766487	4E-05
1399	ENSG00000257851	HNRNPA3P10	0.456658	0.863154	4.02E-05
1400	ENSG00000173852	DPY19L1	-0.37564	4.655095	4.02E-05
1401	ENSG00000113658	SMAD5	-0.40684	5.046972	4.02E-05
1402	ENSG00000270189		-0.65065	2.286878	4.02E-05
1403	ENSG00000163754	GYG1	0.376803	5.88025	4.05E-05
1404	ENSG00000123178	SPRYD7	-0.51093	1.9805	4.06E-05
1405	ENSG00000187824	TMEM220	-0.61414	2.177085	4.13E-05

1406	ENSG00000223478		0.594083	1.535197	4.17E-05
1407	ENSG00000101132	PFDN4	0.53324	3.073833	4.18E-05
1408	ENSG00000171791	BCL2	-0.48631	6.572884	4.18E-05
1409	ENSG00000179348	GATA2	-0.91229	2.985689	4.19E-05
1410	ENSG00000196407	THEM5	-0.76362	0.740393	4.19E-05
1411	ENSG00000205281	GOLGA6L10	-0.85931	-0.64639	4.25E-05
1412	ENSG00000146215	CRIP3	-0.97559	0.214878	4.32E-05
1413	ENSG00000159348	CYB5R1	-0.33288	4.475508	4.35E-05
1414	ENSG00000110074	FOXRED1	0.334223	3.7758	4.35E-05
1415	ENSG00000211937	IGHV2-5	1.475662	1.141313	4.36E-05
1416	ENSG00000108953	YWHAE	0.415054	7.016811	4.37E-05
1417	ENSG00000183048	SLC25A10	0.89024	-0.84429	4.39E-05
1418	ENSG00000104921	FCER2	-0.78902	3.344159	4.39E-05
1419	ENSG00000132740	IGHMBP2	0.314632	4.050299	4.39E-05
1420	ENSG00000170486	KRT72	-1.42071	2.319271	4.4E-05
1421	ENSG00000176428	VPS37D	1.047731	-1.0928	4.4E-05
1422	ENSG00000164405	UQCRQ	0.40416	5.315947	4.42E-05
1423	ENSG00000169217	CD2BP2	0.274326	6.342901	4.46E-05
1424	ENSG00000185697	MYBL1	-0.6817	6.573165	4.46E-05
1425	ENSG00000176619	LMNB2	0.45404	4.409339	4.47E-05
1426	ENSG00000271885		-0.25704	5.622147	4.47E-05
1427	ENSG00000158850	B4GALT3	0.324148	5.597903	4.49E-05
1428	ENSG00000123739	PLA2G12A	-0.46683	4.051532	4.49E-05
1429	ENSG00000063241	ISOC2	0.600556	2.880406	4.49E-05
1430	ENSG00000248445		-0.58662	0.444484	4.53E-05
1431	ENSG00000197183	C20orf112	-0.45499	5.963634	4.56E-05
1432	ENSG00000008018	PSMB1	0.266924	7.199245	4.58E-05
1433	ENSG00000176101	SSNA1	0.37978	5.086251	4.58E-05
1434	ENSG00000196155	PLEKHG4	-0.55034	3.147021	4.58E-05
1435	ENSG00000240891	PLCXD2	0.776754	3.686139	4.6E-05
1436	ENSG00000055332	EIF2AK2	0.694957	7.085415	4.6E-05
1437	ENSG00000112394	SLC16A10	-1.09585	2.11179	4.6E-05
1438	ENSG00000112414	GPR126	-1.30945	-1.15194	4.6E-05
1439	ENSG00000269290		-0.70376	1.107661	4.6E-05
1440	ENSG00000248334	WHAMMP2	-0.50642	3.546335	4.61E-05
1441	ENSG00000154760	SLFN13	0.442739	6.409858	4.62E-05
1442	ENSG00000140416	TPM1	-0.53619	3.304564	4.62E-05
1443	ENSG00000157212	PAXIP1	0.315453	4.130017	4.63E-05
1444	ENSG00000188559	RALGAPA2	-0.34235	5.337221	4.64E-05
1445	ENSG00000231259		0.276985	4.376897	4.65E-05
1446	ENSG00000160949	TONSL	0.585724	2.042949	4.68E-05
1447	ENSG00000224208		0.496621	1.173939	4.68E-05
1448	ENSG00000270015		-0.4716	3.287014	4.68E-05
1449	ENSG00000235896	IGKV3D-7	1.153181	1.353671	4.7E-05

1450	ENSG00000171659	GPR34	-0.93009	1.0343	4.71E-05
1451	ENSG00000211679	IGLC3	1.167846	7.2769	4.73E-05
1452	ENSG00000131844	MCCC2	0.274999	5.146617	4.73E-05
1453	ENSG00000255240		-0.85776	0.470372	4.73E-05
1454	ENSG00000141562	NARF	0.334648	5.981269	4.74E-05
1455	ENSG00000107679	PLEKHA1	-0.38125	6.130824	4.74E-05
1456	ENSG00000162777	DENND2D	0.597266	6.544606	4.75E-05
1457	ENSG00000259976		-0.5987	3.491854	4.75E-05
1458	ENSG00000148019	CEP78	0.556388	6.653636	4.77E-05
1459	ENSG00000243480	AMY2A	-0.57171	1.683492	4.77E-05
1460	ENSG00000133985	TTC9	-0.87949	3.783545	4.79E-05
1461	ENSG00000171444	MCC	-0.58004	2.393127	4.82E-05
1462	ENSG00000254395	IGHV4-55	1.463796	-1.24425	4.84E-05
1463	ENSG00000204220	PFDN6	0.38914	3.708111	4.85E-05
1464	ENSG00000166398	KIAA0355	-0.45278	4.995538	4.86E-05
1465	ENSG00000141458	NPC1	0.469494	5.876634	4.87E-05
1466	ENSG00000155085	AK9	-0.46934	2.861675	4.87E-05
1467	ENSG00000124920	MYRF	0.856157	2.624274	4.87E-05
1468	ENSG00000065833	ME1	-0.81934	0.761345	4.92E-05
1469	ENSG00000214026	MRPL23	0.437387	4.547869	4.93E-05
1470	ENSG00000214826	DDX12P	0.594785	2.450336	4.94E-05
1471	ENSG00000143553	SNAPIN	0.432907	4.344196	4.94E-05
1472	ENSG00000266184		0.502431	0.251846	4.98E-05
1473	ENSG00000149929	HIRIP3	0.443221	3.34159	4.99E-05
1474	ENSG00000163378	EOGT	0.352234	4.306177	5.02E-05
1475	ENSG00000242265	PEG10	-0.86805	1.352352	5.02E-05
1476	ENSG00000164305	CASP3	0.750533	6.277056	5.02E-05
1477	ENSG00000182389	CACNB4	-1.00629	0.8759	5.1E-05
1478	ENSG00000140479	PCSK6	-1.09609	1.333623	5.1E-05
1479	ENSG00000160746	ANO10	-0.53677	3.245117	5.12E-05
1480	ENSG00000154358	OBSCN	-0.76206	4.655105	5.12E-05
1481	ENSG00000167130	DOLPP1	0.372487	2.718973	5.13E-05
1482	ENSG00000181585	TMIE	-1.2111	-1.40053	5.13E-05
1483	ENSG00000254231		-0.75194	-0.7959	5.16E-05
1484	ENSG00000211976	IGHV3-73	1.554695	-0.30847	5.18E-05
1485	ENSG00000129595	EPB41L4A	-0.75247	1.878595	5.22E-05
1486	ENSG00000158482	SNX29P1	-0.66057	0.938637	5.27E-05
1487	ENSG00000196684	HSH2D	0.468036	6.764801	5.27E-05
1488	ENSG00000185567	AHNAK2	-0.85465	0.657402	5.32E-05
1489	ENSG00000115539	PDCL3	0.408005	5.104107	5.35E-05
1490	ENSG00000273230		-0.77423	-0.51901	5.4E-05
1491	ENSG00000116191	RALGPS2	-0.7127	5.90613	5.4E-05
1492	ENSG00000143149	ALDH9A1	0.298418	5.890779	5.42E-05
1493	ENSG00000089012	SIRPG	0.847481	4.419438	5.43E-05

1494	ENSG00000134765	DSC1	-1.6926	-0.19268	5.45E-05
1495	ENSG00000074695	LMAN1	0.319853	6.759065	5.48E-05
1496	ENSG00000155966	AFF2	-0.79733	-0.11882	5.5E-05
1497	ENSG00000126016	AMOT	-0.57127	1.515049	5.52E-05
1498	ENSG00000211767	TRBJ2-3	0.64111	1.273029	5.56E-05
1499	ENSG00000232119	MCTS1	0.423533	4.527083	5.56E-05
1500	ENSG00000186810	CXCR3	1.125506	4.150468	5.57E-05
1501	ENSG00000115317	HTRA2	0.307199	3.73522	5.58E-05
1502	ENSG00000002834	LASP1	0.453979	7.686543	5.61E-05
1503	ENSG00000247077	PGAM5	0.323601	4.377975	5.65E-05
1504	ENSG00000260948		-0.75838	0.230783	5.65E-05
1505	ENSG00000215006	CHCHD2P2	0.486181	1.938474	5.68E-05
1506	ENSG00000246214		-0.86605	-0.5711	5.69E-05
1507	ENSG00000152527	PLEKHH2	-0.99493	-0.81292	5.69E-05
1508	ENSG00000225886		1.22073	-0.16027	5.72E-05
1509	ENSG00000131475	VPS25	0.353537	4.839241	5.73E-05
1510	ENSG00000115241	PPM1G	0.368676	6.330885	5.75E-05
1511	ENSG00000138795	LEF1	-0.59437	6.895154	5.77E-05
1512	ENSG00000125870	SNRPB2	0.341841	6.247797	5.78E-05
1513	ENSG00000231113		-0.44528	2.093258	5.8E-05
1514	ENSG00000145908	ZNF300	-0.57749	2.299524	5.8E-05
1515	ENSG00000130956	HABP4	-0.58196	3.46743	5.8E-05
1516	ENSG00000169021	UQCRFS1	0.283684	6.561951	5.84E-05
1517	ENSG00000182107	TMEM30B	-0.6559	1.689503	5.84E-05
1518	ENSG00000225936		-1.10068	0.120066	5.85E-05
1519	ENSG00000173530	TNFRSF10D	-0.66913	3.492768	5.86E-05
1520	ENSG00000237763	AMY1A	-0.62109	0.61105	5.87E-05
1521	ENSG00000055118	KCNH2	-0.89351	0.030839	5.89E-05
1522	ENSG00000154640	BTG3	0.666314	5.2509	5.91E-05
1523	ENSG00000183978	COA3	0.567674	3.654399	5.91E-05
1524	ENSG00000075914	EXOSC7	0.243954	4.435017	5.91E-05
1525	ENSG00000125445	MRPS7	0.2617	4.896622	5.94E-05
1526	ENSG00000243811	APOBEC3D	0.552007	3.95734	5.95E-05
1527	ENSG00000081692	JMJD4	0.457951	1.955501	5.95E-05
1528	ENSG00000272316		-0.53039	2.092856	5.96E-05
1529	ENSG00000068831	RASGRP2	-0.31004	7.945133	5.97E-05
1530	ENSG00000113924	HGD	-0.73092	1.295681	6E-05
1531	ENSG00000198860	TSEN15	0.279949	5.186447	6.01E-05
1532	ENSG00000115364	MRPL19	0.312383	4.606892	6.03E-05
1533	ENSG00000213228	RPL12P38	0.443631	2.198581	6.04E-05
1534	ENSG00000126267	COX6B1	0.354047	6.644444	6.04E-05
1535	ENSG00000183049	CAMK1D	-0.37754	5.712305	6.05E-05
1536	ENSG00000185668	POU3F1	1.332757	-1.59788	6.08E-05
1537	ENSG00000170473	WIBG	0.307759	4.17789	6.14E-05

1538	ENSG00000092208	GEMIN2	0.399467	2.745664	6.14E-05
1539	ENSG00000131165	CHMP1A	0.37153	6.073216	6.17E-05
1540	ENSG00000177879	AP3S1	0.270743	6.207915	6.18E-05
1541	ENSG00000240041	IGHJ4	1.222588	-1.12267	6.29E-05
1542	ENSG00000105619	TFPT	0.351831	3.240431	6.3E-05
1543	ENSG00000145979	TBC1D7	0.387663	3.333027	6.3E-05
1544	ENSG00000122122	SASH3	0.33448	7.934294	6.32E-05
1545	ENSG00000121766	ZCCHC17	0.287234	5.021661	6.32E-05
1546	ENSG00000123636	BAZ2B	-0.47663	5.740379	6.34E-05
1547	ENSG00000116478	HDAC1	0.340901	7.122241	6.36E-05
1548	ENSG00000155324	GRAMD3	0.526631	3.168808	6.39E-05
1549	ENSG00000122705	CLTA	0.363938	5.652712	6.39E-05
1550	ENSG00000072195	SPEG	-0.87976	1.333172	6.43E-05
1551	ENSG00000125148	MT2A	0.94875	5.094584	6.46E-05
1552	ENSG00000234444	ZNF736	-0.43163	5.441906	6.46E-05
1553	ENSG00000222037	IGLC6	1.32309	3.790494	6.52E-05
1554	ENSG00000102796	DHRS12	-0.37202	3.31567	6.56E-05
1555	ENSG00000149292	TTC12	-0.48683	3.631606	6.56E-05
1556	ENSG00000182866	LCK	0.439377	7.708877	6.59E-05
1557	ENSG00000175707	C1orf172	-0.9435	-1.05615	6.61E-05
1558	ENSG00000124659	TBCC	0.332199	5.448254	6.62E-05
1559	ENSG00000162840	MT2P1	1.359269	0.588738	6.63E-05
1560	ENSG00000103064	SLC7A6	-0.44182	5.480576	6.64E-05
1561	ENSG00000143156	NME7	0.398079	2.22372	6.66E-05
1562	ENSG00000163534	FCRL1	-0.95914	5.165121	6.66E-05
1563	ENSG00000165568	AKR1E2	-0.90096	-0.46199	6.72E-05
1564	ENSG00000211654	IGLV5-37	1.649811	-2.24406	6.83E-05
1565	ENSG00000134539	KLRD1	0.626466	6.945405	6.83E-05
1566	ENSG00000257093	KIAA1147	-0.48448	6.988494	6.83E-05
1567	ENSG00000156395	SORCS3	-1.43456	-1.23061	6.88E-05
1568	ENSG00000142864	SERBP1	0.336066	7.937862	6.92E-05
1569	ENSG00000128040	SPINK2	-1.16629	-0.48195	6.94E-05
1570	ENSG00000223459	FAM115B	-0.81274	1.101017	6.95E-05
1571	ENSG00000151150	ANK3	-0.67346	4.26609	7.02E-05
1572	ENSG00000157873	TNFRSF14	0.280961	7.455808	7.1E-05
1573	ENSG00000151233	GXYLT1	0.43005	5.779768	7.11E-05
1574	ENSG00000149131	SERPING1	1.604	3.586077	7.11E-05
1575	ENSG00000134489	HRH4	-0.9642	1.055903	7.16E-05
1576	ENSG00000033011	ALG1	0.367735	2.923019	7.22E-05
1577	ENSG00000137996	RTCA	0.265358	5.211944	7.22E-05
1578	ENSG00000143252	SDHC	0.282024	6.129227	7.27E-05
1579	ENSG00000183527	PSMG1	0.305962	3.527698	7.3E-05
1580	ENSG00000147123	NDUFB11	0.327606	5.670083	7.33E-05
1581	ENSG00000196922	ZNF252P	-0.36218	4.406674	7.34E-05

1582	ENSG00000237424	FOXD2-AS1	0.597367	1.569311	7.39E-05
1583	ENSG00000004142	POLDIP2	0.317814	5.576987	7.43E-05
1584	ENSG00000171962	LRRC48	-0.46881	1.687227	7.49E-05
1585	ENSG00000027869	SH2D2A	0.90399	5.574563	7.53E-05
1586	ENSG00000175216	CKAP5	0.343386	5.902428	7.53E-05
1587	ENSG00000187667	WHAMMP3	-0.43497	4.267006	7.53E-05
1588	ENSG00000240038	AMY2B	-0.52811	4.06283	7.53E-05
1589	ENSG00000177570	SAMD12	-1.01889	0.11792	7.53E-05
1590	ENSG00000198780	FAM169A	-0.76349	4.897278	7.54E-05
1591	ENSG00000141030	COPS3	0.351002	6.210004	7.55E-05
1592	ENSG00000162645	GBP2	0.478611	8.644208	7.57E-05
1593	ENSG00000196976	LAGE3	0.416772	2.789475	7.57E-05
1594	ENSG00000184445	KNTC1	0.391652	4.953793	7.61E-05
1595	ENSG00000104129	DNAJC17	0.287642	3.254134	7.61E-05
1596	ENSG00000101493	ZNF516	-0.5231	4.753205	7.67E-05
1597	ENSG00000165264	NDUFB6	0.424841	4.249892	7.68E-05
1598	ENSG00000159352	PSMD4	0.32667	6.550275	7.71E-05
1599	ENSG00000233859	ADH5P4	0.402772	2.152489	7.75E-05
1600	ENSG00000007516	BAIAP3	-0.80175	2.907601	7.76E-05
1601	ENSG00000254815		-0.82855	0.527441	7.77E-05
1602	ENSG00000054148	PHPT1	0.433904	4.172534	7.84E-05
1603	ENSG00000065057	NTHL1	0.70701	2.094503	7.86E-05
1604	ENSG00000228956		-0.50561	3.219861	7.86E-05
1605	ENSG00000170860	LSM3	0.420301	5.084712	7.9E-05
1606	ENSG00000005471	ABCB4	-0.62348	1.497175	7.92E-05
1607	ENSG00000169976	SF3B5	0.303167	6.383339	7.95E-05
1608	ENSG00000260645		-1.07173	-1.01677	7.95E-05
1609	ENSG00000264198		0.491718	7.180634	7.97E-05
1610	ENSG00000135597	REPS1	-0.20246	5.517661	7.98E-05
1611	ENSG00000075340	ADD2	-0.62826	2.691485	8E-05
1612	ENSG00000154545	MAGED4	-0.92772	-0.50413	8E-05
1613	ENSG00000119013	NDUFB3	0.522159	4.380102	8.02E-05
1614	ENSG00000031691	CENPQ	0.711671	1.917723	8.05E-05
1615	ENSG00000175324	LSM1	0.374293	4.067034	8.05E-05
1616	ENSG00000005249	PRKAR2B	-0.88181	5.379868	8.1E-05
1617	ENSG00000185272	RBM11	-0.76861	0.821367	8.15E-05
1618	ENSG00000230138		-0.95732	-0.6737	8.15E-05
1619	ENSG00000101605	MYOM1	-0.87395	1.329239	8.19E-05
1620	ENSG00000213235	EEF1A1P16	-0.29526	8.812678	8.24E-05
1621	ENSG00000161036	LRWD1	0.345584	3.673845	8.28E-05
1622	ENSG00000258501	EIF3LP1	-0.43873	3.121761	8.31E-05
1623	ENSG00000102554	KLF5	-0.76449	3.202254	8.35E-05
1624	ENSG00000115128		0.342989	5.873663	8.37E-05
1625	ENSG00000115884	SDC1	1.66878	-1.62412	8.4E-05

1626	ENSG00000121073	SLC35B1	0.278532	5.442431	8.4E-05
1627	ENSG00000005022	SLC25A5	0.293562	7.836997	8.43E-05
1628	ENSG00000115484	CCT4	0.306131	6.730279	8.44E-05
1629	ENSG00000187778	MCRS1	0.242906	5.845196	8.58E-05
1630	ENSG00000163590	PPM1L	-0.55002	4.843635	8.6E-05
1631	ENSG00000166557	TMED3	0.368759	4.996628	8.63E-05
1632	ENSG00000138166	DUSP5	0.636772	6.753689	8.66E-05
1633	ENSG00000170144	HNRNPA3	0.217905	9.142561	8.72E-05
1634	ENSG00000204455	TRIM51BP	-1.48155	-1.02522	8.74E-05
1635	ENSG00000205476	CCDC85C	-0.60902	1.157252	8.77E-05
1636	ENSG00000128708	HAT1	0.386005	5.757825	8.83E-05
1637	ENSG00000183508	FAM46C	0.76118	8.673244	8.84E-05
1638	ENSG00000228325		1.149688	1.549941	8.85E-05
1639	ENSG00000185621	LMLN	-0.4433	2.631622	8.92E-05
1640	ENSG00000212734	C17orf100	-0.70378	0.721595	8.96E-05
1641	ENSG00000166446	CDYL2	0.336405	5.044282	9.01E-05
1642	ENSG00000175873		0.701338	2.371292	9.08E-05
1643	ENSG00000154743	TSEN2	-0.39765	2.301945	9.08E-05
1644	ENSG00000068394	GPKOW	0.314313	4.370849	9.12E-05
1645	ENSG00000167085	PHB	0.361096	4.875075	9.14E-05
1646	ENSG00000144381	HSPD1	0.507039	7.574289	9.16E-05
1647	ENSG00000260912		-0.56945	0.66791	9.17E-05
1648	ENSG00000155959	VBP1	0.3424	5.487294	9.19E-05
1649	ENSG00000106624	AEBP1	-0.63671	2.105209	9.22E-05
1650	ENSG00000069712	KIAA1107	-0.69036	2.05655	9.22E-05
1651	ENSG00000133460	SLC2A11	-0.44995	2.607916	9.23E-05
1652	ENSG00000152804	HHEX	-0.68054	4.339748	9.23E-05
1653	ENSG00000068976	PYGM	-0.46552	2.100116	9.46E-05
1654	ENSG00000160191	PDE9A	-0.73469	1.54953	9.46E-05
1655	ENSG00000168890	TMEM150A	-0.38699	2.913763	9.58E-05
1656	ENSG00000185664	PMEL	-0.67449	0.208761	9.6E-05
1657	ENSG00000241566	IGKV2D-24	1.327743	0.088573	9.6E-05
1658	ENSG00000117480	FAAH	-0.48357	2.225351	9.71E-05
1659	ENSG00000073464	CLCN4	-0.71392	1.305554	9.71E-05
1660	ENSG00000143851	PTPN7	0.618566	6.42783	9.75E-05
1661	ENSG00000229164	TRAC	0.507414	8.168298	9.79E-05
1662	ENSG00000260398		-0.85037	-0.19847	9.85E-05
1663	ENSG00000271936		0.679755	-0.52071	9.86E-05
1664	ENSG00000211670	IGLV3-9	1.300295	-0.05159	9.87E-05
1665	ENSG00000004779	NDUFAB1	0.32587	4.884165	9.88E-05
1666	ENSG00000108518	PFN1	0.490583	9.515971	9.92E-05
1667	ENSG00000119421	NDUFA8	0.428853	3.860315	9.92E-05
1668	ENSG00000135686	KLHL36	-0.29972	5.870256	9.93E-05
1669	ENSG00000205542	TMSB4X	0.376056	11.01356	0.000101

1670	ENSG00000173482	PTPRM	-1.18592	3.055818	0.000102
1671	ENSG00000178498	DTX3	0.42342	4.475655	0.000102
1672	ENSG00000241351	IGKV3-11	1.203425	3.886236	0.000103
1673	ENSG00000225964		1.272007	0.932084	0.000104
1674	ENSG00000005448	WDR54	0.370387	3.501668	0.000104
1675	ENSG00000086065	CHMP5	0.49813	5.632368	0.000104
1676	ENSG00000204860	FAM201A	-1.0272	-0.98578	0.000104
1677	ENSG00000240671	IGKV1-8	1.11794	1.394554	0.000105
1678	ENSG00000125970	RALY	0.335887	7.100079	0.000107
1679	ENSG00000112144	ICK	-0.29291	4.816504	0.000107
1680	ENSG00000203668	CHML	-0.41101	5.114963	0.000107
1681	ENSG00000081014	AP4E1	0.29477	4.983718	0.000107
1682	ENSG00000175756	AURKAIP1	0.383536	5.109818	0.000107
1683	ENSG00000122121	XPNPEP2	0.71781	1.150507	0.000109
1684	ENSG00000116663	FBXO6	0.718225	4.151594	0.000109
1685	ENSG00000253818	IGLV1-41	1.550488	0.103467	0.000109
1686	ENSG00000255026		-0.87077	2.664907	0.000109
1687	ENSG00000072506	HSD17B10	0.365231	5.101841	0.00011
1688	ENSG00000101844	ATG4A	0.315747	4.341445	0.00011
1689	ENSG00000176490	DIRAS1	-1.07914	0.200858	0.00011
1690	ENSG00000185189	NRBP2	-0.41571	3.474562	0.00011
1691	ENSG00000203827	NBPF16	-0.30919	5.277971	0.00011
1692	ENSG00000223519	KIF28P	-1.16479	-1.09702	0.000111
1693	ENSG00000211965	IGHV3-49	1.46414	0.853167	0.000111
1694	ENSG00000101751	POLI	-0.54475	4.433856	0.000111
1695	ENSG00000167315	ACAA2	0.466468	5.08828	0.000112
1696	ENSG00000137288	UQCC2	0.32798	3.829818	0.000112
1697	ENSG00000211638	IGLV8-61	1.465686	1.868629	0.000112
1698	ENSG00000162739	SLAMF6	0.703162	6.697013	0.000112
1699	ENSG00000167371	PRRT2	-0.75736	-0.1822	0.000112
1700	ENSG00000134548	C12orf39	-1.18197	1.295972	0.000113
1701	ENSG00000111335	OAS2	0.970351	7.261672	0.000113
1702	ENSG00000128944	KNSTRN	0.450459	2.453103	0.000113
1703	ENSG00000136167	LCP1	0.348241	11.27515	0.000113
1704	ENSG00000162368	CMPK1	-0.21248	7.787814	0.000113
1705	ENSG00000167555	ZNF528	-0.64244	2.917334	0.000113
1706	ENSG00000188157	AGRN	0.831254	2.414032	0.000113
1707	ENSG00000020426	MNAT1	0.422299	3.270008	0.000114
1708	ENSG00000104814	MAP4K1	0.416948	6.41999	0.000117
1709	ENSG00000130396	MLLT4	-0.53138	3.48777	0.000117
1710	ENSG00000085449	WDFY1	0.426351	7.122324	0.000117
1711	ENSG00000168497	SDPR	-0.9792	6.251718	0.000117
1712	ENSG00000238000		0.671851	2.451304	0.000117
1713	ENSG00000178896	EXOSC4	0.500544	1.832561	0.000117

1714	ENSG00000182749	PAQR7	-0.62364	2.384149	0.000117
1715	ENSG00000125877	ITPA	0.356101	4.919277	0.000117
1716	ENSG00000147168	IL2RG	0.460852	8.508777	0.000118
1717	ENSG00000143365	RORC	-0.81056	2.019292	0.000119
1718	ENSG00000167770	OTUB1	0.294796	6.274064	0.00012
1719	ENSG00000198846	TOX	0.584103	4.870081	0.000121
1720	ENSG00000211593	IGKJ5	1.035574	2.981103	0.000121
1721	ENSG00000100028	SNRPD3	0.250212	5.904137	0.000121
1722	ENSG00000196648	GOLGA6L20	-0.60451	1.008645	0.000121
1723	ENSG00000127585	FBXL16	-1.02851	1.681198	0.000123
1724	ENSG00000205955	HSP90AA5P	0.569785	1.059492	0.000123
1725	ENSG00000137200	CMTR1	0.313348	6.521722	0.000124
1726	ENSG00000211893	IGHG2	1.345277	6.485146	0.000124
1727	ENSG00000137168	PPIL1	0.48027	3.556114	0.000125
1728	ENSG00000239819	IGKV1D-8	1.081931	0.673888	0.000126
1729	ENSG00000183444	OR7E38P	0.424503	1.664829	0.000126
1730	ENSG00000046647	GEMIN8	0.229661	3.254993	0.000126
1731	ENSG00000181038	METTL23	0.27878	4.742211	0.000126
1732	ENSG00000139168	ZCRB1	0.353824	5.082454	0.000127
1733	ENSG00000162521	RBBP4	0.228515	7.012727	0.000127
1734	ENSG00000100485	SOS2	-0.32965	6.539559	0.000128
1735	ENSG00000140990	NDUFB10	0.374753	5.060768	0.000129
1736	ENSG00000166147	FBN1	0.637144	-0.20122	0.00013
1737	ENSG00000226660	TRBV2	0.663191	2.425317	0.000131
1738	ENSG00000119699	TGFB3	-0.4015	1.82243	0.000131
1739	ENSG00000011105	TSPAN9	-0.94655	1.560155	0.000131
1740	ENSG00000105507	CABP5	-1.17637	0.851937	0.000131
1741	ENSG00000162373	BEND5	-0.62383	0.516353	0.000131
1742	ENSG00000154473	BUB3	0.247328	7.367828	0.000132
1743	ENSG00000197763	TXNRD3	-0.83098	-0.65254	0.000132
1744	ENSG00000203832	NBPF20	-0.28949	5.488131	0.000132
1745	ENSG00000154134	ROBO3	-0.748	2.180441	0.000133
1746	ENSG00000116668	SWT1	-0.40656	3.573955	0.000133
1747	ENSG00000087995	METTL2A	0.361881	2.952377	0.000133
1748	ENSG00000130165	ELOF1	0.295909	5.22699	0.000133
1749	ENSG00000143157	POGK	-0.23458	6.26071	0.000133
1750	ENSG00000211625	IGKV3D-20	1.208117	3.726098	0.000135
1751	ENSG00000270179		-0.69277	0.196951	0.000135
1752	ENSG00000179639	FCER1A	-1.00091	5.005076	0.000135
1753	ENSG00000096080	MRPS18A	0.383215	2.770887	0.000137
1754	ENSG00000142102	ATHL1	-0.51444	7.136877	0.000138
1755	ENSG00000070950	RAD18	0.259202	4.484995	0.000138
1756	ENSG00000229211		0.608742	-0.3978	0.000139
1757	ENSG00000166780	C16orf45	-0.52086	1.866332	0.000139

1758	ENSG00000132846	ZBED3	-0.64384	1.745273	0.000139
1759	ENSG00000107249	GLIS3	-0.98018	-0.5841	0.000139
1760	ENSG00000170779	CDCA4	0.445083	4.113892	0.00014
1761	ENSG00000153574	RPIA	0.284429	5.304501	0.00014
1762	ENSG00000251364		-0.68179	1.305571	0.00014
1763	ENSG00000171451	DSEL	-1.15598	0.638317	0.00014
1764	ENSG00000008277	ADAM22	-0.51583	2.532104	0.000141
1765	ENSG00000117016	RIMS3	-0.56596	1.889193	0.000141
1766	ENSG00000112695	COX7A2	0.328356	6.564118	0.000142
1767	ENSG00000116793	PHTF1	0.344248	4.269946	0.000142
1768	ENSG00000113734	BNIP1	0.480221	3.970546	0.000142
1769	ENSG00000110871	COQ5	0.485969	3.754999	0.000142
1770	ENSG00000267481		-0.54767	1.923226	0.000143
1771	ENSG00000170265	ZNF282	0.379547	3.715208	0.000145
1772	ENSG00000229083	PSMA6P2	0.480134	2.134856	0.000147
1773	ENSG00000233585		0.5407	0.656318	0.000147
1774	ENSG00000187994	RINL	0.459444	3.665407	0.000149
1775	ENSG00000158106	RHPN1	-0.69273	2.908651	0.000149
1776	ENSG00000171291	ZNF439	-0.54695	3.02932	0.00015
1777	ENSG00000197093	GAL3ST4	-0.69681	2.025665	0.00015
1778	ENSG00000010932	FMO1	0.554064	2.166835	0.00015
1779	ENSG00000090266	NDUFB2	0.355415	5.564328	0.000151
1780	ENSG00000081148	IMPG2	-0.59042	0.571545	0.000151
1781	ENSG00000143502	SUSD4	-0.75394	1.318989	0.000152
1782	ENSG00000102007	PLP2	-0.39915	6.189682	0.000152
1783	ENSG00000213592		0.462844	0.431117	0.000153
1784	ENSG00000115561	CHMP3	-0.24395	5.796358	0.000154
1785	ENSG00000103495	MAZ	0.379473	4.782834	0.000154
1786	ENSG00000173915	USMG5	0.371136	5.482155	0.000155
1787	ENSG00000115109	EPB41L5	-0.36875	2.852414	0.000155
1788	ENSG00000136810	TXN	0.435114	5.796734	0.000156
1789	ENSG00000111196	MAGOHB	0.350989	3.302205	0.000156
1790	ENSG00000172985	SH3RF3	-0.66739	1.10778	0.000158
1791	ENSG00000115935	WIPF1	0.352344	8.924304	0.000158
1792	ENSG00000258733		-0.4848	1.911111	0.000158
1793	ENSG00000162972	C2orf47	0.411916	3.513928	0.000159
1794	ENSG00000159403	C1R	0.568652	1.037125	0.000159
1795	ENSG00000091181	IL5RA	-0.9946	0.21344	0.000159
1796	ENSG00000180881	CAPS2	-0.62724	0.728839	0.000159
1797	ENSG00000198720	ANKRD13B	0.603749	0.246179	0.000161
1798	ENSG00000128228	SDF2L1	0.525131	3.688884	0.000161
1799	ENSG00000184983	NDUFA6	0.332307	5.810803	0.000161
1800	ENSG00000125657	TNFSF9	1.168252	2.390689	0.000162
1801	ENSG00000230055	CISD3	0.723544	3.463605	0.000162

1802	ENSG00000156345	CDK20	-0.43336	1.817326	0.000162
1803	ENSG00000180758	GPR157	-0.80179	1.934501	0.000162
1804	ENSG00000140995	DEF8	-0.43143	5.319341	0.000162
1805	ENSG00000260689	HNRNPA3P11	0.448325	1.277539	0.000162
1806	ENSG00000119537	KDSR	-0.35972	4.688511	0.000162
1807	ENSG00000171314	PGAM1	0.362125	7.578123	0.000163
1808	ENSG00000110888	CAPRIN2	-0.37138	5.327196	0.000163
1809	ENSG00000186300	ZNF555	-0.41277	2.32911	0.000163
1810	ENSG00000229931		0.684802	0.330793	0.000163
1811	ENSG00000173821	RNF213	0.458882	9.810991	0.000164
1812	ENSG00000132832		0.802816	0.753932	0.000165
1813	ENSG00000211664	IGLV2-18	1.145282	1.951891	0.000165
1814	ENSG00000085662	AKR1B1	0.303717	5.864686	0.000166
1815	ENSG00000168646	AXIN2	-0.77448	3.083061	0.000166
1816	ENSG00000140398	NEIL1	-0.62699	3.89343	0.000167
1817	ENSG00000168672	FAM84B	-0.44044	4.029985	0.000167
1818	ENSG00000126432	PRDX5	0.361176	6.088227	0.000167
1819	ENSG00000128815	WDFY4	-0.60064	5.411712	0.000168
1820	ENSG00000013275	PSMC4	0.322167	5.709633	0.000168
1821	ENSG00000258727		-0.43621	2.365132	0.000169
1822	ENSG00000224041	IGKV3D-15	1.128747	3.432707	0.000169
1823	ENSG00000186010	NDUFA13	0.334242	4.426771	0.000169
1824	ENSG00000157796	WDR19	-0.42224	4.135144	0.00017
1825	ENSG00000174744	BRMS1	0.273831	5.538049	0.00017
1826	ENSG00000186275	NBPF12	-0.24468	5.493928	0.00017
1827	ENSG00000131355	EMR3	-0.81151	3.232396	0.00017
1828	ENSG00000129116	PALLD	-0.583	2.936847	0.000171
1829	ENSG00000147601	TERF1	0.439866	5.496294	0.000171
1830	ENSG00000196954	CASP4	0.372332	7.705205	0.000171
1831	ENSG00000021300	PLEKHB1	-0.57871	3.494213	0.000171
1832	ENSG00000119280	C1orf198	-0.46453	2.367419	0.000171
1833	ENSG00000167747	C19orf48	0.440563	4.344138	0.000172
1834	ENSG00000271738		-0.77811	-0.76735	0.000173
1835	ENSG00000167600	CYP2S1	-0.94288	1.385582	0.000173
1836	ENSG00000253291	TRBV7-7	0.833513	0.050818	0.000174
1837	ENSG00000124733	MEA1	0.311391	4.889544	0.000174
1838	ENSG00000225948		-0.92004	0.329773	0.000174
1839	ENSG00000249825		-0.69314	0.408874	0.000175
1840	ENSG00000084110	HAL	-0.74131	3.097081	0.000175
1841	ENSG00000159674	SPON2	0.656529	5.724514	0.000175
1842	ENSG00000142188	TMEM50B	-0.23533	5.977881	0.000175
1843	ENSG00000073905	VDAC1P1	0.442199	1.550946	0.000176
1844	ENSG00000244701		-0.43163	2.101427	0.000178
1845	ENSG00000233137		-0.30675	6.981976	0.00018

1846	ENSG00000254876		-0.67277	0.707895	0.00018
1847	ENSG00000211632	IGKV3D-11	1.082228	2.586537	0.000181
1848	ENSG00000169245	CXCL10	1.742993	3.724422	0.000181
1849	ENSG00000262580		-0.63576	3.147307	0.000181
1850	ENSG00000007264	MATK	0.526427	5.521584	0.000182
1851	ENSG00000047634	SCML1	-0.77487	4.814133	0.000182
1852	ENSG00000187243	MAGED4B	-0.8793	-0.48079	0.000182
1853	ENSG00000239264	TXNDC5	0.738378	1.570943	0.000182
1854	ENSG00000166341	DCHS1	-0.83418	1.91371	0.000182
1855	ENSG00000232021	LEF1-AS1	-0.85993	0.730125	0.000182
1856	ENSG00000253998	IGKV2-29	1.324553	1.112143	0.000183
1857	ENSG00000137310	TCF19	0.977778	-1.32826	0.000183
1858	ENSG00000149150	SLC43A1	0.422346	2.844854	0.000183
1859	ENSG00000109536	FRG1	0.304577	5.310759	0.000184
1860	ENSG00000116157	GPX7	0.397359	3.38797	0.000184
1861	ENSG00000127831	VIL1	-1.00621	-0.01204	0.000184
1862	ENSG00000196405	EVL	0.280295	8.638509	0.000185
1863	ENSG00000261355		-0.68228	3.769211	0.000185
1864	ENSG00000184500	PROS1	-1.09836	0.720813	0.000185
1865	ENSG00000163812	ZDHHC3	0.194728	6.129894	0.000185
1866	ENSG00000100387	RBX1	0.369297	5.785558	0.000185
1867	ENSG00000146674	IGFBP3	-0.8229	2.649861	0.000186
1868	ENSG00000154978	VOPP1	0.311123	7.30112	0.000187
1869	ENSG00000182552	RWDD4	0.278149	4.885557	0.000188
1870	ENSG00000154723	ATP5J	0.332375	5.568899	0.000189
1871	ENSG00000132603	NIP7	0.300331	5.236628	0.00019
1872	ENSG00000266714	MYO15B	-0.56202	6.307754	0.00019
1873	ENSG00000214900	C14orf182	-0.50277	2.903596	0.000191
1874	ENSG00000101193	GID8	0.229261	5.564124	0.000191
1875	ENSG00000211655	IGLV1-36	1.267578	0.69639	0.000191
1876	ENSG00000166136	NDUFB8	0.253839	5.069769	0.000191
1877	ENSG00000272452		-0.48921	1.008004	0.000191
1878	ENSG00000116649	SRM	0.502025	4.328524	0.000192
1879	ENSG00000074219	TEAD2	-0.97608	-1.01394	0.000192
1880	ENSG00000049246	PER3	-0.39188	2.761563	0.000193
1881	ENSG00000110042	DTX4	-0.51955	3.243708	0.000193
1882	ENSG00000259032	ENSAP2	0.303188	3.259236	0.000194
1883	ENSG00000114923	SLC4A3	-1.26599	-0.63958	0.000195
1884	ENSG00000104835	SARS2	0.433469	1.42674	0.000195
1885	ENSG00000123131	PRDX4	0.455224	3.952932	0.000198
1886	ENSG00000174516	PELI3	-0.63889	2.251058	0.000198
1887	ENSG00000179750	APOBEC3B	0.979453	4.386706	0.0002
1888	ENSG00000173548	SNX33	-0.53159	2.227717	0.0002
1889	ENSG00000211967	IGHV3-53	1.336269	0.256959	0.0002

1890	ENSG00000111110	PPM1H	-0.8978	0.780385	0.000201
1891	ENSG00000161513	FDXR	0.660765	2.159863	0.000203
1892	ENSG00000103510	KAT8	-0.21807	5.390532	0.000203
1893	ENSG00000099804	CDC34	0.442825	4.96477	0.000204
1894	ENSG00000078668	VDAC3	0.26246	6.53458	0.000204
1895	ENSG00000155099	TMEM55A	-0.64647	3.547681	0.000204
1896	ENSG00000181744	C3orf58	0.4075	5.368286	0.000204
1897	ENSG00000232216	IGHV3-43	1.380526	-0.31238	0.000204
1898	ENSG00000149418	ST14	-0.83988	2.673304	0.000206
1899	ENSG00000213585	VDAC1	0.401044	6.644137	0.000207
1900	ENSG00000131016	AKAP12	-0.81696	2.27469	0.000207
1901	ENSG00000171680	PLEKHG5	-0.96933	0.335516	0.000207
1902	ENSG00000101230	ISM1	-1.19902	-0.74689	0.000209
1903	ENSG00000187193	MT1X	0.873926	2.620057	0.000209
1904	ENSG00000122644	ARL4A	-0.67116	5.437577	0.00021
1905	ENSG00000128266	GNAZ	-0.76969	2.294235	0.000213
1906	ENSG00000211794	TRAV12-3	0.641908	1.638781	0.000214
1907	ENSG00000140391	TSPAN3	-0.27393	5.584524	0.000215
1908	ENSG00000165804	ZNF219	-0.73414	0.641003	0.000216
1909	ENSG00000135519	KCNH3	-0.65024	1.747078	0.000216
1910	ENSG00000105289	TJP3	-0.64141	1.179144	0.000217
1911	ENSG00000197385	ZNF860	-0.75411	0.434264	0.000217
1912	ENSG00000108826	MRPL27	0.412746	4.360369	0.000218
1913	ENSG00000167515	TRAPPC2L	0.253971	4.803043	0.000218
1914	ENSG00000198832		-0.58141	2.555549	0.000218
1915	ENSG00000273033		-0.46959	4.052689	0.000219
1916	ENSG00000123352	SPATS2	0.373814	3.250686	0.00022
1917	ENSG00000165678	GHITM	0.225539	7.874424	0.00022
1918	ENSG00000221866	PLXNA4	-0.67493	1.708626	0.00022
1919	ENSG00000205213	LGR4	-0.78716	0.802488	0.000221
1920	ENSG00000132749	MTL5	-0.72675	0.486898	0.000221
1921	ENSG00000171621	SPSB1	0.628838	2.800246	0.000222
1922	ENSG00000106636	YKT6	0.224775	6.609202	0.000222
1923	ENSG00000186088	GSAP	-0.39522	6.462881	0.000223
1924	ENSG00000238062	SPATA3-AS1	1.257124	-0.89416	0.000224
1925	ENSG00000171984	C20orf196	0.34432	2.618082	0.000224
1926	ENSG00000260290		0.244484	2.757363	0.000225
1927	ENSG00000076242	MLH1	0.248095	5.098166	0.000225
1928	ENSG00000173660	UQCRH	0.333111	6.645009	0.000226
1929	ENSG00000154719	MRPL39	0.421499	3.995111	0.000227
1930	ENSG00000142065	ZFP14	-0.42283	3.533708	0.000228
1931	ENSG00000123870	ZNF137P	-0.55646	1.518057	0.000228
1932	ENSG00000211645	IGLV1-50	1.269599	0.425014	0.000231
1933	ENSG00000134594	RAB33A	0.791503	2.460766	0.000232

1934	ENSG00000178078	STAP2	0.692398	-0.58331	0.000232
1935	ENSG00000125944	HNRNPR	0.200939	7.855674	0.000232
1936	ENSG00000175155	YPEL2	-0.46081	6.252371	0.000232
1937	ENSG00000185950	IRS2	-0.70459	5.466109	0.000232
1938	ENSG00000246898	LINC00920	-0.91457	1.121847	0.000232
1939	ENSG00000260698		-0.64929	0.275031	0.000233
1940	ENSG00000242013	USP27X	-0.3874	1.60588	0.000233
1941	ENSG00000137074	APTX	0.239803	4.268193	0.000235
1942	ENSG00000228839		-0.60566	-0.09826	0.000235
1943	ENSG00000109184	DCUN1D4	-0.34593	4.758818	0.000235
1944	ENSG00000132109	TRIM21	0.44864	5.204158	0.000235
1945	ENSG00000110046	ATG2A	0.758198	6.058188	0.000236
1946	ENSG00000204852	TCTN1	-0.59404	2.198935	0.000236
1947	ENSG00000233966	UBE2SP1	0.521478	2.184008	0.000237
1948	ENSG00000047365	ARAP2	0.437532	8.029905	0.000237
1949	ENSG00000103591	AAGAB	0.228204	5.665561	0.000237
1950	ENSG00000272498		-0.78521	1.50774	0.000237
1951	ENSG00000065923	SLC9A7	-0.4888	2.31589	0.000238
1952	ENSG00000150712	MTMR12	0.258226	5.712814	0.000238
1953	ENSG00000144642	RBMS3	-0.80798	-0.90686	0.000238
1954	ENSG00000268222	EEF1A1P7	-0.27952	8.238595	0.000238
1955	ENSG00000211727	TRBV7-6	0.825081	0.882324	0.000242
1956	ENSG00000198106	SNX29P2	-0.77255	0.975626	0.000242
1957	ENSG00000189319	FAM53B	0.472639	7.310932	0.000244
1958	ENSG00000130706	ADRM1	0.323803	5.456607	0.000244
1959	ENSG00000122025	FLT3	-1.06001	2.208989	0.000245
1960	ENSG00000172508	CARNS1	-0.56556	3.122257	0.000246
1961	ENSG00000197785	ATAD3A	0.354001	3.716364	0.000246
1962	ENSG00000226711	FAM66C	-0.70044	0.392832	0.000247
1963	ENSG00000198455	ZXDB	-0.33193	4.995938	0.000248
1964	ENSG00000167112	TRUB2	0.320876	3.436291	0.00025
1965	ENSG00000177409	SAMD9L	0.865925	7.603904	0.000252
1966	ENSG00000145194	ECE2	0.625963	0.953215	0.000252
1967	ENSG00000064309	CDON	-0.76953	-0.51763	0.000252
1968	ENSG00000186815	TPCN1	-0.62575	5.155447	0.000252
1969	ENSG00000172586	CHCHD1	0.368115	3.950593	0.000253
1970	ENSG00000172500	FIBP	0.403046	5.08244	0.000253
1971	ENSG00000092841	MYL6	0.313119	9.373586	0.000253
1972	ENSG00000263155	MYZAP	-1.13905	0.172617	0.000253
1973	ENSG00000104728	ARHGEF10	-0.66679	1.223765	0.000253
1974	ENSG00000229715	EEF1DP3	-0.68058	-0.171	0.000253
1975	ENSG00000100347	SAMM50	0.25506	4.875204	0.000254
1976	ENSG00000141696	LEPREL4	-0.7418	0.362285	0.000255
1977	ENSG00000189369	GSPT2	0.408153	2.971047	0.000255

1978	ENSG00000140905	GCSH	0.538724	1.600757	0.000255
1979	ENSG00000131368	MRPS25	-0.23289	5.867326	0.000256
1980	ENSG00000171365	CLCN5	-0.44897	3.471108	0.000257
1981	ENSG00000092199	HNRNPC	0.256539	9.534826	0.000258
1982	ENSG00000186204	CYP4F12	-1.31388	-1.68076	0.000259
1983	ENSG00000240065	PSMB9	0.84383	3.116221	0.000259
1984	ENSG00000245954		-0.73278	2.633755	0.000261
1985	ENSG00000111788		0.566247	2.334514	0.000262
1986	ENSG00000135624	CCT7	0.261639	6.903519	0.000262
1987	ENSG00000213619	NDUFS3	0.209897	5.609964	0.000262
1988	ENSG00000179456	ZBTB18	-0.50745	6.316005	0.000262
1989	ENSG00000139343	SNRPF	0.319423	4.965471	0.000263
1990	ENSG00000270505	IGHV1OR15-1	0.666184	2.835592	0.000264
1991	ENSG00000111537	IFNG	0.952245	4.092659	0.000264
1992	ENSG00000124243	BCAS4	0.378707	5.295291	0.000264
1993	ENSG00000070759	TESK2	-0.3542	3.523199	0.000265
1994	ENSG00000124097	HMGB1P1	0.725567	-0.56816	0.000266
1995	ENSG00000235531		0.560548	1.659362	0.000268
1996	ENSG00000197258	EIF4BP6	-0.30542	3.979563	0.000272
1997	ENSG00000181218	HIST3H2A	0.851586	0.55483	0.000273
1998	ENSG00000127955	GNAI1	-0.80124	-0.4129	0.000273
1999	ENSG00000178773	CPNE7	0.893729	-0.08121	0.000274
2000	ENSG00000168291	PDHB	0.238924	5.679285	0.000276
2001	ENSG00000127191	TRAF2	0.394659	3.739212	0.000276
2002	ENSG00000180644	PRF1	0.656894	8.419829	0.000276
2003	ENSG00000198169	ZNF251	-0.30478	3.574013	0.000276
2004	ENSG00000179091	CYC1	0.295252	5.637144	0.000277
2005	ENSG00000090530	LEPREL1	-1.0466	-1.24859	0.000277
2006	ENSG00000126351	THRA	-0.63266	4.681372	0.000278
2007	ENSG00000237693	IRGM	-1.06586	-1.37234	0.000278
2008	ENSG00000152953	STK32B	-1.05497	-0.93257	0.000279
2009	ENSG00000272078		0.817185	1.281599	0.00028
2010	ENSG00000122674	CCZ1	0.210271	5.949341	0.00028
2011	ENSG00000116824	CD2	0.416891	7.437144	0.000281
2012	ENSG00000140319	SRP14	0.293303	7.691144	0.000282
2013	ENSG00000184752	NDUFA12	0.371778	5.452671	0.000282
2014	ENSG00000139737	SLAIN1	0.29588	4.124705	0.000284
2015	ENSG00000180098	TRNAU1AP	0.255303	4.381577	0.000284
2016	ENSG00000144677	CTDSPL	-0.87106	2.820156	0.000284
2017	ENSG00000143870	PDIA6	0.343189	6.607761	0.000284
2018	ENSG00000225630	MTND2P28	-0.3882	10.42856	0.000284
2019	ENSG00000113716	HMGXB3	0.336753	5.529402	0.000285
2020	ENSG00000247596	TWF2	0.40106	5.409607	0.000285
2021	ENSG00000197265	GTF2E2	0.290618	4.875507	0.000285

2022	ENSG00000101298	SNPH	-0.46965	3.392025	0.000285
2023	ENSG00000197894	ADH5	0.250762	6.252582	0.000285
2024	ENSG00000186567	CEACAM19	-0.92961	-0.84965	0.000285
2025	ENSG00000257495		-0.85694	0.025036	0.000289
2026	ENSG00000102030	NAA10	0.266428	4.627423	0.000289
2027	ENSG00000132122	SPATA6	-0.77017	2.47311	0.000289
2028	ENSG00000150753	CCT5	0.315445	6.663695	0.000292
2029	ENSG00000239388	ASB14	-0.47467	1.573112	0.000292
2030	ENSG00000171421	MRPL36	0.340975	2.865994	0.000295
2031	ENSG00000168758	SEMA4C	-0.66286	4.696529	0.000295
2032	ENSG00000146376	ARHGAP18	0.407587	4.890637	0.000295
2033	ENSG00000162385	MAGOH	0.347355	5.336127	0.000295
2034	ENSG00000168411	RFWD3	0.354029	5.36179	0.000296
2035	ENSG00000139180	NDUFA9	0.296245	4.55689	0.000296
2036	ENSG00000102172	SMS	0.331859	5.593247	0.000298
2037	ENSG00000239571	IGKV2D-30	1.100547	2.213711	0.000299
2038	ENSG00000095303	PTGS1	-0.78563	5.660867	0.000302
2039	ENSG00000044446	PHKA2	-0.21829	5.246936	0.000303
2040	ENSG00000038382	TRIO	-0.45505	5.430263	0.000303
2041	ENSG00000161654	LSM12	0.20647	5.841862	0.000304
2042	ENSG00000009830	POMT2	-0.25129	2.71351	0.000304
2043	ENSG00000162073	PAQR4	0.830383	1.714507	0.000306
2044	ENSG00000167377	ZNF23	-0.52614	0.627864	0.000306
2045	ENSG00000196372	ASB13	-0.85178	2.363268	0.000306
2046	ENSG00000080819	CPOX	0.460165	4.339075	0.000307
2047	ENSG00000147894	C9orf72	-0.45529	7.312376	0.000307
2048	ENSG00000100442	FKBP3	0.329807	4.586844	0.000309
2049	ENSG00000183666	GUSBP1	-0.35332	2.796276	0.000309
2050	ENSG00000050030	KIAA2022	-0.93672	-0.60496	0.000309
2051	ENSG00000143167	GPA33	-0.55726	3.125539	0.00031
2052	ENSG00000243156	MICAL3	-0.61564	2.696201	0.00031
2053	ENSG00000088827	SIGLEC1	1.811414	4.214901	0.00031
2054	ENSG00000103995	CEP152	0.375057	4.02711	0.00031
2055	ENSG00000163468	CCT3	0.288727	7.176806	0.00031
2056	ENSG00000147394	ZNF185	-0.68036	5.150091	0.000312
2057	ENSG00000267074		0.466116	3.779136	0.000312
2058	ENSG00000013573	DDX11	0.668102	3.623319	0.000312
2059	ENSG00000134779	TPGS2	0.294048	5.451339	0.000312
2060	ENSG00000163935	SFMBT1	-0.41496	4.472944	0.000313
2061	ENSG00000260844		-0.99048	-0.62937	0.000313
2062	ENSG00000267251		1.575235	-1.07081	0.000314
2063	ENSG00000235036		0.452684	0.643356	0.000315
2064	ENSG00000135596	MICAL1	-0.24836	6.936984	0.00032
2065	ENSG00000080822	CLDND1	0.478604	7.424758	0.00032

2066	ENSG00000242472	IGHJ5	1.08586	-0.75879	0.000322
2067	ENSG00000211650	IGLV5-45	1.294188	-0.2653	0.000322
2068	ENSG00000145331	TRMT10A	0.402459	2.451921	0.000324
2069	ENSG00000166704	ZNF606	-0.42108	2.455561	0.000324
2070	ENSG00000134769	DTNA	-0.91157	-0.7451	0.000324
2071	ENSG00000104689	TNFRSF10A	-0.3965	4.112451	0.000325
2072	ENSG00000115514	TXNDC9	0.42835	4.586101	0.000327
2073	ENSG00000136522	MRPL47	0.412612	5.215225	0.000328
2074	ENSG00000070010	UFD1L	0.254695	6.255903	0.000328
2075	ENSG00000108798	ABI3	0.533666	5.345539	0.00033
2076	ENSG00000132424	PNISR	-0.33105	8.629745	0.00033
2077	ENSG00000169689	STRA13	0.45362	3.686436	0.000332
2078	ENSG00000153064	BANK1	-0.69487	5.835883	0.000332
2079	ENSG00000105669	COPE	0.331106	6.215118	0.000333
2080	ENSG00000187134	AKR1C1	-0.6756	0.267645	0.000337
2081	ENSG00000271620	IGHV3OR16-7	1.339246	-2.07108	0.000338
2082	ENSG00000100290	BIK	0.69388	0.312781	0.000341
2083	ENSG00000259516	ANP32AP1	0.386908	2.329587	0.000341
2084	ENSG00000214595	EML6	-0.56135	2.064572	0.000341
2085	ENSG00000014138	POLA2	0.404466	3.510649	0.000343
2086	ENSG00000137522	RNF121	0.230069	3.838442	0.000344
2087	ENSG00000223509		-0.44391	4.125568	0.000346
2088	ENSG00000164530	PI16	-0.96115	0.978355	0.000346
2089	ENSG00000103353	UBFD1	0.27478	5.083113	0.000346
2090	ENSG00000138735	PDE5A	-0.51841	3.510381	0.000348
2091	ENSG00000144152	FBLN7	-0.83296	1.355202	0.000348
2092	ENSG00000198130	HIBCH	0.391296	3.47979	0.000349
2093	ENSG00000228775	WEE2-AS1	-0.69678	-0.37409	0.000352
2094	ENSG00000172339	ALG14	0.527931	1.801504	0.000354
2095	ENSG00000168811	IL12A	0.532372	1.741766	0.000354
2096	ENSG00000251039	IGKV2D-40	1.071919	0.388624	0.000356
2097	ENSG00000148175	STOM	0.425597	7.510472	0.000357
2098	ENSG00000114423	CBLB	0.393424	7.032014	0.000357
2099	ENSG00000049089	COL9A2	-0.58992	2.348294	0.000357
2100	ENSG00000165071	TMEM71	-0.32236	6.452121	0.000358
2101	ENSG00000249210	GAPDHP38	0.563745	-0.11355	0.000359
2102	ENSG00000068383	INPP5A	-0.3388	3.55873	0.00036
2103	ENSG00000108294	PSMB3	0.337384	6.481367	0.000362
2104	ENSG00000230091	TMEM254-AS1	-0.72718	-0.82192	0.000364
2105	ENSG00000108039	XPNPEP1	0.22708	6.255181	0.000364
2106	ENSG00000263417	GTSCR1	-1.01196	-1.43037	0.000365
2107	ENSG00000121380	BCL2L14	1.122681	-0.45102	0.000366
2108	ENSG00000143942	CHAC2	0.638935	1.588113	0.000367
2109	ENSG00000163568	AIM2	0.793551	3.578473	0.000367

2110	ENSG00000211972	IGHV3-66	1.241684	-0.04869	0.000367
2111	ENSG00000136895	GARNL3	-0.7113	-0.63875	0.000367
2112	ENSG00000172215	CXCR6	1.05493	3.874412	0.000368
2113	ENSG00000177294	FBXO39	0.996668	-0.84735	0.000368
2114	ENSG00000224159	HMGB1P9	0.820288	-0.27277	0.000368
2115	ENSG00000259838	TCEB1P2	0.488091	2.137429	0.000368
2116	ENSG00000177324	BEND2	-1.07044	2.127245	0.000368
2117	ENSG00000213650		0.45453	2.19425	0.00037
2118	ENSG00000272005		-0.42678	2.635643	0.000372
2119	ENSG00000148935	GAS2	-0.71244	-0.8429	0.000372
2120	ENSG00000104518	GSDMD	0.46687	5.991862	0.000372
2121	ENSG00000158813	EDA	-0.72192	0.835252	0.000373
2122	ENSG00000165609	NUDT5	0.283923	5.455953	0.000373
2123	ENSG00000184209	SNRNP35	0.259552	3.955522	0.000375
2124	ENSG00000138642	HERC6	0.676361	5.217175	0.000376
2125	ENSG00000186260	MKL2	-0.39179	4.010634	0.000376
2126	ENSG00000251369	ZNF550	-0.44077	3.946013	0.000376
2127	ENSG00000138639	ARHGAP24	-0.67585	3.326376	0.000376
2128	ENSG00000182809	CRIP2	-0.82241	1.200187	0.000377
2129	ENSG00000213199	ASIC3	-0.58165	0.7641	0.000378
2130	ENSG00000175334	BANF1	0.4007	5.37454	0.000379
2131	ENSG00000178202	KDELC2	0.336799	4.281642	0.00038
2132	ENSG00000184305	CCSER1	-0.6332	0.546054	0.000381
2133	ENSG00000087087	SRRT	0.349792	6.839597	0.000381
2134	ENSG00000111906	HDDC2	0.380814	4.807638	0.000382
2135	ENSG00000174099	MSRB3	-0.64571	1.602857	0.000385
2136	ENSG00000108924	HLF	-0.71218	0.91429	0.000385
2137	ENSG00000135473	PAN2	-0.34534	5.666895	0.000386
2138	ENSG00000168397	ATG4B	0.262435	5.672889	0.000387
2139	ENSG00000051825	MPHOSPH9	0.308885	5.927567	0.000389
2140	ENSG00000260461		-0.8167	1.48296	0.000389
2141	ENSG00000211935	IGHV1-3	1.062623	0.256209	0.00039
2142	ENSG00000224272		1.341432	-0.88032	0.00039
2143	ENSG00000225783	MIAT	0.533907	7.728061	0.00039
2144	ENSG00000267454	ZNF582-AS1	-0.6008	0.044987	0.00039
2145	ENSG00000253930		-0.59071	0.614658	0.000392
2146	ENSG00000152104	PTPN14	-0.71517	-0.61091	0.000392
2147	ENSG00000122565	CBX3	0.249027	7.582484	0.000392
2148	ENSG00000134215	VAV3	-0.3201	5.86734	0.000392
2149	ENSG00000126107	HECTD3	0.219963	5.081264	0.000396
2150	ENSG00000135127	CCDC64	0.528506	5.413348	0.000397
2151	ENSG00000156463	SH3RF2	0.882973	-0.29833	0.000397
2152	ENSG00000171100	MTM1	-0.39242	5.058889	0.000398
2153	ENSG00000198909	MAP3K3	-0.35851	6.787577	0.0004

2154	ENSG00000235082	SUMO1P3	0.38844	0.890387	0.0004
2155	ENSG00000107611	CUBN	-0.70247	2.864902	0.0004
2156	ENSG00000272565		0.438812	5.032042	0.000401
2157	ENSG00000214810	CYCSP55	0.453695	2.083638	0.000401
2158	ENSG00000168924	LETM1	0.268111	4.852626	0.000401
2159	ENSG00000260396		-0.89545	-1.09264	0.000404
2160	ENSG00000117322	CR2	-0.77388	2.274567	0.000406
2161	ENSG00000026297	RNASET2	-0.31363	6.951111	0.000407
2162	ENSG00000154930	ACSS1	0.304101	6.351804	0.000407
2163	ENSG00000165716	FAM69B	-0.58033	0.161669	0.000408
2164	ENSG00000151090	THRB	-0.92674	-1.16782	0.000408
2165	ENSG00000105402	NAPA	0.424013	6.779235	0.00041
2166	ENSG00000226742	HSBP1L1	-0.46249	2.757285	0.00041
2167	ENSG00000143603	KCNN3	0.947374	-0.43293	0.00041
2168	ENSG00000186462	NAP1L2	-0.58659	2.052909	0.000411
2169	ENSG00000135525	MAP7	-0.75147	-0.24925	0.000411
2170	ENSG00000133424	LARGE	-0.72221	1.829918	0.000413
2171	ENSG00000114126	TFDP2	0.392731	5.161608	0.000415
2172	ENSG00000000460	C1orf112	0.414627	2.68669	0.000416
2173	ENSG00000229153	EPHA1-AS1	-0.77543	1.335303	0.000416
2174	ENSG00000227165	WDR11-AS1	-0.8914	0.602499	0.000416
2175	ENSG00000172531	PPP1CA	0.446323	7.220474	0.000417
2176	ENSG00000010219	DYRK4	0.27881	3.31701	0.000417
2177	ENSG00000140948	ZCCHC14	-0.42333	4.376139	0.000417
2178	ENSG00000184009	ACTG1	0.479528	10.21446	0.000417
2179	ENSG00000147419	CCDC25	0.25379	5.409031	0.000418
2180	ENSG00000143797	MBOAT2	-0.57218	2.739172	0.000419
2181	ENSG00000172005	MAL	-0.61411	4.638319	0.000419
2182	ENSG00000125656	CLPP	0.393951	4.419301	0.000422
2183	ENSG00000166289	PLEKHF1	0.756147	4.109143	0.000423
2184	ENSG00000065268	WDR18	0.448326	3.362882	0.000425
2185	ENSG00000131018	SYNE1	0.383622	8.741186	0.000425
2186	ENSG00000230584	CCT5P2	0.474997	0.275762	0.000425
2187	ENSG00000269970		-0.9	-0.29032	0.000428
2188	ENSG00000163611	SPICE1	-0.34035	3.80757	0.000429
2189	ENSG00000259093		0.519469	1.272339	0.00043
2190	ENSG00000104725	NEFL	-1.08909	0.301511	0.00043
2191	ENSG00000104341	LAPTM4B	-0.59115	2.152015	0.000431
2192	ENSG00000172115	CYCS	0.362021	6.912991	0.000431
2193	ENSG00000156831	NSMCE2	0.27713	4.154395	0.000431
2194	ENSG00000186564	FOXD2	0.495004	0.200947	0.000432
2195	ENSG00000106479	ZNF862	-0.47483	4.741522	0.000432
2196	ENSG00000104886	PLEKHJ1	0.389536	5.079681	0.000434
2197	ENSG00000154917	RAB6B	-0.57073	1.31608	0.000435

2198	ENSG00000156127	BATF	0.699174	3.982998	0.000437
2199	ENSG00000221923	ZNF880	-0.4662	2.605887	0.000437
2200	ENSG00000261557	EEF1A1P38	-0.26552	8.800135	0.000438
2201	ENSG00000164978	NUDT2	0.425924	3.544257	0.00044
2202	ENSG00000206077	ZDHHC11B	-1.08704	1.28871	0.00044
2203	ENSG00000140386	SCAPER	-0.33788	4.395934	0.00044
2204	ENSG00000079387	SENP1	0.27149	5.164452	0.000441
2205	ENSG00000107077	KDM4C	-0.22516	6.196398	0.000441
2206	ENSG00000205707	LYRM5	-0.31828	3.534143	0.000441
2207	ENSG00000145949	MYLK4	-0.66095	0.544294	0.000441
2208	ENSG00000169224	GCSAML	-0.95217	3.157282	0.000441
2209	ENSG00000163751	CPA3	-0.83621	2.711626	0.000442
2210	ENSG00000233155	HMGA1P8	0.620371	-0.80977	0.000443
2211	ENSG00000187531	SIRT7	0.205166	5.699201	0.000444
2212	ENSG00000272505		-0.76862	0.100204	0.000444
2213	ENSG00000118432	CNR1	-1.19136	-1.07668	0.000444
2214	ENSG00000140374	ETFA	0.318425	5.992516	0.000444
2215	ENSG00000153310	FAM49B	0.265008	8.244901	0.000444
2216	ENSG00000079805	DNM2	0.251919	7.616649	0.000446
2217	ENSG00000128335	APOL2	0.505616	5.372073	0.000449
2218	ENSG00000062524	LTK	-0.55595	2.961333	0.000452
2219	ENSG00000173193	PARP14	0.558198	8.172691	0.000455
2220	ENSG00000167920	TMEM99	0.493977	1.225337	0.000457
2221	ENSG00000050405	LIMA1	0.519992	3.801222	0.000457
2222	ENSG00000153666	GOLGA8I	-0.49879	1.079969	0.000457
2223	ENSG00000142089	IFITM3	0.778389	8.04363	0.00046
2224	ENSG00000161010	C5orf45	-0.35615	4.661044	0.000462
2225	ENSG00000137073	UBAP2	0.291645	4.605831	0.000466
2226	ENSG00000145365	TIFA	0.482059	4.724341	0.000466
2227	ENSG00000117461	PIK3R3	0.525908	2.64038	0.000467
2228	ENSG00000197880	MDS2	-0.90709	0.397187	0.000467
2229	ENSG00000255354		-0.72123	1.578716	0.000467
2230	ENSG00000167476	JSRP1	0.631512	0.003155	0.000469
2231	ENSG00000249978	TRGV7	0.780385	1.149584	0.00047
2232	ENSG00000154265	ABCA5	-0.54271	5.663061	0.00047
2233	ENSG00000204161	C10orf128	-0.57469	4.424879	0.000473
2234	ENSG00000117519	CNN3	-0.82317	0.242513	0.000473
2235	ENSG00000232104		0.695	0.372639	0.000474
2236	ENSG00000065534	MYLK	-0.8036	3.069553	0.000475
2237	ENSG00000240040		0.846507	0.060737	0.000477
2238	ENSG00000161692	DBF4B	0.517178	1.626233	0.000478
2239	ENSG00000234028		-0.52932	1.717662	0.000478
2240	ENSG00000145246	ATP10D	-0.54617	4.897263	0.000478
2241	ENSG00000066933	MYO9A	-0.31619	5.59437	0.000479

2242	ENSG00000188738	FSIP2	-1.02328	-0.59112	0.000479
2243	ENSG00000272909		-0.53722	0.972105	0.00048
2244	ENSG00000197852	FAM212B	-0.72823	3.370282	0.00048
2245	ENSG00000246627	CACNA1C-AS1	-0.71655	0.042961	0.000482
2246	ENSG00000244560		-0.53287	1.707154	0.000483
2247	ENSG00000134986	NREP	-0.54655	2.96583	0.000483
2248	ENSG00000100124	ANKRD54	0.278904	3.43668	0.000484
2249	ENSG00000089169	RPH3A	-1.13987	2.168429	0.000488
2250	ENSG00000169435	RASSF6	-1.02351	0.456032	0.000488
2251	ENSG00000272763		1.068107	-1.37194	0.000488
2252	ENSG00000135148	TRAFD1	0.490482	6.019479	0.000494
2253	ENSG00000226976	COX6A1P2	0.432558	2.389068	0.000498
2254	ENSG00000142303	ADAMTS10	-0.70627	3.357533	0.000498
2255	ENSG00000171428	NAT1	0.517339	3.065884	0.000498
2256	ENSG00000134297	PLEKHA8P1	-0.47095	1.824518	0.000498
2257	ENSG00000032389	TSSC1	0.352167	4.467642	0.0005
2258	ENSG00000259243	GOLGA6L19	-0.7391	-0.67689	0.0005
2259	ENSG00000197296	FITM2	-0.43217	2.253792	0.000502
2260	ENSG00000244437	IGKV3-15	1.09975	3.947772	0.000504
2261	ENSG00000101846	STS	-0.34118	3.761581	0.000504
2262	ENSG00000161996	WDR90	0.420744	2.229397	0.000504
2263	ENSG00000081019	RSBN1	-0.31276	6.13532	0.000505
2264	ENSG00000105401	CDC37	0.177025	7.03537	0.000509
2265	ENSG00000174669	SLC29A2	-0.45171	1.873481	0.000509
2266	ENSG00000109534	GAR1	0.303414	3.66238	0.00051
2267	ENSG00000198258	UBL5	0.265137	6.250337	0.000512
2268	ENSG00000145916	RMND5B	-0.39382	3.991289	0.000513
2269	ENSG00000123892	RAB38	0.609813	1.141348	0.000514
2270	ENSG00000249986	YWHAQP6	0.336297	1.698775	0.000515
2271	ENSG00000254709	IGLL5	0.890367	1.802913	0.000516
2272	ENSG00000164919	COX6C	0.377738	6.454241	0.000516
2273	ENSG00000088808	PPP1R13B	-0.48981	3.494228	0.000516
2274	ENSG00000139173	TMEM117	-0.52033	1.217807	0.000516
2275	ENSG00000157404	KIT	-0.83092	1.191162	0.000516
2276	ENSG00000226137	BAIAP2-AS1	-0.96325	2.514664	0.000516
2277	ENSG00000272282		0.607136	3.043525	0.000517
2278	ENSG00000115207	GTF3C2	-0.19051	5.414809	0.000518
2279	ENSG00000153885	KCTD15	-0.66069	3.052045	0.000518
2280	ENSG00000168062	BATF2	1.430952	0.428854	0.000518
2281	ENSG00000251867		-0.55455	1.19192	0.000518
2282	ENSG00000255837	TAS2R20	-0.58429	0.514494	0.000518
2283	ENSG00000230207	RPL4P5	-0.324	5.320501	0.000519
2284	ENSG00000164543	STK17A	0.545593	7.293603	0.00052
2285	ENSG00000219102	HNRNPA3P12	0.440746	-0.1655	0.000521

2286	ENSG00000240219		-0.49265	1.991856	0.000522
2287	ENSG00000143420	ENSA	0.224542	7.309297	0.000522
2288	ENSG00000250462	LRRC37BP1	-0.26183	2.738689	0.000522
2289	ENSG00000100997	ABHD12	0.285685	4.647877	0.000523
2290	ENSG00000179051	RCC2	0.31444	6.651177	0.000523
2291	ENSG00000168701	TMEM208	0.302612	4.529277	0.000524
2292	ENSG00000266208		0.644035	-0.15528	0.000526
2293	ENSG00000100567	PSMA3	0.296231	6.4792	0.000526
2294	ENSG00000176715	ACSF3	0.298181	5.17022	0.000528
2295	ENSG00000135632	SMYD5	-0.324	4.082822	0.000529
2296	ENSG00000165702	GFI1B	-0.84478	2.68097	0.00053
2297	ENSG00000271895		-0.5012	3.161128	0.00053
2298	ENSG00000105393	BABAM1	0.242798	4.775708	0.000531
2299	ENSG00000128294	TPST2	0.392723	6.030802	0.000534
2300	ENSG00000010030	ETV7	1.217841	1.62097	0.000542
2301	ENSG00000237298	TTN-AS1	-0.34154	3.45501	0.000542
2302	ENSG00000229645	LINC00341	-0.35407	4.653006	0.000542
2303	ENSG00000119185	ITGB1BP1	0.296074	5.659569	0.000545
2304	ENSG00000180879	SSR4	0.252957	7.04104	0.000545
2305	ENSG00000196724	ZNF418	-0.67138	0.589686	0.000545
2306	ENSG00000223886		0.513284	1.559574	0.000546
2307	ENSG00000165113	GKAP1	-0.53695	2.366179	0.000546
2308	ENSG00000166105	GLB1L3	0.501429	2.392951	0.000548
2309	ENSG00000080298	RFX3	-0.40675	5.199264	0.00055
2310	ENSG00000204946	ZNF783	-0.22552	4.787681	0.000552
2311	ENSG00000249047	COX6B1P5	0.769007	-0.68042	0.000552
2312	ENSG00000174876	AMY1B	-0.53371	0.662304	0.000552
2313	ENSG00000254748	HNRNPCP8	0.439282	-0.09858	0.000557
2314	ENSG00000163113	OTUD7B	0.340739	3.144518	0.000559
2315	ENSG00000111331	OAS3	0.98565	6.90796	0.00056
2316	ENSG00000167972	ABCA3	-0.58487	2.497797	0.000561
2317	ENSG00000189144	ZNF573	-0.35856	2.02042	0.000562
2318	ENSG00000230626		0.391663	2.468576	0.000563
2319	ENSG00000246922	UBAP1L	-0.41997	3.400552	0.000565
2320	ENSG00000213463	SYNJ2BP	-0.32438	3.126261	0.000567
2321	ENSG00000151465	CDC123	0.252087	5.84608	0.000567
2322	ENSG00000146263	MMS22L	0.351803	4.058206	0.000568
2323	ENSG00000198740	ZNF652	-0.2824	5.683911	0.000569
2324	ENSG00000188868	ZNF563	-0.6206	1.06002	0.000571
2325	ENSG00000103363	TCEB2	0.320408	6.363002	0.000572
2326	ENSG00000253719	ATXN7L3B	-0.19575	6.891256	0.000573
2327	ENSG00000178538	CA8	-0.73922	0.187293	0.000574
2328	ENSG00000142235	LMTK3	-0.69294	1.178929	0.000574
2329	ENSG00000228167		0.557732	0.247887	0.000578

2330	ENSG00000160183	TMPRSS3	0.631981	0.793511	0.000578
2331	ENSG00000168502	SOGA2	-0.71381	-0.86553	0.000578
2332	ENSG00000214077	GNAQP1	-0.76593	-0.52958	0.000579
2333	ENSG00000148600	CDHR1	-0.58931	1.108428	0.000579
2334	ENSG00000062822	POLD1	0.477985	3.378666	0.000586
2335	ENSG00000149923	PPP4C	0.298487	6.268634	0.000586
2336	ENSG00000260807		-0.74667	2.006205	0.000586
2337	ENSG00000079257	LXN	0.442795	2.394962	0.000586
2338	ENSG00000187522	HSPA14	0.297228	5.527212	0.000588
2339	ENSG00000174807	CD248	0.919308	0.809769	0.00059
2340	ENSG00000248415	GAPDHP61	0.429269	1.239842	0.000594
2341	ENSG00000176124	DLEU1	0.288888	2.424966	0.000596
2342	ENSG00000184465	WDR27	-0.4459	3.388831	0.000597
2343	ENSG00000198182	ZNF607	-0.62873	1.83969	0.0006
2344	ENSG00000243836	WDR86-AS1	-0.65922	1.145397	0.000602
2345	ENSG00000168016	TRANK1	0.547073	6.962853	0.000603
2346	ENSG00000173692	PSMD1	0.303861	6.107866	0.000603
2347	ENSG00000018869	ZNF582	-0.49223	1.644587	0.000604
2348	ENSG00000138755	CXCL9	1.118254	1.367251	0.000605
2349	ENSG00000132274	TRIM22	0.481059	8.881336	0.000605
2350	ENSG00000163827	LRRC2	1.125624	-1.13843	0.000607
2351	ENSG00000116133	DHCR24	0.694575	3.194555	0.000609
2352	ENSG00000152207	CYSLTR2	-0.71448	3.050348	0.00061
2353	ENSG00000187051	RPS19BP1	0.275563	5.491601	0.000611
2354	ENSG00000183520	UTP11L	0.364535	4.253903	0.000612
2355	ENSG00000135387	CAPRIN1	0.215936	7.429364	0.000613
2356	ENSG00000259419	HNRNPCP3	0.296393	3.277018	0.000614
2357	ENSG00000213740	SERBP1P1	0.299651	4.19198	0.000614
2358	ENSG00000101082	SLA2	0.850127	6.714954	0.000616
2359	ENSG00000167702	KIFC2	-0.36962	3.873631	0.000619
2360	ENSG00000188130	MAPK12	0.637932	-0.17509	0.00062
2361	ENSG00000067066	SP100	0.294015	7.796227	0.000621
2362	ENSG00000233999	IGKV3OR2-268	1.041847	1.513133	0.000623
2363	ENSG00000096093	EFHC1	-0.41546	3.034662	0.000625
2364	ENSG00000132383	RPA1	0.266597	6.37089	0.000625
2365	ENSG00000215005	HSPD1P7	0.515176	0.741918	0.000626
2366	ENSG00000143702	CEP170	-0.39513	5.628211	0.000626
2367	ENSG00000174175	SELP	-0.75974	2.384357	0.000628
2368	ENSG00000143466	IKBKE	0.332688	5.505844	0.00063
2369	ENSG00000120832	MTERFD3	-0.41645	3.023005	0.000631
2370	ENSG00000198298	ZNF485	-0.51435	0.925266	0.000631
2371	ENSG00000249476		-0.69983	0.538264	0.000631
2372	ENSG00000144290	SLC4A10	-0.90273	3.300091	0.000631
2373	ENSG00000138663	COPS4	0.321332	5.39972	0.000632

2374	ENSG00000243238	IGKV2-30	1.076034	2.883578	0.000632
2375	ENSG00000182952	HMGN4	0.262341	6.678191	0.000632
2376	ENSG00000177084	POLE	0.240649	5.170053	0.000632
2377	ENSG00000188747	NOXA1	-0.5742	1.494136	0.000632
2378	ENSG00000182117	NOP10	0.370653	5.784457	0.000634
2379	ENSG00000188687	SLC4A5	-0.47046	0.499068	0.000634
2380	ENSG00000229769	TRBV10-2	0.845459	0.185296	0.000636
2381	ENSG00000258645	HSPE1P2	0.676901	-0.21383	0.000639
2382	ENSG00000211639	IGLV4-60	1.486207	-0.50602	0.000646
2383	ENSG00000160298	C21orf58	0.547179	1.386709	0.000648
2384	ENSG00000246982		-0.67307	0.163208	0.000648
2385	ENSG00000172348	RCAN2	0.883717	1.914415	0.000653
2386	ENSG00000100764	PSMC1	0.251952	6.717791	0.000653
2387	ENSG00000185052	SLC24A3	-0.83325	0.22305	0.000654
2388	ENSG00000177479	ARIH2	0.157962	6.90602	0.000656
2389	ENSG00000124602	UNC5CL	-0.5114	0.37264	0.000656
2390	ENSG00000196793	ZNF239	-0.61474	-0.04035	0.000656
2391	ENSG00000125458	NT5C	0.30231	3.929383	0.000657
2392	ENSG00000101460	MAP1LC3A	0.686929	1.867543	0.00066
2393	ENSG00000146574	CCZ1B	0.212195	5.55142	0.00066
2394	ENSG00000196533	C1orf186	-0.56344	1.121114	0.000661
2395	ENSG00000115641	FHL2	0.474504	1.766035	0.000662
2396	ENSG00000182173	TSEN54	0.425932	6.084345	0.000662
2397	ENSG00000122188	LAX1	0.548089	4.774297	0.000666
2398	ENSG00000160318	CLDND2	0.544777	2.435831	0.000668
2399	ENSG00000135052	GOLM1	-0.40653	4.799887	0.000668
2400	ENSG00000168614	NBPF9	-0.28551	5.604536	0.000669
2401	ENSG00000170190	SLC16A5	-0.72348	2.760133	0.000669
2402	ENSG00000100342	APOL1	0.640309	4.772007	0.000672
2403	ENSG00000071794	HLTF	0.377413	4.448874	0.000674
2404	ENSG00000142252	GEMIN7	0.359548	3.245445	0.000676
2405	ENSG00000168899	VAMP5	0.616807	5.136803	0.000677
2406	ENSG00000243264	IGKV2D-29	1.325018	0.955037	0.000677
2407	ENSG00000180354	MTURN	-0.43558	5.966353	0.00068
2408	ENSG00000166313	APBB1	-0.44078	4.867012	0.000681
2409	ENSG00000261589		-0.64317	0.610247	0.000681
2410	ENSG00000150681	RGS18	-0.66522	6.088982	0.000681
2411	ENSG00000104522	TSTA3	0.414531	4.726472	0.000683
2412	ENSG00000114446	IFT57	-0.2722	5.13143	0.000684
2413	ENSG00000038210	PI4K2B	0.439897	5.702836	0.000686
2414	ENSG00000176928	GCNT4	-0.79257	3.605444	0.000686
2415	ENSG00000074527	NTN4	-0.93036	0.111492	0.000686
2416	ENSG00000272734	ADIRF-AS1	-0.61542	1.251448	0.000686
2417	ENSG00000125743	SNRPD2	0.314204	6.90946	0.000689

2418	ENSG00000109390	NDUFC1	0.286724	4.083598	0.000689
2419	ENSG00000182872	RBM10	0.246029	5.443949	0.000689
2420	ENSG00000226791		0.494342	0.966292	0.00069
2421	ENSG00000182195	LDOC1	-0.71993	0.659749	0.000691
2422	ENSG00000079691	LRRC16A	-0.51028	2.034247	0.000691
2423	ENSG00000162576	MXRA8	-0.59137	0.989436	0.000692
2424	ENSG00000128596	CCDC136	-0.52879	1.917543	0.000701
2425	ENSG00000100142	POLR2F	0.27528	4.163686	0.000703
2426	ENSG00000037280	FLT4	-0.84711	1.434873	0.000703
2427	ENSG00000181359	HSP90AA6P	0.590584	2.789015	0.000704
2428	ENSG00000066468	FGFR2	-0.8752	0.883696	0.000704
2429	ENSG00000125166	GOT2	0.356197	4.763463	0.000705
2430	ENSG00000213625	LEPROT	-0.36535	6.329895	0.000705
2431	ENSG00000178965	C1orf173	1.30287	-0.64142	0.000705
2432	ENSG00000197670		0.569056	0.76461	0.000706
2433	ENSG00000218996		-0.62597	1.730689	0.000707
2434	ENSG00000170089		0.271797	4.054723	0.000707
2435	ENSG00000141552	ANAPC11	0.315482	4.37295	0.000708
2436	ENSG00000263327	TAPT1-AS1	-0.58414	1.142261	0.000708
2437	ENSG00000025770	NCAPH2	0.344869	5.37211	0.00071
2438	ENSG00000176700	SCAND2P	-0.26219	3.830444	0.00071
2439	ENSG00000173786	CNP	0.404096	5.533323	0.000712
2440	ENSG00000182004	SNRPE	0.314504	5.264174	0.000712
2441	ENSG00000225663	FAM195B	0.322205	5.357374	0.000713
2442	ENSG00000088280	ASAP3	-0.71499	0.233101	0.000713
2443	ENSG00000243063	IGKV3-7	0.973702	0.329239	0.000714
2444	ENSG00000109436	TBC1D9	-0.50382	5.267358	0.000714
2445	ENSG00000141527	CARD14	-0.64051	0.219482	0.000714
2446	ENSG00000264350	SNRPGP2	0.478034	1.995329	0.000716
2447	ENSG00000119922	IFIT2	1.067499	6.587848	0.000716
2448	ENSG00000260302		0.690871	1.056438	0.000716
2449	ENSG00000211799	TRAV19	0.642838	2.063042	0.000719
2450	ENSG00000151320	AKAP6	-0.80427	-0.47273	0.00072
2451	ENSG00000171222	SCAND1	0.50492	4.542089	0.000721
2452	ENSG00000197903	HIST1H2BK	0.410345	3.131535	0.000721
2453	ENSG00000121390	PSPC1	0.2435	5.90275	0.000724
2454	ENSG00000110811	LEPREL2	-0.78598	-0.60139	0.000724
2455	ENSG00000100628	ASB2	0.551697	2.216242	0.000726
2456	ENSG00000133739	LRRCC1	0.47754	3.30625	0.000726
2457	ENSG00000132464	ENAM	1.049339	-0.36255	0.000727
2458	ENSG00000260910	LINC00565	-0.56951	2.159952	0.000731
2459	ENSG00000118363	SPCS2	0.314386	6.350809	0.000733
2460	ENSG00000264920		0.500152	0.901853	0.000734
2461	ENSG00000164251	F2RL1	-0.78936	1.691925	0.000735

2462	ENSG00000140688	C16orf58	-0.27697	4.287771	0.000735
2463	ENSG00000172428	MYEOV2	0.372796	4.439823	0.000737
2464	ENSG00000165169	DYNLT3	-0.32801	5.239929	0.000743
2465	ENSG00000197937	ZNF347	-0.56399	1.431657	0.000748
2466	ENSG00000159640	ACE	-0.64559	0.414157	0.00075
2467	ENSG00000116497	S100PBP	0.354673	5.321577	0.000755
2468	ENSG00000130518	KIAA1683	-0.56242	3.584845	0.000755
2469	ENSG00000168883	USP39	0.201498	5.846314	0.000756
2470	ENSG00000225756	DBH-AS1	-0.88855	0.16199	0.000756
2471	ENSG00000203326	ZNF525	-0.35005	4.220595	0.000756
2472	ENSG00000152042	NBPF11	-0.24772	5.525057	0.000757
2473	ENSG00000129646	QRICH2	-0.51648	1.466377	0.000758
2474	ENSG00000249931	GOLGA8K	-0.70482	0.424478	0.000762
2475	ENSG00000134330	IAH1	0.2464	4.979328	0.000764
2476	ENSG00000118200	CAMSAP2	-0.65332	2.594142	0.000764
2477	ENSG00000136014	USP44	-0.65826	0.914055	0.000764
2478	ENSG00000136147	PHF11	0.315507	6.413847	0.000765
2479	ENSG00000125246	CLYBL	-0.39166	2.372993	0.000766
2480	ENSG00000187726	DNAJB13	-0.87826	2.448379	0.00077
2481	ENSG00000160014	CALM3	0.359999	7.717771	0.00077
2482	ENSG00000171817	ZNF540	-0.71611	2.081526	0.000772
2483	ENSG00000231672	DIRC3	-0.72314	-0.54307	0.000773
2484	ENSG00000159784	FAM131B	-0.67464	0.31516	0.000775
2485	ENSG00000124571	XPO5	0.254983	5.191001	0.000777
2486	ENSG00000114942	EEF1B2	-0.3505	9.179257	0.000778
2487	ENSG00000065357	DGKA	-0.31729	7.477021	0.000778
2488	ENSG00000196267	ZNF836	-0.44645	2.75928	0.000778
2489	ENSG00000115841	RMDN2	-0.47445	2.195622	0.000778
2490	ENSG00000159335	PTMS	0.570299	3.32241	0.000779
2491	ENSG00000008324	SS18L2	0.328847	4.983642	0.000781
2492	ENSG00000174197	MGA	-0.30379	6.401529	0.000782
2493	ENSG00000184206	GOLGA6L4	-0.52271	1.014131	0.000785
2494	ENSG00000258824		-0.57131	-0.03154	0.000791
2495	ENSG00000197808	ZNF461	-0.39093	2.879507	0.000792
2496	ENSG00000235363	SNRPGP10	1.00793	-1.33717	0.000792
2497	ENSG00000154263	ABCA10	-0.73082	1.047591	0.000793
2498	ENSG00000271730		-0.81771	-0.69475	0.000793
2499	ENSG00000008086	CDKL5	-0.81062	0.655653	0.000801
2500	ENSG00000137815	RTF1	0.221862	6.571275	0.000803
2501	ENSG00000272168	CASC15	-0.84091	-0.72194	0.000803
2502	ENSG00000108700	CCL8	1.940696	-1.9302	0.000804
2503	ENSG00000205236		-0.51345	1.082676	0.000809
2504	ENSG00000119608	PROX2	-0.6132	0.516632	0.000812
2505	ENSG00000104325	DECR1	0.411289	6.173544	0.000813

2506	ENSG00000133302	ANKRD32	0.382084	4.274407	0.000813
2507	ENSG00000224298		0.928997	-0.45539	0.000816
2508	ENSG00000129084	PSMA1	0.241452	6.058244	0.000816
2509	ENSG00000180739	S1PR5	0.586138	6.063582	0.000817
2510	ENSG00000104154	SLC30A4	-0.45595	3.151608	0.000817
2511	ENSG00000206561	COLQ	-0.53661	2.890971	0.000817
2512	ENSG00000225791	TRAM2-AS1	-0.55449	1.021763	0.000817
2513	ENSG00000102678	FGF9	-0.88774	0.657714	0.000817
2514	ENSG00000211710	TRBV4-1	1.053728	2.109644	0.000819
2515	ENSG00000078319	PMS2P1	0.293491	1.925512	0.000819
2516	ENSG00000105486	LIG1	0.290023	4.824572	0.000819
2517	ENSG00000259225		-1.24744	-0.58138	0.00082
2518	ENSG00000168939	SPRY3	-0.42848	0.926017	0.00082
2519	ENSG00000246790		-0.86291	-0.68901	0.00082
2520	ENSG00000137177	KIF13A	-0.60623	4.593694	0.000821
2521	ENSG00000243417		-0.39044	4.937185	0.000822
2522	ENSG00000105982	RNF32	-0.37092	1.43075	0.000825
2523	ENSG00000177119	ANO6	-0.32286	6.640006	0.000826
2524	ENSG00000123297	TSFM	0.276568	3.527835	0.000826
2525	ENSG00000256862		-0.73898	1.097233	0.00083
2526	ENSG00000176293	ZNF135	-0.79961	-0.55103	0.000831
2527	ENSG00000087077	TRIP6	0.402092	2.219829	0.000834
2528	ENSG00000162852	CNST	-0.29078	6.23618	0.000836
2529	ENSG00000066027	PPP2R5A	0.217708	7.128495	0.000836
2530	ENSG00000196177	ACADSB	-0.30603	5.114912	0.000839
2531	ENSG00000100410	PHF5A	0.338997	5.063845	0.000842
2532	ENSG00000197312	DDI2	0.443942	5.835807	0.000842
2533	ENSG00000167325	RRM1	0.647011	5.920574	0.000843
2534	ENSG00000186834	HEXIM1	0.414203	6.330548	0.000844
2535	ENSG00000236287	ZBED5	-0.30812	6.280714	0.000844
2536	ENSG00000169223	LMAN2	0.321983	6.413204	0.000844
2537	ENSG00000259174		-0.84281	-0.17093	0.000845
2538	ENSG00000117748	RPA2	0.3332	5.814342	0.000846
2539	ENSG00000119408	NEK6	-0.49786	4.248066	0.000848
2540	ENSG00000205133	TRIQK	-0.6267	3.710902	0.000848
2541	ENSG00000253991		0.450334	0.762245	0.000861
2542	ENSG00000225648	SBDSP1	-0.36059	6.01737	0.000863
2543	ENSG00000110031	LPXN	0.489806	7.298504	0.00087
2544	ENSG00000233837	EIF3LP2	-0.38492	2.542304	0.00087
2545	ENSG00000127995	CASD1	-0.26839	5.610655	0.000871
2546	ENSG00000071967	CYBRD1	-0.86409	4.201729	0.000871
2547	ENSG00000196458	ZNF605	-0.53303	3.882146	0.000873
2548	ENSG00000203812	HIST2H2AA4	0.589848	2.957841	0.000875
2549	ENSG00000112578	BYSL	0.363607	2.375697	0.000875

2550	ENSG00000188227	ZNF793	-0.6058	1.604139	0.000875
2551	ENSG00000010292	NCAPD2	0.541428	5.561599	0.000879
2552	ENSG00000129521	EGLN3	-0.71834	1.137276	0.000879
2553	ENSG00000134285	FKBP11	0.368081	5.495407	0.000885
2554	ENSG00000100731	PCNX	-0.23965	7.605621	0.000885
2555	ENSG00000227191	TRGC2	0.510328	6.404572	0.000886
2556	ENSG00000227184	EPPK1	-0.77479	-0.14666	0.000892
2557	ENSG00000213930	GALT	-0.27479	4.767573	0.000894
2558	ENSG00000010310	GIPR	0.582254	2.576348	0.000894
2559	ENSG00000235326		0.61527	0.292924	0.000895
2560	ENSG00000105699	LSR	-0.56169	3.374941	0.000896
2561	ENSG00000198939	ZFP2	-0.80016	-0.95152	0.000896
2562	ENSG00000198040	ZNF84	-0.34337	4.561675	0.000897
2563	ENSG00000144445	KANSL1L	-0.38556	3.751188	0.000897
2564	ENSG00000174842	GLMN	0.463902	3.818624	0.000906
2565	ENSG00000166164	BRD7	0.328691	6.726981	0.000906
2566	ENSG00000171246	NPTX1	1.247066	-0.46106	0.000909
2567	ENSG00000253705	IGHV3-41	1.238732	-1.74097	0.000909
2568	ENSG00000133104	SPG20	-0.44233	5.214733	0.000909
2569	ENSG00000260655		-0.66293	3.095251	0.000909
2570	ENSG00000205726	ITSN1	-0.62139	2.56727	0.000911
2571	ENSG00000198690	FAN1	-0.45067	3.759237	0.000912
2572	ENSG00000188612	SUMO2	0.236629	6.733169	0.000912
2573	ENSG00000117262	GPR89A	0.220398	4.252418	0.000913
2574	ENSG00000211942	IGHV3-13	1.445019	-1.43184	0.00092
2575	ENSG00000153363	LINC00467	0.52661	0.367518	0.000921
2576	ENSG00000119673	ACOT2	0.380961	3.12917	0.000921
2577	ENSG00000232774		0.433007	0.983064	0.000921
2578	ENSG00000178860	MSC	0.733096	3.181938	0.000935
2579	ENSG00000136098	NEK3	-0.5117	2.621683	0.000938
2580	ENSG00000183207	RUVBL2	0.356043	4.337058	0.00094
2581	ENSG00000231167	YBX1P2	0.231839	5.341717	0.00094
2582	ENSG00000105676	ARMC6	0.394943	3.989033	0.00094
2583	ENSG00000074071	MRPS34	0.386403	4.531633	0.000948
2584	ENSG00000058866	DGKG	-0.74088	2.091984	0.000948
2585	ENSG00000006831	ADIPOR2	0.302871	6.561311	0.000951
2586	ENSG00000234771		-0.39142	3.884042	0.000951
2587	ENSG00000075651	PLD1	-0.52941	2.572245	0.000952
2588	ENSG00000102934	PLLP	-0.93765	-1.18775	0.000952
2589	ENSG00000066135	KDM4A	0.30007	5.026074	0.000954
2590	ENSG00000143951	WDPCP	-0.28569	2.765955	0.000954
2591	ENSG00000138439	FAM117B	-0.46967	5.099922	0.000954
2592	ENSG00000079335	CDC14A	-0.3695	5.89028	0.000955
2593	ENSG00000081189	MEF2C	-0.42822	5.839565	0.000958

2594	ENSG00000254667		0.653377	1.143772	0.000958
2595	ENSG00000124613	ZNF391	-0.71679	0.126601	0.000959
2596	ENSG00000104626	ERI1	0.346102	4.237215	0.00096
2597	ENSG00000151502	VPS26B	0.304604	6.590079	0.000966
2598	ENSG00000132432	SEC61G	0.306588	5.623027	0.000966
2599	ENSG00000136108	CKAP2	0.562531	5.567759	0.000969
2600	ENSG00000188033	ZNF490	0.402893	0.970323	0.000969
2601	ENSG00000261468		-0.4295	3.234977	0.00097
2602	ENSG00000198155	ZNF876P	-0.6972	-0.2932	0.00097
2603	ENSG00000272761		-0.72352	1.128673	0.000974
2604	ENSG00000170161		0.505565	-0.06383	0.000974
2605	ENSG00000249565	SERBP1P5	0.395464	1.271876	0.000976
2606	ENSG00000165802	NSMF	0.346559	5.779654	0.000976
2607	ENSG00000240535		0.573774	0.082948	0.000978
2608	ENSG00000148362	C9orf142	0.307342	5.689529	0.00098
2609	ENSG00000179855	GIPC3	-0.63696	0.562675	0.00098
2610	ENSG00000239557		0.364393	4.260506	0.000982
2611	ENSG00000162367	TAL1	-0.77953	1.825043	0.000982
2612	ENSG00000175866	BAIAP2	-0.53228	2.540714	0.000984
2613	ENSG00000176953	NFATC2IP	0.162165	6.398755	0.000985
2614	ENSG00000246089		0.557751	0.960416	0.000985
2615	ENSG00000249855	EEF1A1P19	-0.25888	11.19676	0.000986
2616	ENSG00000121406	ZNF549	-0.38914	4.051037	0.000986
2617	ENSG00000179088	C12orf42	-0.50354	1.199652	0.000986
2618	ENSG00000142444	C19orf52	0.25383	3.124204	0.000993
2619	ENSG00000240854		0.392536	2.175194	0.000994
2620	ENSG00000229180		-0.44716	2.9344	0.000994
2621	ENSG00000174775	HRAS	0.296306	3.169343	0.000995
2622	ENSG00000158552	ZFAND2B	0.203486	5.499621	0.000995
2623	ENSG00000157514	TSC22D3	-0.51785	9.804866	0.000995
2624	ENSG00000213757		-0.39697	3.858593	0.000999
2625	ENSG00000116459	ATP5F1	0.24811	7.347356	0.001005
2626	ENSG00000248180	GAPDHP60	0.422968	2.465727	0.001005
2627	ENSG00000211771	TRBJ2-7	0.634172	-0.37305	0.001008
2628	ENSG00000155657	TTN	-0.52748	6.019289	0.00101
2629	ENSG00000140470	ADAMTS17	-0.55673	0.630202	0.001016
2630	ENSG00000017260	ATP2C1	0.192927	6.272556	0.001016
2631	ENSG00000100554	ATP6V1D	0.369154	5.375151	0.001017
2632	ENSG00000145284	SCD5	0.640673	2.243559	0.001018
2633	ENSG00000178966	RMI1	0.487305	2.645235	0.00102
2634	ENSG00000068745	IP6K2	0.29458	6.029138	0.00102
2635	ENSG00000172465	TCEAL1	-0.35546	2.394491	0.00102
2636	ENSG00000168612	ZSWIM1	-0.39997	2.362889	0.00102
2637	ENSG00000119121	TRPM6	-0.76782	0.927161	0.00102

2638	ENSG00000152684	PELO	0.603717	3.519714	0.001022
2639	ENSG00000236152	MRPS36P1	0.919179	-0.85903	0.001024
2640	ENSG00000137103	TMEM8B	-0.5454	1.97274	0.001024
2641	ENSG00000215105	TTC3P1	-0.50415	0.932432	0.001024
2642	ENSG00000227986	TRIM60P18	-0.59606	0.421927	0.001028
2643	ENSG00000183625	CCR3	-0.73489	2.089996	0.001029
2644	ENSG00000231245	C1DP1	0.292986	3.356606	0.00103
2645	ENSG00000140968	IRF8	-0.35107	6.885078	0.001036
2646	ENSG00000269680		-0.62343	-0.57634	0.001036
2647	ENSG00000115604	IL18R1	-0.4231	4.659134	0.001041
2648	ENSG00000113971	NPHP3	-0.32793	5.010893	0.001041
2649	ENSG00000224660	SH3BP5-AS1	-0.35628	4.991408	0.001044
2650	ENSG00000107779	BMPR1A	-0.37499	3.474147	0.001045
2651	ENSG00000079819	EPB41L2	-0.35531	3.721447	0.001046
2652	ENSG00000214389	RPS3AP26	-0.36381	5.78007	0.001047
2653	ENSG00000205336	GPR56	0.577274	6.94631	0.00105
2654	ENSG00000105364	MRPL4	0.283903	3.915023	0.001053
2655	ENSG00000189180	ZNF33A	-0.357	6.42005	0.001055
2656	ENSG00000141741	MIEN1	0.266142	4.822854	0.001057
2657	ENSG00000149177	PTPRJ	0.42606	7.064425	0.001058
2658	ENSG00000204802		0.584517	-0.56458	0.001063
2659	ENSG00000254012		-0.2888	5.421959	0.001063
2660	ENSG00000133619	KRBA1	-0.33981	2.517687	0.001063
2661	ENSG00000179979	CRIPAK	-0.4186	3.500491	0.001075
2662	ENSG00000133265	HSPBP1	0.410759	3.43657	0.001082
2663	ENSG00000122497	NBPF14	-0.32453	5.393667	0.001089
2664	ENSG00000151729	SLC25A4	0.33795	3.202463	0.00109
2665	ENSG00000149054	ZNF215	1.056206	-0.45015	0.001091
2666	ENSG00000174944	P2RY14	-0.73909	2.383956	0.001091
2667	ENSG00000010610	CD4	-0.65501	7.138896	0.001094
2668	ENSG00000197816	CCDC180	-0.72151	1.132541	0.001096
2669	ENSG00000117226	GBP3	0.455698	5.700709	0.001101
2670	ENSG00000127124	HIVEP3	0.450789	4.838701	0.001101
2671	ENSG00000111224	PARP11	0.281484	4.976397	0.001106
2672	ENSG00000103152	MPG	0.373316	3.327269	0.001111
2673	ENSG00000165490	C11orf82	0.497875	1.954611	0.001116
2674	ENSG00000168785	TSPAN5	0.398301	3.971313	0.001125
2675	ENSG00000153774	CFDP1	0.335099	4.778928	0.001129
2676	ENSG00000233328	PFN1P1	0.474773	5.308318	0.001132
2677	ENSG00000165959	CLMN	-0.59813	4.556616	0.001132
2678	ENSG00000140092	FBLN5	-0.66299	2.015849	0.001132
2679	ENSG00000079150	FKBP7	0.449456	0.273793	0.001134
2680	ENSG00000109452	INPP4B	-0.51374	6.273319	0.001136
2681	ENSG00000211952	IGHV4-28	1.21678	-0.70604	0.001138

2682	ENSG00000118961	C2orf43	0.388246	4.133066	0.00115
2683	ENSG00000181523	SGSH	-0.3291	5.507854	0.001151
2684	ENSG00000232499		0.291082	2.448112	0.001154
2685	ENSG00000139631	CSAD	-0.31903	4.330104	0.001154
2686	ENSG00000129038	LOXL1	-0.75796	-0.75473	0.001154
2687	ENSG00000167720	SRR	0.32251	1.79154	0.001155
2688	ENSG00000204580	DDR1	-0.47763	0.899894	0.001156
2689	ENSG00000211611	IGKV6-21	1.193909	-0.13565	0.001157
2690	ENSG00000251234	PSMA2P2	0.467154	0.663592	0.001158
2691	ENSG00000196876	SCN8A	-0.59514	0.375364	0.001163
2692	ENSG00000155755	TMEM237	0.290014	2.288837	0.001164
2693	ENSG00000100348	TXN2	0.2772	5.523123	0.00117
2694	ENSG00000156162	DPY19L4	-0.34019	3.92181	0.001171
2695	ENSG00000100889	PCK2	0.387501	4.126209	0.001172
2696	ENSG00000113966	ARL6	-0.43041	0.919868	0.001173
2697	ENSG00000104833	TUBB4A	0.509344	1.244881	0.001178
2698	ENSG00000250722	SEPP1	-0.74872	-0.49902	0.001181
2699	ENSG00000103647	CORO2B	-1.19268	-0.78745	0.001182
2700	ENSG00000134153	EMC7	0.247384	5.753338	0.001184
2701	ENSG00000249637		0.604015	-0.80216	0.001184
2702	ENSG00000169957	ZNF768	-0.51543	2.336809	0.001184
2703	ENSG00000130304	SLC27A1	-0.5988	2.893972	0.001189
2704	ENSG00000232369		0.416575	0.790657	0.00119
2705	ENSG00000133193	FAM104A	0.239656	4.866756	0.001191
2706	ENSG00000028839	TBPL1	0.226462	4.933174	0.001191
2707	ENSG00000140025	EFCAB11	0.387325	0.942367	0.001196
2708	ENSG00000138646	HERC5	0.730393	5.476058	0.001197
2709	ENSG00000143409	FAM63A	-0.6591	3.032264	0.001208
2710	ENSG00000138641	HERC3	-0.28859	6.362878	0.001209
2711	ENSG00000114861	FOXP1	-0.31341	7.119184	0.001209
2712	ENSG00000179598	PLD6	-0.4593	1.601566	0.001209
2713	ENSG00000164897	TMUB1	0.345596	4.561323	0.00122
2714	ENSG00000168237	GLYCTK	0.207189	3.808089	0.001221
2715	ENSG00000119698	PPP4R4	-1.02584	-1.4735	0.001223
2716	ENSG00000106588	PSMA2	0.297645	4.21205	0.001223
2717	ENSG00000177076	ACER2	-0.54373	0.397669	0.001226
2718	ENSG00000163840	DTX3L	0.515806	6.636186	0.001227
2719	ENSG00000125434	SLC25A35	0.499405	2.295986	0.001232
2720	ENSG00000082805	ERC1	-0.33682	4.779658	0.001233
2721	ENSG00000204253	HNRNPCP2	0.314635	2.717322	0.001234
2722	ENSG00000213801	ZNF816-ZNF321P	-0.53854	0.51164	0.001235
2723	ENSG00000178115	GOLGA8Q	-0.74242	1.568819	0.001236
2724	ENSG00000132970	WASF3	-0.81418	-0.43717	0.001236
2725	ENSG00000107796	ACTA2	0.554697	1.405536	0.001242

2726	ENSG00000196642	RABL6	0.327365	5.488893	0.001242
2727	ENSG00000129667	RHBDF2	0.317099	6.206581	0.001243
2728	ENSG00000169093	ASMTL	0.41097	3.81546	0.001244
2729	ENSG00000197457	STMN3	-0.44669	4.898275	0.001244
2730	ENSG00000177425	PAWR	-0.50288	2.294742	0.001245
2731	ENSG00000106105	GARS	0.346759	6.099289	0.001246
2732	ENSG00000271141		-0.68471	-0.53773	0.001246
2733	ENSG00000181610	MRPS23	0.292497	4.374606	0.001247
2734	ENSG00000180071	ANKRD18A	-0.7226	-0.77895	0.001248
2735	ENSG00000196305	IARS	0.312773	6.094559	0.001251
2736	ENSG00000159131	GART	0.390664	5.329142	0.001252
2737	ENSG00000170515	PA2G4	0.250708	6.083959	0.001252
2738	ENSG00000118939	UCHL3	0.319976	2.568482	0.001255
2739	ENSG00000112640	PPP2R5D	0.351365	5.075945	0.001256
2740	ENSG00000146386	ABRACL	0.289602	5.974185	0.001259
2741	ENSG00000106789	CORO2A	-0.49351	4.175667	0.001259
2742	ENSG00000133983	COX16	0.311976	4.325182	0.00126
2743	ENSG00000197451	HNRNPAB	0.267302	5.699083	0.001261
2744	ENSG00000163006	CCDC138	0.32504	2.499342	0.001268
2745	ENSG00000117153	KLHL12	0.174055	5.524091	0.001268
2746	ENSG00000102931	ARL2BP	-0.18397	5.687838	0.001268
2747	ENSG00000237706	TRIM51EP	-1.19531	-1.10821	0.001268
2748	ENSG00000148331	ASB6	0.281219	4.689415	0.00127
2749	ENSG00000092931	MFSD11	0.193893	4.594437	0.001273
2750	ENSG00000023516	AKAP11	-0.27283	6.687488	0.001275
2751	ENSG00000268520		0.874678	-0.91056	0.001283
2752	ENSG00000236565	HNRNPA3P5	0.361695	0.596487	0.001286
2753	ENSG00000135953	MFSD9	0.239905	3.783943	0.001287
2754	ENSG00000091129	NRCAM	-0.89396	0.61996	0.001296
2755	ENSG00000107736	CDH23	-0.60537	4.452095	0.0013
2756	ENSG00000186026	ZNF284	-0.43706	1.15758	0.001302
2757	ENSG00000167769	ACER1	-0.77768	-0.88709	0.001305
2758	ENSG00000138430	OLA1	0.20716	5.923622	0.001305
2759	ENSG00000109062	SLC9A3R1	0.349572	6.614393	0.001307
2760	ENSG00000106367	AP1S1	0.644811	3.2628	0.001309
2761	ENSG00000237765	FAM200B	-0.33804	4.305116	0.001309
2762	ENSG00000198618	PPIAP22	0.850876	1.191117	0.001309
2763	ENSG00000188822	CNR2	-0.62544	2.466287	0.001309
2764	ENSG00000236861		-0.66087	-0.72068	0.001309
2765	ENSG00000259030	FPGT-TNNI3K	-0.82257	-0.69029	0.001312
2766	ENSG00000086288	NME8	-0.70827	1.970838	0.001316
2767	ENSG00000215302		-0.39408	3.23157	0.001319
2768	ENSG00000110013	SIAE	-0.50186	2.97089	0.001319
2769	ENSG00000128383	APOBEC3A	0.804516	6.821373	0.00132

2770	ENSG00000103365	GGA2	0.52629	7.80708	0.001324
2771	ENSG00000171611	PTCRA	-0.74636	1.523412	0.001327
2772	ENSG00000126001	CEP250	0.257226	5.288563	0.001327
2773	ENSG00000185513	L3MBTL1	-0.38073	2.901634	0.001328
2774	ENSG00000135900	MRPL44	0.265681	4.974036	0.001328
2775	ENSG00000256069	A2MP1	-0.85497	1.494258	0.001329
2776	ENSG00000197019	SERTAD1	0.590987	5.168489	0.001333
2777	ENSG00000229162		0.684215	-0.74784	0.001334
2778	ENSG00000086475	SEPHS1	0.171335	5.000663	0.001334
2779	ENSG00000129354	AP1M2	-0.88224	-1.08622	0.001335
2780	ENSG00000126822	PLEKHG3	-0.67201	4.789199	0.001339
2781	ENSG00000196782	MAML3	-0.6793	3.347349	0.001339
2782	ENSG00000128789	PSMG2	0.256327	5.979594	0.001344
2783	ENSG00000186814	ZSCAN30	-0.39319	3.954674	0.001347
2784	ENSG00000169239	CA5B	-0.34783	5.109116	0.001349
2785	ENSG00000116337	AMPD2	-0.42677	6.587141	0.001349
2786	ENSG00000012124	CD22	-0.58855	5.412227	0.001349
2787	ENSG00000160285	LSS	0.306763	5.327721	0.001357
2788	ENSG00000019102	VSIG2	-0.76038	1.558694	0.001357
2789	ENSG00000136286	MYO1G	0.262888	8.262488	0.001361
2790	ENSG00000170043	TRAPPC1	0.376749	5.640958	0.001365
2791	ENSG00000260490		0.239976	3.271494	0.001365
2792	ENSG00000157500	APPL1	-0.18075	6.070494	0.001365
2793	ENSG00000225031	EIF4BP7	-0.29113	3.737229	0.001365
2794	ENSG00000253352	TUG1	-0.24476	7.692929	0.001366
2795	ENSG00000047648	ARHGAP6	-0.65415	1.01939	0.001366
2796	ENSG00000236530		0.544098	0.103635	0.001369
2797	ENSG00000261737		0.420068	-0.14579	0.00137
2798	ENSG00000111676	ATN1	-0.4707	4.313055	0.00137
2799	ENSG00000164430	MB21D1	0.406263	4.288835	0.001372
2800	ENSG00000185522	C11orf35	-0.56438	0.516518	0.001384
2801	ENSG00000168803	ADAL	0.370937	1.946701	0.001387
2802	ENSG00000137500	CCDC90B	0.282426	4.971054	0.001388
2803	ENSG00000127445	PIN1	0.308798	5.128623	0.001388
2804	ENSG00000225471		-0.41268	2.64321	0.00139
2805	ENSG00000140464	PML	0.405737	5.591774	0.001392
2806	ENSG00000125356	NDUFA1	0.319416	6.040735	0.001394
2807	ENSG00000164024	METAP1	0.137957	5.678316	0.00141
2808	ENSG00000108424	KPNB1	0.236212	7.65203	0.001415
2809	ENSG00000121895	TMEM156	0.398853	4.307701	0.001415
2810	ENSG00000261739	GOLGA8S	-0.61078	0.138011	0.00142
2811	ENSG00000050628	PTGER3	-1.04778	1.099516	0.00142
2812	ENSG00000213071	LPAL2	-0.53508	1.487523	0.001423
2813	ENSG00000203836	NBPF24	-0.23031	5.504946	0.001427

2814	ENSG00000137055	PLAA	0.281704	5.512435	0.001428
2815	ENSG00000154102	C16orf74	-0.52816	1.782777	0.001433
2816	ENSG00000233695	GAS6-AS1	-0.91877	-0.16642	0.00144
2817	ENSG00000049323	LTBP1	-0.7788	2.001777	0.001444
2818	ENSG00000162913	C1orf145	-0.6784	-0.10725	0.001444
2819	ENSG00000219951		0.546005	-0.12025	0.001447
2820	ENSG00000147099	HDAC8	0.206448	3.715763	0.001447
2821	ENSG00000175787	ZNF169	-0.30211	3.012558	0.001447
2822	ENSG00000272949		-0.50132	0.706385	0.001447
2823	ENSG00000172673	THEMIS	0.562428	6.477915	0.001447
2824	ENSG00000023839	ABCC2	-0.36504	1.691389	0.001447
2825	ENSG00000159618	GPR114	0.546692	4.658169	0.001453
2826	ENSG00000023228	NDUFS1	0.186799	6.098966	0.001453
2827	ENSG00000197951	ZNF71	-0.39249	2.951449	0.001454
2828	ENSG00000185761	ADAMTSL5	-0.57522	0.542698	0.001462
2829	ENSG00000087258	GNAO1	-0.68579	1.826023	0.001469
2830	ENSG00000147443	DOK2	0.323276	6.684308	0.001471
2831	ENSG00000090263	MRPS33	0.304412	4.10552	0.001472
2832	ENSG00000197860	SGTB	-0.36154	6.036473	0.001472
2833	ENSG00000241163	LINC00877	-0.4829	3.845625	0.001472
2834	ENSG00000164902	PHAX	0.350289	5.046698	0.001474
2835	ENSG00000127511	SIN3B	-0.28107	5.421721	0.001476
2836	ENSG00000162496	DHRS3	-0.53238	3.989733	0.00148
2837	ENSG00000183691	NOG	-1.20093	0.314819	0.00148
2838	ENSG00000183941	HIST2H4A	0.484959	2.493812	0.001481
2839	ENSG00000115685	PPP1R7	0.263555	5.718287	0.001484
2840	ENSG00000272666		0.758416	0.529496	0.001484
2841	ENSG00000214140	PRCD	-0.71886	-1.06888	0.001484
2842	ENSG00000124491	F13A1	-0.77095	6.823977	0.001485
2843	ENSG00000171045	TSNARE1	-0.31767	3.498053	0.001489
2844	ENSG00000180817	PPA1	0.275019	6.38971	0.001492
2845	ENSG00000205581	HMGN1	0.229464	7.704259	0.001493
2846	ENSG00000105641	SLC5A5	-0.63289	-0.90105	0.001493
2847	ENSG00000257698		0.434282	1.192468	0.001496
2848	ENSG00000089289	IGBP1	0.181794	6.68019	0.001505
2849	ENSG00000139131	YARS2	0.220512	3.677654	0.00151
2850	ENSG00000130413	STK33	0.665669	-0.47341	0.001511
2851	ENSG00000221955	SLC12A8	0.888502	-0.72004	0.001523
2852	ENSG00000257337		-0.44955	1.748982	0.001526
2853	ENSG00000065911	MTHFD2	0.500102	6.309391	0.001527
2854	ENSG00000132640	BTBD3	-0.4912	3.032443	0.001532
2855	ENSG00000185101	ANO9	-0.44405	4.944232	0.001533
2856	ENSG00000185986	SDHAP3	0.180716	3.524464	0.001536
2857	ENSG00000213462	ERV3-1	-0.44061	3.508065	0.001544

2858	ENSG00000260335		0.404118	5.246915	0.001548
2859	ENSG00000213337	ANKRD39	0.264849	3.044082	0.001549
2860	ENSG00000205837	LINC00487	1.167765	-0.68521	0.00155
2861	ENSG00000248175		-0.36932	3.622681	0.001553
2862	ENSG00000149503	INCENP	0.368102	3.320133	0.001557
2863	ENSG00000156508	EEF1A1	-0.24807	14.07285	0.001557
2864	ENSG00000166902	MRPL16	0.226829	4.587213	0.001559
2865	ENSG00000244052	RPL5P24	-0.28946	6.021358	0.00156
2866	ENSG00000105053	VRK3	0.254457	5.195683	0.00156
2867	ENSG00000109919	MTCH2	0.267995	5.307971	0.001564
2868	ENSG00000119943	PYROXD2	-0.50333	2.881486	0.001571
2869	ENSG00000168461	RAB31	-0.45477	6.946139	0.001574
2870	ENSG00000123119	NECAB1	-0.95439	-0.71385	0.001579
2871	ENSG00000111145	ELK3	-0.25917	5.956967	0.001582
2872	ENSG00000100368	CSF2RB	-0.50628	6.872576	0.001586
2873	ENSG00000130402	ACTN4	0.451313	7.118887	0.001586
2874	ENSG00000231105		-0.57478	0.459209	0.001586
2875	ENSG00000066583	ISOC1	0.275686	4.047874	0.001593
2876	ENSG00000081059	TCF7	-0.48115	7.754285	0.001597
2877	ENSG00000125779	PANK2	0.259923	5.333743	0.001603
2878	ENSG00000169087	HSPBAP1	-0.28265	4.651589	0.001604
2879	ENSG00000116670	MAD2L2	0.32192	4.607939	0.001612
2880	ENSG00000230280	HNRNPA1P59	0.503657	-0.57935	0.001614
2881	ENSG00000128641	MYO1B	-0.78685	-0.72724	0.001614
2882	ENSG00000161911	TREML1	-0.88173	3.852817	0.001614
2883	ENSG00000139610	CELA1	-0.89274	-1.22559	0.001616
2884	ENSG00000223551	TMSB4XP4	0.392772	7.115611	0.001619
2885	ENSG00000203710	CR1	-0.71802	5.131774	0.001623
2886	ENSG00000162694	EXTL2	0.375102	2.618578	0.001623
2887	ENSG00000049769	PPP1R3F	-0.43842	1.791683	0.001627
2888	ENSG00000133028	SCO1	0.241255	3.977601	0.001628
2889	ENSG00000234797	RPS3AP6	-0.34941	5.589151	0.001628
2890	ENSG00000227709		0.561594	-0.78355	0.001636
2891	ENSG00000163995	ABLIM2	-0.50515	-0.0974	0.001636
2892	ENSG00000111666	CHPT1	-0.45956	4.845313	0.001636
2893	ENSG00000186399	GOLGA8R	-0.71435	1.851477	0.001636
2894	ENSG00000241399	CD302	-0.78596	4.01757	0.001636
2895	ENSG00000186871	ERCC6L	0.524375	1.282619	0.001637
2896	ENSG00000122643	NT5C3A	0.481759	6.244533	0.001644
2897	ENSG00000176946	THAP4	0.309484	4.775044	0.001647
2898	ENSG00000251432		-0.5853	-0.34871	0.00165
2899	ENSG00000213144		0.313155	1.849771	0.001652
2900	ENSG00000250155		-0.61265	0.401945	0.001659
2901	ENSG00000072415	MPP5	-0.27777	4.183077	0.001665

2902	ENSG00000232267	ACTR3P2	0.456022	1.437958	0.001665
2903	ENSG00000138443	ABI2	-0.29733	4.840689	0.001667
2904	ENSG00000175727	MLXIP	-0.30755	6.397944	0.001667
2905	ENSG00000236304		-0.81576	3.839108	0.001668
2906	ENSG00000233306	TRGV2	0.593283	2.583996	0.001668
2907	ENSG00000141295	SCRN2	0.324248	4.261955	0.001678
2908	ENSG00000170954	ZNF415	-0.73704	-0.46527	0.001681
2909	ENSG00000100228	RAB36	-0.79191	-0.92138	0.001681
2910	ENSG00000102879	CORO1A	0.328148	9.626952	0.001683
2911	ENSG00000135318	NT5E	-0.68268	2.619591	0.001691
2912	ENSG00000242866	STRC	-0.65831	-0.57373	0.001691
2913	ENSG00000104825	NFKBIB	0.421579	5.046962	0.001692
2914	ENSG00000107863	ARHGAP21	-0.28053	5.828804	0.001693
2915	ENSG00000108100	CCNY	-0.24532	7.242378	0.001694
2916	ENSG00000143321	HDGF	0.276298	7.351103	0.001695
2917	ENSG00000170006	TMEM154	-0.39525	6.3439	0.001698
2918	ENSG00000269911	FAM226A	-0.55829	0.158376	0.001702
2919	ENSG00000173239	LIPM	1.427924	-1.92523	0.001702
2920	ENSG00000004864	SLC25A13	-0.25244	3.773014	0.001709
2921	ENSG00000198960	ARMCX6	0.224574	4.863351	0.00171
2922	ENSG00000159176	CSRP1	0.269328	5.884024	0.001715
2923	ENSG00000166226	CCT2	0.307838	6.958123	0.001723
2924	ENSG00000171132	PRKCE	-0.26047	4.258981	0.001728
2925	ENSG00000236753	MKLN1-AS	-0.45763	-0.03198	0.001728
2926	ENSG00000115504	EHBP1	-0.34306	3.964091	0.001732
2927	ENSG00000118922	KLF12	-0.33628	7.624647	0.001738
2928	ENSG00000108846	ABCC3	-0.67135	3.988706	0.001738
2929	ENSG00000053372	MRTO4	0.297481	4.117362	0.001739
2930	ENSG00000131508	UBE2D2	0.188572	6.728371	0.001739
2931	ENSG00000197476		1.19116	-1.25312	0.001739
2932	ENSG00000246705	H2AFJ	0.383523	4.238653	0.00174
2933	ENSG00000205413	SAMD9	0.611727	7.478444	0.001742
2934	ENSG00000154188	ANGPT1	-0.78279	-0.10197	0.001742
2935	ENSG00000129932	DOHH	0.454131	1.640911	0.001746
2936	ENSG00000114993	RTKN	0.594786	-0.32847	0.001757
2937	ENSG00000172197	MBOAT1	-0.38606	3.655595	0.001759
2938	ENSG00000188290	HES4	0.79506	1.892804	0.001763
2939	ENSG00000117118	SDHB	0.195124	6.155291	0.001768
2940	ENSG00000259472		-0.54174	0.267406	0.001768
2941	ENSG00000167508	MVD	0.416185	5.124685	0.001771
2942	ENSG00000170315	UBB	0.404005	8.19459	0.001773
2943	ENSG00000247746	USP51	-0.50813	0.431278	0.001776
2944	ENSG00000203819	HIST2H2BC	0.558883	-0.6203	0.001779
2945	ENSG00000167210	LOXHD1	-0.89472	0.822244	0.001783

2946	ENSG00000182217	HIST2H4B	0.494133	2.502723	0.001784
2947	ENSG00000111348	ARHGDIB	0.245333	10.13677	0.001789
2948	ENSG00000125826	RBCK1	0.405332	6.140731	0.00179
2949	ENSG00000237438	CECR7	-0.52947	0.529569	0.001791
2950	ENSG00000196644	GPR89C	0.2227	3.827608	0.001793
2951	ENSG00000163655	GMPS	0.200692	5.679111	0.001794
2952	ENSG00000157259	GATAD1	-0.29263	4.820848	0.001795
2953	ENSG00000124795	DEK	0.257038	7.849877	0.001795
2954	ENSG00000079462	PAFAH1B3	0.425465	2.872515	0.001797
2955	ENSG00000270441		-0.65027	0.13258	0.001807
2956	ENSG00000149480	MTA2	0.223988	6.881035	0.001808
2957	ENSG00000179271	GADD45GIP1	0.323066	4.149656	0.001809
2958	ENSG00000248309	MEF2C-AS1	-0.51906	0.083714	0.001813
2959	ENSG00000261996		-0.79159	-0.25482	0.001813
2960	ENSG00000224650	IGHV3-74	1.114137	1.235237	0.001815
2961	ENSG00000227973	PIN4P1	0.550488	-0.31649	0.001815
2962	ENSG00000188375	H3F3C	0.551471	3.549346	0.001816
2963	ENSG00000261372		-0.59986	-0.24573	0.001816
2964	ENSG00000035928	RFC1	0.285015	6.368891	0.001816
2965	ENSG00000259699	HMGB1P8	0.474896	0.340697	0.001819
2966	ENSG00000135899	SP110	0.408684	6.781593	0.001819
2967	ENSG00000197217	ENTPD4	-0.25559	7.115362	0.001819
2968	ENSG00000183558	HIST2H2AA3	0.556879	2.984558	0.001822
2969	ENSG00000163864	NMNAT3	-0.50876	0.753971	0.001824
2970	ENSG00000258572		0.725676	-0.40931	0.001832
2971	ENSG00000214226	C17orf67	-0.48322	1.202503	0.001832
2972	ENSG00000186603	HPDL	0.667979	-0.65521	0.001836
2973	ENSG00000166575	TMEM135	0.320711	4.209578	0.001836
2974	ENSG00000228463		-0.45678	0.661041	0.001838
2975	ENSG00000198682	PAPSS2	-0.56477	3.788131	0.001838
2976	ENSG00000068912	ERLEC1	0.265247	5.917406	0.001842
2977	ENSG00000054356	PTPRN	0.877321	-1.10852	0.001843
2978	ENSG00000104824	HNRNPL	0.218993	7.719853	0.00185
2979	ENSG00000205930	C21orf49	-0.755	-0.17725	0.001853
2980	ENSG00000123992	DNPEP	0.183729	5.585039	0.001857
2981	ENSG00000143842	SOX13	-0.55242	2.394075	0.00186
2982	ENSG00000145428	RNF175	-0.48392	2.552688	0.00186
2983	ENSG00000164292	RHOBTB3	-0.49294	2.461652	0.001861
2984	ENSG00000174749	C4orf32	-0.36962	3.945672	0.001863
2985	ENSG00000247134		-0.5519	0.917786	0.001865
2986	ENSG00000242193		-0.75328	1.641453	0.001865
2987	ENSG00000179295	PTPN11	0.215364	6.955122	0.001871
2988	ENSG00000163235	TGFA	-0.65901	2.217883	0.001878
2989	ENSG00000267364		0.549511	2.221686	0.001879

2990	ENSG00000172878	METAP1D	-0.41061	2.217628	0.001879
2991	ENSG00000040275	SPDL1	0.394341	3.031343	0.001881
2992	ENSG00000106400	ZNHIT1	0.23999	5.31174	0.001885
2993	ENSG00000214029	ZNF891	-0.48048	2.188159	0.001885
2994	ENSG00000090971	NAT14	0.447007	1.479836	0.001888
2995	ENSG00000126067	PSMB2	0.362347	6.30955	0.00189
2996	ENSG00000151575	TEX9	-0.57751	-0.0928	0.001895
2997	ENSG00000211789	TRAV12-2	0.591673	2.173967	0.001895
2998	ENSG00000205531	NAP1L4	0.191836	7.300494	0.001903
2999	ENSG00000089335	ZNF302	-0.35777	4.632993	0.00191
3000	ENSG00000147586	MRPS28	0.310179	3.559771	0.001922
3001	ENSG00000075223	SEMA3C	-0.57302	2.819665	0.001922
3002	ENSG00000060688	SNRNP40	0.266269	4.840531	0.001926
3003	ENSG00000167232	ZNF91	-0.38232	4.759727	0.001929
3004	ENSG00000160877	NACC1	0.400522	4.7537	0.001929
3005	ENSG00000164967	RPP25L	0.359985	2.818106	0.001931
3006	ENSG00000101236	RNF24	-0.39753	5.407463	0.001936
3007	ENSG00000141027	NCOR1	0.293636	7.185186	0.00194
3008	ENSG00000267508	ZNF285	-0.86729	-0.4431	0.00194
3009	ENSG00000013725	CD6	0.451462	7.851972	0.001943
3010	ENSG00000104679	R3HCC1	0.253087	4.381519	0.001944
3011	ENSG00000170396	ZNF804A	-0.62372	0.712383	0.001947
3012	ENSG00000267128	RNF157-AS1	-0.61606	0.465809	0.001948
3013	ENSG00000156709	AIFM1	0.326139	4.483295	0.00195
3014	ENSG00000120708	TGFBI	-0.65887	7.185119	0.001951
3015	ENSG00000166822	TMEM170A	0.243682	6.048913	0.00196
3016	ENSG00000175581	MRPL48	0.340748	4.216698	0.001961
3017	ENSG00000163584	RPL22L1	0.314033	5.188087	0.001962
3018	ENSG00000091640	SPAG7	0.201011	5.432334	0.001964
3019	ENSG00000131069	ACSS2	-0.55087	3.994684	0.001965
3020	ENSG00000138785	INTS12	0.241165	4.484879	0.001974
3021	ENSG00000230067	HSPD1P6	0.441953	1.921874	0.001975
3022	ENSG00000103037	SETD6	-0.30939	3.332066	0.001984
3023	ENSG00000246263		-0.28427	3.18359	0.00199
3024	ENSG00000164221	CCDC112	-0.54587	3.089508	0.001997
3025	ENSG00000050730	TNIP3	0.988907	1.959712	0.002002
3026	ENSG00000167283	ATP5L	0.257789	7.504926	0.002012
3027	ENSG00000271856		-0.41161	4.937623	0.002012
3028	ENSG00000130147	SH3BP4	-0.64334	1.279005	0.002012
3029	ENSG00000250132		-0.67446	-0.87043	0.002012
3030	ENSG00000266296	ARIH2P1	0.349664	1.146331	0.002012
3031	ENSG00000258405	ZNF578	-0.55755	-0.23683	0.002019
3032	ENSG00000213445	SIPA1	0.345822	7.10695	0.002021
3033	ENSG00000187735	TCEA1	0.174119	7.444245	0.002023

3034	ENSG00000251595	ABCA11P	-0.69855	-0.80248	0.002026
3035	ENSG00000168970	JMJD7-PLA2G4B	-0.46678	3.361042	0.002029
3036	ENSG00000238105	GOLGA2P5	-0.40463	4.520614	0.002033
3037	ENSG00000187260	WDR86	-0.42042	1.01361	0.00204
3038	ENSG00000257242	C12orf79	-0.44414	3.976205	0.00204
3039	ENSG00000102445	KIAA0226L	-0.60185	4.975193	0.00204
3040	ENSG00000174886	NDUFA11	0.248192	4.777113	0.002045
3041	ENSG00000233280		-0.8242	1.8221	0.002045
3042	ENSG00000123454	DBH	-0.97661	-1.01584	0.002052
3043	ENSG00000169727	GPS1	0.268106	5.657815	0.002052
3044	ENSG00000138758	SEPT11	0.212756	5.685414	0.002053
3045	ENSG00000112182	BACH2	-0.58217	5.726362	0.002053
3046	ENSG00000258900	HNRNPCP1	0.319487	2.394176	0.002054
3047	ENSG00000168939	SPRY3	-0.37387	0.961348	0.002054
3048	ENSG00000131375	CAPN7	-0.23134	6.090551	0.002058
3049	ENSG00000214279		-0.55907	2.39432	0.002058
3050	ENSG00000186665	C17orf58	0.41228	1.347708	0.002066
3051	ENSG00000065978	YBX1	0.21328	9.018847	0.002085
3052	ENSG00000260296		-0.51455	0.822952	0.002089
3053	ENSG00000103479	RBL2	-0.31012	8.31566	0.002098
3054	ENSG00000087299	L2HGDH	0.309718	1.460719	0.002105
3055	ENSG00000128059	PPAT	0.286807	3.619187	0.002105
3056	ENSG00000156171	DRAM2	-0.29059	6.231749	0.002105
3057	ENSG00000073578	SDHA	0.246005	6.129906	0.002108
3058	ENSG00000141447	OSBPL1A	-0.69183	2.616975	0.002108
3059	ENSG00000114107	CEP70	-0.32143	2.654451	0.002116
3060	ENSG00000143507	DUSP10	0.723385	5.167765	0.002126
3061	ENSG00000111452	GPR133	-0.91427	0.686099	0.002128
3062	ENSG00000140350	ANP32A	0.256937	7.213835	0.002136
3063	ENSG00000187109	NAP1L1	-0.22221	9.453563	0.002141
3064	ENSG00000183134	PTGDR2	-0.76545	1.132826	0.002146
3065	ENSG00000149136	SSRP1	0.292239	6.395826	0.002148
3066	ENSG00000114956	DGUOK	0.198175	5.141446	0.002148
3067	ENSG00000177590		-0.83869	-1.26244	0.002148
3068	ENSG00000166710	B2M	0.258934	13.56167	0.002159
3069	ENSG00000216624	GAPDHP72	0.374174	2.492714	0.00216
3070	ENSG00000111801	BTN3A3	0.342653	7.308298	0.002162
3071	ENSG00000088812	ATRN	-0.28467	5.183159	0.002163
3072	ENSG00000107731	UNC5B	0.864769	-1.06776	0.002164
3073	ENSG00000102870	ZNF629	-0.53878	1.212047	0.002164
3074	ENSG00000142173	COL6A2	-0.5818	4.42087	0.002166
3075	ENSG00000197142	ACSL5	0.316933	6.66188	0.002171
3076	ENSG00000169093	ASMTL	0.395775	3.801999	0.00218
3077	ENSG00000267554		0.388907	3.69686	0.002185

3078	ENSG00000229200	TRBV7-8	0.60002	1.911376	0.002193
3079	ENSG00000127554	GFER	0.247835	3.481913	0.002196
3080	ENSG00000180376	CCDC66	-0.35454	5.0635	0.002196
3081	ENSG00000113555	PCDH12	-0.41265	1.832997	0.002196
3082	ENSG00000164120	HPGD	-0.51722	3.589668	0.002198
3083	ENSG00000034677	RNF19A	0.530195	8.012954	0.002205
3084	ENSG00000182325	FBXL6	0.262586	3.412521	0.002217
3085	ENSG00000119705	SLIRP	0.341376	4.066142	0.002225
3086	ENSG00000143183	TMCO1	0.257172	6.139935	0.002225
3087	ENSG00000248333	CDK11B	0.241894	5.880243	0.002226
3088	ENSG00000117643	MAN1C1	-0.44951	3.74562	0.002226
3089	ENSG00000198520	C1orf228	-0.38361	3.963028	0.002234
3090	ENSG00000166405	RIC3	-0.44133	3.957546	0.002235
3091	ENSG00000131724	IL13RA1	-0.5122	5.735351	0.002246
3092	ENSG00000181061	HIGD1A	0.268416	5.994167	0.002249
3093	ENSG00000162813	BPNT1	0.482893	4.037625	0.002261
3094	ENSG00000218582	GAPDHP63	0.411323	0.959337	0.002261
3095	ENSG00000149196	C11orf73	0.35467	4.54624	0.002262
3096	ENSG00000198205	ZXDA	-0.2528	3.859406	0.002262
3097	ENSG00000198276	UCKL1	0.178991	4.846425	0.002272
3098	ENSG00000248923	MTND5P11	-0.40375	6.363258	0.002272
3099	ENSG00000111215	PRR4	-0.34062	2.224448	0.002281
3100	ENSG00000214362		-0.36176	3.146368	0.002289
3101	ENSG00000125775	SDCBP2	0.454838	0.232926	0.002294
3102	ENSG00000204209	DAXX	0.343277	5.539771	0.002295
3103	ENSG00000180902	D2HGDH	0.340754	4.012563	0.002308
3104	ENSG00000186105	LRRC70	-0.60246	0.46802	0.002311
3105	ENSG00000115602	IL1RL1	-0.82269	1.581408	0.002315
3106	ENSG00000268895	A1BG-AS1	-0.44001	0.439169	0.00232
3107	ENSG00000103061	SLC7A6OS	0.254392	2.795903	0.002322
3108	ENSG00000239272	RPL21P10	-0.36405	1.123357	0.002324
3109	ENSG00000243746	EEF1A1P10	-0.28483	6.657969	0.002338
3110	ENSG00000135940	COX5B	0.321799	6.242106	0.002355
3111	ENSG00000160710	ADAR	0.319193	8.980454	0.002365
3112	ENSG00000100949	RABGGTA	0.211573	4.835103	0.002365
3113	ENSG00000066294	CD84	0.375045	7.183725	0.002367
3114	ENSG00000171310	CHST11	0.277569	6.465432	0.002367
3115	ENSG00000267278	MAP3K14-AS1	-0.36383	2.034231	0.002374
3116	ENSG00000175294	CATSPER1	-0.6479	1.712863	0.002375
3117	ENSG00000101773	RBBP8	0.460122	5.123536	0.002378
3118	ENSG00000197223	C1D	0.277931	4.711771	0.002386
3119	ENSG00000198265	HELZ	0.195594	7.434531	0.002388
3120	ENSG00000138614	VWA9	0.252822	5.072935	0.0024
3121	ENSG00000185127	C6orf120	-0.24476	5.57587	0.002405

3122	ENSG00000100319	ZMAT5	0.343711	2.651605	0.002406
3123	ENSG00000146007	ZMAT2	0.238613	6.653537	0.002406
3124	ENSG00000247679		-0.35222	1.718459	0.002406
3125	ENSG00000132207	SLX1A	0.356307	2.242831	0.002413
3126	ENSG00000186577	C6orf1	0.306734	3.789961	0.002429
3127	ENSG00000160799	CCDC12	0.312221	5.434152	0.002429
3128	ENSG00000186017	ZNF566	-0.38633	2.957738	0.002429
3129	ENSG00000198752	CDC42BPB	-0.52843	3.252408	0.002436
3130	ENSG00000113407	TARS	0.467767	6.078666	0.002439
3131	ENSG00000136877	FPGS	0.264078	5.146264	0.002451
3132	ENSG00000163050	ADCK3	-0.21856	5.237791	0.002452
3133	ENSG00000166402	TUB	-0.61905	1.058702	0.002458
3134	ENSG00000163634	THOC7	0.277562	5.02701	0.002493
3135	ENSG00000163029	SMC6	0.309142	5.873529	0.002499
3136	ENSG00000068615	REEP1	-1.04776	-0.90382	0.002506
3137	ENSG00000121848	RNF115	0.259844	6.053923	0.002509
3138	ENSG00000119862	LGALSL	-0.66782	2.064797	0.00251
3139	ENSG00000186318	BACE1	-0.61306	2.271322	0.002523
3140	ENSG00000095464	PDE6C	-0.67497	-0.21195	0.002526
3141	ENSG00000123360	PDE1B	0.385199	3.734974	0.002531
3142	ENSG00000182685	BRICD5	-0.47667	1.186726	0.002531
3143	ENSG00000025796	SEC63	-0.14711	6.611533	0.002539
3144	ENSG00000003249	DBNDD1	-0.97431	-0.72522	0.00254
3145	ENSG00000169599	NFU1	0.287825	4.413837	0.00254
3146	ENSG00000163882	POLR2H	0.27516	4.210952	0.002542
3147	ENSG00000241679		-0.52429	-0.32961	0.002544
3148	ENSG00000182158	CREB3L2	-0.30379	5.061251	0.00255
3149	ENSG00000256269	HMBS	0.502237	3.231162	0.002553
3150	ENSG00000067955	CBFB	0.200082	6.653366	0.002566
3151	ENSG00000211788	TRAV13-1	0.511366	2.081619	0.002567
3152	ENSG00000172006	ZNF554	-0.33023	1.830998	0.002568
3153	ENSG00000048544	MRPS10	0.259089	5.234962	0.00257
3154	ENSG00000266053		-0.36535	2.017398	0.002573
3155	ENSG00000181741	FDX1P1	0.276346	1.742457	0.002575
3156	ENSG00000103429	BFAR	0.255052	5.969254	0.00258
3157	ENSG00000184897	H1FX	0.685217	5.28598	0.002582
3158	ENSG00000178163	ZNF518B	-0.2278	5.289971	0.002582
3159	ENSG00000185721	DRG1	0.200606	5.393688	0.002585
3160	ENSG00000163781	TOPBP1	0.239347	6.729749	0.002588
3161	ENSG00000119812	FAM98A	0.286461	4.503108	0.002597
3162	ENSG00000213860	RPL21P75	-0.32044	6.546459	0.002603
3163	ENSG00000196466	ZNF799	-0.34288	2.315594	0.002604
3164	ENSG00000163386	NBPF10	-0.29098	6.269058	0.002608
3165	ENSG00000145050	MANF	0.411625	5.284561	0.00261

3166	ENSG00000204934	ATP6V0E2-AS1	-0.72256	0.205711	0.00261
3167	ENSG00000163053	SLC16A14	0.7579	-0.80486	0.002613
3168	ENSG00000183814	LIN9	0.413517	2.285612	0.002613
3169	ENSG00000099203	TMED1	0.323279	2.179412	0.002613
3170	ENSG00000224597	PTCHD3P1	-0.34977	2.8925	0.002624
3171	ENSG00000150760	DOCK1	-0.74672	-0.30494	0.00263
3172	ENSG00000141096	DPEP3	-0.59799	-0.15691	0.002637
3173	ENSG00000115526	CHST10	0.462389	2.77227	0.00264
3174	ENSG00000101464	PIGU	0.292032	3.265156	0.002647
3175	ENSG00000139278	GLIPR1	-0.2502	7.721026	0.002649
3176	ENSG00000271180		0.466818	0.136758	0.002651
3177	ENSG00000101473	ACOT8	0.226622	3.788373	0.002652
3178	ENSG00000167766	ZNF83	-0.41213	5.628048	0.002655
3179	ENSG00000092094	OSGEP	-0.19872	5.533191	0.002659
3180	ENSG00000092098	RNF31	0.301936	4.502394	0.00266
3181	ENSG00000232653	GOLGA8N	-0.70776	1.715034	0.002668
3182	ENSG00000158373	HIST1H2BD	0.533996	1.887591	0.002672
3183	ENSG00000188313	PLSCR1	0.578088	6.529941	0.00268
3184	ENSG00000221946	FXYD7	-0.56705	0.910831	0.00268
3185	ENSG00000216895		0.340558	1.29461	0.002681
3186	ENSG00000169247	SH3TC2	-0.8272	-0.09193	0.002693
3187	ENSG00000232472	EEF1B2P3	-0.32378	4.789364	0.002702
3188	ENSG00000173597	SULT1B1	-0.7347	4.291498	0.002709
3189	ENSG00000231621		-0.748	0.851056	0.002719
3190	ENSG00000176681	LRRC37A	-0.27668	3.77345	0.002736
3191	ENSG00000261512		-0.36144	4.441297	0.002742
3192	ENSG00000258427	RBM8B	0.311202	4.322695	0.002757
3193	ENSG00000185946	RNPC3	-0.30116	5.186724	0.002757
3194	ENSG00000175606	TMEM70	0.33631	4.286507	0.002762
3195	ENSG00000224429		0.418707	1.479633	0.002762
3196	ENSG00000142686	C1orf216	0.322988	3.642965	0.002777
3197	ENSG00000188384	CSPG4P8	-0.55793	-0.59517	0.002778
3198	ENSG00000005194	CIAPIN1	0.189028	4.930441	0.002799
3199	ENSG00000185379	RAD51D	0.294986	2.63537	0.0028
3200	ENSG00000167291	TBC1D16	-0.48431	1.350715	0.0028
3201	ENSG00000145247	OCIAD2	0.361037	5.03565	0.002811
3202	ENSG00000034510	TMSB10	0.328373	10.04924	0.002811
3203	ENSG00000145882	PCYOX1L	-0.3613	4.543729	0.002817
3204	ENSG00000185245	GP1BA	-0.53027	1.94636	0.002817
3205	ENSG00000246859	STARD4-AS1	-0.60228	0.410805	0.002844
3206	ENSG00000133812	SBF2	-0.37731	4.89353	0.00285
3207	ENSG00000134996	OSTF1	0.293484	6.649693	0.002852
3208	ENSG00000164054	SHISA5	0.420264	7.365189	0.002856
3209	ENSG00000181625	SLX1B	0.294188	2.956475	0.002857

3210	ENSG00000213539	YBX1P6	0.220669	4.192104	0.002857
3211	ENSG00000116791	CRYZ	0.351399	4.666853	0.002867
3212	ENSG00000173566	NUDT18	0.415198	3.226192	0.002881
3213	ENSG00000112651	MRPL2	0.204598	3.495394	0.002881
3214	ENSG00000105784	RUNDC3B	-0.89514	-1.07558	0.002886
3215	ENSG00000253234	IGLV2-5	0.877937	-0.81251	0.002894
3216	ENSG00000163001	CCDC104	-0.31302	4.436358	0.002901
3217	ENSG00000132541	HRSP12	0.503506	2.555212	0.002915
3218	ENSG00000118507	AKAP7	-0.3799	3.506467	0.002915
3219	ENSG00000072501	SMC1A	0.246935	6.80261	0.002924
3220	ENSG00000206127	GOLGA8O	-0.66801	1.965423	0.002928
3221	ENSG00000130303	BST2	0.556457	6.304668	0.002933
3222	ENSG00000095002	MSH2	0.387784	4.597889	0.002933
3223	ENSG00000007237	GAS7	-0.49555	6.750113	0.002933
3224	ENSG00000241560	ZBTB20-AS1	0.632081	0.128897	0.002938
3225	ENSG00000238083	LRRC37A2	-0.26894	4.046749	0.002938
3226	ENSG00000259483		-0.93832	-1.19907	0.002944
3227	ENSG00000112701	SENP6	-0.19953	6.860007	0.002949
3228	ENSG00000111144	LTA4H	-0.50872	7.716746	0.002973
3229	ENSG00000261834	IGHV3OR16-15	1.102033	-0.97945	0.002977
3230	ENSG00000123064	DDX54	0.271874	5.467213	0.002977
3231	ENSG00000105341	ATP5SL	0.239778	4.77065	0.002983
3232	ENSG00000183066	WBP2NL	-0.56662	-0.28928	0.002983
3233	ENSG00000255624		-0.52288	0.263867	0.002984
3234	ENSG00000163735	CXCL5	-0.86427	3.701324	0.002993
3235	ENSG00000172250	SERHL	-0.49577	0.590939	0.002994
3236	ENSG00000090104	RGS1	0.850884	8.490843	0.002996
3237	ENSG00000108799	EZH1	-0.19133	6.232309	0.002996
3238	ENSG00000254429		-0.63693	2.579566	0.002996
3239	ENSG00000119383	PPP2R4	0.390059	5.832488	0.00301
3240	ENSG00000141293	SKAP1	0.351298	6.203585	0.003019
3241	ENSG00000267169		-0.40428	1.919434	0.003034
3242	ENSG00000176986	SEC24C	0.209902	7.121122	0.003043
3243	ENSG00000187554	TLR5	-0.81021	3.419112	0.003043
3244	ENSG00000166452	AKIP1	0.374188	2.657842	0.003049
3245	ENSG00000154608	CEP170P1	-0.43709	3.701222	0.003049
3246	ENSG00000104427	ZC2HC1A	-0.46161	2.289663	0.003052
3247	ENSG00000137269	LRRC1	-0.47757	0.807737	0.003052
3248	ENSG00000205056		-0.77204	1.040656	0.003058
3249	ENSG00000152778	IFIT5	0.477911	5.750365	0.003062
3250	ENSG00000128218	VPREB3	-0.83025	1.006189	0.003064
3251	ENSG00000127990	SGCE	-0.68468	0.098364	0.003073
3252	ENSG00000162086	ZNF75A	-0.2832	4.491025	0.003076
3253	ENSG00000134245	WNT2B	-0.5259	0.327515	0.003076

3254	ENSG00000041353	RAB27B	-0.47845	4.271283	0.003077
3255	ENSG00000075275	CELSR1	-0.45515	2.188609	0.003078
3256	ENSG00000004700	RECQL	0.315781	6.06081	0.003089
3257	ENSG00000002549	LAP3	0.580673	7.199715	0.003093
3258	ENSG00000090924	PLEKHG2	0.388804	5.153132	0.003094
3259	ENSG00000169813	HNRNPF	0.317952	7.64859	0.003107
3260	ENSG00000215447		-0.49957	0.861392	0.003119
3261	ENSG00000095906	NUBP2	0.279826	4.591328	0.00312
3262	ENSG00000214646		0.427545	0.252806	0.003122
3263	ENSG00000136478	TEX2	-0.26153	4.235384	0.003124
3264	ENSG00000196511	TPK1	-0.34977	4.102362	0.003126
3265	ENSG00000269640		0.494697	3.768531	0.003127
3266	ENSG00000211821	TRDV2	-0.9925	2.002838	0.003128
3267	ENSG00000198171	DDRGK1	0.219158	4.613635	0.003136
3268	ENSG00000090520	DNAJB11	0.268405	6.67774	0.003141
3269	ENSG00000106617	PRKAG2	-0.22664	5.615152	0.003141
3270	ENSG00000132196	HSD17B7	0.295496	3.54014	0.003152
3271	ENSG00000110492	MDK	0.51867	0.756824	0.003154
3272	ENSG00000171793	CTPS1	0.711216	4.962444	0.003154
3273	ENSG00000141510	TP53	0.323055	4.81115	0.003157
3274	ENSG00000229638	RPL4P4	-0.26677	5.826394	0.003157
3275	ENSG00000211900	IGHJ6	0.990622	-0.06847	0.003159
3276	ENSG00000151117	TMEM86A	-0.42299	1.150234	0.003159
3277	ENSG00000204611	ZNF616	-0.49912	2.135274	0.003159
3278	ENSG00000213500	LAP3P2	0.595134	2.548609	0.003164
3279	ENSG00000047621	C12orf4	0.266465	4.463469	0.003167
3280	ENSG00000130649	CYP2E1	-0.57484	-0.4115	0.003179
3281	ENSG00000141664	ZCCHC2	0.358659	6.129845	0.003181
3282	ENSG00000267119		-0.28708	6.286191	0.003181
3283	ENSG00000121060	TRIM25	0.360447	6.829691	0.003184
3284	ENSG00000131828	PDHA1	0.172289	5.235524	0.003188
3285	ENSG00000180263	FGD6	-0.48292	3.547519	0.003189
3286	ENSG00000235151		1.131586	-1.09255	0.003193
3287	ENSG00000251634		-0.64672	3.915636	0.003197
3288	ENSG00000023892	DEF6	0.183811	6.84056	0.003199
3289	ENSG00000179397	C1orf101	-0.51337	-0.48633	0.003208
3290	ENSG00000165424	ZCCHC24	-0.48074	2.29871	0.003209
3291	ENSG00000113645	WWC1	-0.68292	-0.43983	0.003211
3292	ENSG00000215283	HMGB3P24	0.550958	-0.73253	0.003211
3293	ENSG00000091527	CDV3	0.183998	8.493441	0.003211
3294	ENSG00000180855	ZNF443	-0.36558	1.541472	0.003235
3295	ENSG00000273151		0.40683	2.366391	0.003241
3296	ENSG00000259207	ITGB3	-0.84996	2.918249	0.003241
3297	ENSG00000130332	LSM7	0.269936	4.979634	0.003258

3298	ENSG00000258441	LINC00641	-0.41545	5.34904	0.00327
3299	ENSG00000156876	SASS6	0.304272	3.33413	0.003272
3300	ENSG00000269834		-0.51483	0.694705	0.003274
3301	ENSG00000175274	TP53I11	-0.39423	4.554247	0.003286
3302	ENSG00000213420	GPC2	-0.57288	0.913966	0.003294
3303	ENSG00000204514	ZNF814	-0.31249	3.733152	0.003299
3304	ENSG00000232690	HSPE1P9	0.709634	-0.9826	0.003306
3305	ENSG00000223768	LINC00205	-0.37488	1.292327	0.003313
3306	ENSG00000092201	SUPT16H	0.227709	7.23906	0.003315
3307	ENSG00000272669		0.65214	1.632063	0.003316
3308	ENSG00000107262	BAG1	0.310798	5.770801	0.003316
3309	ENSG00000186470	BTN3A2	0.333677	8.068865	0.003327
3310	ENSG00000005844	ITGAL	0.318442	9.080417	0.003327
3311	ENSG00000187742	SECISBP2	-0.18889	6.72403	0.003344
3312	ENSG00000241506	PSMC1P1	0.298208	1.798595	0.003355
3313	ENSG00000110717	NDUFS8	0.276951	4.660002	0.003355
3314	ENSG00000065518	NDUFB4	0.234274	5.81456	0.00337
3315	ENSG00000268544		-0.48668	1.092865	0.003375
3316	ENSG00000120860	CCDC53	0.351801	4.727756	0.003409
3317	ENSG00000213790	OLA1P1	0.249722	2.045195	0.003414
3318	ENSG00000120262	CCDC170	-1.12981	1.028343	0.003414
3319	ENSG00000233834		0.813906	-1.11332	0.003416
3320	ENSG00000004399	PLXND1	-0.43251	5.803102	0.003416
3321	ENSG00000179532	DNHD1	-0.34863	5.183105	0.00342
3322	ENSG00000187912	CLEC17A	-0.68745	2.261395	0.003427
3323	ENSG00000163785	RYK	-0.27882	5.547829	0.003428
3324	ENSG00000198838	RYR3	-0.45174	1.170024	0.00343
3325	ENSG00000018189	RUFY3	-0.31522	5.10679	0.00343
3326	ENSG00000119392	GLE1	0.202088	5.239688	0.003432
3327	ENSG00000049249	TNFRSF9	0.885981	2.403048	0.003449
3328	ENSG00000070915	SLC12A3	-0.79683	-1.18605	0.003451
3329	ENSG00000166033	HTRA1	-1.12631	-1.87	0.003451
3330	ENSG00000164105	SAP30	0.473762	3.264729	0.003453
3331	ENSG00000173457	PPP1R14B	0.375922	3.84645	0.003453
3332	ENSG00000182986	ZNF320	-0.35954	3.535687	0.003453
3333	ENSG00000100216	TOMM22	0.200637	5.938793	0.003461
3334	ENSG00000235602	POU5F1P3	-0.71221	-0.0977	0.003461
3335	ENSG00000163492	CCDC141	-0.59305	2.170831	0.003461
3336	ENSG00000161057	PSMC2	0.261025	6.405569	0.003468
3337	ENSG00000226015	CCT8P1	0.244382	4.94459	0.003477
3338	ENSG00000247765		-0.9437	-1.24071	0.003482
3339	ENSG00000120802	TMPO	0.219602	7.483468	0.003492
3340	ENSG00000126698	DNAJC8	0.199435	6.89414	0.003492
3341	ENSG00000125835	SNRPB	0.299041	6.930153	0.003503

3342	ENSG00000125910	S1PR4	0.431433	5.852822	0.003504
3343	ENSG00000106799	TGFBR1	-0.25771	6.762149	0.003512
3344	ENSG00000135437	RDH5	-0.51946	0.424053	0.003512
3345	ENSG00000236876	TMSB4XP1	0.507103	7.258688	0.003524
3346	ENSG00000135749	PCNXL2	-0.31247	5.085845	0.003526
3347	ENSG00000112299	VNN1	-0.68148	4.80066	0.003526
3348	ENSG00000085733	CTTN	-0.70052	2.965428	0.003526
3349	ENSG00000165782	TMEM55B	-0.24509	5.913201	0.003532
3350	ENSG00000053918	KCNQ1	-0.65012	4.369898	0.003535
3351	ENSG00000116237	ICMT	0.393325	4.792138	0.003558
3352	ENSG00000170819	BFSP2	0.664145	-1.24368	0.00356
3353	ENSG00000164039	BDH2	0.327163	3.850618	0.00356
3354	ENSG00000106701	FSD1L	-0.40948	2.812209	0.00356
3355	ENSG00000169991	IFFO2	-0.45593	4.983176	0.00356
3356	ENSG00000196562	SULF2	-0.58875	6.149897	0.003561
3357	ENSG00000189409	MMP23B	0.585972	0.889	0.003563
3358	ENSG00000225507		-0.31584	1.8191	0.003565
3359	ENSG00000103942	HOMER2	-0.41596	1.796726	0.003576
3360	ENSG00000123395	C12orf44	0.256611	3.459839	0.003592
3361	ENSG00000174946	GPR171	0.639385	5.273725	0.003597
3362	ENSG00000118680	MYL12B	0.210053	8.595107	0.003603
3363	ENSG00000181722	ZBTB20	-0.42407	2.220049	0.003607
3364	ENSG00000138134	STAMBPL1	0.289169	4.249271	0.00361
3365	ENSG00000123416	TUBA1B	0.498836	6.317293	0.003612
3366	ENSG00000224546	EIF4BP3	-0.29535	3.565129	0.003625
3367	ENSG00000156795	WDYHV1	0.369264	2.371547	0.003627
3368	ENSG00000224831		0.273506	1.359737	0.003654
3369	ENSG00000107020	PLGRKT	0.343417	3.760245	0.003661
3370	ENSG00000182319		-0.51992	2.702214	0.003665
3371	ENSG00000153443	UBALD1	0.314637	3.713797	0.003677
3372	ENSG00000121753	BAI2	-0.62904	-0.19963	0.00368
3373	ENSG00000170949	ZNF160	-0.26613	5.092456	0.003684
3374	ENSG00000238249	HMGN2P17	0.448361	1.200169	0.003689
3375	ENSG00000186812	ZNF397	-0.39021	3.703071	0.003699
3376	ENSG00000132623	ANKEF1	-0.3787	1.549426	0.003712
3377	ENSG00000163686	ABHD6	-0.33501	2.712966	0.003718
3378	ENSG00000228589	SPCS2P4	0.429157	1.205589	0.003724
3379	ENSG00000271198	VDAC3P1	0.38496	0.796566	0.003724
3380	ENSG00000189190	ZNF600	-0.4857	4.272757	0.00373
3381	ENSG00000184436	THAP7	-0.2404	4.320727	0.00373
3382	ENSG00000116729	WLS	-0.87148	2.472332	0.00373
3383	ENSG00000116990	MYCL	-1.07693	2.636759	0.003731
3384	ENSG00000099795	NDUFB7	0.395677	5.001063	0.003738
3385	ENSG00000105808	RASA4	-0.43458	3.315542	0.00375

3386	ENSG00000248571		0.934759	-0.57637	0.003751
3387	ENSG00000228486	LINC01125	-0.36998	1.184744	0.003755
3388	ENSG00000272501		-0.6273	1.566961	0.00376
3389	ENSG00000238103	RPL9P7	-0.34144	7.16269	0.003761
3390	ENSG00000123124	WWP1	-0.19656	6.826103	0.003766
3391	ENSG00000147533	GOLGA7	0.143167	6.681598	0.003768
3392	ENSG00000090006	LTBP4	-0.35039	4.625144	0.003768
3393	ENSG00000240754		-0.70279	-1.07974	0.003775
3394	ENSG00000178950	GAK	0.218434	6.594906	0.00379
3395	ENSG00000173369	C1QB	1.151366	2.856605	0.003799
3396	ENSG00000197345	MRPL21	0.241689	4.439295	0.0038
3397	ENSG00000140943	MBTPS1	-0.13484	6.969315	0.0038
3398	ENSG00000254363		-0.64015	0.635068	0.003831
3399	ENSG00000142632	ARHGEF19	0.255463	2.64911	0.003835
3400	ENSG00000115042	FAHD2A	0.217805	3.463588	0.003836
3401	ENSG00000182903	ZNF721	-0.38766	5.358588	0.003836
3402	ENSG00000088053	GP6	-0.70423	0.812184	0.003836
3403	ENSG00000155893	ACPL2	-0.24747	3.764524	0.003842
3404	ENSG00000122477	LRRC39	-0.41056	0.806343	0.003847
3405	ENSG00000244567		-0.30041	2.093759	0.003854
3406	ENSG00000120656	TAF12	0.314759	4.544344	0.00386
3407	ENSG00000165917	RAPSN	-0.64219	-0.82146	0.003872
3408	ENSG00000101752	MIB1	-0.23801	6.118129	0.003874
3409	ENSG00000129473	BCL2L2	-0.33434	3.436809	0.003881
3410	ENSG00000177889	UBE2N	0.250951	7.075381	0.003886
3411	ENSG00000162545	CAMK2N1	-0.56178	0.956988	0.003886
3412	ENSG00000104980	TIMM44	0.18768	4.338497	0.003886
3413	ENSG00000111481	COPZ1	0.23582	6.746405	0.003886
3414	ENSG00000089060	SLC8B1	-0.2603	5.095127	0.003896
3415	ENSG00000184702	SEPT5	-0.76402	2.254448	0.003902
3416	ENSG00000243279	PRAF2	0.311879	3.358793	0.003923
3417	ENSG00000228232	GAPDHP1	0.340157	3.853629	0.003925
3418	ENSG00000231345		0.390391	1.194602	0.003932
3419	ENSG00000133317	LGALS12	-0.56006	1.821555	0.003934
3420	ENSG00000251000		0.390721	-0.0268	0.003942
3421	ENSG00000203485	INF2	-0.43864	4.337176	0.003948
3422	ENSG00000111885	MAN1A1	0.322802	6.697263	0.003951
3423	ENSG00000261804		-0.99203	-1.66667	0.003951
3424	ENSG00000171159	C9orf16	0.306024	5.104212	0.003958
3425	ENSG00000132561	MATN2	-0.69877	-0.90145	0.00396
3426	ENSG00000100577	GSTZ1	0.350749	2.496565	0.003967
3427	ENSG00000160221	C21orf33	0.242717	5.325786	0.003967
3428	ENSG00000157306		-0.45486	0.486828	0.003969
3429	ENSG00000140836	ZFHX3	-0.57721	3.625964	0.003986

3430	ENSG00000167383	ZNF229	-0.8198	-1.18837	0.003988
3431	ENSG00000162836	ACP6	-0.37519	2.603609	0.003989
3432	ENSG00000138798	EGF	-0.8299	1.75528	0.003997
3433	ENSG00000177663	IL17RA	-0.42912	6.377193	0.003997
3434	ENSG00000164707	SLC13A4	-0.7916	0.015476	0.004042
3435	ENSG00000171262	FAM98B	0.457411	4.77916	0.004049
3436	ENSG00000132591	ERAL1	0.26121	4.374711	0.004049
3437	ENSG00000184100	BRD7P2	0.431423	0.311116	0.00405
3438	ENSG00000260276		0.285832	1.025309	0.004057
3439	ENSG00000135945	REV1	-0.18154	5.857458	0.004073
3440	ENSG00000241553	ARPC4	0.284377	7.074708	0.00408
3441	ENSG00000189376	C8orf76	0.289775	2.449412	0.004081
3442	ENSG00000156510	HKDC1	-0.82235	0.156486	0.004081
3443	ENSG00000213938	SEPHS1P6	0.299637	0.455579	0.004082
3444	ENSG00000148248	SURF4	0.347476	7.602908	0.004086
3445	ENSG00000234518	PTGES3P1	0.308156	3.079618	0.004086
3446	ENSG00000260766		-0.51297	0.421537	0.004088
3447	ENSG00000147010	SH3KBP1	0.207253	7.414021	0.004104
3448	ENSG00000116698	SMG7	0.182918	6.609568	0.004109
3449	ENSG00000155974	GRIP1	-0.41553	1.21036	0.00411
3450	ENSG00000113068	PFDN1	0.214954	5.803359	0.004114
3451	ENSG00000249667		-0.71817	-0.149	0.004116
3452	ENSG00000125977	EIF2S2	0.282844	6.647481	0.004124
3453	ENSG00000196670	ZFP62	-0.28693	4.301741	0.004127
3454	ENSG00000154370	TRIM11	0.211782	5.000066	0.004136
3455	ENSG00000169020	ATP5I	0.379795	5.283065	0.004139
3456	ENSG00000154856	APCDD1	-0.81696	-0.76951	0.004157
3457	ENSG00000145431	PDGFC	-0.57998	1.576361	0.004172
3458	ENSG00000234882	EIF3EP1	-0.24977	4.036203	0.004174
3459	ENSG00000176809	LRRC37A3	-0.30225	3.004149	0.004182
3460	ENSG00000226029		-0.43249	1.285622	0.004184
3461	ENSG00000077713	SLC25A43	-0.41299	3.131938	0.00419
3462	ENSG00000196712	NF1	0.219426	6.206826	0.004203
3463	ENSG00000172331	BPGM	0.499791	5.508922	0.004206
3464	ENSG00000214046	SMIM7	0.17618	5.084391	0.004206
3465	ENSG00000270574		-0.46058	0.187536	0.00421
3466	ENSG00000144476	ACKR3	-0.62827	1.176973	0.00421
3467	ENSG00000006007	GDE1	0.185502	5.800429	0.004216
3468	ENSG00000124942	AHNAK	-0.49429	10.96951	0.004216
3469	ENSG00000101888	NXT2	0.245896	4.738748	0.004216
3470	ENSG00000171202	TMEM126A	0.262526	4.36028	0.004221
3471	ENSG00000257576		0.487658	-0.19566	0.004222
3472	ENSG00000237984	PTENP1	-0.52324	-0.19891	0.004226
3473	ENSG00000117593	DARS2	0.366641	3.043441	0.004239

3474	ENSG00000181908		-1.12315	-1.22951	0.004245
3475	ENSG00000171766	GATM	-0.47067	1.249833	0.004245
3476	ENSG00000165359	DDX26B	-0.30265	6.365477	0.004264
3477	ENSG00000145216	FIP1L1	0.186845	5.197026	0.004269
3478	ENSG00000240898		-0.44184	0.635505	0.004272
3479	ENSG00000263753	LINC00667	-0.26572	5.241187	0.004272
3480	ENSG00000174444	RPL4	-0.24733	11.37652	0.00428
3481	ENSG00000077235	GTF3C1	0.354159	6.600354	0.004285
3482	ENSG00000254539		-0.57769	-0.26734	0.004285
3483	ENSG00000148498	PARD3	-0.6779	0.377486	0.004285
3484	ENSG00000070371	CLTCL1	-0.68673	0.946089	0.004286
3485	ENSG00000159164	SV2A	-0.41642	1.330417	0.004294
3486	ENSG00000163219	ARHGAP25	0.242629	7.202445	0.004297
3487	ENSG00000065491	TBC1D22B	0.195966	4.437729	0.004297
3488	ENSG00000163867	ZMYM6	-0.25629	5.146647	0.004297
3489	ENSG00000188641	DPYD	-0.45801	6.872352	0.004297
3490	ENSG00000171643	S100Z	-0.81954	1.580643	0.004297
3491	ENSG00000261490		-0.45593	3.966204	0.004301
3492	ENSG00000140848	CPNE2	-0.61321	3.297372	0.004304
3493	ENSG00000002586	CD99	0.303286	6.21628	0.004308
3494	ENSG00000231528	FAM225A	1.024338	1.755841	0.004309
3495	ENSG00000188092	GPR89B	0.198489	4.255569	0.004309
3496	ENSG00000128487	SPECC1	-0.67466	3.97504	0.004316
3497	ENSG00000204634	TBC1D8	-0.43068	5.013452	0.004323
3498	ENSG00000197646	PDCD1LG2	0.694626	0.365969	0.004354
3499	ENSG00000103316	CRYM	-0.96209	-1.40035	0.004384
3500	ENSG00000138686	BBS7	0.309763	3.311431	0.004407
3501	ENSG00000183486	MX2	0.503577	7.119998	0.004419
3502	ENSG00000271948		-0.44061	1.472223	0.004421
3503	ENSG00000135426	TESPA1	-0.29446	6.005104	0.004426
3504	ENSG00000165476	REEP3	-0.24788	5.009577	0.004436
3505	ENSG00000261253		-0.71782	0.031081	0.004456
3506	ENSG00000270472	IGHV3OR16-9	1.039103	-0.06009	0.00446
3507	ENSG00000187164	KIAA1598	-0.7372	4.059882	0.004461
3508	ENSG00000160219	GAB3	0.327406	6.151244	0.004467
3509	ENSG00000175701	LINC00116	0.411959	2.10772	0.004477
3510	ENSG00000196172	ZNF681	-0.43045	2.350393	0.004485
3511	ENSG00000132376	INPP5K	0.26306	6.335605	0.004486
3512	ENSG00000269928		-0.3368	3.020338	0.004486
3513	ENSG00000179104	TMTC2	-0.62669	3.175129	0.004491
3514	ENSG00000133030	MPRIP	-0.298	7.289713	0.004493
3515	ENSG00000249489	GAPDHP70	0.43185	0.326795	0.004501
3516	ENSG00000134852	CLOCK	-0.28707	5.185191	0.004501
3517	ENSG00000133703	KRAS	0.311675	7.061547	0.004507

3518	ENSG00000198339	HIST1H4I	0.558928	-0.46653	0.004514
3519	ENSG00000176261	ZBTB8OS	0.276501	4.489581	0.004524
3520	ENSG00000227053		0.692999	-1.0883	0.004531
3521	ENSG00000157578	LCA5L	-0.71579	-1.05846	0.004542
3522	ENSG00000182795	C1orf116	-0.7316	-1.12224	0.004548
3523	ENSG00000108389	MTMR4	-0.17651	5.807309	0.004548
3524	ENSG00000068650	ATP11A	-0.34499	6.277751	0.004572
3525	ENSG00000116678	LEPR	-0.51042	3.251298	0.004576
3526	ENSG00000156261	CCT8	0.232196	7.557845	0.004594
3527	ENSG00000145012	LPP	-0.24657	6.542192	0.004597
3528	ENSG00000221883	ARIH2OS	0.35419	1.085959	0.0046
3529	ENSG00000188321	ZNF559	-0.42237	4.344746	0.004621
3530	ENSG00000169282	KCNAB1	-0.39771	0.678213	0.004624
3531	ENSG00000084652	TXLNA	0.297057	5.508828	0.004636
3532	ENSG00000146701	MDH2	0.325142	6.048111	0.00464
3533	ENSG00000111358	GTF2H3	0.223636	4.684223	0.00464
3534	ENSG00000147570	DNAJC5B	-0.65709	-1.0573	0.004643
3535	ENSG00000240682	ISY1	0.187024	4.846702	0.004647
3536	ENSG00000130813	C19orf66	0.336016	5.581383	0.004655
3537	ENSG00000237190	CDKN2AIPNL	0.26705	3.464928	0.004655
3538	ENSG00000255993	PSMC1P9	0.259315	3.009861	0.004655
3539	ENSG00000134717	BTF3L4	-0.17516	5.959166	0.004655
3540	ENSG00000122406	RPL5	-0.27466	10.32601	0.004655
3541	ENSG00000144362	PHOSPHO2	-0.34727	0.396061	0.004661
3542	ENSG00000133112	TPT1	-0.27928	12.24992	0.004672
3543	ENSG00000246273	SBF2-AS1	-0.43188	0.365017	0.004672
3544	ENSG00000196628	TCF4	-0.38572	5.403438	0.004681
3545	ENSG00000211764	TRBJ2-1	0.571045	-0.62213	0.0047
3546	ENSG00000165494	PCF11	-0.27375	7.043878	0.004706
3547	ENSG00000168259	DNAJC7	0.223552	6.199042	0.004714
3548	ENSG00000123095	BHLHE41	0.702684	0.489908	0.004717
3549	ENSG00000134202	GSTM3	-0.54063	2.591817	0.004726
3550	ENSG00000151632	AKR1C2	-0.60179	-0.1511	0.004726
3551	ENSG00000223891	OSER1-AS1	-0.50815	2.265955	0.004743
3552	ENSG00000197181	PIWIL2	-0.63003	-0.87423	0.004745
3553	ENSG00000188171	ZNF626	-0.45995	2.347959	0.004745
3554	ENSG00000212993	POU5F1B	-0.43367	0.02866	0.004746
3555	ENSG00000110400	PVRL1	-0.51063	2.811568	0.004753
3556	ENSG00000167461	RAB8A	0.245808	5.479837	0.004767
3557	ENSG00000261534		-0.60619	0.441134	0.004779
3558	ENSG00000156958	GALK2	0.385508	3.473615	0.004788
3559	ENSG00000139719	VPS33A	0.251575	4.164171	0.004788
3560	ENSG00000213246	SUPT4H1	0.182253	6.6366	0.004789
3561	ENSG00000173272	MZT2A	0.389318	4.141655	0.004799

3562	ENSG00000227203	SUB1P1	0.855335	0.423145	0.004804
3563	ENSG00000169683	LRRC45	0.471241	3.148615	0.004804
3564	ENSG00000101138	CSTF1	0.331056	4.683948	0.004804
3565	ENSG00000146085	MUT	0.272804	4.492739	0.004804
3566	ENSG00000176148	TCP11L1	0.211704	3.57811	0.004806
3567	ENSG00000158966	CACHD1	-0.79245	0.477692	0.004812
3568	ENSG00000250348		-0.84256	-1.26001	0.004813
3569	ENSG00000169442	CD52	0.382147	9.028808	0.004814
3570	ENSG00000107897	ACBD5	0.206216	5.891652	0.004817
3571	ENSG00000246334	PRR7-AS1	-0.54952	1.449558	0.00482
3572	ENSG00000255964		0.47025	-0.31499	0.004825
3573	ENSG00000121680	PEX16	0.212951	4.526495	0.004854
3574	ENSG00000072134	EPN2	-0.36466	2.069794	0.004854
3575	ENSG00000128185	DGCR6L	0.318743	4.582171	0.004865
3576	ENSG00000169118	CSNK1G1	0.229634	5.280255	0.00487
3577	ENSG00000158545	ZC3H18	0.28137	5.680084	0.004887
3578	ENSG00000198612	COPS8	0.188443	5.742631	0.004888
3579	ENSG00000152082	MZT2B	0.453023	4.576379	0.004899
3580	ENSG00000101342	TLDC2	-0.39512	3.610626	0.004904
3581	ENSG00000135549	PKIB	-0.67138	-0.3224	0.004909
3582	ENSG00000117682	DHDDS	0.175317	4.229641	0.004929
3583	ENSG00000267654		0.810469	-0.61494	0.004929
3584	ENSG00000080493	SLC4A4	-0.51032	3.189383	0.004948
3585	ENSG00000182512	GLRX5	0.399365	4.461747	0.004953
3586	ENSG00000159069	FBXW5	0.305432	6.290048	0.004953
3587	ENSG00000230565	ZNF32-AS2	-0.53819	-0.27215	0.004959
3588	ENSG00000100385	IL2RB	0.499295	7.761848	0.004967
3589	ENSG00000159904	ZNF890P	-0.55703	1.579745	0.004968
3590	ENSG00000139890	REM2	-0.44844	1.650064	0.004969
3591	ENSG00000185608	MRPL40	0.296167	3.78953	0.004994
3592	ENSG00000137135	ARHGEF39	0.288839	1.434394	0.004997
3593	ENSG00000136463	TACO1	0.254703	3.266495	0.004997
3594	ENSG00000213492	NT5C3AP1	0.4372	3.321114	0.005001
3595	ENSG00000108474	PIGL	-0.32765	3.617505	0.005005
3596	ENSG00000140563	MCTP2	0.337043	5.082297	0.005015
3597	ENSG00000169313	P2RY12	-0.70755	1.433124	0.005025
3598	ENSG00000234456	MAGI2-AS3	-0.83315	0.989888	0.005034
3599	ENSG00000186364	NUDT17	-0.33253	0.956417	0.005041
3600	ENSG00000254170		-0.36931	4.688049	0.005041
3601	ENSG00000154928	EPHB1	-0.72244	0.584442	0.005056
3602	ENSG00000259746	HSPE1P3	0.595608	-0.85562	0.005067
3603	ENSG00000162430	SEPN1	-0.54427	4.479752	0.00507
3604	ENSG00000133818	RRAS2	0.393306	4.513534	0.005073
3605	ENSG00000272760		-0.36272	1.689143	0.005073

3606	ENSG00000120057	SFRP5	-1.02624	-1.49433	0.005073
3607	ENSG00000113296	THBS4	-0.54083	0.169296	0.005095
3608	ENSG00000101084	C20orf24	0.441977	3.163222	0.005102
3609	ENSG00000149256	TENM4	-1.39452	-2.01418	0.005105
3610	ENSG00000269896		-0.57421	-0.32371	0.00511
3611	ENSG00000126457	PRMT1	0.225323	5.953382	0.005134
3612	ENSG00000108679	LGALS3BP	0.733176	4.813267	0.005154
3613	ENSG00000077549	CAPZB	0.274957	7.908517	0.005166
3614	ENSG00000259413		0.397552	-0.31511	0.005172
3615	ENSG00000198482	ZNF808	-0.2755	4.166923	0.005172
3616	ENSG00000179361	ARID3B	0.22491	5.143083	0.005179
3617	ENSG00000122378	FAM213A	-0.3688	1.879883	0.00518
3618	ENSG00000233093	LINC00892	-0.74238	0.881712	0.005198
3619	ENSG00000261474		-0.59933	-0.83683	0.005206
3620	ENSG00000232788		-0.4	0.664314	0.005225
3621	ENSG00000260693		-0.50227	0.870054	0.005231
3622	ENSG00000272941		0.444228	0.893666	0.005232
3623	ENSG00000225684	FAM225B	1.00906	1.384758	0.005238
3624	ENSG00000100151	PICK1	-0.2095	3.527959	0.005247
3625	ENSG00000174013	FBXO45	0.29371	4.136953	0.00525
3626	ENSG00000203867	RBM20	-0.7818	-0.69348	0.00525
3627	ENSG00000083838	ZNF446	-0.34866	2.756355	0.005259
3628	ENSG00000159958	TNFRSF13C	-0.56629	2.204616	0.005263
3629	ENSG00000126264	HCST	0.365739	6.420972	0.00527
3630	ENSG00000236698	EIF1AXP1	0.346696	3.595497	0.005286
3631	ENSG00000139921	TMX1	0.269494	6.20874	0.005299
3632	ENSG00000249437	NAIP	-0.62574	5.1912	0.005301
3633	ENSG00000188372	ZP3	-0.40653	1.622776	0.005307
3634	ENSG00000248569		0.325154	0.639012	0.005309
3635	ENSG00000122545	SEPT7	0.203649	8.53734	0.005311
3636	ENSG00000267533		0.466358	1.967106	0.005334
3637	ENSG00000134248	LAMTOR5	0.187768	5.932435	0.005334
3638	ENSG00000119403	PHF19	0.198646	5.647789	0.005337
3639	ENSG00000259330	LINC00984	-0.4285	2.913146	0.005338
3640	ENSG00000100902	PSMA6	0.361448	3.750798	0.005341
3641	ENSG00000070770	CSNK2A2	0.19075	6.109887	0.005344
3642	ENSG00000125352	RNF113A	0.200456	4.644277	0.005348
3643	ENSG00000124313	IQSEC2	-0.64883	2.638079	0.005348
3644	ENSG00000141682	PMAIP1	0.440702	6.580572	0.005356
3645	ENSG00000152234	ATP5A1	0.179012	8.47645	0.005368
3646	ENSG00000091592	NLRP1	-0.21222	8.051049	0.005378
3647	ENSG00000170727	BOP1	0.300913	3.722009	0.005394
3648	ENSG00000231128		0.642835	0.247396	0.005398
3649	ENSG00000004455	AK2	0.199761	6.841862	0.005401

3650	ENSG00000108561	C1QBP	0.204195	5.601922	0.005404
3651	ENSG00000204315	FKBPL	0.427944	0.989781	0.005414
3652	ENSG00000224040	HMGN1P4	0.375983	2.573335	0.005414
3653	ENSG00000056050	C4orf27	0.363983	3.496544	0.005414
3654	ENSG00000132475	H3F3B	0.284055	10.16719	0.005414
3655	ENSG00000067208	EVI5	-0.39911	3.899057	0.005414
3656	ENSG00000120885	CLU	-0.74619	5.35967	0.005414
3657	ENSG00000233387		-0.81896	-0.60564	0.005418
3658	ENSG00000105205	CLC	-0.74547	4.554102	0.005426
3659	ENSG00000187800	PEAR1	-0.76914	1.521201	0.00543
3660	ENSG00000111726	CMAS	0.225283	4.55221	0.005453
3661	ENSG00000014919	COX15	0.287605	4.688248	0.005454
3662	ENSG00000169299	PGM2	0.269292	5.247051	0.005473
3663	ENSG00000251606		-0.81816	-1.13302	0.005477
3664	ENSG00000122566	HNRNPA2B1	0.176743	9.919629	0.005481
3665	ENSG00000106305	AIMP2	0.282025	2.003369	0.005482
3666	ENSG00000131732	ZCCHC9	0.23264	4.250057	0.005495
3667	ENSG00000235437		-0.25239	3.917462	0.005501
3668	ENSG00000143595	AQP10	-0.94562	-0.29654	0.005507
3669	ENSG00000143314	MRPL24	0.30056	4.140023	0.005514
3670	ENSG00000219626	FAM228B	-0.37756	2.628878	0.005514
3671	ENSG00000181544	FANCB	0.347197	1.306665	0.005537
3672	ENSG00000111412	C12orf49	0.190103	5.045794	0.005537
3673	ENSG00000213864	EEF1B2P2	-0.48439	0.148005	0.005537
3674	ENSG00000092200	RPGRIP1	-0.63754	0.968216	0.005568
3675	ENSG00000146731	CCT6A	0.194772	7.095018	0.00558
3676	ENSG00000267352	SH3GL1P3	-0.32529	2.433327	0.005582
3677	ENSG00000141582	CBX4	0.365741	5.591081	0.005585
3678	ENSG00000174255	ZNF80	0.616213	1.638016	0.005606
3679	ENSG00000146733	PSPH	0.342154	2.337691	0.005606
3680	ENSG00000128881	TTBK2	-0.31539	5.192113	0.005606
3681	ENSG00000211940	IGHV3-9	1.177845	1.004894	0.005618
3682	ENSG00000145919	BOD1	-0.26725	3.938597	0.005618
3683	ENSG00000151023	ENKUR	-0.74655	2.338837	0.005618
3684	ENSG00000172059	KLF11	-0.47522	5.906676	0.005628
3685	ENSG00000204010	IFIT1B	1.205686	0.704071	0.005632
3686	ENSG00000164885	CDK5	0.427369	2.971865	0.005632
3687	ENSG00000178222	RNF212	-0.66527	-0.21123	0.005632
3688	ENSG00000080947	CROCCP3	-0.39594	2.578867	0.005633
3689	ENSG00000269202		-0.72618	-0.78632	0.005644
3690	ENSG00000163444	TMEM183A	0.160947	7.002807	0.005646
3691	ENSG00000216809		-0.59243	-0.31696	0.00565
3692	ENSG00000119900	OGFRL1	-0.38342	7.411248	0.005666
3693	ENSG00000131148	EMC8	0.220223	4.265278	0.005669

3694	ENSG00000165527	ARF6	0.25545	7.57831	0.005675
3695	ENSG00000177875	C12orf68	-0.97922	-1.43272	0.005675
3696	ENSG00000242457	RBBP4P2	0.238604	2.353748	0.005682
3697	ENSG00000151240	DIP2C	-0.37849	2.382535	0.005682
3698	ENSG00000189067	LITAF	0.350141	9.061403	0.005687
3699	ENSG00000159461	AMFR	-0.21062	6.31674	0.005687
3700	ENSG00000167578	RAB4B	0.263902	4.229169	0.005691
3701	ENSG00000245205	EEF1A1P4	-0.25445	2.149998	0.005691
3702	ENSG00000164048	ZNF589	-0.22704	4.893088	0.005699
3703	ENSG00000129559	NEDD8	0.252991	5.891855	0.005702
3704	ENSG00000254098	IGKV2-26	0.990301	-0.32544	0.005714
3705	ENSG00000115661	STK16	0.291812	4.580744	0.005714
3706	ENSG00000160712	IL6R	-0.38882	6.619548	0.005721
3707	ENSG00000231313	CLIC1P1	0.324889	3.839208	0.005728
3708	ENSG00000171490	RSL1D1	-0.21236	7.416742	0.005728
3709	ENSG00000101000	PROCR	-0.39819	0.498244	0.005736
3710	ENSG00000253738		-0.28769	4.696843	0.005751
3711	ENSG00000165637	VDAC2	0.183937	6.413906	0.00577
3712	ENSG00000135736	CCDC102A	0.476547	1.924559	0.005783
3713	ENSG00000269404	SPIB	-0.55319	3.361031	0.005783
3714	ENSG00000215914	MMP23A	0.601827	0.330046	0.005802
3715	ENSG00000198734	F5	-0.52516	4.42669	0.005802
3716	ENSG00000182247	UBE2E2	-0.34274	3.336555	0.005814
3717	ENSG00000217897	HSPE1P8	0.563068	-0.52115	0.005815
3718	ENSG00000116750	UCHL5	0.180222	5.432729	0.005824
3719	ENSG00000258768		-0.46388	0.710392	0.005845
3720	ENSG00000228794	LINC01128	-0.37724	3.765493	0.005879
3721	ENSG00000112486	CCR6	-0.7903	0.546736	0.005879
3722	ENSG00000164904	ALDH7A1	-0.53683	0.113313	0.005879
3723	ENSG00000168824		-0.78599	2.412928	0.005879
3724	ENSG00000133477	FAM83F	-0.97118	-0.40488	0.005879
3725	ENSG00000149639	SOGA1	-0.5211	3.394121	0.005894
3726	ENSG00000146021	KLHL3	-0.38548	3.782504	0.005904
3727	ENSG00000083750	RRAGB	-0.23715	3.655867	0.005912
3728	ENSG00000183688	FAM101B	-0.43627	5.521735	0.005919
3729	ENSG00000136156	ITM2B	-0.25395	9.298573	0.005921
3730	ENSG00000159189	C1QC	1.402499	0.121315	0.005925
3731	ENSG00000178467	P4HTM	-0.21602	4.794671	0.005925
3732	ENSG00000059377	TBXAS1	-0.53351	6.092009	0.005929
3733	ENSG00000269001	ZNF818P	-0.67934	-0.63719	0.005933
3734	ENSG00000196569	LAMA2	-0.5553	0.321549	0.005934
3735	ENSG00000047597	XK	-0.68054	2.23085	0.005951
3736	ENSG00000145700	ANKRD31	-0.69113	-0.81774	0.005951
3737	ENSG00000163607	GTPBP8	0.309573	4.033549	0.005955

3738	ENSG00000234184		-0.74871	0.341381	0.005985
3739	ENSG00000149260	CAPN5	-0.40761	1.501408	0.005985
3740	ENSG00000127920	GNG11	-0.72626	4.7286	0.006011
3741	ENSG00000250687		-0.64379	1.240713	0.006022
3742	ENSG00000243777		-0.33099	3.112256	0.006042
3743	ENSG00000163393	SLC22A15	-0.49357	3.396823	0.006042
3744	ENSG00000130783	CCDC62	-0.51989	-0.52815	0.006042
3745	ENSG00000167792	NDUFV1	0.186655	6.334408	0.006046
3746	ENSG00000001629	ANKIB1	0.200068	6.342322	0.006052
3747	ENSG00000146856	AGBL3	-0.44303	0.651431	0.006052
3748	ENSG00000186193	SAPCD2	0.634454	0.948258	0.006053
3749	ENSG00000211899	IGHM	0.807216	8.319206	0.006059
3750	ENSG00000100580	TMED8	-0.17404	5.586358	0.006059
3751	ENSG00000183813	CCR4	-0.77026	5.039821	0.006059
3752	ENSG00000214264		0.463588	-0.38538	0.006063
3753	ENSG00000144034	TPRKB	0.243793	4.20677	0.006063
3754	ENSG00000175741	RWDD4P2	0.289969	1.198401	0.006076
3755	ENSG00000145425	RPS3A	-0.32489	10.59402	0.006083
3756	ENSG00000132581	SDF2	0.242015	5.230947	0.00609
3757	ENSG00000127311	HELB	0.376603	4.428817	0.006099
3758	ENSG00000105197	TIMM50	0.170051	4.648448	0.006103
3759	ENSG00000172009	THOP1	0.266357	3.89688	0.006108
3760	ENSG00000214517	PPME1	0.228158	3.595917	0.006124
3761	ENSG00000100991	TRPC4AP	0.209881	6.928534	0.006127
3762	ENSG00000137478	FCHSD2	-0.26288	6.112619	0.006127
3763	ENSG00000183648	NDUFB1	0.320144	4.748844	0.006134
3764	ENSG00000196268	ZNF493	-0.33471	4.17051	0.006136
3765	ENSG00000100814	CCNB1IP1	0.264509	5.066292	0.006146
3766	ENSG00000111341	MGP	-0.68412	-0.82276	0.006146
3767	ENSG00000134575	ACP2	0.399723	3.770661	0.006167
3768	ENSG00000180992	MRPL14	0.252858	4.078124	0.006168
3769	ENSG00000160305	DIP2A	0.316432	6.94855	0.006173
3770	ENSG00000138615	CILP	-0.52033	-0.65765	0.006173
3771	ENSG00000070495	JMJD6	0.515584	6.465054	0.006173
3772	ENSG00000103522	IL21R	0.765918	4.942484	0.006182
3773	ENSG00000102362	SYTL4	-0.77508	-0.27525	0.006193
3774	ENSG00000185024	BRF1	0.260275	4.97691	0.006209
3775	ENSG00000171867	PRNP	-0.33127	7.50282	0.006227
3776	ENSG00000266401		-0.89583	-0.89485	0.006228
3777	ENSG00000138018	EPT1	0.28278	6.007913	0.006254
3778	ENSG00000104907	TRMT1	-0.17456	5.098318	0.006256
3779	ENSG00000164649	CDCA7L	0.322817	4.207851	0.00627
3780	ENSG00000074842	C19orf10	0.298058	5.289641	0.006276
3781	ENSG00000172766	NAA16	-0.22751	5.575873	0.006276

3782	ENSG00000088986	DYNLL1	0.430932	5.899192	0.006282
3783	ENSG00000147036	LANCL3	0.377986	1.756059	0.006282
3784	ENSG00000168675	LDLRAD4	-0.37695	5.271316	0.006293
3785	ENSG00000260244		-0.65706	0.664553	0.006303
3786	ENSG00000166736	HTR3A	-0.98876	-0.95825	0.006303
3787	ENSG00000198856	OSTC	0.221576	6.094321	0.006309
3788	ENSG00000211778	TRAV4	0.535168	1.312093	0.006325
3789	ENSG00000268166		-0.72645	-0.83243	0.006338
3790	ENSG00000125898	FAM110A	-0.41294	4.032521	0.006355
3791	ENSG00000009724	MASP2	-0.50425	2.73159	0.006363
3792	ENSG00000142408	CACNG8	-0.83428	-1.2805	0.006377
3793	ENSG00000170906	NDUFA3	0.261311	4.59	0.006381
3794	ENSG00000158092	NCK1	0.191208	5.775453	0.006381
3795	ENSG00000110497	AMBRA1	0.290518	4.898481	0.006382
3796	ENSG00000185475	TMEM179B	0.223124	3.999935	0.006382
3797	ENSG00000114204	SERPINI2	-1.10589	-1.88935	0.006382
3798	ENSG00000100426	ZBED4	0.285713	5.670608	0.006383
3799	ENSG00000135678	CPM	-0.45925	2.792345	0.006391
3800	ENSG00000171103	TRMT61B	-0.17921	2.968311	0.006402
3801	ENSG00000075413	MARK3	0.143855	6.697013	0.006408
3802	ENSG00000165475	CRYL1	-0.37451	3.64897	0.00642
3803	ENSG00000100034	PPM1F	-0.43299	6.261274	0.006428
3804	ENSG00000117450	PRDX1	0.275217	6.739836	0.006446
3805	ENSG00000091732	ZC3HC1	0.217388	3.27738	0.006446
3806	ENSG00000184923	NUTM2A	-0.37133	1.035285	0.00645
3807	ENSG00000048342	CC2D2A	-0.56471	0.232038	0.006457
3808	ENSG00000175536	LIPT2	-0.42277	0.668682	0.006459
3809	ENSG00000170209	ANKK1	-0.61209	-0.67425	0.00646
3810	ENSG00000178952	TUFM	0.22539	6.864602	0.006466
3811	ENSG00000132704	FCRL2	-0.57566	4.157649	0.006467
3812	ENSG00000226396	RPS14P3	-0.43112	1.494871	0.006469
3813	ENSG00000254614		-0.6655	2.187108	0.006471
3814	ENSG00000102898	NUTF2	0.247038	5.207634	0.006473
3815	ENSG00000100372	SLC25A17	0.242471	3.807266	0.00648
3816	ENSG00000120049	KCNIP2	-0.43946	0.389037	0.006481
3817	ENSG00000104964	AES	0.241195	8.420241	0.006493
3818	ENSG00000184227	ACOT1	0.400873	2.354608	0.006509
3819	ENSG00000240505	TNFRSF13B	0.601828	2.252584	0.006522
3820	ENSG00000230581		-0.71975	-0.26311	0.006537
3821	ENSG00000113140	SPARC	-0.75197	6.304922	0.006538
3822	ENSG00000248632		0.6408	-0.6123	0.006541
3823	ENSG00000236259		0.447493	-0.3558	0.006541
3824	ENSG00000139725	RHOF	0.371853	2.444062	0.006557
3825	ENSG00000215464		-0.27605	3.902929	0.006561

3826	ENSG00000100784	RPS6KA5	-0.30505	4.194395	0.006564
3827	ENSG00000111052	LIN7A	-0.67437	2.911175	0.006583
3828	ENSG00000138386	NAB1	0.521754	5.918221	0.00659
3829	ENSG00000069509	FUNDC1	0.260207	2.955129	0.006598
3830	ENSG00000154736	ADAMTS5	-1.32919	-2.08099	0.006598
3831	ENSG00000231169	EEF1B2P1	-0.3491	3.758575	0.006602
3832	ENSG00000136732	GYPC	0.394255	6.6066	0.006632
3833	ENSG00000047249	ATP6V1H	0.237855	4.658305	0.006635
3834	ENSG00000271858		-0.50732	0.678697	0.006635
3835	ENSG00000203872	C6orf163	-0.55733	-0.55571	0.006636
3836	ENSG00000198015	MRPL42	0.213433	5.414835	0.006637
3837	ENSG00000236570	RAD23BP1	0.317369	1.616539	0.006638
3838	ENSG00000247556	OIP5-AS1	-0.24273	7.207724	0.006638
3839	ENSG00000166848	TERF2IP	0.227778	7.020259	0.006652
3840	ENSG00000271147		-0.38766	1.613314	0.006665
3841	ENSG00000130511	SSBP4	0.422488	5.322962	0.006665
3842	ENSG00000183011	LSMD1	0.384122	4.191207	0.006667
3843	ENSG00000162517	PEF1	0.265838	5.533322	0.006668
3844	ENSG00000115652	UXS1	-0.19071	5.566057	0.006668
3845	ENSG00000175662	TOM1L2	-0.24251	4.735568	0.006668
3846	ENSG00000135077	HAVCR2	0.662942	5.552722	0.006688
3847	ENSG00000058799	YIPF1	0.239797	4.699004	0.006688
3848	ENSG00000268362		-0.35507	1.377801	0.006693
3849	ENSG00000034053	APBA2	-0.40974	5.25244	0.006744
3850	ENSG00000109063	MYH3	-0.32359	2.293263	0.006744
3851	ENSG00000077044	DGKD	-0.22417	7.059356	0.006746
3852	ENSG00000187145	MRPS21	0.216542	5.479664	0.006749
3853	ENSG00000106524	ANKMY2	0.244805	4.412018	0.006778
3854	ENSG00000106615	RHEB	0.28949	5.282266	0.006786
3855	ENSG00000257150	PGAM1P5	0.410584	-0.24058	0.006787
3856	ENSG00000136573	BLK	-0.43411	4.680008	0.006798
3857	ENSG00000158882	TOMM40L	0.272065	3.759714	0.006815
3858	ENSG00000169129	AFAP1L2	0.411897	1.331307	0.006823
3859	ENSG00000187733	AMY1C	-0.40951	0.642561	0.006825
3860	ENSG00000119514	GALNT12	-0.3346	2.061515	0.006831
3861	ENSG00000153283	CD96	0.292014	7.967816	0.006833
3862	ENSG00000163545	NUAK2	-0.45126	4.082857	0.006834
3863	ENSG00000077463	SIRT6	0.259116	3.431489	0.006838
3864	ENSG00000172590	MRPL52	0.235895	4.312966	0.006863
3865	ENSG00000234289	H2BFS	0.365188	1.979273	0.006872
3866	ENSG00000105258	POLR2I	0.268159	3.222701	0.006889
3867	ENSG00000184887	BTBD6	0.188912	5.261164	0.006897
3868	ENSG00000245970		-0.31885	3.018027	0.006901
3869	ENSG00000203666	EFCAB2	-0.32122	1.94698	0.006901

3870	ENSG00000138640	FAM13A	-0.35794	4.205327	0.006907
3871	ENSG00000227408	AMYP1	-0.41993	0.26845	0.006914
3872	ENSG00000106948	AKNA	0.344013	8.856454	0.006926
3873	ENSG00000269343	ZNF587B	-0.30333	4.343632	0.006941
3874	ENSG00000227879	PSPC1P1	0.281788	2.783294	0.006969
3875	ENSG00000088256	GNA11	-0.4739	1.320768	0.006969
3876	ENSG00000213383		0.313661	1.475504	0.006972
3877	ENSG00000137806	NDUFAF1	0.462437	2.8052	0.00699
3878	ENSG00000257511		-0.18827	4.545111	0.006992
3879	ENSG00000256271	CACNA1C-AS2	-0.48662	0.766568	0.006998
3880	ENSG00000171530	TBCA	0.253753	5.701802	0.006999
3881	ENSG00000039523	FAM65A	-0.32566	5.011936	0.007002
3882	ENSG00000164512	ANKRD55	-0.63338	2.007921	0.007011
3883	ENSG00000164258	NDUFS4	0.242668	4.383577	0.007017
3884	ENSG00000164934	DCAF13	0.268296	5.413904	0.007028
3885	ENSG00000132535	DLG4	-0.47146	3.227989	0.007033
3886	ENSG00000261824	LINC00662	-0.3302	1.766909	0.007048
3887	ENSG00000102119	EMD	0.329204	6.311566	0.007052
3888	ENSG00000177989	ODF3B	0.586842	4.568665	0.007054
3889	ENSG00000105122	RASAL3	0.299912	6.571948	0.007054
3890	ENSG00000090975	PITPNM2	-0.3548	4.233941	0.007054
3891	ENSG00000171368	TPPP	-0.75258	1.671952	0.007054
3892	ENSG00000141425	RPRD1A	-0.23095	5.331862	0.007064
3893	ENSG00000117505	DR1	0.187079	7.272451	0.007098
3894	ENSG00000267243		-0.80304	-1.35425	0.007102
3895	ENSG00000077312	SNRPA	0.3578	5.201279	0.007117
3896	ENSG00000092529	CAPN3	-0.45538	0.711974	0.007117
3897	ENSG00000149534	MS4A2	-0.89979	0.324341	0.007117
3898	ENSG00000137871	ZNF280D	-0.29999	5.442531	0.007121
3899	ENSG00000151689	INPP1	0.327033	3.246698	0.007122
3900	ENSG00000172037	LAMB2	-0.87328	0.377601	0.007137
3901	ENSG00000188725	SMIM15	0.278524	5.243741	0.007139
3902	ENSG00000184831	APOO	0.274771	2.027612	0.00714
3903	ENSG00000164587	RPS14	-0.26772	10.36291	0.007148
3904	ENSG00000143315	PIGM	-0.30486	2.667643	0.007148
3905	ENSG00000099250	NRP1	-0.64573	0.852549	0.007149
3906	ENSG00000112232	KHDRBS2	-0.7009	-1.06845	0.007149
3907	ENSG00000157045	NTAN1	0.238384	5.076818	0.007154
3908	ENSG00000166529	ZSCAN21	0.223615	2.006943	0.007154
3909	ENSG00000166046	TCP11L2	-0.38687	5.353441	0.00717
3910	ENSG00000152926	ZNF117	-0.56669	3.931128	0.00717
3911	ENSG00000143436	MRPL9	0.164026	4.981028	0.007183
3912	ENSG00000154330	PGM5	-0.84985	0.000921	0.007183
3913	ENSG00000138336	TET1	0.608867	1.552876	0.007192

3914	ENSG00000139637	C12orf10	0.227922	4.292456	0.007195
3915	ENSG00000266356		0.414278	0.37343	0.0072
3916	ENSG00000214413	BBIP1	-0.25021	5.137537	0.0072
3917	ENSG00000102984	ZNF821	0.2774	2.903946	0.007232
3918	ENSG00000185839		0.296194	1.121522	0.007301
3919	ENSG00000261375		-0.85787	-1.19083	0.007301
3920	ENSG00000101811	CSTF2	0.263725	3.363646	0.007309
3921	ENSG00000143374	TARS2	0.199856	3.981546	0.007316
3922	ENSG00000151079	KCNA6	-0.90583	-1.3764	0.007316
3923	ENSG00000255545		-0.80252	-0.92298	0.007327
3924	ENSG00000115020	PIKFYVE	-0.24523	7.028346	0.007328
3925	ENSG00000228929		-0.27214	7.805274	0.007337
3926	ENSG00000225393		-0.74987	-1.06665	0.007345
3927	ENSG00000168243	GNG4	1.375856	-2.24391	0.007354
3928	ENSG00000103876	FAH	-0.39659	2.41245	0.007354
3929	ENSG00000238142		-0.51231	0.952957	0.007362
3930	ENSG00000007080	CCDC124	0.312597	4.013437	0.007372
3931	ENSG00000198796	ALPK2	-0.75041	-1.02969	0.007372
3932	ENSG00000221890	NPTXR	-0.49691	1.709607	0.007407
3933	ENSG00000131944	C19orf40	0.340674	0.928999	0.007426
3934	ENSG00000112234	FBXL4	-0.23535	4.787479	0.007426
3935	ENSG00000175066	GK5	0.383834	5.465806	0.007452
3936	ENSG00000100721	TCL1A	-0.92273	3.238734	0.007452
3937	ENSG00000166681	NGFRAP1	-0.41391	3.425343	0.007459
3938	ENSG00000150938	CRIM1	0.316121	3.866638	0.007471
3939	ENSG00000173409	ARV1	0.408122	3.049154	0.007484
3940	ENSG00000206052	DOK6	-0.47625	2.684493	0.007485
3941	ENSG00000163682	RPL9	-0.3124	9.994715	0.007522
3942	ENSG00000158683	PKD1L1	-0.61499	-0.95692	0.007522
3943	ENSG00000231154	MORF4L2-AS1	-0.54329	-0.51859	0.007535
3944	ENSG00000130772	MED18	0.415782	2.758119	0.007554
3945	ENSG00000259261	IGHV4OR15-8	1.049098	-0.67477	0.007555
3946	ENSG00000139116	KIF21A	0.374934	5.016153	0.007555
3947	ENSG00000164300	SERINC5	-0.40659	3.79122	0.007562
3948	ENSG00000242372	EIF6	0.250911	5.741595	0.007564
3949	ENSG00000156574	NODAL	-0.62045	-0.36208	0.007564
3950	ENSG00000105287	PRKD2	0.289648	6.034226	0.007568
3951	ENSG00000162616	DNAJB4	0.475377	3.486036	0.007573
3952	ENSG00000135048	TMEM2	0.688702	8.156835	0.007584
3953	ENSG00000143952	VPS54	0.167711	5.650845	0.007605
3954	ENSG00000187621	TCL6	-0.76361	1.031719	0.007615
3955	ENSG00000178381	ZFAND2A	0.212508	5.124474	0.007635
3956	ENSG00000182568	SATB1	-0.29217	7.800493	0.007641
3957	ENSG00000172922	RNASEH2C	0.21047	4.877083	0.007654

3958	ENSG00000065882	TBC1D1	0.210465	6.418296	0.00766
3959	ENSG00000267414		0.562845	-0.79629	0.007665
3960	ENSG00000163319	MRPS18C	0.327188	3.508215	0.007665
3961	ENSG00000165672	PRDX3	0.297659	6.387231	0.007668
3962	ENSG00000272686		-0.3673	1.234201	0.007671
3963	ENSG00000104852	SNRNP70	-0.21725	7.67519	0.007677
3964	ENSG00000002586	CD99	0.28637	6.212989	0.007678
3965	ENSG00000217643	PTGES3P2	0.418909	-0.16875	0.007687
3966	ENSG00000167657	DAPK3	0.281076	3.935849	0.007687
3967	ENSG00000236312	RPL34P34	-0.2945	4.381847	0.007687
3968	ENSG00000165115	KIF27	-0.37673	2.347866	0.007716
3969	ENSG00000177548	RABEP2	0.238832	4.373703	0.007727
3970	ENSG00000255769		-0.50965	-0.31632	0.007741
3971	ENSG00000156675	RAB11FIP1	-0.43291	6.90931	0.007747
3972	ENSG00000092203	TOX4	0.235217	6.293841	0.007758
3973	ENSG00000198522	GPN1	0.227105	5.713026	0.007774
3974	ENSG00000115091	ACTR3	0.260412	8.952062	0.007776
3975	ENSG00000148291	SURF2	0.237472	3.287995	0.007784
3976	ENSG00000197705	KLHL14	0.624933	2.303128	0.007804
3977	ENSG00000154227	CERS3	-0.72871	-0.25718	0.007805
3978	ENSG00000032742	IFT88	-0.23358	3.520931	0.007809
3979	ENSG00000157593	SLC35B2	0.215908	5.00594	0.007809
3980	ENSG00000100678	SLC8A3	-0.76211	-1.29204	0.007837
3981	ENSG00000214872	SMTNL1	0.566291	0.852228	0.007838
3982	ENSG00000205639	MFSD2B	0.430866	1.918909	0.007847
3983	ENSG00000136636	KCTD3	-0.53554	2.607113	0.007867
3984	ENSG00000214425	LRRC37A4P	-0.32024	5.174835	0.007868
3985	ENSG00000211810	TRAV29DV5	0.495144	1.407521	0.00787
3986	ENSG00000203705	TATDN3	0.319565	4.089528	0.007871
3987	ENSG00000110651	CD81	0.373041	6.771673	0.007878
3988	ENSG00000137185	ZSCAN9	-0.24306	2.871669	0.007898
3989	ENSG00000075391	RASAL2	-0.59083	0.006215	0.007912
3990	ENSG00000173163	COMMD1	0.207951	4.374759	0.007917
3991	ENSG00000162881	OXER1	-0.71265	0.090004	0.007959
3992	ENSG00000082153	BZW1	0.294049	8.89101	0.007966
3993	ENSG00000176092	AIM1L	-0.52536	0.482305	0.007967
3994	ENSG00000198561	CTNND1	-0.35021	4.920819	0.007972
3995	ENSG00000236144		-0.44731	2.600656	0.007972
3996	ENSG00000168439	STIP1	0.396297	5.859079	0.007994
3997	ENSG00000112110	MRPL18	0.278002	4.491808	0.007994
3998	ENSG00000135898	GPR55	0.494823	2.384512	0.007996
3999	ENSG00000083290	ULK2	-0.45457	4.90346	0.008
4000	ENSG00000243943	ZNF512	-0.37665	4.494364	0.008004
4001	ENSG00000179978		-0.5943	4.497056	0.008005

4002	ENSG00000185010	F8	0.394073	3.086204	0.008017
4003	ENSG00000154124	FAM105B	0.462824	6.878596	0.008021
4004	ENSG00000168772	CXXC4	-0.96942	-1.94475	0.008022
4005	ENSG00000149573	MPZL2	-0.63844	1.466822	0.008023
4006	ENSG00000109956	B3GAT1	-0.51976	3.833247	0.008058
4007	ENSG00000164398	ACSL6	-0.32864	4.082505	0.008079
4008	ENSG00000142168	SOD1	0.211298	6.915603	0.008122
4009	ENSG00000128654	MTX2	0.26919	3.493059	0.008124
4010	ENSG00000136840	ST6GALNAC4	0.342413	3.870462	0.008139
4011	ENSG00000203896	LIME1	0.36633	1.639538	0.008139
4012	ENSG00000232385	RPS3AP25	-0.37315	1.094058	0.008139
4013	ENSG00000204745		0.150368	4.367265	0.008143
4014	ENSG00000132305	IMMT	0.204306	6.190226	0.008148
4015	ENSG00000100095	SEZ6L	-0.8175	0.402627	0.008177
4016	ENSG00000166900	STX3	-0.46094	4.56015	0.008184
4017	ENSG00000144959	NCEH1	-0.43187	3.639862	0.008191
4018	ENSG00000125971	DYNLRB1	0.264612	5.468924	0.008193
4019	ENSG00000173599	PC	-0.42806	1.105073	0.008197
4020	ENSG00000130159	ECSIT	0.240827	4.367971	0.008199
4021	ENSG00000101945	SUV39H1	0.291767	3.011121	0.008221
4022	ENSG00000111737	RAB35	0.261382	6.147429	0.008225
4023	ENSG00000175213	ZNF408	0.290922	3.331771	0.008241
4024	ENSG00000130733	YIPF2	0.203476	3.527682	0.008241
4025	ENSG00000101224	CDC25B	0.287089	7.103347	0.008271
4026	ENSG00000178053	MLF1	-0.62274	1.480718	0.008271
4027	ENSG00000131381	ZFYVE20	-0.20603	5.379314	0.008272
4028	ENSG00000261015		-0.40036	1.09226	0.008275
4029	ENSG00000236829		-0.41387	0.314993	0.008275
4030	ENSG00000143158	MPC2	0.212015	4.897461	0.00829
4031	ENSG00000165434	PGM2L1	-0.38797	4.27326	0.008292
4032	ENSG00000244153	WWP1P1	-0.24093	2.418062	0.008292
4033	ENSG00000104131	EIF3J	0.313105	5.963121	0.008304
4034	ENSG00000175203	DCTN2	0.195151	5.891358	0.008306
4035	ENSG00000212789	ST13P5	-0.27321	1.188081	0.008331
4036	ENSG00000123552	USP45	-0.29382	4.00026	0.008334
4037	ENSG00000213104	NPM1P46	0.305272	4.301452	0.008336
4038	ENSG00000139351	SYCP3	-0.49585	-0.54757	0.008336
4039	ENSG00000160961	ZNF333	-0.22905	4.723799	0.008349
4040	ENSG00000182150	ERCC6L2	-0.24253	5.005595	0.008349
4041	ENSG00000005513	SOX8	-0.71977	-0.53073	0.008349
4042	ENSG00000144455	SUMF1	-0.39242	4.251439	0.008368
4043	ENSG00000168300	PCMTD1	-0.2787	6.123322	0.008374
4044	ENSG00000177432	NAP1L5	0.428821	3.929303	0.008388
4045	ENSG00000166763	STRCP1	-0.54674	-0.51139	0.008388

4046	ENSG00000151553	FAM160B1	0.376286	6.797392	0.008407
4047	ENSG00000183943	PRKX	0.326168	7.077682	0.008407
4048	ENSG00000244649		0.65303	0.788242	0.008436
4049	ENSG00000184602	SNN	-0.32035	7.251968	0.008437
4050	ENSG00000184992	BRI3BP	0.34536	4.608506	0.008469
4051	ENSG00000051382	PIK3CB	-0.24744	5.296329	0.008486
4052	ENSG00000165046	LETM2	0.514791	1.998292	0.008489
4053	ENSG00000082898	XPO1	0.313861	7.504479	0.008489
4054	ENSG00000204152	TIMM23B	0.290764	3.264376	0.008505
4055	ENSG00000233913		-0.24859	5.130994	0.008516
4056	ENSG00000134352	IL6ST	-0.37062	7.873177	0.008522
4057	ENSG00000112699	GMDS	0.303008	3.303485	0.00854
4058	ENSG00000011485	PPP5C	0.329668	5.076258	0.008552
4059	ENSG00000139722	VPS37B	0.514113	6.551877	0.008569
4060	ENSG00000230330	HMGN2P3	0.566083	-0.54144	0.008607
4061	ENSG00000066923	STAG3	0.278286	3.071823	0.008612
4062	ENSG00000071246	VASH1	-0.47352	4.224703	0.008618
4063	ENSG00000153113	CAST	-0.20424	8.211133	0.008633
4064	ENSG00000260382		0.410476	-0.52145	0.008645
4065	ENSG00000129696	TTI2	0.30538	2.580557	0.008653
4066	ENSG00000035141	FAM136A	0.166917	5.362224	0.008653
4067	ENSG00000197822	OCLN	-0.68096	2.22626	0.008653
4068	ENSG00000198453	ZNF568	-0.29717	2.95085	0.008666
4069	ENSG00000213443		0.283891	5.76423	0.008693
4070	ENSG00000162594	IL23R	-0.8	-0.81621	0.008699
4071	ENSG00000226200		-0.29883	2.422645	0.008711
4072	ENSG00000123191	ATP7B	-0.36059	-0.02984	0.008741
4073	ENSG00000100983	GSS	0.168775	4.610657	0.008746
4074	ENSG00000119684	MLH3	-0.24593	4.529655	0.008756
4075	ENSG00000261064		-0.55585	0.200164	0.008766
4076	ENSG00000233954		0.463895	1.611689	0.008802
4077	ENSG00000128340	RAC2	0.379781	8.519102	0.008827
4078	ENSG00000157193	LRP8	0.215819	3.77443	0.008842
4079	ENSG00000125354	SEPT6	0.191052	8.0109	0.008842
4080	ENSG00000011021	CLCN6	-0.20266	4.574618	0.008842
4081	ENSG00000100883	SRP54	0.343882	5.729244	0.008853
4082	ENSG00000241657	TRBV11-2	0.537319	2.001631	0.008861
4083	ENSG00000113269	RNF130	-0.43721	7.12751	0.008888
4084	ENSG00000254449	SF3A3P2	0.216284	2.383728	0.008892
4085	ENSG00000188626	GOLGA8M	-0.57574	0.636237	0.00892
4086	ENSG00000109667	SLC2A9	-0.6487	2.150277	0.008938
4087	ENSG00000167799	NUDT8	0.536545	-0.46816	0.008984
4088	ENSG00000124549	BTN2A3P	0.298517	2.184046	0.008984
4089	ENSG00000123453	SARDH	-0.62517	0.262699	0.008999

4090	ENSG00000254454	RCC2P6	0.381466	0.983655	0.009001
4091	ENSG00000125122	LRRC29	-0.40428	0.420567	0.009004
4092	ENSG00000138744	NAAA	-0.45637	6.384254	0.009011
4093	ENSG00000231822		0.257502	2.959317	0.009025
4094	ENSG00000171105	INSR	-0.46943	3.4684	0.009059
4095	ENSG00000269751		-0.37595	1.742728	0.009074
4096	ENSG00000175538	KCNE3	-0.67648	3.830904	0.009075
4097	ENSG00000087191	PSMC5	0.204761	6.407623	0.009088
4098	ENSG00000213866	YBX1P10	0.306022	3.203702	0.009101
4099	ENSG00000170469	SPATA24	0.284526	0.788283	0.009116
4100	ENSG00000267046		0.58495	1.607	0.009131
4101	ENSG00000197497	ZNF665	-0.56531	-0.08898	0.009137
4102	ENSG00000258521		0.782775	0.332136	0.009152
4103	ENSG00000213673	SLC25A5P3	0.387713	0.350051	0.009153
4104	ENSG00000163947	ARHGEF3	0.270829	7.210042	0.009153
4105	ENSG00000260318	COX6CP1	0.417718	2.033557	0.009177
4106	ENSG00000229994	RPL5P4	-0.33986	1.486831	0.009182
4107	ENSG00000120053	GOT1	0.327435	3.503459	0.009198
4108	ENSG00000188076	SCGB1C1	-0.93917	-1.50085	0.009198
4109	ENSG00000163872	YEATS2	0.275571	5.225549	0.009199
4110	ENSG00000110700	RPS13	-0.24318	9.531846	0.00921
4111	ENSG00000138297	TIMM23	0.284377	4.980872	0.009225
4112	ENSG00000139174	PRICKLE1	-0.64663	-0.38156	0.00923
4113	ENSG00000101265	RASSF2	-0.2895	8.026821	0.00924
4114	ENSG00000108852	MPP2	-0.70627	-0.9808	0.009267
4115	ENSG00000244021		-0.30411	6.54116	0.009269
4116	ENSG00000134333	LDHA	0.50815	8.988895	0.009276
4117	ENSG00000101189	MRGBP	0.198577	4.252321	0.009279
4118	ENSG00000105700	KXD1	0.15668	5.366562	0.009284
4119	ENSG00000185689	C6orf201	-0.58228	-0.80062	0.009284
4120	ENSG00000249193	HSPD1P5	0.602965	-0.70806	0.009286
4121	ENSG00000130520	LSM4	0.227283	5.350328	0.009286
4122	ENSG00000078804	TP53INP2	0.586168	5.58294	0.009299
4123	ENSG00000107949	BCCIP	0.211319	5.525881	0.009313
4124	ENSG00000116604	MEF2D	0.331914	7.213704	0.009333
4125	ENSG00000267809	NDUFV2P1	0.467789	0.519975	0.009334
4126	ENSG00000248873	SERBP1P6	0.271848	2.905989	0.009334
4127	ENSG00000117242	PINK1-AS	-0.25383	2.274714	0.009336
4128	ENSG00000082213	C5orf22	0.256885	4.70048	0.009359
4129	ENSG00000118690	ARMC2	0.343569	2.554357	0.009376
4130	ENSG00000168216	LMBRD1	-0.16545	6.121952	0.00941
4131	ENSG00000187535	IFT140	-0.32114	1.932481	0.00941
4132	ENSG00000115525	ST3GAL5	0.343528	6.195219	0.009416
4133	ENSG00000205085	FAM71F2	-0.53096	-0.81456	0.009425

4134	ENSG00000245498		-0.54914	-0.21656	0.009427
4135	ENSG00000273045		-0.42942	0.087946	0.009437
4136	ENSG00000123575	FAM199X	-0.1667	6.677141	0.009446
4137	ENSG00000262745		0.726904	-1.1272	0.009475
4138	ENSG00000106244	PDAP1	0.206863	5.662016	0.00949
4139	ENSG00000163406	SLC15A2	-0.52067	2.940346	0.00949
4140	ENSG00000142233	NTN5	-0.55246	-0.29573	0.00949
4141	ENSG00000123500	COL10A1	-0.65564	-0.363	0.009512
4142	ENSG00000198925	ATG9A	0.288675	5.821616	0.009532
4143	ENSG00000107758	PPP3CB	0.184392	6.170625	0.009535
4144	ENSG00000078140	UBE2K	0.133619	6.639682	0.009535
4145	ENSG00000221968	FADS3	-0.49408	3.213589	0.00955
4146	ENSG00000159899	NPR2	-0.40453	0.607906	0.009552
4147	ENSG00000230667	SETSIP	0.311981	0.942458	0.009568
4148	ENSG00000099624	ATP5D	0.371821	4.284042	0.009596
4149	ENSG00000139546	TARBP2	0.188543	4.015542	0.009596
4150	ENSG00000173372	C1QA	0.938798	3.376151	0.009605
4151	ENSG00000181045	SLC26A11	-0.32023	4.221115	0.009611
4152	ENSG00000225496		0.275046	0.941827	0.009635
4153	ENSG00000173041	ZNF680	-0.3118	3.253024	0.009635
4154	ENSG00000153162	BMP6	-0.54442	1.96523	0.009652
4155	ENSG00000213409		0.26412	1.139729	0.009667
4156	ENSG00000140199	SLC12A6	-0.32708	6.198175	0.009674
4157	ENSG00000178685	PARP10	0.473738	5.875933	0.009678
4158	ENSG00000123989	CHPF	0.506166	2.109893	0.009684
4159	ENSG00000135999	EPC2	-0.2068	6.370414	0.00969
4160	ENSG00000169188	APEX2	0.278852	3.984739	0.00969
4161	ENSG00000104763	ASAH1	-0.32878	8.33075	0.009698
4162	ENSG00000257043		0.620904	0.194012	0.009721
4163	ENSG00000268912		-0.69372	-1.06717	0.009724
4164	ENSG00000196924	FLNA	0.393896	10.42837	0.009746
4165	ENSG00000223704		-0.59609	0.677203	0.009746
4166	ENSG00000212978		0.46779	1.334209	0.009747
4167	ENSG00000079246	XRCC5	0.186931	8.554783	0.009761
4168	ENSG00000180964	TCEAL8	0.251182	4.396773	0.009775
4169	ENSG00000136521	NDUFB5	0.241398	5.649409	0.009778
4170	ENSG00000141568	FOXK2	0.237371	5.269471	0.009781
4171	ENSG00000156471	PTDSS1	-0.16272	6.708908	0.009813
4172	ENSG00000173818	ENDOV	-0.21837	2.395171	0.009822
4173	ENSG00000184925	LCN12	0.620932	-0.4981	0.009825
4174	ENSG00000264098		-0.35626	1.626053	0.009826
4175	ENSG00000077684	JADE1	-0.17952	5.938958	0.009829
4176	ENSG00000273456		-0.55508	-0.75267	0.00983
4177	ENSG00000105607	GCDH	0.215312	3.931845	0.009844

4178	ENSG00000127366	TAS2R5	-0.65402	-0.57528	0.009875
4179	ENSG00000204397	CARD16	0.35479	6.209951	0.009879
4180	ENSG00000155229	MMS19	-0.15345	5.796994	0.009888
4181	ENSG00000170382	LRRN2	-0.47153	2.41733	0.009893
4182	ENSG00000132965	ALOX5AP	0.285691	6.395909	0.009895
4183	ENSG00000257499		0.271312	1.846684	0.009895
4184	ENSG00000213782	DDX47	-0.38929	3.949481	0.009902
4185	ENSG00000247373		0.43701	-0.43875	0.009905
4186	ENSG00000223511		0.536121	0.994362	0.009932
4187	ENSG00000197530	MIB2	0.350049	5.188036	0.009932
4188	ENSG00000188811	NHLRC3	-0.25274	5.239784	0.009932
4189	ENSG00000100220	RTCB	0.253243	5.932493	0.009937
4190	ENSG00000118276	B4GALT6	-0.43695	1.421173	0.009955
4191	ENSG00000073350	LLGL2	0.28076	5.353022	0.009968
4192	ENSG00000102241	HTATSF1	0.155914	6.068722	0.009976
4193	ENSG00000258150		-0.33317	0.888242	0.009976
4194	ENSG00000107281	NPDC1	0.42239	3.758683	0.009979
4195	ENSG00000182518	FAM104B	0.274249	2.536069	0.009996
4196	ENSG00000162241	SLC25A45	-0.35136	4.64077	0.009996
4197	ENSG00000182240	BACE2	-0.48677	1.682266	0.009996
4198	ENSG00000165406	MARCH8	-0.25225	6.422433	0.010009
4199	ENSG00000260727	SLC7A5P1	0.903923	-1.08217	0.010027
4200	ENSG00000153933	DGKE	-0.23518	5.409228	0.010027
4201	ENSG00000188342	GTF2F2	0.238212	4.399785	0.010061
4202	ENSG00000145743	FBXL17	-0.24984	4.789934	0.010075
4203	ENSG00000071127	WDR1	0.241091	8.493477	0.010079
4204	ENSG00000228889	UBAC2-AS1	0.508767	-0.44201	0.010097
4205	ENSG00000256235	SMIM3	-0.4169	4.299519	0.010097
4206	ENSG00000119953	SMNDC1	0.182124	5.864565	0.010116
4207	ENSG00000102125	TAZ	-0.18606	5.343543	0.010118
4208	ENSG00000260400		0.785621	-0.24914	0.010127
4209	ENSG00000106236	NPTX2	1.315484	-1.51031	0.010149
4210	ENSG00000188158	NHS	-0.90171	0.908841	0.010169
4211	ENSG00000124721	DNAH8	0.55296	0.824069	0.010177
4212	ENSG00000227124	ZNF717	-0.44479	1.947554	0.01019
4213	ENSG00000229151		-0.73078	-1.54468	0.010213
4214	ENSG00000126254	RBM42	0.330688	4.491615	0.010224
4215	ENSG00000110777	POU2AF1	0.561802	4.355179	0.010252
4216	ENSG00000177150	FAM210A	0.403317	4.028503	0.010252
4217	ENSG00000099338	CATSPERG	-0.31874	1.301544	0.010285
4218	ENSG00000205221	VIT	1.116425	-1.53399	0.010289
4219	ENSG00000143799	PARP1	0.390459	6.646321	0.010289
4220	ENSG00000115325	DOK1	0.277056	4.160326	0.010289
4221	ENSG00000038274	MAT2B	0.268666	7.338949	0.010289

4222	ENSG00000041988	THAP3	0.252249	3.139479	0.01029
4223	ENSG00000090376	IRAK3	-0.41006	6.815095	0.010301
4224	ENSG00000101843	PSMD10	0.191113	5.172518	0.010324
4225	ENSG00000100359	SGSM3	-0.23741	5.588141	0.010331
4226	ENSG00000261740		0.27381	2.58484	0.010333
4227	ENSG00000184584	TMEM173	-0.31914	5.830917	0.010346
4228	ENSG00000058272	PPP1R12A	0.171145	7.450088	0.010357
4229	ENSG00000214367	HAUS3	0.389142	6.290072	0.010357
4230	ENSG00000104812	GYS1	0.315042	4.500622	0.010358
4231	ENSG00000255717	SNHG1	-0.30032	7.257703	0.010358
4232	ENSG00000234274	COX7BP2	0.89865	-0.89822	0.010379
4233	ENSG00000105520		-0.44786	4.054514	0.010381
4234	ENSG00000221978	CCNL2	-0.20652	7.208501	0.010384
4235	ENSG00000219755		-0.28498	4.389845	0.010384
4236	ENSG00000143353	LYPLAL1	-0.27086	3.765216	0.010397
4237	ENSG00000089094	KDM2B	0.296261	6.192865	0.010401
4238	ENSG00000084092	NOA1	0.161245	4.761538	0.010413
4239	ENSG00000183621	ZNF438	-0.29181	3.643484	0.010422
4240	ENSG00000134668	SPOCD1	-0.85416	0.883259	0.010435
4241	ENSG00000168066	SF1	-0.23223	8.426001	0.010447
4242	ENSG00000267544		0.478719	0.032787	0.010486
4243	ENSG00000164050	PLXNB1	-0.36321	0.537218	0.010505
4244	ENSG00000120594	PLXDC2	-0.47002	6.24071	0.010506
4245	ENSG00000138035	PNPT1	0.341921	5.044215	0.01051
4246	ENSG00000169896	ITGAM	-0.50667	7.053774	0.01051
4247	ENSG00000141934	PPAP2C	0.879834	-1.4968	0.010555
4248	ENSG00000164815	ORC5	0.258272	3.750264	0.010555
4249	ENSG00000186448	ZNF197	0.241784	3.943602	0.010555
4250	ENSG00000185291	IL3RA	-0.52876	3.359482	0.010555
4251	ENSG00000177599	ZNF491	-0.55325	-0.16442	0.010555
4252	ENSG00000188056	TREML4	-0.75835	0.877885	0.010555
4253	ENSG00000214562	NUTM2D	-0.32866	1.143122	0.01056
4254	ENSG00000178732	GP5	-0.55238	0.657498	0.010564
4255	ENSG00000120833	SOCS2	-0.48653	4.09541	0.010565
4256	ENSG00000233012	HDAC1P2	0.374264	0.32352	0.010566
4257	ENSG00000078081	LAMP3	0.781712	1.976704	0.010568
4258	ENSG00000198237		-0.36581	0.898205	0.010572
4259	ENSG00000254690		-0.38931	-0.02221	0.010574
4260	ENSG00000147140	NONO	0.131027	8.030666	0.01059
4261	ENSG00000240622	RPL7P15	-0.27697	5.522905	0.01059
4262	ENSG00000104218	CSPP1	-0.16123	4.127355	0.010593
4263	ENSG00000144645	OSBPL10	-0.50275	3.342408	0.010593
4264	ENSG00000130638	ATXN10	0.185215	6.014943	0.010608
4265	ENSG00000138600	SPPL2A	0.265886	6.221048	0.010611

4266	ENSG00000040199	PHLPP2	-0.2424	4.771391	0.010617
4267	ENSG00000189283	FHIT	-0.50986	4.056233	0.01062
4268	ENSG00000143995	MEIS1	-0.62686	1.865394	0.01064
4269	ENSG00000261584		-0.65015	0.309909	0.01065
4270	ENSG00000105229	PIAS4	0.247359	3.549193	0.010663
4271	ENSG00000151789	ZNF385D	-0.87471	-1.08669	0.010679
4272	ENSG00000105767	CADM4	-0.4572	-0.0153	0.010681
4273	ENSG00000240489	SETP14	0.204938	2.037021	0.010685
4274	ENSG00000147403	RPL10	-0.22565	10.96101	0.010699
4275	ENSG00000077514	POLD3	0.231109	4.362262	0.010712
4276	ENSG00000184083	FAM120C	-0.23587	2.985806	0.010719
4277	ENSG00000172757	CFL1	0.354552	9.233316	0.010735
4278	ENSG00000157600	TMEM164	-0.31685	5.811605	0.010746
4279	ENSG00000100027	YPEL1	0.337126	3.305106	0.01075
4280	ENSG00000129484	PARP2	0.203253	3.36453	0.01075
4281	ENSG00000172270	BSG	0.345566	6.918994	0.010765
4282	ENSG00000183439	TRIM61	0.555899	-0.6494	0.010793
4283	ENSG00000113790	EHHADH	0.563407	-0.28379	0.010808
4284	ENSG00000120875	DUSP4	1.000171	5.434659	0.010809
4285	ENSG00000158122	AAED1	-0.3154	4.305604	0.010814
4286	ENSG00000104885	DOT1L	0.316429	5.108562	0.01082
4287	ENSG00000104635	SLC39A14	0.295493	3.173188	0.010826
4288	ENSG00000108861	DUSP3	-0.37146	5.170457	0.010826
4289	ENSG00000156411	C14orf2	0.255228	6.033649	0.010859
4290	ENSG00000123353	ORMDL2	0.27234	3.601476	0.010867
4291	ENSG00000143847	PPFIA4	-0.75334	-0.13743	0.010867
4292	ENSG00000103184	SEC14L5	-0.86708	-0.16632	0.010867
4293	ENSG00000141642	ELAC1	0.267	2.411332	0.010894
4294	ENSG00000165801	ARHGEF40	-0.71703	4.299793	0.010894
4295	ENSG00000105717	PBX4	-0.50764	3.068006	0.010945
4296	ENSG00000059691	PET112	0.30474	3.207673	0.010951
4297	ENSG00000174799	CEP135	-0.22457	4.963329	0.010951
4298	ENSG00000135185	TMEM243	-0.22628	5.494849	0.010951
4299	ENSG00000103528	SYT17	-0.59444	1.147014	0.010951
4300	ENSG00000124641	MED20	0.382439	3.090534	0.010955
4301	ENSG00000164053	ATRIP	0.222501	2.687335	0.010955
4302	ENSG00000115286	NDUFS7	0.281581	4.097452	0.010969
4303	ENSG00000221817		-0.30358	3.619154	0.011015
4304	ENSG00000229474	PATL2	0.366527	5.824625	0.011025
4305	ENSG00000214552	COPS8P2	0.280271	1.218726	0.011025
4306	ENSG00000136908	DPM2	0.296296	4.201125	0.011037
4307	ENSG00000148225	WDR31	-0.75303	-1.2925	0.011046
4308	ENSG00000177311	ZBTB38	0.255167	7.097277	0.011054
4309	ENSG00000112697	TMEM30A	-0.15084	7.803588	0.011084

4310	ENSG00000162882	HAAO	-0.5856	0.875129	0.011105
4311	ENSG00000197549	PRAMENP	-0.75586	-1.43539	0.011105
4312	ENSG00000273179		0.513209	1.3437	0.011124
4313	ENSG00000197599	CCDC154	-0.46647	0.706656	0.011129
4314	ENSG00000135801	TAF5L	0.232356	5.460952	0.011129
4315	ENSG00000113356	POLR3G	0.321804	1.899458	0.011135
4316	ENSG00000182957	SPATA13	0.211306	7.810986	0.011135
4317	ENSG00000108395	TRIM37	0.167257	4.927361	0.011135
4318	ENSG00000271680		-0.68814	-0.10615	0.011135
4319	ENSG00000221994	ZNF630	-0.3521	1.171238	0.011206
4320	ENSG00000159714	ZDHHC1	-0.7551	0.195607	0.011208
4321	ENSG00000185298	CCDC137	0.204976	3.992512	0.011252
4322	ENSG00000225523	IGKV6D-21	1.002088	-0.51866	0.011273
4323	ENSG00000258033		0.267145	0.595012	0.011312
4324	ENSG00000163322	FAM175A	-0.26962	3.695957	0.011312
4325	ENSG00000147383	NSDHL	0.221469	3.054403	0.011312
4326	ENSG00000213051	RPL5P5	-0.29838	2.428291	0.011312
4327	ENSG00000082781	ITGB5	-0.60826	2.844148	0.011312
4328	ENSG00000143222	UFC1	0.177604	6.654126	0.011313
4329	ENSG00000232208		-0.6808	-0.78776	0.011316
4330	ENSG00000253982		-0.39643	2.819485	0.011334
4331	ENSG00000012779	ALOX5	-0.46459	6.170537	0.011336
4332	ENSG00000106484	MEST	-0.39189	1.747287	0.011361
4333	ENSG00000135423	GLS2	-0.41952	1.96722	0.011364
4334	ENSG00000206417	H1FX-AS1	0.389445	-0.40885	0.011398
4335	ENSG00000076356	PLXNA2	-0.37784	1.831462	0.011418
4336	ENSG00000153130	SCOC	-0.26941	4.737122	0.011425
4337	ENSG00000087303	NID2	-0.35087	2.005585	0.011432
4338	ENSG00000115107	STEAP3	-0.77963	1.120227	0.011448
4339	ENSG00000151690	MFSD6	0.256444	6.0297	0.011449
4340	ENSG00000271870		-0.35514	2.195651	0.01148
4341	ENSG00000213047	DENND1B	0.290549	6.136305	0.011488
4342	ENSG00000174206	C12orf66	-0.20956	2.982063	0.011488
4343	ENSG00000160917	CPSF4	0.139936	4.694303	0.011519
4344	ENSG00000114902	SPCS1	0.180146	6.175858	0.011578
4345	ENSG00000197284		-0.97202	0.590958	0.011578
4346	ENSG00000155380	SLC16A1	0.608075	4.911849	0.011584
4347	ENSG00000249684		-0.59684	0.086096	0.011586
4348	ENSG00000107175	CREB3	0.230477	4.40823	0.011593
4349	ENSG00000174851	YIF1A	0.334367	3.659969	0.011599
4350	ENSG00000088247	KHSRP	0.261634	6.11681	0.011614
4351	ENSG00000231333	RPL34P6	-0.33783	1.041549	0.011614
4352	ENSG00000214787	MS4A4E	-0.78262	0.153608	0.011614
4353	ENSG00000023171	GRAMD1B	0.426934	4.646241	0.011622

4354	ENSG00000128656	CHN1	-0.59697	2.05813	0.011622
4355	ENSG00000233476	EEF1A1P6	-0.23249	7.674305	0.011623
4356	ENSG00000230795	HLA-K	-0.789	-0.68421	0.011631
4357	ENSG00000154957	ZNF18	0.198026	4.213566	0.011638
4358	ENSG00000108691	CCL2	1.668729	3.555855	0.011647
4359	ENSG00000261278		0.543042	-0.08885	0.011659
4360	ENSG00000259834		-0.28857	6.179559	0.011671
4361	ENSG00000155906	RMND1	0.227963	3.173122	0.011679
4362	ENSG00000099814	CEP170B	-0.52316	0.236075	0.011718
4363	ENSG00000138101	DTNB	0.185598	3.319499	0.011729
4364	ENSG00000133105	RXFP2	-1.01044	-1.27317	0.011734
4365	ENSG00000133789	SWAP70	-0.38787	5.886844	0.011737
4366	ENSG00000150459	SAP18	0.162612	6.896644	0.011763
4367	ENSG00000220749	RPL21P28	-0.27642	3.99774	0.011763
4368	ENSG00000100077	ADRBK2	-0.3325	6.449805	0.011773
4369	ENSG00000108523	RNF167	0.296791	6.6214	0.011825
4370	ENSG00000166971	AKTIP	-0.21531	4.796012	0.011825
4371	ENSG00000239470		-0.27076	3.813622	0.01184
4372	ENSG00000243910	TUBA4B	0.463353	2.680379	0.011848
4373	ENSG00000196544	C17orf59	0.325881	3.306064	0.011848
4374	ENSG00000130734	ATG4D	0.25179	4.269966	0.011848
4375	ENSG00000239900	ADSL	0.166498	5.910656	0.011848
4376	ENSG00000238228	OR7E7P	-0.54952	-0.51902	0.011866
4377	ENSG00000100181	TPTEP1	-0.6004	3.073914	0.011894
4378	ENSG00000123136	DDX39A	0.211042	6.882409	0.011912
4379	ENSG00000204311	DFNB59	-0.43607	-0.0238	0.011994
4380	ENSG00000213281	NRAS	0.233247	7.6475	0.012012
4381	ENSG00000188807	TMEM201	0.332206	2.662382	0.012023
4382	ENSG00000102901	CENPT	-0.17649	5.203634	0.012023
4383	ENSG00000100056	DGCR14	0.2552	4.474604	0.012025
4384	ENSG00000214114	MYCBP	0.273549	4.977409	0.012049
4385	ENSG00000188895	MSL1	-0.27027	7.024836	0.012052
4386	ENSG00000134369	NAV1	-0.40722	2.674417	0.012062
4387	ENSG00000239911	PRKAG2-AS1	-0.40768	1.322806	0.012076
4388	ENSG00000160185	UBASH3A	0.347025	4.486609	0.012112
4389	ENSG00000149564	ESAM	-0.55997	2.451494	0.012114
4390	ENSG00000215912	TTC34	-0.80133	0.435074	0.012117
4391	ENSG00000088682	COQ9	0.244538	3.95186	0.012127
4392	ENSG00000181666	HKR1	-0.21382	4.551998	0.012132
4393	ENSG00000258461		-0.44723	0.950651	0.012141
4394	ENSG00000272098		-0.54435	2.858761	0.012172
4395	ENSG00000196323	ZBTB44	-0.19403	7.347623	0.012188
4396	ENSG00000135503	ACVR1B	-0.39457	4.146921	0.012208
4397	ENSG00000172460	PRSS30P	-0.69107	0.748639	0.012228

4398	ENSG00000255569	TRAV1-1	0.602525	0.12641	0.012232
4399	ENSG00000269399		0.535647	-0.25125	0.012233
4400	ENSG00000223855		-0.72438	0.894653	0.012236
4401	ENSG00000023572	GLRX2	0.269405	1.777071	0.012247
4402	ENSG00000254858	MPV17L2	0.26831	2.235721	0.012317
4403	ENSG00000132635	PCED1A	-0.24056	4.913099	0.012317
4404	ENSG00000138606	SHF	-0.57842	-0.06981	0.012317
4405	ENSG00000166750	SLFN5	0.405117	8.129445	0.012325
4406	ENSG00000083845	RPS5	-0.24444	9.300901	0.012335
4407	ENSG00000078237	C12orf5	0.343059	4.466878	0.012348
4408	ENSG00000006744	ELAC2	0.148326	5.314039	0.012355
4409	ENSG00000145945	FAM50B	0.387078	0.899312	0.012367
4410	ENSG00000029534	ANK1	-0.53933	2.712398	0.012392
4411	ENSG00000166337	TAF10	0.162752	5.960197	0.012398
4412	ENSG00000058453	CROCC	-0.26779	4.689096	0.012403
4413	ENSG00000139679	LPAR6	-0.38146	6.517595	0.012418
4414	ENSG00000112799	LY86	-0.51965	4.988248	0.012434
4415	ENSG00000144231	POLR2D	0.191579	4.434878	0.012439
4416	ENSG00000116489	CAPZA1	0.356872	8.689365	0.012454
4417	ENSG00000196123	KIAA0895L	0.255028	2.647145	0.012465
4418	ENSG00000242100	RPL9P32	-0.30232	6.210334	0.012477
4419	ENSG00000174469	CNTNAP2	-1.09386	-0.68094	0.012491
4420	ENSG00000253760		0.517141	-0.51322	0.012499
4421	ENSG00000130725	UBE2M	0.268334	4.734167	0.012516
4422	ENSG00000135679	MDM2	0.182854	6.432975	0.012564
4423	ENSG00000165410	CFL2	-0.35095	4.037898	0.012585
4424	ENSG00000090061	CCNK	0.251136	5.921129	0.012586
4425	ENSG00000010270	STARD3NL	0.206329	5.60287	0.012586
4426	ENSG00000211777	TRAV3	0.51334	1.279278	0.01259
4427	ENSG00000172123	SLFN12	0.309527	4.059255	0.01259
4428	ENSG00000183569	SERHL2	-0.32196	0.602325	0.01259
4429	ENSG00000263563	UBBP4	0.331865	6.951722	0.012607
4430	ENSG00000108654	DDX5	-0.16662	11.34141	0.012607
4431	ENSG00000165209	STRBP	-0.27371	4.585159	0.012607
4432	ENSG00000167863	ATP5H	0.218837	6.237707	0.012628
4433	ENSG00000206560	ANKRD28	-0.22573	6.139878	0.01263
4434	ENSG00000265206	MIR142	0.372041	4.735096	0.012636
4435	ENSG00000085788	DDHD2	-0.18331	5.952166	0.012636
4436	ENSG00000141646	SMAD4	-0.22932	6.422768	0.012642
4437	ENSG00000211779	TRAV5	0.563756	0.531701	0.012689
4438	ENSG00000054282	SDCCAG8	-0.19941	4.336692	0.012693
4439	ENSG00000255455		-0.30073	3.400121	0.012698
4440	ENSG00000100138	NHP2L1	0.207787	6.754692	0.012699
4441	ENSG00000118292	C1orf54	-0.26997	1.673321	0.012717

4442	ENSG00000204370	SDHD	0.196269	6.262903	0.012748
4443	ENSG00000081665	ZNF506	-0.3559	4.856446	0.012749
4444	ENSG00000088356	PDRG1	0.246789	3.910443	0.012782
4445	ENSG00000260784		-0.77558	-1.20408	0.012789
4446	ENSG00000134460	IL2RA	-0.44783	3.270716	0.012815
4447	ENSG00000133059	DSTYK	-0.21859	5.3085	0.012816
4448	ENSG00000105514	RAB3D	-0.73066	3.660683	0.012829
4449	ENSG00000119559	C19orf25	0.289821	3.891035	0.012856
4450	ENSG00000119681	LTBP2	-0.32003	0.884806	0.012856
4451	ENSG00000139970	RTN1	-0.78679	2.837111	0.012856
4452	ENSG00000114209	PDCD10	0.242867	5.578964	0.012859
4453	ENSG00000106123	EPHB6	-0.43993	4.242009	0.012868
4454	ENSG00000179909	ZNF154	-0.55605	3.23033	0.012872
4455	ENSG00000128891	C15orf57	0.192613	4.119756	0.012878
4456	ENSG00000255310		-0.32383	2.516683	0.012878
4457	ENSG00000160208	RRP1B	0.222766	5.568381	0.012897
4458	ENSG00000086827	ZW10	0.362079	3.887879	0.012913
4459	ENSG00000130559	CAMSAP1	-0.2516	4.931691	0.012919
4460	ENSG00000213658	LAT	0.347813	3.071208	0.012926
4461	ENSG00000197459	HIST1H2BH	0.571012	0.65662	0.012933
4462	ENSG00000163528	CHCHD4	0.315995	2.57655	0.012934
4463	ENSG00000103187	COTL1	0.35732	9.247472	0.012936
4464	ENSG00000168781	PPIP5K1	-0.23344	3.66323	0.012949
4465	ENSG00000100294	MCAT	0.267675	2.06866	0.012968
4466	ENSG00000175634	RPS6KB2	0.17474	5.684862	0.01302
4467	ENSG00000228071	RPL7P47	-0.25058	7.194192	0.013057
4468	ENSG00000227525	RPL7P6	-0.27408	3.092193	0.013059
4469	ENSG00000234978		-0.62323	-0.59396	0.013063
4470	ENSG00000229598	PRDX3P1	0.440519	1.818695	0.013067
4471	ENSG00000215769		0.215782	2.973421	0.013087
4472	ENSG00000123144	C19orf43	0.224792	6.869168	0.013098
4473	ENSG00000242574	HLA-DMB	-0.85775	1.594038	0.013106
4474	ENSG00000271347		-0.38085	0.874061	0.013144
4475	ENSG00000138658	C4orf21	0.308058	3.745917	0.013156
4476	ENSG00000069493	CLEC2D	0.372462	7.392316	0.013158
4477	ENSG00000145293	ENOPH1	0.130121	5.206383	0.013158
4478	ENSG00000065150	IPO5	0.126233	6.920816	0.01317
4479	ENSG00000138463	DIRC2	-0.34703	3.769713	0.01317
4480	ENSG00000109065	NAT9	-0.15697	4.511918	0.013223
4481	ENSG00000136143	SUCLA2	0.27002	4.329001	0.013243
4482	ENSG00000154001	PPP2R5E	0.143566	6.104412	0.013243
4483	ENSG00000167550	RHEBL1	0.380565	2.284341	0.013261
4484	ENSG00000182154	MRPL41	0.349625	3.75232	0.013263
4485	ENSG00000189091	SF3B3	0.265056	6.659095	0.013263

4486	ENSG00000133131	MORC4	-0.29178	3.078206	0.013264
4487	ENSG00000246366		-0.49751	0.255581	0.013264
4488	ENSG00000269837		-0.29992	2.064158	0.013283
4489	ENSG00000175746	C15orf54	-0.88398	-0.99213	0.013283
4490	ENSG00000184226	PCDH9	-0.66387	1.895918	0.013293
4491	ENSG00000164077	MON1A	0.376639	2.265165	0.013297
4492	ENSG00000160813	PPP1R35	0.291939	2.772555	0.013297
4493	ENSG00000171467	ZNF318	-0.32271	6.039766	0.013311
4494	ENSG00000158887	MPZ	0.307282	0.938255	0.013311
4495	ENSG00000129518	EAPP	0.210668	6.040074	0.013311
4496	ENSG00000117335	CD46	-0.19754	8.185982	0.013311
4497	ENSG00000178338	ZNF354B	-0.29	3.687846	0.013318
4498	ENSG00000244627		-0.34554	0.426457	0.013319
4499	ENSG00000270012		-0.32419	1.734309	0.013403
4500	ENSG00000104969	SGTA	0.265941	5.223853	0.013412
4501	ENSG00000246082	NUDT16P1	-1.05249	0.500226	0.013455
4502	ENSG00000235916		0.457829	-0.2457	0.013495
4503	ENSG00000249119	MTND6P4	-0.38408	6.88222	0.0135
4504	ENSG00000138303	ASCC1	0.218093	4.24166	0.013501
4505	ENSG00000112081	SRSF3	0.22411	8.753421	0.013505
4506	ENSG00000080503	SMARCA2	-0.15313	6.858267	0.013522
4507	ENSG00000163736	PPBP	-0.7149	6.312991	0.013563
4508	ENSG00000138629	UBL7	0.299832	4.932988	0.013573
4509	ENSG00000137714	FDX1	0.216364	4.658712	0.013573
4510	ENSG00000006747	SCIN	0.849333	-1.39198	0.01358
4511	ENSG00000179715	PCED1B	0.288375	5.48752	0.013593
4512	ENSG00000165548	TMEM63C	0.478039	2.042176	0.013616
4513	ENSG00000156265	MAP3K7CL	-0.6019	6.249602	0.013617
4514	ENSG00000121210	KIAA0922	-0.22851	6.612023	0.013642
4515	ENSG00000103671	TRIP4	0.216932	4.971204	0.013643
4516	ENSG00000172638	EFEMP2	-0.32433	1.696472	0.013662
4517	ENSG00000225200		-0.27876	4.920812	0.013668
4518	ENSG00000116171	SCP2	0.213995	6.960788	0.013676
4519	ENSG00000087085	ACHE	1.213399	-2.27594	0.013684
4520	ENSG00000183401	CCDC159	-0.23624	4.161267	0.013689
4521	ENSG00000260401		-0.7636	0.734657	0.01372
4522	ENSG00000156973	PDE6D	0.350761	4.237497	0.013721
4523	ENSG00000144115	THNSL2	-0.92037	0.190772	0.013746
4524	ENSG00000255909		0.36716	0.688776	0.013747
4525	ENSG00000158481	CD1C	-0.6445	3.885015	0.013751
4526	ENSG00000102910	LONP2	-0.1197	6.191628	0.013756
4527	ENSG00000188659	FAM154B	-1.44814	-0.35932	0.013756
4528	ENSG00000111786	SRSF9	0.090777	7.105111	0.013764
4529	ENSG00000140090	SLC24A4	-0.60953	3.938455	0.013766

4530	ENSG00000153253	SCN3A	-0.60384	1.212944	0.013824
4531	ENSG00000127824	TUBA4A	0.450971	7.335856	0.013842
4532	ENSG00000166797	FAM96A	0.275161	5.55849	0.013879
4533	ENSG00000123130	ACOT9	0.270658	5.893877	0.013902
4534	ENSG00000184840	TMED9	0.19063	6.59576	0.013917
4535	ENSG00000251323		-0.44492	0.70297	0.013917
4536	ENSG00000213080		0.296444	1.151983	0.013921
4537	ENSG00000110799	VWF	-0.63897	0.530525	0.013934
4538	ENSG00000160683	CXCR5	-0.5956	0.987408	0.013936
4539	ENSG00000196652	ZKSCAN5	0.230455	4.099853	0.013942
4540	ENSG00000270157		-0.45959	2.698711	0.013974
4541	ENSG00000103245	NARFL	0.2135	3.646474	0.013984
4542	ENSG00000174780	SRP72	0.144018	7.521507	0.013984
4543	ENSG00000112195	TREML2	-0.51826	2.824179	0.013996
4544	ENSG00000249014	HMGN2P4	0.667185	-1.24264	0.013999
4545	ENSG00000100227	POLDIP3	0.20622	6.633243	0.014031
4546	ENSG00000185340	GAS2L1	-0.59887	2.718369	0.014034
4547	ENSG00000162419	GMEB1	0.231779	4.688355	0.014036
4548	ENSG00000121879	PIK3CA	-0.30232	6.674344	0.014067
4549	ENSG00000255587	RAB44	-0.58677	1.77873	0.014085
4550	ENSG00000100427	MLC1	0.418674	3.900101	0.01412
4551	ENSG00000170345	FOS	-1.09596	8.995659	0.014143
4552	ENSG00000253506	NACA2	-0.18536	4.057919	0.01417
4553	ENSG00000143947	RPS27A	-0.28077	10.5458	0.014174
4554	ENSG00000254760		0.559409	-0.4264	0.014182
4555	ENSG00000166428	PLD4	-0.59324	3.173187	0.014183
4556	ENSG00000258130		-0.30109	0.937515	0.014199
4557	ENSG00000174374	WBSCR16	0.245579	3.477869	0.014206
4558	ENSG00000160325	CACFD1	-0.33364	2.224881	0.014209
4559	ENSG00000124508	BTN2A2	0.296062	4.888895	0.014226
4560	ENSG00000102974	CTCF	0.211107	6.055995	0.014226
4561	ENSG00000213995	CARKD	0.185039	4.975064	0.014226
4562	ENSG00000178913	TAF7	-0.1609	7.482981	0.014226
4563	ENSG00000152767	FARP1	-0.4969	0.694916	0.014226
4564	ENSG00000169704	GP9	-0.65031	1.737478	0.014239
4565	ENSG00000172613	RAD9A	0.284904	3.843764	0.014255
4566	ENSG00000187953	PMS2CL	0.152595	3.916162	0.014266
4567	ENSG00000259407		-0.42917	-0.43972	0.014266
4568	ENSG00000185291	IL3RA	-0.50975	3.348828	0.014395
4569	ENSG00000105063	PPP6R1	0.25065	6.957831	0.014418
4570	ENSG00000112992	NNT	0.230343	6.189671	0.014428
4571	ENSG00000145354	CISD2	0.292251	4.971297	0.01444
4572	ENSG00000064666	CNN2	0.347286	7.323539	0.014461
4573	ENSG00000163803	PLB1	-0.60186	2.858239	0.014461

4574	ENSG00000186081	KRT5	-0.83248	0.749056	0.014469
4575	ENSG00000158470	B4GALT5	0.412435	6.818677	0.014473
4576	ENSG00000175215	CTDSP2	-0.2927	7.619295	0.014479
4577	ENSG00000243071		-0.26778	3.84629	0.01448
4578	ENSG00000146066	HIGD2A	0.250529	5.922572	0.014499
4579	ENSG00000225698	IGHV3-72	0.935234	-0.24216	0.014527
4580	ENSG00000123338	NCKAP1L	0.237223	7.906076	0.014529
4581	ENSG00000205089	CCNI2	-0.39084	-0.34588	0.014539
4582	ENSG00000176209	SMIM19	0.223654	4.357189	0.014546
4583	ENSG00000124802	EEF1E1	0.223126	3.280788	0.014581
4584	ENSG00000125347	IRF1	0.313019	8.791552	0.014599
4585	ENSG00000109099	PMP22	-0.7044	-0.09433	0.01463
4586	ENSG00000013392	RWDD2A	-0.28832	2.267155	0.014647
4587	ENSG00000196139	AKR1C3	-0.5254	2.587062	0.014647
4588	ENSG00000136986	DERL1	0.227761	6.934635	0.014672
4589	ENSG00000151687	ANKAR	-0.25444	2.066033	0.014672
4590	ENSG00000166839	ANKDD1A	0.304782	3.375901	0.014672
4591	ENSG00000185838	GNB1L	0.341524	1.527165	0.014712
4592	ENSG00000048052	HDAC9	-0.39908	4.32968	0.014712
4593	ENSG00000224307		-1.19461	-1.5382	0.014714
4594	ENSG00000102302	FGD1	-0.22476	1.7691	0.014718
4595	ENSG00000131242	RAB11FIP4	0.210363	6.546034	0.014719
4596	ENSG00000266501		0.273725	1.159291	0.014724
4597	ENSG00000214561	RBBP4P4	0.318842	0.473987	0.014726
4598	ENSG00000099953	MMP11	-0.65991	-0.51029	0.014736
4599	ENSG00000235944	ZNF815P	-0.3833	3.225385	0.014741
4600	ENSG00000244582	RPL21P120	-0.30198	1.843395	0.014752
4601	ENSG00000096654	ZNF184	0.545311	4.935977	0.014752
4602	ENSG00000162191	UBXN1	0.162226	7.011754	0.014769
4603	ENSG00000186283	TOR3A	0.221511	5.403563	0.014798
4604	ENSG00000127586	CHTF18	0.257137	3.151368	0.014815
4605	ENSG00000011007	TCEB3	0.181886	5.634331	0.014827
4606	ENSG00000139344	AMDHD1	-0.45439	0.300674	0.01484
4607	ENSG00000174776	WDR49	-0.89764	0.231419	0.014853
4608	ENSG00000080839	RBL1	0.233898	4.706975	0.014869
4609	ENSG00000186184	POLR1D	0.167033	6.691251	0.014874
4610	ENSG00000159377	PSMB4	0.145264	7.667019	0.014915
4611	ENSG00000114554	PLXNA1	-0.37372	3.311807	0.014925
4612	ENSG00000230568	SF3A3P1	0.266448	0.422127	0.014983
4613	ENSG00000067221	STOML1	0.398191	1.976486	0.014995
4614	ENSG00000145912	NHP2	0.24097	3.709271	0.015007
4615	ENSG00000136045	PWP1	0.185591	5.908707	0.015052
4616	ENSG00000108786	HSD17B1	-0.41536	0.773526	0.015052
4617	ENSG00000087088	BAX	0.255307	5.910977	0.015065

4618	ENSG00000100811	YY1	-0.13098	7.581451	0.015072
4619	ENSG00000120256	LRP11	-0.50095	0.211983	0.015076
4620	ENSG00000232864		0.341524	0.160511	0.015116
4621	ENSG00000141543	EIF4A3	0.309852	5.900673	0.015126
4622	ENSG00000186104	CYP2R1	-0.25882	4.599241	0.015136
4623	ENSG00000102921	N4BP1	0.357405	6.89356	0.015188
4624	ENSG00000130997	POLN	0.343921	0.194046	0.015188
4625	ENSG00000272462		-0.33485	2.520631	0.015188
4626	ENSG00000182379	NXPH4	1.135283	-1.20402	0.015204
4627	ENSG00000232818	RPS2P32	0.439322	-0.44531	0.015226
4628	ENSG00000186409	CCDC30	-0.45153	1.363733	0.015252
4629	ENSG00000248785	HIGD1AP14	0.450374	-0.72764	0.015257
4630	ENSG00000095066	HOOK2	-0.24722	2.528412	0.015287
4631	ENSG00000151611	MMAA	0.249916	3.350552	0.015295
4632	ENSG00000139746	RBM26	-0.18285	7.06801	0.015296
4633	ENSG00000241007	SEPT7P6	0.233747	2.907705	0.015339
4634	ENSG00000168876	ANKRD49	-0.22444	6.15968	0.015339
4635	ENSG00000214309	MBLAC1	0.474221	-0.33904	0.015355
4636	ENSG00000234176	HSPA8P1	0.864001	2.286471	0.015373
4637	ENSG00000266786		0.520902	2.909499	0.015398
4638	ENSG00000102390	PBDC1	0.24317	4.885995	0.015419
4639	ENSG00000179918	SEPHS2	0.22625	5.441665	0.015429
4640	ENSG00000204099	NEU4	0.97617	0.757335	0.015448
4641	ENSG00000069275	NUCKS1	0.210861	7.500788	0.015448
4642	ENSG00000121774	KHDRBS1	0.203847	6.991917	0.015495
4643	ENSG00000126947	ARMCX1	-0.42788	0.598935	0.015499
4644	ENSG00000237181		0.326476	1.889839	0.015507
4645	ENSG00000112759	SLC29A1	0.397025	3.041589	0.015542
4646	ENSG00000257176		-0.41893	1.731485	0.015549
4647	ENSG00000177738		-0.22828	2.359421	0.015611
4648	ENSG00000136247	ZDHHC4	0.140562	4.329785	0.015654
4649	ENSG00000171408	PDE7B	-0.51987	0.158897	0.015659
4650	ENSG00000008838	MED24	0.240762	5.254711	0.015663
4651	ENSG00000253785		0.265542	2.042238	0.015673
4652	ENSG00000092068	SLC7A8	-0.79815	-1.39115	0.015673
4653	ENSG00000135439	AGAP2	0.282577	4.796898	0.015687
4654	ENSG00000165591	FAAH2	-0.48241	2.092414	0.015691
4655	ENSG00000001461	NIPAL3	0.227885	5.490514	0.015709
4656	ENSG00000215717	TMEM167B	-0.1823	7.099162	0.01571
4657	ENSG00000214110	LDHAP4	0.509063	3.139008	0.015719
4658	ENSG00000230847		-0.7762	0.292455	0.015745
4659	ENSG00000173678	SPDYE2B	-0.37624	1.992243	0.01577
4660	ENSG00000121989	ACVR2A	-0.44973	3.862402	0.015777
4661	ENSG00000168961	LGALS9	0.511746	6.459334	0.015791

4662	ENSG00000211685	IGLC7	1.123852	2.563166	0.015819
4663	ENSG00000149657	LSM14B	0.224283	5.065254	0.015819
4664	ENSG00000125651	GTF2F1	0.180376	5.955894	0.015819
4665	ENSG00000242571	RPL21P11	-0.26603	3.731918	0.015819
4666	ENSG00000164162	ANAPC10	0.264011	3.488238	0.015853
4667	ENSG00000255730		1.039878	-1.77571	0.015888
4668	ENSG00000227242	NBPF13P	-0.39968	1.066658	0.015893
4669	ENSG00000145103	ILDR1	0.458288	-0.82094	0.015906
4670	ENSG00000175414	ARL10	-0.39922	3.298952	0.015913
4671	ENSG00000211765	TRBJ2-2	0.403433	0.063192	0.015957
4672	ENSG00000174306	ZHX3	-0.26004	3.719706	0.015975
4673	ENSG00000166847	DCTN5	0.195505	6.097333	0.015993
4674	ENSG00000011376	LARS2	0.23968	3.705467	0.016036
4675	ENSG00000238268		-0.63233	0.573952	0.016036
4676	ENSG00000078269	SYNJ2	-0.29768	4.316143	0.016066
4677	ENSG00000257335	MGAM	-0.71454	3.329823	0.016092
4678	ENSG00000178464		-0.22184	9.873794	0.016186
4679	ENSG00000105726	ATP13A1	0.218095	5.753356	0.016234
4680	ENSG00000120458	MSANTD2	-0.30659	3.390184	0.016273
4681	ENSG00000138829	FBN2	-0.67215	2.864467	0.016279
4682	ENSG00000170293	CMTM8	-0.51617	0.592567	0.016286
4683	ENSG00000034693	PEX3	0.256127	3.561616	0.016293
4684	ENSG00000155265	GOLGA7B	-0.33874	3.448448	0.016293
4685	ENSG00000049883	PTCD2	0.311012	1.906495	0.016381
4686	ENSG00000180530	NRIP1	-0.3728	6.438628	0.016402
4687	ENSG00000115825	PRKD3	-0.20804	6.614971	0.016412
4688	ENSG00000170606	HSPA4	0.213216	6.461888	0.016482
4689	ENSG00000118855	MFSD1	-0.29633	6.638674	0.016484
4690	ENSG00000134470	IL15RA	0.313622	3.265709	0.016509
4691	ENSG00000068308	OTUD5	0.159485	6.266525	0.016563
4692	ENSG00000162892	IL24	-0.29977	4.548074	0.016563
4693	ENSG00000011275	RNF216	-0.2109	6.390689	0.016577
4694	ENSG00000188352	FOCAD	0.215017	4.196859	0.016607
4695	ENSG00000167202	TBC1D2B	0.323134	6.642659	0.01661
4696	ENSG00000267453		-0.93808	-1.54856	0.016692
4697	ENSG00000150667	FSIP1	0.422229	-0.47699	0.016695
4698	ENSG00000177842	ZNF620	0.306122	1.214275	0.016729
4699	ENSG00000224174		-0.44764	0.027947	0.016779
4700	ENSG00000073331	ALPK1	-0.36788	4.324878	0.016797
4701	ENSG00000255760		-0.72251	0.204013	0.016848
4702	ENSG00000224892	RPS4XP16	-0.30086	4.79305	0.01687
4703	ENSG00000254837		-0.59249	0.364574	0.01687
4704	ENSG00000261997		-0.62267	0.863241	0.01687
4705	ENSG00000122970	IFT81	-0.43926	0.285585	0.016876

4706	ENSG00000233223		0.302348	1.265923	0.01692
4707	ENSG00000196169	KIF19	0.706911	2.574901	0.016923
4708	ENSG00000008394	MGST1	-0.60766	3.119557	0.016932
4709	ENSG00000168884	TNIP2	0.19766	5.729708	0.016937
4710	ENSG00000158864	NDUFS2	0.199439	5.944708	0.016938
4711	ENSG00000176945	MUC20	-0.55784	1.052669	0.016953
4712	ENSG00000135407	AVIL	-0.39187	1.27397	0.016974
4713	ENSG00000144867	SRPRB	0.281428	4.887357	0.016985
4714	ENSG00000228037		-0.36454	0.066767	0.017025
4715	ENSG00000140854	KATNB1	0.219614	4.060719	0.01709
4716	ENSG00000151876	FBXO4	0.202786	3.340966	0.017108
4717	ENSG00000160446	ZDHHC12	0.290868	4.223168	0.017112
4718	ENSG00000157734	SNX22	-0.39222	3.041081	0.017117
4719	ENSG00000151366	NDUFC2	0.214665	4.24859	0.017126
4720	ENSG00000117533	VAMP4	-0.20474	5.654564	0.017133
4721	ENSG00000148516	ZEB1	-0.43282	5.868865	0.017159
4722	ENSG00000141140	MYO19	0.170506	4.730294	0.017194
4723	ENSG00000113448	PDE4D	0.544594	8.530303	0.017223
4724	ENSG00000026103	FAS	0.264115	6.07896	0.017223
4725	ENSG00000107147	KCNT1	-0.56925	-1.06307	0.017223
4726	ENSG00000230903	RPL9P8	-0.29758	7.30445	0.017229
4727	ENSG00000149531	FRG1B	0.252619	4.265278	0.01723
4728	ENSG00000132361	CLUH	0.239161	4.613939	0.017233
4729	ENSG00000142405	NLRP12	-0.64825	3.357663	0.017233
4730	ENSG00000182732	RGS6	-0.6035	0.496911	0.017245
4731	ENSG00000158109	TPRG1L	-0.20283	5.789005	0.017266
4732	ENSG00000164330	EBF1	-0.4961	2.586089	0.017284
4733	ENSG00000125245	GPR18	0.486308	4.999473	0.01729
4734	ENSG00000273295		-1.81899	-1.11432	0.017303
4735	ENSG00000132507	EIF5A	0.214746	7.653538	0.017312
4736	ENSG00000197696	NMB	0.340619	0.902671	0.017352
4737	ENSG00000180233	ZNRF2	0.198851	5.285927	0.017353
4738	ENSG00000186047	DLEU7	-0.49148	0.239302	0.01736
4739	ENSG00000273226		-0.35805	0.602482	0.017365
4740	ENSG00000005020	SKAP2	-0.39539	5.938642	0.017365
4741	ENSG00000157020	SEC13	0.155092	5.880817	0.01737
4742	ENSG00000198816	ZNF358	-0.45355	1.206374	0.017382
4743	ENSG00000230606		-0.34057	5.170025	0.017406
4744	ENSG00000225975		-0.31296	0.227445	0.01745
4745	ENSG00000121644	DESI2	0.21599	5.83014	0.017492
4746	ENSG00000138688	KIAA1109	-0.26201	8.157852	0.017492
4747	ENSG00000165914	TTC7B	-0.44648	1.941176	0.017495
4748	ENSG00000240376		-0.23736	7.701318	0.017551
4749	ENSG00000179010	MRFAP1	0.220929	7.46025	0.017562

4750	ENSG00000100823	APEX1	-0.12757	6.412459	0.017589
4751	ENSG00000255320		-0.36713	0.949224	0.017589
4752	ENSG00000006194	ZNF263	-0.14434	5.48494	0.017649
4753	ENSG00000011132	APBA3	0.25498	4.40936	0.017674
4754	ENSG00000224086		-0.52614	-0.11725	0.017691
4755	ENSG00000235609		-0.44089	0.911636	0.017714
4756	ENSG00000132170	PPARG	1.079342	-0.42571	0.017718
4757	ENSG00000182500	ORAI1	0.271356	5.732291	0.017728
4758	ENSG00000140839	CLEC18B	-0.42166	-0.53728	0.017761
4759	ENSG00000007372	PAX6	-0.40605	1.529996	0.017769
4760	ENSG00000229605		-0.25928	3.907982	0.017775
4761	ENSG00000106603	COA1	0.147947	5.620481	0.017795
4762	ENSG00000118162	KPTN	0.347192	1.978647	0.017841
4763	ENSG00000171916	LGALS9C	0.421846	2.901434	0.017844
4764	ENSG00000136518	ACTL6A	0.238869	4.757737	0.017893
4765	ENSG00000125843	AP5S1	0.253987	2.691267	0.017923
4766	ENSG00000147164	SNX12	0.174786	4.812932	0.017925
4767	ENSG00000225032		-0.36557	1.966954	0.018025
4768	ENSG00000259431	THTPA	-0.33342	1.422514	0.018036
4769	ENSG00000167644	C19orf33	-0.81937	-1.45029	0.018036
4770	ENSG00000162825	NBPF8	-0.43466	1.586118	0.018036
4771	ENSG00000135272	MDFIC	-0.23197	6.681454	0.018038
4772	ENSG00000148429	USP6NL	-0.34141	4.54946	0.018038
4773	ENSG00000258515		-0.40175	1.328247	0.018038
4774	ENSG00000186716	BCR	0.313785	6.140468	0.018064
4775	ENSG00000168566	SNRNP48	-0.18519	4.758018	0.018067
4776	ENSG00000101361	NOP56	0.183762	6.556037	0.018071
4777	ENSG00000182208	MOB2	0.200178	5.445038	0.018133
4778	ENSG00000122986	HVCN1	-0.39452	4.282827	0.018136
4779	ENSG00000086061	DNAJA1	0.321816	7.827063	0.018174
4780	ENSG00000110711	AIP	0.183842	6.01557	0.018204
4781	ENSG00000240919		-0.30067	4.790443	0.018208
4782	ENSG00000150768	DLAT	0.181761	4.60892	0.018249
4783	ENSG00000117115	PADI2	-0.7765	3.178265	0.018252
4784	ENSG00000160229	ZNF66	-0.3664	0.993503	0.018258
4785	ENSG00000154328	NEIL2	-0.23321	2.783425	0.01826
4786	ENSG00000267174		-1.07333	-1.92512	0.018268
4787	ENSG00000078304	PPP2R5C	0.239784	8.976152	0.018294
4788	ENSG00000256053	APOPT1	0.19237	3.315741	0.018299
4789	ENSG00000018510	AGPS	0.16043	6.22451	0.018299
4790	ENSG00000144357	UBR3	-0.19802	6.00535	0.018299
4791	ENSG00000211812	TRAV26-2	0.542491	0.054279	0.018309
4792	ENSG00000146005	PSD2	0.643067	-1.12739	0.01835
4793	ENSG00000119457	SLC46A2	-0.87828	1.331994	0.018368

4794	ENSG00000178691	SUZ12	0.184072	6.453024	0.018382
4795	ENSG00000237200	ZBTB40-IT1	-0.59402	-0.8739	0.018382
4796	ENSG00000105221	AKT2	0.155707	7.165648	0.01844
4797	ENSG00000182700	IGIP	-0.50056	2.2656	0.018447
4798	ENSG00000148606	POLR3A	0.20372	4.666663	0.018541
4799	ENSG00000213216		-0.27919	4.710603	0.018553
4800	ENSG00000162804	SNED1	-0.41947	3.903924	0.018576
4801	ENSG00000132002	DNAJB1	0.422373	8.44523	0.018615
4802	ENSG00000105968	H2AFV	0.236151	7.320195	0.018671
4803	ENSG00000115839	RAB3GAP1	-0.31297	5.777992	0.018671
4804	ENSG00000169372	CRADD	0.180252	3.090282	0.018688
4805	ENSG00000262848		0.391916	-0.00434	0.018711
4806	ENSG00000084234	APLP2	-0.37806	8.552585	0.018737
4807	ENSG00000149485	FADS1	-0.37173	3.342737	0.018758
4808	ENSG00000111729	CLEC4A	-0.47152	5.03083	0.018758
4809	ENSG00000102878	HSF4	-0.29176	1.664465	0.018787
4810	ENSG00000157017	GHRL	-0.41948	-0.03971	0.018823
4811	ENSG00000187187	ZNF546	-0.36238	1.909289	0.018861
4812	ENSG00000131931	THAP1	0.180687	4.220673	0.018873
4813	ENSG00000055950	MRPL43	0.173395	4.694431	0.018875
4814	ENSG00000105085	MED26	0.371501	3.768978	0.018893
4815	ENSG00000121064	SCPEP1	-0.4703	6.481115	0.018953
4816	ENSG00000145990	GFOD1	0.273432	5.086562	0.018969
4817	ENSG00000119042	SATB2	-0.58379	-0.02487	0.018973
4818	ENSG00000136938	ANP32B	0.193719	7.165398	0.018991
4819	ENSG00000273038		-0.41425	2.356801	0.018991
4820	ENSG00000179840	C1orf200	0.463473	-0.79172	0.019011
4821	ENSG00000102753	KPNA3	0.199083	6.147033	0.019011
4822	ENSG00000215021	PHB2	0.167826	7.132709	0.01905
4823	ENSG00000174021	GNG5	0.24348	6.167209	0.019075
4824	ENSG00000212127	TAS2R14	-0.38106	1.872742	0.019085
4825	ENSG00000254064		-0.48695	-0.74976	0.019089
4826	ENSG00000104691	UBXN8	0.264223	2.431842	0.019096
4827	ENSG00000204130	RUFY2	-0.18269	4.03726	0.019143
4828	ENSG00000189195	BTBD8	-0.6174	-0.97404	0.019143
4829	ENSG00000134001	EIF2S1	0.26873	5.809977	0.019145
4830	ENSG00000108829	LRRC59	0.223538	6.008798	0.019145
4831	ENSG00000139726	DENR	0.189245	5.874171	0.019145
4832	ENSG00000267589		-0.37322	0.630053	0.019145
4833	ENSG00000196968	FUT11	0.242413	4.908806	0.019147
4834	ENSG00000230124		-0.40007	2.261373	0.019147
4835	ENSG00000234004		-0.27664	4.732248	0.019161
4836	ENSG00000254701		-0.3485	-0.3082	0.019195
4837	ENSG00000114745	GORASP1	-0.30312	4.783119	0.019212

4838	ENSG00000234571		0.89131	-1.01301	0.019223
4839	ENSG00000172974		0.170612	3.810482	0.019225
4840	ENSG00000247627	MTND4P12	-0.32194	9.028368	0.019228
4841	ENSG00000076067	RBMS2	-0.33568	2.436918	0.019247
4842	ENSG00000155621	C9orf85	0.223764	2.793837	0.019277
4843	ENSG00000153037	SRP19	0.19335	4.820512	0.019283
4844	ENSG00000179698	KIAA1875	-0.36785	0.424716	0.019321
4845	ENSG00000267023	LRRC37A16P	-0.21563	4.642652	0.019325
4846	ENSG00000152147	GEMIN6	0.326298	2.388172	0.019339
4847	ENSG00000058804	NDC1	0.174652	4.679835	0.019339
4848	ENSG00000177917	ARL6IP6	0.187033	4.828118	0.019339
4849	ENSG00000102309	PIN4	0.327482	3.21626	0.019354
4850	ENSG00000267312		0.309103	1.427882	0.019354
4851	ENSG00000109861	CTSC	0.264067	7.79548	0.019359
4852	ENSG00000090920	FCGBP	-0.49889	4.153922	0.019374
4853	ENSG00000107937	GTPBP4	0.302813	6.0452	0.019412
4854	ENSG00000224956		-0.4188	2.821461	0.019412
4855	ENSG00000042286	AIFM2	0.235241	2.244752	0.019454
4856	ENSG00000198146	ZNF770	-0.29271	6.203774	0.019465
4857	ENSG00000198915	RASGEF1A	0.404137	3.032418	0.019495
4858	ENSG00000163344	PMVK	0.332161	3.801155	0.019516
4859	ENSG00000272144		-0.58121	-0.54886	0.019564
4860	ENSG00000186063	AIDA	0.206402	6.577982	0.019576
4861	ENSG00000126861	OMG	0.370704	1.357736	0.019588
4862	ENSG00000233396		-0.46984	-0.16328	0.019596
4863	ENSG00000178537	SLC25A20	0.481066	4.629221	0.019598
4864	ENSG00000107874	CUEDC2	0.24101	4.484714	0.019627
4865	ENSG00000164867	NOS3	-0.56509	-0.21108	0.019634
4866	ENSG00000168026	TTC21A	-0.34996	2.128088	0.019634
4867	ENSG00000100767	PAPLN	-0.38119	1.941536	0.019634
4868	ENSG00000100362	PVALB	-1.03775	-2.18629	0.019634
4869	ENSG00000197713	RPE	0.300222	4.307416	0.019636
4870	ENSG00000075142	SRI	0.187447	5.840789	0.019643
4871	ENSG00000108055	SMC3	0.220377	6.674993	0.019651
4872	ENSG00000104823	ECH1	0.315232	5.168757	0.019662
4873	ENSG00000164344	KLKB1	-0.62646	0.527305	0.019662
4874	ENSG00000227486		-0.4476	-0.5417	0.019696
4875	ENSG00000237037	NDUFA6-AS1	-0.28245	3.245922	0.019702
4876	ENSG00000175518	UBQLNL	-0.65209	-0.66706	0.019702
4877	ENSG00000159692	CTBP1	0.199715	6.742516	0.019731
4878	ENSG00000196159	FAT4	-0.82978	0.22978	0.019762
4879	ENSG00000072952	MRVI1	-0.71839	2.02429	0.01977
4880	ENSG00000259634		-0.27853	1.763006	0.019827
4881	ENSG00000272821		0.453337	2.717482	0.019845

4882	ENSG00000197016	ZNF470	-0.41828	1.146577	0.019859
4883	ENSG00000257956		0.787883	-0.33279	0.019864
4884	ENSG00000258476		-0.72288	-0.29794	0.019871
4885	ENSG00000172469	MANEA	0.312038	4.165279	0.019881
4886	ENSG00000111229	ARPC3	0.199417	8.528089	0.019884
4887	ENSG00000262370		-0.56201	2.786365	0.019944
4888	ENSG00000185158	LRRC37B	-0.15507	4.532016	0.019947
4889	ENSG00000132004	FBXW9	0.295177	0.579738	0.019954
4890	ENSG00000157483	MYO1E	-0.39937	2.477089	0.019976
4891	ENSG00000226524		-0.54238	-1.14232	0.019991
4892	ENSG00000149547	EI24	0.153854	5.351268	0.020026
4893	ENSG00000205744	DENND1C	0.210807	6.617016	0.020035
4894	ENSG00000235576		0.459527	3.243711	0.020045
4895	ENSG00000115267	IFIH1	0.425148	5.59668	0.020045
4896	ENSG00000234338		-0.65576	-0.33283	0.020051
4897	ENSG00000197415	VEPH1	-0.69211	-0.33736	0.020068
4898	ENSG00000131943	C19orf12	0.18696	4.811176	0.02007
4899	ENSG00000230793	SMARCE1P5	0.283112	0.079485	0.020117
4900	ENSG00000249485	RBBP4P1	0.25138	1.194307	0.02012
4901	ENSG00000196422	PPP1R26	-0.5677	1.34018	0.020133
4902	ENSG00000143776	CDC42BPA	-0.40368	0.715887	0.020166
4903	ENSG00000103260	METRN	0.340545	1.654315	0.02017
4904	ENSG00000242802	AP5Z1	0.235724	5.471379	0.020179
4905	ENSG00000187699	C2orf88	-0.49261	4.133421	0.020183
4906	ENSG00000168067	MAP4K2	-0.18781	5.74406	0.020187
4907	ENSG00000188818	ZDHHC11	-0.49541	0.816935	0.020193
4908	ENSG00000211584	SLC48A1	-0.35756	2.417514	0.020255
4909	ENSG00000025772	TOMM34	0.247902	3.750664	0.020275
4910	ENSG00000099365	STX1B	-0.46791	-0.39637	0.020306
4911	ENSG00000124155	PIGT	0.233959	6.001247	0.020327
4912	ENSG00000171385	KCND3	-0.66431	-0.90448	0.020327
4913	ENSG00000169905	TOR1AIP2	0.147237	5.905631	0.020347
4914	ENSG00000261428		-0.49003	0.866833	0.020358
4915	ENSG00000127337	YEATS4	0.260828	4.463477	0.020382
4916	ENSG00000146054	TRIM7	-0.64896	0.385867	0.020389
4917	ENSG00000246985	SOCS2-AS1	-0.3713	0.229282	0.02042
4918	ENSG00000168061	SAC3D1	0.532534	0.695889	0.020452
4919	ENSG00000137831	UACA	-0.54482	1.445609	0.020452
4920	ENSG00000182541	LIMK2	-0.26129	6.624412	0.020461
4921	ENSG00000117425	PTCH2	-0.38371	0.20894	0.020465
4922	ENSG00000083535	PIBF1	-0.2388	4.235859	0.020474
4923	ENSG00000196199	MPHOSPH8	-0.21939	6.616928	0.020488
4924	ENSG00000176593		-0.39551	2.333764	0.020499
4925	ENSG00000169230	PRELID1	0.293798	5.136093	0.0205

4926	ENSG00000068400	GRIPAP1	0.182368	6.081102	0.020505
4927	ENSG00000094841	UPRT	0.167581	4.392373	0.020515
4928	ENSG00000136010	ALDH1L2	0.578859	-0.83855	0.02057
4929	ENSG00000219133		-0.33212	1.107293	0.020574
4930	ENSG00000151006	PRSS53	-0.33762	1.377357	0.020574
4931	ENSG00000181819	KCTD9P2	0.363396	1.47257	0.020584
4932	ENSG00000249617		-0.25079	4.17941	0.020586
4933	ENSG00000127526	SLC35E1	0.344189	5.7008	0.020592
4934	ENSG00000134375	TIMM17A	0.253504	5.851038	0.020592
4935	ENSG00000172771	EFCAB12	-0.38883	0.244574	0.020592
4936	ENSG00000163138	PACRGL	-0.19693	2.691471	0.020643
4937	ENSG00000197429	IPP	0.207617	3.078685	0.020644
4938	ENSG00000070785	EIF2B3	0.221649	3.607771	0.020653
4939	ENSG00000127080	IPPK	0.315232	2.253628	0.020792
4940	ENSG00000237550	UBE2Q2P6	-0.29055	7.302713	0.020831
4941	ENSG00000180015		0.411847	-0.7206	0.020833
4942	ENSG00000108465	CDK5RAP3	-0.16186	6.988544	0.020833
4943	ENSG00000214022	REPIN1	-0.26262	5.073453	0.020833
4944	ENSG00000075234	TTC38	0.320236	5.751937	0.020834
4945	ENSG00000008869	HEATR5B	-0.21646	6.502287	0.020845
4946	ENSG00000064547	LPAR2	-0.22576	4.81761	0.020845
4947	ENSG00000214548	MEG3	-1.06807	0.732221	0.020845
4948	ENSG00000119411	BSPRY	0.688485	-0.83464	0.020854
4949	ENSG00000269069		-0.29701	1.098618	0.020854
4950	ENSG00000178425	NT5DC1	-0.22733	5.035693	0.020873
4951	ENSG00000147316	MCPH1	0.215028	5.421754	0.020881
4952	ENSG00000153071	DAB2	-0.42757	3.19348	0.020971
4953	ENSG00000259092	TRAV30	0.621641	-0.70601	0.020973
4954	ENSG00000108641	B9D1	0.414816	-0.12411	0.020996
4955	ENSG00000079459	FDFT1	0.173336	6.442739	0.020997
4956	ENSG00000270906		-0.30846	3.506765	0.020999
4957	ENSG00000134490	TMEM241	0.253638	1.748945	0.021043
4958	ENSG00000186481	ANKRD20A5P	-0.46823	0.839297	0.021063
4959	ENSG00000272512		0.828404	-0.54663	0.021073
4960	ENSG00000072210	ALDH3A2	-0.25968	4.856998	0.021089
4961	ENSG00000272288		0.298142	0.84287	0.021143
4962	ENSG00000124019	FAM124B	-0.59516	-0.29931	0.021207
4963	ENSG00000236937	PTGES3P4	0.26976	1.296583	0.02123
4964	ENSG00000112146	FBXO9	-0.16606	6.027112	0.021254
4965	ENSG00000184743	ATL3	-0.2002	5.710275	0.021256
4966	ENSG00000142875	PRKACB	0.256984	7.584463	0.021257
4967	ENSG00000236088	COX10-AS1	-0.31498	2.993478	0.021264
4968	ENSG00000248578	NPM1P21	0.194156	4.870352	0.021271
4969	ENSG00000159339	PADI4	-0.60984	3.23016	0.021287

4970	ENSG00000011304	PTBP1	0.206068	7.405016	0.021288
4971	ENSG00000111669	TPI1	0.26934	7.805578	0.021368
4972	ENSG00000131127	ZNF141	-0.33187	4.025772	0.021376
4973	ENSG00000203865	ATP1A1OS	-0.25077	1.333768	0.021381
4974	ENSG00000231952	DPY19L1P2	-0.34812	0.425791	0.021381
4975	ENSG00000240652		-0.2925	6.342489	0.021403
4976	ENSG00000078124	ACER3	-0.25637	5.239204	0.021464
4977	ENSG00000165511	C10orf25	-0.56176	0.351821	0.021474
4978	ENSG00000124333	VAMP7	0.150472	5.576651	0.021495
4979	ENSG00000185875	THNSL1	0.322872	1.803766	0.021527
4980	ENSG00000228343		-0.27461	2.057081	0.021527
4981	ENSG00000198961	PJA2	-0.17493	8.075881	0.021593
4982	ENSG00000104872	PIH1D1	0.152176	5.588886	0.021602
4983	ENSG00000184281	TSSC4	0.188226	4.958367	0.021606
4984	ENSG00000100504	PYGL	-0.59116	5.006124	0.021655
4985	ENSG00000157741	UBN2	-0.31501	5.259307	0.021709
4986	ENSG00000181039	ANKRD34A	-0.39606	-0.18235	0.021734
4987	ENSG00000272979		-0.51377	-1.0014	0.021734
4988	ENSG00000185261	KIAA0825	0.427499	2.107769	0.021744
4989	ENSG00000150048	CLEC1A	-0.53757	0.210049	0.021766
4990	ENSG00000214391		0.492434	0.196029	0.02179
4991	ENSG00000117640	MTFR1L	-0.18372	5.036323	0.02179
4992	ENSG00000137154	RPS6	-0.23819	11.06658	0.021813
4993	ENSG00000166068	SPRED1	-0.46706	2.589512	0.021831
4994	ENSG00000158467	AHCYL2	-0.26439	4.402718	0.021852
4995	ENSG00000173258	ZNF483	-0.40325	1.930608	0.02186
4996	ENSG00000244490	RWDD4P1	0.253854	0.522245	0.021888
4997	ENSG00000173141	MRP63	0.206448	4.177921	0.02194
4998	ENSG00000155659	VSIG4	-0.78507	1.251996	0.02194
4999	ENSG00000105352	CEACAM4	-1.06527	0.822813	0.021972
5000	ENSG00000140941	MAP1LC3B	0.199661	7.399808	0.021974
5001	ENSG00000107201	DDX58	0.517956	5.872674	0.022004
5002	ENSG00000197841	ZNF181	-0.359	3.013857	0.022004
5003	ENSG00000103051	COG4	0.325472	5.035846	0.022005
5004	ENSG00000214189	ZNF788	0.457987	-0.64597	0.022029
5005	ENSG00000128524	ATP6V1F	0.209443	6.130691	0.022031
5006	ENSG00000169860	P2RY1	-0.57767	0.232784	0.022039
5007	ENSG00000224971	SUMO2P3	0.463661	-0.87379	0.022052
5008	ENSG00000156990	RPUSD3	0.341677	3.837065	0.022056
5009	ENSG00000163430	FSTL1	-0.6901	0.338976	0.022085
5010	ENSG00000244219		0.56405	-1.07448	0.022089
5011	ENSG00000167524		-0.36697	2.267982	0.022105
5012	ENSG00000206562	METTL6	0.265628	2.936147	0.022125
5013	ENSG00000244921		-0.29539	4.515051	0.02213

5014	ENSG00000101181	MTG2	0.213978	3.876703	0.022136
5015	ENSG00000170322	NFRKB	-0.17058	5.40976	0.022139
5016	ENSG00000100029	PES1	0.191962	5.071762	0.022173
5017	ENSG00000206082	LINC01002	-0.41467	2.161499	0.022186
5018	ENSG00000150457	LATS2	-0.3115	4.244432	0.022218
5019	ENSG00000134910	STT3A	0.304828	5.764904	0.02223
5020	ENSG00000240674		-0.25029	3.760486	0.022265
5021	ENSG00000154655	L3MBTL4	-0.3549	1.352424	0.022265
5022	ENSG00000092421	SEMA6A	-0.29565	1.459526	0.022326
5023	ENSG00000117586	TNFSF4	-0.37765	2.660476	0.022338
5024	ENSG00000117143	UAP1	0.52554	5.766528	0.022379
5025	ENSG00000170271	FAXDC2	-0.51949	3.245925	0.022379
5026	ENSG00000135392	DNAJC14	0.210871	3.970635	0.022394
5027	ENSG00000259328		-0.44362	-0.26041	0.022394
5028	ENSG00000109911	ELP4	0.225581	3.558206	0.022419
5029	ENSG00000119138	KLF9	-0.36724	6.504271	0.02242
5030	ENSG00000233025	CRYZP1	-0.53692	-0.92552	0.02242
5031	ENSG00000259291		0.383476	0.858742	0.022423
5032	ENSG00000135842	FAM129A	0.352552	6.965812	0.022444
5033	ENSG00000134283	PPHLN1	0.120354	6.555362	0.022444
5034	ENSG00000269906		0.433673	-0.52326	0.022447
5035	ENSG00000163624	CDS1	-0.69366	-0.20986	0.022464
5036	ENSG00000051620	HEBP2	-0.38105	5.34324	0.022494
5037	ENSG00000188242		-0.24405	2.659079	0.022534
5038	ENSG00000173581	CCDC106	-0.44094	0.581691	0.022534
5039	ENSG00000242067	RPL9P28	-0.32446	4.481893	0.022538
5040	ENSG00000172731	LRRC20	0.566224	1.453301	0.022553
5041	ENSG00000131116	ZNF428	0.273792	3.463126	0.022553
5042	ENSG00000089775	ZBTB25	-0.22615	5.849477	0.022557
5043	ENSG00000272040		-0.4694	-0.2824	0.022582
5044	ENSG00000078967	UBE2D4	-0.18503	3.396834	0.022593
5045	ENSG00000237111	IGHJ3P	0.531068	-0.46892	0.022653
5046	ENSG00000235552	RPL6P27	-0.20378	9.24471	0.022653
5047	ENSG00000089091	DZANK1	-0.28992	1.627623	0.022672
5048	ENSG00000111644	ACRBP	-0.48034	4.205775	0.022702
5049	ENSG00000273265		-0.45794	0.465492	0.022725
5050	ENSG00000235217	TSPY26P	-0.36386	1.460297	0.022731
5051	ENSG00000172301	COPRS	0.271913	2.349982	0.022766
5052	ENSG00000213613		-0.23411	5.61162	0.022767
5053	ENSG00000235381		-0.45054	0.532382	0.022785
5054	ENSG00000111291	GPRC5D	0.803589	-0.06314	0.022808
5055	ENSG00000175354	PTPN2	0.177317	6.72977	0.022844
5056	ENSG00000223972	DDX11L1	-0.74158	-0.87456	0.022845
5057	ENSG00000137767	SQRDL	0.318069	5.548281	0.02287

5058	ENSG00000101445	PPP1R16B	0.510352	8.202917	0.022872
5059	ENSG00000107223	EDF1	0.171303	7.014911	0.022872
5060	ENSG00000254996	ANKHD1-EIF4EBP3	-0.32808	2.142801	0.022912
5061	ENSG00000166762	CATSPER2	-0.50349	1.498505	0.022912
5062	ENSG00000130770	ATPIF1	0.242872	5.785451	0.022967
5063	ENSG00000232470		-0.29665	0.585988	0.022967
5064	ENSG00000245571		0.360808	1.771681	0.022969
5065	ENSG00000173011	TADA2B	-0.19421	5.868119	0.022969
5066	ENSG00000173681	CXorf23	-0.20608	4.100521	0.023011
5067	ENSG00000006453	BAIAP2L1	-0.37167	0.438561	0.023073
5068	ENSG00000079974	RABL2B	-0.21809	4.608956	0.023078
5069	ENSG00000101444	AHCY	0.229514	5.187178	0.023087
5070	ENSG00000105926	MPP6	-0.25568	4.161984	0.023089
5071	ENSG00000071051	NCK2	-0.21897	5.840399	0.023093
5072	ENSG00000076258	FMO4	-0.31842	1.698485	0.023102
5073	ENSG00000168303	MPLKIP	0.225367	3.759463	0.023136
5074	ENSG00000137040	RANBP6	-0.17626	6.439912	0.023138
5075	ENSG00000151806	GUF1	0.180882	4.988598	0.023221
5076	ENSG00000183579	ZNRF3	-0.52082	-1.07232	0.023221
5077	ENSG00000205189	ZBTB10	-0.35361	5.933327	0.023233
5078	ENSG00000007047	MARK4	0.259855	4.302216	0.023242
5079	ENSG00000187653	TMSB4XP8	0.552758	-0.8531	0.023255
5080	ENSG00000142599	RERE	-0.29492	6.105968	0.023262
5081	ENSG00000247626	MARS2	0.220424	3.579878	0.023264
5082	ENSG00000003056	M6PR	0.242317	7.230547	0.023283
5083	ENSG00000108773	KAT2A	-0.21762	4.994937	0.023293
5084	ENSG00000180822	PSMG4	-0.23674	3.268208	0.02332
5085	ENSG00000173480	ZNF417	-0.25973	4.11606	0.023335
5086	ENSG00000242060	RPS3AP49	-0.37299	1.774337	0.023335
5087	ENSG00000144485	HES6	0.40592	1.342236	0.023337
5088	ENSG00000145604	SKP2	0.191911	3.972762	0.023337
5089	ENSG00000102805	CLN5	-0.18285	4.202163	0.023354
5090	ENSG00000258810		-0.83063	-1.48271	0.023358
5091	ENSG00000184575	XPOT	0.143867	6.662197	0.023366
5092	ENSG00000228903	RASA4CP	-0.3158	3.378519	0.023371
5093	ENSG00000143486	EIF2D	-0.16674	5.593916	0.023376
5094	ENSG00000198890	PRMT6	0.229185	3.692743	0.023402
5095	ENSG00000103356	EARS2	0.201381	3.306628	0.023421
5096	ENSG00000064225	ST3GAL6	-0.4453	3.020207	0.023444
5097	ENSG00000112599	GUCA1B	-0.39575	-0.26822	0.023489
5098	ENSG00000270055		-0.39984	3.704589	0.023489
5099	ENSG00000156564	LRFN2	-0.82281	-1.52346	0.023489
5100	ENSG00000053702	NRIP2	-0.31899	2.531958	0.023545
5101	ENSG00000196083	IL1RAP	-0.39864	4.993353	0.023545

5102	ENSG00000179841	AKAP5	0.571553	4.754605	0.023556
5103	ENSG00000273002		0.35991	-0.5693	0.023556
5104	ENSG00000086589	RBM22	0.16879	6.482016	0.023556
5105	ENSG00000122543	OCM	-0.52915	-0.7508	0.023556
5106	ENSG00000149516	MS4A3	-0.64884	2.877073	0.023575
5107	ENSG00000052802	MSMO1	0.323767	4.372541	0.023594
5108	ENSG00000115282	TTC31	0.129683	5.113213	0.0236
5109	ENSG00000157657	ZNF618	-0.59137	-0.01267	0.023603
5110	ENSG00000139178	C1RL	-0.45397	4.019698	0.023678
5111	ENSG00000232926		-0.51632	-0.56438	0.023743
5112	ENSG00000105246	EBI3	0.689956	0.03787	0.023748
5113	ENSG00000218208		-0.3	5.459403	0.023756
5114	ENSG00000188647	PTAR1	-0.21353	7.350356	0.023779
5115	ENSG00000070882	OSBPL3	0.178953	5.822201	0.023816
5116	ENSG00000205871	RPS3AP47	-0.41798	2.12475	0.02382
5117	ENSG00000228784	LINC00954	-0.34897	2.511154	0.023883
5118	ENSG00000182220	ATP6AP2	-0.1878	7.58886	0.023889
5119	ENSG00000103005	USB1	0.281463	5.667663	0.023899
5120	ENSG00000152782	PANK1	-0.42027	0.03627	0.023932
5121	ENSG00000178234	GALNT11	0.24725	5.693048	0.023934
5122	ENSG00000038427	VCAN	-0.57685	9.322031	0.023946
5123	ENSG00000203761	MSTO2P	-0.21619	3.146322	0.023978
5124	ENSG00000185043	CIB1	0.23195	7.057889	0.023985
5125	ENSG00000155660	PDIA4	0.352987	6.242036	0.023993
5126	ENSG00000028116	VRK2	0.266093	4.939428	0.023998
5127	ENSG00000143294	PRCC	0.207261	5.178238	0.024035
5128	ENSG00000259959		-0.32133	2.209979	0.02404
5129	ENSG00000196781	TLE1	-0.33424	3.634523	0.02404
5130	ENSG00000196655	TRAPPC4	0.188037	5.262312	0.024083
5131	ENSG00000255040		0.331277	0.399798	0.024105
5132	ENSG00000160408	ST6GALNAC6	0.244068	6.346148	0.024133
5133	ENSG00000204262	COL5A2	-0.45579	-0.36821	0.024133
5134	ENSG00000253716		-0.39947	0.846875	0.024136
5135	ENSG00000183935	HTR7P1	-0.46402	-0.27431	0.02417
5136	ENSG00000273329		-0.30306	0.92747	0.024189
5137	ENSG00000101365	IDH3B	0.122369	6.398751	0.024197
5138	ENSG00000108439	PNPO	0.388759	2.119942	0.024203
5139	ENSG00000158691	ZSCAN12	0.245633	3.551911	0.024203
5140	ENSG00000116586	LAMTOR2	0.294835	4.411841	0.024224
5141	ENSG00000180211		-0.22815	6.744221	0.024224
5142	ENSG00000127334	DYRK2	-0.20229	7.272094	0.024247
5143	ENSG00000105971	CAV2	-0.70891	-0.3102	0.024249
5144	ENSG00000242485	MRPL20	0.175345	5.413351	0.024253
5145	ENSG00000244313		-0.2185	7.324487	0.024314

5146	ENSG00000074800	ENO1	0.306599	9.101121	0.024332
5147	ENSG00000179476	C14orf28	-0.26376	2.833979	0.024365
5148	ENSG00000112365	ZBTB24	-0.20293	5.31397	0.024396
5149	ENSG00000197635	DPP4	-0.26385	5.060633	0.024424
5150	ENSG00000174080	CTSF	-0.35712	2.849727	0.024424
5151	ENSG00000183735	TBK1	0.269551	6.271239	0.024442
5152	ENSG00000268366		0.578068	-0.84485	0.024455
5153	ENSG00000256771	ZNF253	-0.35345	3.442967	0.024486
5154	ENSG00000213307	RPL18P11	-0.36998	-0.3275	0.024486
5155	ENSG00000133574	GIMAP4	0.471344	7.201971	0.024491
5156	ENSG00000176390	CRLF3	-0.142	6.592397	0.024497
5157	ENSG00000127540	UQCR11	0.207145	5.396877	0.02452
5158	ENSG00000171204	TMEM126B	0.210908	5.026177	0.024656
5159	ENSG00000181444	ZNF467	-0.73816	2.655398	0.024656
5160	ENSG00000179542	SLITRK4	-0.76681	1.39566	0.024658
5161	ENSG00000271430		-0.34976	3.759773	0.024685
5162	ENSG00000196453	ZNF777	0.295975	3.413637	0.024715
5163	ENSG00000104408	EIF3E	-0.19204	9.161745	0.024715
5164	ENSG00000166133	RPUSD2	0.23363	3.161833	0.024722
5165	ENSG00000226415	TPI1P1	0.291073	2.951119	0.024792
5166	ENSG00000144136	SLC20A1	0.218954	6.867884	0.024796
5167	ENSG00000110315	RNF141	-0.30118	5.946641	0.024819
5168	ENSG00000272077		-0.42733	0.338999	0.024888
5169	ENSG00000132356	PRKAA1	-0.17673	7.12853	0.024971
5170	ENSG00000248477		-0.54113	1.283206	0.024983
5171	ENSG00000257103	LSM14A	0.093988	7.929289	0.025
5172	ENSG00000127527	EPS15L1	0.176366	5.228269	0.025041
5173	ENSG00000029364	SLC39A9	0.15087	6.060911	0.025041
5174	ENSG00000166123	GPT2	0.412328	1.248566	0.025047
5175	ENSG00000160213	CSTB	0.26101	6.116597	0.025064
5176	ENSG00000167674		0.23483	4.101048	0.025064
5177	ENSG00000119535	CSF3R	-0.5171	7.642229	0.025087
5178	ENSG00000108270	AATF	0.217965	5.753323	0.02512
5179	ENSG00000185813	PCYT2	0.223199	4.083824	0.02518
5180	ENSG00000196547	MAN2A2	-0.20355	6.343032	0.025197
5181	ENSG00000005379	BZRAP1	0.303537	5.942822	0.025215
5182	ENSG00000119041	GTF3C3	0.16824	5.517201	0.025234
5183	ENSG00000063244	U2AF2	0.177303	6.959282	0.025247
5184	ENSG00000256167	ATF4P4	0.33702	-0.02443	0.025307
5185	ENSG00000117592	PRDX6	0.202403	6.792502	0.02538
5186	ENSG00000160796	NBEAL2	0.238404	7.866217	0.025418
5187	ENSG00000176273	SLC35G1	0.263156	2.026894	0.025464
5188	ENSG00000205937	RNPS1	0.186239	6.842744	0.025527
5189	ENSG00000272502		0.564243	-0.46305	0.025577

5190	ENSG00000249353	NPM1P27	0.248376	3.549752	0.025577
5191	ENSG00000214655	ZSWIM8	0.207341	5.808528	0.025577
5192	ENSG00000232573	RPL3P4	-0.25848	7.318451	0.025577
5193	ENSG00000110844	PRPF40B	-0.31536	1.061094	0.025577
5194	ENSG00000158477	CD1A	-0.82781	-0.19224	0.025577
5195	ENSG00000214784		-0.30409	1.77041	0.025613
5196	ENSG00000228499	TMSB10P1	0.557317	3.852294	0.02562
5197	ENSG00000170385	SLC30A1	0.308746	5.456471	0.02562
5198	ENSG00000022976	ZNF839	-0.17015	4.03361	0.02562
5199	ENSG00000142937	RPS8	-0.22308	10.34148	0.02562
5200	ENSG00000183496	MEX3B	-0.38791	0.422519	0.02562
5201	ENSG00000214465	SMARCE1P6	0.195112	1.878086	0.025623
5202	ENSG00000272556		0.419341	-0.72831	0.025651
5203	ENSG00000183751	TBL3	0.203874	3.895295	0.025667
5204	ENSG00000084073	ZMPSTE24	0.170339	5.604064	0.025667
5205	ENSG00000100612	DHRS7	-0.15757	6.129246	0.025685
5206	ENSG00000173210	ABLIM3	-0.62685	2.568603	0.025719
5207	ENSG00000183155	RABIF	0.213425	4.536791	0.025742
5208	ENSG00000214108	TPT1P5	-0.25889	4.163837	0.025742
5209	ENSG00000055044	NOP58	0.345835	7.145813	0.025748
5210	ENSG00000120333	MRPS14	0.195913	4.004766	0.025783
5211	ENSG00000100226	GTPBP1	0.461341	7.003984	0.025791
5212	ENSG00000160602	NEK8	-0.23877	2.657716	0.025802
5213	ENSG00000211716	TRBV9	0.415715	2.35736	0.025806
5214	ENSG00000004059	ARF5	0.178769	6.21321	0.025835
5215	ENSG00000101574	METTL4	-0.20734	4.545215	0.025835
5216	ENSG00000102221	JADE3	-0.41069	1.674001	0.025835
5217	ENSG00000055732	MCOLN3	-0.56671	0.099317	0.025839
5218	ENSG00000165682	CLEC1B	-0.47228	2.821806	0.025839
5219	ENSG00000158488	CD1E	-0.93293	-0.51594	0.02584
5220	ENSG00000103145	HCFC1R1	0.275648	2.887499	0.025873
5221	ENSG00000112033	PPARD	-0.26168	4.884846	0.025948
5222	ENSG00000135362	PRR5L	0.452262	5.655825	0.025973
5223	ENSG00000175265	GOLGA8A	-0.31913	7.183136	0.025973
5224	ENSG00000051523	CYBA	0.206062	8.014097	0.026008
5225	ENSG00000223511		0.446491	1.010627	0.02605
5226	ENSG00000196557	CACNA1H	-0.45781	0.803768	0.02605
5227	ENSG00000182272	B4GALNT4	-0.67285	-0.80254	0.02605
5228	ENSG00000213261		-0.40126	1.864677	0.026098
5229	ENSG00000243368	MCCC1-AS1	-0.48351	-0.3537	0.026106
5230	ENSG00000143319	ISG20L2	0.24151	5.791911	0.026162
5231	ENSG00000166199	ALKBH3	0.17468	3.655281	0.026193
5232	ENSG00000112972	HMGCS1	0.32748	5.894176	0.026218
5233	ENSG00000197363	ZNF517	-0.20904	2.220514	0.026218

5234	ENSG00000005961	ITGA2B	-0.71605	4.593122	0.026224
5235	ENSG00000180773	SLC36A4	-0.24926	5.041095	0.026246
5236	ENSG00000242951		0.253377	4.926347	0.0263
5237	ENSG00000123329	ARHGAP9	0.209368	7.24936	0.0263
5238	ENSG00000137814	HAUS2	0.206911	5.06601	0.0263
5239	ENSG00000129993	CBFA2T3	-0.38884	3.317132	0.0263
5240	ENSG00000214846		-0.53521	-0.91336	0.0263
5241	ENSG00000184787	UBE2G2	-0.11889	7.113406	0.026305
5242	ENSG00000163435	ELF3	-0.62386	-0.23935	0.026361
5243	ENSG00000230149		0.368112	1.165814	0.026373
5244	ENSG00000166685	COG1	-0.1662	4.834967	0.026379
5245	ENSG00000107560	RAB11FIP2	-0.22132	5.556566	0.026389
5246	ENSG00000160392	C19orf47	0.258036	3.223699	0.026438
5247	ENSG00000176108	CHMP6	0.259383	3.670029	0.026456
5248	ENSG00000183726	TMEM50A	0.156273	7.442206	0.026469
5249	ENSG00000197056	ZMYM1	-0.30121	3.876729	0.026469
5250	ENSG00000173991	TCAP	-0.38553	0.5797	0.026469
5251	ENSG00000171033	PKIA	-0.32149	3.599515	0.026499
5252	ENSG00000260078		-0.54712	1.961697	0.02655
5253	ENSG00000141429	GALNT1	0.15526	7.026718	0.026625
5254	ENSG00000064490	RFXANK	0.225139	4.220758	0.026628
5255	ENSG00000006576	PHTF2	-0.20326	6.044889	0.026628
5256	ENSG00000228434		-0.36143	0.273291	0.026628
5257	ENSG00000272201		-0.26473	1.006103	0.026698
5258	ENSG00000181904	C5orf24	-0.19872	5.696533	0.026749
5259	ENSG00000228960	OR2A9P	-0.34508	1.793274	0.026749
5260	ENSG00000157833	GAREML	0.33648	1.230472	0.026778
5261	ENSG00000262904		0.332443	0.426322	0.026796
5262	ENSG00000134250	NOTCH2	-0.29421	8.093685	0.026796
5263	ENSG00000102189	EEA1	-0.31206	5.542256	0.026808
5264	ENSG00000259295	CSPG4P12	-0.41996	-0.55769	0.026892
5265	ENSG00000259531		0.265379	2.613643	0.02696
5266	ENSG00000112290	WASF1	-0.52614	0.532855	0.02696
5267	ENSG00000251158		-0.88136	0.831459	0.026964
5268	ENSG00000165457	FOLR2	-0.59254	1.344518	0.026996
5269	ENSG00000189233	NUGGC	0.318293	3.011177	0.02705
5270	ENSG00000125966	MMP24	-0.34699	0.680913	0.027054
5271	ENSG00000153767	GTF2E1	0.318938	3.603612	0.027063
5272	ENSG00000161677	JOSD2	0.37939	2.820019	0.027065
5273	ENSG00000132199	ENOSF1	0.238802	4.62378	0.027069
5274	ENSG00000111271	ACAD10	-0.26896	4.133899	0.027086
5275	ENSG00000257433		0.429786	0.293628	0.027116
5276	ENSG00000115290	GRB14	-0.71821	-0.38613	0.027158
5277	ENSG00000198134		0.227601	2.600431	0.027166

5278	ENSG00000229018		0.246405	0.21816	0.027216
5279	ENSG00000105254	TBCB	0.215352	5.605008	0.027222
5280	ENSG00000149809	TM7SF2	0.205077	2.2035	0.027366
5281	ENSG00000100418	DESI1	0.191464	5.475733	0.027366
5282	ENSG00000116251	RPL22	-0.22163	9.427694	0.027467
5283	ENSG00000182134	TDRKH	-0.327	2.338287	0.027467
5284	ENSG00000230783		-0.382	4.986543	0.027525
5285	ENSG00000229897	SEPT7P7	0.216822	3.195474	0.027525
5286	ENSG00000245849	RAD51-AS1	-0.29933	3.325199	0.027578
5287	ENSG00000081026	MAGI3	-0.32548	1.513486	0.027618
5288	ENSG00000105618	PRPF31	0.172373	4.680191	0.027643
5289	ENSG00000263142	LRRC37A17P	-0.28106	3.995416	0.027665
5290	ENSG00000122971	ACADS	0.233467	3.13819	0.027717
5291	ENSG00000250565	ATP6V1E2	-0.37433	2.442578	0.027723
5292	ENSG00000271843		-0.38376	1.131534	0.027775
5293	ENSG00000105248	CCDC94	0.276377	4.350095	0.027776
5294	ENSG00000229227		-0.32151	0.279065	0.02778
5295	ENSG00000198105	ZNF248	-0.35316	3.255879	0.027783
5296	ENSG00000231062		-0.69078	-1.03491	0.027783
5297	ENSG00000256427		-0.56621	0.081475	0.027795
5298	ENSG00000100099	HPS4	-0.16165	5.236512	0.027897
5299	ENSG00000132388	UBE2G1	0.148924	6.639969	0.027934
5300	ENSG00000058056	USP13	-0.24284	3.378744	0.027934
5301	ENSG00000168301	KCTD6	-0.25149	3.135941	0.027958
5302	ENSG00000174579	MSL2	0.265439	7.359206	0.027963
5303	ENSG00000170667	RASA4B	-0.3437	2.284217	0.027963
5304	ENSG00000171471	MAP1LC3B2	0.362484	2.059299	0.027973
5305	ENSG00000184428	TOP1MT	-0.34215	4.438995	0.027973
5306	ENSG00000171777	RASGRP4	-0.53896	4.807305	0.027973
5307	ENSG00000266651		0.471777	-0.56237	0.027976
5308	ENSG00000170734	POLH	0.340067	3.75641	0.027984
5309	ENSG00000156671	SAMD8	-0.23244	6.334804	0.027984
5310	ENSG00000263232	ATP5A1P3	0.173116	2.404798	0.027988
5311	ENSG00000105854	PON2	-0.27395	3.230816	0.027991
5312	ENSG00000213240	NOTCH2NL	-0.20231	4.970133	0.027997
5313	ENSG00000120659	TNFSF11	-0.72532	-0.89536	0.027999
5314	ENSG00000146373	RNF217	-0.58585	2.685945	0.02801
5315	ENSG00000100890	KIAA0391	0.513528	-0.52945	0.028053
5316	ENSG00000253368	TRNP1	-0.64366	0.094481	0.028068
5317	ENSG00000166091	CMTM5	-0.61916	2.233972	0.028103
5318	ENSG00000228847	ATP5G2P4	0.215959	3.12668	0.028117
5319	ENSG00000101187	SLCO4A1	1.138488	1.379313	0.02814
5320	ENSG00000246115		0.238837	1.128017	0.02814
5321	ENSG00000139055	ERP27	-0.26012	3.188654	0.02814

5322	ENSG00000176386	CDC26	0.196924	3.626526	0.028173
5323	ENSG00000239665		-0.24927	3.77027	0.028173
5324	ENSG00000157168	NRG1	-0.86824	1.090307	0.028173
5325	ENSG00000197417	SHPK	0.294855	0.474071	0.028284
5326	ENSG00000168090	COPS6	0.162909	5.633532	0.028332
5327	ENSG00000131746	TNS4	0.626077	-0.90938	0.028366
5328	ENSG00000171502	COL24A1	-0.42187	0.076781	0.028366
5329	ENSG00000256039		0.610867	4.209675	0.028376
5330	ENSG00000238005		-0.51004	-0.8576	0.028376
5331	ENSG00000224861	YBX1P1	0.261546	1.161434	0.028421
5332	ENSG00000089693	MLF2	0.185477	6.182466	0.028421
5333	ENSG00000214894	LINC00243	-0.73395	-0.96539	0.028421
5334	ENSG00000126249	PDCD2L	0.261998	1.329189	0.028438
5335	ENSG00000267102		0.314708	1.356413	0.028467
5336	ENSG00000172578	KLHL6	-0.30404	5.696789	0.028509
5337	ENSG00000182504	CEP97	0.22601	4.843716	0.028515
5338	ENSG00000243431	RPL5P30	-0.26414	3.698692	0.028515
5339	ENSG00000260917		-0.23499	2.900807	0.028518
5340	ENSG00000174130	TLR6	-0.32903	3.928006	0.028518
5341	ENSG00000225712	ATP5G2P1	0.752	-0.41153	0.028523
5342	ENSG00000196967	ZNF585A	-0.3146	2.980202	0.028523
5343	ENSG00000174171		-0.35989	3.657471	0.028531
5344	ENSG00000273266		0.455392	-0.48829	0.028545
5345	ENSG00000090447	TFAP4	-0.33683	2.332671	0.02857
5346	ENSG00000131398	KCNC3	-0.44796	2.430502	0.028574
5347	ENSG00000147604	RPL7	-0.22796	10.47961	0.028607
5348	ENSG00000143110	C1orf162	-0.46245	6.580589	0.028609
5349	ENSG00000138495	COX17	0.24145	4.306036	0.028632
5350	ENSG00000156414	TDRD9	-0.58718	1.71711	0.028651
5351	ENSG00000181982	CCDC149	-0.61771	2.827573	0.028654
5352	ENSG00000170581	STAT2	0.369993	7.332849	0.028658
5353	ENSG00000129465	RIPK3	0.184442	4.242901	0.02869
5354	ENSG00000166024	R3HCC1L	0.178782	5.394643	0.02869
5355	ENSG00000180096	SEPT1	0.192624	6.621938	0.028723
5356	ENSG00000179115	FARSA	0.281481	4.146418	0.028772
5357	ENSG00000213881	NPM1P6	0.235688	1.754138	0.028772
5358	ENSG00000174514	MFSD4	-0.32231	1.627864	0.028772
5359	ENSG00000189136	UBE2Q2P1	-0.4562	0.184201	0.028772
5360	ENSG00000041880	PARP3	0.245437	3.316161	0.028808
5361	ENSG00000115257	PCSK4	-0.43956	-0.28827	0.028863
5362	ENSG00000267519	MIR24-2	-0.5113	5.497745	0.028942
5363	ENSG00000162366	PDZK1IP1	-0.68379	1.814834	0.028945
5364	ENSG00000179967	PPP1R14BP3	0.334792	2.262781	0.02896
5365	ENSG00000182318	ZSCAN22	0.513087	2.048907	0.028988

5366	ENSG00000230701	FBXW4P1	0.444402	0.758834	0.029003
5367	ENSG00000164308	ERAP2	0.48712	7.882752	0.029027
5368	ENSG00000224805	LINC00853	-0.68944	-0.49977	0.029029
5369	ENSG00000267342		-0.34917	0.294603	0.029051
5370	ENSG00000250696		-0.9351	-0.98677	0.029051
5371	ENSG00000265625		-0.36783	0.83956	0.029052
5372	ENSG00000091972	CD200	-0.52137	1.929958	0.029052
5373	ENSG00000169231	THBS3	-0.21714	3.38867	0.029073
5374	ENSG00000214194	LINC00998	0.289821	3.872129	0.029095
5375	ENSG00000189043	NDUFA4	0.200456	6.780975	0.029095
5376	ENSG00000114331	ACAP2	-0.15874	7.65523	0.029095
5377	ENSG00000146247	PHIP	-0.21819	7.855556	0.029095
5378	ENSG00000185591	SP1	-0.17128	7.031345	0.029098
5379	ENSG00000198885	ITPRIPL1	0.336714	3.091417	0.029108
5380	ENSG00000120586	MRC1	-0.68977	-0.58665	0.029148
5381	ENSG00000260000		0.34166	0.005222	0.029154
5382	ENSG00000066044	ELAVL1	0.209788	5.269505	0.029183
5383	ENSG00000256690		-0.44356	0.134019	0.029183
5384	ENSG00000137207	YIPF3	0.181653	5.568863	0.029189
5385	ENSG00000167664	TMIGD2	-0.35814	2.779262	0.029189
5386	ENSG00000196605	ZNF846	-0.33059	2.266822	0.02923
5387	ENSG00000065457	ADAT1	0.18557	4.773739	0.029386
5388	ENSG00000002587	HS3ST1	-0.65964	-0.77649	0.029386
5389	ENSG00000137857	DUOX1	-0.39707	0.215419	0.02948
5390	ENSG00000167523	SPATA33	0.212128	1.534154	0.029502
5391	ENSG00000139182	CLSTN3	0.276123	5.620496	0.029502
5392	ENSG00000224383	C17orf72	-0.24051	2.797435	0.029548
5393	ENSG00000231999		-0.21511	1.732846	0.029627
5394	ENSG00000126467	TSKS	-0.51675	0.172505	0.029627
5395	ENSG00000229809	ZNF688	0.250322	3.344727	0.029766
5396	ENSG00000171224	C10orf35	-0.49311	0.213026	0.029829
5397	ENSG00000261232		0.460611	0.356144	0.029912
5398	ENSG00000249661	TNRC18P1	-0.63166	-1.10598	0.029925
5399	ENSG00000117090	SLAMF1	-0.24327	4.993967	0.029955
5400	ENSG00000223822	EEF1A1P1	-0.37627	1.777539	0.029958
5401	ENSG00000138031	ADCY3	0.288196	3.598871	0.02996
5402	ENSG00000115271	GCA	-0.52164	5.884448	0.029971
5403	ENSG00000261040		-0.53653	-0.94839	0.029971
5404	ENSG00000154734	ADAMTS1	-0.7869	-0.70798	0.029975
5405	ENSG00000108298	RPL19	-0.17823	10.6619	0.029989
5406	ENSG00000182873		-0.37258	1.096164	0.029989
5407	ENSG00000017427	IGF1	0.74178	-0.85863	0.029991
5408	ENSG00000197721	CR1L	-0.5716	0.333877	0.03
5409	ENSG00000259105	RPS3AP4	-0.35579	5.322851	0.030016

5410	ENSG00000269930		-0.545	-0.53154	0.030016
5411	ENSG00000176160	HSF5	-0.36585	0.88319	0.03003
5412	ENSG00000105516	DBP	-0.38779	2.551507	0.030044
5413	ENSG00000124380	SNRNP27	0.152578	5.266026	0.030178
5414	ENSG00000146072	TNFRSF21	-0.56517	1.943134	0.0302
5415	ENSG00000183258	DDX41	0.142533	5.653562	0.030221
5416	ENSG00000164463	CREBRF	-0.22509	7.785019	0.030369
5417	ENSG00000254595		-0.35133	1.211103	0.030369
5418	ENSG00000159713	TPPP3	-0.68718	3.109443	0.030379
5419	ENSG00000243927	MRPS6	0.233265	4.995828	0.03043
5420	ENSG00000108344	PSMD3	0.172643	5.667492	0.030431
5421	ENSG00000198843		0.168961	7.403842	0.030507
5422	ENSG00000270230		-0.36011	3.012714	0.030507
5423	ENSG00000118482	PHF3	-0.24841	7.655085	0.030532
5424	ENSG00000143452	HORMAD1	-0.6414	-0.39883	0.030533
5425	ENSG00000134802	SLC43A3	0.423568	4.164589	0.030545
5426	ENSG00000130312	MRPL34	0.248239	4.143163	0.030546
5427	ENSG00000235912		0.416901	3.042312	0.030593
5428	ENSG00000125740	FOSB	-0.83981	7.580774	0.030618
5429	ENSG00000161103		0.348109	-0.11797	0.030675
5430	ENSG00000141968	VAV1	0.209339	6.830848	0.030675
5431	ENSG00000256223	ZNF10	-0.47479	3.453795	0.030675
5432	ENSG00000272655		0.181399	2.472092	0.030699
5433	ENSG00000111275	ALDH2	-0.52542	5.208239	0.0307
5434	ENSG00000107789	MINPP1	0.33944	2.902226	0.030719
5435	ENSG00000198189	HSD17B11	-0.20991	7.201416	0.030738
5436	ENSG00000123219	CENPK	0.492636	3.947401	0.030747
5437	ENSG00000197857	ZNF44	-0.19852	4.994248	0.030747
5438	ENSG00000254052	IGHVIII-67-4	1.229827	-0.67637	0.030782
5439	ENSG00000105404	RABAC1	0.22343	5.03055	0.030786
5440	ENSG00000039650	PNKP	0.166584	4.65058	0.030799
5441	ENSG00000143494	VASH2	-0.59363	-0.07059	0.030834
5442	ENSG00000131089	ARHGEF9	-0.26242	4.397679	0.030877
5443	ENSG00000101246	ARFRP1	0.193623	4.510861	0.0309
5444	ENSG00000073008	PVR	-0.46975	2.080588	0.030916
5445	ENSG00000136738	STAM	-0.20723	4.746407	0.030929
5446	ENSG00000140993	TIGD7	-0.35186	0.320666	0.03094
5447	ENSG00000226816		-0.73296	-0.03956	0.03094
5448	ENSG00000020633	RUNX3	0.330149	8.777723	0.030946
5449	ENSG00000055070	SZRD1	0.172646	7.093106	0.030977
5450	ENSG00000163737	PF4	-0.55846	4.844388	0.030997
5451	ENSG00000166454	ATMIN	0.100278	6.608198	0.031046
5452	ENSG00000119636	CCDC176	-0.3423	1.064104	0.031104
5453	ENSG00000211623	IGKV2D-26	0.804269	-0.97647	0.031133

5454	ENSG00000229619	MBNL1-AS1	-0.26693	2.244174	0.031133
5455	ENSG00000131149	GSE1	0.16802	6.433023	0.031134
5456	ENSG00000242327		-0.28617	2.077634	0.031156
5457	ENSG00000271550		-0.37841	0.489229	0.031231
5458	ENSG00000025434	NR1H3	0.4309	1.797581	0.031236
5459	ENSG00000172803	SNX32	0.33138	0.906388	0.031236
5460	ENSG00000128692	EIF2S2P4	0.232125	2.546178	0.031259
5461	ENSG00000186522	SEPT10	-0.39343	1.233469	0.031327
5462	ENSG00000119616	FCF1	0.16592	5.15864	0.03136
5463	ENSG00000170584	NUDCD2	0.15631	4.927729	0.03136
5464	ENSG00000183718	TRIM52	-0.16089	5.561314	0.03136
5465	ENSG00000177374	HIC1	0.563118	2.099021	0.031487
5466	ENSG00000131871	VIMP	0.178779	5.511534	0.031493
5467	ENSG00000198231	DDX42	-0.12325	6.905499	0.031513
5468	ENSG00000082014	SMARCD3	-0.39322	4.386622	0.031513
5469	ENSG00000120910	PPP3CC	0.169059	6.103847	0.031525
5470	ENSG00000224857		-0.54507	-0.97407	0.031562
5471	ENSG00000126814	TRMT5	0.244158	2.665964	0.031616
5472	ENSG00000206344	HCG27	-0.53055	-0.48077	0.031616
5473	ENSG00000226543	MYL6P1	0.308062	0.478057	0.031618
5474	ENSG00000169598	DFFB	0.204899	3.648671	0.031618
5475	ENSG00000198730	CTR9	0.257226	6.684653	0.03164
5476	ENSG00000126561	STAT5A	0.334812	6.998877	0.03166
5477	ENSG00000256238		0.245583	1.724692	0.031688
5478	ENSG00000186625	KATNA1	0.130985	4.509212	0.031725
5479	ENSG00000204282	TNRC6C-AS1	-0.3004	5.117566	0.03174
5480	ENSG00000236762		-0.20764	5.172124	0.031785
5481	ENSG00000196150	ZNF250	-0.51428	2.900256	0.031785
5482	ENSG00000152443	ZNF776	-0.21095	4.463064	0.031835
5483	ENSG00000143641	GALNT2	0.243122	5.11328	0.031852
5484	ENSG00000243095	RPL3P10	-0.23557	4.453737	0.03187
5485	ENSG00000138363	ATIC	0.204626	5.308112	0.031972
5486	ENSG00000130024	PHF10	-0.22058	6.685479	0.032026
5487	ENSG00000107719	PALD1	-0.50756	0.098857	0.032029
5488	ENSG00000262155		-0.55514	-0.82032	0.032042
5489	ENSG00000248008	DYNLL1-AS1	0.425317	0.144609	0.032068
5490	ENSG00000184203	PPP1R2	-0.13447	7.43815	0.032068
5491	ENSG00000166477	LEO1	0.277514	4.094019	0.032116
5492	ENSG00000157778	PSMG3	0.167218	3.821725	0.032116
5493	ENSG00000126838	PZP	-0.59927	2.026938	0.032116
5494	ENSG00000140463	BBS4	0.174543	3.961001	0.03212
5495	ENSG00000072422	RHOBTB1	-0.46807	1.708457	0.03212
5496	ENSG00000105993	DNAJB6	0.27121	7.83402	0.032173
5497	ENSG00000117151	CTBS	-0.20784	5.660048	0.032192

5498	ENSG00000135916	ITM2C	0.38671	5.55349	0.032215
5499	ENSG00000182965	NPM1P14	0.235808	1.483611	0.032248
5500	ENSG00000198324	FAM109A	-0.43247	2.238916	0.032281
5501	ENSG00000250575		-0.40537	2.806296	0.032285
5502	ENSG00000110514	MADD	0.296948	7.119713	0.032291
5503	ENSG00000243199		-0.20629	3.174544	0.032311
5504	ENSG00000171681	ATF7IP	0.228444	6.983182	0.032313
5505	ENSG00000228205		-0.20212	4.632283	0.032323
5506	ENSG00000105705	SUGP1	0.16836	4.452195	0.032338
5507	ENSG00000105519	CAPS	-0.25153	4.183846	0.032358
5508	ENSG00000164038	SLC9B2	0.175777	3.795947	0.032358
5509	ENSG00000261247	GOLGA8T	-0.49419	-0.61274	0.032365
5510	ENSG00000160226	C21orf2	-0.23235	3.702985	0.032368
5511	ENSG00000176055	MBLAC2	-0.26952	2.992733	0.032368
5512	ENSG00000171155	C1GALT1C1	0.278071	4.032228	0.032438
5513	ENSG00000151746	BICD1	0.229273	3.128421	0.032438
5514	ENSG00000156313	RPGR	-0.23954	4.109296	0.032452
5515	ENSG00000197279	ZNF165	-0.73369	0.59138	0.032465
5516	ENSG00000261560		0.515293	1.106773	0.032465
5517	ENSG00000254419		-0.38397	0.128617	0.032469
5518	ENSG00000105383	CD33	-0.67707	4.636161	0.032476
5519	ENSG00000257497		-0.26293	1.653291	0.0325
5520	ENSG00000148680	HTR7	-0.46358	0.216163	0.032516
5521	ENSG00000179085	DPM3	0.250925	2.72747	0.032526
5522	ENSG00000161405	IKZF3	0.403833	5.438725	0.032583
5523	ENSG00000003147	ICA1	0.500843	1.942719	0.032598
5524	ENSG00000163606	CD200R1	0.390221	3.902395	0.032598
5525	ENSG00000215068		-0.36035	1.748473	0.032598
5526	ENSG00000166548	TK2	-0.38237	4.161893	0.032598
5527	ENSG00000140830	TXNL4B	0.19413	3.710871	0.032617
5528	ENSG00000269907		0.193445	2.667643	0.032808
5529	ENSG00000107554	DNMBP	-0.28562	3.878312	0.032836
5530	ENSG00000005108	THSD7A	-0.7532	-1.52369	0.032836
5531	ENSG00000173114	LRRN3	-0.6265	2.884125	0.032917
5532	ENSG00000144580	RQCD1	0.206374	6.266016	0.032922
5533	ENSG00000108960	MMD	-0.36172	5.704062	0.032938
5534	ENSG00000154146	NRGN	-0.51827	5.798048	0.032961
5535	ENSG00000071575	TRIB2	0.19355	6.584054	0.033026
5536	ENSG00000261528		0.708545	-0.63795	0.033032
5537	ENSG00000163354	DCST2	-0.38561	-0.66227	0.033032
5538	ENSG00000224152		-0.51317	-0.21891	0.033032
5539	ENSG00000213493	ACTN4P1	0.361298	0.786525	0.03314
5540	ENSG00000070614	NDST1	-0.46076	4.345082	0.033158
5541	ENSG00000100473	COCH	-0.48581	0.741758	0.033158

5542	ENSG00000205808	PPAPDC2	-0.2491	2.54434	0.033174
5543	ENSG00000152332	UHMK1	0.127104	8.573362	0.033193
5544	ENSG00000165934	CPSF2	0.255924	5.747638	0.033271
5545	ENSG00000109272	PF4V1	-0.56397	2.80362	0.033276
5546	ENSG00000140471	LINS	-0.18517	5.986969	0.033282
5547	ENSG00000239198	RPL5P22	-0.26923	1.126675	0.033382
5548	ENSG00000110851	PRDM4	-0.17284	6.012145	0.033406
5549	ENSG00000178074	C2orf69	-0.15529	5.215978	0.033456
5550	ENSG00000068001	HYAL2	-0.43373	1.741446	0.03346
5551	ENSG00000124104	SNX21	-0.39673	1.669786	0.033478
5552	ENSG00000220201	ZGLP1	-0.37242	0.246645	0.033481
5553	ENSG00000220157	HNRNPA1P12	0.240543	0.83778	0.033484
5554	ENSG00000235173	FAM203A	0.382361	1.880199	0.033493
5555	ENSG00000261794	GOLGA8H	-0.52852	-1.11217	0.033496
5556	ENSG00000268061	NAPA-AS1	0.295561	0.4865	0.033537
5557	ENSG00000139117	CPNE8	-0.36136	4.013757	0.033537
5558	ENSG00000232098		-0.39676	1.079169	0.03355
5559	ENSG00000257605		-0.33785	0.335153	0.033593
5560	ENSG00000154822	PLCL2	-0.2204	6.969519	0.033644
5561	ENSG00000124702	KLHDC3	0.187376	5.846696	0.033658
5562	ENSG00000124279	FASTKD3	0.228978	3.678625	0.033705
5563	ENSG00000134278	SPIRE1	-0.34958	1.841034	0.033718
5564	ENSG00000162961	DPY30	0.223684	4.17266	0.033727
5565	ENSG00000172175	MALT1	-0.24659	7.269152	0.033794
5566	ENSG00000153214	TMEM87B	-0.21808	5.285165	0.03385
5567	ENSG00000109906	ZBTB16	-0.4549	2.148779	0.03385
5568	ENSG00000211753	TRBV28	0.777626	2.432068	0.033918
5569	ENSG00000102897	LYRM1	0.181232	4.512422	0.033918
5570	ENSG00000100365	NCF4	-0.37726	5.247714	0.034027
5571	ENSG00000137880	GCHFR	0.246963	3.130518	0.034039
5572	ENSG00000175984	DENND2C	-0.45312	0.735998	0.034051
5573	ENSG00000247157		-0.38256	0.254155	0.034067
5574	ENSG00000131374	TBC1D5	-0.20875	6.267616	0.034073
5575	ENSG00000084676	NCOA1	0.168036	7.305949	0.034079
5576	ENSG00000160783	PMF1	0.221341	4.584431	0.034081
5577	ENSG00000212719	C17orf51	-0.3744	0.759857	0.034082
5578	ENSG00000243660	ZNF487	0.223904	3.573606	0.034112
5579	ENSG00000272325	NUDT3	-0.19237	4.826364	0.034128
5580	ENSG00000156697	UTP14A	0.143296	4.428609	0.034155
5581	ENSG00000251442	LINC01094	-0.61982	-0.0354	0.034161
5582	ENSG00000248472	DDX11L9	-0.63649	-0.62999	0.034218
5583	ENSG00000120137	PANK3	-0.18377	7.243331	0.034246
5584	ENSG00000117408	IPO13	0.230683	3.991469	0.034269
5585	ENSG00000067829	IDH3G	0.183701	5.516481	0.034347

5586	ENSG00000198959	TGM2	0.642312	2.243429	0.034401
5587	ENSG00000235703	LINC00894	-0.29368	2.803569	0.034461
5588	ENSG00000129277	CCL4	0.746315	7.313855	0.03448
5589	ENSG00000182944	EWSR1	0.14573	8.357312	0.03448
5590	ENSG00000121716	PILRB	0.302085	3.213826	0.034563
5591	ENSG00000123415	SMUG1	0.276222	3.24055	0.034585
5592	ENSG00000167074	TEF	-0.26424	2.787235	0.034585
5593	ENSG00000180190	TDRP	-0.6333	-0.14956	0.034652
5594	ENSG00000133138	TBC1D8B	-0.62242	-0.85213	0.034717
5595	ENSG00000126088	UROD	0.187074	5.12359	0.034733
5596	ENSG00000163683	SMIM14	-0.34122	5.337476	0.034849
5597	ENSG00000162643	WDR63	0.598583	0.580186	0.034899
5598	ENSG00000269228		-0.31501	2.55276	0.034899
5599	ENSG00000272746		-0.41233	-0.823	0.03496
5600	ENSG00000264577		-0.26109	2.678233	0.03496
5601	ENSG00000178927	C17orf62	0.26033	6.470353	0.035046
5602	ENSG00000173486	FKBP2	0.207212	4.158806	0.035058
5603	ENSG00000271941		-0.33903	0.111268	0.035088
5604	ENSG00000196663	TECPR2	-0.31284	3.822265	0.035143
5605	ENSG00000114395	CYB561D2	0.181634	4.669737	0.035145
5606	ENSG00000196646	ZNF136	-0.28245	5.036874	0.035177
5607	ENSG00000262951		-0.4694	-0.46639	0.035186
5608	ENSG00000272632		-0.7705	-1.34911	0.035212
5609	ENSG00000106692	FKTN	-0.25675	4.184932	0.035245
5610	ENSG00000158717	RNF166	0.196727	7.217162	0.035273
5611	ENSG00000128191	DGCR8	0.156061	4.906741	0.03529
5612	ENSG00000142197	DOPEY2	-0.21858	4.838904	0.035303
5613	ENSG00000162510	MATN1	-0.54143	-0.0751	0.035341
5614	ENSG00000158062	UBXN11	-0.25984	6.335798	0.035355
5615	ENSG00000240003	RPL7P24	-0.22941	5.485507	0.035388
5616	ENSG00000150477	KIAA1328	-0.25293	3.100939	0.035392
5617	ENSG00000150540	HNMT	-0.44017	3.981214	0.035392
5618	ENSG00000215481	BCRP3	0.331333	1.316721	0.035402
5619	ENSG00000138107	ACTR1A	0.199473	6.39118	0.035404
5620	ENSG00000240143		0.284149	1.425038	0.035429
5621	ENSG00000155330	C16orf87	0.239841	4.41604	0.035438
5622	ENSG00000162542	TMCO4	0.181392	4.05158	0.03546
5623	ENSG00000152061	RABGAP1L	0.16338	7.600575	0.035498
5624	ENSG00000165185	KIAA1958	-0.28127	3.26966	0.035498
5625	ENSG00000197557	TTC30A	-0.29769	0.575456	0.035498
5626	ENSG00000172167	MTBP	0.217143	2.369745	0.035517
5627	ENSG00000213213	CCDC183	-0.45022	-0.75589	0.035517
5628	ENSG00000204131	NHSL2	-0.54481	2.984066	0.035517
5629	ENSG00000177106	EPS8L2	0.315908	2.028123	0.035525

5630	ENSG00000143390	RFX5	0.141082	6.468268	0.035542
5631	ENSG00000049768	FOXP3	-0.39268	1.759827	0.035559
5632	ENSG00000099326	MZF1	-0.29761	4.00769	0.035584
5633	ENSG00000168528	SERINC2	-0.59842	0.203043	0.035584
5634	ENSG00000196214	ZNF766	-0.22977	4.897442	0.035629
5635	ENSG00000213574	LDHAP5	0.486541	1.35785	0.03563
5636	ENSG00000235674	LDHAP2	0.274487	2.39656	0.035641
5637	ENSG00000260563		-0.2469	1.976371	0.035641
5638	ENSG00000111142	METAP2	0.185836	6.529391	0.03567
5639	ENSG00000065054	SLC9A3R2	-0.43304	-0.33249	0.035731
5640	ENSG00000144134	RABL2A	-0.21179	4.138543	0.035765
5641	ENSG00000268849	SIGLEC22P	-0.78474	0.165238	0.035765
5642	ENSG00000229447		-0.37896	-0.10233	0.035788
5643	ENSG00000100354	TNRC6B	-0.20569	6.933314	0.035855
5644	ENSG00000230383		-0.23402	2.078251	0.035865
5645	ENSG00000163512	AZI2	0.203878	4.485675	0.035883
5646	ENSG00000234465	PINLYP	0.417877	-0.51173	0.035933
5647	ENSG00000227825	SLC9A7P1	-0.54739	2.379534	0.035933
5648	ENSG00000189077	TMEM120A	0.201559	4.623874	0.035943
5649	ENSG00000128284	APOL3	0.25773	6.819244	0.035989
5650	ENSG00000226221		-0.2139	6.012313	0.036001
5651	ENSG00000264868		-0.80751	-0.10891	0.036003
5652	ENSG00000153250	RBMS1	-0.16908	7.151192	0.036022
5653	ENSG00000116985	BMP8B	-0.2463	2.156117	0.036145
5654	ENSG00000166667	SPDYE6	-0.34095	1.0666	0.036183
5655	ENSG00000169902	TPST1	-0.36542	1.264398	0.036183
5656	ENSG00000267326		0.237477	3.103901	0.036233
5657	ENSG00000095739	BAMBI	-0.51589	-0.99704	0.036233
5658	ENSG00000149212	SESN3	-0.46617	7.961009	0.036237
5659	ENSG00000239736	CEACAMP3	-0.9424	-0.56433	0.036237
5660	ENSG00000196510	ANAPC7	0.147202	4.795097	0.036241
5661	ENSG00000074935	TUBE1	-0.22728	3.987313	0.036247
5662	ENSG00000224914	LINC00863	-0.24447	1.660602	0.036315
5663	ENSG00000163221	S100A12	-0.52323	5.84479	0.036328
5664	ENSG00000139651	ZNF740	-0.18646	5.38613	0.03634
5665	ENSG00000166126	AMN	-0.48158	-0.73516	0.03634
5666	ENSG00000143772	ITPKB	-0.28603	7.162844	0.036358
5667	ENSG00000271721		-0.3985	0.166652	0.036367
5668	ENSG00000178809	TRIM73	-0.33027	0.748255	0.036444
5669	ENSG00000179965	ZNF771	0.445553	-0.70564	0.036454
5670	ENSG00000198270	TMEM116	-0.28849	3.526429	0.036478
5671	ENSG00000164172	MOCS2	0.135462	4.623807	0.036491
5672	ENSG00000158716	DUSP23	0.420147	2.885949	0.036515
5673	ENSG00000239917	RPS10P16	-0.23299	1.299155	0.036527

5674	ENSG00000214783	POLR2J4	-0.23902	2.182924	0.03653
5675	ENSG00000165661	QSOX2	-0.23376	5.145512	0.036543
5676	ENSG00000163818	LZTFL1	-0.1923	4.195166	0.036644
5677	ENSG00000198315	ZKSCAN8	-0.25013	6.395494	0.036685
5678	ENSG00000079482	OPHN1	-0.63905	-0.04099	0.036708
5679	ENSG00000213309	RPL9P18	-0.40906	2.775919	0.036732
5680	ENSG00000127774	EMC6	0.443061	-0.08208	0.036877
5681	ENSG00000233057	EEF1A1P14	-0.2089	5.657608	0.03688
5682	ENSG00000124574	ABCC10	0.208593	4.768318	0.037019
5683	ENSG00000127837	AAMP	0.163679	5.712019	0.037041
5684	ENSG00000196388	INCA1	-0.38398	0.399507	0.037041
5685	ENSG00000111843	TMEM14C	-0.28699	5.188265	0.037088
5686	ENSG00000117394	SLC2A1	0.287008	5.723159	0.037088
5687	ENSG00000073584	SMARCE1	0.123474	6.397705	0.037088
5688	ENSG00000238072		0.200669	3.048321	0.0371
5689	ENSG00000158457	TSPAN33	-0.30035	4.143001	0.037129
5690	ENSG00000071054	MAP4K4	-0.15325	7.503377	0.037145
5691	ENSG00000112996	MRPS30	0.146119	5.03706	0.037147
5692	ENSG00000213512	GBP7	0.539492	-0.56737	0.037165
5693	ENSG00000215146		-0.69697	-0.96547	0.037184
5694	ENSG00000172007	RAB33B	-0.18511	4.402532	0.037194
5695	ENSG00000146463	ZMYM4	-0.15016	5.629552	0.037262
5696	ENSG00000216938	RPL7P58	-0.30036	2.058988	0.037274
5697	ENSG00000260306		-0.40705	0.866122	0.037355
5698	ENSG00000112305	SMAP1	0.129155	3.672295	0.037357
5699	ENSG00000173209	AHSA2	-0.22964	6.416175	0.037431
5700	ENSG00000185414	MRPL30	0.193928	4.149622	0.037443
5701	ENSG00000228224	NACAP1	-0.16073	6.142701	0.037443
5702	ENSG00000105220	GPI	0.254545	6.667299	0.037455
5703	ENSG00000119714	GPR68	0.439345	3.402849	0.037491
5704	ENSG00000131100	ATP6V1E1	0.166763	6.675032	0.037491
5705	ENSG00000233822	HIST1H2BN	0.326702	0.935885	0.037542
5706	ENSG00000126522	ASL	0.164278	3.562856	0.037542
5707	ENSG00000124787	RPP40	0.32085	1.296344	0.037543
5708	ENSG00000104299	INTS9	0.180761	4.155216	0.037543
5709	ENSG00000239779	WBP1	-0.22946	3.22717	0.037543
5710	ENSG00000258634		-0.22957	2.376781	0.037543
5711	ENSG00000197535	MYO5A	-0.1567	7.015788	0.03755
5712	ENSG00000127399	LRRC61	0.375247	1.714598	0.037587
5713	ENSG00000225828	FAM229A	-0.24969	2.467163	0.037689
5714	ENSG00000204536	CCHCR1	0.362607	0.095198	0.037714
5715	ENSG00000181135	ZNF707	0.203951	3.513734	0.037775
5716	ENSG00000179950	PUF60	0.208863	6.21012	0.037824
5717	ENSG00000236090	LDHAP3	0.500153	1.6045	0.037886

5718	ENSG00000130764	LRRC47	0.156511	4.988081	0.037897
5719	ENSG00000171940	ZNF217	0.250202	6.954606	0.037985
5720	ENSG00000273271		-0.29593	2.009857	0.038014
5721	ENSG00000143543	JTB	0.147953	6.809163	0.038128
5722	ENSG00000120889	TNFRSF10B	-0.22068	6.441837	0.038132
5723	ENSG00000104894	CD37	-0.18012	7.661468	0.038148
5724	ENSG00000088726	TMEM40	-0.53312	2.542744	0.038163
5725	ENSG00000253729	PRKDC	0.173513	6.981003	0.038241
5726	ENSG00000240695		0.211667	1.068438	0.038246
5727	ENSG00000181924	COA4	0.174253	4.089067	0.03825
5728	ENSG00000165644	COMTD1	0.397595	1.535876	0.038262
5729	ENSG00000025708	TYMP	0.477069	7.100581	0.038263
5730	ENSG00000227403		0.325179	2.156456	0.038292
5731	ENSG00000100591	AHSA1	0.297852	5.66123	0.038333
5732	ENSG00000235314	LINC00957	-0.32194	0.650178	0.038339
5733	ENSG00000256229	ZNF486	-0.32191	2.624367	0.038378
5734	ENSG00000266910		-0.41171	0.202191	0.038426
5735	ENSG00000145495	MARCH6	-0.20579	7.872122	0.038445
5736	ENSG00000196151	WDSUB1	-0.2115	3.274338	0.038511
5737	ENSG00000130244	FAM98C	0.170342	4.042806	0.038532
5738	ENSG00000223745		-0.29711	4.837026	0.038532
5739	ENSG00000268947		-0.43678	-0.41906	0.038556
5740	ENSG00000143727	ACP1	0.129105	6.594799	0.038585
5741	ENSG00000120318	ARAP3	-0.35841	3.286803	0.038676
5742	ENSG00000084070	SMAP2	-0.23769	8.373687	0.038677
5743	ENSG00000143013	LMO4	0.160187	4.959184	0.038727
5744	ENSG00000269553		-0.60471	-0.83228	0.038729
5745	ENSG00000179240		-0.43304	0.710822	0.038736
5746	ENSG00000158428	C2orf62	-0.50423	-0.62042	0.038764
5747	ENSG00000074370	ATP2A3	0.205988	7.912817	0.038775
5748	ENSG00000005075	POLR2J	0.162693	4.835327	0.038775
5749	ENSG00000170445	HARS	0.113856	5.795861	0.038818
5750	ENSG00000196074	SYCP2	-0.37002	3.188429	0.038818
5751	ENSG00000198851	CD3E	0.281627	8.085818	0.038868
5752	ENSG00000133935	C14orf1	0.155215	4.185735	0.038898
5753	ENSG00000114853	ZBTB47	-0.49156	1.194114	0.038916
5754	ENSG00000227939	RPL3P2	-0.23799	2.122004	0.038919
5755	ENSG00000227765		0.248064	4.778789	0.038934
5756	ENSG00000186501	TMEM222	0.166365	4.892835	0.038948
5757	ENSG00000140543	DET1	-0.25165	1.792572	0.038983
5758	ENSG00000181143	MUC16	-0.91226	-2.10069	0.038985
5759	ENSG00000189308	LIN54	0.169795	4.939871	0.039085
5760	ENSG00000141294	LRRC46	0.261861	0.153521	0.039087
5761	ENSG00000211460	TSN	0.117798	6.468403	0.039087

5762	ENSG00000187266	EPOR	-0.26762	2.392566	0.039133
5763	ENSG00000231177	LINC00852	-0.33061	2.249591	0.039161
5764	ENSG00000197136	PCNXL3	0.180266	5.934192	0.039164
5765	ENSG00000239653	PSMD6-AS2	-0.40563	2.935428	0.039164
5766	ENSG00000169062	UPF3A	0.185304	5.497054	0.039174
5767	ENSG00000130748	TMEM160	0.521977	1.489519	0.039191
5768	ENSG00000151445	VIPAS39	0.176623	4.266052	0.039288
5769	ENSG00000169019	COMMD8	0.22507	4.928738	0.039291
5770	ENSG00000241852	C8orf58	-0.26745	2.482007	0.039291
5771	ENSG00000106263	EIF3B	0.157323	6.77908	0.03932
5772	ENSG00000230163		-0.27425	0.381167	0.03948
5773	ENSG00000261098		-0.28925	2.666845	0.03948
5774	ENSG00000155858	LSM11	-0.21813	3.118406	0.039486
5775	ENSG00000008516	MMP25	-0.60568	3.215024	0.039513
5776	ENSG00000167565	SERTAD3	0.372186	5.232514	0.039531
5777	ENSG00000214941	ZSWIM7	-0.16066	3.92314	0.039531
5778	ENSG00000255031		-0.30153	1.693593	0.039536
5779	ENSG00000112237	CCNC	0.166921	6.002335	0.039629
5780	ENSG00000141837	CACNA1A	0.524375	-0.72062	0.03964
5781	ENSG00000261754		0.57636	1.147233	0.03965
5782	ENSG00000035862	TIMP2	-0.46848	4.931205	0.03966
5783	ENSG00000118508	RAB32	-0.38015	4.619049	0.039663
5784	ENSG00000116922	C1orf109	-0.19845	3.188949	0.039672
5785	ENSG00000143633	C1orf131	0.215176	3.796918	0.039742
5786	ENSG00000225423	TNPO1P1	0.423206	-0.8178	0.039744
5787	ENSG00000010165	METTL13	0.33369	3.723431	0.039744
5788	ENSG00000214199	EEF1A1P12	-0.20248	5.945998	0.039744
5789	ENSG00000122729	ACO1	-0.23536	3.204343	0.039744
5790	ENSG00000241106	HLA-DOB	-0.42028	1.197975	0.039744
5791	ENSG00000147852	VLDLR	-0.48987	0.174295	0.039744
5792	ENSG00000196998	WDR45	0.115634	5.58757	0.039787
5793	ENSG00000268913		-0.3516	1.210939	0.039867
5794	ENSG00000078814	MYH7B	-0.33356	0.115513	0.039894
5795	ENSG00000167394	ZNF668	0.29561	2.728302	0.039946
5796	ENSG00000244480		-0.33438	1.102554	0.039948
5797	ENSG00000174500	GCSAM	-0.3568	3.545165	0.03995
5798	ENSG00000183605	SFXN4	0.238052	3.27092	0.039976
5799	ENSG00000129048	ACKR4	-0.38297	-0.09849	0.040013
5800	ENSG00000167986	DDB1	0.191686	7.083384	0.040024
5801	ENSG00000228300	C19orf24	0.260429	3.891093	0.04005
5802	ENSG00000175322	ZNF519	-0.25634	1.800689	0.040064
5803	ENSG00000113231	PDE8B	-0.32293	1.216436	0.04008
5804	ENSG00000143499	SMYD2	-0.15978	5.256242	0.040112
5805	ENSG00000258357		-0.78271	0.909366	0.040149

5806	ENSG00000136449	MYCBPAP	0.572763	-1.04157	0.040198
5807	ENSG00000164620	RELL2	0.280772	2.328849	0.040202
5808	ENSG00000229833	PET100	0.20469	4.323416	0.040202
5809	ENSG00000246228	CASC8	-0.97	-2.14516	0.040206
5810	ENSG00000141002	TCF25	0.172309	6.861365	0.040251
5811	ENSG00000173198	CYSLTR1	-0.33472	4.626454	0.040288
5812	ENSG00000229132	EIF4A1P10	0.212735	3.31931	0.040323
5813	ENSG00000262519	TXNP4	0.358634	1.737311	0.040326
5814	ENSG00000133247	SUV420H2	0.254476	2.797752	0.040326
5815	ENSG00000225972	MTND1P23	-0.29464	9.620117	0.040387
5816	ENSG00000237772		-0.66111	-1.28854	0.040393
5817	ENSG00000166348	USP54	-0.21858	2.158213	0.040394
5818	ENSG00000146833	TRIM4	-0.19397	5.106911	0.040415
5819	ENSG00000129007	CALML4	-0.1653	4.850565	0.04042
5820	ENSG00000226823		0.258087	0.060789	0.040435
5821	ENSG00000246250		-0.62538	-0.91176	0.040435
5822	ENSG00000152556	PFKM	-0.17895	3.02832	0.040481
5823	ENSG00000260686		-0.39897	0.615701	0.040487
5824	ENSG00000138678	AGPAT9	-0.40064	3.814131	0.04049
5825	ENSG00000203734	ECT2L	-0.44925	-0.8706	0.04049
5826	ENSG00000237440	ZNF737	-0.35239	3.259903	0.040524
5827	ENSG00000131979	GCH1	0.338775	7.189045	0.040566
5828	ENSG00000117751	PPP1R8	0.198138	5.910242	0.040566
5829	ENSG00000067836	ROGDI	-0.49656	4.027526	0.040566
5830	ENSG00000115183	TANC1	-0.63479	-0.52173	0.040566
5831	ENSG00000211785	TRAV12-1	0.363159	1.951929	0.040605
5832	ENSG00000133808	MICALCL	-0.5385	1.545492	0.040605
5833	ENSG00000163513	TGFBR2	-0.17085	8.147296	0.040683
5834	ENSG00000171790	SLFNL1	0.500189	0.390854	0.040715
5835	ENSG00000140105	WARS	0.444015	8.58902	0.040728
5836	ENSG00000175221	MED16	0.301609	4.31409	0.040787
5837	ENSG00000236137		0.489208	-0.61195	0.040795
5838	ENSG00000228265		0.383135	-0.70875	0.040922
5839	ENSG00000137166	FOXP4	0.329601	3.740914	0.040932
5840	ENSG00000134058	CDK7	0.193721	4.444096	0.040961
5841	ENSG00000181163	NPM1	0.195539	8.879266	0.040963
5842	ENSG00000114857	NKTR	-0.27646	8.613869	0.041017
5843	ENSG00000036448	MYOM2	-1.28551	4.054862	0.041039
5844	ENSG00000100100	PIK3IP1	-0.3483	8.045251	0.041078
5845	ENSG00000108961	RANGRF	0.20926	3.210473	0.041153
5846	ENSG00000175166	PSMD2	0.178732	6.9212	0.041166
5847	ENSG00000184967	NOC4L	0.238313	3.275044	0.041169
5848	ENSG00000136811	ODF2	0.17753	5.293388	0.041171
5849	ENSG00000177854	TMEM187	0.334175	0.964419	0.041233

5850	ENSG00000136802	LRRC8A	0.247625	5.198631	0.041254
5851	ENSG00000167123	CERCAM	0.505073	2.115482	0.041266
5852	ENSG00000238084		0.39186	-0.28749	0.041266
5853	ENSG00000108771	DHX58	0.383562	4.314981	0.041266
5854	ENSG00000233968		-0.69128	0.116046	0.041266
5855	ENSG00000060069	CTDP1	0.323614	5.105225	0.041286
5856	ENSG00000116106	EPHA4	-0.48832	5.187707	0.041312
5857	ENSG00000165819	METTL3	-0.21024	5.490191	0.041348
5858	ENSG00000241057		-0.36751	-0.29314	0.041406
5859	ENSG00000198546	ZNF511	0.182927	4.393618	0.041529
5860	ENSG00000134551	PRH2	-0.43902	-1.05138	0.041549
5861	ENSG00000130489	SCO2	0.457348	4.748073	0.041557
5862	ENSG00000148908	RGS10	0.196832	6.443304	0.041578
5863	ENSG00000116874	WARS2	0.367306	3.309871	0.04159
5864	ENSG00000134644	PUM1	-0.13938	6.704441	0.041616
5865	ENSG00000152689	RASGRP3	0.282453	4.434386	0.041616
5866	ENSG00000144554	FANCD2	0.183606	4.105486	0.041669
5867	ENSG00000120697	ALG5	0.166883	4.754192	0.041669
5868	ENSG00000112339	HBS1L	0.168384	5.976424	0.041692
5869	ENSG00000255518		-0.43971	0.044489	0.041721
5870	ENSG00000149273	RPS3	-0.18127	10.83357	0.041745
5871	ENSG00000164056	SPRY1	-0.43717	1.866895	0.041754
5872	ENSG00000142621	FHAD1	-0.59384	-0.2708	0.041854
5873	ENSG00000178301	AQP11	-0.35473	0.235746	0.041862
5874	ENSG00000115677	HDLBP	0.209521	7.277689	0.041886
5875	ENSG00000237943	PRKCQ-AS1	-0.21785	5.152388	0.041886
5876	ENSG00000095319	NUP188	0.197065	5.617954	0.04191
5877	ENSG00000005700	IBTK	0.157453	6.316461	0.041918
5878	ENSG00000162441	LZIC	0.145565	4.670682	0.042021
5879	ENSG00000042753	AP2S1	0.28144	5.072333	0.04207
5880	ENSG00000165775	FUNDC2	0.15823	6.076648	0.042111
5881	ENSG00000099725	PRKY	0.258282	4.114152	0.042112
5882	ENSG00000122335	SERAC1	-0.21737	2.779966	0.042112
5883	ENSG00000211747	TRBV20-1	0.280059	3.875789	0.042121
5884	ENSG00000117289	TXNIP	-0.43566	10.62582	0.042145
5885	ENSG00000186350	RXRA	-0.33131	6.38684	0.042198
5886	ENSG00000076351	SLC46A1	-0.41709	0.150185	0.042211
5887	ENSG00000215835		0.243715	1.244461	0.042222
5888	ENSG00000260261		-0.38037	0.12365	0.042232
5889	ENSG00000184402	SS18L1	0.187004	4.413838	0.042298
5890	ENSG00000187688	TRPV2	0.245969	5.778468	0.042325
5891	ENSG00000196497	IPO4	0.218019	1.472525	0.042339
5892	ENSG00000158042	MRPL17	0.222517	3.522662	0.042349
5893	ENSG00000257122	RRN3P3	0.196492	3.343731	0.042398

5894	ENSG00000067533	RRP15	-0.22981	4.385283	0.042412
5895	ENSG00000138756	BMP2K	-0.23036	4.011875	0.042438
5896	ENSG00000183793	NPIPA5	-0.22569	2.933788	0.042458
5897	ENSG00000101166	SLMO2	0.169054	6.72356	0.042482
5898	ENSG00000236946	HNRNPA1P70	-0.48864	-0.71627	0.042496
5899	ENSG00000203778	FAM229B	-0.31442	-0.03247	0.042509
5900	ENSG00000137817	PARP6	-0.11856	6.216722	0.042517
5901	ENSG00000229161	TCP1P1	0.188641	1.944744	0.042523
5902	ENSG00000013374	NUB1	0.178867	6.75656	0.042589
5903	ENSG00000136827	TOR1A	0.209898	5.587616	0.042602
5904	ENSG00000230850		-0.54033	-0.56945	0.04266
5905	ENSG00000114473	IQCG	-0.21282	3.104315	0.042729
5906	ENSG00000102003	SYP	0.334158	-0.40135	0.042738
5907	ENSG00000113384	GOLPH3	0.195854	6.728131	0.042744
5908	ENSG00000143178	TBX19	-0.24957	2.322992	0.042744
5909	ENSG00000270195		0.331907	-0.06221	0.042758
5910	ENSG00000100316	RPL3	-0.19765	11.36546	0.04287
5911	ENSG00000132514	CLEC10A	-0.59375	3.316496	0.042894
5912	ENSG00000162889	MAPKAPK2	0.232321	7.819397	0.042897
5913	ENSG00000205238	SPDYE2	-0.33187	1.99918	0.042907
5914	ENSG00000135390	ATP5G2	0.168421	7.646478	0.042936
5915	ENSG00000226102	SEPT7P3	0.183047	3.596479	0.042995
5916	ENSG00000150455	TIRAP	-0.17072	2.747696	0.042995
5917	ENSG00000179031		-0.28864	1.489783	0.043023
5918	ENSG00000259918	NDUFA5P11	0.263682	1.497791	0.043038
5919	ENSG00000026950	BTN3A1	0.172323	8.232953	0.043045
5920	ENSG00000120555	SEPT7P9	0.200582	1.656375	0.043057
5921	ENSG00000176903	PNMA1	-0.18017	4.76827	0.043067
5922	ENSG00000248278	SUMO2P17	0.222865	1.602593	0.043077
5923	ENSG00000065427	KARS	0.140193	7.454425	0.043119
5924	ENSG00000244754	N4BP2L2	-0.15862	7.708756	0.043119
5925	ENSG00000259612	EEF1A1P22	-0.2028	5.781493	0.043119
5926	ENSG00000188690	UROS	0.214666	4.528619	0.043206
5927	ENSG00000175471	MCTP1	-0.31747	5.337654	0.043212
5928	ENSG00000163902	RPN1	0.173187	7.581488	0.043236
5929	ENSG00000230395		0.209515	0.896576	0.043247
5930	ENSG00000257275		-0.84922	0.329727	0.043278
5931	ENSG00000237940		-0.36232	2.083101	0.043304
5932	ENSG00000259577		-0.38202	0.814804	0.043319
5933	ENSG00000070214	SLC44A1	-0.20001	5.489238	0.04345
5934	ENSG00000198286	CARD11	0.194747	6.846981	0.043475
5935	ENSG00000107551	RASSF4	-0.46143	5.570628	0.043544
5936	ENSG00000186205	MARC1	-0.80373	2.429612	0.043562
5937	ENSG00000107372	ZFAND5	-0.17194	8.202906	0.043647

5938	ENSG00000114125	RNF7	0.164518	6.077785	0.043683
5939	ENSG00000240401		-0.41367	-0.78398	0.043683
5940	ENSG00000225889		-0.31994	3.255074	0.043701
5941	ENSG00000174083	PIK3R6	-0.3582	2.086885	0.04371
5942	ENSG00000111860	CEP85L	-0.26901	6.715708	0.043755
5943	ENSG00000172830	SSH3	-0.24133	3.020217	0.043811
5944	ENSG00000260412		-0.83561	-1.8943	0.043826
5945	ENSG00000141576	RNF157	-0.25948	4.802847	0.04384
5946	ENSG00000235768	BRD7P5	0.318146	-0.02977	0.043873
5947	ENSG00000135441	BLOC1S1	0.24747	5.092101	0.043922
5948	ENSG00000148660	CAMK2G	-0.18681	5.561387	0.043922
5949	ENSG00000061918	GUCY1B3	-0.3959	2.709683	0.043951
5950	ENSG00000138668	HNRNPD	0.122524	7.922757	0.043999
5951	ENSG00000234036	TXNP6	0.398904	-0.7739	0.04409
5952	ENSG00000071073	MGAT4A	-0.264	8.013675	0.044158
5953	ENSG00000177963	RIC8A	0.164814	6.4461	0.044191
5954	ENSG00000179364	PACS2	-0.19132	4.878761	0.044191
5955	ENSG00000218891	ZNF579	-0.35954	0.595649	0.044196
5956	ENSG00000151491	EPS8	-0.3596	2.64489	0.044214
5957	ENSG00000198223	CSF2RA	-0.35346	3.441429	0.04426
5958	ENSG00000258704		0.299076	0.538762	0.044264
5959	ENSG00000152284	TCF7L1	-0.33264	0.130868	0.044264
5960	ENSG00000118894	FAM86A	0.281053	1.07851	0.044451
5961	ENSG00000253954	HMGN1P38	0.575548	-1.20072	0.04446
5962	ENSG00000243536	ANTXRLP1	0.453836	0.488803	0.044533
5963	ENSG00000032219	ARID4A	-0.19664	6.405439	0.044572
5964	ENSG00000236397	DDX11L2	-0.6637	-0.54436	0.044591
5965	ENSG00000126768	TIMM17B	0.149889	4.720686	0.044655
5966	ENSG00000042317	SPATA7	-0.32834	1.315264	0.044661
5967	ENSG00000228544	CCDC183-AS1	-0.36071	0.18557	0.044661
5968	ENSG00000182324	KCNJ14	-0.37562	-0.41127	0.044719
5969	ENSG00000122435	TRMT13	-0.27553	4.487244	0.044726
5970	ENSG00000138382	METTL5	0.212212	4.095579	0.044741
5971	ENSG00000131791	PRKAB2	-0.1813	5.011042	0.044748
5972	ENSG00000251002		-0.36432	0.369794	0.044748
5973	ENSG00000101898		0.220274	0.186699	0.04476
5974	ENSG00000259706	HSP90B2P	0.220318	4.404889	0.044815
5975	ENSG00000020129	NCDN	0.314552	3.714359	0.044832
5976	ENSG00000249673	NOP14-AS1	0.167822	3.630081	0.044914
5977	ENSG00000231799		0.257891	0.532793	0.044947
5978	ENSG00000101745	ANKRD12	-0.29734	8.121967	0.044987
5979	ENSG00000148219	ASTN2	-0.4271	-0.03459	0.045002
5980	ENSG00000140400	MAN2C1	-0.20023	6.641677	0.045007
5981	ENSG00000105185	PDCD5	0.156143	4.782004	0.045025

5982	ENSG00000260521		-0.22303	4.39954	0.045025
5983	ENSG00000153066	TXNDC11	0.228813	6.515109	0.045086
5984	ENSG00000107960	OBFC1	-0.341	4.362767	0.045101
5985	ENSG00000267319		0.552189	0.568896	0.04512
5986	ENSG00000180979	LRRC57	0.168753	4.327872	0.045123
5987	ENSG00000138182	KIF20B	0.378073	4.811475	0.045142
5988	ENSG00000164961	KIAA0196	0.15949	5.752346	0.045171
5989	ENSG00000197498	RPF2	0.214253	3.713501	0.045252
5990	ENSG00000196296	ATP2A1	0.332121	-0.44105	0.045289
5991	ENSG00000174917	C19orf70	0.192973	4.208967	0.045331
5992	ENSG00000150456	N6AMT2	0.324794	0.815336	0.045332
5993	ENSG00000189223	PAX8-AS1	-0.85724	3.245805	0.04537
5994	ENSG00000185201	IFITM2	0.347655	8.15069	0.045381
5995	ENSG00000137509	PRCP	-0.25298	6.519495	0.045426
5996	ENSG00000089327	FXYD5	-0.22546	7.457405	0.045503
5997	ENSG00000267279		-0.64273	-0.27043	0.045503
5998	ENSG00000198689	SLC9A6	-0.13152	5.179821	0.045567
5999	ENSG00000205208	C4orf46	0.206392	3.873334	0.04561
6000	ENSG00000234961		-0.44246	-0.1643	0.045639
6001	ENSG00000267858		-0.41046	-0.51222	0.045667
6002	ENSG00000256720		-0.50641	1.816242	0.045737
6003	ENSG00000137970	RPL7P9	-0.2178	5.952949	0.045825
6004	ENSG00000183748		-0.69417	-0.52766	0.045874
6005	ENSG00000196459	TRAPPC2	0.138143	4.727346	0.045906
6006	ENSG00000225439	BOLA3-AS1	-0.35787	-0.69521	0.046162
6007	ENSG00000204872		-0.61104	-1.23307	0.046162
6008	ENSG00000171302	CANT1	0.18997	5.299163	0.046172
6009	ENSG00000088035	ALG6	0.153851	4.520978	0.046193
6010	ENSG00000228492	RAB11FIP1P1	-0.38383	1.372725	0.046193
6011	ENSG00000137713	PPP2R1B	0.225976	5.377158	0.046194
6012	ENSG00000107798	LIPA	-0.26724	7.182757	0.046236
6013	ENSG00000224195		-0.40412	-0.32011	0.046257
6014	ENSG00000267123		0.57764	-0.94265	0.046397
6015	ENSG00000048991	R3HDM1	0.173519	5.209237	0.046397
6016	ENSG00000106066	CPVL	-0.48353	7.623077	0.046397
6017	ENSG00000107954	NEURL1	-0.4029	2.981509	0.046487
6018	ENSG00000117054	ACADM	0.254509	5.093772	0.046523
6019	ENSG00000110958	PTGES3	0.186355	7.736764	0.046559
6020	ENSG00000126012	KDM5C	0.193639	6.846002	0.046652
6021	ENSG00000074803	SLC12A1	-1.04835	-2.6572	0.046652
6022	ENSG00000257226		-0.54957	-0.42522	0.046667
6023	ENSG00000237828	PA2G4P1	0.278335	-0.33441	0.046759
6024	ENSG00000271997		-0.44726	0.39782	0.046796
6025	ENSG00000187239	FNBP1	0.197712	7.958957	0.046817

6026	ENSG00000011258	MBTD1	-0.14333	5.844613	0.046834
6027	ENSG00000259954	IL21R-AS1	0.775327	-0.2096	0.046845
6028	ENSG00000116857	TMEM9	0.181932	3.781496	0.046845
6029	ENSG00000127947	PTPN12	-0.19294	7.138344	0.046851
6030	ENSG00000025293	PHF20	-0.23022	6.292699	0.046851
6031	ENSG00000180539	C9orf139	-0.65079	0.686905	0.046851
6032	ENSG00000118777	ABCG2	0.650405	-1.0379	0.046852
6033	ENSG00000143185	XCL2	0.487168	2.930382	0.046942
6034	ENSG00000142733	MAP3K6	-0.38492	1.969968	0.046992
6035	ENSG00000167525	PROCA1	-0.24258	1.850244	0.047013
6036	ENSG00000114631	PODXL2	-0.30674	0.833055	0.047013
6037	ENSG00000250138		-0.75754	0.937655	0.047083
6038	ENSG00000111752	PHC1	-0.25278	5.311597	0.04709
6039	ENSG00000206337	HCP5	0.36932	6.559999	0.047091
6040	ENSG00000130684	ZNF337	-0.27438	1.803081	0.047092
6041	ENSG00000155254	MARVELD1	-0.72539	1.90007	0.047162
6042	ENSG00000239223	RPL34P31	-0.23759	4.350457	0.047238
6043	ENSG00000270223		-0.40666	-0.29572	0.047264
6044	ENSG00000156787	TBC1D31	0.299722	3.999	0.047292
6045	ENSG00000091947	TMEM101	0.186934	4.008985	0.047292
6046	ENSG00000174903	RAB1B	0.223275	6.133232	0.047308
6047	ENSG00000171497	PPID	0.1817	4.56503	0.047323
6048	ENSG00000162522	KIAA1522	-0.37897	0.916257	0.047349
6049	ENSG00000225093	RPL3P7	-0.24818	5.269376	0.047376
6050	ENSG00000164124	TMEM144	-0.62823	0.859963	0.047376
6051	ENSG00000161835	GRASP	-0.52554	5.317394	0.047397
6052	ENSG00000226284	ARPC3P1	0.2018	5.853514	0.047402
6053	ENSG00000124614	RPS10	-0.22593	6.7838	0.047419
6054	ENSG00000184113	CLDN5	-0.59222	-0.05703	0.047436
6055	ENSG00000188986	NELFB	0.219043	5.131322	0.047436
6056	ENSG00000167895	TMC8	-0.24203	7.968102	0.047442
6057	ENSG00000231682		-0.55415	-0.62919	0.047482
6058	ENSG00000142347	MYO1F	0.23845	8.397544	0.047506
6059	ENSG00000133612	AGAP3	-0.25034	4.760892	0.047526
6060	ENSG00000184368	MAP7D2	-0.39973	-0.81841	0.047536
6061	ENSG00000131378	RFTN1	0.278223	6.582112	0.047665
6062	ENSG00000106665	CLIP2	-0.32535	3.404857	0.047702
6063	ENSG00000090273	NUDC	0.191937	5.162032	0.047824
6064	ENSG00000253431		0.348621	-0.43797	0.047825
6065	ENSG00000273183		-0.39866	-0.41084	0.047847
6066	ENSG00000099256	PRTFDC1	0.352434	0.279305	0.047979
6067	ENSG00000150967	ABCB9	0.348653	1.266392	0.04799
6068	ENSG00000172667	ZMAT3	-0.14053	5.72771	0.048026
6069	ENSG00000187037	GPR141	-0.3821	2.440082	0.048046

6070	ENSG00000245680	ZNF585B	-0.30923	3.331232	0.048062
6071	ENSG00000225873	LINC00694	-0.55569	-0.26319	0.048062
6072	ENSG00000054118	THRAP3	0.264022	7.229477	0.048092
6073	ENSG00000203497	PDCD4-AS1	-0.19395	2.566817	0.04815
6074	ENSG00000244157		0.285217	-0.09632	0.048202
6075	ENSG00000086730	LAT2	-0.29413	5.972563	0.048244
6076	ENSG00000124333	VAMP7	0.129853	5.579072	0.048271
6077	ENSG00000153015	CWC27	0.188752	4.586857	0.048368
6078	ENSG00000103415	HMOX2	0.188786	5.304932	0.048385
6079	ENSG00000198794	SCAMP5	-0.46187	2.01281	0.048418
6080	ENSG00000177932	ZNF354C	-0.20603	3.825748	0.048518
6081	ENSG00000182973	CNOT10	0.120097	5.101331	0.048575
6082	ENSG00000164754	RAD21	0.151894	8.296743	0.04863
6083	ENSG00000135709	KIAA0513	-0.34365	5.690849	0.04863
6084	ENSG00000150045	KLRF1	-0.4339	5.552037	0.048696
6085	ENSG00000103044	HAS3	-0.30562	1.344665	0.048714
6086	ENSG00000130812	ANGPTL6	0.276807	0.239982	0.048748
6087	ENSG00000133134	BEX2	-0.29256	3.240978	0.048748
6088	ENSG00000246582		-0.33254	0.497464	0.048748
6089	ENSG00000145623	OSMR	-0.47837	1.622992	0.048748
6090	ENSG00000249256	ATP5LP3	0.299291	1.824562	0.048756
6091	ENSG00000218018		0.432438	2.719143	0.048775
6092	ENSG00000224137		-0.44929	0.514081	0.048775
6093	ENSG00000100697	DICER1	-0.1905	7.451579	0.048905
6094	ENSG00000101856	PGRMC1	-0.27813	5.841322	0.048979
6095	ENSG00000176102	CSTF3	0.170218	4.19274	0.049018
6096	ENSG00000254332		0.375297	-0.55692	0.049027
6097	ENSG00000241837	ATP5O	0.172901	6.453142	0.049027
6098	ENSG00000225422	RBMS1P1	-0.25542	2.546554	0.049062
6099	ENSG00000198363	ASPH	-0.27347	4.294973	0.049218
6100	ENSG00000157429	ZNF19	-0.29399	0.351373	0.049218
6101	ENSG00000035403	VCL	-0.34939	6.760665	0.049218
6102	ENSG00000269951		-0.36521	1.118165	0.049287
6103	ENSG00000134755	DSC2	-0.81772	1.451242	0.049287
6104	ENSG00000253683		0.55431	-0.28234	0.049344
6105	ENSG00000259248	USP3-AS1	-0.33137	2.295963	0.049368
6106	ENSG00000068654	POLR1A	0.166619	4.685511	0.049414
6107	ENSG00000183431	SF3A3	0.099919	6.427298	0.049414
6108	ENSG00000103494	RPGRIP1L	-0.27316	1.535884	0.049414
6109	ENSG00000113845	TIMMDC1	0.225803	4.72127	0.049426
6110	ENSG00000204116	CHIC1	-0.23019	5.305125	0.049426
6111	ENSG00000115956	PLEK	0.27418	9.244912	0.049436
6112	ENSG00000136051	KIAA1033	-0.25218	7.346297	0.049457
6113	ENSG00000240616		-0.21554	5.888577	0.049516

6114	ENSG00000196092	PAX5	-0.48839	5.013052	0.04958
6115	ENSG00000155008	APOOL	0.236587	1.533238	0.049631
6116	ENSG00000205758	CRYZL1	-0.17134	4.373329	0.049651
6117	ENSG00000101255	TRIB3	0.32741	3.114834	0.049664
6118	ENSG00000242114	MTFP1	0.359058	2.556596	0.049737
6119	ENSG00000251633	GYG1P1	0.425639	-0.96311	0.049743
6120	ENSG00000134480	CCNH	0.381995	6.181865	0.049743
6121	ENSG00000174695	TMEM167A	-0.16868	7.237232	0.049743
6122	ENSG00000116127	ALMS1	-0.22443	5.66262	0.049743
6123	ENSG00000163704	PRRT3	-0.2811	0.780907	0.049745
6124	ENSG00000272463		0.576607	-0.39194	0.04975
6125	ENSG00000046653	GPM6B	-0.37265	1.716175	0.049758
6126	ENSG00000085185	BCORL1	-0.33764	2.424634	0.049931
6127	ENSG00000176170	SPHK1	0.472662	3.462735	0.049957
6128	ENSG00000023734	STRAP	0.212024	6.696216	0.049957
6129	ENSG00000242125	SNHG3	-0.22629	6.05559	0.049957
6130	ENSG00000100744	GSKIP	0.211275	4.489094	0.049988
6131	ENSG00000111716	LDHB	0.185006	8.798236	0.049994

# = Rank by adj.P.Val; EnsemblID = Ensembl Stable ID name of the transcript; HGNC = HUGO Gene Nomenclature Committee name of the gene; logFC = Log_2 fold change; AveExpr = average expression across all samples, in log2 counts per million reads; adj.P.Val = Benjamini-Hochberg false discovery rate adjusted p-value.
