# Supplementary material for: Gene dysregulation in acute HIV-1 infection – early transcriptomic analysis reveals the crucial biological functions affected
Source: Front Cell Infect Microbiol. 2023 Apr 3;13:1074847. doi: 10.3389/fcimb.2023.1074847 (PMC10106835; doi:10.3389/fcimb.2023.1074847)
Supplement: Supplementary Table 2 — Full list of differentially expressed genes that correlate with viral load in acute HIV cases. # = Rank by rho; EnsemblID = Ensembl Stable ID name of the transcript; HGNC = HUGO Gene Nomenclature Committee name of the gene; rho = Spearman correlation coefficient; adj.P.Val = false discovery rate adjusted p-value. [file DataSheet_2.pdf]

Supplementary Table 2: Full list of differentially expressed genes that correlate with viral load in acute HIV cases

#	EnsemblID	HGNC	rho	adj.P.Val
1	ENSG00000102678	FGF9	-0.77206	0.005716
2	ENSG00000163629	PTPN13	-0.73793	0.010658
3	ENSG00000163935	SFMBT1	-0.73005	0.010658
4	ENSG00000126947	ARMCX1	-0.70895	0.017514
5	ENSG00000158122	AAED1	-0.7064	0.017514
6	ENSG00000109099	PMP22	-0.7009	0.017529
7	ENSG00000151117	TMEM86A	-0.68936	0.023146
8	ENSG00000256223	ZNF10	-0.68071	0.023798
9	ENSG00000179456	ZBTB18	-0.6798	0.023798
10	ENSG00000138463	DIRC2	-0.67931	0.023798
11	ENSG00000151150	ANK3	-0.67488	0.024206
12	ENSG00000008282	SYPL1	-0.67241	0.024469
13	ENSG00000114861	FOXP1	-0.67094	0.024469
14	ENSG00000146072	TNFRSF21	-0.66034	0.026987
15	ENSG00000121879	PIK3CA	-0.65764	0.028206
16	ENSG00000143674	MAP3K21	-0.65336	0.028655
17	ENSG00000160683	CXCR5	-0.65123	0.029541
18	ENSG00000165494	PCF11	-0.64762	0.030407
19	ENSG00000110851	PRDM4	-0.6468	0.030407
20	ENSG00000196150	ZNF250	-0.644	0.030407
21	ENSG00000145495	MARCHF6	-0.64236	0.030407
22	ENSG00000196267	ZNF836	-0.63957	0.030407
23	ENSG00000157483	MYO1E	-0.63915	0.030407
24	ENSG00000168280	KIF5C	-0.63892	0.030407
25	ENSG00000183496	MEX3B	-0.63222	0.032468
26	ENSG00000162804	SNED1	-0.63102	0.032468
27	ENSG00000112486	CCR6	-0.63092	0.032468
28	ENSG00000133477	FAM83F	-0.63082	0.032468
29	ENSG00000165661	QSOX2	-0.63005	0.032507
30	ENSG00000197860	SGTB	-0.62118	0.038689
31	ENSG00000186310	NAP1L3	-0.6191	0.038689
32	ENSG00000197279	ZNF165	-0.61722	0.03954
33	ENSG00000089091	DZANK1	-0.6155	0.040784
34	ENSG00000147180	ZNF711	-0.61328	0.042218
35	ENSG00000177076	ACER2	-0.61184	0.042218
36	ENSG00000104341	LAPTM4B	-0.60722	0.044254
37	ENSG00000198932	GPRASP1	-0.6069	0.044254
38	ENSG00000182107	TMEM30B	-0.60672	0.044254
39	ENSG00000040199	PHLPP2	-0.60426	0.044297
40	ENSG00000073417	PDE8A	-0.60106	0.0452
41	ENSG00000270230	MTND6P22	-0.60032	0.0452

42	ENSG00000081665	ZNF506	-0.59983	0.04522
43	ENSG00000237440	ZNF737	-0.5986	0.045575
44	ENSG00000175984	DENND2C	-0.59376	0.047866
45	ENSG00000163785	RYK	-0.59212	0.048109
46	ENSG00000182809	CRIP2	-0.58963	0.048356
47	ENSG00000091129	NRCAM	-0.58959	0.048356
48	ENSG00000171368	TPPP	-0.58931	0.048356
49	ENSG00000100099	HPS4	-0.58857	0.048479
50	ENSG00000203867	RBM20	-0.58732	0.049066
51	ENSG00000104738	MCM4	0.587685	0.049066
52	ENSG00000146410	MTFR2	0.589061	0.048356
53	ENSG00000166803	KIAA0101	0.589163	0.048356
54	ENSG00000134901	KDELC1	0.589918	0.048356
55	ENSG00000136213	CHST12	0.591206	0.048356
56	ENSG00000122952	ZWINT	0.592118	0.048109
57	ENSG00000164611	PTTG1	0.59293	0.048109
58	ENSG00000093009	CDC45	0.593596	0.047866
59	ENSG00000198176	TFDP1	0.593596	0.047866
60	ENSG00000090889	KIF4A	0.594089	0.047866
61	ENSG00000166851	PLK1	0.595074	0.047866
62	ENSG00000156374	PCGF6	0.595221	0.047866
63	ENSG00000164087	POC1A	0.598004	0.045733
64	ENSG00000075218	GTSE1	0.599162	0.045454
65	ENSG00000089685	BIRC5	0.60032	0.0452
66	ENSG00000024526	DEPDC1	0.600715	0.0452
67	ENSG00000104147	OIP5	0.602099	0.0452
68	ENSG00000238062	SPATA3-AS1	0.602839	0.045137
69	ENSG00000154920	EME1	0.604044	0.044297
70	ENSG00000182054	IDH2	0.604433	0.044297
71	ENSG00000161888	SPC24	0.605471	0.044297
72	ENSG00000127564	PKMYT1	0.605964	0.044297
73	ENSG00000004468	CD38	0.606897	0.044254
74	ENSG00000097021	ACOT7	0.609681	0.043394
75	ENSG00000211630	IGKV1D-13	0.61055	0.043048
76	ENSG00000165480	SKA3	0.611898	0.042218
77	ENSG00000100479	POLE2	0.61222	0.042218
78	ENSG00000110063	DCPS	0.618625	0.038689
79	ENSG00000144354	CDCA7	0.618719	0.038689
80	ENSG00000160298	C21orf58	0.619687	0.038689
81	ENSG00000211639	IGLV4-60	0.620826	0.038689
82	ENSG00000158402	CDC25C	0.626649	0.034458
83	ENSG00000145386	CCNA2	0.627494	0.034316
84	ENSG00000175063	UBE2C	0.632512	0.032468
85	ENSG00000115163	CENPA	0.634225	0.032468

86	ENSG00000072571	HMMR	0.636778	0.031231
87	ENSG00000152253	SPC25	0.638492	0.030407
88	ENSG00000253497	IGKV1-13	0.640966	0.030407
89	ENSG00000102384	CENPI	0.641775	0.030407
90	ENSG00000101003	GINS1	0.64532	0.030407
91	ENSG00000064886	CHI3L2	0.649754	0.02985
92	ENSG00000171320	ESCO2	0.653932	0.028655
93	ENSG00000117399	CDC20	0.65558	0.028655
94	ENSG00000134057	CCNB1	0.660591	0.026987
95	ENSG00000163808	KIF15	0.662643	0.026987
96	ENSG00000157456	CCNB2	0.663875	0.026987
97	ENSG00000135451	TROAP	0.666092	0.026987
98	ENSG00000138180	CEP55	0.67734	0.023798
99	ENSG00000126787	DLGAP5	0.687523	0.023146
100	ENSG00000121211	MND1	0.704375	0.017514
101	ENSG00000117650	NEK2	0.730968	0.010658

# = Rank by rho; EnsemblID = Ensembl Stable ID name of the transcript; HGNC = HUGO Gene Nomenclature Committee name of the gene; rho = Spearman correlation coefficient; adj.P.Val = false discovery rate adjusted p-value.
