# Supplementary material for: Gene dysregulation in acute HIV-1 infection – early transcriptomic analysis reveals the crucial biological functions affected
Source: Front Cell Infect Microbiol. 2023 Apr 3;13:1074847. doi: 10.3389/fcimb.2023.1074847 (PMC10106835; doi:10.3389/fcimb.2023.1074847)
Supplement: Supplementary Table 3 — Effect of adjustment for CD4+ T cell count (%) on Top 50 DEG. Pos. = Position ranked by adj.P.Val; HGNC = HUGO Gene Nomenclature Committee name of the gene; logFC = Log2 fold change; AveExpr = average expression across all samples, in log2 counts per million reads; adj.P.Val = Benjamini- Hochberg false discovery rate adjusted p-value; no-adj pos. = comparative position in the previous table, without adjustment for CD4+ T cell count (%). Yellow = top 50 DEG concordant in both analyses; orange = top 50 DEG discordant between analyses (though all within top 100). [file DataSheet_3.pdf]

Supplementary Table 3: Effect of adjustment for CD4+ T cell count (%) on Top 50 DEG

WITHOUT ADJUSTMENT FOR CD4+ COUNT

Pos.	HGNC	logFC	AveExpr	adj.P.Val
1	TMEM155	3.27	1.46	4.46259E-35
2	CDCA7	2.47	3.92	9.42442E-34
3	NUSAP1	1.98	4.08	1.07822E-26
4	KIAA0101	2.78	2.65	3.19793E-26
5	TYMS	2.47	2.94	3.19793E-26
6	NCAPG	2.33	2.42	3.19793E-26
7	CDC45	2.84	1.21	1.12004E-25
8	CD8BP	1.57	4.15	1.12004E-25
9	CCNA2	2.32	3.17	3.87894E-25
10	DTL	2.89	2.32	6.17847E-25
11	MELK	2.49	1.52	6.17847E-25
12	KIF11	2.17	3.63	6.17847E-25
13	CD8B	1.50	6.85	3.76833E-24
14	BUB1B	2.21	1.90	6.30044E-24
15	MYL6B	1.56	2.43	6.30044E-24
16	MKI67	2.58	4.84	6.65383E-24
17	ANLN	2.35	1.53	1.37422E-23
18	CCL5	1.39	9.83	1.53502E-23
19	RRM2	2.65	4.21	1.781E-23
20	PBK	2.76	0.55	1.99406E-23
21	UHRF1	1.92	2.58	2.07217E-23
22	CLSPN	2.30	2.04	2.77335E-23
23	CDC6	2.41	2.14	4.18561E-23
24	MCM10	2.82	0.88	5.57103E-23
25	CDCA5	2.49	2.11	1.00003E-22
26	TOP2A	2.11	4.70	1.00003E-22
27	APOBEC3H	1.67	2.12	1.00003E-22
28	FABP5	1.64	3.84	1.00003E-22
29	KIF15	2.38	1.76	1.24638E-22
30	ASPM	2.26	2.80	1.57062E-22
31	BRIP1	1.40	2.48	1.96808E-22
32	SKA3	2.78	0.30	2.38623E-22
33	GINS2	2.34	1.67	4.245E-22
34	KIF7	2.73	-0.83	4.96291E-22
35	E2F8	2.68	0.49	4.96291E-22
36	EXO1	2.34	1.19	4.96291E-22
37	KIF2C	2.16	1.84	4.96291E-22
38	POLQ	2.10	1.56	4.96291E-22
39	MCM4	1.71	4.82	4.96291E-22
40	TPX2	2.33	2.67	6.44989E-22
41	SLC27A2	2.14	1.83	8.64879E-22
42	CDC25A	2.63	0.73	9.18752E-22
43	KIFC1	2.12	2.14	1.22804E-21
44	ESCO2	2.40	0.82	1.62804E-21
45	E2F7	2.30	1.31	1.73031E-21
46	CENPM	1.95	1.52	3.65861E-21
47	NEIL3	2.57	-0.34	4.13626E-21
48	CD8A	1.59	8.66	4.22226E-21
49	MIR4435-1HG	1.14	4.94	4.22226E-21
50	RAD54L	1.81	0.60	5.04336E-21
52	CDKN3	1.98	1.46	6.33662E-21
58	FANCI	1.17	4.63	1.64818E-20
59	STMN1	1.51	5.91	1.89655E-20
61	HMGB2	1.17	7.61	2.17405E-20
79	CENPU	1.59	3.22	3.37666E-19
84	EZH2	1.23	5.14	8.61787E-19

WITH ADJUSTMENT FOR CD4+ COUNT

Pos. (no-adj pos.)	HGNC	logFC	AveExpr	adj.P.Val
1 (1)	TMEM155	3.10	1.44	3.77986E-25
2 (2)	CDCA7	2.27	3.91	5.61263E-24
3 (13)	CD8B	1.46	6.85	1.59314E-18
4 (8)	CD8BP	1.48	4.14	2.57486E-18
5 (3)	NUSAP1	1.73	4.08	4.18957E-18
6 (4)	KIAA0101	2.52	2.64	8.80319E-18
7 (5)	TYMS	2.19	2.93	2.30858E-17
8 (6)	NCAPG	2.04	2.42	6.01457E-17
9 (18)	CCL5	1.28	9.83	6.06466E-17
10 (7)	CDC45	2.47	1.20	1.65346E-16
11 (10)	DTL	2.58	2.31	1.83553E-16
12 (12)	KIF11	1.89	3.62	2.96901E-16
13 (15)	MYL6B	1.41	2.43	3.71845E-16
14 (9)	CCNA2	2.01	3.17	3.78736E-16
15 (14)	BUB1B	2.00	1.90	6.16117E-16
16 (26)	TOP2A	1.94	4.69	6.16117E-16
17 (16)	MKI67	2.25	4.83	7.07135E-16
18 (11)	MELK	2.13	1.51	9.07092E-16
19 (48)	CD8A	1.50	8.66	1.13983E-15
20 (17)	ANLN	2.08	1.52	1.30187E-15
21 (31)	BRIP1	1.27	2.48	1.30187E-15
22 (19)	RRM2	2.30	4.20	2.45967E-15
23 (22)	CLSPN	2.03	2.03	3.01827E-15
24 (21)	UHRF1	1.68	2.58	4.19565E-15
25 (59)	STMN1	1.40	5.90	4.19565E-15
26 (39)	MCM4	1.52	4.81	4.22719E-15
27 (23)	CDC6	2.10	2.13	5.18483E-15
28 (24)	MCM10	2.49	0.87	9.72422E-15
29 (29)	KIF15	2.09	1.75	9.72422E-15
30 (30)	ASPM	1.99	2.79	9.72422E-15
31 (20)	PBK	2.39	0.54	1.14544E-14
32 (33)	GINS2	2.10	1.66	1.14544E-14
33 (46)	CENPM	1.81	1.51	1.66859E-14
34 (36)	EXO1	2.09	1.18	1.71812E-14
35 (38)	POLQ	1.86	1.55	1.71812E-14
36 (61)	HMGB2	1.05	7.59	1.77961E-14
37 (37)	KIF2C	1.93	1.83	1.78345E-14
38 (42)	CDC25A	2.34	0.72	1.8918E-14
39 (25)	CDCA5	2.13	2.10	2.94507E-14
40 (43)	KIFC1	1.89	2.13	3.39277E-14
41 (27)	APOBEC3H	1.38	2.11	3.96751E-14
42 (45)	E2F7	2.03	1.30	4.18547E-14
43 (28)	FABP5	1.38	3.83	4.40282E-14
44 (52)	CDKN3	1.75	1.44	7.57801E-14
45 (84)	EZH2	1.15	5.13	7.65555E-14
46 (34)	KIF7	2.44	-0.83	1.10919E-13
47 (35)	E2F8	2.28	0.48	1.10919E-13
49 (50)	RAD54L	1.63	0.60	1.26104E-13
49 (58)	FANCI	1.01	4.63	1.64369E-13
50 (79)	CENPU	1.44	3.21	2.06533E-13
52 (32)	SKA3	2.30	0.29	2.26687E-13
58 (47)	NEIL3	2.28	-0.35	2.92486E-13
59 (40)	TPX2	1.93	2.66	3.40802E-13
61 (44)	ESCO2	2.04	0.81	3.92518E-13
66 (41)	SLC27A2	1.75	1.83	7.56526E-13
74 (49)	MIR4435-1HG	0.90	4.94	1.7109E-12

Footnotes: Pos. = Position ranked by adj.P.Val; HGNC = HUGO Gene Nomenclature Committee name of the gene; logFC = Log2 fold change; AveExpr = average expression across all samples, in log2 counts per million reads; adj.P.Val = Benjamini- Hochberg false discovery rate adjusted p-value; no-adj pos. = comparative position in the previous table, without adjustment for CD4+ T cell count (%). Yellow = top 50 DEG concordant in both analyses; orange = top 50 DEG discordant between analyses (all featured within top 100).
