# Supplementary material for: Gene dysregulation in acute HIV-1 infection – early transcriptomic analysis reveals the crucial biological functions affected
Source: Front Cell Infect Microbiol. 2023 Apr 3;13:1074847. doi: 10.3389/fcimb.2023.1074847 (PMC10106835; doi:10.3389/fcimb.2023.1074847)
Supplement: Supplementary Table 4 — Full list of differentially expressed gene sets – acute HIV cases versus HIV negative controls as determined by gene set enrichment analysis (GSEA). (A) Overexpressed gene sets. (B) Underexpressed gene sets. # = Rank by NES; Name = Gene Ontology name of the geneset; Size = the number of genes in the geneset; NES = Normalized Enrichment Score; NOM p-val = nominal P value (statistical significance of the observed ES relative to null distribution); FDR q-val = False Discovery Rate Q value (the P value has been adjusted for FDR). [file DataSheet_4.pdf]

Supplementary Table 4: Full list of differentially expressed gene sets – acute HIV cases versus HIV negative controls as determined by gene set enrichment analysis (GSEA). A) Overexpressed gene sets. B) Underexpressed gene sets.

A)

#	NAME	SIZE	NES	NOM p-val	FDR q-val
1	GO_CHROMOSOME_SEGREGATION	155	3.12792	<0.001	<6.60E-06
2	GO_MITOTIC_SISTER_CHROMATID_SEGREGATION	82	3.109688	<0.001	<6.60E-06
3	GO_SISTER_CHROMATID_SEGREGATION	96	3.082613	<0.001	<6.60E-06
4	GO_NUCLEAR_CHROMOSOME_SEGREGATION	125	3.037414	<0.001	<6.60E-06
5	GO_MITOTIC_NUCLEAR_DIVISION	133	3.015512	<0.001	<6.60E-06
6	GO_CELL_CYCLE_CHECKPOINT	91	3.001413	<0.001	<6.60E-06
7	GO_MITOTIC_CELL_CYCLE_CHECKPOINT	69	2.972932	<0.001	<6.60E-06
8	GO_CELL_CYCLE_G1_S_PHASE_TRANSITION	108	2.972604	<0.001	<6.60E-06
9	GO_ORGANELLE_FISSION	193	2.970232	<0.001	<6.60E-06
10	GO_DNA_REPLICATION	138	2.955059	<0.001	<6.60E-06
11	GO_MITOTIC_CELL_CYCLE	416	2.922412	<0.001	<6.60E-06
12	GO_CELL_DIVISION	243	2.898836	<0.001	<6.60E-06
13	GO_MEIOTIC_CELL_CYCLE_PROCESS	76	2.879818	<0.001	<6.60E-06
14	GO_DNA_DEPENDENT_DNA_REPLICATION	87	2.87035	<0.001	<6.60E-06
15	GO_MICROTUBULE_CYTOSKELETON_ORGANIZATION_INVOLVED_IN_MITOSIS	66	2.869477	<0.001	<6.60E-06
16	GO_MEIOTIC_CELL_CYCLE	94	2.868924	<0.001	<6.60E-06
17	GO_CELL_CYCLE_PHASE_TRANSITION	252	2.858066	<0.001	<6.60E-06
18	GO_POSITIVE_REGULATION_OF_CELL_CYCLE_PROCESS	93	2.855885	<0.001	<6.60E-06
19	GO_REGULATION_OF_CHROMOSOME_SEGREGATION	55	2.851661	<0.001	<6.60E-06
20	GO_DNA_INTEGRITY_CHECKPOINT	66	2.844946	<0.001	<6.60E-06
21	GO_REGULATION_OF_NUCLEAR_DIVISION	78	2.833164	<0.001	<6.60E-06
22	GO_CHROMOSOME_SEPARATION	43	2.828484	<0.001	<6.60E-06
23	GO_DNA_CONFORMATION_CHANGE	128	2.823176	<0.001	<6.60E-06
24	GO_SPINDLE_ORGANIZATION	80	2.80245	<0.001	<6.60E-06
25	GO_REGULATION_OF_SISTER_CHROMATID_SEGREGATION	41	2.79333	<0.001	<6.60E-06
26	GO_MITOTIC_SPINDLE_ORGANIZATION	56	2.778918	<0.001	<6.60E-06
27	GO_METAPHASE_ANAPHASE_TRANSITION_OF_CELL_CYCLE	31	2.772703	<0.001	<6.60E-06
28	GO_REGULATION_OF_CHROMOSOME_SEPARATION	33	2.762958	<0.001	<6.60E-06
29	GO_CELL_CYCLE_DNA_REPLICATION	44	2.742908	<0.001	<6.60E-06
30	GO_NEGATIVE_REGULATION_OF_CELL_CYCLE_PROCESS	136	2.712687	<0.001	<6.60E-06
31	GO_POSITIVE_REGULATION_OF_CELL_CYCLE	116	2.698439	<0.001	<6.60E-06
32	GO_REGULATION_OF_DNA_REPLICATION	45	2.693861	<0.001	<6.60E-06
33	GO_NEGATIVE_REGULATION_OF_CELL_CYCLE_PHASE_TRANSITION	107	2.689305	<0.001	<6.60E-06
34	GO_REGULATION_OF_CELL_CYCLE_PHASE_TRANSITION	180	2.685047	<0.001	<6.60E-06
35	GO_MITOTIC_DNA_INTEGRITY_CHECKPOINT	42	2.681066	<0.001	<6.60E-06
36	GO_CENTROMERE_COMPLEX_ASSEMBLY	29	2.675187	<0.001	<6.60E-06

37	GO_NEGATIVE_REGULATION_OF_MITOTIC_CELL_CYCLE	133	2.670119	<0.001	<6.60E-06
38	GO_REGULATION_OF_TRANSCRIPTION_INVOLVED_IN_G1_S_TRANSITION_OF_MITOTIC_CELL_CYCLE	19	2.668397	<0.001	<6.60E-06
39	GO_DNA_PACKAGING	82	2.66796	<0.001	<6.60E-06
40	GO_DNA_GEOMETRIC_CHANGE	50	2.66624	<0.001	<6.60E-06
41	GO_NEGATIVE_REGULATION_OF_NUCLEAR_DIVISION	29	2.663813	<0.001	<6.60E-06
42	GO_PROTEIN_DNA_COMPLEX_SUBUNIT_ORGANIZATION	98	2.651023	<0.001	<6.60E-06
43	GO_NEGATIVE_REGULATION_OF_CHROMOSOME_SEGREGATION	24	2.643225	<0.001	<6.60E-06
44	GO_DNA_REPLICATION_INITIATION	27	2.643191	<0.001	<6.60E-06
45	GO_COMPLEMENT_ACTIVATION	97	2.637208	<0.001	<6.60E-06
46	GO_HUMORAL_IMMUNE_RESPONSE_MEDIATED_BY_CIRCULATING_IMMUNOGLOBULIN	95	2.632879	<0.001	<6.60E-06
47	GO_MEIOTIC_CHROMOSOME_SEGREGATION	41	2.6313	<0.001	<6.60E-06
48	GO_MEIOSIS_I_CELL_CYCLE_PROCESS	53	2.629026	<0.001	<6.60E-06
49	GO_SIGNAL_TRANSDUCTION_INVOLVED_IN_CELL_CYCLE_CHECKPOINT	29	2.628416	<0.001	<6.60E-06
50	GO_REGULATION_OF_CELL_CYCLE_PROCESS	277	2.61734	<0.001	<6.60E-06
51	GO_REGULATION_OF_DNA_DEPENDENT_DNA_REPLICATION	28	2.604467	<0.001	<6.60E-06
52	GO_METAPHASE_PLATE_CONGRESSION	30	2.60269	<0.001	<6.60E-06
53	GO_CELL_CYCLE_G2_M_PHASE_TRANSITION	126	2.599672	<0.001	<6.60E-06
54	GO_REGULATION_OF_MITOTIC_CELL_CYCLE	246	2.596684	<0.001	<6.60E-06
55	GO_KINETOCHORE_ORGANIZATION	17	2.585083	<0.001	<6.60E-06
56	GO_NEGATIVE_REGULATION_OF_CELL_CYCLE	231	2.583847	<0.001	<6.60E-06
57	GO_CHROMOSOME_LOCALIZATION	35	2.582653	<0.001	<6.60E-06
58	GO_REGULATION_OF_CYCLIN_DEPENDENT_PROTEIN_KINASE_ACTIVITY	42	2.572751	<0.001	<6.60E-06
59	GO_POSITIVE_REGULATION_OF_CELL_CYCLE_PHASE_TRANSITION	39	2.571314	<0.001	<6.60E-06
60	GO_REGULATION_OF_CELL_CYCLE_G2_M_PHASE_TRANSITION	103	2.570251	<0.001	<6.60E-06
61	GO_POSITIVE_REGULATION_OF_MITOTIC_CELL_CYCLE	56	2.567554	<0.001	<6.60E-06
62	GO_CHROMOSOME_ORGANIZATION	427	2.561677	<0.001	<6.60E-06
63	GO_DNA_REPAIR	233	2.556828	<0.001	<6.60E-06
64	GO_NEGATIVE_REGULATION_OF_CHROMOSOME_ORGANIZATION	51	2.554247	<0.001	<6.60E-06
65	GO_POSITIVE_REGULATION_OF_CELL_CYCLE_ARREST	23	2.553874	<0.001	<6.60E-06
66	GO_NEGATIVE_REGULATION_OF_METAPHASE_ANAPHASE_TRANSITION_OF_CELL_CYCLE	21	2.54859	<0.001	<6.60E-06
67	GO_B_CELL_MEDIATED_IMMUNITY	119	2.543857	<0.001	<6.60E-06
68	GO_REGULATION_OF_CELL_CYCLE_ARREST	29	2.540933	<0.001	<6.60E-06
69	GO_DOUBLE_STRAND_BREAK_REPAIR	108	2.538638	<0.001	<6.60E-06
70	GO_MICROTUBULE_CYTOSKELETON_ORGANIZATION	199	2.535935	<0.001	<6.60E-06
71	GO_SPINDLE_ASSEMBLY	50	2.525703	<0.001	<6.60E-06
72	GO_CELLULAR_RESPONSE_TO_DNA_DAMAGE_STIMULUS	323	2.520593	<0.001	<6.60E-06
73	GO_IMMUNOGLOBULIN_PRODUCTION	105	2.512441	<0.001	<6.60E-06
74	GO_DNA_METABOLIC_PROCESS	362	2.507067	<0.001	<6.60E-06
75	GO_DNA_STRAND_ELONGATION	16	2.506893	<0.001	<6.60E-06
76	GO_CHROMATIN_REMODELING_AT_CENTROMERE	25	2.506599	<0.001	<6.60E-06
77	GO_CYTOKINESIS	55	2.505293	<0.001	<6.60E-06
78	GO_NUCLEOSOME_ORGANIZATION	52	2.505126	<0.001	<6.60E-06
79	GO_NUCLEOSOME_ASSEMBLY	43	2.504551	<0.001	<6.60E-06
80	GO_MITOTIC_METAPHASE_PLATE_CONGRESSION	23	2.502864	<0.001	<6.60E-06

81	GO_DNA_REPLICATION_INDEPENDENT_NUCLEOSOME_ORGANIZATION	29	2.499239	<0.001	<6.60E-06
82	GO_REGULATION_OF_CELL_CYCLE_G1_S_PHASE_TRANSITION	57	2.494866	<0.001	<6.60E-06
83	GO_REGULATION_OF_HUMORAL_IMMUNE_RESPONSE	73	2.485472	<0.001	<6.60E-06
84	GO_MITOTIC_SPINDLE_ASSEMBLY	30	2.48522	<0.001	<6.60E-06
85	GO_DNA_STRAND_ELONGATION_INVOLVED_IN_DNA_REPLICATION	15	2.485081	<0.001	<6.60E-06
86	GO_DNA_RECOMBINATION	124	2.483826	<0.001	<6.60E-06
87	GO_HUMORAL_IMMUNE_RESPONSE	129	2.483382	<0.001	<6.60E-06
88	GO_RECOMBINATIONAL_REPAIR	67	2.472922	<0.001	<6.60E-06
89	GO_MICROTUBULE_ORGANIZING_CENTER_ORGANIZATION	51	2.469607	<0.001	<6.60E-06
90	GO_REGULATION_OF_MICROTUBULE_CYTOSKELETON_ORGANIZATION	62	2.468602	<0.001	<6.60E-06
91	GO_SIGNAL_TRANSDUCTION_BY_P53_CLASS_MEDIATOR	99	2.468332	<0.001	<6.60E-06
92	GO_REGULATION_OF_CELL_CYCLE	423	2.464054	<0.001	<6.60E-06
93	GO_HISTONE_EXCHANGE	28	2.460003	<0.001	<6.60E-06
94	GO_FC_RECEPTOR_MEDIATED_STIMULATORY_SIGNALING_PATHWAY	80	2.457058	<0.001	<6.60E-06
95	GO_SISTER_CHROMATID_COHESION	31	2.453386	<0.001	<6.60E-06
96	GO_SIGNAL_TRANSDUCTION_IN_RESPONSE_TO_DNA_DAMAGE	51	2.452599	<0.001	<6.60E-06
97	GO_PHAGOCYTOSIS_RECOGNITION	49	2.450502	<0.001	<6.60E-06
98	GO_ATTACHMENT_OF_SPINDLE_MICROTUBULES_TO_KINETOCHORE	19	2.449955	<0.001	<6.60E-06
99	GO_LYMPHOCYTE_MEDIATED_IMMUNITY	167	2.449774	<0.001	<6.60E-06
100	GO_CHROMATIN_ASSEMBLY_OR_DISASSEMBLY	67	2.44137	<0.001	<6.60E-06
101	GO_REGULATION_OF_CHROMOSOME_ORGANIZATION	122	2.426902	<0.001	<6.60E-06
102	GO_FC_EPSILON_RECEPTOR_SIGNALING_PATHWAY	99	2.419287	<0.001	<6.60E-06
103	GO_MITOTIC_G1_S_TRANSITION_CHECKPOINT	25	2.416626	<0.001	<6.60E-06
104	GO_ANAPHASE_PROMOTING_COMPLEX_DEPENDENT_CATABOLIC_PROCESS	50	2.414882	<0.001	<6.60E-06
105	GO_DNA_DAMAGE_RESPONSE_SIGNAL_TRANSDUCTION_BY_P53_CLASS_MEDIATOR	41	2.414501	<0.001	<6.60E-06
106	GO_NEGATIVE_REGULATION_OF_CELL_CYCLE_G2_M_PHASE_TRANSITION	52	2.413469	<0.001	<6.60E-06
107	GO_PRODUCTION_OF_MOLECULAR_MEDIATOR_OF_IMMUNE_RESPONSE	127	2.411274	<0.001	<6.60E-06
108	GO_REGULATION_OF_MICROTUBULE_BASED_PROCESS	68	2.410039	<0.001	<6.60E-06
109	GO_CYTOSKELETON_DEPENDENT_CYTOKINESIS	36	2.399931	<0.001	<6.60E-06
110	GO_POSITIVE_REGULATION_OF_B_CELL_ACTIVATION	72	2.395207	<0.001	<6.60E-06
111	GO_MITOTIC_CYTOKINESIS	31	2.392329	<0.001	<6.60E-06
112	GO_ATP_DEPENDENT_CHROMATIN_REMODELING	34	2.390495	<0.001	<6.60E-06
113	GO_ADAPTIVE_IMMUNE_RESPONSE	283	2.371826	<0.001	<6.60E-06
114	GO_MICROTUBULE_BASED_PROCESS	262	2.36752	<0.001	<6.60E-06
115	GO_MICROTUBULE_POLYMERIZATION_OR_DEPOLYMERIZATION	34	2.364617	<0.001	<6.60E-06
116	GO_CHROMATIN_REMODELING	70	2.363085	<0.001	<6.60E-06
117	GO_POSITIVE_REGULATION_OF_MITOTIC_NUCLEAR_DIVISION	21	2.358835	<0.001	<6.60E-06
118	GO_ADAPTIVE_IMMUNE_RESPONSE_BASED_ON_SOMATIC_RECOMBINATION_OF_IMMUNE_RECEPTORS_BUILT_FROM_IMMUNOGLOBULIN_SUPERFAMILY_DOMAINS	168	2.357492	<0.001	<6.60E-06
119	GO_NEGATIVE_REGULATION_OF_CELL_CYCLE_G1_S_PHASE_TRANSITION	41	2.348305	<0.001	<6.60E-06
120	GO_CHROMOSOME_CONDENSATION	20	2.345261	<0.001	<6.60E-06
121	GO_SPINDLE_LOCALIZATION	18	2.337231	<0.001	<6.60E-06
122	GO_REGULATION_OF_CYTOKINESIS	30	2.335655	<0.001	<6.60E-06

123	GO_PROTEIN_LOCALIZATION_TO_CHROMOSOME_CENTROMERIC_REGION	17	2.327841	<0.001	<6.60E-06
124	GO_DEFENSE_RESPONSE_TO_BACTERIUM	84	2.32302	<0.001	<6.60E-06
125	GO_CELLULAR_RESPONSE_TO_RADIATION	43	2.30286	<0.001	<6.60E-06
126	GO_CHROMOSOME_ORGANIZATION_INVOLVED_IN_MEIOTIC_CELL_CYCLE	34	2.293063	<0.001	<6.60E-06
127	GO_REGULATION_OF_B_CELL_ACTIVATION	86	2.285823	<0.001	<6.60E-06
128	GO_REGULATION_OF_CENTROSOME_CYCLE	25	2.28284	<0.001	<6.60E-06
129	GO_PHAGOCYTOSIS	149	2.27652	<0.001	6.61E-06
130	GO_PROTEIN_LOCALIZATION_TO_CHROMOSOME	48	2.276217	<0.001	6.56E-06
131	GO_HOMOLOGOUS_RECOMBINATION	23	2.272102	<0.001	6.51E-06
132	GO_FC_RECEPTOR_SIGNALING_PATHWAY	124	2.271907	<0.001	6.46E-06
133	GO_TELOMERE_ORGANIZATION	70	2.263712	<0.001	6.41E-06
134	GO_G2_DNA_DAMAGE_CHECKPOINT	15	2.256826	<0.001	6.37E-06
135	GO_REGULATION_OF_CELL_DIVISION	55	2.246473	<0.001	6.32E-06
136	GO_ANTIGEN_PROCESSING_AND_PRESENTATION_OF_PEPTIDE_OR_POLYSACCHARIDE_ANTIGEN_VIA_MHC_CLASS_II	28	2.235628	<0.001	1.26E-05
137	GO_CELLULAR_RESPONSE_TO_UV	24	2.224398	<0.001	1.25E-05
138	GO_RESPONSE_TO_UV	40	2.212977	<0.001	1.85E-05
139	GO_POSITIVE_REGULATION_OF_DNA_REPLICATION	15	2.207582	<0.001	3.07E-05
140	GO_HISTONE_PHOSPHORYLATION	16	2.198512	<0.001	4.27E-05
141	GO_B_CELL_RECEPTOR_SIGNALING_PATHWAY	74	2.189282	<0.001	6.64E-05
142	GO_CELLULAR_RESPONSE_TO_LIGHT_STIMULUS	28	2.18764	<0.001	7.18E-05
143	GO_DNA_BIOSYNTHETIC_PROCESS	76	2.182219	<0.001	8.32E-05
144	GO_POSITIVE_REGULATION_OF_NUCLEAR_DIVISION	25	2.182198	<0.001	8.26E-05
145	GO_CHROMATIN_ORGANIZATION_INVOLVED_IN_REGULATION_OF_TRANSCRIPTION	30	2.180725	<0.001	9.37E-05
146	GO_ANIMAL_ORGAN_REGENERATION	22	2.180713	<0.001	9.30E-05
147	GO_CELL_CYCLE_ARREST	73	2.167949	<0.001	1.39E-04
148	GO_REGULATION_OF_MEIOTIC_CELL_CYCLE	19	2.166404	<0.001	1.49E-04
149	GO_NEGATIVE_REGULATION_OF_ORGANELLE_ORGANIZATION	119	2.157564	<0.001	1.71E-04
150	GO_FEMALE_MEIOTIC_NUCLEAR_DIVISION	16	2.154675	<0.001	1.76E-04
151	GO_HOMOLOGOUS_CHROMOSOME_SEGREGATION	26	2.139124	<0.001	2.36E-04
152	GO_REGULATION_OF_SIGNAL_TRANSDUCTION_BY_P53_CLASS_MEDIATOR	69	2.136436	<0.001	2.46E-04
153	GO_MEMBRANE_INVAGINATION	64	2.132841	<0.001	2.50E-04
154	GO_OXIDATIVE_PHOSPHORYLATION	78	2.132296	<0.001	2.54E-04
155	GO_ANTIGEN_PROCESSING_AND_PRESENTATION	76	2.121087	<0.001	3.07E-04
156	GO_REGULATION_OF_DNA_METABOLIC_PROCESS	119	2.113213	<0.001	3.54E-04
157	GO_G0_TO_G1_TRANSITION	19	2.110727	<0.001	3.73E-04
158	GO_POSITIVE_REGULATION_OF_CHROMOSOME_SEGREGATION	17	2.109074	<0.001	3.92E-04
159	GO_TELOMERE_MAINTENANCE_VIA_SEMI_CONSERVATIVE_REPLICATION	20	2.107104	<0.001	4.11E-04
160	GO_RETROGRADE_VESICLE_MEDIATED_TRANSPORT_GOLGI_TO_ENDOPLASMIC_RETICULUM	25	2.10695	<0.001	4.09E-04
161	GO_NEGATIVE_REGULATION_OF_GENE_EXPRESSION_EPIGENETIC	23	2.095096	<0.001	4.69E-04
162	GO_DNA_SYNTHESIS_INVOLVED_IN_DNA_REPAIR	29	2.094288	<0.001	4.82E-04
163	GO_ANTIGEN_RECEPTOR_MEDIATED_SIGNALING_PATHWAY	157	2.091057	<0.001	5.05E-04
164	GO_REGULATION_OF_GENE_EXPRESSION_EPIGENETIC	42	2.089978	<0.001	5.18E-04

165	GO_FOLIC_ACID_CONTAINING_COMPOUND_METABOLIC_PROCESS	16	2.087601	<0.001	5.56E-04
166	GO_REGULATION_OF_CELLULAR_AMINO_ACID_METABOLIC_PROCESS	33	2.085167	<0.001	5.63E-04
167	GO_FEMALE_GAMETE_GENERATION	41	2.084418	0.0012	5.60E-04
168	GO_ANTIGEN_PROCESSING_AND_PRESENTATION_OF_PEPTIDE_ANTIGEN	67	2.084132	<0.001	5.56E-04
169	GO_ACTIVATION_OF_IMMUNE_RESPONSE	244	2.080836	<0.001	5.73E-04
170	GO_POSITIVE_REGULATION_OF_LYMPHOCYTE_ACTIVATION	138	2.080518	<0.001	5.74E-04
171	GO_MICROTUBULE_DEPOLYMERIZATION	17	2.078105	<0.001	5.96E-04
172	GO_TRANSLESION_SYNTHESIS	27	2.073701	<0.001	6.62E-04
173	GO_POSITIVE_REGULATION_OF_DNA_METABOLIC_PROCESS	71	2.066606	<0.001	7.22E-04
174	GO_NEGATIVE_REGULATION_OF_VIRAL_GENOME_REPLICATION	31	2.0642	<0.001	7.52E-04
175	GO_CHROMATIN_ORGANIZATION	228	2.061901	<0.001	7.72E-04
176	GO_ATP_SYNTHESIS_COUPLED_ELECTRON_TRANSPORT	60	2.060155	<0.001	7.92E-04
177	GO_INTERSTRAND_CROSS_LINK_REPAIR	30	2.054076	0.001253	8.69E-04
178	GO_IMMUNE_RESPONSE_REGULATING_SIGNALING_PATHWAY	226	2.051117	<0.001	9.02E-04
179	GO_RESPONSE_TO_RADIATION	118	2.04851	<0.001	9.40E-04
180	GO_RESPONSE_TO_IONIZING_RADIATION	47	2.048021	<0.001	9.53E-04
181	GO_B_CELL_ACTIVATION	128	2.047597	<0.001	9.53E-04
182	GO_POSTREPLICATION_REPAIR	33	2.046234	0.00123	9.84E-04
183	GO_POSITIVE_REGULATION_OF_IMMUNE_RESPONSE	302	2.045372	<0.001	9.88E-04
184	GO_TRANSLATIONAL_TERMINATION	66	2.043309	<0.001	0.001006
185	GO_CENTROSOME_DUPLICATION	26	2.037362	<0.001	0.001106
186	GO_REGULATION_OF_CELLULAR_AMINE_METABOLIC_PROCESS	36	2.036527	<0.001	0.001128
187	GO_RESPONSE_TO_TYPE_I_INTERFERON	41	2.03574	<0.001	0.00114
188	GO_PTERIDINE_CONTAINING_COMPOUND_METABOLIC_PROCESS	17	2.034862	<0.001	0.001161
189	GO_REGULATION_OF_DNA_BINDING	38	2.028633	<0.001	0.001281
190	GO_CELLULAR_PROCESS_INVOLVED_IN_REPRODUCTION_IN_MULTICELLULAR_ORGANISM	85	2.027628	<0.001	0.001292
191	GO_HEPATICOBILIARY_SYSTEM_DEVELOPMENT	39	2.016879	<0.001	0.001511
192	GO_REGULATION_OF_DNA_TEMPLATED_TRANSCRIPTION_IN_RESPONSE_TO_STRESS	43	2.010455	0.001186	0.001601
193	GO_MITOCHONDRIAL_TRANSLATIONAL_TERMINATION	61	2.007317	<0.001	0.001663
194	GO_CYTOKINETIC_PROCESS	15	2.005188	<0.001	0.001725
195	GO_REGULATION_OF_RESPONSE_TO_DNA_DAMAGE_STIMULUS	81	1.998024	<0.001	0.001934
196	GO_CHROMATIN_SILENCING	16	1.9946	<0.001	0.002032
197	GO_POSITIVE_REGULATION_OF_CYTOKINESIS	15	1.994332	<0.001	0.002026
198	GO_PROTEIN_MODIFICATION_BY_SMALL_PROTEIN_REMOVAL	100	1.988368	<0.001	0.002234
199	GO_LEUKOCYTE_MEDIATED_IMMUNITY	285	1.988142	<0.001	0.002227
200	GO_INNATE_IMMUNE_RESPONSE	311	1.987489	<0.001	0.002246
201	GO_POSITIVE_REGULATION_OF_CELL_CYCLE_G2_M_PHASE_TRANSITION	17	1.986144	0.003995	0.002285
202	GO_REGULATION_OF_DNA_BIOSYNTHETIC_PROCESS	37	1.985959	0.001233	0.002278
203	GO_REGULATION_OF_MICROTUBULE_POLYMERIZATION_OR_DEPOLYMERIZATION	23	1.985336	<0.001	0.002275
204	GO_NUCLEOSIDE_METABOLIC_PROCESS	39	1.983842	0.001233	0.002318
205	GO_REGULATION_OF_TRANSCRIPTION_FROM_RNA_POLYMERASE_II_PROMOTER_IN_RESPONSE_TO_HYPOXIA	39	1.983734	<0.001	0.002311
206	GO_REGULATION_OF_IMMUNE_EFFECTOR_PROCESS	171	1.980751	<0.001	0.002391
207	GO_REGULATION_OF_DNA_REPAIR	53	1.975482	0.001147	0.002519

208	GO_OOGENESIS	24	1.970779	<0.001	0.002678
209	GO_SCF_DEPENDENT_PROTEASOMAL_UBIQUITIN_DEPENDENT_PROTEIN_CATABOLIC_PROCESS	44	1.967246	<0.001	0.002771
210	GO_REGULATION_OF_VIRAL_GENOME_REPLICATION	47	1.961266	<0.001	0.002976
211	GO_MALE_MEIOTIC_NUCLEAR_DIVISION	16	1.953987	0.001404	0.003268
212	GO_DNA_DAMAGE_RESPONSE_DETECTION_OF_DNA_DAMAGE	24	1.9514	<0.001	0.003385
213	GO_CELLULAR_PROTEIN_CONTAINING_COMPLEX_ASSEMBLY	357	1.95013	<0.001	0.003449
214	GO_POSITIVE_REGULATION_OF_CELL_ACTIVATION	151	1.946708	<0.001	0.003591
215	GO_POSITIVE_REGULATION_OF_DNA_BIOSYNTHETIC_PROCESS	28	1.946124	<0.001	0.00359
216	GO_NUCLEOSIDE_MONOPHOSPHATE_BIOSYNTHETIC_PROCESS	21	1.944585	<0.001	0.003633
217	GO_MITOCHONDRIAL_TRANSLATION	83	1.942321	<0.001	0.003733
218	GO_RESPONSE_TO_VIRUS	122	1.938892	<0.001	0.003821
219	GO_LEUKOCYTE_APOPTOTIC_PROCESS	34	1.930379	0.001235	0.004323
220	GO_REGULATION_OF_LYMPHOCYTE_ACTIVATION	184	1.927579	<0.001	0.004454
221	GO_RESPIRATORY_ELECTRON_TRANSPORT_CHAIN	68	1.926147	<0.001	0.004511
222	GO_OOCYTE_DIFFERENTIATION	18	1.926139	0.001339	0.004491
223	GO_REGULATION_OF_IMMUNE_RESPONSE	370	1.922474	<0.001	0.004673
224	GO_REPRODUCTION	346	1.921988	<0.001	0.004667
225	GO_REGULATION_OF_UBIQUITIN_PROTEIN_TRANSFERASE_ACTIVITY	21	1.918155	0.002706	0.0049
226	GO_POSITIVE_REGULATION_OF_FIBROBLAST_PROLIFERATION	15	1.914149	<0.001	0.005157
227	GO_DNA_DEPENDENT_DNA_REPLICATION_MAINTENANCE_OF_FIDELITY	22	1.912336	0.002699	0.005303
228	GO_DEFENSE_RESPONSE_TO_OTHER_ORGANISM	357	1.912333	<0.001	0.005279
229	GO_CYTOSKELETON_ORGANIZATION	388	1.906033	<0.001	0.005709
230	GO_LYMPHOCYTE_ACTIVATION	261	1.905756	<0.001	0.005699
231	GO_REGULATION_OF_DOUBLE_STRAND_BREAK_REPAIR	37	1.901481	0.002436	0.005983
232	GO_PYRIMIDINE_NUCLEOSIDE_METABOLIC_PROCESS	18	1.899389	<0.001	0.00613
233	GO_POSITIVE_REGULATION_OF_INNATE_IMMUNE_RESPONSE	80	1.89917	0.001079	0.006118
234	GO_POSITIVE_REGULATION_OF_PROTEIN_LOCALIZATION_TO_NUCLEUS	27	1.896991	0.002571	0.006291
235	GO_TRANSLATIONAL_ELONGATION	72	1.896146	0.002203	0.006315
236	GO_NON_RECOMBINATIONAL_REPAIR	35	1.894803	<0.001	0.006404
237	GO_NEGATIVE_REGULATION_OF_VIRAL_PROCESS	42	1.891827	0.001182	0.006645
238	GO_BASE_EXCISION_REPAIR	23	1.891113	0.005195	0.006667
239	GO_GLYCOSYL_COMPOUND_METABOLIC_PROCESS	43	1.889059	<0.001	0.006746
240	GO_REGULATION_OF_HEMATOPOIETIC_PROGENITOR_CELL_DIFFERENTIATION	40	1.887446	0.001182	0.006849
241	GO_ERROR_PRONE_TRANSLESION_SYNTHESIS	16	1.886841	0.001403	0.006852
242	GO_REGENERATION	47	1.884659	0.001193	0.007003
243	GO_RECEPTOR_MEDIATED_ENDOCYTOSIS	117	1.882633	<0.001	0.007139
244	GO_REGULATION_OF_HEMATOPOIETIC_STEM_CELL_DIFFERENTIATION	39	1.88115	0.001209	0.007245
245	GO_IMMUNE_EFFECTOR_PROCESS	420	1.880678	<0.001	0.007226
246	GO_PYRIMIDINE_CONTAINING_COMPOUND_METABOLIC_PROCESS	42	1.880187	0.001192	0.007234
247	GO_RESPONSE_TO_OXYGEN_LEVELS	94	1.87915	<0.001	0.007288
248	GO_NEGATIVE_REGULATION_OF_VIRAL_LIFE_CYCLE	37	1.878525	0.002472	0.00731
249	GO_CELLULAR_RESPIRATION	95	1.876912	<0.001	0.00742
250	GO_CELLULAR_RESPONSE_TO_OXYGEN_LEVELS	66	1.872523	<0.001	0.007744
251	GO_MITOCHONDRIAL_ELECTRON_TRANSPORT_NADH_TO_UBIQUINONE	36	1.862904	0.00615	0.008674

252	GO_ESTABLISHMENT_OF_ORGANELLE_LOCALIZATION	128	1.860415	<0.001	0.008892
253	GO_REGULATION_OF_CENTROSOME_DUPLICATION	16	1.858515	0.001379	0.009005
254	GO_CELL_RECOGNITION	88	1.858225	<0.001	0.009003
255	GO_DEOXYRIBONUCLEOTIDE_METABOLIC_PROCESS	21	1.858064	0.001353	0.008981
256	GO_RESPONSE_TO_ESTRADIOL	25	1.855981	0.006494	0.009132
257	GO_TRANSCRIPTION_COUPLED_NUCLEOTIDE_EXCISION_REPAIR	34	1.854288	0.003717	0.009295
258	GO_ERROR_FREE_TRANSLESION_SYNTHESIS	16	1.854211	0.001439	0.009265
259	GO_NUCLEOTIDE_EXCISION_REPAIR_DNA_GAP_FILLING	15	1.854204	0.002755	0.00923
260	GO_VIRAL_GENOME_REPLICATION	56	1.853335	0.001131	0.009312
261	GO_DNA_METHYLATION_OR_DEMETHYLATION	30	1.851401	0.002454	0.009475
262	GO_DEFENSE_RESPONSE_TO_VIRUS	95	1.846987	<0.001	0.009981
263	GO_DEVELOPMENT_OF_PRIMARY_SEXUAL_CHARACTERISTICS	56	1.846439	0.001149	0.010001
264	GO_CELLULAR_RESPONSE_TO_REACTIVE_OXYGEN_SPECIES	48	1.845131	<0.001	0.010076
265	GO_MITOCHONDRIAL_GENE_EXPRESSION	92	1.842366	<0.001	0.01034
266	GO_REGULATION_OF_ORGANELLE_ORGANIZATION	389	1.841233	<0.001	0.010416
267	GO_POSITIVE_REGULATION_OF_DNA_BINDING	21	1.840075	0.002567	0.010488
268	GO_REGULATION_OF_PROTEIN_SERINE_THREONINE_KINASE_ACTIVITY	141	1.839018	<0.001	0.010592
269	GO_NUCLEIC_ACID_PHOSPHODIESTER_BOND_HYDROLYSIS	102	1.837548	<0.001	0.01072
270	GO_ANTIGEN_PROCESSING_AND_PRESENTATION_OF_EXOGENOUS_PEP TIDE_ANTIGEN_VIA_MHC_CLASS_I	36	1.837447	0.004866	0.010693
271	GO_DNA_MODIFICATION	40	1.836339	0.00243	0.010798
272	GO_POSITIVE_REGULATION_OF_IMMUNE_SYSTEM_PROCESS	389	1.826306	<0.001	0.012204
273	GO_MICROTUBULE_BASED_MOVEMENT	91	1.820981	<0.001	0.012917
274	GO_RESPONSE_TO_HYDROGEN_PEROXIDE	37	1.819235	<0.001	0.013124
275	GO_NUCLEOTIDE_EXCISION_REPAIR	49	1.818022	0.001151	0.013259
276	GO_RESPONSE_TO_BACTERIUM	190	1.815885	<0.001	0.013519
277	GO_NUCLEOSIDE_MONOPHOSPHATE_METABOLIC_PROCESS	34	1.815581	0.011166	0.01351
278	GO_RESPONSE_TO_REACTIVE_OXYGEN_SPECIES	63	1.812933	<0.001	0.013877
279	GO_ONE_CARBON_METABOLIC_PROCESS	16	1.812666	0.009845	0.013876
280	GO_POSITIVE_REGULATION_OF_RESPONSE_TO_BIOTIC_STIMULUS	90	1.812425	0.001054	0.013875
281	GO_CELLULAR_RESPONSE_TO_IONIZING_RADIATION	17	1.811922	0.004082	0.013895
282	GO_REGULATION_OF_DNA_RECOMBINATION	37	1.810406	0.00612	0.014112
283	GO_CELLULAR_RESPONSE_TO_ABIOTIC_STIMULUS	71	1.809695	0.001125	0.014161
284	GO_RIBONUCLEOSIDE_METABOLIC_PROCESS	24	1.808929	<0.001	0.014246
285	GO_ORGANELLE_ASSEMBLY	255	1.808865	<0.001	0.014211
286	GO_RESPONSE_TO_BIOTIC_STIMULUS	470	1.808661	<0.001	0.014209
287	GO_ACTIVATION_OF_INNATE_IMMUNE_RESPONSE	57	1.803976	0.002245	0.014917
288	GO_AGING	73	1.802949	0.0022	0.01503
289	GO_HEMATOPOIETIC_STEM_CELL_DIFFERENTIATION	44	1.802802	0.005862	0.015008
290	GO_REGULATION_OF_MRNA_CATABOLIC_PROCESS	68	1.802286	0.001125	0.015035
291	GO_GLYCOSYL_COMPOUND_BIOSYNTHETIC_PROCESS	15	1.798459	0.006831	0.015637
292	GO_RESPONSE_TO_ETHANOL	24	1.797552	0.002564	0.01577
293	GO_LEUKOCYTE_MIGRATION	165	1.794595	<0.001	0.016235
294	GO_NADH_DEHYDROGENASE_COMPLEX_ASSEMBLY	40	1.792669	0.001175	0.016506
295	GO_REGULATION_OF_INNATE_IMMUNE_RESPONSE	110	1.790694	<0.001	0.016796

296	GO_PROTEASOMAL_PROTEIN_CATABOLIC_PROCESS	163	1.789846	<0.001	0.016888
297	GO_PROTEASOMAL_UBIQUITIN_INDEPENDENT_PROTEIN_CATABOLIC_PROCESS	15	1.786265	0.00813	0.017374
298	GO_FIBROBLAST_PROLIFERATION	24	1.784353	0.002649	0.01767
299	GO_REGULATION_OF_SYMBIOTIC_PROCESS	87	1.784081	0.001096	0.017645
300	GO_REGULATION_OF_DOUBLE_STRAND_BREAK_REPAIR_VIA_HOMOLOGOUS_RECOMBINATION	21	1.783543	0.005236	0.017686
301	GO_RESPONSE_TO_LIGHT_STIMULUS	77	1.783326	<0.001	0.017652
302	GO_CELLULAR_RESPONSE_TO_HYDROGEN_PEROXIDE	24	1.780257	0.00271	0.018159
303	GO_VITAMIN_METABOLIC_PROCESS	38	1.77591	0.003576	0.018965
304	GO_RESPONSE_TO_DRUG	85	1.773454	0.00216	0.019361
305	GO_GAMETE_GENERATION	156	1.773305	<0.001	0.019311
306	GO_INTERFERON_GAMMA_MEDIATED_SIGNALING_PATHWAY	33	1.771817	0.004902	0.019561
307	GO_REGULATION_OF_CELLULAR_KETONE_METABOLIC_PROCESS	62	1.771418	0.002268	0.019556
308	GO_NEGATIVE_REGULATION_OF_CELLULAR_COMPONENT_ORGANIZATION	188	1.766044	<0.001	0.020563
309	GO_TELOMERE_MAINTENANCE_VIA_TELOMERE_LENGTHENING	32	1.765556	0.007813	0.020604
310	GO_POSITIVE_REGULATION_OF_CHROMOSOME_ORGANIZATION	59	1.762532	0.001148	0.021223
311	GO_SEX_DIFFERENTIATION	70	1.762354	0.003322	0.021188
312	GO_ORGANELLE_LOCALIZATION	194	1.762103	<0.001	0.021158
313	GO_NUCLEOSIDE_CATABOLIC_PROCESS	16	1.760962	0.009602	0.021354
314	GO_RESPONSE_TO_CADMIUM_ION	24	1.760409	0.013995	0.021405
315	GO_RESPONSE_TO_ABIOTIC_STIMULUS	303	1.759674	<0.001	0.021509
316	GO_AMINE_METABOLIC_PROCESS	53	1.757814	0.006881	0.021885
317	GO_REGULATION_OF_LEUKOCYTE_APOPTOTIC_PROCESS	26	1.757092	0.010025	0.021974
318	GO_PROTEIN_MODIFICATION_BY_SMALL_PROTEIN_CONJUGATION_OR_REMOVAL	321	1.756748	<0.001	0.021982
319	GO_ANTIGEN_PROCESSING_AND_PRESENTATION_OF_PEPTIDE_ANTIGEN_VIA_MHC_CLASS_I	41	1.756233	0.004646	0.022062
320	GO_PROTEIN_INSERTION_INTO_MEMBRANE	30	1.755943	0.005031	0.022049
321	GO_REGULATION_OF_VIRAL_LIFE_CYCLE	62	1.755794	0.002294	0.022017
322	GO_REGULATION_OF_PROTEIN_MODIFICATION_BY_SMALL_PROTEIN_CONJUGATION_OR_REMOVAL	73	1.755766	0.004469	0.021952
323	GO_MODIFICATION_DEPENDENT_MACROMOLECULE_CATABOLIC_PROCESS	207	1.754588	<0.001	0.022118
324	GO_WATER_SOLUBLE_VITAMIN_METABOLIC_PROCESS	30	1.753777	0.007624	0.022225
325	GO_RNA_DEPENDENT_DNA_BIOSYNTHETIC_PROCESS	28	1.753318	0.007595	0.022287
326	GO_CARBOHYDRATE_DERIVATIVE_CATABOLIC_PROCESS	53	1.75011	0.004603	0.022996
327	GO_POSITIVE_REGULATION_OF_PROTEIN_MODIFICATION_BY_SMALL_PROTEIN_CONJUGATION_OR_REMOVAL	42	1.748507	0.009852	0.023274
328	GO_CELL_MATURATION	39	1.748308	0.004796	0.023252
329	GO_ESTABLISHMENT_OF_PROTEIN_LOCALIZATION_TO_MITOCHONDRIAL_MEMBRANE	26	1.746124	0.003979	0.023716
330	GO_POSITIVE_REGULATION_OF_CELL_DIVISION	28	1.745719	0.006234	0.023734
331	GO_PYRIMIDINE_CONTAINING_COMPOUND_BIOSYNTHETIC_PROCESS	18	1.740662	0.013316	0.024935
332	GO_ELECTRON_TRANSPORT_CHAIN	89	1.738454	0.001073	0.025354
333	GO_NUCLEOSIDE_TRIPHOSPHATE_METABOLIC_PROCESS	47	1.734977	0.005734	0.02612
334	GO_PROTEIN_POLYUBIQUITINATION	113	1.733323	<0.001	0.026464
335	GO_MALE_GAMETE_GENERATION	124	1.731613	<0.001	0.026861
336	GO_MISMATCH_REPAIR	15	1.73027	0.015471	0.027148
337	GO_REGULATION_OF_REPRODUCTIVE_PROCESS	43	1.729133	0.002404	0.027305

338	GO_RESPONSE_TO_TOXIC_SUBSTANCE	68	1.72681	0.00453	0.027837
339	GO_AEROBIC_RESPIRATION	41	1.72423	0.00479	0.028458
340	GO_CILIARY_BASAL_BODY_PLASMA_MEMBRANE_DOCKING	38	1.723517	0.008363	0.028543
341	GO_GLYCOSYL_COMPOUND_CATABOLIC_PROCESS	16	1.722244	0.006859	0.028841
342	GO_ALPHA_AMINO_ACID_BIOSYNTHETIC_PROCESS	20	1.718858	0.010458	0.029613
343	GO_REGULATION_OF_RESPONSE_TO_BIOTIC_STIMULUS	142	1.7116	<0.001	0.031743
344	GO_REPLICATION_FORK_PROCESSING	17	1.710581	0.005405	0.032014
345	GO_REGULATION_OF_TELOMERE_MAINTENANCE	27	1.705356	0.020779	0.033503
346	GO_CELLULAR_PROTEIN_CATABOLIC_PROCESS	235	1.705159	<0.001	0.033463
347	GO_NUCLEOSIDE_PHOSPHATE_BIOSYNTHETIC_PROCESS	93	1.705011	0.001076	0.033399
348	GO_NEGATIVE_REGULATION_OF_PROTEIN_MODIFICATION_BY_SMALL_PROTEIN_CONJUGATION_OR_REMOVAL	28	1.704944	0.014066	0.033312
349	GO_NEGATIVE_REGULATION_OF_RESPONSE_TO_DNA_DAMAGE_STIMULUS	23	1.704191	0.012	0.033463
350	GO_RESPONSE_TO_ALCOHOL	57	1.702901	0.004556	0.033743
351	GO_SYMBIOTIC_PROCESS	310	1.702518	<0.001	0.033739
352	GO_GERM_CELL_DEVELOPMENT	63	1.700148	0.003363	0.034375
353	GO_CELL_AGING	35	1.698683	0.008516	0.034799
354	GO_POSITIVE_REGULATION_OF_MEMBRANE_PERMEABILITY	18	1.698142	0.018519	0.034862
355	GO_HOMOLOGOUS_CHROMOSOME_PAIRING_AT_MEIOSIS	20	1.697556	0.017833	0.034995
356	GO_PEPTIDYL_THREONINE_MODIFICATION	43	1.697215	0.010714	0.035036
357	GO_POSITIVE_REGULATION_OF_LYMPHOCYTE_MEDIATED_IMMUNITY	35	1.696865	0.007528	0.035047
358	GO_SEXUAL_REPRODUCTION	186	1.696533	<0.001	0.035039
359	GO_NUCLEAR_TRANSPORT	110	1.696281	0.005302	0.035003
360	GO_T_CELL_RECEPTOR_SIGNALING_PATHWAY	90	1.694549	0.0022	0.035472
361	GO_REGULATION_OF_TRANSFERASE_ACTIVITY	306	1.69395	<0.001	0.035612
362	GO_NUCLEOTIDE_EXCISION_REPAIR_DNA_INCISION	19	1.692893	0.01328	0.03587
363	GO_PROTEIN_MODIFICATION_BY_SMALL_PROTEIN_CONJUGATION	272	1.692665	<0.001	0.035867
364	GO_RESPONSE_TO_VITAMIN	15	1.688382	0.025745	0.037273
365	GO_MITOCHONDRIAL_MEMBRANE_ORGANIZATION	55	1.686961	0.004561	0.037617
366	GO_CELLULAR_KETONE_METABOLIC_PROCESS	78	1.686719	<0.001	0.037584
367	GO_MITOCHONDRIAL_OUTER_MEMBRANE_PERMEABILIZATION	17	1.686419	0.017735	0.037593
368	GO_PYRIMIDINE_NUCLEOTIDE_METABOLIC_PROCESS	24	1.68417	0.011628	0.038234
369	GO_RESPONSE_TO_INTERFERON_GAMMA	58	1.683603	0.00232	0.038335
370	GO_HEMATOPOIETIC_PROGENITOR_CELL_DIFFERENTIATION	67	1.681042	0.001135	0.039159
371	GO_REGULATION_OF_TELOMERASE_ACTIVITY	18	1.679812	0.025606	0.03954
372	GO_DICARBOXYLIC_ACID_METABOLIC_PROCESS	34	1.678086	0.019417	0.040093
373	GO_POSITIVE_REGULATION_OF_CELL_KILLING	21	1.676451	0.022636	0.040536
374	GO_LYMPHOCYTE_APOPTOTIC_PROCESS	26	1.67253	0.018892	0.041793
375	GO_REGULATION_OF_REACTIVE_OXYGEN_SPECIES_METABOLIC_PROCESS	41	1.671652	0.011601	0.041983
376	GO_INNATE_IMMUNE_RESPONSE_ACTIVATING_SIGNAL_TRANSDUCTION	45	1.668992	0.020166	0.042904
377	GO_MITOCHONDRION_ORGANIZATION	214	1.668291	<0.001	0.043007
378	GO_CELLULAR_RESPONSE_TO_CHEMICAL_STRESS	92	1.663922	0.004343	0.044627
379	GO_POSITIVE_REGULATION_OF_TRANSFERASE_ACTIVITY	198	1.662632	0.004057	0.045022
380	GO_POSITIVE_REGULATION_OF_BINDING	55	1.660549	0.010333	0.045771

381	GO_MICROTUBULE_POLYMERIZATION	17	1.659573	0.023288	0.045987
382	GO_RESPONSE_TO_NUTRIENT	31	1.659394	0.025063	0.045931
383	GO_ATP_METABOLIC_PROCESS	125	1.658947	0.002055	0.045989
384	GO_MITOCHONDRIAL_RESPIRATORY_CHAIN_COMPLEX_ASSEMBLY	54	1.658928	0.009281	0.045875
385	GO_REPRODUCTIVE_SYSTEM_DEVELOPMENT	104	1.656948	0.008412	0.046505
386	GO_RESPONSE_TO_FATTY_ACID	18	1.656561	0.01931	0.046572
387	GO_SPLICEOSOMAL_SNRNP_ASSEMBLY	16	1.65636	0.026087	0.046539
388	GO_REGULATION_OF_OXIDOREDUCTASE_ACTIVITY	23	1.655922	0.02574	0.046607
389	GO_REGULATION_OF_BINDING	108	1.654314	0.008529	0.047107
390	GO_COVALENT_CHROMATIN_MODIFICATION	133	1.651354	0.002099	0.048162
391	GO_INNER_MITOCHONDRIAL_MEMBRANE_ORGANIZATION	28	1.649913	0.022843	0.048651
392	GO_PROTEIN_COMPLEX_OLIGOMERIZATION	51	1.649878	0.01979	0.048551
393	GO_NEURAL_PRECURSOR_CELL_PROLIFERATION	37	1.649489	0.01087	0.048591
394	GO_SOMATIC_DIVERSIFICATION_OF_IMMUNE_RECEPTORS	29	1.649053	0.030151	0.048636
395	GO_REGULATION_OF_STEM_CELL_DIFFERENTIATION	48	1.64763	0.01555	0.049132
396	GO_NUCLEOBASE_CONTAINING_SMALL_MOLECULE_CATABOLIC_PROCE SS	21	1.647014	0.02656	0.049304
397	GO_DEFENSE_RESPONSE	481	1.645278	<0.001	0.049856
398	GO_PROTEIN_TETRAMERIZATION	23	1.644238	0.023904	0.050211
399	GO_RNA_PHOSPHODIESTER_BOND_HYDROLYSIS	52	1.644072	0.023121	0.050168
400	GO_POSITIVE_REGULATION_OF_PRODUCTION_OF_MOLECULAR_MEDIA TOR_OF_IMMUNE_RESPONSE	26	1.643562	0.016624	0.050228
401	GO_REGULATION_OF_TELOMERE_MAINTENANCE_VIA_TELOMERE_LEN GTHENING	22	1.641544	0.020253	0.051038
402	GO_NUCLEOBASE_CONTAINING_SMALL_MOLECULE_BIOSYNTHETIC_PR OCESS	42	1.64031	0.015588	0.051507
403	GO_MULTICELLULAR_ORGANISM_REPRODUCTION	189	1.640258	<0.001	0.051407
404	GO_REGULATION_OF_REACTIVE_OXYGEN_SPECIES_BIOSYNTHETIC_PRO CESS	15	1.637691	0.026611	0.052358
405	GO_ANTIMICROBIAL_HUMORAL_RESPONSE	17	1.634835	0.018945	0.053467
406	GO_NUCLEAR_ENVELOPE_ORGANIZATION	16	1.634627	0.026667	0.053438
407	GO_CELLULAR_NITROGEN_COMPOUND_CATABOLIC_PROCESS	186	1.633132	0.002045	0.053916
408	GO_CELLULAR_PROTEIN_COMPLEX_DISASSEMBLY	110	1.632255	0.007511	0.054196
409	GO_POSITIVE_REGULATION_OF_TELOMERE_MAINTENANCE	20	1.629744	0.028351	0.055241
410	GO_EMBRYONIC_PLACENTA_DEVELOPMENT	17	1.628528	0.022758	0.055662
411	GO_ENDOCYTOSIS	223	1.628339	0.001022	0.055625
412	GO_PURINE_NUCLEOSIDE_TRIPHOSPHATE_METABOLIC_PROCESS	35	1.628335	0.017178	0.05549
413	GO_REGULATION_OF_NUCLEOCYTOPLASMIC_TRANSPORT	36	1.627776	0.022727	0.055626
414	GO_CELLULAR_MACROMOLECULE_CATABOLIC_PROCESS	338	1.62652	<0.001	0.056077
415	GO_NUCLEAR_EXPORT	60	1.623381	0.012332	0.057419
416	GO_REGULATION_OF_PROTEIN_LOCALIZATION_TO_NUCLEUS	36	1.62321	0.020408	0.057363
417	GO_PROTEIN_CATABOLIC_PROCESS	274	1.622618	<0.001	0.057496
418	GO_POSITIVE_REGULATION_OF_COLD_INDUCED_THERMOGENESIS	21	1.621743	0.019659	0.057779
419	GO_POSITIVE_REGULATION_OF_MITOCHONDRION_ORGANIZATION	35	1.618035	0.021845	0.059528
420	GO_DEVELOPMENTAL_PROCESS_INVOLVED_IN_REPRODUCTION	218	1.617983	0.001008	0.059405
421	GO_RNA_CATABOLIC_PROCESS	123	1.617403	<0.001	0.059578
422	GO_MACROMOLECULE_CATABOLIC_PROCESS	396	1.616249	<0.001	0.059946
423	GO_PYRIMIDINE_CONTAINING_COMPOUND_CATABOLIC_PROCESS	21	1.615983	0.034853	0.059931

424	GO_REGULATION_OF_DNA_TEMPLATED_TRANSCRIPTION_ELONGATION	16	1.612316	0.034965	0.061568
425	GO_VIRAL_LIFE_CYCLE	112	1.609513	0.003202	0.062873
426	GO_TUMOR_NECROSIS_FACTOR_MEDIATED_SIGNALING_PATHWAY	67	1.609432	0.006689	0.062754
427	GO_DNA_TEMPLATED_TRANSCRIPTION_ELONGATION	39	1.607808	0.015495	0.063506
428	GO_REGULATION_OF_PROTEIN_KINASE_ACTIVITY	229	1.607273	0.002014	0.063628
429	GO_POSITIVE_REGULATION_OF_DEFENSE_RESPONSE	113	1.605062	0.003148	0.064584
430	GO_REGULATION_OF_CHROMATIN_ORGANIZATION	53	1.603078	0.013793	0.065471
431	GO_ENERGY_DERIVATION_BY_OXIDATION_OF_ORGANIC_COMPOUNDS	123	1.603033	0.003122	0.065347
432	GO_REGULATION_OF_CELL_ACTIVATION	217	1.60163	0.001011	0.065927
433	GO_PROTEIN_LOCALIZATION_TO_CYTOSKELETON	22	1.60154	0.030383	0.065807
434	GO_B_CELL_ACTIVATION_INVOLVED_IN_IMMUNE_RESPONSE	27	1.60145	0.025221	0.065714
435	GO_ANATOMICAL_STRUCTURE_HOMEOSTASIS	145	1.600763	0.001036	0.06593
436	GO_NATURAL_KILLER_CELL_MEDIATED_IMMUNITY	19	1.595916	0.030144	0.068314
437	GO_NUCLEOSIDE_TRIPHOSPHATE_BIOSYNTHETIC_PROCESS	37	1.595474	0.02952	0.068408
438	GO_REGULATION_OF_MRNA_METABOLIC_PROCESS	102	1.594077	0.003188	0.068975
439	GO_RIBONUCLEOPROTEIN_COMPLEX_BIOGENESIS	129	1.592725	0.00625	0.069532
440	GO_DNA_METHYLATION	19	1.591644	0.032043	0.070027
441	GO_POSITIVE_REGULATION_OF_TYPE_I_INTERFERON_PRODUCTION	24	1.589988	0.040951	0.070806
442	GO_POSTTRANSCRIPTIONAL_REGULATION_OF_GENE_EXPRESSION	183	1.586612	0.002045	0.072483
443	GO_ORGANONITROGEN_COMPOUND_CATABOLIC_PROCESS	378	1.584838	<0.001	0.073366
444	GO_POSITIVE_REGULATION_OF_NUCLEOCYTOPLASMIC_TRANSPORT	17	1.584734	0.056376	0.073281
445	GO_GENE_SILENCING	64	1.584671	0.025959	0.073164
446	GO_MALE_SEX_DIFFERENTIATION	45	1.583849	0.024764	0.073544
447	GO_RIBONUCLEOSIDE_TRIPHOSPHATE_METABOLIC_PROCESS	34	1.582974	0.026895	0.07387
448	GO_REGULATION_OF_MITOCHONDRIAL_MEMBRANE_PERMEABILITY_IN VOLVED_IN_APOPTOTIC_PROCESS	18	1.582619	0.041783	0.073914
449	GO_RNA_MODIFICATION	40	1.582483	0.030374	0.073814
450	GO_REGULATION_OF_GENE_SILENCING	26	1.576217	0.032138	0.077348
451	GO_REGULATION_OF_DEFENSE_RESPONSE_TO_VIRUS_BY_HOST	16	1.574751	0.039295	0.078075
452	GO_REACTIVE_OXYGEN_SPECIES_BIOSYNTHETIC_PROCESS	21	1.572992	0.033943	0.078938
453	GO_INTERLEUKIN_1_MEDIATED_SIGNALING_PATHWAY	44	1.572547	0.028202	0.079006
454	GO_REGULATION_OF_CYTOSKELETON_ORGANIZATION	162	1.57144	0.005107	0.079442
455	GO_ORGANIC_CYCLIC_COMPOUND_CATABOLIC_PROCESS	194	1.570487	0.001019	0.079818
456	GO_CELLULAR_AMINO_ACID_BIOSYNTHETIC_PROCESS	23	1.570029	0.036745	0.079933
457	GO_SERINE_FAMILY_AMINO_ACID_METABOLIC_PROCESS	17	1.566607	0.045872	0.081857
458	GO_CELLULAR_RESPONSE_TO_HEAT	30	1.563758	0.038557	0.083392
459	GO_SOMATIC_DIVERSIFICATION_OF_IMMUNOGLOBULINS	24	1.558204	0.051047	0.086664
460	GO_REGULATION_OF_MITOCHONDRIAL_OUTER_MEMBRANE_PERMEAB ILIZATION_INVOLVED_IN_APOPTOTIC_SIGNALING_PATHWAY	16	1.556887	0.05007	0.087375
461	GO_NEGATIVE_REGULATION_OF_LEUKOCYTE_APOPTOTIC_PROCESS	15	1.556218	0.04755	0.087589
462	GO_MULTI_ORGANISM_PROCESS	247	1.555206	0.004044	0.088071
463	GO_PROTEIN_CONTAINING_COMPLEX_DISASSEMBLY	133	1.554964	0.006263	0.088069
464	GO_IMMUNE_SYSTEM_DEVELOPMENT	301	1.553555	<0.001	0.088818
465	GO_PROTEIN_LOCALIZATION_TO_MITOCHONDRION	57	1.553011	0.028249	0.089042
466	GO_NUCLEUS_ORGANIZATION	39	1.547476	0.031439	0.092697
467	GO_SPERMATID_DIFFERENTIATION	40	1.546063	0.036817	0.093494

468	GO_PROTEIN_HOMOOLIGOMERIZATION	42	1.545881	0.040964	0.093416
469	GO_PROTEIN_HOMOTETRAMERIZATION	16	1.544952	0.05663	0.093822
470	GO_RNA_SPLICING_VIA_TRANSESTERIFICATION_REACTIONS	121	1.540893	0.009414	0.096327
471	GO_POSITIVE_T_CELL_SELECTION	16	1.538364	0.04577	0.097929
472	GO_EXECUTION_PHASE_OF_APOPTOSIS	20	1.537629	0.049869	0.098227
473	GO_PROTEIN_AUTOUBIQUITINATION	20	1.535029	0.060686	0.099811
474	GO_POSITIVE_REGULATION_OF_DNA_REPAIR	30	1.534402	0.026316	0.100069
475	GO_RNA_PHOSPHODIESTER_BOND_HYDROLYSIS_ENDONUCLEOLYTIC	25	1.53404	0.05598	0.10014
476	GO_RESPONSE_TO_KETONE	44	1.531756	0.036145	0.101514
477	GO_POSITIVE_REGULATION_OF_VIRAL_GENOME_REPLICATION	15	1.531369	0.047076	0.101607
478	GO_POST_TRANSLATIONAL_PROTEIN_MODIFICATION	104	1.530463	0.011653	0.102054
479	GO_DETECTION_OF_STIMULUS	61	1.528669	0.024859	0.103088
480	GO_POSITIVE_REGULATION_OF_CANONICAL_WNT_SIGNALING_PATHWAY	52	1.528574	0.034682	0.102972
481	GO_T_CELL_APOPTOTIC_PROCESS	19	1.527464	0.058187	0.103529
482	GO_NEGATIVE_REGULATION_OF_DNA_METABOLIC_PROCESS	32	1.52738	0.050617	0.103396
483	GO_ESTABLISHMENT_OF_TISSUE_POLARITY	48	1.524204	0.050459	0.105507
484	GO_ATP_SYNTHESIS_COUPLED_PROTON_TRANSPORT	16	1.523216	0.052198	0.106046
485	GO_PROTEIN_DEPOLYMERIZATION	42	1.522648	0.049161	0.106273
486	GO_RIBOSOME_BIOGENESIS	84	1.521472	0.016216	0.106937
487	GO_TYPE_I_INTERFERON_PRODUCTION	44	1.521429	0.043376	0.106758
488	GO_REGULATION_OF_ALPHA_BETA_T_CELL_ACTIVATION	33	1.520677	0.047919	0.107101
489	GO_POSITIVE_REGULATION_OF_LEUKOCYTE_MEDIATED_IMMUNITY	41	1.519101	0.045244	0.108034
490	GO_MEMBRANE_ORGANIZATION	281	1.518413	<0.001	0.108338
491	GO_TRANSCRIPTION_ELONGATION_FROM_RNA_POLYMERASE_II_PROMOTER	32	1.515362	0.054414	0.110496
492	GO_POSITIVE_REGULATION_OF_RESPONSE_TO_DNA_DAMAGE_STIMULUS	39	1.512723	0.051807	0.112337
493	GO_PURINE_CONTAINING_COMPOUND_BIOSYNTHETIC_PROCESS	74	1.512197	0.032609	0.112528
494	GO_NEGATIVE_REGULATION_OF_BIOSYNTHETIC_PROCESS	417	1.511903	<0.001	0.112556
495	GO_APOPTOTIC_SIGNALING_PATHWAY	160	1.510795	0.01032	0.113245
496	GO_REGULATION_OF_MONOOXYGENASE_ACTIVITY	16	1.509431	0.064738	0.114012
497	GO_RIBONUCLEOSIDE_TRIPHOSPHATE_BIOSYNTHETIC_PROCESS	30	1.509074	0.063119	0.114049
498	GO_REGULATION_OF_CIRCADIAN_RHYTHM	33	1.508919	0.05985	0.113926
499	GO_REGULATION_OF_HEMOPOIESIS	147	1.508091	0.008377	0.114349
500	GO_CORTICAL_CYTOSKELETON_ORGANIZATION	18	1.505591	0.0625	0.116193
501	GO_CELL_KILLING	44	1.50507	0.04142	0.116398
502	GO_MITOCHONDRIAL_TRANSPORT	93	1.5045	0.026288	0.116612
503	GO_REGULATION_OF_CYSTEINE_TYPE_ENDOPEPTIDASE_ACTIVITY	72	1.50445	0.020089	0.11642
504	GO_CELLULAR_COMPONENT_DISASSEMBLY	199	1.504059	0.005071	0.116508
505	GO_RESPONSE_TO_INORGANIC_SUBSTANCE	146	1.503257	0.007209	0.116933
506	GO_CELLULAR_AMINO_ACID_METABOLIC_PROCESS	112	1.502827	0.019979	0.117091
507	GO_REGULATION_OF_CELLULAR_RESPONSE_TO_STRESS	213	1.49894	0.002028	0.120036
508	GO_REGULATION_OF_MITOCHONDRION_ORGANIZATION	53	1.497302	0.03805	0.12124
509	GO_BLASTOCYST_DEVELOPMENT	37	1.497297	0.053724	0.121003
510	GO_INTRINSIC_APOPTOTIC_SIGNALING_PATHWAY	79	1.496298	0.03433	0.12161
511	GO_PEPTIDYL_SERINE_MODIFICATION	91	1.496182	0.020585	0.12146

512	GO_RESPONSE_TO_COPPER_ION	16	1.49483	0.074896	0.122327
513	GO_NEGATIVE_REGULATION_OF_DNA_BINDING_TRANSCRIPTION_FACTOR_ACTIVITY	41	1.494746	0.058542	0.122172
514	GO_PLACENTA_DEVELOPMENT	36	1.494633	0.049813	0.122037
515	GO_POSITIVE_REGULATION_OF_PROTEIN_KINASE_ACTIVITY	141	1.493261	0.014523	0.122885
516	GO_NEGATIVE_REGULATION_OF_CYSTEINE_TYPE_ENDOPEPTIDASE_ACTIVITY	25	1.492619	0.075064	0.123147
517	GO_RESPONSE_TO_OXIDATIVE_STRESS	121	1.492539	0.016771	0.122957
518	GO_RESPONSE_TO_INTERFERON_BETA	19	1.491263	0.076923	0.123735
519	GO_POSITIVE_REGULATION_OF_CELL_POPULATION_PROLIFERATION	245	1.489716	0.00202	0.124851
520	GO_ANATOMICAL_STRUCTURE_MATURATION	51	1.489222	0.056471	0.125024
521	GO_PHOSPHATIDYLCHOLINE_METABOLIC_PROCESS	17	1.488939	0.069014	0.125006
522	GO_DNA_TEMPLATED_TRANSCRIPTION_TERMINATION	30	1.488795	0.054569	0.124893
523	GO_IN_UTERO_EMBRYONIC_DEVELOPMENT	103	1.487503	0.019108	0.125701
524	GO_SOMATIC_RECOMBINATION_OF_IMMUNOGLOBULIN_GENE_SEGMENTS	22	1.486109	0.050802	0.126649
525	GO_PROTEIN_ACETYLATION	60	1.483968	0.053993	0.128133
526	GO_PROTEIN_K63_LINKED_UBIQUITINATION	17	1.481604	0.078729	0.129833
527	GO_NCRNA_METABOLIC_PROCESS	135	1.481458	0.017764	0.129719
528	GO_NEGATIVE_REGULATION_OF_BINDING	45	1.481352	0.057692	0.129566
529	GO_REGULATION_OF_PROTEIN_EXPORT_FROM_NUCLEUS	19	1.47926	0.065596	0.131066
530	GO_REACTIVE_OXYGEN_SPECIES_METABOLIC_PROCESS	62	1.478422	0.041237	0.131504
531	GO_NUCLEAR_TRANSCRIBED_MRNA_CATABOLIC_PROCESS_DEADENYLATION_DEPENDENT_DECAY	18	1.475205	0.083223	0.134203
532	GO_INTRINSIC_APOPTOTIC_SIGNALING_PATHWAY_IN_RESPONSE_TO_DNA_DAMAGE	31	1.47273	0.057644	0.13619
533	GO_MORPHOGENESIS_OF_A_POLARIZED_EPITHELIUM	52	1.47073	0.051512	0.137798
534	GO_SPLICEOSOMAL_COMPLEX_ASSEMBLY	17	1.47052	0.080556	0.13774
535	GO_RNA_EXPORT_FROM_NUCLEUS	37	1.470339	0.068881	0.137627
536	GO_T_CELL_MEDIATED_IMMUNITY	40	1.467205	0.085185	0.140157
537	GO_IMPORT_INTO_NUCLEUS	46	1.466164	0.059649	0.140832
538	GO_CELL_ACTIVATION	437	1.464571	<0.001	0.142081
539	GO_NCRNA_PROCESSING	107	1.462789	0.033155	0.143538
540	GO_ORGANIC_ACID_BIOSYNTHETIC_PROCESS	90	1.462537	0.026795	0.143494
541	GO_FEMALE_SEX_DIFFERENTIATION	23	1.462511	0.081579	0.143247
542	GO_RRNA_METABOLIC_PROCESS	65	1.461592	0.051876	0.14384
543	GO_PROTEIN_LOCALIZATION_TO_NUCLEUS	78	1.460272	0.035792	0.144769
544	GO_REGULATION_OF_GLIOGENESIS	31	1.459829	0.06053	0.14492
545	GO_RIBONUCLEOSIDE_MONOPHOSPHATE_BIOSYNTHETIC_PROCESS	15	1.45899	0.074648	0.145396
546	GO_POSITIVE_REGULATION_OF_TELOMERE_MAINTENANCE_VIA_TELOMERE_LENGTHENING	15	1.45866	0.084165	0.145431
547	GO_PROTEIN_MONOUBIQUITINATION	19	1.456898	0.086022	0.146917
548	GO_TERMINATION_OF_RNA_POLYMERASE_II_TRANSCRIPTION	19	1.45652	0.081717	0.147031
549	GO_HISTONE_H3_ACETYLATION	17	1.455112	0.079609	0.148082
550	GO_RESPONSE_TO_INTERLEUKIN_1	65	1.454697	0.045556	0.148194
551	GO_T_CELL_SELECTION	18	1.453746	0.092593	0.148838
552	GO_REGULATION_OF_ORGANELLE_ASSEMBLY	46	1.453339	0.056845	0.148935
553	GO_CRANIAL_SKELETAL_SYSTEM_DEVELOPMENT	16	1.452352	0.098782	0.14963

554	GO_IMMUNOGLOBULIN_PRODUCTION_INVOLVED_IN_IMMUNOGLOBULIN_MEDIATED_IMMUNE_RESPONSE	21	1.451705	0.073333	0.149995
555	GO_RHYTHMIC_PROCESS	71	1.451391	0.052339	0.150043
556	GO_APOPTOTIC_MITOCHONDRIAL_CHANGES	35	1.450459	0.066584	0.150611
557	GO_PROTEIN_LOCALIZATION_TO_ORGANELLE	305	1.450369	0.001003	0.150441
558	GO_MEMBRANE_DOCKING	53	1.45029	0.072331	0.150236
559	GO_GENERATION_OF_PRECURSOR_METABOLITES_AND_ENERGY	201	1.44991	0.008163	0.150355
560	GO_METHYLATION	89	1.447837	0.033298	0.151994
561	GO_ACTIVATION_OF_PROTEIN_KINASE_ACTIVITY	84	1.44742	0.032859	0.152139
562	GO_RESPONSE_TO_ORGANIC_CYCLIC_COMPOUND	195	1.444613	0.010121	0.154721
563	GO_LYMPHOCYTE_ACTIVATION_INVOLVED_IN_IMMUNE_RESPONSE	55	1.441637	0.057274	0.157314
564	GO_REGULATION_OF_CELL_AGING	23	1.438983	0.09281	0.159622
565	GO_RNA_3_END_PROCESSING	46	1.438402	0.06698	0.159944
566	GO_PROTEIN_TARGETING_TO_MITOCHONDRION	40	1.43773	0.066667	0.160372
567	GO_CIRCADIAN_RHYTHM	50	1.437447	0.06752	0.160371
568	GO_POSITIVE_REGULATION_OF_ORGANELLE_ORGANIZATION	185	1.436821	0.018462	0.160713
569	GO_RNA_LOCALIZATION	68	1.43616	0.056983	0.16108
570	GO_REGULATION_OF_T_CELL_RECEPTOR_SIGNALING_PATHWAY	22	1.434977	0.093385	0.161922
571	GO_RESPONSE_TO_GAMMA_RADIATION	19	1.433708	0.104683	0.162873
572	GO_DEMETHYLATION	18	1.433439	0.112188	0.162858
573	GO_REGULATION_OF_ATP_METABOLIC_PROCESS	34	1.430559	0.073232	0.165571
574	GO_RIBOSE_PHOSPHATE_BIOSYNTHETIC_PROCESS	70	1.42955	0.0625	0.166263
575	GO_CYTOKINE_MEDIATED_SIGNALING_PATHWAY	249	1.429439	0.002022	0.166117
576	GO_PROTEOLYSIS	479	1.429355	<0.001	0.165907
577	GO_PEPTIDYL_LYSINE_METHYLATION	33	1.428257	0.094711	0.16677
578	GO_REGULATION_OF_LYMPHOCYTE_APOPTOTIC_PROCESS	19	1.426639	0.120739	0.16818
579	GO_REGULATION_OF_DOUBLE_STRAND_BREAK_REPAIR_VIA_NONHOMOLOGOUS_END_JOINING	15	1.424129	0.097765	0.170449
580	GO_NUCLEOBASE_CONTAINING_SMALL_MOLECULE_METABOLIC_PROCESS	199	1.42406	0.01631	0.170216
581	GO_MONOCARBOXYLIC_ACID_BIOSYNTHETIC_PROCESS	52	1.423308	0.064403	0.17068
582	GO_POSITIVE_REGULATION_OF_PROTEIN_SERINE_THREONINE_KINASE_ACTIVITY	76	1.423084	0.057971	0.170629
583	GO_CELLULAR_MODIFIED_AMINO_ACID_BIOSYNTHETIC_PROCESS	22	1.421968	0.099063	0.171493
584	GO_CELLULAR_RESPONSE_TO_INORGANIC_SUBSTANCE	60	1.420683	0.060433	0.172597
585	GO_RESPONSE_TO_CYTOKINE	362	1.420508	0.00502	0.172504
586	GO_T_CELL_ACTIVATION	154	1.417818	0.030738	0.175148
587	GO_EXTRINSIC_APOPTOTIC_SIGNALING_PATHWAY	64	1.416102	0.063927	0.176712
588	GO_REGULATION_OF_NEURAL_PRECURSOR_CELL_PROLIFERATION	20	1.415838	0.113233	0.1767
589	GO_RESPONSE_TO_ALKALOID	25	1.414665	0.103226	0.177731
590	GO_NEGATIVE_REGULATION_OF_NUCLEOBASE_CONTAINING_COMPOUND_METABOLIC_PROCESS	389	1.413102	0.001001	0.179135
591	GO_PROTEIN_K48_LINKED_UBIQUITINATION	22	1.412576	0.105333	0.179428
592	GO_MRNA_EXPORT_FROM_NUCLEUS	28	1.411709	0.090216	0.180081
593	GO_SMALL_MOLECULE_BIOSYNTHETIC_PROCESS	204	1.40939	0.0111	0.18231
594	GO_POSITIVE_REGULATION_OF_WNT_SIGNALING_PATHWAY	59	1.409121	0.086758	0.182327
595	GO_NEGATIVE_REGULATION_OF_CELLULAR_AMIDE_METABOLIC_PROCESS	43	1.408798	0.086435	0.182368
596	GO_REGULATION_OF_DEFENSE_RESPONSE	200	1.407863	0.019289	0.183069

597	GO_ALCOHOL_BIOSYNTHETIC_PROCESS	54	1.405817	0.067442	0.185067
598	GO_PURINE_NUCLEOSIDE_METABOLIC_PROCESS	17	1.403783	0.098592	0.186979
599	GO_NEGATIVE_REGULATION_OF_REPRODUCTIVE_PROCESS	17	1.402024	0.127072	0.188789
600	GO_TRICARBOXYLIC_ACID_CYCLE	17	1.400774	0.118046	0.18995
601	GO_RNA_SPLICING	145	1.400732	0.04456	0.189686
602	GO_NCRNA_TRANSCRIPTION	34	1.39979	0.093867	0.190429
603	GO_REGULATION_OF_PROTEASOMAL_PROTEIN_CATABOLIC_PROCESS	63	1.399619	0.075621	0.1903
604	GO_LONG_CHAIN_FATTY_ACID_TRANSPORT	19	1.399605	0.116037	0.189999
605	GO_GLAND_DEVELOPMENT	108	1.399411	0.054223	0.189901
606	GO_LYMPHOCYTE_HOMEOSTASIS	25	1.39778	0.10691	0.19152
607	GO_NEGATIVE_REGULATION_OF_NF_KAPPAB_TRANSCRIPTION_FACTOR_ACTIVITY	24	1.395425	0.117197	0.193812
608	GO_RELEASE_OF_CYTOCHROME_C_FROM_MITOCHONDRIA	17	1.393213	0.102288	0.195945
609	GO_TRNA_METABOLIC_PROCESS	51	1.391657	0.088578	0.197507
610	GO_POSITIVE_REGULATION_OF_REPRODUCTIVE_PROCESS	19	1.391459	0.137931	0.19743
611	GO_RESPONSE_TO_STEROID_HORMONE	74	1.391028	0.073064	0.197622
612	GO_RAS_PROTEIN_SIGNAL_TRANSDUCTION	96	1.3901	0.060768	0.198341
613	GO_REGULATION_OF_RESPONSE_TO_STRESS	413	1.3899	0.005005	0.198265
614	GO_POSITIVE_REGULATION_OF_PEPTIDASE_ACTIVITY	59	1.385925	0.0906	0.202477
615	GO_POSITIVE_REGULATION_OF_IMMUNE_EFFECTOR_PROCESS	63	1.385013	0.08362	0.203195
616	GO_LEUKOCYTE_MEDIATED_CYTOTOXICITY	33	1.383339	0.103832	0.204868
617	GO_REGULATION_OF_DEFENSE_RESPONSE_TO_VIRUS	27	1.378625	0.12	0.210133
618	GO_SUPEROXIDE_METABOLIC_PROCESS	17	1.377911	0.118644	0.210698
619	GO_REGULATION_OF_CELL_POPULATION_PROLIFERATION	424	1.377271	0.004004	0.211128
620	GO_MRNA_METABOLIC_PROCESS	253	1.376983	0.020161	0.211162
621	GO_RNA_PROCESSING	271	1.375227	0.012085	0.213004
622	GO_IRE1_MEDIATED_UNFOLDED_PROTEIN_RESPONSE	15	1.373152	0.153295	0.215191
623	GO_REGULATION_OF_B_CELL_PROLIFERATION	23	1.37084	0.13601	0.217678
624	GO_MULTI_ORGANISM_LOCALIZATION	20	1.369643	0.137119	0.218786
625	GO_B_CELL_DIFFERENTIATION	28	1.369297	0.118321	0.21887
626	GO_CRISTAE_FORMATION	20	1.366972	0.143799	0.22152
627	GO_PROTEIN_FOLDING	82	1.365625	0.074398	0.222932
628	GO_RESPONSE_TO_ACTIVITY	16	1.363244	0.142473	0.225542
629	GO_NEGATIVE_REGULATION_OF_RNA_BIOSYNTHETIC_PROCESS	344	1.363219	0.014028	0.225211
630	GO_REGULATION_OF_MORPHOGENESIS_OF_AN_EPITHELIUM	57	1.362589	0.096998	0.225633
631	GO_TRNA_PROCESSING	35	1.362094	0.113971	0.225893
632	GO_NEGATIVE_REGULATION_OF_DNA_RECOMBINATION	15	1.361999	0.142659	0.225657
633	GO_DETOXIFICATION	41	1.360936	0.13422	0.226617
634	GO_NEGATIVE_REGULATION_OF_CHROMATIN_ORGANIZATION	15	1.359825	0.13881	0.227748
635	GO_REGULATION_OF_LYMPHOCYTE_MEDIATED_IMMUNITY	47	1.358695	0.126857	0.228852
636	GO_REGULATION_OF_MEMBRANE_PERMEABILITY	23	1.355976	0.150065	0.232088
637	GO_POSITIVE_REGULATION_OF_VIRAL_LIFE_CYCLE	24	1.355824	0.135526	0.2319
638	GO_RNA_METHYLATION	15	1.35514	0.136045	0.232383
639	GO_ALPHA_BETA_T_CELL_ACTIVATION	49	1.353192	0.117305	0.234646
640	GO_NON_CANONICAL_WNT_SIGNALING_PATHWAY	51	1.353004	0.126168	0.234513

641	GO_POSITIVE_REGULATION_OF_CELLULAR_PROTEIN_LOCALIZATION	105	1.34983	0.063034	0.238428
642	GO_CELLULAR_MODIFIED_AMINO_ACID_METABOLIC_PROCESS	55	1.349141	0.112412	0.23896
643	GO_REGULATION_OF_SMALL_MOLECULE_METABOLIC_PROCESS	130	1.346666	0.070157	0.241835
644	GO_ACTIVATION_OF_MAPK_ACTIVITY	36	1.346363	0.132191	0.241868
645	GO_POSITIVE_REGULATION_OF_DOUBLE_STRAND_BREAK_REPAIR	18	1.346252	0.14011	0.241685
646	GO_HIPPOCAMPUS_DEVELOPMENT	20	1.34581	0.138381	0.241883
647	GO_POSITIVE_REGULATION_OF_NEURON_APOPTOTIC_PROCESS	20	1.340368	0.146505	0.248884
648	GO_CARDIAC_MUSCLE_CELL_PROLIFERATION	15	1.340263	0.158345	0.248639
649	GO_STEM_CELL_PROLIFERATION	22	1.339787	0.143416	0.248906
650	GO_REGULATION_OF_PEPTIDYL_LYSINE_ACETYLATION	23	1.339335	0.145833	0.249093
651	GO_REGULATION_OF_T_CELL_DIFFERENTIATION	54	1.338999	0.115976	0.249176
652	GO_HISTONE_UBIQUITINATION	17	1.335654	0.145251	0.253519
653	GO_NEGATIVE_REGULATION_OF_CANONICAL_WNT_SIGNALING_PATHWAY	58	1.335327	0.121979	0.253578
654	GO_NEGATIVE_REGULATION_OF_TRANSMEMBRANE_TRANSPORT	23	1.333949	0.156915	0.255232
655	GO_T_CELL_DIFFERENTIATION	86	1.333232	0.0913	0.255817
656	GO_NEGATIVE_REGULATION_OF_LEUKOCYTE_DIFFERENTIATION	22	1.332412	0.156627	0.256598
657	GO_POSITIVE_REGULATION_OF_DEPHOSPHORYLATION	17	1.331955	0.152926	0.256831
658	GO_INTERACTION_WITH_SYMBIONT	25	1.33116	0.161917	0.25747
659	GO_POSITIVE_REGULATION_OF_PROTEOLYSIS	109	1.330891	0.090234	0.25742
660	GO_REGULATION_OF_ESTABLISHMENT_OF_PROTEIN_LOCALIZATION_TO_MITOCHONDRION	27	1.330769	0.119845	0.257206
661	GO_PURINE_CONTAINING_COMPOUND_CATABOLIC_PROCESS	19	1.330246	0.15522	0.257553
662	GO_POSITIVE_REGULATION_OF_APOPTOTIC_SIGNALING_PATHWAY	49	1.329994	0.123515	0.257476
663	GO_POSITIVE_REGULATION_OF_ESTABLISHMENT_OF_PROTEIN_LOCALIZATION_TO_MITOCHONDRION	24	1.329197	0.14489	0.258163
664	GO_POSITIVE_REGULATION_OF_DNA_RECOMBINATION	17	1.328778	0.151558	0.258317
665	GO_HOMEOSTASIS_OF_NUMBER_OF_CELLS	81	1.327613	0.113979	0.25955
666	GO_MRNA_PROCESSING	161	1.325932	0.062822	0.261477
667	GO_REGULATION_OF_RESPONSE_TO_ENDOPLASMIC_RETICULUM_STRESS	24	1.32587	0.153342	0.26117
668	GO_HISTONE_METHYLATION	38	1.323948	0.146012	0.2635
669	GO_POSITIVE_REGULATION_OF_RESPONSE_TO_EXTERNAL_STIMULUS	143	1.323894	0.073575	0.263175
670	GO_LYMPHOCYTE_DIFFERENTIATION	105	1.323162	0.089662	0.263879
671	GO_ESTABLISHMENT_OR_MAINTENANCE_OF_CELL_POLARITY	63	1.32292	0.12306	0.263815
672	GO_SNRNA_TRANSCRIPTION	22	1.321472	0.17415	0.265454
673	GO_LEUKOCYTE_HOMEOSTASIS	30	1.319911	0.159033	0.267336
674	GO_RESPONSE_TO_TUMOR_NECROSIS_FACTOR	95	1.319335	0.09806	0.267724
675	GO_NEGATIVE_REGULATION_OF_HEMOPOIESIS	35	1.318951	0.158416	0.267847
676	GO_NEGATIVE_REGULATION_OF_ANTIGEN_RECEPTOR_MEDIATED_SIGNALING_PATHWAY	16	1.318593	0.17934	0.267975
677	GO_CELLULAR_RESPONSE_TO_PEPTIDE_HORMONE_STIMULUS	68	1.317805	0.122942	0.268725
678	GO_ESTABLISHMENT_OF_PROTEIN_LOCALIZATION_TO_ORGANELLE	166	1.316176	0.068507	0.270772
679	GO_REGULATION_OF_CD4_POSITIVE_ALPHA_BETA_T_CELL_ACTIVATION	21	1.315143	0.188329	0.271804
680	GO_REGULATION_OF_CELLULAR_RESPONSE_TO_HEAT	18	1.313482	0.185034	0.273725
681	GO_ALPHA_AMINO_ACID_METABOLIC_PROCESS	59	1.310013	0.143982	0.278405
682	GO_RESPONSE_TO_ORGANOPHOSPHORUS	29	1.30982	0.152913	0.278279
683	GO_ORGANIC_HYDROXY_COMPOUND_BIOSYNTHETIC_PROCESS	72	1.308762	0.120575	0.279446

684	GO_REGULATION_OF_ALPHA_BETA_T_CELL_DIFFERENTIATION	21	1.308191	0.184388	0.279917
685	GO_ORGANELLE_FUSION	29	1.30785	0.175743	0.280046
686	GO_NEGATIVE_REGULATION_OF_PROTEIN_BINDING	30	1.307641	0.160099	0.279909
687	GO_REGULATION_OF_PEPTIDASE_ACTIVITY	110	1.30701	0.101803	0.280478
688	GO_MYELOID_CELL_HOMEOSTASIS	45	1.306865	0.158879	0.28028
689	GO_FATTY_ACID_TRANSPORT	24	1.304877	0.164751	0.282687
690	GO_PROTEIN_PEPTIDYL_PROLYL_ISOMERIZATION	15	1.302583	0.190345	0.28564
691	GO_TRNA_MODIFICATION	17	1.302334	0.191837	0.285626
692	GO_CELL_DEATH_IN_RESPONSE_TO_OXIDATIVE_STRESS	19	1.301082	0.174515	0.287067
693	GO_PEPTIDE_BIOSYNTHETIC_PROCESS	209	1.299707	0.070994	0.288634
694	GO_INTRINSIC_APOPTOTIC_SIGNALING_PATHWAY_BY_P53_CLASS_MEDIATOR	22	1.298594	0.181579	0.289851
695	GO_NUCLEOSIDE_PHOSPHATE_CATABOLIC_PROCESS	30	1.297323	0.170918	0.291311
696	GO_PROTEIN_TRANSMEMBRANE_TRANSPORT	23	1.297012	0.175573	0.291318
697	GO_POSITIVE_REGULATION_OF_MOLECULAR_FUNCTION	490	1.296622	0.013	0.291494
698	GO_DEVELOPMENTAL_MATURATION	67	1.29367	0.140798	0.2955
699	GO_REGULATION_OF_APOPTOTIC_SIGNALING_PATHWAY	102	1.293585	0.116453	0.295223
700	GO_RESPONSE_TO_LIPID	203	1.290849	0.072597	0.298953
701	GO_CELLULAR_RESPONSE_TO_NITROGEN_COMPOUND	157	1.289901	0.085303	0.299987
702	GO_RESPONSE_TO_OXYGEN_CONTAINING_COMPOUND	384	1.289771	0.03507	0.299758
703	GO_RESPONSE_TO_HEAT	41	1.28955	0.176259	0.299694
704	GO_RESPONSE_TO_NITROGEN_COMPOUND	253	1.288217	0.065392	0.301295
705	GO_REGULATION_OF_LYMPHOCYTE_DIFFERENTIATION	58	1.287874	0.150857	0.301395
706	GO_EMBRYO_DEVELOPMENT_ENDING_IN_BIRTH_OR_EGG_HATCHING	160	1.287787	0.08866	0.301113
707	GO_INTERFERON_BETA_PRODUCTION	16	1.28755	0.205163	0.301053
708	GO_AMIDE_BIOSYNTHETIC_PROCESS	246	1.2874	0.047284	0.30087
709	GO_PROTON_TRANSMEMBRANE_TRANSPORT	53	1.28555	0.152326	0.303306
710	GO_HISTONE_DEACETYLATION	16	1.285312	0.18232	0.303252
711	GO_CELLULAR_RESPONSE_TO_OXYGEN_CONTAINING_COMPOUND	279	1.285162	0.038191	0.303064
712	GO_REGULATION_OF_PROTEIN_ACETYLATION	24	1.284604	0.187335	0.30351
713	GO_REACTIVE_NITROGEN_SPECIES_METABOLIC_PROCESS	15	1.28363	0.179916	0.304636
714	GO_TRANSCRIPTION_BY_RNA_POLYMERASE_I	15	1.283267	0.182561	0.304767
715	GO_POSITIVE_REGULATION_OF_CYSTEINE_TYPE_ENDOPEPTIDASE_ACTIVITY	48	1.283053	0.15986	0.304647
716	GO_CELLULAR_RESPONSE_TO_PEPTIDE	92	1.282422	0.127137	0.305117
717	GO_POSITIVE_REGULATION_OF_CATALYTIC_ACTIVITY	408	1.280843	0.025025	0.307122
718	GO_REGULATION_OF_FATTY_ACID_METABOLIC_PROCESS	22	1.27846	0.192661	0.310447
719	GO_GLUTAMINE_FAMILY_AMINO_ACID_METABOLIC_PROCESS	17	1.278188	0.209655	0.310424
720	GO_ESTABLISHMENT_OF_RNA_LOCALIZATION	52	1.277575	0.18583	0.310949
721	GO_POSITIVE_REGULATION_OF_PROTEIN_CATABOLIC_PROCESS	60	1.27685	0.160775	0.311649
722	GO_CELLULAR_ALDEHYDE_METABOLIC_PROCESS	15	1.276095	0.201449	0.312414
723	GO_REGULATION_OF_CELLULAR_PROTEIN_LOCALIZATION	164	1.275739	0.095679	0.312539
724	GO_REGULATION_OF_PROTEASOMAL_UBIQUITIN_DEPENDENT_PROTEIN_CATABOLIC_PROCESS	48	1.275669	0.163146	0.312211
725	GO_CD4_POSITIVE_ALPHA_BETA_T_CELL_ACTIVATION	33	1.275284	0.188119	0.312344
726	GO_REGULATION_OF_PROTEIN_CATABOLIC_PROCESS	118	1.273489	0.12382	0.314824
727	GO_NIK_NF_KAPPAB_SIGNALING	70	1.270075	0.163697	0.319897

728	GO_SMALL_GTPASE_MEDIATED_SIGNAL_TRANSDUCTION	143	1.269992	0.120743	0.319621
729	GO_RESPONSE_TO_INTERLEUKIN_12	16	1.267433	0.206128	0.323304
730	GO_REGULATION_OF_MITOCHONDRIAL_MEMBRANE_POTENTIAL	24	1.266176	0.196286	0.324938
731	GO_REGULATION_OF_T_CELL_ACTIVATION	110	1.264696	0.143611	0.326828
732	GO_REGULATION_OF_POSTTRANSCRIPTIONAL_GENE_SILENCING	21	1.264283	0.207792	0.327055
733	GO_RIBONUCLEOSIDE_MONOPHOSPHATE_METABOLIC_PROCESS	23	1.263808	0.213072	0.327373
734	GO_POSITIVE_REGULATION_OF_CYTOKINE_PRODUCTION	123	1.256084	0.129843	0.339747
735	GO_NEGATIVE_REGULATION_OF_TRANSCRIPTION_BY_RNA_POLYMERAS E_II	242	1.25593	0.080564	0.339533
736	GO_STRIATED_MUSCLE_CELL_PROLIFERATION	18	1.255479	0.223776	0.339794
737	GO_NEGATIVE_REGULATION_OF_CYTOSKELETON_ORGANIZATION	44	1.25469	0.204413	0.340579
738	GO_PEPTIDYL_LYSINE_ACETYLATION	53	1.253775	0.190972	0.34164
739	GO_MITOCHONDRIAL_TRANSMEMBRANE_TRANSPORT	41	1.252917	0.192261	0.342534
740	GO_ATP_BIOSYNTHETIC_PROCESS	25	1.252399	0.206329	0.342905
741	GO_POSITIVE_REGULATION_OF_ADAPTIVE_IMMUNE_RESPONSE	35	1.252101	0.212531	0.342944
742	GO_REGULATION_OF_CATABOLIC_PROCESS	279	1.249905	0.075301	0.346115
743	GO_PROTEIN_ACYLATION	73	1.249106	0.176275	0.346947
744	GO_RESPONSE_TO_XENOBIOTIC_STIMULUS	28	1.249008	0.219822	0.346648
745	GO_POSITIVE_REGULATION_OF_NUCLEOBASE_CONTAINING_COMPOUN D_METABOLIC_PROCESS	457	1.248298	0.043043	0.347419
746	GO_ORGANELLE_DISASSEMBLY	31	1.244771	0.236453	0.352891
747	GO_ORGANOPHOSPHATE_BIOSYNTHETIC_PROCESS	174	1.242926	0.122574	0.355594
748	GO_REGULATION_OF_ERYTHROCYTE_DIFFERENTIATION	18	1.24155	0.236878	0.357438
749	GO_REGULATION_OF_CELLULAR_PROTEIN_CATABOLIC_PROCESS	81	1.241026	0.188865	0.357897
750	GO_EXTRINSIC_APOPTOTIC_SIGNALING_PATHWAY_VIA_DEATH_DOMAI N_RECEPTORS	23	1.239551	0.214756	0.359952
751	GO_REGULATORY_T_CELL_DIFFERENTIATION	19	1.233712	0.228454	0.369548
752	GO_RIBONUCLEOPROTEIN_COMPLEX_SUBUNIT_ORGANIZATION	64	1.231908	0.188534	0.372144
753	GO_POSITIVE_REGULATION_OF_INTRINSIC_APOPTOTIC_SIGNALING_PAT HWAY	19	1.230047	0.234043	0.37492
754	GO_RESPONSE_TO_TOPOLOGICALLY_INCORRECT_PROTEIN	50	1.229939	0.197661	0.374607
755	GO_POSITIVE_REGULATION_OF_RESPONSE_TO_CYTOKINE_STIMULUS	17	1.229072	0.22807	0.375692
756	GO_PEPTIDYL_LYSINE_MODIFICATION	113	1.226036	0.187696	0.380583
757	GO_LYMPHOCYTE_COSTIMULATION	20	1.225942	0.237598	0.380251
758	GO_HISTONE_H4_ACETYLATION	26	1.224853	0.23375	0.381697
759	GO_RESPONSE_TO_LEUKEMIA_INHIBITORY_FACTOR	26	1.22354	0.252845	0.383539
760	GO_CYTOSOLIC_CALCIUM_ION_TRANSPORT	43	1.222935	0.206273	0.384034
761	GO_PROTEIN_METHYLATION	48	1.222172	0.219833	0.384896
762	GO_RESPONSE_TO_AXON_INJURY	15	1.221502	0.251429	0.38557
763	GO_REGULATION_OF_MITOCHONDRIAL_GENE_EXPRESSION	15	1.221032	0.23913	0.385867
764	GO_PEPTIDYL_TYROSINE_DEPHOSPHORYLATION	33	1.220249	0.236613	0.386748
765	GO_RESPONSE_TO_CORTICOSTEROID	31	1.219369	0.242462	0.387714
766	GO_INTERACTION_WITH_HOST	59	1.217789	0.211251	0.39006
767	GO_REGULATION_OF_PROTEIN_STABILITY	82	1.217037	0.195749	0.390864
768	GO_REGULATION_OF_INTRACELLULAR_PROTEIN_TRANSPORT	89	1.215395	0.183896	0.393344
769	GO_REGULATION_OF_ANTIGEN_RECEPTOR_MEDIATED_SIGNALING_PAT HWAY	32	1.211371	0.240695	0.400139
770	GO_REGULATION_OF_PROTEIN_MODIFICATION_PROCESS	498	1.210445	0.052	0.40139

771	GO_NAD_METABOLIC_PROCESS	19	1.209731	0.244681	0.402157
772	GO_T_CELL_HOMEOSTASIS	17	1.20934	0.257453	0.402293
773	GO_LEUKOCYTE_DIFFERENTIATION	139	1.208054	0.197531	0.404012
774	GO_MRNA_3_END_PROCESSING	33	1.207609	0.258142	0.404298
775	GO_ORGANIC_ACID_METABOLIC_PROCESS	323	1.206309	0.086086	0.40623
776	GO_REGULATION_OF_HISTONE_MODIFICATION	40	1.206102	0.244947	0.40609
777	GO_CELLULAR_RESPONSE_TO_MECHANICAL_STIMULUS	17	1.205733	0.248968	0.406233
778	GO_DEVELOPMENT_OF_PRIMARY_FEMALE_SEXUAL_CHARACTERISTICS	22	1.205402	0.25298	0.406314
779	GO_REGULATION_OF_UBIQUITIN_DEPENDENT_PROTEIN_CATABOLIC_PROCESS	58	1.205009	0.231293	0.406548
780	GO_REGULATION_OF_PROTEOLYSIS	192	1.204457	0.140816	0.407053
781	GO_LIMBIC_SYSTEM_DEVELOPMENT	27	1.202908	0.255725	0.409323
782	GO_REGULATION_OF_ALCOHOL_BIOSYNTHETIC_PROCESS	21	1.201429	0.265333	0.411533
783	GO_RESPONSE_TO_PEPTIDE	118	1.201428	0.209593	0.411009
784	GO_POSITIVE_REGULATION_OF_NEURON_DEATH	31	1.201395	0.25062	0.410556
785	GO_SMOOTHENED_SIGNALING_PATHWAY	36	1.201336	0.25	0.410145
786	GO_NUCLEOBASE_CONTAINING_COMPOUND_TRANSPORT	62	1.200054	0.238522	0.411958
787	GO_POSITIVE_REGULATION_OF_ALPHA_BETA_T_CELL_ACTIVATION	26	1.199148	0.252564	0.413119
788	GO_REGULATION_OF_LIPID_BIOSYNTHETIC_PROCESS	48	1.198731	0.245018	0.413351
789	GO_INTERLEUKIN_10_PRODUCTION	17	1.19631	0.247191	0.417284
790	GO_REGULATION_OF_EPITHELIAL_CELL_DIFFERENTIATION	28	1.196289	0.257822	0.416792
791	GO_REGULATION_OF_CELL_KILLING	29	1.195596	0.265725	0.417552
792	GO_CELLULAR_RESPONSE_TO_BIOTIC_STIMULUS	60	1.19404	0.245434	0.419835
793	GO_EMBRYO_DEVELOPMENT	223	1.193683	0.139394	0.419989
794	GO_POSITIVE_REGULATION_OF_VIRAL_PROCESS	42	1.193184	0.246988	0.420385
795	GO_REGULATION_OF_INTRACELLULAR_TRANSPORT	107	1.192329	0.205319	0.421409
796	GO_NEGATIVE_REGULATION_OF_EXTRINSIC_APOPTOTIC_SIGNALING_PATHWAY	23	1.191475	0.27947	0.422455
797	GO_MUSCLE_CELL_APOPTOTIC_PROCESS	15	1.190431	0.2879	0.423792
798	GO_REGULATION_OF_CARDIAC_MUSCLE_TISSUE_DEVELOPMENT	22	1.190392	0.27086	0.423341
799	GO_POSITIVE_REGULATION_OF_CELLULAR_PROTEIN_CATABOLIC_PROCESS	47	1.190375	0.252656	0.422849
800	GO_REGULATION_OF_CELLULAR_CATABOLIC_PROCESS	241	1.187267	0.166667	0.428032
801	GO_EMBRYONIC_ORGAN_DEVELOPMENT	77	1.186197	0.220377	0.42954
802	GO_PROTEIN_IMPORT	55	1.185678	0.255973	0.429994
803	GO_INFLAMMATORY_RESPONSE_TO_ANTIGENIC_STIMULUS	16	1.184045	0.280576	0.43264
804	GO_POSITIVE_REGULATION_OF_CELLULAR_BIOSYNTHETIC_PROCESS	471	1.18395	0.102102	0.432263
805	GO_PROTEIN_AUTOPHOSPHORYLATION	62	1.183099	0.253333	0.433267
806	GO_HISTONE_H3_K9_MODIFICATION	15	1.181927	0.262178	0.434941
807	GO_PROTEIN_STABILIZATION	54	1.181853	0.247674	0.434565
808	GO_CARBOHYDRATE_DERIVATIVE_BIOSYNTHETIC_PROCESS	194	1.181822	0.160081	0.434092
809	GO_POSITIVE_REGULATION_OF_INTRACELLULAR_PROTEIN_TRANSPORT	62	1.181168	0.249425	0.434787
810	GO_POSITIVE_REGULATION_OF_MRNA_METABOLIC_PROCESS	19	1.180423	0.263374	0.435614
811	GO_REGULATION_OF_CD4_POSITIVE_ALPHA_BETA_T_CELL_DIFFERENTIATION	16	1.179698	0.269437	0.436401
812	GO_PROTEIN_CONTAINING_COMPLEX_LOCALIZATION	84	1.179275	0.236443	0.436664
813	GO_HOMEOSTATIC_PROCESS	487	1.179193	0.093	0.436261

814	GO_OXIDATION_REDUCTION_PROCESS	323	1.177875	0.139139	0.438266
815	GO_REGULATION_OF_PHOSPHORYLATION	424	1.175586	0.117	0.442085
816	GO_CELLULAR_RESPONSE_TO_TOPOLOGICALLY_INCORRECT_PROTEIN	39	1.174385	0.273165	0.443861
817	GO_POSITIVE_REGULATION_OF_UBIQUITIN_DEPENDENT_PROTEIN_CATABOLIC_PROCESS	37	1.174353	0.272727	0.443382
818	GO_NATURAL_KILLER_CELL_ACTIVATION	16	1.174284	0.289835	0.442976
819	GO_NEGATIVE_REGULATION_OF_WNT_SIGNALING_PATHWAY	64	1.172936	0.25395	0.445026
820	GO_REGULATION_OF_MUSCLE_ORGAN_DEVELOPMENT	35	1.17203	0.264563	0.446211
821	GO_POSITIVE_REGULATION_OF_INTRACELLULAR_TRANSPORT	72	1.170464	0.252548	0.448649
822	GO_EAR_DEVELOPMENT	37	1.170331	0.266187	0.448337
823	GO_POLYOL_BIOSYNTHETIC_PROCESS	21	1.170051	0.301351	0.448331
824	GO_POSITIVE_REGULATION_OF_MAP_KINASE_ACTIVITY	56	1.169151	0.272727	0.44945
825	GO_ACTIVATION_OF_CYSTEINE_TYPE_ENDOPEPTIDASE_ACTIVITY_INVOLVED_IN_APOPTOTIC_PROCESS	31	1.168275	0.28801	0.450565
826	GO_INTRINSIC_APOPTOTIC_SIGNALING_PATHWAY_IN_RESPONSE_TO_ENDOPLASMIC_RETICULUM_STRESS	22	1.165119	0.272483	0.456191
827	GO_POSITIVE_REGULATION_OF_CELL_DEATH	199	1.164582	0.191641	0.456702
828	GO_STEM_CELL_DIFFERENTIATION	77	1.163502	0.282895	0.458245
829	GO_RESPONSE_TO_HORMONE	198	1.163272	0.206333	0.458135
830	GO_ESTABLISHMENT_OF_CELL_POLARITY	41	1.16225	0.267689	0.45954
831	GO_REGULATION_OF_INTRINSIC_APOPTOTIC_SIGNALING_PATHWAY	41	1.16205	0.285024	0.459364
832	GO_RESPONSE_TO_PEPTIDE_HORMONE	92	1.161976	0.254386	0.458965
833	GO_TELENCEPHALON_DEVELOPMENT	60	1.161973	0.266819	0.45842
834	GO_REGULATION_OF_SMOOTHENED_SIGNALING_PATHWAY	18	1.161833	0.312329	0.458125
835	GO_REGULATION_OF_LEUKOCYTE_DIFFERENTIATION	78	1.161807	0.266234	0.457617
836	GO_SMALL_MOLECULE_CATABOLIC_PROCESS	134	1.161234	0.227702	0.458126
837	GO_POSITIVE_REGULATION_OF_PROTEOLYSIS_INVOLVED_IN_CELLULAR_PROTEIN_CATABOLIC_PROCESS	44	1.159489	0.298204	0.460962
838	GO_T_CELL_MEDIATED_CYTOTOXICITY	17	1.155374	0.300268	0.46841
839	GO_ALPHA_BETA_T_CELL_DIFFERENTIATION	35	1.153592	0.301471	0.471311
840	GO_ACTOMYOSIN_STRUCTURE_ORGANIZATION	49	1.152484	0.290822	0.472919
841	GO_POSITIVE_REGULATION_OF_ATP_METABOLIC_PROCESS	16	1.152029	0.299584	0.473242
842	GO_REGULATION_OF_IMMUNOGLOBULIN_PRODUCTION	21	1.151564	0.30274	0.473587
843	GO_FATTY_ACID_BIOSYNTHETIC_PROCESS	38	1.150626	0.304878	0.474848
844	GO_REGULATION_OF_GLUCOSE_TRANSMEMBRANE_TRANSPORT	15	1.149558	0.319728	0.476397
845	GO_STEROID_BIOSYNTHETIC_PROCESS	53	1.145343	0.315668	0.484135
846	GO_REGULATION_OF_RESPONSE_TO_CYTOKINE_STIMULUS	48	1.145257	0.305164	0.483736
847	GO_INTERLEUKIN_1_PRODUCTION	23	1.14444	0.308399	0.484799
848	GO_SIGNAL_TRANSDUCTION_IN_ABSENCE_OF_LIGAND	19	1.144394	0.320442	0.484324
849	GO_CELLULAR_RESPONSE_TO_ORGANIC_CYCLIC_COMPOUND	117	1.143572	0.267159	0.485392
850	GO_IMPORT_INTO_CELL	45	1.14063	0.307143	0.490694
851	GO_CELLULAR_RESPONSE_TO_LIPID	128	1.140467	0.294858	0.490458
852	GO_CARBOHYDRATE_DERIVATIVE_METABOLIC_PROCESS	311	1.139966	0.222334	0.490908
853	GO_POSITIVE_REGULATION_OF_PATTERN_RECOGNITION_RECEPTOR_SIGNALING_PATHWAY	17	1.138635	0.318373	0.493088
854	GO_CD4_POSITIVE_ALPHA_BETA_T_CELL_DIFFERENTIATION	25	1.138355	0.322368	0.493079
855	GO_AMINOGLYCAN_METABOLIC_PROCESS	34	1.138327	0.31744	0.492556
856	GO_REGULATION_OF_GENERATION_OF_PRECURSOR_METABOLITES_AND_ENERGY	46	1.137467	0.311534	0.493673

857	GO_NEGATIVE_REGULATION_OF_CELL_DEATH	238	1.13711	0.243629	0.493812
858	GO_NEGATIVE_REGULATION_OF_MYELOID_CELL_DIFFERENTIATION	22	1.135568	0.330214	0.496229
859	GO_REGULATION_OF_CELL_DEATH	435	1.135243	0.194194	0.496302
860	GO_CELLULAR_OXIDANT_DETOXIFICATION	29	1.135231	0.323194	0.495755
861	GO_NEGATIVE_REGULATION_OF_MOLECULAR_FUNCTION	271	1.135215	0.237134	0.495205
862	GO_PIGMENT_BIOSYNTHETIC_PROCESS	20	1.134868	0.328823	0.495311
863	GO_CELLULAR_SENESCENCE	27	1.132817	0.322785	0.498798
864	GO_INTRACILIARY_TRANSPORT	19	1.132707	0.313699	0.498446
865	GO_MRNA_TRANSPORT	36	1.132093	0.312727	0.499072
866	GO_REGULATION_OF_PHOSPHORUS_METABOLIC_PROCESS	477	1.131573	0.176176	0.499564
867	GO_MAINTENANCE_OF_LOCATION_IN_CELL	55	1.130096	0.308126	0.501998
868	GO_MODULATION_OF_PROCESS_OF_OTHER_ORGANISM	34	1.12912	0.333745	0.503343
869	GO_RESPONSE_TO_METAL_ION	93	1.127339	0.297826	0.506281
870	GO_INTERLEUKIN_2_PRODUCTION	18	1.127297	0.342282	0.505789
871	GO_MUCOPOLYSACCHARIDE_METABOLIC_PROCESS	24	1.12721	0.326343	0.505384
872	GO_PEPTIDE_METABOLIC_PROCESS	248	1.124485	0.248741	0.510338
873	GO_PALLIUM_DEVELOPMENT	49	1.124301	0.329327	0.510142
874	GO_GROWTH	227	1.121711	0.264706	0.514791
875	GO_REGULATION_OF_HEART_GROWTH	20	1.119499	0.332468	0.518635
876	GO_CELLULAR_AMIDE_METABOLIC_PROCESS	325	1.119438	0.211055	0.518167
877	GO_AUTOPHAGY_OF_MITOCHONDRION	21	1.118952	0.337398	0.518575
878	GO_REGULATION_OF_PRODUCTION_OF_MOLECULAR_MEDIATOR_OF_I MMUNE_RESPONSE	39	1.117987	0.32801	0.519942
879	GO_EPITHELIUM_DEVELOPMENT	263	1.114452	0.250752	0.526564
880	GO_CALCIUM_ION_TRANSMEMBRANE_IMPORT_INTO_CYTOSOL	33	1.113942	0.351351	0.527013
881	GO_CELLULAR_RESPONSE_TO_MOLECULE_OF_BACTERIAL_ORIGIN	53	1.113868	0.326554	0.526563
882	GO_ORGANONITROGEN_COMPOUND_BIOSYNTHETIC_PROCESS	499	1.111975	0.202202	0.529821
883	GO_INTERLEUKIN_8_PRODUCTION	17	1.111327	0.357043	0.530506
884	GO_NEGATIVE_REGULATION_OF_CELL_POPULATION_PROLIFERATION	181	1.11124	0.298583	0.530083
885	GO_ALPHA_BETA_T_CELL_ACTIVATION_INVOLVED_IN_IMMUNE_RESPO NSE	19	1.11102	0.357634	0.529948
886	GO_POSITIVE_REGULATION_OF_PROTEIN_MODIFICATION_PROCESS	305	1.109899	0.264794	0.531684
887	GO_SMOOTH_MUSCLE_CONTRACTION	18	1.109493	0.367072	0.531909
888	GO_REGULATION_OF_EXTRINSIC_APOPTOTIC_SIGNALING_PATHWAY	41	1.109231	0.361416	0.531853
889	GO_GOLGI_VESICLE_TRANSPORT	88	1.109104	0.335129	0.531533
890	GO_PROTEIN_POLYMERIZATION	79	1.108823	0.331162	0.531502
891	GO_PEROXISOME_ORGANIZATION	16	1.107228	0.353909	0.534193
892	GO_POSITIVE_REGULATION_OF_LEUKOCYTE_CELL_CELL_ADHESION	75	1.105307	0.350706	0.537563
893	GO_REGULATION_OF_DEPHOSPHORYLATION	63	1.104337	0.349493	0.53888
894	GO_REGULATION_OF_LEUKOCYTE_MEDIATED_CYTOTOXICITY	22	1.103787	0.381078	0.539329
895	GO_REGULATION_OF_RESPONSE_TO_EXTERNAL_STIMULUS	264	1.103676	0.292879	0.538962
896	GO_POSITIVE_REGULATION_OF_LEUKOCYTE_PROLIFERATION	50	1.102911	0.356725	0.539923
897	GO_B_CELL_PROLIFERATION	32	1.102696	0.351418	0.539718
898	GO_REGULATION_OF_ATPASE_ACTIVITY	27	1.101361	0.346348	0.541805
899	GO_AXIS_SPECIFICATION	18	1.100973	0.364372	0.541978
900	GO_NEGATIVE_REGULATION_OF_APOPTOTIC_SIGNALING_PATHWAY	53	1.100208	0.347625	0.542877

901	GO_MODULATION_BY_HOST_OF_SYMBIONT_PROCESS	18	1.100011	0.366022	0.542666
902	GO_MACROMOLECULE_DEACYLATION	27	1.099089	0.355844	0.543985
903	GO_T_CELL_DIFFERENTIATION_INVOLVED_IN_IMMUNE_RESPONSE	20	1.095093	0.371202	0.551543
904	GO_REGULATION_OF_TOLL_LIKE_RECEPTOR_SIGNALING_PATHWAY	24	1.094654	0.371578	0.551841
905	GO_PRODUCTION_OF_SMALL_RNA_INVOLVED_IN_GENE_SILENCING_BY_RNA	16	1.094135	0.364486	0.552244
906	GO_REGULATION_OF_CELLULAR_LOCALIZATION	266	1.09318	0.311871	0.553592
907	GO_PROTEIN_SUMOYLATION	17	1.093151	0.372928	0.553039
908	GO_TEMPERATURE_HOMEOSTASIS	37	1.092851	0.354399	0.553036
909	GO_POSITIVE_REGULATION_OF_PROTEIN_METABOLIC_PROCESS	440	1.09141	0.259259	0.555355
910	GO_PURINE_NUCLEOSIDE_MONOPHOSPHATE_METABOLIC_PROCESS	19	1.089432	0.381333	0.558723
911	GO_VASCULAR_PROCESS_IN_CIRCULATORY_SYSTEM	32	1.088234	0.388206	0.560646
912	GO_REGULATION_OF_STEROID_BIOSYNTHETIC_PROCESS	23	1.087645	0.38189	0.561238
913	GO_ORGANOPHOSPHATE_CATABOLIC_PROCESS	44	1.084328	0.379023	0.567412
914	GO_POSITIVE_REGULATION_OF_CALCIUM_ION_TRANSPORT	22	1.083665	0.376774	0.568124
915	GO_WATER_HOMEOSTASIS	15	1.082095	0.391304	0.570697
916	GO_FATTY_ACID_BETA_OXIDATION	25	1.081953	0.39433	0.570369
917	GO_NUCLEOSIDE_BISPHOSPHATE_BIOSYNTHETIC_PROCESS	21	1.081394	0.360215	0.570867
918	GO_REGULATION_OF_HISTONE_METHYLATION	18	1.080212	0.379642	0.572651
919	GO_BODY_FLUID_SECRETION	22	1.078571	0.380449	0.575409
920	GO_REGULATION_OF_MRNA_PROCESSING	36	1.077695	0.389937	0.576536
921	GO_CYTOKINE_PRODUCTION_INVOLVED_IN_IMMUNE_RESPONSE	27	1.074318	0.404822	0.582944
922	GO_CELLULAR_RESPONSE_TO_TOXIC_SUBSTANCE	32	1.073593	0.384906	0.58377
923	GO_TRANSCRIPTION_INITIATION_FROM_RNA_POLYMERASE_II_PROMOTER	55	1.073474	0.387543	0.5834
924	GO_NEGATIVE_REGULATION_OF_PROTEASOMAL_PROTEIN_CATABOLIC_PROCESS	17	1.070799	0.38843	0.588296
925	GO_NEGATIVE_REGULATION_OF_CATALYTIC_ACTIVITY	197	1.0707	0.351215	0.58786
926	GO_ENTRY_INTO_HOST	36	1.070538	0.371747	0.587557
927	GO_REGULATION_OF_ORGAN_GROWTH	27	1.068003	0.395172	0.592295
928	GO_ADAPTIVE_THERMOGENESIS	34	1.067336	0.39467	0.593009
929	GO_NEGATIVE_REGULATION_OF_MAPK_CASCADE	46	1.065669	0.390845	0.595768
930	GO_CELLULAR_RESPONSE_TO_ALCOHOL	24	1.064649	0.413072	0.597272
931	GO_ENDOPLASMIC_RETICULUM_UNFOLDED_PROTEIN_RESPONSE	31	1.063729	0.400996	0.598553
932	GO_REGULATION_OF_EMBRYONIC_DEVELOPMENT	18	1.063392	0.411928	0.598583
933	GO_REGULATION_OF_PROTEIN_LOCALIZATION	279	1.06218	0.372608	0.600481
934	GO_RESPONSE_TO_CHEMOKINE	25	1.062123	0.395674	0.599953
935	GO_NEGATIVE_REGULATION_OF_LYMPHOCYTE_ACTIVATION	41	1.06003	0.402353	0.603622
936	GO_PEPTIDYL_AMINO_ACID_MODIFICATION	354	1.058976	0.358	0.605182
937	GO_ACTIVATED_T_CELL_PROLIFERATION	20	1.058448	0.382924	0.6056
938	GO_CELL_ACTIVATION_INVOLVED_IN_IMMUNE_RESPONSE	173	1.058178	0.388776	0.605535
939	GO_SULFUR_COMPOUND_METABOLIC_PROCESS	99	1.058003	0.40836	0.605249
940	GO_REGULATION_OF_ANIMAL_ORGAN_MORPHOGENESIS	70	1.056671	0.398883	0.607295
941	GO_POSITIVE_REGULATION_OF_CELLULAR_COMPONENT_ORGANIZATION	325	1.056664	0.367203	0.606667
942	GO_CARDIAC_CELL_DEVELOPMENT	18	1.054555	0.425648	0.610334
943	GO_SEQUESTERING_OF_CALCIUM_ION	31	1.054141	0.413317	0.610523

944	GO_PEROXISOMAL_TRANSPORT	15	1.053474	0.408392	0.611245
945	GO_RESPONSE_TO_ESTROGEN	15	1.052743	0.421125	0.612179
946	GO_VIRION_ASSEMBLY	16	1.05231	0.417127	0.612443
947	GO_INTERLEUKIN_1_BETA_PRODUCTION	21	1.05141	0.402344	0.613697
948	GO_NEGATIVE_REGULATION_OF_MRNA_METABOLIC_PROCESS	23	1.051378	0.432778	0.61312
949	GO_RESPONSE_TO_TEMPERATURE_STIMULUS	52	1.05074	0.390909	0.61379
950	GO_NEGATIVE_REGULATION_OF_DEPHOSPHORYLATION	34	1.047836	0.424505	0.619097
951	GO_EPITHELIAL_CELL_DIFFERENTIATION	125	1.04755	0.416233	0.61906
952	GO_HEART_GROWTH	23	1.045955	0.40129	0.621798
953	GO_DNA_TEMPLATED_TRANSCRIPTION_INITIATION	62	1.045137	0.407745	0.622809
954	GO_NEGATIVE_REGULATION_OF_ION_TRANSMEMBRANE_TRANSPORT	20	1.045059	0.419737	0.622303
955	GO_POSITIVE_REGULATION_OF_CHROMATIN_ORGANIZATION	28	1.044532	0.428571	0.622731
956	GO_PIGMENT_METABOLIC_PROCESS	23	1.043687	0.428202	0.623857
957	GO_LEUKOCYTE_CELL_CELL_ADHESION	102	1.042113	0.428419	0.62647
958	GO_REGULATION_OF_ADAPTIVE_IMMUNE_RESPONSE	52	1.041498	0.409611	0.627072
959	GO_ERYTHROCYTE_HOMEOSTASIS	36	1.041313	0.423899	0.626826
960	GO_RIBOSOMAL_SMALL_SUBUNIT_BIOGENESIS	22	1.040501	0.416337	0.627865
961	GO_POSITIVE_REGULATION_OF_RNA_BIOSYNTHETIC_PROCESS	370	1.040349	0.386	0.627528
962	GO_FOREBRAIN_DEVELOPMENT	94	1.035488	0.425214	0.637053
963	GO_MORPHOGENESIS_OF_AN_EPITHELIUM	132	1.033917	0.435467	0.639638
964	GO_REGULATION_OF_PROTEIN_TARGETING_TO_MITOCHONDRION	15	1.03382	0.430168	0.639176
965	GO_CELLULAR_LIPID_CATABOLIC_PROCESS	52	1.03293	0.424138	0.640352
966	GO_REGULATION_OF_CELLULAR_AMIDE_METABOLIC_PROCESS	117	1.03209	0.42736	0.641407
967	GO_EMBRYONIC_CRANIAL_SKELETON_MORPHOGENESIS	15	1.031484	0.431694	0.642004
968	GO_REGULATION_OF_DNA_BINDING_TRANSCRIPTION_FACTOR_ACTIVITY	108	1.029094	0.439746	0.646247
969	GO_CELLULAR_RESPONSE_TO_EXTERNAL_STIMULUS	67	1.02847	0.433898	0.646834
970	GO_NEGATIVE_REGULATION_OF_PROTEIN_CATABOLIC_PROCESS	42	1.027957	0.431655	0.647198
971	GO_POSITIVE_REGULATION_OF_CATABOLIC_PROCESS	111	1.027803	0.448568	0.646836
972	GO_CELLULAR_HOMEOSTASIS	245	1.025631	0.46371	0.650628
973	GO_EMBRYONIC_SKELETAL_SYSTEM_MORPHOGENESIS	20	1.025047	0.433198	0.651159
974	GO_CEREBRAL_CORTEX_DEVELOPMENT	39	1.024655	0.452229	0.651302
975	GO_POSITIVE_REGULATION_OF_CD4_POSITIVE_ALPHA_BETA_T_CELL_ACTIVATION	15	1.022863	0.454545	0.654367
976	GO_REGULATION_OF_MEGAKARYOCYTE_DIFFERENTIATION	21	1.021885	0.453473	0.655705
977	GO_MONOCARBOXYLIC_ACID_METABOLIC_PROCESS	165	1.021616	0.466598	0.655565
978	GO_MONOCARBOXYLIC_ACID_TRANSPORT	39	1.021523	0.45	0.655073
979	GO_POSITIVE_REGULATION_OF_INTERFERON_GAMMA_PRODUCTION	20	1.020712	0.439894	0.656098
980	GO_REGULATION_OF_PROTEIN_CONTAINING_COMPLEX_ASSEMBLY	124	1.017937	0.453305	0.66099
981	GO_CARDIOCYTE_DIFFERENTIATION	27	1.015964	0.458607	0.664392
982	GO_DEVELOPMENTAL_GROWTH	147	1.012862	0.480208	0.669992
983	GO_REGULATION_OF_PATTERN_RECOGNITION_RECEPTOR_SIGNALING_PATHWAY	31	1.012353	0.449814	0.670307
984	GO_NEUTRAL_LIPID_METABOLIC_PROCESS	22	1.011351	0.457364	0.671619
985	GO_NEGATIVE_REGULATION_OF_HYDROLASE_ACTIVITY	110	1.011255	0.472103	0.671125
986	GO_POSITIVE_REGULATION_OF_REACTIVE_OXYGEN_SPECIES_METABOLIC_PROCESS	18	1.00967	0.468	0.673716

987	GO_NEGATIVE_REGULATION_OF_PROTEIN_METABOLIC_PROCESS	293	1.007393	0.485944	0.677797
988	GO_CELLULAR_RESPONSE_TO_VIRUS	20	1.007299	0.460338	0.677298
989	GO_MONOSACCHARIDE_BIOSYNTHETIC_PROCESS	25	1.005575	0.463824	0.680149
990	GO_MEMBRANE_FUSION	35	1.004311	0.468402	0.681996
991	GO_LEUKOCYTE_PROLIFERATION	100	1.003176	0.486373	0.683606
992	GO_SPROUTING_ANGIOGENESIS	31	1.00315	0.462299	0.682969
993	GO_POSITIVE_REGULATION_OF_GLIOGENESIS	17	1.001224	0.466292	0.686218
994	GO_REGULATION_OF_TUMOR_NECROSIS_FACTOR_MEDIATED_SIGNALING_PATHWAY	15	0.999976	0.475177	0.688033
995	GO_T_CELL_ACTIVATION_INVOLVED_IN_IMMUNE_RESPONSE	30	0.999519	0.460859	0.688296
996	GO_CILIUM_ORGANIZATION	114	0.999105	0.5	0.688441
997	GO_POSITIVE_REGULATION_OF_PROTEIN_CONTAINING_COMPLEX_ASSEMBLY	75	0.998784	0.484513	0.688403
998	GO_VASCULOGENESIS	15	0.993312	0.472067	0.699
999	GO_THIOESTER_BIOSYNTHETIC_PROCESS	15	0.993071	0.467391	0.698809
1000	GO_PROTEIN_DEPHOSPHORYLATION	101	0.992254	0.509636	0.69981
1001	GO_REGULATION_OF_PHOSPHATASE_ACTIVITY	52	0.991376	0.487973	0.700983
1002	GO_COLUMNAR_CUBOIDAL_EPITHELIAL_CELL_DIFFERENTIATION	22	0.990786	0.475871	0.701472
1003	GO_POSITIVE_REGULATION_OF_ATPASE_ACTIVITY	19	0.990753	0.494398	0.700845
1004	GO_REGULATION_OF_INFLAMMATORY_RESPONSE	76	0.988315	0.49725	0.704999
1005	GO_NEGATIVE_REGULATION_OF_INTRACELLULAR_PROTEIN_TRANSPORT	15	0.98626	0.50511	0.708436
1006	GO_NEURON_APOPTOTIC_PROCESS	70	0.985814	0.5	0.708647
1007	GO_REGULATION_OF_PROTEIN_BINDING	62	0.984108	0.505039	0.711491
1008	GO_PURINE_CONTAINING_COMPOUND_METABOLIC_PROCESS	147	0.98324	0.513458	0.712552
1009	GO_CELL_REDOX_HOMEOSTASIS	25	0.977591	0.5	0.723257
1010	GO_NEGATIVE_REGULATION_OF_TYPE_I_INTERFERON_PRODUCTION	19	0.977265	0.499338	0.723202
1011	GO_REGULATION_OF_T_CELL_MEDIATED_IMMUNITY	24	0.976544	0.514357	0.723918
1012	GO_NEGATIVE_REGULATION_OF_LEUKOCYTE_CELL_CELL_ADHESION	33	0.974301	0.524406	0.727765
1013	GO_REGULATION_OF_CALCIUM_ION_TRANSPORT_INTO_CYTOSOL	25	0.973502	0.509804	0.728685
1014	GO_REGULATION_OF_B_CELL_MEDIATED_IMMUNITY	16	0.973479	0.506224	0.728006
1015	GO_FATTY_ACID_METABOLIC_PROCESS	98	0.972785	0.536585	0.728649
1016	GO_POSITIVE_REGULATION_OF_MUSCLE_TISSUE_DEVELOPMENT	18	0.97278	0.503989	0.727941
1017	GO_CELL_MOTILITY	448	0.972759	0.581162	0.727259
1018	GO_REGULATION_OF_LEUKOCYTE_MEDIATED_IMMUNITY	61	0.971635	0.516534	0.728844
1019	GO_POSITIVE_REGULATION_OF_T_CELL_MEDIATED_IMMUNITY	18	0.971274	0.492898	0.728857
1020	GO_TUBE_DEVELOPMENT	231	0.970665	0.560729	0.729377
1021	GO_REGULATION_OF_PEPTIDYL_THREONINE_PHOSPHORYLATION	15	0.968245	0.515193	0.733542
1022	GO_REGULATION_OF_MAP_KINASE_ACTIVITY	81	0.967347	0.539891	0.734616
1023	GO_POSITIVE_REGULATION_OF_PHOSPHORUS_METABOLIC_PROCESS	284	0.966621	0.569277	0.735358
1024	GO_HEAD_DEVELOPMENT	191	0.965858	0.560606	0.736156
1025	GO_RESPONSE_TO_EXTRACELLULAR_STIMULUS	99	0.965542	0.526882	0.736049
1026	GO_POSITIVE_REGULATION_OF_ESTABLISHMENT_OF_PROTEIN_LOCALIZATION	120	0.963636	0.56341	0.739092
1027	GO_POTASSIUM_ION_TRANSPORT	40	0.96289	0.517857	0.739827
1028	GO_LIPID_OXIDATION	32	0.962883	0.529486	0.739131
1029	GO_NEGATIVE_REGULATION_OF_PROTEIN_SERINE_THREONINE_KINASE_ACTIVITY	40	0.962148	0.509456	0.739885

1030	GO_NEGATIVE_REGULATION_OF_INTRACELLULAR_TRANSPORT	16	0.961746	0.511628	0.739966
1031	GO_ORGAN_GROWTH	41	0.960464	0.547879	0.741714
1032	GO_NUCLEAR_TRANSCRIBED_MRNA_CATABOLIC_PROCESS	50	0.95971	0.536165	0.742458
1033	GO_INTERFERON_GAMMA_PRODUCTION	35	0.959577	0.515263	0.741962
1034	GO_GLIOGENESIS	85	0.958995	0.556634	0.7424
1035	GO_EXOCYTIC_PROCESS	17	0.95885	0.51989	0.741983
1036	GO_REGULATION_OF_INTRACELLULAR_STEROID_HORMONE_RECEPTOR_SIGNALING_PATHWAY	18	0.958124	0.51105	0.742666
1037	GO_POSITIVE_REGULATION_OF_HEMOPOIESIS	57	0.957234	0.536782	0.743752
1038	GO_ENDOMEMBRANE_SYSTEM_ORGANIZATION	120	0.95711	0.577004	0.743264
1039	GO_CARDIAC_MUSCLE_CELL_DIFFERENTIATION	21	0.956968	0.50983	0.742842
1040	GO_REGULATION_OF_RAS_PROTEIN_SIGNAL_TRANSDUCTION	54	0.956239	0.543353	0.743523
1041	GO_CANONICAL_WNT_SIGNALING_PATHWAY	91	0.955281	0.555914	0.744705
1042	GO_POSITIVE_REGULATION_OF_MULTICELLULAR_ORGANISMAL_PROCESS	426	0.954521	0.637638	0.745471
1043	GO_REGULATION_OF_SYNAPTIC_PLASTICITY	39	0.953615	0.530466	0.746515
1044	GO_NEGATIVE_REGULATION_OF_RESPONSE_TO_CYTOKINE_STIMULUS	18	0.953277	0.527152	0.74645
1045	GO_REGULATION_OF_HYDROLASE_ACTIVITY	348	0.952322	0.617618	0.747625
1046	GO_CYTOKINE_PRODUCTION	211	0.948281	0.596758	0.754909
1047	GO_POSITIVE_REGULATION_OF_TRANSCRIPTION_BY_RNA_POLYMERASE_II	264	0.947392	0.617854	0.7559
1048	GO_REGULATION_OF_PROTEIN_DEPHOSPHORYLATION	40	0.947157	0.553571	0.755605
1049	GO_NEGATIVE_REGULATION_OF_TRANSFERASE_ACTIVITY	82	0.946885	0.568909	0.755425
1050	GO_MODULATION_BY_SYMBIONT_OF_HOST_PROCESS	15	0.946804	0.542936	0.754865
1051	GO_ORGANOPHOSPHATE_METABOLIC_PROCESS	299	0.945647	0.612613	0.756427
1052	GO_CELLULAR_TRANSITION_METAL_ION_HOMEOSTASIS	29	0.944553	0.551857	0.75779
1053	GO_REGULATION_OF_LEUKOCYTE_PROLIFERATION	76	0.943573	0.562914	0.758948
1054	GO_MAINTENANCE_OF_LOCATION	75	0.942269	0.566921	0.760743
1055	GO_NEGATIVE_REGULATION_OF_UBIQUITIN_DEPENDENT_PROTEIN_CATABOLIC_PROCESS	17	0.942143	0.534188	0.760262
1056	GO_POSITIVE_REGULATION_OF_CELL_CELL_ADHESION	81	0.942074	0.569593	0.759663
1057	GO_POSITIVE_REGULATION_OF_INSULIN_SECRETION	17	0.941942	0.549932	0.759192
1058	GO_NEGATIVE_REGULATION_OF_PROTEIN_MODIFICATION_PROCESS	178	0.941762	0.607505	0.758802
1059	GO_CELLULAR_RESPONSE_TO_EXTRACELLULAR_STIMULUS	52	0.939855	0.559819	0.761728
1060	GO_IMPORT_ACROSS_PLASMA_MEMBRANE	32	0.939385	0.563591	0.761873
1061	GO_NEGATIVE_REGULATION_OF_SMALL_MOLECULE_METABOLIC_PROCESS	18	0.937426	0.52381	0.764853
1062	GO_POSITIVE_REGULATION_OF_MYELOID_CELL_DIFFERENTIATION	26	0.937414	0.542784	0.764158
1063	GO_CELLULAR_COMPONENT_MAINTENANCE	17	0.935912	0.517289	0.76629
1064	GO_TOXIN_TRANSPORT	15	0.934762	0.549356	0.767815
1065	GO_PROTEIN_O_LINKED_GLYCOSYLATION	26	0.933742	0.536364	0.768997
1066	GO_LIPID_CATABOLIC_PROCESS	76	0.933559	0.566631	0.76862
1067	GO_GLYCOSYLATION	63	0.93332	0.566038	0.768345
1068	GO_MOVEMENT_IN_HOST_ENVIRONMENT	42	0.93281	0.5625	0.768608
1069	GO_RIBOSE_PHOSPHATE_METABOLIC_PROCESS	138	0.932347	0.605372	0.76875
1070	GO_ALPHA_AMINO_ACID_CATABOLIC_PROCESS	35	0.931896	0.568408	0.768878
1071	GO_POSITIVE_REGULATION_OF_ALPHA_BETA_T_CELL_DIFFERENTIATION	18	0.931455	0.554953	0.768959
1072	GO_RESPONSE_TO_RETINOIC_ACID	17	0.931002	0.559889	0.769117

1073	GO_RESPONSE_TO_ENDOGENOUS_STIMULUS	363	0.929658	0.679037	0.770842
1074	GO_CELLULAR_RESPONSE_TO_HORMONE_STIMULUS	138	0.929622	0.626582	0.770193
1075	GO_REGULATION_OF_INTRACELLULAR_SIGNAL_TRANSDUCTION	476	0.92853	0.699	0.771511
1076	GO_NEGATIVE_REGULATION_OF_CELLULAR_PROTEIN_LOCALIZATION	37	0.927819	0.561661	0.772206
1077	GO_TISSUE_REGENERATION	17	0.925611	0.567065	0.775662
1078	GO_POSITIVE_REGULATION_OF_TRANSLATION	42	0.924434	0.566308	0.77719
1079	GO_METENCEPHALON_DEVELOPMENT	28	0.920929	0.585242	0.782918
1080	GO_NEGATIVE_REGULATION_OF_PHOSPHORUS_METABOLIC_PROCESS	162	0.920471	0.636929	0.783035
1081	GO_MUSCLE_CELL_PROLIFERATION	49	0.919738	0.60514	0.783681
1082	GO_NEGATIVE_REGULATION_OF_TRANSPORT	101	0.918815	0.597861	0.784731
1083	GO_RESPONSE_TO_MOLECULE_OF_BACTERIAL_ORIGIN	87	0.916796	0.619459	0.787765
1084	GO_RESPONSE_TO_AMYLOID_BETA	16	0.916673	0.571429	0.787256
1085	GO_LACTATION	15	0.916298	0.571027	0.787205
1086	GO_POSITIVE_REGULATION_OF_T_CELL_PROLIFERATION	38	0.915118	0.593564	0.788695
1087	GO_NEGATIVE_REGULATION_OF_DEVELOPMENTAL_PROCESS	204	0.914895	0.669706	0.788387
1088	GO_RIBOSOMAL_LARGE_SUBUNIT_BIOGENESIS	20	0.914544	0.591029	0.788289
1089	GO_CELLULAR_RESPONSE_TO_AMYLOID_BETA	16	0.913278	0.578445	0.78984
1090	GO_POSITIVE_REGULATION_OF_CALCIUM_ION_TRANSMEMBRANE_TRA NSPORT	15	0.913276	0.579772	0.789117
1091	GO_POSITIVE_REGULATION_OF_HYDROLASE_ACTIVITY	213	0.911918	0.684157	0.790909
1092	GO_NEGATIVE_REGULATION_OF_PROTEOLYSIS	80	0.910456	0.613082	0.792921
1093	GO_CARBOHYDRATE_DERIVATIVE_TRANSPORT	16	0.908932	0.590327	0.794964
1094	GO_RIBOSOME_ASSEMBLY	22	0.908319	0.58681	0.795302
1095	GO_NEGATIVE_REGULATION_OF_IMMUNE_SYSTEM_PROCESS	124	0.907308	0.639791	0.796381
1096	GO_MUSCLE_ADAPTATION	23	0.907187	0.586162	0.795895
1097	GO_SENSORY_PERCEPTION_OF_MECHANICAL_STIMULUS	33	0.906195	0.59322	0.797011
1098	GO_PROTEIN_MATURATION	74	0.903372	0.621858	0.801335
1099	GO_LYMPHOCYTE_MIGRATION	24	0.901591	0.609314	0.803751
1100	GO_NEGATIVE_REGULATION_OF_CELL_DIFFERENTIATION	147	0.897974	0.648454	0.80962
1101	GO_REGULATION_OF_CYTOSOLIC_CALCIUM_ION_CONCENTRATION	79	0.896929	0.646799	0.81075
1102	GO_POSITIVE_REGULATION_OF_TRANSPORTER_ACTIVITY	20	0.896928	0.59279	0.810018
1103	GO_REGULATION_OF_VIRAL_TRANSCRIPTION	26	0.896008	0.623342	0.810951
1104	GO_REGULATION_OF_WNT_SIGNALING_PATHWAY	100	0.895014	0.643083	0.812029
1105	GO_STEROL_BIOSYNTHETIC_PROCESS	29	0.894304	0.613316	0.812538
1106	GO_NEGATIVE_REGULATION_OF_ION_TRANSPORT	27	0.893927	0.619824	0.812445
1107	GO_INORGANIC_ION_IMPORT_ACROSS_PLASMA_MEMBRANE	17	0.893815	0.586867	0.811908
1108	GO_ORGANIC_ACID_CATABOLIC_PROCESS	80	0.891998	0.646018	0.814403
1109	GO_FATTY_ACID_CATABOLIC_PROCESS	35	0.891893	0.607013	0.813857
1110	GO_INTRACELLULAR_PROTEIN_TRANSPORT	337	0.891223	0.746493	0.814332
1111	GO_TERPENOID_METABOLIC_PROCESS	22	0.889285	0.603723	0.817053
1112	GO_ACTIN_MEDIATED_CELL_CONTRACTION	25	0.888573	0.624366	0.817592
1113	GO_NEGATIVE_REGULATION_OF_CELL_ACTIVATION	52	0.888073	0.639629	0.81777
1114	GO_FATTY_ACID_DERIVATIVE_BIOSYNTHETIC_PROCESS	22	0.887545	0.615385	0.817915
1115	GO_NEGATIVE_REGULATION_OF_CATABOLIC_PROCESS	76	0.883424	0.648738	0.824369
1116	GO_MUSCLE_HYPERTROPHY	21	0.882146	0.601307	0.825892

1117	GO_SIGNAL_TRANSDUCTION_BY_PROTEIN_PHOSPHORYLATION	235	0.875884	0.748231	0.835963
1118	GO_POSITIVE_REGULATION_OF_DEVELOPMENTAL_PROCESS	310	0.875534	0.774874	0.835844
1119	GO_NEGATIVE_REGULATION_OF_PHOSPHORYLATION	127	0.874011	0.6875	0.837654
1120	GO_NEGATIVE_REGULATION_OF_SUPRAMOLECULAR_FIBER_ORGANIZATION	38	0.873961	0.633374	0.836983
1121	GO_ALCOHOL_METABOLIC_PROCESS	108	0.873736	0.68984	0.836606
1122	GO_MEGAKARYOCYTE_DIFFERENTIATION	25	0.873089	0.627141	0.836952
1123	GO_NEGATIVE_REGULATION_OF_RESPONSE_TO_STIMULUS	417	0.87298	0.802	0.836405
1124	GO_DIVALENT_INORGANIC_CATION_HOMEOSTASIS	114	0.87079	0.689873	0.839377
1125	GO_AMINOGLYCAN_BIOSYNTHETIC_PROCESS	26	0.870072	0.624672	0.839864
1126	GO_DETECTION_OF_STIMULUS_INVOLVED_IN_SENSORY_PERCEPTION	17	0.868896	0.617354	0.841085
1127	GO_AROMATIC_AMINO_ACID_FAMILY_METABOLIC_PROCESS	15	0.868475	0.639716	0.841029
1128	GO_CELL_CELL_SIGNALING_BY_WNT	131	0.868167	0.710251	0.840817
1129	GO_REGULATION_OF_RESPONSE_TO_OXIDATIVE_STRESS	24	0.867963	0.628989	0.840418
1130	GO_POSITIVE_REGULATION_OF_ION_TRANSMEMBRANE_TRANSPORT	35	0.867181	0.632099	0.840988
1131	GO_REGULATION_OF_MYELOID_CELL_DIFFERENTIATION	65	0.865291	0.6566	0.843421
1132	GO_MAMMARY_GLAND_DEVELOPMENT	36	0.865146	0.644802	0.842941
1133	GO_POSITIVE_REGULATION_OF_HORMONE_SECRETION	28	0.862413	0.661104	0.846778
1134	GO_PEPTIDYL_PROLINE_MODIFICATION	21	0.862002	0.629827	0.846707
1135	GO_PROTEIN_TRANSPORT_ALONG_MICROTUBULE	22	0.861872	0.618863	0.846173
1136	GO_NEGATIVE_REGULATION_OF_PEPTIDASE_ACTIVITY	53	0.861657	0.663605	0.845801
1137	GO_TISSUE_MORPHOGENESIS	153	0.86028	0.720041	0.847317
1138	GO_MESENCHYMAL_CELL_DIFFERENTIATION	45	0.860221	0.657109	0.846663
1139	GO_MULTICELLULAR_ORGANISMAL_HOMEOSTASIS	118	0.859052	0.71714	0.847934
1140	GO_REGULATION_OF_TUBE_SIZE	20	0.85835	0.643836	0.848299
1141	GO_MONOCARBOXYLIC_ACID_CATABOLIC_PROCESS	37	0.857352	0.653753	0.849223
1142	GO_REGULATION_OF_CYTOKINE_PRODUCTION_INVOLVED_IN_IMMUNE_RESPONSE	22	0.856091	0.644872	0.850509
1143	GO_POSITIVE_REGULATION_OF_TRANSPORT	226	0.855591	0.752275	0.850588
1144	GO_PRIMARY_ALCOHOL_METABOLIC_PROCESS	21	0.85468	0.663564	0.851359
1145	GO_TYROSINE_PHOSPHORYLATION_OF_STAT_PROTEIN	18	0.853747	0.660665	0.852142
1146	GO_CELL_PROJECTION_ASSEMBLY	166	0.853599	0.762887	0.851637
1147	GO_EPITHELIAL_TO_MESENCHYMAL_TRANSITION	30	0.852156	0.674102	0.853291
1148	GO_DEPHOSPHORYLATION	157	0.852089	0.742857	0.852661
1149	GO_REGULATION_OF_CELLULAR_COMPONENT_BIOGENESIS	256	0.84865	0.801619	0.857477
1150	GO_MUSCLE_TISSUE_DEVELOPMENT	83	0.848155	0.705628	0.857542
1151	GO_REGULATION_OF_GROWTH	163	0.84805	0.757764	0.856952
1152	GO_REGULATION_OF_PEPTIDE_TRANSPORT	195	0.847304	0.798576	0.857373
1153	GO_T_CELL_MIGRATION	17	0.84708	0.644979	0.857
1154	GO_POLYOL_METABOLIC_PROCESS	37	0.846983	0.652862	0.85642
1155	GO_ORGANIC_HYDROXY_COMPOUND_METABOLIC_PROCESS	146	0.846423	0.75129	0.856577
1156	GO_MICROTUBULE_BUNDLE_FORMATION	21	0.846136	0.659211	0.85628
1157	GO_NUCLEOTIDE_PHOSPHORYLATION	41	0.846045	0.681657	0.855682
1158	GO_POSITIVE_REGULATION_OF_EPITHELIAL_CELL_PROLIFERATION	41	0.845929	0.685512	0.855124
1159	GO_PROTEIN_TARGETING	136	0.845051	0.743992	0.855796
1160	GO_INTERLEUKIN_12_PRODUCTION	19	0.844863	0.664402	0.85537

1161	GO_REGULATION_OF_CELL_DIFFERENTIATION	434	0.842926	0.868	0.857785
1162	GO_ESTABLISHMENT_OF_PROTEIN_LOCALIZATION_TO_MEMBRANE	119	0.842802	0.74109	0.857256
1163	GO_NEGATIVE_REGULATION_OF_INTRINSIC_APOPTOTIC_SIGNALING_PATHWAY	22	0.837371	0.662634	0.865146
1164	GO_NEUROTRANSMITTER_TRANSPORT	43	0.835635	0.699401	0.867167
1165	GO_NEGATIVE_REGULATION_OF_SIGNALING	352	0.835487	0.851703	0.866654
1166	GO_MIDBRAIN_DEVELOPMENT	22	0.835075	0.655218	0.866539
1167	GO_MITOCHONDRIAL_RNA_METABOLIC_PROCESS	17	0.834859	0.663877	0.866112
1168	GO_POSITIVE_REGULATION_OF_PEPTIDE_HORMONE_SECRETION	21	0.832809	0.692209	0.868627
1169	GO_T_CELL_PROLIFERATION	65	0.830991	0.713496	0.870672
1170	GO_CARBOHYDRATE_METABOLIC_PROCESS	160	0.827413	0.769547	0.875373
1171	GO_REGULATION_OF_CHOLESTEROL_BIOSYNTHETIC_PROCESS	15	0.825398	0.682336	0.87767
1172	GO_REGULATION_OF_NERVOUS_SYSTEM_PROCESS	30	0.824494	0.691358	0.878323
1173	GO_CELLULAR_ION_HOMEOSTASIS	152	0.823828	0.773101	0.878562
1174	GO_INTRACELLULAR_RECEPTOR_SIGNALING_PATHWAY	61	0.822484	0.699214	0.879851
1175	GO_VENTRICULAR_SEPTUM_DEVELOPMENT	16	0.822164	0.684636	0.879579
1176	GO_MUSCLE_SYSTEM_PROCESS	84	0.821788	0.736443	0.879413
1177	GO_GOLGI_VESICLE_BUDDING	20	0.821368	0.695538	0.879293
1178	GO_RESPONSE_TO_MECHANICAL_STIMULUS	44	0.820977	0.726415	0.879145
1179	GO_PROTEIN_N_LINKED_GLYCOSYLATION	19	0.820698	0.691391	0.878802
1180	GO_POSITIVE_REGULATION_OF_SIGNALING	455	0.819955	0.886	0.879165
1181	GO_MYELOID_CELL_DIFFERENTIATION	113	0.819624	0.768743	0.878897
1182	GO_RESPONSE_TO_CAMP	21	0.818586	0.705805	0.879718
1183	GO_MYELOID_LEUKOCYTE_MEDIATED_IMMUNITY	124	0.818419	0.78178	0.879236
1184	GO_CATION_TRANSMEMBRANE_TRANSPORT	180	0.818399	0.791152	0.878525
1185	GO_NEGATIVE_REGULATION_OF_INNATE_IMMUNE_RESPONSE	20	0.818207	0.692513	0.878059
1186	GO_POSITIVE_REGULATION_OF_PHAGOCYTOSIS	22	0.816644	0.690349	0.87957
1187	GO_MAINTENANCE_OF_PROTEIN_LOCATION	29	0.815635	0.696517	0.880371
1188	GO_NEGATIVE_REGULATION_OF_LIPID_METABOLIC_PROCESS	17	0.815634	0.687671	0.87963
1189	GO_POSITIVE_REGULATION_OF_LYMPHOCYTE_DIFFERENTIATION	33	0.815457	0.717233	0.879167
1190	GO_POST_EMBRYONIC_DEVELOPMENT	19	0.814227	0.705882	0.880239
1191	GO_TRANSMEMBRANE_TRANSPORT	354	0.812165	0.872	0.882576
1192	GO_REGULATION_OF_CELL_CELL_ADHESION	114	0.809986	0.792059	0.885077
1193	GO_REGULATION_OF_PHOSPHOPROTEIN_PHOSPHATASE_ACTIVITY	33	0.808943	0.727157	0.885849
1194	GO_EMBRYONIC_ORGAN_MORPHOGENESIS	48	0.808834	0.720329	0.885269
1195	GO_CARBOHYDRATE_TRANSMEMBRANE_TRANSPORT	25	0.80871	0.713178	0.884699
1196	GO_VESICLE_TARGETING_TO_FROM_OR_WITHIN_GOLGI	19	0.806317	0.703104	0.887378
1197	GO_PATHWAY_RESTRICTED_SMAD_PROTEIN_PHOSPHORYLATION	19	0.80607	0.69863	0.887011
1198	GO_REGULATION_OF_MRNA_SPLICING_VIA_SPLICEOSOME	27	0.803437	0.704134	0.890096
1199	GO_NEGATIVE_REGULATION_OF_CELLULAR_PROTEIN_CATABOLIC_PROCESS	26	0.803072	0.706767	0.889871
1200	GO_TOLL_LIKE_RECEPTOR_SIGNALING_PATHWAY	38	0.803005	0.725209	0.889217
1201	GO_INORGANIC_ION_TRANSMEMBRANE_TRANSPORT	174	0.802757	0.816033	0.888818
1202	GO_TRANSPORT_ALONG_MICROTUBULE	41	0.799994	0.743682	0.891909
1203	GO_POSITIVE_REGULATION_OF_TYROSINE_PHOSPHORYLATION_OF_STAT_PROTEIN	15	0.799595	0.717115	0.891692
1204	GO_POSITIVE_REGULATION_OF_VIRAL_TRANSCRIPTION	18	0.799145	0.702811	0.891636

1205	GO_POSITIVE_REGULATION_OF_OSSIFICATION	21	0.798735	0.723097	0.891519
1206	GO_NUCLEOSIDE_DIPHOSPHATE_METABOLIC_PROCESS	47	0.798277	0.753201	0.891424
1207	GO_MUSCLE_CONTRACTION	61	0.798267	0.75885	0.8907
1208	GO_TUBE_FORMATION	39	0.797979	0.749701	0.890365
1209	GO_CENTRAL_NERVOUS_SYSTEM_DEVELOPMENT	245	0.797154	0.850505	0.890788
1210	GO_POSITIVE_REGULATION_OF_GROWTH	55	0.796821	0.761741	0.890527
1211	GO_MONOVALENT_INORGANIC_CATION_TRANSPORT	116	0.795621	0.800839	0.891448
1212	GO_REGULATION_OF_STEROID_METABOLIC_PROCESS	30	0.794452	0.721598	0.892365
1213	GO_MYELOID_LEUKOCYTE_ACTIVATION	155	0.793385	0.823347	0.893076
1214	GO_POSITIVE_REGULATION_OF_ION_TRANSPORT	52	0.793113	0.754587	0.892723
1215	GO_MAINTENANCE_OF_CELL_NUMBER	47	0.792623	0.762632	0.892695
1216	GO_MACROAUTOPHAGY	75	0.792167	0.765795	0.892576
1217	GO_SENSORY_PERCEPTION_OF_PAIN	18	0.791723	0.723849	0.89246
1218	GO_ENDOSOME_ORGANIZATION	24	0.788288	0.728192	0.896489
1219	GO_REGULATION_OF_PROTEIN_TARGETING	29	0.787992	0.754037	0.896188
1220	GO_NEGATIVE_REGULATION_OF_PEPTIDE_SECRETION	24	0.787721	0.728924	0.895838
1221	GO_CELLULAR_RESPONSE_TO_ENDOGENOUS_STIMULUS	305	0.784938	0.895267	0.898822
1222	GO_REGULATION_OF_EPITHELIAL_TO_MESENCHYMAL_TRANSITION	18	0.784574	0.713306	0.898608
1223	GO_CELLULAR_AMINO_ACID_CATABOLIC_PROCESS	41	0.784152	0.737783	0.898476
1224	GO_HEART_DEVELOPMENT	118	0.781794	0.804646	0.900974
1225	GO_ORGANIC_ACID_TRANSPORT	65	0.780974	0.789888	0.901404
1226	GO_LIPID_BIOSYNTHETIC_PROCESS	178	0.779434	0.850153	0.902646
1227	GO_NEGATIVE_REGULATION_OF_CELLULAR_CATABOLIC_PROCESS	60	0.778827	0.784651	0.902757
1228	GO_REGULATION_OF_PHAGOCYTOSIS	26	0.778205	0.726316	0.902883
1229	GO_REGULATION_OF_MUSCLE_CONTRACTION	26	0.777777	0.725289	0.902716
1230	GO_SULFUR_COMPOUND_BIOSYNTHETIC_PROCESS	48	0.776354	0.756725	0.903865
1231	GO_CHEMICAL_HOMEOSTASIS	259	0.775607	0.896274	0.904094
1232	GO_NEGATIVE_REGULATION_OF_RESPONSE_TO_BIOTIC_STIMULUS	28	0.773863	0.750929	0.905756
1233	GO_CIRCULATORY_SYSTEM_DEVELOPMENT	237	0.773515	0.886869	0.905462
1234	GO_REGULATION_OF_STRIATED_MUSCLE_CONTRACTION	15	0.772262	0.738686	0.906332
1235	GO_T_CELL_DIFFERENTIATION_IN_THYMUS	25	0.771572	0.758256	0.906474
1236	GO_NEGATIVE_REGULATION_OF_AUTOPHAGY	16	0.771295	0.714481	0.906118
1237	GO_NEURAL_TUBE_DEVELOPMENT	43	0.769834	0.78219	0.907302
1238	GO_NEGATIVE_REGULATION_OF_INTRACELLULAR_SIGNAL_TRANSDUCTION	128	0.768042	0.836798	0.908848
1239	GO_REGULATION_OF_STRIATED_MUSCLE_CELL_DIFFERENTIATION	19	0.767729	0.74121	0.908511
1240	GO_NEURON_DEATH	96	0.766953	0.83049	0.908769
1241	GO_COPII_COATED_VESICLE_BUDDING	18	0.766672	0.749337	0.908408
1242	GO_ENSHEATHMENT_OF_NEURONS	48	0.766622	0.79186	0.90773
1243	GO_STEROID_METABOLIC_PROCESS	81	0.761011	0.833333	0.914154
1244	GO_REGULATION_OF_POSTSYNAPTIC_MEMBRANE_NEUROTRANSMITTER_RECEPTOR_LEVELS	23	0.7581	0.779255	0.916943
1245	GO_HEXOSE_CATABOLIC_PROCESS	19	0.7573	0.761773	0.917212
1246	GO_NEGATIVE_REGULATION_OF_NEURON_APOPTOTIC_PROCESS	35	0.756925	0.783147	0.916942
1247	GO_CARDIAC_SEPTUM_MORPHOGENESIS	18	0.755733	0.752434	0.917721
1248	GO_GOLGI_ORGANIZATION	43	0.754359	0.787336	0.918661

1249	GO_SCHWANN_CELL_DIFFERENTIATION	19	0.752514	0.761442	0.920144
1250	GO_POSITIVE_REGULATION_OF_AUTOPHAGY	28	0.751959	0.781523	0.920101
1251	GO_REGULATION_OF_AUTOPHAGY	86	0.751582	0.822771	0.919833
1252	GO_MULTIVESICULAR_BODY_SORTING_PATHWAY	18	0.748678	0.770308	0.922564
1253	GO_ION_TRANSMEMBRANE_TRANSPORT	234	0.747185	0.90625	0.923609
1254	GO_SUBSTRATE_ADHESION_DEPENDENT_CELL_SPREADING	23	0.745564	0.777778	0.924816
1255	GO_POSITIVE_REGULATION_OF_CELLULAR_COMPONENT_BIOGENESIS	149	0.744508	0.886577	0.925377
1256	GO_REGULATION_OF_CATION_TRANSMEMBRANE_TRANSPORT	66	0.74382	0.826136	0.925463
1257	GO_METAL_ION_HOMEOSTASIS	149	0.7435	0.88601	0.925128
1258	GO_NEURAL_TUBE_FORMATION	29	0.742557	0.789926	0.925476
1259	GO_ION_HOMEOSTASIS	182	0.741331	0.893037	0.926225
1260	GO_REGULATION_OF_CELLULAR_RESPONSE_TO_INSULIN_STIMULUS	15	0.74	0.785714	0.92705
1261	GO_CALCIUM_MEDIATED_SIGNALING	56	0.73934	0.839225	0.927127
1262	GO_RECEPTOR_INTERNALIZATION	24	0.739001	0.777487	0.926777
1263	GO_TOR_SIGNALING	33	0.738977	0.797546	0.926064
1264	GO_MONOSACCHARIDE_CATABOLIC_PROCESS	22	0.738275	0.780585	0.926206
1265	GO_CARDIAC_MUSCLE_TISSUE_DEVELOPMENT	36	0.738142	0.812887	0.925647
1266	GO_NADH_METABOLIC_PROCESS	18	0.737659	0.772169	0.925454
1267	GO_NEGATIVE_REGULATION_OF_SECRETION	41	0.737653	0.818075	0.924729
1268	GO_STRIATED_MUSCLE_CELL_DIFFERENTIATION	52	0.737433	0.832377	0.924246
1269	GO_RESPONSE_TO_ENDOPLASMIC_RETICULUM_STRESS	76	0.736455	0.842163	0.924672
1270	GO_VESICLE_TARGETING	23	0.736176	0.787402	0.924266
1271	GO_CELL_SURFACE_RECEPTOR_SIGNALING_PATHWAY_INVOLVED_IN_C ELL_CELL_SIGNALING	147	0.733605	0.889804	0.926445
1272	GO_REGULATION_OF_TRANSPORT	406	0.733371	0.965966	0.925996
1273	GO_CARDIAC_MUSCLE_CELL_CONTRACTION	15	0.732037	0.790411	0.926798
1274	GO_ACTIN_FILAMENT_BASED_PROCESS	193	0.730453	0.919797	0.92785
1275	GO_POSITIVE_REGULATION_OF_PROTEIN_TYROSINE_KINASE_ACTIVITY	16	0.730381	0.765928	0.9272
1276	GO_VESICLE_ORGANIZATION	91	0.729112	0.853503	0.927924
1277	GO_MAINTENANCE_OF_PROTEIN_LOCATION_IN_CELL	20	0.727914	0.778506	0.928489
1278	GO_POSITIVE_REGULATION_OF_RECEPTOR_SIGNALING_PATHWAY_VIA_ STAT	20	0.727471	0.797082	0.928275
1279	GO_ISOPRENOID_METABOLIC_PROCESS	26	0.726596	0.789072	0.928506
1280	GO_ACTIN_FILAMENT_BASED_MOVEMENT	33	0.72586	0.800496	0.928596
1281	GO_REGULATION_OF_MACROAUTOPHAGY	39	0.724922	0.831933	0.928899
1282	GO_CELLULAR_RESPONSE_TO_KETONE	23	0.724848	0.79118	0.928248
1283	GO_POSITIVE_REGULATION_OF_CYTOSKELETON_ORGANIZATION	61	0.721294	0.841866	0.931342
1284	GO_MUSCLE_CELL_DIFFERENTIATION	70	0.720062	0.853439	0.931971
1285	GO_CALCIUM_ION_TRANSMEMBRANE_TRANSPORT	69	0.716617	0.86	0.93499
1286	GO_CYTOPLASMIC_PATTERN_RECOGNITION_RECEPTOR_SIGNALING_PA THWAY	21	0.714231	0.819737	0.936853
1287	GO_REGULATION_OF_TOR_SIGNALING	24	0.709303	0.815445	0.941406
1288	GO_CARBOHYDRATE_BIOSYNTHETIC_PROCESS	55	0.707718	0.857809	0.94235
1289	GO_REGULATION_OF_CALCIUM_ION_TRANSMEMBRANE_TRANSPORT	31	0.707711	0.837532	0.941632
1290	GO_NUCLEOSIDE_BISPHOSPHATE_METABOLIC_PROCESS	40	0.706588	0.839094	0.942079
1291	GO_TRANSITION_METAL_ION_HOMEOSTASIS	36	0.703178	0.840149	0.944792

1292	GO_NUCLEAR_TRANSCRIBED_MRNA_CATABOLIC_PROCESS_NONSENSE_MEDIATED_DECAY	28	0.702303	0.836086	0.944972
1293	GO_GLYCEROLIPID_BIOSYNTHETIC_PROCESS	57	0.699751	0.85426	0.946851
1294	GO_PHOSPHOLIPID_BIOSYNTHETIC_PROCESS	67	0.696559	0.859107	0.949245
1295	GO_GLUCOSE_METABOLIC_PROCESS	60	0.694567	0.871972	0.950517
1296	GO_NEGATIVE_REGULATION_OF_ESTABLISHMENT_OF_PROTEIN_LOCALIZATION	44	0.694045	0.86342	0.950318
1297	GO_REGULATION_OF_CALCIUM_ION_TRANSMEMBRANE_TRANSPORTER_ACTIVITY	15	0.692927	0.822558	0.950654
1298	GO_REGULATION_OF_TRANSMEMBRANE_TRANSPORT	112	0.692518	0.909862	0.950286
1299	GO_HORMONE_MEDIATED_SIGNALING_PATHWAY	45	0.690421	0.863527	0.951568
1300	GO_THIOESTER_METABOLIC_PROCESS	30	0.690117	0.845	0.951144
1301	GO_REGULATION_OF_CALCIUM_ION_TRANSPORT	50	0.690033	0.872812	0.950498
1302	GO_SECOND_MESSENGER_MEDIATED_SIGNALING	95	0.687822	0.896257	0.951847
1303	GO_REGULATION_OF_CATION_CHANNEL_ACTIVITY	28	0.68758	0.848366	0.951363
1304	GO_NEGATIVE_REGULATION_OF_CELL_CELL_ADHESION	45	0.686995	0.877294	0.951177
1305	GO_POSITIVE_REGULATION_OF_LEUKOCYTE_DIFFERENTIATION	42	0.686692	0.831742	0.950727
1306	GO_RECEPTOR_METABOLIC_PROCESS	46	0.682782	0.869048	0.953757
1307	GO_REGULATION_OF_BODY_FLUID_LEVELS	118	0.681271	0.924051	0.954429
1308	GO_CARBOHYDRATE_TRANSPORT	31	0.675449	0.848259	0.959029
1309	GO_REGULATION_OF_LIPID_METABOLIC_PROCESS	109	0.675269	0.924388	0.958458
1310	GO_PANCREAS_DEVELOPMENT	16	0.671835	0.84509	0.960832
1311	GO_REGULATION_OF_CARBOHYDRATE_CATABOLIC_PROCESS	23	0.671429	0.863291	0.960458
1312	GO_REGULATION_OF_PROTEIN_TYROSINE_KINASE_ACTIVITY	26	0.670595	0.875481	0.960426
1313	GO_FERTILIZATION	37	0.670522	0.846523	0.959764
1314	GO_POSITIVE_REGULATION_OF_CELL_ADHESION	112	0.668616	0.927802	0.960742
1315	GO_EPITHELIAL_TUBE_MORPHOGENESIS	68	0.668235	0.890122	0.960355
1316	GO_PROTEIN_LOCALIZATION_TO_MEMBRANE	188	0.665986	0.951992	0.961633
1317	GO_CELL_GROWTH	112	0.665772	0.932059	0.961083
1318	GO_NEGATIVE_REGULATION_OF_ERBB_SIGNALING_PATHWAY	16	0.665192	0.836592	0.960814
1319	GO_CHAPERONE_MEDIATED_PROTEIN_FOLDING	22	0.665128	0.876095	0.960142
1320	GO_MONOSACCHARIDE_METABOLIC_PROCESS	73	0.662536	0.904551	0.961698
1321	GO_POSITIVE_REGULATION_OF_PROTEIN_LOCALIZATION_TO_MEMBRANE	37	0.661977	0.862027	0.961439
1322	GO_MUSCLE_STRUCTURE_DEVELOPMENT	131	0.660635	0.936524	0.961843
1323	GO_LEUKOCYTE_CHEMOTAXIS	50	0.659559	0.893148	0.962024
1324	GO_PATTERN_RECOGNITION_RECEPTOR_SIGNALING_PATHWAY	55	0.6586	0.907429	0.962094
1325	GO_NEURON_MIGRATION	33	0.657672	0.883436	0.962167
1326	GO_REGULATION_OF_HEART_RATE	19	0.656674	0.85695	0.962269
1327	GO_REGULATION_OF_MUSCLE_CELL_DIFFERENTIATION	31	0.656661	0.885678	0.961555
1328	GO_CELL_CHEMOTAXIS	66	0.656367	0.902331	0.961066
1329	GO_STEROL_METABOLIC_PROCESS	49	0.656033	0.896355	0.960615
1330	GO_GLYCEROPHOSPHOLIPID_METABOLIC_PROCESS	85	0.654031	0.92973	0.961531
1331	GO_EMBRYONIC_SKELETAL_SYSTEM_DEVELOPMENT	27	0.653669	0.872562	0.961124
1332	GO_DICARBOXYLIC_ACID_TRANSPORT	17	0.653339	0.872848	0.960685
1333	GO_RESPONSE_TO_OSMOTIC_STRESS	17	0.652752	0.842896	0.960424
1334	GO_CERAMIDE_BIOSYNTHETIC_PROCESS	15	0.65253	0.853147	0.959891

1335	GO_REGULATION_OF_CELL_ADHESION	184	0.65231	0.95209	0.959347
1336	GO_REGULATION_OF_CALCIUM_MEDIATED_SIGNALING	25	0.6495	0.874005	0.960878
1337	GO_REGULATION_OF_ION_TRANSMEMBRANE_TRANSPORT	97	0.646508	0.930108	0.962593
1338	GO_GLYCOPROTEIN_BIOSYNTHETIC_PROCESS	86	0.645989	0.940347	0.962264
1339	GO_NEGATIVE_REGULATION_OF_ERK1_AND_ERK2_CASCADE	20	0.645493	0.860304	0.961951
1340	GO_RESPONSE_TO_PURINE_CONTAINING_COMPOUND	29	0.644599	0.886452	0.961936
1341	GO_SKELETAL_MUSCLE_ORGAN_DEVELOPMENT	40	0.64186	0.89199	0.9633
1342	GO_REGULATION_OF_RECEPTOR_SIGNALING_PATHWAY_VIA_STAT	30	0.638606	0.881612	0.965071
1343	GO_PLASMA_MEMBRANE_ORGANIZATION	24	0.637882	0.873221	0.964877
1344	GO_COTRANSLATIONAL_PROTEIN_TARGETING_TO_MEMBRANE	25	0.637384	0.890152	0.964546
1345	GO_POSITIVE_REGULATION_OF_PLASMA_MEMBRANE_BOUNDED_CELL_PROJECTION_ASSEMBLY	32	0.636591	0.89196	0.964404
1346	GO_INFLAMMATORY_RESPONSE	174	0.636024	0.969231	0.964112
1347	GO_POSITIVE_REGULATION_OF_TRANSMEMBRANE_TRANSPORT	45	0.635401	0.913253	0.963842
1348	GO_GLYCEROLIPID_METABOLIC_PROCESS	95	0.635312	0.929577	0.963184
1349	GO_POSITIVE_REGULATION_OF_TUMOR_NECROSIS_FACTOR_SUPERFAMILY_CYTOKINE_PRODUCTION	20	0.634837	0.89396	0.962813
1350	GO_PROCESS_UTILIZING_AUTOPHAGIC_MECHANISM	133	0.634604	0.940189	0.962258
1351	GO_PHOSPHATIDYLINOSITOL_3_KINASE_SIGNALING	40	0.634136	0.892025	0.961879
1352	GO_CATION_TRANSPORT	228	0.633124	0.978788	0.961914
1353	GO_FIBROBLAST_GROWTH_FACTOR_RECEPTOR_SIGNALING_PATHWAY	26	0.631242	0.897985	0.96252
1354	GO_I_KAPPAB_KINASE_NF_KAPPAB_SIGNALING	76	0.629614	0.930464	0.962993
1355	GO_NEGATIVE_REGULATION_OF_TUMOR_NECROSIS_FACTOR_SUPERFAMILY_CYTOKINE_PRODUCTION	15	0.629559	0.875181	0.962323
1356	GO_GLIAL_CELL_DIFFERENTIATION	67	0.627994	0.927374	0.962763
1357	GO_REGULATION_OF_NUCLEOTIDE_METABOLIC_PROCESS	28	0.625377	0.901373	0.963887
1358	GO_NEGATIVE_REGULATION_OF_ENDOTHELIAL_CELL_PROLIFERATION	19	0.625288	0.883221	0.963241
1359	GO_NEGATIVE_REGULATION_OF_CELL_ADHESION	73	0.625031	0.924259	0.962715
1360	GO_CELLULAR_LIPID_METABOLIC_PROCESS	253	0.621785	0.98494	0.964305
1361	GO_REGULATION_OF_PROTEIN_LOCALIZATION_TO_MEMBRANE	49	0.621252	0.932243	0.96397
1362	GO_REGULATION_OF_ANION_TRANSPORT	17	0.619909	0.884038	0.964175
1363	GO_INTRACELLULAR_STEROID_HORMONE_RECEPTOR_SIGNALING_PATHWAY	25	0.619188	0.90641	0.963919
1364	GO_GLUCOSE_CATABOLIC_PROCESS	15	0.618857	0.879383	0.96344
1365	GO_PIGMENTATION	32	0.618419	0.9	0.963032
1366	GO_ION_TRANSPORT	329	0.618044	0.991944	0.96258
1367	GO_GLYCOPROTEIN_METABOLIC_PROCESS	97	0.617339	0.947255	0.962325
1368	GO_POSITIVE_REGULATION_OF_INFLAMMATORY_RESPONSE	27	0.61548	0.912987	0.962873
1369	GO_NEUTROPHIL_MIGRATION	30	0.614508	0.911111	0.962818
1370	GO_CELLULAR_MONOVALENT_INORGANIC_CATION_HOMEOSTASIS	25	0.613462	0.896461	0.962796
1371	GO_TETRAPYRROLE_METABOLIC_PROCESS	18	0.609621	0.888131	0.964573
1372	GO_CARBOHYDRATE_CATABOLIC_PROCESS	54	0.609362	0.916955	0.964037
1373	GO_NEGATIVE_REGULATION_OF_CYTOKINE_PRODUCTION	74	0.607875	0.945711	0.964293
1374	GO_LIPID_MODIFICATION	71	0.606782	0.946961	0.964307
1375	GO_SINGLE_FERTILIZATION	30	0.602298	0.913965	0.966316
1376	GO_RESPONSE_TO_CALCIUM_ION	33	0.601711	0.938519	0.96597
1377	GO_ESTABLISHMENT_OF_PROTEIN_LOCALIZATION_TO_ENDOPLASMIC_RETICULUM	31	0.598385	0.926918	0.967274

1378	GO_POSITIVE_REGULATION_OF_CELL_GROWTH	30	0.5976	0.930435	0.96705
1379	GO_RESPONSE_TO_FIBROBLAST_GROWTH_FACTOR	29	0.597383	0.894479	0.966459
1380	GO_NEGATIVE_REGULATION_OF_MAP_KINASE_ACTIVITY	22	0.596772	0.910622	0.966119
1381	GO_CERAMIDE_METABOLIC_PROCESS	21	0.596226	0.9047	0.965722
1382	GO_MUSCLE_CELL_DEVELOPMENT	36	0.594222	0.938801	0.966202
1383	GO_ORGANELLE_MEMBRANE_FUSION	21	0.594222	0.919897	0.965504
1384	GO_CELLULAR_RESPONSE_TO_STEROID_HORMONE_STIMULUS	44	0.59416	0.938534	0.964844
1385	GO_MYELOID_CELL_DEVELOPMENT	16	0.593689	0.895689	0.964416
1386	GO_ENDOPLASMIC_RETICULUM_ORGANIZATION	16	0.590804	0.918508	0.965353
1387	GO_PROTEIN_LOCALIZATION_TO_ENDOPLASMIC_RETICULUM	32	0.59	0.936789	0.965112
1388	GO_ACTIN_NUCLEATION	17	0.589463	0.904828	0.964711
1389	GO_UBIQUITIN_DEPENDENT_ERAD_PATHWAY	17	0.58628	0.907895	0.965704
1390	GO_DIVALENT_INORGANIC_CATION_TRANSPORT	98	0.580699	0.972281	0.967959
1391	GO_ORGANIC_ACID_TRANSMEMBRANE_TRANSPORT	28	0.569215	0.941177	0.973016
1392	GO_CELLULAR_CARBOHYDRATE_METABOLIC_PROCESS	76	0.568099	0.97062	0.972807
1393	GO_REGULATION_OF_CILIUM_ASSEMBLY	16	0.564948	0.93166	0.97362
1394	GO_CYTOPLASMIC_TRANSLATION	20	0.554732	0.950928	0.977631
1395	GO_POSITIVE_REGULATION_OF_LEUKOCYTE_CHEMOTAXIS	21	0.546633	0.934871	0.98047
1396	GO_RESPONSE_TO_ACID_CHEMICAL	29	0.540852	0.947644	0.982102
1397	GO_ERAD_PATHWAY	24	0.539906	0.938802	0.981773
1398	GO_RETROGRADE_TRANSPORT_ENDOSOME_TO_GOLGI	24	0.536962	0.932225	0.982209
1399	GO_PH_REDUCTION	16	0.536714	0.943912	0.981602
1400	GO_NEGATIVE_REGULATION_OF_CELLULAR_RESPONSE_TO_TRANSFORMING_GROWTH_FACTOR_BETA_STIMULUS	24	0.535898	0.941634	0.981199
1401	GO_CELLULAR_HORMONE_METABOLIC_PROCESS	21	0.524525	0.952196	0.984647
1402	GO_REGULATION_OF_RELEASE_OF_SEQUESTERED_CALCIUM_ION_INTO_CYTOSOL	18	0.523688	0.954545	0.984253
1403	GO_POSITIVE_REGULATION_OF_CELLULAR_AMIDE_METABOLIC_PROCESS	50	0.521914	0.971564	0.984125
1404	GO_REGULATION_OF_LIPID_TRANSPORT	21	0.521104	0.946124	0.983694
1405	GO_TUMOR_NECROSIS_FACTOR_SUPERFAMILY_CYTOKINE_PRODUCTION	36	0.520959	0.967581	0.983046
1406	GO_SPHINGOLIPID_BIOSYNTHETIC_PROCESS	26	0.516856	0.962042	0.983637
1407	GO_MONOVALENT_INORGANIC_CATION_HOMEOSTASIS	33	0.515715	0.964504	0.983311
1408	GO_POSITIVE_REGULATION_OF_INTERLEUKIN_6_PRODUCTION	20	0.514179	0.949535	0.983124
1409	GO_GRANULOCYTE_MIGRATION	32	0.51354	0.970169	0.982657
1410	GO_PYRUVATE_METABOLIC_PROCESS	45	0.509456	0.973333	0.983235
1411	GO_ENDOPLASMIC_RETICULUM_TO_GOLGI_VESICLE_MEDIATED_TRANSPORT	46	0.500105	0.977778	0.985297
1412	GO_VESICLE_BUDDING_FROM_MEMBRANE	28	0.499873	0.968176	0.984667
1413	GO_RESPONSE_TO_STARVATION	44	0.49598	0.97981	0.985102
1414	GO_MEMBRANE_LIPID_BIOSYNTHETIC_PROCESS	34	0.488234	0.979294	0.986415
1415	GO_CELLULAR_RESPONSE_TO_DRUG	15	0.480743	0.953652	0.987474
1416	GO_REGULATION_OF_RNA_SPLICING	38	0.477331	0.990196	0.987519
1417	GO_MATERNAL_PROCESS_INVOLVED_IN_FEMALE_PREGNANCY	19	0.474371	0.979396	0.987478
1418	GO_NEUTROPHIL_CHEMOTAXIS	27	0.440127	0.987374	0.993035
1419	GO_MYELOID_LEUKOCYTE_MIGRATION	44	0.428026	0.994083	0.994029
1420	GO_VIRAL_GENE_EXPRESSION	54	0.400075	0.995408	0.996349

1421	GO_GRANULOCYTE_CHEMOTAXIS	29	0.361866	0.996183	0.998179

B)

#	NAME	SIZE	NES	NOM p-val	FDR q-val
1	GO_REGULATION_OF_AXONOGENESIS	41	-2.74759	<0.001	<0.001
2	GO_DENDRITIC_SPINE_DEVELOPMENT	29	-2.70039	<0.001	<0.001
3	GO_CELL_MORPHOGENESIS_INVOLVED_IN_NEURON_DIFFERENTIATION	140	-2.66732	<0.001	<0.001
4	GO_REGULATION_OF_DENDRITIC_SPINE_DEVELOPMENT	23	-2.63455	<0.001	<0.001
5	GO_RESPONSE_TO_BMP	29	-2.51239	<0.001	0.001208
6	GO_AXON_DEVELOPMENT	121	-2.46939	<0.001	0.001276
7	GO_CELL_PART_MORPHOGENESIS	164	-2.46491	<0.001	0.001329
8	GO_CELL_MORPHOGENESIS_INVOLVED_IN_DIFFERENTIATION	174	-2.45851	<0.001	0.00137
9	GO_REGULATION_OF_SYNAPSE_ASSEMBLY	22	-2.44752	<0.001	0.001412
10	GO_NEURON_PROJECTION_GUIDANCE	67	-2.40743	<0.001	0.002084
11	GO_DENDRITIC_SPINE_MORPHOGENESIS	20	-2.40742	<0.001	0.00191
12	GO_CELL_JUNCTION_ASSEMBLY	88	-2.4021	<0.001	0.001763
13	GO_CELL_JUNCTION_ORGANIZATION	155	-2.36485	<0.001	0.00321
14	GO_SYNAPSE_ASSEMBLY	35	-2.36315	<0.001	0.003116
15	GO_NEURON_PROJECTION_ORGANIZATION	33	-2.30102	<0.001	0.00383
16	GO_POSTSYNAPSE_ORGANIZATION	50	-2.29564	<0.001	0.003715
17	GO_REGULATION_OF_CELL_MORPHOGENESIS_INVOLVED_IN_DIFFEREN TIATION	71	-2.29543	<0.001	0.003614
18	GO_REGULATION_OF_DENDRITIC_SPINE_MORPHOGENESIS	16	-2.29313	<0.001	0.003424
19	GO_CELLULAR_COMPONENT_MORPHOGENESIS	188	-2.27445	<0.001	0.003969
20	GO_BONE_MINERALIZATION	27	-2.26092	<0.001	0.004531
21	GO_POSITIVE_REGULATION_OF_STRESS_ACTIVATED_PROTEIN_KINASE_ SIGNALING_CASCADE	42	-2.2575	<0.001	0.004405
22	GO_REGULATION_OF_EXTENT_OF_CELL_GROWTH	22	-2.25221	<0.001	0.004433
23	GO_POSITIVE_REGULATION_OF_JNK_CASCADE	35	-2.24562	<0.001	0.004606
24	GO_NEGATIVE_REGULATION_OF_AXONOGENESIS	15	-2.2158	<0.001	0.005842
25	GO_RETINA_DEVELOPMENT_IN_CAMERA_TYPE_EYE	28	-2.21057	<0.001	0.006017
26	GO_SPINAL_CORD_DEVELOPMENT	22	-2.20429	<0.001	0.006196
27	GO_SYNAPSE_ORGANIZATION	100	-2.1887	<0.001	0.006673
28	GO_SMOOTH_MUSCLE_CELL_MIGRATION	20	-2.17847	<0.001	0.006563
29	GO_NEGATIVE_REGULATION_OF_DEVELOPMENTAL_GROWTH	26	-2.15662	0.004545	0.007667
30	GO_POSITIVE_REGULATION_OF_ACTIN_FILAMENT_BUNDLE_ASSEMBLY	19	-2.15299	<0.001	0.007714
31	GO_DENDRITE_DEVELOPMENT	60	-2.14141	<0.001	0.008426
32	GO_REGULATION_OF_POSTSYNAPSE_ORGANIZATION	31	-2.13896	<0.001	0.008391
33	GO_HOMOPHILIC_CELL_ADHESION_VIA_PLASMA_MEMBRANE_ADHESI ON_MOLECULES	23	-2.13288	<0.001	0.008663
34	GO_REGULATION_OF_SYSTEMIC_ARTERIAL_BLOOD_PRESSURE	19	-2.13136	<0.001	0.008515
35	GO_NEGATIVE_REGULATION_OF_CELLULAR_COMPONENT_MOVEMENT	77	-2.12356	<0.001	0.008822
36	GO_CELL_CELL_JUNCTION_ORGANIZATION	39	-2.11645	<0.001	0.00926
37	GO_REGULATION_OF_DENDRITE_MORPHOGENESIS	23	-2.10341	<0.001	0.009995
38	GO_POSITIVE_REGULATION_OF_AXONOGENESIS	18	-2.09949	<0.001	0.010198

39	GO_CELL_MORPHOGENESIS	239	-2.09556	<0.001	0.010393
40	GO_NEURON_DEVELOPMENT	261	-2.09498	<0.001	0.010179
41	GO_POSITIVE_REGULATION_OF_DENDRITE_MORPHOGENESIS	16	-2.09471	<0.001	0.009936
42	GO_REGULATION_OF_REGULATED_SECRETORY_PATHWAY	38	-2.07195	<0.001	0.012185
43	GO_EXOCRINE_SYSTEM_DEVELOPMENT	15	-2.06975	<0.001	0.012149
44	GO_POSITIVE_REGULATION_OF_NEURON_DIFFERENTIATION	81	-2.06894	<0.001	0.011997
45	GO_POSITIVE_REGULATION_OF_SYNAPSE_ASSEMBLY	15	-2.06359	0.003559	0.012382
46	GO_REGULATION_OF_SYNAPSE_STRUCTURE_OR_ACTIVITY	56	-2.06342	<0.001	0.012119
47	GO_MESODERM_DEVELOPMENT	19	-2.05828	<0.001	0.012306
48	GO_MUSCLE_CELL_MIGRATION	22	-2.05624	<0.001	0.012266
49	GO_DENDRITE_MORPHOGENESIS	36	-2.05529	<0.001	0.012163
50	GO_CENTRAL_NERVOUS_SYSTEM_NEURON_DIFFERENTIATION	36	-2.0365	<0.001	0.01348
51	GO_EXTRACELLULAR_STRUCTURE_ORGANIZATION	79	-2.02527	<0.001	0.014846
52	GO_PROTEIN_LOCALIZATION_TO_PLASMA_MEMBRANE	74	-2.01022	<0.001	0.016907
53	GO_REGULATION_OF_DENDRITE_DEVELOPMENT	36	-2.00742	<0.001	0.016994
54	GO_AXON_EXTENSION	27	-1.99899	<0.001	0.017997
55	GO_APPENDAGE_DEVELOPMENT	42	-1.99623	<0.001	0.017961
56	GO_POSITIVE_REGULATION_OF_SYNAPTIC_TRANSMISSION	33	-1.99587	0.00565	0.017679
57	GO_REGULATION_OF_SYNAPTIC_VESICLE_EXOCYTOSIS	18	-1.98552	0.003676	0.018663
58	GO_ACTIN_FILAMENT_DEPOLYMERIZATION	22	-1.98492	<0.001	0.018588
59	GO_RESPIRATORY_SYSTEM_DEVELOPMENT	41	-1.9747	0.005747	0.019997
60	GO_JNK_CASCADE	51	-1.9632	<0.001	0.021452
61	GO_DEVELOPMENTAL_GROWTH_INVOLVED_IN_MORPHOGENESIS	44	-1.96314	<0.001	0.021106
62	GO_NEURON_DIFFERENTIATION	305	-1.95332	<0.001	0.02263
63	GO_KERATINOCYTE_DIFFERENTIATION	27	-1.9366	<0.001	0.02511
64	GO_NEURON_RECOGNITION	15	-1.93478	<0.001	0.025227
65	GO_ANTERIOR_POSTERIOR_PATTERN_SPECIFICATION	44	-1.93197	<0.001	0.025599
66	GO_GLAND_MORPHOGENESIS	30	-1.93025	<0.001	0.025568
67	GO_MUSCLE_ORGAN_MORPHOGENESIS	16	-1.92981	0.00692	0.025269
68	GO_PLATELET_DEGRANULATION	42	-1.92415	0.006757	0.026084
69	GO_ARTERY_DEVELOPMENT	18	-1.92268	<0.001	0.025913
70	GO_TRANSMEMBRANE_RECEPTOR_PROTEIN_SERINE_THREONINE_KINASE_SIGNALING_PATHWAY	74	-1.91907	<0.001	0.02633
71	GO_ENDOTHELIUM_DEVELOPMENT	24	-1.91793	<0.001	0.026111
72	GO_EPHRIN_RECEPTOR_SIGNALING_PATHWAY	25	-1.91445	0.004464	0.026611
73	GO_REGULATION_OF_NEURON_PROJECTION_DEVELOPMENT	113	-1.91121	<0.001	0.027015
74	GO_REGULATION_OF_NEUROTRANSMITTER_TRANSPORT	27	-1.90924	<0.001	0.027116
75	GO_FAT_CELL_DIFFERENTIATION	33	-1.90417	0.005587	0.027631
76	GO_REGULATION_OF_CELL_MORPHOGENESIS	119	-1.90374	<0.001	0.027452
77	GO_MULTICELLULAR_ORGANISMAL_SIGNALING	45	-1.89956	<0.001	0.028035
78	GO_CELL_CELL_JUNCTION_ASSEMBLY	27	-1.89646	<0.001	0.028377
79	GO_REGULATION_OF_SYNAPTIC_VESICLE_CYCLE	28	-1.89631	<0.001	0.028067
80	GO_REGULATION_OF_EXOCYTOSIS	48	-1.89327	<0.001	0.028476
81	GO_POSITIVE_REGULATION_OF_NEURON_PROJECTION_DEVELOPMENT	63	-1.89221	<0.001	0.028283
82	GO_CILIUM_OR_FLAGELLUM_DEPENDENT_CELL_MOTILITY	25	-1.88592	<0.001	0.029371

83	GO_BEHAVIOR	121	-1.87517	<0.001	0.031423
84	GO_REGULATION_OF_NEURON_DIFFERENTIATION	146	-1.86853	<0.001	0.03232
85	GO_ENDOTHELIAL_CELL_MIGRATION	53	-1.85447	<0.001	0.035225
86	GO_REGIONALIZATION	70	-1.85387	<0.001	0.035059
87	GO_POSITIVE_REGULATION_OF_CELL_MORPHOGENESIS_INVOLVED_IN_DIFFERENTIATION	41	-1.84788	0.006289	0.036058
88	GO_DEVELOPMENTAL_CELL_GROWTH	47	-1.8463	<0.001	0.035912
89	GO_REGULATION_OF_EPITHELIAL_CELL_MIGRATION	58	-1.8462	<0.001	0.035531
90	GO_REGULATION_OF_CELL_PROJECTION_ORGANIZATION	164	-1.84163	<0.001	0.036398
91	GO_REGULATION_OF_BLOOD_PRESSURE	34	-1.83854	<0.001	0.036826
92	GO_ICOSANOID_METABOLIC_PROCESS	28	-1.83716	0.014634	0.036772
93	GO_REGULATION_OF_CELL_JUNCTION_ASSEMBLY	44	-1.83667	0.005917	0.036586
94	GO_NEURON_PROJECTION_EXTENSION	36	-1.82644	<0.001	0.038985
95	GO_REGULATION_OF_ENDOTHELIAL_CELL_MIGRATION	40	-1.8151	<0.001	0.041868
96	GO_PATTERN_SPECIFICATION_PROCESS	89	-1.81383	<0.001	0.041729
97	GO_SPERM_MOTILITY	18	-1.81382	0.008299	0.041304
98	GO_CILIUM_MOVEMENT	27	-1.80629	0.009615	0.043125
99	GO_MORPHOGENESIS_OF_A_BRANCHING_STRUCTURE	41	-1.79933	0.005814	0.044595
100	GO_PROTEIN_LOCALIZATION_TO_CELL_JUNCTION	38	-1.79761	0.012048	0.044692
101	GO_POST_GOLGI_VESICLE_MEDIATED_TRANSPORT	26	-1.79557	<0.001	0.044905
102	GO_BIOLOGICAL_ADHESION	342	-1.78794	<0.001	0.046816
103	GO_SENSORY_SYSTEM_DEVELOPMENT	79	-1.78285	<0.001	0.04803
104	GO_AMEBOIDAL_TYPE_CELL_MIGRATION	102	-1.77895	<0.001	0.048715
105	GO_EPITHELIAL_CELL_DEVELOPMENT	45	-1.7644	0.007576	0.052943
106	GO_ACTIN_FILAMENT_BUNDLE_ORGANIZATION	43	-1.75781	<0.001	0.055229
107	GO_REGULATION_OF_CELLULAR_RESPONSE_TO_GROWTH_FACTOR_STIMULUS	56	-1.75661	<0.001	0.055131
108	GO_REGULATION_OF_RESPONSE_TO_WOUNDING	34	-1.75499	0.00578	0.055143
109	GO_SENSORY_ORGAN_MORPHOGENESIS	47	-1.75422	0.015152	0.055003
110	GO_ACTIVATION_OF_GTPASE_ACTIVITY	29	-1.7533	0.018605	0.054809
111	GO_CHONDROCYTE_DIFFERENTIATION	27	-1.7356	0.013636	0.061183
112	GO_REGULATION_OF_POSTSYNAPTIC_MEMBRANE_POTENTIAL	17	-1.73179	0.019685	0.062452
113	GO_PROTEOGLYCAN_BIOSYNTHETIC_PROCESS	17	-1.72828	0.021201	0.063315
114	GO_CHEMICAL_SYNAPTIC_TRANSMISSION_POSTSYNAPTIC	17	-1.72459	0.025735	0.064398
115	GO_BONE_MORPHOGENESIS	19	-1.72024	0.01626	0.065713
116	GO_REGULATION_OF_ACTIN_FILAMENT_BUNDLE_ASSEMBLY	28	-1.72005	0.004739	0.065241
117	GO_SOMATIC_STEM_CELL_POPULATION_MAINTENANCE	16	-1.71658	0.029412	0.066243
118	GO_ACTION_POTENTIAL	30	-1.71482	0.009479	0.066424
119	GO_APPENDAGE_MORPHOGENESIS	35	-1.71419	0.022857	0.066327
120	GO_REGULATION_OF_OSSIFICATION	40	-1.71147	0.018519	0.067051
121	GO_EMBRYONIC_APPENDAGE_MORPHOGENESIS	27	-1.71082	0.01005	0.066823
122	GO_POSITIVE_REGULATION_OF_CELL_JUNCTION_ASSEMBLY	24	-1.71006	0.004348	0.066522
123	GO_TAXIS	146	-1.70874	<0.001	0.066587
124	GO_ENDOCRINE_PROCESS	17	-1.70846	0.023411	0.066123
125	GO_SENSORY_PERCEPTION_OF_LIGHT_STIMULUS	34	-1.70842	0.010526	0.065598
126	GO_REGULATION_OF_DEVELOPMENTAL_GROWTH	78	-1.70672	<0.001	0.065865

127	GO_REGULATION_OF_BONE_MINERALIZATION	18	-1.70166	0.021127	0.067417
128	GO_ESTABLISHMENT_OF_PROTEIN_LOCALIZATION_TO_PLASMA_MEMBRANE	23	-1.69878	0.013636	0.067962
129	GO_OSSIFICATION	88	-1.69807	<0.001	0.06787
130	GO_POSITIVE_REGULATION_OF_SUPRAMOLECULAR_FIBER_ORGANIZATION	56	-1.69679	<0.001	0.067839
131	GO_NEUROGENESIS	388	-1.69444	<0.001	0.068452
132	GO_REGULATION_OF_WOUND_HEALING	31	-1.68797	0.010363	0.070481
133	GO_SODIUM_ION_TRANSPORT	42	-1.68677	0.028249	0.070442
134	GO_POSITIVE_REGULATION_OF_CELL_PROJECTION_ORGANIZATION	94	-1.68505	0.013699	0.070777
135	GO_REGULATION_OF_ACTIN_FILAMENT_ORGANIZATION	77	-1.67653	0.009434	0.07411
136	GO_NEGATIVE_REGULATION_OF_CELL_MORPHOGENESIS_INVOLVED_IN_DIFFERENTIATION	21	-1.67327	0.022989	0.07515
137	GO_MESONEPHROS_DEVELOPMENT	16	-1.66581	0.017301	0.078233
138	GO_NERVOUS_SYSTEM_PROCESS	188	-1.66524	<0.001	0.077955
139	GO_CARDIAC_CHAMBER_DEVELOPMENT	38	-1.66168	0.011299	0.079148
140	GO_BIOMINERALIZATION	33	-1.66064	0.015873	0.07926
141	GO_REGULATION_OF_CELL_SIZE	39	-1.65968	0.012422	0.079191
142	GO_ODONTOGENESIS	28	-1.6556	0.027933	0.080316
143	GO_PROTEIN_LOCALIZATION_TO_CELL_PERIPHERY	96	-1.65116	<0.001	0.081863
144	GO_RESPONSE_TO_MONOSACCHARIDE	48	-1.64376	0.005952	0.084485
145	GO_TISSUE_MIGRATION	78	-1.64313	<0.001	0.084174
146	GO_FORMATION_OF_PRIMARY_GERM_LAYER	23	-1.64272	0.036199	0.083764
147	GO_GASTRULATION	36	-1.64192	0.031646	0.083573
148	GO_SMAD_PROTEIN_SIGNAL_TRANSDUCTION	16	-1.63948	0.018315	0.084112
149	GO_CARDIAC_VENTRICLE_MORPHOGENESIS	15	-1.63923	0.037931	0.083717
150	GO_POSITIVE_REGULATION_OF_PROTEIN_BINDING	26	-1.63693	0.023041	0.084444
151	GO_ROOF_OF_MOUTH_DEVELOPMENT	22	-1.63442	0.028926	0.085146
152	GO_STRESS_FIBER_ASSEMBLY	25	-1.63156	0.029126	0.085802
153	GO_POSITIVE_REGULATION_OF_DENDRITE_DEVELOPMENT	21	-1.6308	0.024691	0.085625
154	GO_INTEGRIN_MEDIATED_SIGNALING_PATHWAY	23	-1.62932	0.01992	0.086008
155	GO_CELL_CELL_ADHESION_VIA_PLASMA_MEMBRANE_ADHESION_MOLECULES	41	-1.62841	<0.001	0.085945
156	GO_REGULATION_OF_CELLULAR_COMPONENT_MOVEMENT	246	-1.61939	<0.001	0.090081
157	GO_WOUND_HEALING	121	-1.60766	<0.001	0.095668
158	GO_PHOSPHATIDYLINOSITOL_METABOLIC_PROCESS	43	-1.60501	0.030303	0.096898
159	GO_NEGATIVE_REGULATION_OF_IMMUNE_EFFECTOR_PROCESS	34	-1.60217	0.02	0.097949
160	GO_ODONTOGENESIS_OF_DENTIN_CONTAINING_TOOTH	19	-1.59916	0.046154	0.098948
161	GO_REGULATION_OF_LIPASE_ACTIVITY	19	-1.59797	0.026923	0.09901
162	GO_REGULATION_OF_CHEMOTAXIS	48	-1.59498	0.013986	0.100148
163	GO_MAST_CELL_ACTIVATION	17	-1.59466	0.0369	0.099736
164	GO_PROTEIN_LOCALIZATION_TO_SYNAPSE	28	-1.59404	0.014085	0.099632
165	GO_CENTRAL_NERVOUS_SYSTEM_NEURON_DEVELOPMENT	21	-1.5912	0.032787	0.100819
166	GO_CONNECTIVE_TISSUE_DEVELOPMENT	62	-1.58616	<0.001	0.103316
167	GO_EPIDERMAL_CELL_DIFFERENTIATION	39	-1.58606	0.032432	0.102787
168	GO_EMBRYONIC_MORPHOGENESIS	113	-1.5822	<0.001	0.104397
169	GO_NEGATIVE_REGULATION_OF_RESPONSE_TO_EXTERNAL_STIMULUS	81	-1.57972	<0.001	0.105322

170	GO_ANATOMICAL_STRUCTURE_FORMATION_INVOLVED_IN_MORPHOGENESIS	247	-1.5778	<0.001	0.106006
171	GO_G_PROTEIN_COUPLED_RECEPTOR_SIGNALING_PATHWAY	167	-1.5771	<0.001	0.105689
172	GO_SKIN_DEVELOPMENT	49	-1.57701	<0.001	0.105141
173	GO_REGULATION_OF_COAGULATION	19	-1.57672	0.043307	0.104728
174	GO_MEMORY	21	-1.57498	0.037175	0.105258
175	GO_NEGATIVE_REGULATION_OF_ACTIN_FILAMENT_POLYMERIZATION	17	-1.57313	0.067138	0.105776
176	GO_REGULATION_OF_TRANS_SYNAPTIC_SIGNALING	99	-1.57296	<0.001	0.10525
177	GO_PROTEIN_LOCALIZATION_TO_CILIUM	19	-1.56888	0.030769	0.107067
178	GO_POSITIVE_REGULATION_OF_MAPK_CASCADE	119	-1.56798	<0.001	0.107034
179	GO_CYTOSKELETON_DEPENDENT_INTRACELLULAR_TRANSPORT	53	-1.5667	0.034483	0.107213
180	GO_LENS_DEVELOPMENT_IN_CAMERA_TYPE_EYE	18	-1.56523	0.053957	0.107384
181	GO_REGULATION_OF_ACTOMYOSIN_STRUCTURE_ORGANIZATION	27	-1.56463	0.030837	0.107203
182	GO_SEGMENTATION	19	-1.55963	0.028777	0.10975
183	GO_PROTEOGLYCAN_METABOLIC_PROCESS	20	-1.55292	0.036437	0.113459
184	GO_POSITIVE_REGULATION_OF_VASCULATURE_DEVELOPMENT	29	-1.55272	0.036199	0.112978
185	GO_PROTEIN_LOCALIZATION_TO_POSTSYNAPSE	16	-1.5515	0.074074	0.113081
186	GO_ADULT_BEHAVIOR	26	-1.55094	0.040909	0.112675
187	GO_SYNAPTIC_SIGNALING	146	-1.54554	<0.001	0.115677
188	GO_CELL_SUBSTRATE_JUNCTION_ORGANIZATION	26	-1.5443	0.022321	0.115914
189	GO_ACTIN_FILAMENT_ORGANIZATION	113	-1.54121	<0.001	0.117307
190	GO_PROTEIN_LOCALIZATION_TO_CELL_SURFACE	20	-1.54033	0.037879	0.117268
191	GO_REGULATION_OF_STRESS_ACTIVATED_PROTEIN_KINASE_SIGNALING_CASCADE	61	-1.53849	0.016807	0.11766
192	GO_REGULATION_OF_NOTCH_SIGNALING_PATHWAY	24	-1.5334	0.056522	0.120549
193	GO_RESPONSE_TO_TRANSFORMING_GROWTH_FACTOR_BETA	59	-1.53315	0.025641	0.120028
194	GO_MULTICELLULAR_ORGANISM_GROWTH	37	-1.53041	0.028249	0.121126
195	GO_ADULT_LOCOMOTORY_BEHAVIOR	18	-1.52504	0.043137	0.123856
196	GO_NEGATIVE_REGULATION_OF_EPITHELIAL_CELL_MIGRATION	16	-1.52268	0.059028	0.124915
197	GO_CELLULAR_RESPONSE_TO_CARBOHYDRATE_STIMULUS	30	-1.5176	0.029557	0.127585
198	GO_POSITIVE_REGULATION_OF_PROTEIN_LOCALIZATION_TO_CELL_PERIPHERY	16	-1.51555	0.076923	0.128441
199	GO_REGULATION_OF_GLYCOPROTEIN_METABOLIC_PROCESS	15	-1.51382	0.075601	0.129241
200	GO_CIRCULATORY_SYSTEM_PROCESS	111	-1.51245	0.017241	0.129738
201	GO_SKELETAL_SYSTEM_MORPHOGENESIS	47	-1.51172	0.007634	0.129625
202	GO_CELLULAR_GLUCOSE_HOMEOSTASIS	32	-1.50119	0.04186	0.136752
203	GO_REGULATION_OF_MULTICELLULAR_ORGANISM_GROWTH	17	-1.4968	0.065217	0.139524
204	GO_PHENOL_CONTAINING_COMPOUND_METABOLIC_PROCESS	22	-1.49488	0.075099	0.14021
205	GO_REGULATION_OF_SUPRAMOLECULAR_FIBER_ORGANIZATION	100	-1.49433	<0.001	0.139904
206	GO_POSITIVE_REGULATION_OF_LIPID_METABOLIC_PROCESS	29	-1.49397	0.061135	0.139442
207	GO_RESPONSE_TO_WOUNDING	147	-1.49049	<0.001	0.141338
208	GO_CARTILAGE_DEVELOPMENT	44	-1.4901	0.032258	0.141029
209	GO_REGULATION_OF_CELL_DEVELOPMENT	226	-1.48908	<0.001	0.141083
210	GO_REGULATION_OF_SMALL_GTPASE_MEDIATED_SIGNAL_TRANSDUCTION	94	-1.48802	<0.001	0.141206
211	GO_REGULATION_OF_NERVOUS_SYSTEM_DEVELOPMENT	217	-1.48704	<0.001	0.141233
212	GO_CAMERA_TYPE_EYE_DEVELOPMENT	65	-1.48678	0.024793	0.140771

213	GO_ENZYME_LINKED_RECEPTOR_PROTEIN_SIGNALING_PATHWAY	251	-1.48528	<0.001	0.141214
214	GO_CYTOPLASMIC_MICROTUBULE_ORGANIZATION	17	-1.48507	0.065134	0.140716
215	GO_REGULATION_OF_MEMBRANE_POTENTIAL	85	-1.48368	0.013333	0.141008
216	GO_G_PROTEIN_COUPLED_RECEPTOR_SIGNALING_PATHWAY_COUPLED_TO_CYCLIC_NUCLEOTIDE_SECOND_MESSENGER	46	-1.48352	0.042857	0.140499
217	GO_DIGESTIVE_SYSTEM_DEVELOPMENT	31	-1.48093	0.037209	0.141741
218	GO_RESPONSE_TO_GROWTH_FACTOR	158	-1.48045	<0.001	0.141588
219	GO_RESPONSE_TO_CARBOHYDRATE	51	-1.47721	0.037594	0.143355
220	GO_LOCOMOTORY_BEHAVIOR	45	-1.47695	0.057554	0.142893
221	GO_SKIN_EPIDERMIS_DEVELOPMENT	16	-1.47511	0.077519	0.143667
222	GO_POSITIVE_REGULATION_OF_EPITHELIAL_CELL_MIGRATION	40	-1.47501	0.054545	0.14308
223	GO_VASCULATURE_DEVELOPMENT	158	-1.47184	<0.001	0.144976
224	GO_VACUOLAR_TRANSPORT	37	-1.46886	0.052326	0.146413
225	GO_NEPHRON_DEVELOPMENT	24	-1.46549	0.075893	0.148356
226	GO_POSITIVE_REGULATION_OF_EXOCYTOSIS	22	-1.46525	0.062069	0.147936
227	GO_INSULIN_SECRETION_INVOLVED_IN_CELLULAR_RESPONSE_TO_GLUCOSE_STIMULUS	17	-1.46504	0.0681	0.147466
228	GO_NON_MOTILE_CILIUM_ASSEMBLY	19	-1.46176	0.074219	0.149285
229	GO_REGULATION_OF_VASCULATURE_DEVELOPMENT	68	-1.46109	0.010417	0.149217
230	GO_FIBROBLAST_MIGRATION	16	-1.45987	0.091912	0.149602
231	GO_ANIMAL_ORGAN_MORPHOGENESIS	235	-1.45669	<0.001	0.151522
232	GO_BRANCHING_MORPHOGENESIS_OF_AN_EPITHELIAL_TUBE	32	-1.4558	0.089474	0.151508
233	GO_REGULATION_OF_OSTEOBLAST_DIFFERENTIATION	22	-1.45233	0.049383	0.153746
234	GO_TRANSFORMING_GROWTH_FACTOR_BETA_RECEPTOR_SIGNALING_PATHWAY	48	-1.44977	0.043478	0.155051
235	GO_HEART_PROCESS	55	-1.44898	0.048	0.154965
236	GO_CELL_CELL_ADHESION	206	-1.44853	0.05	0.154628
237	GO_CAMERA_TYPE_EYE_MORPHOGENESIS	27	-1.44807	0.043668	0.154311
238	GO_POSITIVE_REGULATION_OF_GTPASE_ACTIVITY	108	-1.44628	0.018519	0.154869
239	GO_POSITIVE_REGULATION_OF_CARBOHYDRATE_METABOLIC_PROCESS	23	-1.44479	0.047414	0.155501
240	GO_REGULATION_OF_GTPASE_ACTIVITY	130	-1.44397	0.02381	0.155441
241	GO_REGULATION_OF_CELLULAR_COMPONENT_SIZE	94	-1.43807	0.046875	0.159687
242	GO_CARDIAC_CONDUCTION	32	-1.4366	0.061856	0.160187
243	GO_BONE_REMODELING	21	-1.43584	0.071161	0.160134
244	GO_BONE_DEVELOPMENT	50	-1.43504	0.057325	0.160243
245	GO_SENSORY_ORGAN_DEVELOPMENT	108	-1.43308	<0.001	0.161342
246	GO_CHLORIDE_TRANSPORT	16	-1.43224	0.10453	0.161369
247	GO_FATTY_ACID_DERIVATIVE_METABOLIC_PROCESS	39	-1.42999	0.076923	0.162577
248	GO_KIDNEY_MORPHOGENESIS	17	-1.42713	0.076364	0.164317
249	GO_SIGNAL_RELEASE	129	-1.4252	<0.001	0.165395
250	GO_DIGESTION	19	-1.42472	0.104839	0.165109
251	GO_EYE_MORPHOGENESIS	33	-1.42409	0.058201	0.164949
252	GO_REGULATION_OF_PROTEIN_CONTAINING_COMPLEX_DISASSEMBLY	38	-1.42242	0.084848	0.165666
253	GO_PEPTIDE_HORMONE_SECRETION	59	-1.41978	0.025641	0.167209
254	GO_PHOSPHOLIPID_TRANSPORT	15	-1.41905	0.115132	0.167227
255	GO_SYNAPTIC_VESICLE_EXOCYTOSIS	24	-1.41905	0.084112	0.16658
256	GO_POSITIVE_REGULATION_OF_NERVOUS_SYSTEM_DEVELOPMENT	128	-1.41515	<0.001	0.169308

257	GO_POSITIVE_REGULATION_OF_ENDOCYTOSIS	25	-1.415	0.073171	0.168802
258	GO_POSITIVE_REGULATION_OF_ENDOTHELIAL_CELL_MIGRATION	27	-1.41443	0.090498	0.168612
259	GO_BLOOD_VESSEL_ENDOTHELIAL_CELL_MIGRATION	29	-1.41011	0.078341	0.171678
260	GO_MEMBRANE_DEPOLARIZATION	20	-1.40674	0.096	0.173997
261	GO_LYSOSOMAL_TRANSPORT	24	-1.40472	0.088983	0.17509
262	GO_COGNITION	62	-1.40157	0.021277	0.177248
263	GO_POSITIVE_REGULATION_OF_DEVELOPMENTAL_GROWTH	40	-1.40077	0.064103	0.177193
264	GO_LAMELLIPODIUM_ORGANIZATION	27	-1.39844	0.077982	0.178586
265	GO_REGULATION_OF_NEURONAL_SYNAPTIC_PLASTICITY	15	-1.39708	0.113971	0.179058
266	GO_HOMOTYPIC_CELL_CELL_ADHESION	23	-1.39602	0.097166	0.179343
267	GO_NEGATIVE_REGULATION_OF_MULTICELLULAR_ORGANISMAL_PROCESS	270	-1.39312	<0.001	0.181462
268	GO_LIPOPROTEIN_BIOSYNTHETIC_PROCESS	24	-1.392	0.102362	0.181878
269	GO_REGULATION_OF_VESICLE_MEDIATED_TRANSPORT	130	-1.39147	<0.001	0.181697
270	GO_POSITIVE_REGULATION_OF_ERK1_AND_ERK2_CASCADE	38	-1.39043	0.046358	0.181961
271	GO_REGULATION_OF_ANATOMICAL_STRUCTURE_MORPHOGENESIS	252	-1.38454	<0.001	0.186468
272	GO_HORMONE_METABOLIC_PROCESS	40	-1.38193	0.079096	0.188291
273	GO_INORGANIC_ANION_TRANSMEMBRANE_TRANSPORT	20	-1.38043	0.094118	0.188974
274	GO_REGULATION_OF_HORMONE_LEVELS	108	-1.37511	0.05	0.19311
275	GO_GOLGI_TO_PLASMA_MEMBRANE_TRANSPORT	17	-1.37494	0.132576	0.192609
276	GO_STRESS_ACTIVATED_PROTEIN_KINASE_SIGNALING_CASCADE	71	-1.37437	0.066667	0.192511
277	GO_RUFFLE_ORGANIZATION	21	-1.37366	0.104	0.192423
278	GO_FOCAL_ADHESION_ASSEMBLY	22	-1.37285	0.12987	0.192507
279	GO_MULTI_MULTICELLULAR_ORGANISM_PROCESS	45	-1.36895	0.058824	0.195532
280	GO_AMYLOID_PRECURSOR_PROTEIN_METABOLIC_PROCESS	23	-1.3637	0.084746	0.200026
281	GO_TUBE_MORPHOGENESIS	190	-1.36319	0.071429	0.199739
282	GO_POSITIVE_REGULATION_OF_CELL_DEVELOPMENT	130	-1.36126	0.03125	0.201152
283	GO_SKELETAL_SYSTEM_DEVELOPMENT	112	-1.35975	0.020408	0.202026
284	GO_LAMELLIPODIUM_ASSEMBLY	24	-1.35805	0.130252	0.202974
285	GO_NEGATIVE_REGULATION_OF_CELL_DEVELOPMENT	73	-1.35289	0.050633	0.207375
286	GO_SODIUM_ION_TRANSMEMBRANE_TRANSPORT	34	-1.35285	0.091892	0.206685
287	GO_REGULATION_OF_ANATOMICAL_STRUCTURE_SIZE	112	-1.35001	0.027778	0.208818
288	GO_REGULATION_OF_PROTEIN_DEPOLYMERIZATION	30	-1.34676	0.07489	0.211463
289	GO_NEGATIVE_REGULATION_OF_NERVOUS_SYSTEM_DEVELOPMENT	63	-1.34587	0.072072	0.211692
290	GO_SOMITE_DEVELOPMENT	19	-1.34418	0.121673	0.212771
291	GO_NOTCH_SIGNALING_PATHWAY	47	-1.344	0.067568	0.212189
292	GO_NEGATIVE_REGULATION_OF_CELL_GROWTH	49	-1.3438	0.064516	0.211663
293	GO_REGULATION_OF_TRANSMEMBRANE_RECEPTOR_PROTEIN_SERINE_THREONINE_KINASE_SIGNALING_PATHWAY	53	-1.3412	0.084967	0.213641
294	GO_VESICLE_MEDIATED_TRANSPORT_TO_THE_PLASMA_MEMBRANE	27	-1.34048	0.10396	0.213769
295	GO_NEGATIVE_REGULATION_OF_GROWTH	67	-1.33888	0.063158	0.214724
296	GO_NEGATIVE_REGULATION_OF_CELL_PROJECTION_ORGANIZATION	42	-1.33858	0.10625	0.21434
297	GO_NEGATIVE_REGULATION_OF_NEURON_DIFFERENTIATION	43	-1.338	0.093168	0.214298
298	GO_EMBRYONIC_PATTERN_SPECIFICATION	16	-1.3369	0.133858	0.214757
299	GO_BLOOD_VESSEL_MORPHOGENESIS	130	-1.33253	0.03125	0.218406
300	GO_NEGATIVE_REGULATION_OF_PROTEIN_KINASE_B_SIGNALING	15	-1.32856	0.137931	0.221972

301	GO_APICAL_JUNCTION_ASSEMBLY	16	-1.32721	0.142857	0.222691
302	GO_CARDIAC_VENTRICLE_DEVELOPMENT	27	-1.32562	0.113636	0.223523
303	GO_REGULATION_OF_SECRETION	164	-1.32131	0.033333	0.227537
304	GO_REGULATION_OF_SYSTEM_PROCESS	115	-1.31806	0.032258	0.230504
305	GO_ERBB_SIGNALING_PATHWAY	44	-1.31591	0.129412	0.231905
306	GO_HEART_MORPHOGENESIS	53	-1.31484	0.086207	0.232217
307	GO_NEGATIVE_REGULATION_OF_INFLAMMATORY_RESPONSE	33	-1.31198	0.135135	0.234508
308	GO_NEGATIVE_REGULATION_OF_EPITHELIAL_CELL_PROLIFERATION	40	-1.31085	0.112745	0.235099
309	GO_POSITIVE_REGULATION_OF_PEPTIDYL_SERINE_PHOSPHORYLATION	20	-1.31078	0.126482	0.23446
310	GO_SUPRAMOLECULAR_FIBER_ORGANIZATION	181	-1.30228	0.052632	0.243309
311	GO_NEGATIVE_REGULATION_OF_PROTEIN_CONTAINING_COMPLEX_ASSEMBLY	37	-1.30201	0.120879	0.242817
312	GO_CELL_MATRIX_ADHESION	57	-1.29981	0.082707	0.24466
313	GO_REGULATION_OF_MUSCLE_HYPERTROPHY	15	-1.29654	0.17603	0.247673
314	GO_DIGESTIVE_SYSTEM_PROCESS	16	-1.29239	0.17301	0.252019
315	GO_FILOPODIUM_ASSEMBLY	15	-1.29225	0.184116	0.251441
316	GO_NEURAL_CREST_CELL_DIFFERENTIATION	17	-1.29124	0.148014	0.251825
317	GO_EPIDERMIS_DEVELOPMENT	63	-1.28916	0.1	0.253435
318	GO_REGULATION_OF_LIPID_KINASE_ACTIVITY	16	-1.2866	0.169014	0.255782
319	GO_PHOSPHATIDYLINOSITOL_BIOSYNTHETIC_PROCESS	28	-1.28597	0.135135	0.25567
320	GO_NEGATIVE_REGULATION_OF_NEURON_PROJECTION_DEVELOPMENT	33	-1.28529	0.136364	0.255624
321	GO_UNSATURATED_FATTY_ACID_METABOLIC_PROCESS	27	-1.28263	0.169725	0.258394
322	GO_EPITHELIAL_CELL_PROLIFERATION	91	-1.28225	0.111111	0.257998
323	GO_REGULATION_OF_ACTIN_FILAMENT_BASED_PROCESS	110	-1.28221	0.037736	0.257256
324	GO_NEGATIVE_REGULATION_OF_CELLULAR_RESPONSE_TO_GROWTH_FACTOR_STIMULUS	36	-1.28096	0.167539	0.258033
325	GO_PEPTIDE_SECRETION	124	-1.27811	0.02439	0.260591
326	GO_ADIPOSE_TISSUE_DEVELOPMENT	16	-1.278	0.143836	0.259942
327	GO_MICROTUBULE_BASED_TRANSPORT	46	-1.27471	0.146341	0.263131
328	GO_POSITIVE_REGULATION_OF_CELL_DIFFERENTIATION	217	-1.27344	0.0625	0.263921
329	GO_PROTEIN_KINASE_B_SIGNALING	57	-1.26577	0.116071	0.2722
330	GO_IRON_ION_HOMEOSTASIS	22	-1.26373	0.168	0.27387
331	GO_REGULATION_OF_ACTIN_FILAMENT_LENGTH	56	-1.26097	0.137097	0.276442
332	GO_MESENCHYME_DEVELOPMENT	55	-1.25855	0.09322	0.278819
333	GO_SENSORY_PERCEPTION	92	-1.25337	0.092308	0.284668
334	GO_REGULATION_OF_PROTEIN_LOCALIZATION_TO_PLASMA_MEMBRANE	21	-1.24851	0.158996	0.290264
335	GO_CALCIUM_ION_IMPORT	16	-1.24814	0.185965	0.289965
336	GO_VESICLE_MEDIATED_TRANSPORT_IN_SYNAPSE	52	-1.24257	0.121951	0.296723
337	GO_CHEMOKINE_PRODUCTION	16	-1.24177	0.207273	0.296947
338	GO_COAGULATION	84	-1.24118	0.126582	0.296818
339	GO_REGULATION_OF_CELL_PROJECTION_ASSEMBLY	55	-1.23439	0.173554	0.305142
340	GO_MOLTING_CYCLE	18	-1.23279	0.1875	0.30638
341	GO_AMYLOID_BETA_METABOLIC_PROCESS	16	-1.23273	0.21223	0.305569
342	GO_CYTOSOLIC_TRANSPORT	39	-1.23259	0.144654	0.304849
343	GO_CARDIAC_SEPTUM_DEVELOPMENT	26	-1.22963	0.192488	0.307957

344	GO_POSITIVE_REGULATION_OF_CELLULAR_CARBOHYDRATE_METABOLIC_PROCESS	16	-1.22692	0.202703	0.31068
345	GO_REGULATION_OF_MAPK_CASCADE	171	-1.2246	<0.001	0.312924
346	GO_HORMONE_TRANSPORT	71	-1.22343	0.12963	0.313686
347	GO_KIDNEY_EPITHELIUM_DEVELOPMENT	24	-1.22141	0.195238	0.315739
348	GO_REGULATION_OF_MUSCLE_ADAPTATION	18	-1.22078	0.203509	0.315802
349	GO_REGULATION_OF_PEPTIDYL_SERINE_PHOSPHORYLATION	32	-1.21977	0.163743	0.316243
350	GO_CARDIAC_CHAMBER_MORPHOGENESIS	28	-1.21846	0.210526	0.317318
351	GO_EPIDERMAL_GROWTH_FACTOR_RECEPTOR_SIGNALING_PATHWAY	38	-1.21786	0.191083	0.317249
352	GO_HINDBRAIN_DEVELOPMENT	38	-1.21092	0.20202	0.326457
353	GO_SMOOTH_MUSCLE_CELL_PROLIFERATION	37	-1.20461	0.185897	0.334773
354	GO_NEPHRON_EPITHELIUM_DEVELOPMENT	19	-1.20413	0.214815	0.334588
355	GO_DETECTION_OF_ABIOTIC_STIMULUS	25	-1.20269	0.21875	0.335739
356	GO_POSITIVE_REGULATION_OF_SMALL_MOLECULE_METABOLIC_PROCESS	36	-1.20234	0.223958	0.335348
357	GO_REGULATION_OF_JUN_KINASE_ACTIVITY	19	-1.19969	0.255474	0.338149
358	GO_NEUROTRANSMITTER_SECRETION	36	-1.19956	0.207865	0.337444
359	GO_UROGENITAL_SYSTEM_DEVELOPMENT	65	-1.19904	0.193878	0.337278
360	GO_POSITIVE_REGULATION_OF_TRANSMEMBRANE_RECEPTOR_PROTEIN_SERINE_THREONINE_KINASE_SIGNALING_PATHWAY	22	-1.19732	0.228571	0.339107
361	GO_TRANSMEMBRANE_RECEPTOR_PROTEIN_TYROSINE_KINASE_SIGNALING_PATHWAY	180	-1.19559	0.222222	0.340615
362	GO_POSITIVE_REGULATION_OF_CELLULAR_COMPONENT_MOVEMENT	130	-1.19218	0.147059	0.345052
363	GO_MORPHOGENESIS_OF_EMBRYONIC_EPITHELIUM	35	-1.19104	0.201117	0.345872
364	GO_LIPOPROTEIN_METABOLIC_PROCESS	31	-1.19054	0.198068	0.345642
365	GO_VESICLE_LOCALIZATION	54	-1.19021	0.210526	0.345103
366	GO_STEROL_TRANSPORT	20	-1.18407	0.259414	0.353391
367	GO_LONG_CHAIN_FATTY_ACID_METABOLIC_PROCESS	29	-1.18386	0.234694	0.352753
368	GO_EPITHELIAL_CELL_APOPTOTIC_PROCESS	22	-1.18343	0.241803	0.352375
369	GO_POSITIVE_REGULATION_OF_NIK_NF_KAPPAB_SIGNALING	28	-1.18261	0.233333	0.352595
370	GO_RENAL_SYSTEM_PROCESS	31	-1.18071	0.214953	0.354603
371	GO_REGULATION_OF_BLOOD_CIRCULATION	54	-1.17496	0.214815	0.3626
372	GO_COLLAGEN_METABOLIC_PROCESS	19	-1.16976	0.25098	0.369614
373	GO_ANION_TRANSMEMBRANE_TRANSPORT	62	-1.16314	0.235849	0.37888
374	GO_MUSCLE_ORGAN_DEVELOPMENT	91	-1.16065	0.22807	0.381827
375	GO_ACTIN_POLYMERIZATION_OR_DEPOLYMERIZATION	64	-1.15852	0.258621	0.384176
376	GO_REGULATION_OF_PEPTIDE_SECRETION	102	-1.15844	0.189655	0.383279
377	GO_ENDOSOMAL_TRANSPORT	73	-1.15664	0.2	0.385348
378	GO_CELLULAR_COMPONENT_ASSEMBLY_INVOLVED_IN_MORPHOGENESIS	27	-1.15555	0.270142	0.386227
379	GO_CELL_CELL_SIGNALING	383	-1.15409	<0.001	0.387659
380	GO_INOSITOL_LIPID_MEDIATED_SIGNALING	47	-1.15092	0.255034	0.391671
381	GO_REGULATION_OF_NEUROTRANSMITTER_LEVELS	50	-1.1482	0.245033	0.395073
382	GO_POSITIVE_REGULATION_OF_SECRETION	80	-1.14717	0.228916	0.395679
383	GO_REGULATION_OF_FAT_CELL_DIFFERENTIATION	19	-1.14589	0.259259	0.396747
384	GO_POSITIVE_REGULATION_OF_LIPASE_ACTIVITY	15	-1.14567	0.273381	0.396112
385	GO_ERK1_AND_ERK2_CASCADE	65	-1.14221	0.261682	0.400865
386	GO_CELLULAR_RESPONSE_TO_ACID_CHEMICAL	22	-1.13714	0.291139	0.408376

387	GO_POSITIVE_REGULATION_OF_PEPTIDE_SECRETION	53	-1.1338	0.240876	0.412892
388	GO_ORGANIC_HYDROXY_COMPOUND_TRANSPORT	41	-1.13365	0.264151	0.412062
389	GO_PLATELET_AGGREGATION	18	-1.1335	0.269962	0.411247
390	GO_SOMITOGENESIS	15	-1.12998	0.307432	0.415976
391	GO_ENDOSOME_TO_LYSOSOME_TRANSPORT	16	-1.12874	0.30292	0.417191
392	GO_LOCALIZATION_WITHIN_MEMBRANE	23	-1.12752	0.306034	0.418274
393	GO_SECONDARY_METABOLIC_PROCESS	16	-1.12709	0.287719	0.417896
394	GO_REGULATION_OF_GLUCOSE_METABOLIC_PROCESS	30	-1.12579	0.259804	0.419193
395	GO_RECEPTOR_CLUSTERING	15	-1.12456	0.295374	0.420156
396	GO_POSITIVE_REGULATION_OF_INTRACELLULAR_SIGNAL_TRANSDUCTI ON	247	-1.12409	0.142857	0.419983
397	GO_REGULATION_OF_PEPTIDE_HORMONE_SECRETION	49	-1.12114	0.305344	0.423863
398	GO_RENAL_TUBULE_DEVELOPMENT	17	-1.12042	0.318021	0.423992
399	GO_SPECIFICATION_OF_SYMMETRY	27	-1.1164	0.33	0.429842
400	GO_REGULATION_OF_PHOSPHOLIPASE_ACTIVITY	15	-1.11354	0.320558	0.433579
401	GO_POSITIVE_REGULATION_OF_ANIMAL_ORGAN_MORPHOGENESIS	15	-1.11251	0.300971	0.434294
402	GO_STRIATED_MUSCLE_CONTRACTION	27	-1.10953	0.285024	0.438433
403	GO_LIPID_HOMEOSTASIS	23	-1.10704	0.329004	0.44172
404	GO_INSULIN_SECRETION	52	-1.10475	0.29932	0.444591
405	GO_TISSUE_REMODELING	41	-1.09806	0.303448	0.455397
406	GO_VACUOLE_ORGANIZATION	35	-1.09772	0.333333	0.454877
407	GO_PLATELET_ACTIVATION	53	-1.09277	0.298387	0.462541
408	GO_PEPTIDYL_TYROSINE_MODIFICATION	95	-1.08994	0.26087	0.466459
409	GO_SYNAPTIC_TRANSMISSION_GLUTAMATERGIC	15	-1.08831	0.352518	0.46835
410	GO_SYNAPTIC_VESICLE_RECYCLING	28	-1.08644	0.369668	0.470782
411	GO_CELL_SUBSTRATE_ADHESION	85	-1.08631	0.318182	0.469894
412	GO_PROTEIN_PROCESSING	48	-1.08426	0.316901	0.472481
413	GO_TISSUE_HOMEOSTASIS	62	-1.08151	0.30597	0.476393
414	GO_POLYSACCHARIDE_BIOSYNTHETIC_PROCESS	22	-1.08102	0.334728	0.476114
415	GO_RHO_PROTEIN_SIGNAL_TRANSDUCTION	30	-1.07918	0.339713	0.478474
416	GO_REGULATION_OF_RHO_PROTEIN_SIGNAL_TRANSDUCTION	19	-1.07788	0.333333	0.479635
417	GO_CARDIAC_MUSCLE_CONTRACTION	23	-1.07581	0.362069	0.482219
418	GO_REGULATION_OF_HORMONE_SECRETION	57	-1.07471	0.3125	0.483204
419	GO_REGULATION_OF_MULTI_ORGANISM_PROCESS	24	-1.07376	0.374468	0.483716
420	GO_NEGATIVE_REGULATION_OF_KINASE_ACTIVITY	69	-1.07184	0.366071	0.4861
421	GO_RECEPTOR_LOCALIZATION_TO_SYNAPSE	18	-1.07178	0.359684	0.485056
422	GO_REGULATION_OF_CELL_MATRIX_ADHESION	30	-1.07093	0.339713	0.485476
423	GO_CELL_FATE_COMMITMENT	48	-1.06898	0.343066	0.487794
424	GO_LYSOSOME_LOCALIZATION	21	-1.06876	0.39207	0.487079
425	GO_ORGANOPHOSPHATE_ESTER_TRANSPORT	23	-1.06865	0.35	0.486166
426	GO_POSITIVE_REGULATION_OF_CHEMOTAXIS	31	-1.06577	0.411765	0.490448
427	GO_RUFFLE_ASSEMBLY	16	-1.06399	0.378472	0.492527
428	GO_OSTEOBLAST_DIFFERENTIATION	49	-1.06394	0.383459	0.491482
429	GO_POSITIVE_REGULATION_OF_PROTEIN_KINASE_B_SIGNALING	35	-1.06083	0.327161	0.496074
430	GO_POLYSACCHARIDE_METABOLIC_PROCESS	34	-1.05692	0.4	0.502184

431	GO_INOSITOL_PHOSPHATE_METABOLIC_PROCESS	19	-1.05529	0.397727	0.504037
432	GO_REGULATION_OF_INSULIN_SECRETION	44	-1.05492	0.381579	0.503586
433	GO_NEGATIVE_REGULATION_OF_NEURON_DEATH	47	-1.05386	0.402778	0.504358
434	GO_CYCLIC_NUCLEOTIDE_MEDIATED_SIGNALING	39	-1.05231	0.41954	0.506
435	GO_NEGATIVE_REGULATION_OF_PROTEIN_POLYMERIZATION	20	-1.05142	0.381679	0.506426
436	GO_REGULATION_OF_CHOLESTEROL_METABOLIC_PROCESS	20	-1.04921	0.385593	0.509224
437	GO_NEGATIVE_REGULATION_OF_TRANSMEMBRANE_RECEPTOR_PROTEIN_SERINE_THREONINE_KINASE_SIGNALING_PATHWAY	29	-1.04328	0.434146	0.519161
438	GO_REGULATION_OF_NIK_NF_KAPPAB_SIGNALING	37	-1.04265	0.417989	0.519123
439	GO_POSITIVE_REGULATION_OF_LIPID_LOCALIZATION	15	-1.04183	0.388704	0.51944
440	GO_EPITHELIAL_TUBE_FORMATION	32	-1.04052	0.4	0.520778
441	GO_REGULATION_OF_BIOMINERALIZATION	21	-1.03998	0.403587	0.520546
442	GO_REGULATION_OF_ERBB_SIGNALING_PATHWAY	31	-1.03894	0.414634	0.52143
443	GO_REGULATION_OF_G_PROTEIN_COUPLED_RECEPTOR_SIGNALING_PATHWAY	31	-1.03448	0.380711	0.528872
444	GO_REGULATION_OF_TRANSPORTER_ACTIVITY	51	-1.03432	0.409722	0.527968
445	GO_NEGATIVE_REGULATION_OF_DEFENSE_RESPONSE	55	-1.02844	0.420168	0.537798
446	GO_POSITIVE_REGULATION_OF_PHOSPHATIDYLINOSITOL_3_KINASE_SIGNALING	24	-1.02299	0.4	0.546699
447	GO_REGULATION_OF_CARDIAC_CONDUCTION	16	-1.01786	0.411552	0.555795
448	GO_POSITIVE_REGULATION_OF_DNA_BINDING_TRANSCRIPTION_FACTOR_ACTIVITY	69	-1.01638	0.435185	0.557306
449	GO_NEUROTRANSMITTER_RECEPTOR_TRANSPORT	15	-1.01472	0.46	0.559282
450	GO_NEGATIVE_REGULATION_OF_VASCULATURE_DEVELOPMENT	35	-1.01394	0.471204	0.559483
451	GO_REGULATION_OF_CELLULAR_CARBOHYDRATE_METABOLIC_PROCESS	36	-1.01233	0.447059	0.561241
452	GO_ADENYLATE_CYCLASE_ACTIVATING_G_PROTEIN_COUPLED_RECEPTOR_SIGNALING_PATHWAY	26	-1.01091	0.443396	0.562886
453	GO_METAL_ION_TRANSPORT	166	-1.01038	0.517241	0.562735
454	GO_REGULATION_OF_CELL_ADHESION_MEDIATED_BY_INTEGRIN	16	-1.00916	0.419795	0.563825
455	GO_SCHWANN_CELL_DEVELOPMENT	16	-1.00737	0.452381	0.56595
456	GO_CARBOHYDRATE_HOMEOSTASIS	55	-1.00668	0.470149	0.565949
457	GO_POSITIVE_REGULATION_OF_SMALL_GTPASE_MEDIATED_SIGNAL_TRANSDUCTION	24	-1.00471	0.486607	0.568372
458	GO_REGULATION_OF_PROTEIN_LOCALIZATION_TO_CELL_PERIPHERY	28	-1.00406	0.461929	0.56834
459	GO_REGULATION_OF_ION_TRANSPORT	130	-1.00178	0.543478	0.571421
460	GO_REGULATION_OF_MYELOID_LEUKOCYTE_DIFFERENTIATION	25	-1.0002	0.479821	0.573199
461	GO_POSITIVE_REGULATION_OF_ACTIN_FILAMENT_POLYMERIZATION	30	-0.9958	0.440217	0.580547
462	GO_REGULATION_OF_PHOSPHOLIPID_METABOLIC_PROCESS	21	-0.99571	0.478632	0.579457
463	GO_ENERGY_RESERVE_METABOLIC_PROCESS	26	-0.98902	0.466368	0.59084
464	GO_MYOTUBE_DIFFERENTIATION	17	-0.98893	0.487805	0.589764
465	GO_CELLULAR_IRON_ION_HOMEOSTASIS	17	-0.98851	0.448718	0.589301
466	GO_REGULATION_OF_CARBOHYDRATE_METABOLIC_PROCESS	51	-0.98727	0.46875	0.590284
467	GO_NEGATIVE_REGULATION_OF_PROTEIN_CONTAINING_COMPLEX_DISASSEMBLY	23	-0.98313	0.450704	0.59704
468	GO_VASCULAR_ENDOTHELIAL_GROWTH_FACTOR_RECEPTOR_SIGNALING_PATHWAY	26	-0.98161	0.474178	0.598716
469	GO_POSITIVE_REGULATION_OF_NUCLEOTIDE_METABOLIC_PROCESS	15	-0.97876	0.480427	0.602938
470	GO_CELLULAR_GLUCAN_METABOLIC_PROCESS	27	-0.97867	0.488152	0.601815
471	GO_REGULATION_OF_GLIAL_CELL_DIFFERENTIATION	19	-0.97571	0.488	0.606057
472	GO_POSITIVE_REGULATION_OF_ENDOTHELIAL_CELL_PROLIFERATION	27	-0.97466	0.487047	0.60678

473	GO_MACROPHAGE_ACTIVATION	23	-0.97413	0.46748	0.606492
474	GO_TRANSITION_METAL_ION_TRANSPORT	26	-0.97365	0.484305	0.606104
475	GO_REGULATION_OF_SIGNALING_RECEPTOR_ACTIVITY	31	-0.97344	0.490099	0.605289
476	GO_REGULATION_OF_METAL_ION_TRANSPORT	72	-0.97225	0.533898	0.606304
477	GO_EXPORT_ACROSS_PLASMA_MEMBRANE	18	-0.97006	0.492424	0.609216
478	GO_REGULATION_OF_CELL_SUBSTRATE_ADHESION	45	-0.96867	0.550388	0.610361
479	GO_RETINA_HOMEOSTASIS	19	-0.96086	0.519713	0.624045
480	GO_CYTOKINE_SECRETION	26	-0.95643	0.521552	0.631092
481	GO_MYELOID_LEUKOCYTE_DIFFERENTIATION	45	-0.95407	0.539568	0.634288
482	GO_ANION_TRANSPORT	123	-0.94928	0.619048	0.641671
483	GO_REGULATION_OF_POTASSIUM_ION_TRANSPORT	19	-0.94883	0.524904	0.641218
484	GO_CLATHRIN_DEPENDENT_ENDOCYTOSIS	18	-0.94692	0.533569	0.643594
485	GO_POSITIVE_REGULATION_OF_SMOOTH_MUSCLE_CELL_PROLIFERATIO N	23	-0.94623	0.534979	0.643588
486	GO_NEGATIVE_REGULATION_OF_IMMUNE_RESPONSE	44	-0.94363	0.56	0.647131
487	GO_PRESYNAPTIC_ENDOCYTOSIS	24	-0.94108	0.53719	0.650391
488	GO_GLYCOSPHINGOLIPID_METABOLIC_PROCESS	15	-0.94016	0.523636	0.650682
489	GO_ENDOTHELIAL_CELL_PROLIFERATION	46	-0.93639	0.628571	0.656325
490	GO_ORGANIC_ANION_TRANSPORT	93	-0.93391	0.68254	0.659679
491	GO_ASTROCYTE_DIFFERENTIATION	18	-0.9331	0.53937	0.659837
492	GO_SECRETION	359	-0.92974	1	0.664646
493	GO_POSITIVE_REGULATION_OF_I_KAPPAB_KINASE_NF_KAPPAB_SIGNAL ING	57	-0.92854	0.605042	0.665456
494	GO_POSITIVE_REGULATION_OF_BLOOD_VESSEL_ENDOTHELIAL_CELL_M IGRATION	16	-0.92788	0.543396	0.66527
495	GO_ORGANIC_HYDROXY_COMPOUND_CATABOLIC_PROCESS	16	-0.92584	0.517123	0.667599
496	GO_REGULATION_OF_LEUKOCYTE_MIGRATION	46	-0.92315	0.612245	0.671346
497	GO_RECEPTOR_SIGNALING_PATHWAY_VIA_STAT	38	-0.92218	0.556213	0.671577
498	GO_PHOSPHOLIPID_METABOLIC_PROCESS	109	-0.92148	0.671875	0.671537
499	GO_REGULATION_OF_MUSCLE_SYSTEM_PROCESS	43	-0.91911	0.602649	0.674318
500	GO_EXOCYTOSIS	217	-0.9083	0.733333	0.692081
501	GO_INSULIN_RECEPTOR_SIGNALING_PATHWAY	35	-0.90582	0.605556	0.695087
502	GO_GLIAL_CELL_DEVELOPMENT	36	-0.9025	0.596591	0.699537
503	GO_POSITIVE_REGULATION_OF_NF_KAPPAB_TRANSCRIPTION_FACTOR_ ACTIVITY	52	-0.88477	0.637584	0.729261
504	GO_REGULATION_OF_LIPID_LOCALIZATION	26	-0.87497	0.632076	0.745067
505	GO_REGULATION_OF_PROTEIN_POLYMERIZATION	61	-0.87299	0.731707	0.747075
506	GO_INORGANIC_ANION_TRANSPORT	33	-0.86871	0.721649	0.752912
507	GO_LIPOSACCHARIDE_METABOLIC_PROCESS	24	-0.86548	0.654545	0.75695
508	GO_LIPID_LOCALIZATION	86	-0.86482	0.770115	0.756472
509	GO_REGULATION_OF_CELL_SHAPE	39	-0.86337	0.7	0.757372
510	GO_PERIPHERAL_NERVOUS_SYSTEM_DEVELOPMENT	27	-0.86309	0.674641	0.756342
511	GO_ACUTE_INFLAMMATORY_RESPONSE	22	-0.85883	0.675	0.761985
512	GO_REGULATION_OF_EPITHELIAL_CELL_APOPTOTIC_PROCESS	15	-0.85213	0.625	0.771274
513	GO_LEARNING	31	-0.84979	0.7	0.773486
514	GO_RESPONSE_TO_INSULIN	59	-0.84735	0.728571	0.776131
515	GO_INTERLEUKIN_6_PRODUCTION	35	-0.8447	0.714286	0.778856

516	GO_AMINO_ACID_TRANSPORT	27	-0.84328	0.705314	0.779712
517	GO_AUTOPHAGOSOME_ORGANIZATION	19	-0.84127	0.701754	0.781255
518	GO_CELLULAR_BIOGENIC_AMINE_METABOLIC_PROCESS	16	-0.83823	0.676471	0.784622
519	GO_CELL_ADHESION_MEDIATED_BY_INTEGRIN	19	-0.83765	0.641791	0.783961
520	GO_GLYCOGEN_BIOSYNTHETIC_PROCESS	15	-0.8373	0.66443	0.783074
521	GO_CELLULAR_PIGMENTATION	19	-0.83649	0.732852	0.782693
522	GO_PEPTIDYL_CYSTEINE_MODIFICATION	19	-0.8363	0.675889	0.781529
523	GO_REGULATION_OF_CELL_SUBSTRATE_JUNCTION_ORGANIZATION	16	-0.83484	0.661157	0.782337
524	GO_STEROID_HORMONE_MEDIATED_SIGNALING_PATHWAY	31	-0.82743	0.648352	0.791769
525	GO_LIPID_METABOLIC_PROCESS	339	-0.82641	>0.98	0.791759
526	GO_CELLULAR_RESPONSE_TO_INSULIN_STIMULUS	50	-0.82508	0.783582	0.792296
527	GO_RESPONSE_TO_CATECHOLAMINE	15	-0.82147	0.687708	0.796248
528	GO_PROTEIN_LOCALIZATION_TO_VACUOLE	17	-0.81706	0.698842	0.801459
529	GO_POSITIVE_REGULATION_OF_PROTEIN_POLYMERIZATION	37	-0.80894	0.790698	0.812192
530	GO_ENDOCRINE_SYSTEM_DEVELOPMENT	19	-0.80495	0.715385	0.816169
531	GO_REGULATION_OF_SPROUTING_ANGIOGENESIS	18	-0.79551	0.736434	0.827847
532	GO_REGULATION_OF_MACROPHAGE_ACTIVATION	15	-0.79176	0.690554	0.831519
533	GO_RESPIRATORY_GASEOUS_EXCHANGE_BY_RESPIRATORY_SYSTEM	15	-0.78767	0.749153	0.835584
534	GO_REGULATION_OF_PEPTIDYL_TYROSINE_PHOSPHORYLATION	59	-0.7855	0.880342	0.836878
535	GO_ACTIN_CYTOSKELETON_REORGANIZATION	22	-0.7843	0.743902	0.836876
536	GO_MYOBLAST_DIFFERENTIATION	19	-0.77801	0.762264	0.843662
537	GO_POSITIVE_REGULATION_OF_PEPTIDYL_TYROSINE_PHOSPHORYLATIO N	45	-0.77221	0.776398	0.849799
538	GO_IRON_ION_TRANSPORT	16	-0.7676	0.758007	0.854207
539	GO_REGULATION_OF_RECEPTOR_MEDIATED_ENDOCYTOSIS	24	-0.76266	0.815094	0.858749
540	GO_REGULATION_OF_CARBOHYDRATE_BIOSYNTHETIC_PROCESS	21	-0.7626	0.790698	0.857237
541	GO_INOSITOL_PHOSPHATE_MEDIATED_SIGNALING	16	-0.76182	0.764706	0.856643
542	GO_NEGATIVE_REGULATION_OF_PROTEIN_DEPHOSPHORYLATION	19	-0.75405	0.782787	0.864732
543	GO_ASSOCIATIVE_LEARNING	17	-0.73973	0.788	0.880059
544	GO_REGULATION_OF_PHOSPHATIDYLINOSITOL_3_KINASE_SIGNALING	33	-0.72754	0.851351	0.891905
545	GO_CELLULAR_RESPONSE_TO_CALCIUM_ION	19	-0.71557	0.857708	0.903197
546	GO_REGULATION_OF_CELLULAR_RESPONSE_TO_TRANSFORMING_GRO WTH_FACTOR_BETA_STIMULUS	31	-0.71519	0.835749	0.901946
547	GO_POSITIVE_REGULATION_OF_CELL_SUBSTRATE_ADHESION	24	-0.71284	0.826667	0.902689
548	GO_CELLULAR_CARBOHYDRATE_BIOSYNTHETIC_PROCESS	24	-0.71224	0.821277	0.901563
549	GO_REGULATION_OF_TRANSLATIONAL_INITIATION	22	-0.69902	0.886463	0.9132
550	GO_ENDODERM_DEVELOPMENT	18	-0.6979	0.808594	0.91264
551	GO_CELL_CELL_RECOGNITION	22	-0.69705	0.814815	0.911815
552	GO_POSITIVE_REGULATION_OF_LEUKOCYTE_MIGRATION	29	-0.68863	0.872807	0.918337
553	GO_REGULATION_OF_ENDOCYTOSIS	54	-0.67236	0.944056	0.93111
554	GO_OSTEOCLAST_DIFFERENTIATION	15	-0.67038	0.872549	0.931276
555	GO_NEGATIVE_REGULATION_OF_RESPONSE_TO_ENDOPLASMIC_RETICU LUM_STRESS	16	-0.65908	0.881119	0.938735
556	GO_NEGATIVE_REGULATION_OF_T_CELL_PROLIFERATION	19	-0.65323	0.875502	0.941668
557	GO_POSITIVE_REGULATION_OF_MUSCLE_CELL_DIFFERENTIATION	17	-0.65308	0.886731	0.940089
558	GO_LIPID_PHOSPHORYLATION	21	-0.6478	0.9	0.942572
559	GO_SPERM_EGG_RECOGNITION	15	-0.62004	0.886926	0.960766

560	GO_OLIGODENDROCYTE_DIFFERENTIATION	30	-0.61202	0.928934	0.963961
561	GO_SKELETAL_MUSCLE_CELL_DIFFERENTIATION	16	-0.60453	0.954545	0.966568
562	GO_CALCINEURIN_MEDIATED_SIGNALING	15	-0.60374	0.932432	0.965316
563	GO_CELLULAR_RESPONSE_TO_GLUCOSE_STARVATION	15	-0.59328	0.911184	0.969698
564	GO_MEMBRANE_LIPID_METABOLIC_PROCESS	45	-0.59023	0.975	0.96956
565	GO_SPHINGOLIPID_METABOLIC_PROCESS	36	-0.59005	0.945455	0.967948
566	GO_REGULATION_OF_CELLULAR_PH	23	-0.57118	0.965665	0.975423
567	GO_NEGATIVE_REGULATION_OF_LEUKOCYTE_PROLIFERATION	23	-0.56006	0.936652	0.978271
568	GO_REGULATION_OF_PH	23	-0.55255	0.97992	0.979655
569	GO_REGULATION_OF_LEUKOCYTE_CHEMOTAXIS	27	-0.54514	0.957082	0.980743
570	GO_TRANSLATIONAL_INITIATION	48	-0.53056	>0.98	0.983849
571	GO_CELLULAR_RESPONSE_TO_STARVATION	33	-0.51911	0.977654	0.985617
572	GO_ALTERNATIVE_MRNA_SPLICING_VIA_SPLICEOSOME	17	-0.46609	>0.98	0.995067
573	GO_GLYCOLIPID_BIOSYNTHETIC_PROCESS	16	-0.46394	>0.98	0.993651
574	GO_PROTEIN_TARGETING_TO_MEMBRANE	65	-0.43642	>0.98	0.99489
575	GO_CELL_PROJECTION_ORGANIZATION	394	---	---	>0.995

Table footnote: # = Rank by NES; Name = Gene Ontology name of the geneset; Size = the number of genes in the geneset; NES = Normalized Enrichment Score; NOM p-val = nominal P value (statistical significance of the observed ES relative to null distribution); FDR q-val = False Discovery Rate Q value (the P value has been adjusted for FDR).
