# Supplementary material for: Gene dysregulation in acute HIV-1 infection – early transcriptomic analysis reveals the crucial biological functions affected
Source: Front Cell Infect Microbiol. 2023 Apr 3;13:1074847. doi: 10.3389/fcimb.2023.1074847 (PMC10106835; doi:10.3389/fcimb.2023.1074847)
Supplement: Supplementary Table 5 — Intracellular gene expression changes correlated moderately with measured plasma proteins (significant results only, of forty-nine quantifiable soluble biomarkers). [file DataSheet_5.pdf]

Supplementary Table 5: Intracellular gene expression changes correlated moderately with measured plasma proteins (significant results only, of forty-nine quantifiable soluble biomarkers).

**Protein**	**Aliases**	**Gene (HGNC)**	**Spearman correlation coefficient**	**P value**
C-X-C motif chemokine 10	IP-10, SYCB10	CXCL10	0.701	0.000 ***
Tumor necrosis factor ligand superfamily member 6	Fas ligand, APTL, CD-95L	FASLG	0.462	0.001 **
CD27 antigen	TNFRSF7	CD27	0.401	0.004 **
Tumor necrosis factor receptor superfamily member 1B	TNFR2	TNFRSF1B	0.382	0.007 **
Tumor necrosis factor ligand superfamily member 10	TRAIL	TNFS10	0.374	0.008 **
Interleukin-2	TCGF	IL2	0.352	0.013 *
C-C motif chemokine 2	MCP1, HC11, MCAF	CCL2	0.329	0.021 *
C-X-C motif chemokine 9	MIG, CMK, SCYB9	CXCL9	0.300	0.036 *
Interleukin-15	-	IL15	0.292	0.042 *
Transforming growth factor beta	-	TGFB1	0.285	0.047 *
