# Supplementary figures and images for: Gene dysregulation in acute HIV-1 infection – early transcriptomic analysis reveals the crucial biological functions affected
Source: Front Cell Infect Microbiol. 2023 Apr 3;13:1074847. doi: 10.3389/fcimb.2023.1074847 (PMC10106835; doi:10.3389/fcimb.2023.1074847)

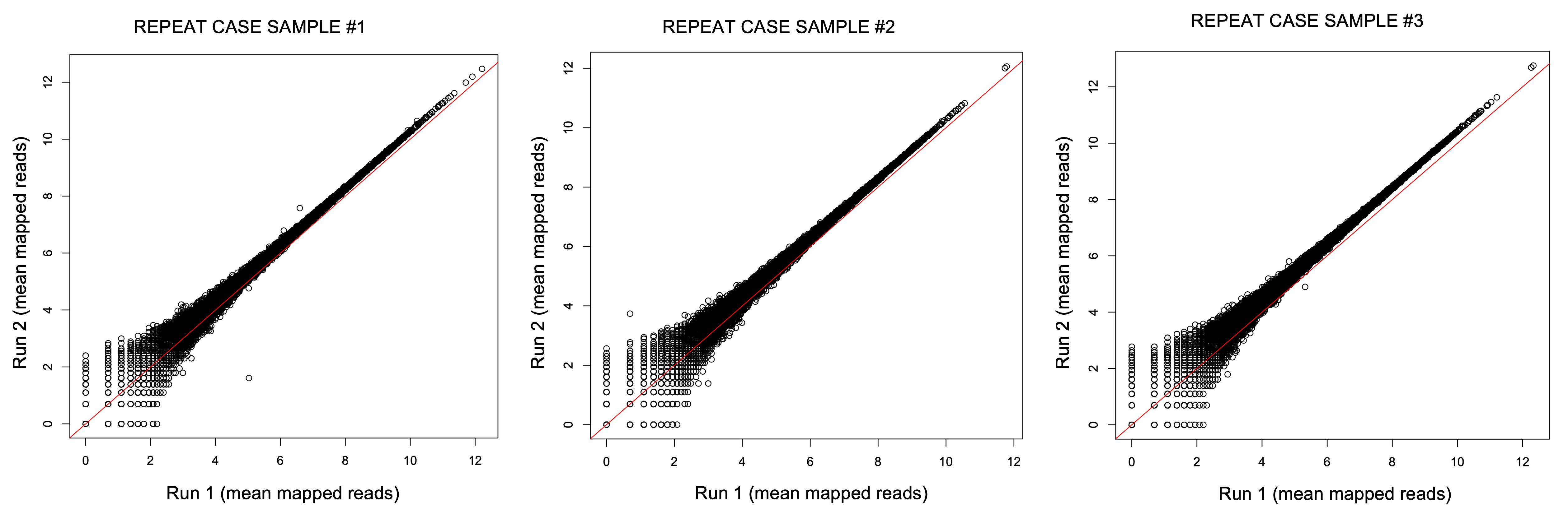

Supplement: Supplementary Figure 1 — Mean mapped reads of three case samples as sequenced on two different RNAseq batches. [file Image_1.png]

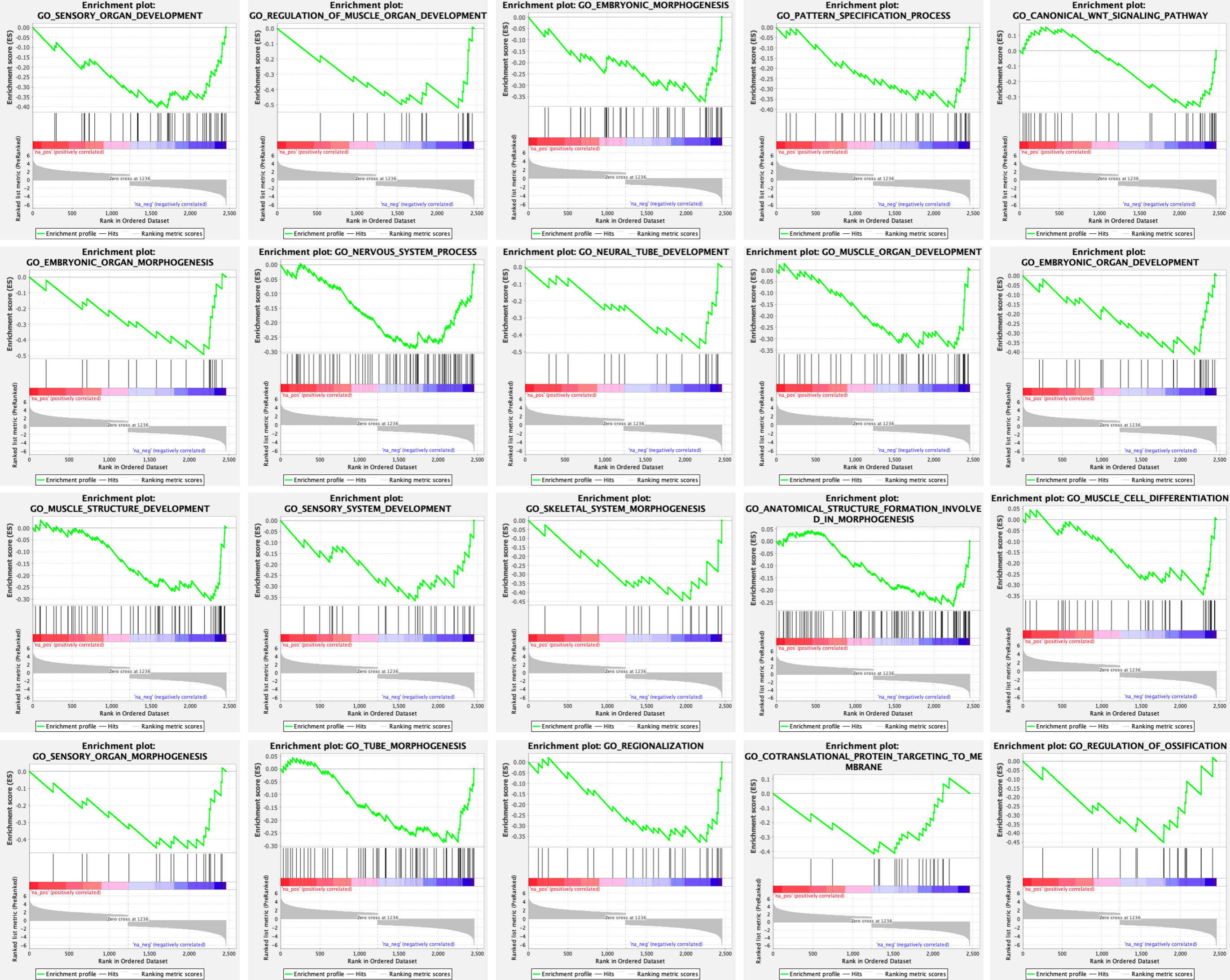

Supplement: Supplementary Figure 2 — Top 20 most up and down-regulated gene sets, associated with adjustment for cell populations. [file Image_2.pdf]
